# Using SXRF and LA-ICP-TOFMS to Explore Evidence of Treatment and Physiological Responses to Leprosy in Medieval Denmark

**DOI:** 10.3390/biology12020184

**Published:** 2023-01-25

**Authors:** Anastasia Brozou, Marcello A. Mannino, Stijn J. M. Van Malderen, Jan Garrevoet, Eric Pubert, Benjamin T. Fuller, M. Christopher Dean, Thomas Colard, Frédéric Santos, Niels Lynnerup, Jesper L. Boldsen, Marie Louise Jørkov, Andrei Dorian Soficaru, Laszlo Vincze, Adeline Le Cabec

**Affiliations:** 1 Department of Archaeology and Heritage Studies, Aarhus University, Moesgård Allé 20, 8270 Højbjerg, Denmark; 2 Department of Biology, University of Rome “Tor Vergata”, Via della Ricerca Scientifica 1, 00133 Rome, Italy; 3 Deutsches Elektronen-Synchrotron DESY, Notkestraße 85, D-22607 Hamburg, Germany; 4 Department of Chemistry, Ghent University, Campus Sterre, Krijgslaan 281-S12, 9000 Gent, Belgium; 5 Univ. Bordeaux, CNRS, MCC, PACEA, UMR 5199, F-33600 Pessac, France; 6 Géosciences Environnement Toulouse, UMR 5563, CNRS, Observatoire Midi-Pyrénées, 31400 Toulouse, France; 7 Department of Earth Sciences, Centre for Human Evolution Research, Natural History Museum, Cromwell Road, London SW7 5BD, UK; 8 Department of Cell and Developmental Biology, University College London, Gower Street, London WC1E 6BT, UK; 9 Department of Oral and Maxillofacial Radiology, University of Lille, Lille University Hospital, F-59000 Lille, France; 10 Department of Forensic Medicine, University of Copenhagen, Frederik V’s Vej 11, 2100 Copenhagen, Denmark; 11 Department of Forensic Medicine, University of Southern Denmark, Campusvej 55, 5230 Odense, Denmark; 12 ‘Francisc I. Rainer’ Institute of Anthropology, Romanian Academy, 050474 Bucharest, Romania; 13 Department of Chemistry, X-ray Microspectroscopy and Imaging Research Group (XMI), Ghent University, Krijgslaan 281 S12, 9000 Ghent, Belgium; 14 Department of Human Evolution, Max Planck Institute for Evolutionary Anthropology, Deutscher Platz 6, D-04103 Leipzig, Germany

**Keywords:** dental tissues, mineral imbalances, zinc, calcium, leprosy treatment, lead

## Abstract

**Simple Summary:**

Leprosy, a chronic infectious disease, leads to blood mineral imbalances: low levels of zinc, calcium, magnesium, and iron and high levels of copper. Interestingly, in late medieval Europe, minerals were used to treat leprosy. We investigated physiological responses to leprosy and possible evidence of treatment in dental tissues of leprosy sufferers from medieval Denmark and early 20th century Romania when multidrug therapy was not then yet invented. Using Synchrotron Fluorescence (SXRF) and laser ablation (LA-ICP-TOFMS), we show marked covariations in the zinc, calcium, and magnesium distributions, which are compatible with clinical studies but cannot be directly attributed to leprosy. Minerals used historically as a treatment for leprosy show no detectable intake (arsenic, mercury) or a diffuse distribution (lead) related to the daily consumption of contaminated water and food. Intense lead enrichments indicate acute incorporations, potentially through the administration of lead-enriched medication or the mobilization of lead from bone stores to the bloodstream during intense physiological stress related to leprosy. However, comparisons with a healthy control group are needed to ascertain these interpretations. The positive correlations and the patterns observed between lead and essential elements may indicate underlying pathophysiological conditions, demonstrating the potential of the two techniques for investigating diseases in past populations.

**Abstract:**

Leprosy can lead to blood depletion in Zn, Ca, Mg, and Fe and blood enrichment in Cu. In late medieval Europe, minerals were used to treat leprosy. Here, physiological responses to leprosy and possible evidence of treatment are investigated in enamel, dentine, and cementum of leprosy sufferers from medieval Denmark (n = 12) and early 20th century Romania (n = 2). Using SXRF and LA-ICP-TOFMS, 12 elements were mapped in 15 tooth thin sections, and the statistical covariation of paired elements was computed to assess their biological relevance. The results show marked covariations in the Zn, Ca, and Mg distributions, which are compatible with clinical studies but cannot be directly attributed to leprosy. Minerals used historically as a treatment for leprosy show no detectable intake (As, Hg) or a diffuse distribution (Pb) related to daily ingestion. Intense Pb enrichments indicate acute incorporations of Pb, potentially through the administration of Pb-enriched medication or the mobilization of Pb from bone stores to the bloodstream during intense physiological stress related to leprosy. However, comparisons with a healthy control group are needed to ascertain these interpretations. The positive correlations and the patterns observed between Pb and essential elements may indicate underlying pathophysiological conditions, demonstrating the potential of SXRF and LA-ICP-TOFMS for paleopathological investigations.

## 1. Introduction

Leprosy (a.k.a. Hansen’s disease) is a chronic infectious disease that is mainly encountered today in South-East Asia, Africa, and Latin America [1]. However, during the medieval period, the disease was highly prevalent in Europe. Caused by two bacteria (*Mycobacterium leprae* and *Mycobacterium lepromatosis*) [2,3], leprosy is expressed through a range of clinical manifestations, with tuberculoid leprosy and lepromatous leprosy being the two extremes of the spectrum. They usually represent, respectively, a paucibacillary (low bacterial load) and a multibacillary (high bacterial load) form [4]. The expression of leprosy depends on the immune status of each infected individual [5], and several factors that affect the immune system’s performance, such as poor living conditions, inadequate diets, and food shortages [6,7,8,9,10], have been considered in relation to the manifestation of the disease. There is a vicious cycle between malnutrition and infection [11,12]. On the one hand, nutritional deficiencies of vitamins and minerals lower the immune status, which increases the susceptibility to infections. On the other hand, however, the micronutrients are further depleted during infections by the invading pathogens, while the requirements are increased [13]. Several studies have reported mineral deficiencies or excesses in the blood serum of individuals suffering from leprosy (e.g., [14,15,16,17,18]).

Using Synchrotron X-ray Fluorescence (SXRF) and Laser Ablation-Inductively Coupled Plasma-Time of Flight Mass Spectrometry (LA-ICP-TOFMS), the present study investigates whether mineral imbalances of calcium (Ca), zinc (Zn), copper (Cu), iron (Fe), and magnesium (Mg) are recorded in dental hard tissues (enamel, dentine, and cementum) of leprosy sufferers from medieval Denmark and early 20th century Romania; an observation that could provide an insight into the physiological responses of leprosy sufferers from past populations. Due to repeated leprosy reactions, which are “acute exacerbations of the signs and symptoms of leprosy occurring during the natural course of the disease” and are the result of the body’s immune response to the bacteria [19], periodicity and variation in strength (depletions and enrichments) in the elemental distributions could be expected. Additional elements of interest are lead (Pb), mercury (Hg), and arsenic (As) since historical records attest to the use of these heavy metals to treat skin diseases, including leprosy, in late medieval and early 20th century Europe [20,21,22].

Unlike bone, dental tissues do not remodel once formed, constituting a record of life and health, as well as age at death [23,24,25,26,27,28]. Having different mineralization processes [29,30], enamel and primary dentine complete their formation during infancy and adolescence, while (cellular and acellular) cementum and secondary dentine develop throughout an individual’s life [29]. Consequently, when combined, enamel, dentine (primary and secondary), and cementum may provide information from as early as the first few months of life until the death of the individual under study. SXRF and synchrotron X-ray absorption spectroscopy have been widely employed to explore the normal and pathological distributions of trace elements in human tissues of modern populations (for a review, see [31]). By visualizing biochemical variations in thin-sectioned dental hard tissues, the present study is the first to explore the potential of SXRF and LA-ICP-TOFMS for the study of pathophysiological changes in past populations.

### Mineral Imbalances in Leprosy

Mineral elements are inorganic substances that are essential in living organisms for a variety of functions, such as the formation and maintenance of bone tissue [32]. They are classified as major (e.g., calcium, potassium, and phosphorus) or trace (e.g., manganese and copper) elements based on the required amounts, which are greater and lesser than 100 mg per day, respectively [33]. Mineral deficiencies or excesses, as well as interrelationships between different mineral elements (i.e., covariations with similar or opposite behaviors) related to diet or disease, lead to disturbances in normal metabolism and tissue structure [34,35,36].

By comparing blood serum levels between leprosy sufferers and a healthy control group, Rao et al. [37] reported depleted levels of Zn, Ca and Mg but elevated levels of Cu in the leprosy group. Such mineral imbalances in leprosy sufferers were also reported by several other studies (e.g., [14,16,38,39,40,41,42,43,44]). The depleted Zn levels correlate with an increased bacterial load [45] and not with the presence or absence of leprosy-related skin lesions [46]. Comparisons of serum levels between leprosy and non-leprosy patients with the same diet revealed that depleted Zn and Ca levels are associated with the disease and not with dietary depletions [47,48]. Moreover, by examining the serum Cu and Zn concentrations of leprosy patients before and during multidrug therapy, Sethi et al. [13] demonstrated a correlation between low Zn and high Cu serum levels with the severity and type of leprosy. Specifically, over the course of the treatment, the levels of both trace elements shifted toward the values of the control group [13].

The progressively depleted blood serum mineral levels in patients suffering from low to high bacterial loads could indicate the redistribution of the minerals from the blood to various tissues (e.g., liver), which is induced by the liberation of endogenous leukocyte mediators caused by the bacilli [49]. However, this has been reported in relation to acute, and not chronic, infections [50]. An alternative explanation could be the low absorption of the elements from the intestine; however, Kumar et al. [51] examined the small bowel in lepromatous leprosy patients and reported a normal absorptive function. Bhattacharya et al. [52] suggest that high blood levels of Cu in leprosy sufferers result from low levels of ascorbic acid (Vitamin C) that facilitate an increased absorption of Cu by the gut. Additionally, depleted serum levels of iron (Fe) in leprosy patients seem to result not from a failure to deliver Fe from Fe stores in the bone marrow but from an impaired circulation of Fe [53,54,55]. The accumulation of mineral elements in leprosy bacilli suggests that the bacteria retain elements originating from the host to cover their metabolic needs [13,56]. Furthermore, the values of oxidative stress in leprosy patients, which are potentially associated with the utilization of mineral elements by leprosy bacilli, are related to the duration of the infection, the bacterial load as well as the type of leprosy [57]. The imbalance of elemental levels in leprosy sufferers may also be related to the defense mechanisms of the hosts. This may either involve withholding nutrient metals from invading bacteria (known as “nutritional immunity”; [58,59]) and/or using the toxic properties of the mineral elements against the bacilli [60,61,62].

## 2. Materials and Methods

**Figure 1:**
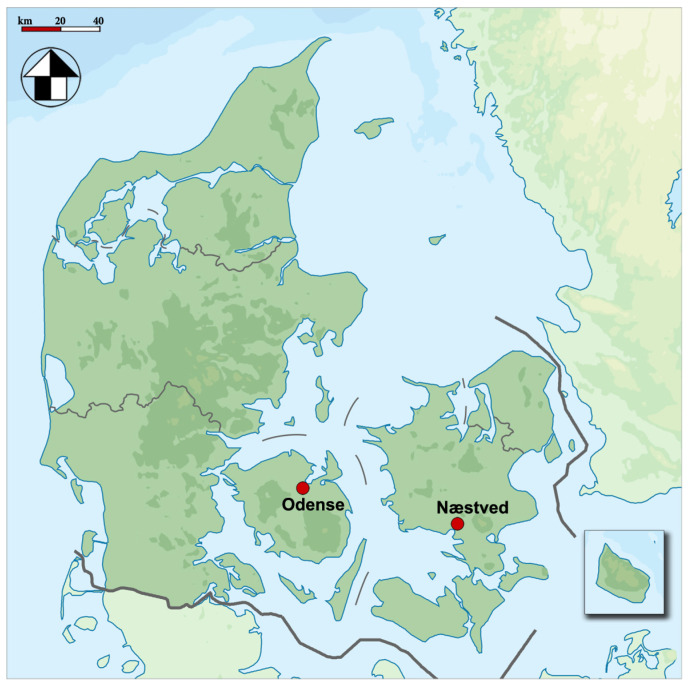
Map of Denmark depicting the location of Odense and Næstved (CC0 1.0 Universal; edited). Graphic programs used: Adobe Photoshop CS5.1 and Adobe Illustrator CS5.1.

Material included in this study derives from the cemeteries of two medieval leprosy hospitals, which were located outside of Næstved and Odense in Denmark (Figure 1). Both hospitals functioned from around 1260 CE (Næstved [63,64]) and 1270 CE (Odense [65,66]) until their dissolution in 1542 CE [67]. The leprosy hospitals at Næstved and Odense are historically documented institutions that have been archaeologically investigated [68,69,70,71,72]. Hundreds of skeletons found in their associated cemeteries have been previously studied [73,74,75].

Six individuals with osteological evidence of leprosy were selected from each site (Table S1). A permanent canine (C) and a permanent first molar (M1) was chosen from each individual (a total of 24 teeth) based on minimal wear and the absence of obvious pathologies, including carious lesions. These two tooth types were preferred as permanent first molars record information from as early as the perinatal period, while permanent canines from the first 4–5 months after birth [76]. Additionally, permanent teeth from two modern (beginning of the 20th century) Romanian individuals with documented evidence of a leprosy infection and who predate the advent of multidrug therapy were included for comparative purposes: the upper right canine and the lower right M1 of individual A1651; and the upper right canine and the lower right M2 of individual R1386. Authorization to work on these teeth was provided by the curating institutions (i.e., Odense Bys Museer, Medicinsk Museion, and the “Francisc I. Rainer” Institute of Anthropology, Romanian Academy).

Teeth were thin-sectioned using a Buehler Linear Precision Saw Isomet 5000 in the Histology Laboratory at PACEA (University of Bordeaux, France). Transmitted light micrographs (TLM) were used to assess the quality of dental tissue microstructure and select the best teeth for SXRF scanning. The visibility of growth increments was screened in both acellular and cellular cementum. Additional selection criteria were also considered, such as minimal visible taphonomic damages, including cracks, as well as tracks left by fungi. In total, 15 teeth from 10 different individuals (eight medieval—four from each site and two modern) were selected to undergo SXRF scanning (Table 1). Sex and age estimations, as well as radiocarbon dates for the eight medieval individuals, are presented in Table S2.

SXRF experiments were performed on the P06 Beamline [77,78], Petra III, at DESY (Deutsches Elektronen-Synchrotron, Hamburg, Germany). The scanning strategy followed a multiscale approach for each tooth (Table 1). First, a fast overview scan with a step size of 200 or 100 μm (dwell time: 10 ms) was acquired to check that the tooth section was well-centered in the field of view and to assess the overall signal-to-noise ratio of the elemental signature. Second, a higher resolution overview scan (10 μm; integration time: 2.5–4 ms) was used to visualize the elemental variation within the entire tooth section (i.e., enamel, dentine, pulp cavity, and cementum). Finally, based on prior observations of the tooth sections under the microscope and on the SXRF signal quality in the 10 µm scans, a small region of interest was selected in the cementum of 14 teeth and the secondary dentine of one tooth (Odense 533M) for scanning at 1–1.5 μm (integration time of 3 ms or 10 ms).

All dental tissues of permanent canines and first molars were targeted with the aim of acquiring elemental values reflecting the entire life period of the individuals under study. Indeed, while enamel and primary dentine start developing shortly before birth in permanent first molars or within the first 4–5 months of postnatal life in permanent canines, both tissues complete their formation between 7 and 10 years of age [79]. Secondary dentine and (cellular and acellular) cementum start forming incrementally once the root dentine area is completed and continue slowly developing until the death of the individual [29,30].

Two teeth that showed the best SXRF signal in cellular cementum (especially regarding the Zn pattern) were selected for comparative LA-ICP-TOFMS. LA-ICP-TOFMS offers high-speed multiplexed nuclide imaging [80,81] and quantitatively reliable images of the distribution of elements that were not quantifiable or reliably detectable with the SXRF setup (notably Pb). LA-ICP-TOFMS imaging was performed at the Department of Chemistry, Ghent University (Belgium) with an Iridia 193 nm ArF* excimer-based laser ablation system (Teledyne Photon Machines, Bozeman, MT, U.S.A.) coupled to an icpTOF 2R (TOFWERK AG, Thun, Switzerland) TOF-based ICP-MS instrument. During the calibration of the LA-ICP-TOFMS data, limits of detection (LOD) and quantitation (LOQ) were calculated, both in ppm, for each captured isotope (see [82]). LOD and LOQ values are reported in Table S3 for the isotopes selected to represent the chemical element it belongs to, based on their natural abundance, ionization potential, and lack of spectral interferences, which can induce some bias in the data. In summary, the isotope providing the cleanest signal was selected to represent each element of interest. Note that, compared to SXRF, LA-ICP-TOFMS shows better detection power in the higher-mass range, since detection power for SXRF is poor in the high-mass range. These are used as a guide for interpreting the results and for comparison with previously published studies. Note the LA-ICP-TOFMS scans left only a barely visible mark (a slight rectangular shadow) on the tooth section as a result of laser ablation. This was corrected with a fine polishing at 1 µm to recover an artefact-free thin section. Visualization and analysis of the SXRF and LA-ICP-TOFMS data were performed in HDIP v-1.3.3.1073 (Teledyne CETAC Technologies, Bozeman, MT, USA). With the aim to denoise the images, a 2D Gauss filter was applied, the kernel size of which is indicated in the Supplementary Information (Files S2 and S3).

To assess whether pairs of elements vary together or in opposition within statistical significance, Pearson’s r and Spearman’s ρ correlation coefficients were calculated on the concentration data measured along a transect on the high-resolution LA-ICP-TOFMS scans. The raw data for the transect of each LA-ICP-TOFMS scan are reported in Tables S4–S7. Note that Spearman’s “ρ” is henceforth spelled “rho” to avoid confusion with *p*-values. Statistical tests and graphs were performed in RStudio (version 1.3.1093 [83]) and Excel; *p*-values were computed with a significance level of α = 0.05. An extensive description of the statistical analysis, as well as of the tooth thin section preparation, the SXRF data acquisition and processing, and the LA-ICP-TOFMS imaging, is provided in the Supplementary Information (File S1).

## 3. Results

### 3.1. Copper (Cu) and Iron (Fe)

**Figure 2:**
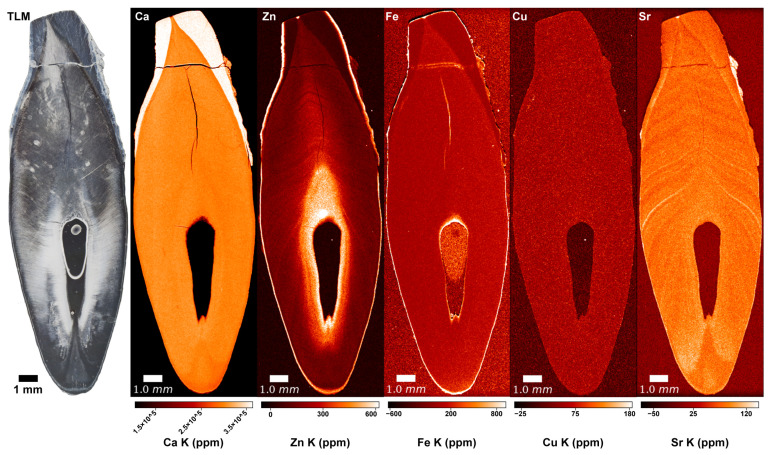
SXRF overview maps of Næstved 305C depicting the distributions of elements potentially impacted in leprosy, i.e., Ca, Zn, Fe, and Cu. The Sr map is shown for comparison with the Zn map since it also shows a pattern of alternating enrichments and depletions visible in the primary dentine. Ca, Fe, and Cu present uniform distributions. Contamination of Fe is visible on the surfaces of the tooth and within cracks. The Zn map reveals a rich pattern of enrichments and depletions in the primary dentine and a substantial enrichment at the outer enamel surface, the secondary dentine, and the cementum. Graphic programs used: Adobe Photoshop CS6 and Adobe Illustrator CS6.

**Figure 3:**
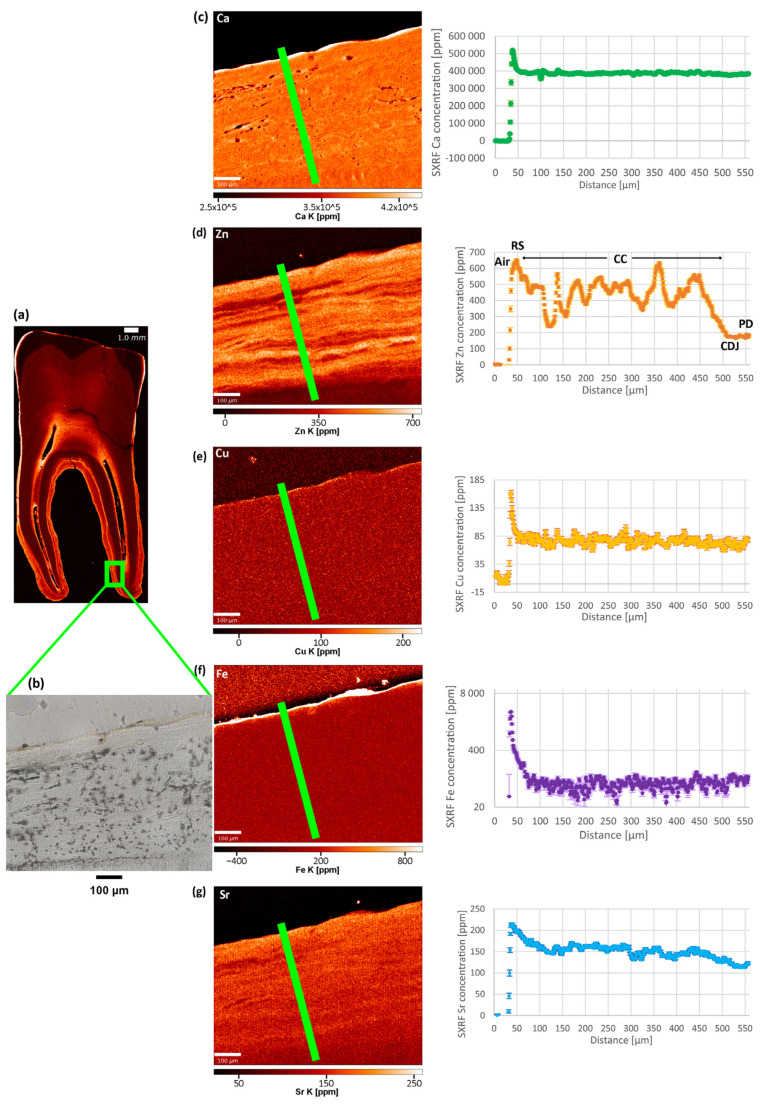
Distributions of elements potentially affected in leprosy (Ca, Zn, Cu, Fe) and Sr in Næstved 268M. The region of interest in the cellular cementum (green frame on the Zn overview map (**a**)) shows few identifiable increments in TLM (**b**). The SXRF data were collected on a scan at 1 µm, for each element of interest, along the green path (**c**–**g**). Elemental concentrations (in ppm) were then plotted against distance along the path in µm. As depicted on the Zn map, the path starts in the air (“Air”), reaches the root surface (RS), the cellular cementum (CC), the cementum-dentine junction (CDJ), and ends in the primary dentine (PD). Calcium (**c**) shows little variation, although a slight regular pattern may be visible on the Ca map. The Zn map (**d**) displays the clearest pattern of alternating enrichments and depletions, while Cu (**e**) and Fe (**f**) show uniform levels throughout the cementum. A subtle pattern recalling that of the Zn map can be observed in the Sr map (**g**). Note that a logarithmic scale was used to plot Fe, as the root surface enrichment at ~3000 ppm obscured the visibility of the pattern within the cementum at ~40–100 ppm. Graphic programs used: Adobe Photoshop CS6 and Adobe Illustrator CS6.

**Figure 4:**
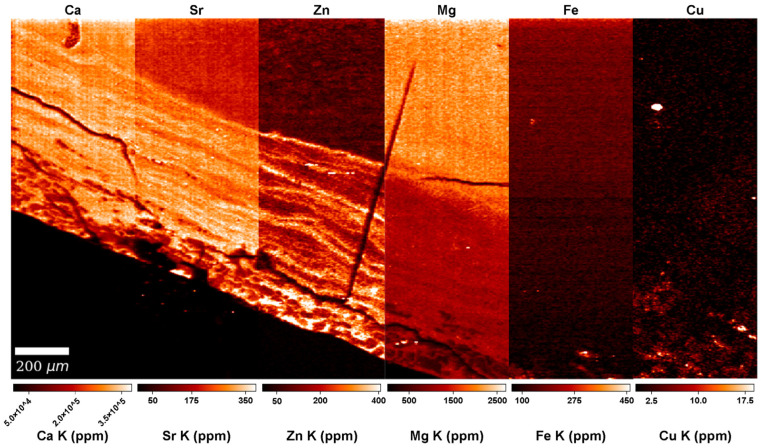
Calibrated LA-ICP-TOFMS high-resolution (2 µm) maps of the cellular cementum of Næstved 211C (See File S3) depicting the distributions of elements potentially impacted in leprosy, i.e., Ca, Zn, Mg, Fe, and Cu, as well as Sr. Note the weak signal in the Mg map, showing the same enrichment and depletion events as in the Ca, Zn, and Sr maps, with a positive, although weak, correlation to the Mg variations (rho = 0.45 for Ca, rho = 0.19 for Zn, and rho = 0.27 for Sr). Graphic programs used: Adobe Photoshop CS6 and Adobe Illustrator CS6.

No distinct pattern was observed in the Cu and Fe distributions in the SXRF overview maps of the modern and archaeological teeth. In overview and high-resolution SXRF maps (Figure 2 and Figure 3, File S2), Cu shows a uniform distribution within all tooth tissues, with baseline levels at ~100 ppm. In all teeth, the enamel is depleted in Fe, and dentine and cementum show values similar to the epoxy bond of the thin section, presumably reflecting a negligible Fe content close to ~0 ppm. Fe enrichment on the root surface and within dental cracks and the pulp chamber indicates exogenous contamination from the burial environment. High-resolution LA-ICP-TOFMS scans (File S3) reveal that in Odense 533M, Fe is at ~0 ppm in the acellular cementum, probably also in the cellular cementum, where the concentration is at ~600–700 ppm, which is similar to the epoxy of the thin section. Copper is <5 ppm in acellular cementum and probably at ~0 ppm in cellular cementum (concentrations similar to epoxy: ~10–20 ppm). The cellular cementum of Næstved 211C shows a considerable Fe enrichment of the surface (1400 ppm) and Fe and Cu enrichment of the sub-surface (Figure 4), respectively, of 200 ppm and ~15 ppm. The secondary dentine of Odense 533M has Cu levels close to ~2–4 ppm, while Fe reaches ~220 ppm, although this could be contamination as the epoxy shows similar values.

### 3.2. Calcium (Ca) and Magnesium (Mg)

The SXRF overview maps of the medieval Danish and modern Romanian teeth show the well-established pattern of Ca-rich enamel (Figure 2, File S2). The enriched Ca levels in enamel (ranging from ~370,000–400,000 ppm) reflect its higher calcification levels compared to dentine (ranging from ~300,000–~380,000 ppm). Dentine has a homogenous Ca distribution with slightly depleted Ca levels around the pulp cavity due to the presence of secondary dentine, as well as within the cementum and at the junction between cementum and primary dentine. Some teeth, among which the modern Romanian A1651C and R1386M2, and the archaeological teeth Næstved 6C, 305M and C and Odense 533M, present accentuated lines reflecting variations in the Ca levels, often located (and best visible) in the dentine above the roof of the pulp chamber. The higher resolution Ca maps reveal subtle alternating enrichments and depletions in the cementum of three teeth (Næstved 211M, Odense 533M, and R1386M; see also Figure 4 showing calibrated LA-ICP-TOFMS high-resolution maps in the cellular cementum of Næstved 211C).

The uncalibrated LA-ICP-TOFMS overview map of Mg acquired in Næstved 211C reveals that cementum is strongly Mg-depleted in comparison to dentine (File S3). At high-resolution (calibrated LA-ICP-TOFMS data), the Mg content in primary dentine ranges from 2000–2200 ppm, while the cellular cementum shows values fluctuating between 1000–1800 ppm (Figure 4). Interestingly, the Mg distribution generally follows the Ca and Zn distributions (rho = 0.45 and 0.19, respectively, *p* < 0.05, File S1). In Odense 533M, the cellular cementum contains Mg between ~1000–~1400 ppm, and these covary positively with Ca (rho = 0.47, *p* < 0.05; File S1) and Zn (rho = 0.50, *p* < 0.05). The secondary dentine is at ~1000 ppm Mg content, with no visible pattern of fluctuations. Ca and Zn show a moderate positive correlation with Mg in secondary dentine (rho = 0.79 and 0.55, respectively, *p* < 0.05; File S1). On the LA-ICP-TOFMS scan for acellular cementum of Odense 533M, the primary dentine contains 1000–1300 ppm, while the acellular cementum is Mg-enriched at 1800–2200 ppm. Note that this area of acellular cementum is affected by cracks, which, however, do not affect the reported concentrations, as they contain no Mg, thus, attesting to an endogenous origin of the Mg detected in the tooth tissues.

### 3.3. Zinc (Zn)

In all teeth, Zn content follows a similar distribution, with an enriched layer at the outer enamel surface and an accumulation in cementum and secondary dentine (Figure 2, see overview SXRF maps in File S2). In enamel, Zn is ~100 ppm with an enriched layer at ~650 ppm within the first tens of microns of the enamel surface, which has been interpreted in light of the process of enamel mineralization and maturation [84,85,86]. In dentine, the Zn baseline fluctuates between ~150–350 ppm, with marked alternating enrichments and depletions following the direction of the long-period growth increments. At higher resolution, a clear banding pattern is seen in the SXRF and LA-ICP-TOFMS Zn maps of cellular cementum (Figure 3 and Figure 4, Files S2 and S3). The secondary dentine seems to record this pattern slightly less clearly (range: ~200–600 ppm; File S2). In TLM, the border between secondary and primary dentine is visible due to the difference in orientation of the long-period growth lines in both tissues (Figure 2). However, due to their similar chemical composition, this border is difficult to identify in elemental mapping when looking at the Ca, P, and Sr maps (File S2). As seen by the Zn map in the secondary dentine of Odense 533M, Zn is the only element that allows this border to be identified with confidence (Figure 2, Files S2 and S3). No differences in the Zn (as well as Cu, Fe, Ca, and Mg) content and distribution are observed between sex and age groups or across chronological periods (Table S2).

### 3.4. Strontium (Sr)

Another observation concerns Sr (Figure 2, Figure 3 and Figure 4), which appears to covary positively with Ca, Mg, and Zn, although Sr is not an element known to bear any significance regarding leprosy. The strongest positive correlations are between Sr and Ca (in the cellular cementum of Næstved 211C, rho = 0.88, while in Odense 533M rho = 0.57, *p* < 0.05 for both; File S1) as well as Sr and Zn (cellular cementum: rho = 0.77 for Næstved 211C and rho = 0.78 for Odense 533M, *p* < 0.05 for both). Sr is slightly less strongly correlated with Mg (rho = 0.69 for Odense 533M and rho = 0.27 for Naestved 211C in the cellular cementum, rho = 0.86 and rho = −0.34 for the secondary dentine and the acellular cementum of Odense 533M). The correlations of Sr with these elements (also for all other pairs of elements concerned) are weak (although significant) in the acellular cementum since the growth increments and accentuated markings are harder to resolve and much more tightly packed in this tissue (File S1). In general, Sr shows a clear pattern of variation, mostly in the primary dentine and in the cellular cementum (Figure 3), which is attested to be an authentic biological signal since these Sr variations follow the direction of the growth increments.

### 3.5. Arsenic (As) and Mercury (Hg)

In the SXRF data, the fitting of the As signal was not good enough to provide reliable data (the second emission peak was missing to safely identify the elemental signature). In the uncalibrated high-resolution LA-ICP-TOFMS map of the cellular cementum of Næstved 211C (File S3), the As level in cementum and primary dentine is close to ~1 ppm with noisy values at ~2–4 ppm. Similarly, in Odense 533M, high-resolution maps (File S3) in the acellular cementum and secondary dentine show a background noise in the tooth tissues at 3–5 ppm (as opposed to ~0 ppm in epoxy), while As is not detected in the cellular cementum. However, with a LOD ≈ of 380 ppm and a LOQ ≈ of 1250 ppm (Table S3), this cannot be considered a reliable biological signal. Additionally, neither the SXRF nor LA-ICP-TOFMS setups were sensitive enough to detect Hg concentrations.

### 3.6. Lead (Pb)

**Figure 5:**
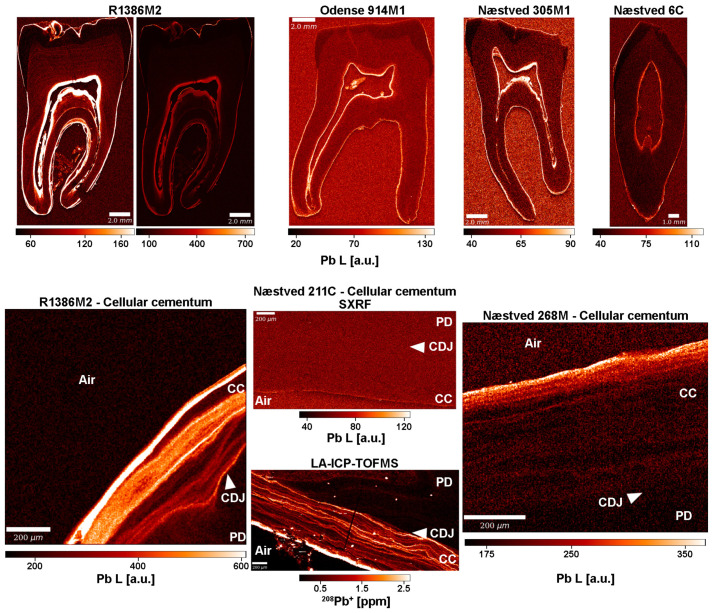
Uncalibrated SXRF Pb maps (detection of the Pb L lines) for the modern R1386M, Odense 914M, Næstved 305M, and 6C showing a preferential accumulation of Pb in the slow-forming secondary dentine and cementum (**top row**). Relative SXRF Pb enrichments and depletions are expressed in arbitrary units (“a.u.”). A very light alternation pattern of Pb is visible in the primary dentine of R1386M. This is confirmed at high-resolution (**bottom row**), where the cellular cementum of R1386M2 scanned in SXRF reveals a well-defined Pb pattern. In Næstved 268M, the surface of the cellular cementum is enriched in Pb, with a decreasing gradient involving an alternation of enrichments and depletions within the sub-surface. This Pb content fades away within the cellular cementum. Note that SXRF is not sensitive enough to resolve the Pb variation in the cellular cementum of Næstved 211C, while LA-ICP-TOFMS can quantify these variations with a high level of detail. “CC” stands for “cellular cementum,” “CDJ” for “cementodentine junction,” and “PD” for “primary dentine.” Graphic programs used: Adobe Photoshop CS6 and Adobe Illustrator CS6.

The SXRF datasets for Pb are uncalibrated and, thus, no quantitative values can be provided; instead, “arbitrary units” (“a.u.”) give an idea about the relative Pb distribution across specimens. Qualitative values are hereafter reported from direct measurements in the software HDIP, while the contrast has been adjusted in the Pb maps (File S2) to better reveal the Pb banding pattern. Overview SXRF maps (Figure 5, File S2) show that primary dentine (50–60 a.u.) is slightly enriched in Pb compared to enamel (~30–40 a.u.), while secondary dentine and cementum reach, overall, higher levels (60–70 a.u.). In Næstved 211C and 6C, the primary dentine contains 40–50 a.u. of Pb, while cementum and secondary dentine reach ~80 a.u.. Lead accumulation increases between dental tissues in Odense 914M (40–50 a.u. in enamel, ~60 a.u. in primary dentine, > 60–80 a.u. in cementum, and 120–140 a.u. in secondary dentine). The modern Romanian teeth show the greatest Pb accumulation with ~40–60 a.u. in enamel, 50–60 a.u. and 80 a.u. in the primary dentine of A1651M and R1386M, respectively, 80–100 a.u. in the secondary dentine of A1651M, while in R1386M it reaches 300 a.u. R1386M seems to concentrate Pb in cementum (Figure 5) with values of ~300 a.u. and peaks at 400 a.u., while in A1651M, the cementum is at ~100 a.u. with peaks at 120 a.u. An alternation of Pb enrichments and depletions is visible within the cementum and secondary dentine of R1386M (Figure 5, File S2). A consistent enrichment of Pb, probably linked to post-eruptive remineralization processes [87], is observed within the first 10 µm of the outer enamel surface in some of the teeth, reaching maximum amounts from ~500–700 a.u. in the M2 of R1386 and weaker values in the archaeological specimens (~80–100 a.u.). No obvious pattern could be observed between sexes or chronological periods in the specimens (Table S2) besides the strong enrichment seen in the modern teeth. The data do not reveal an accumulation of Pb in older individuals, as was suggested by Bercovitz and Laufer [88].

Using LA-ICP-TOFMS, quantitative Pb concentrations were measured in areas of interest in the cellular cementum of Næstved 211C (Figure 5), in the acellular and cellular cementum, and in the secondary dentine of Odense 533M (Table 1). The limits of the current instrumentation are 5 ppm (LOD) and 16 ppm (LOQ) (Table S3). Both teeth show a substantial Pb enrichment at the surface of the cellular cementum, at ~10 ppm in Næstved 211C and at 40–100 ppm in Odense 533M (File S3). No cracks are visible on the surface of the scanned area for Odense 533M, while the three cracks seen in the cementum of Næstved 211C do not show any diffusion of Pb in the inner layers, suggesting that these surface enrichments are biogenic and not taphonomic. Further Pb surface enrichment in Odense 533M involves the surface of the acellular cementum at ~60–80 ppm and the surface of secondary dentine (facing pulp cavity) at ~2–7 ppm (File S3). In Odense 533M, average background Pb levels range from ~0.1–0.3 ppm in primary dentine, ~0.2–0.4 ppm (with peaks at ~2–4 ppm) within the secondary dentine, to ~1.1–1.5 ppm (with a peak at ~8 ppm) in acellular cementum and ~1 ppm (peaks at ~2 ppm and a maximum at ~7 ppm) in cellular cementum.

## 4. Discussion

### 4.1. Exploring Leprosy-Induced Mineral Imbalances in Dental Tissues

Studies conducted on blood samples of modern leprosy patients revealed depleted levels of Zn, Ca, Mg and Fe and elevated levels of Cu compared to healthy control groups [37,39,41,44,55]. These mineral imbalances appeared to be correlated with the severity and type of leprosy but returned to the normal values of the control groups during multidrug leprosy therapy [13]. Here, SXRF and LA-ICP-TOFMS elemental mapping was used to determine if mineral imbalances in leprosy sufferers are imprinted within dental hard tissues. Whether induced by the invading bacteria and/or by the defending host [13,58,62], detected mineral imbalances in blood samples of leprosy sufferers may be associated with recurrent leprosy reactions since, in clinical studies, oral zinc therapy has been shown to reduce the frequency, duration, and severity of reactions [89]. Leprosy reactions may occur as type 1 (or reversal reactions) in borderline leprosy and type 2 (or erythema nodosum leprosum) in lepromatous leprosy, the latter of which may lead to “widespread and recurrent lesions which continue to appear for months or even years” [90]. Due to repeated leprosy reactions, therefore, periodicity and variation in strength of elemental depletions and enrichments may be expected in dental tissues.

Using SXRF, distributions of elements potentially affected by the disease (i.e., Cu, Fe, Zn and Ca) were mapped on the whole teeth, while high-resolution scans were also taken in areas of cementum (Figure 2 and Figure 3, File S2). The observed Ca-enriched levels in enamel and homogenous Ca distributions in dentine were also reported as a normal pattern in human teeth by Cool et al. [91], Dean et al. [84], and Martin et al. [92,93]. Moreover, the alternating Ca enrichments and depletions seen in the higher resolution Ca maps of some teeth (e.g., Næstved 211M, Odense 533M and R1386M) have also been observed by Dean et al. [94] in the acellular cementum of a *Pongo* incisor and in the cellular cementum of a *Pan* incisor. Besides the normal physiological Ca distribution and the slight fluctuations within the tissues of the scanned teeth, no significant Ca variations were observed that could be related to the leprosy infection of these medieval and early 20th-century individuals. Alternating enriched and depleted concentrations are also present in the Zn maps (Figure 2, Figure 3 and Figure 4). The dentine of Næstved 305M and Odense 896M and 914M, for example, shows some marked accentuated bands with strong and broad Zn depletions. A very clear banding pattern of Zn is also visible in the cementum of both medieval and early 20th-century leprosy sufferers (File S2).

Using synchrotron X-ray fluorescence and X-ray diffraction mapping, Stock et al. [95] investigated the Zn intensities in cementum annual growth bands of Beluga whale teeth and reported that Zn is a sensitive indicator of mineralization, with higher and lower levels corresponding, respectively, to the light and dark bands that are visible in TLM. Dean et al. [94] also reported an alignment between high Zn concentrations with light cementum bands, and vice versa, in great ape and fossil hominin teeth and suggested that cementum layers rich in Zn are either indicative of a proliferation in apposition and/or mineralization, or of a decrease in the mineralization rate, which could result in more Zn exchanging with calcium over time. Indeed, the presence of high zinc levels in areas with mineralization activity, such as peritubular dentine, suggests that zinc is involved in the mineralization process [96]. The role of Zn in mineralization was also suggested in relation to high Zn concentrations observed in outer enamel areas [85]. An accumulation of Zn is observed at the outer enamel surface as well as in the cementum and the secondary dentine of all scanned teeth (File S2). The Zn enrichment of the latter two tissues may result from their slow growth rate and prolonged direct contact with tissue fluid during formation [84,94]. Similar results were previously documented in modern and fossil primate teeth [84,92,93,94,97,98,99].

Overall, no distinctive pattern in elemental distributions can be clearly attributed to the leprosy infection. Although enamel and primary dentine develop during childhood, the slow and annual growth of cementum and secondary dentine during adulthood, when these individuals were not only infected by the mycobacterium but had also manifested the disease, could potentially capture the blood mineral imbalances experienced by the leprosy sufferers. However, no significant variation in Fe and Cu is seen in the medieval or 20th-century teeth that could be interpreted as resulting from leprosy. Although studies have reported Fe and Cu concentrations in human teeth (e.g., [97,99,100]), dental hard tissues may not imprint the Fe and Cu variations documented in blood due to the naturally low content of these two elements in teeth. The alternating pattern of Zn (and, to a lesser extent, Ca) enrichments and depletions observed in the cellular cementum is presumably natural and a physiological marker of tooth tissue incremental growth. Although, one cannot exclude that these patterns may also be related to recurrent leprosy reactions and the associated mineral imbalances documented in blood serum. For example, the homeostatic control of Ca by vitamin D [101] may indicate that blood Ca levels would need to drop significantly for a depleted Ca concentration to be imprinted in dental tissues. However, it is noteworthy that in the cellular cementum of Næstved 211C, as well as in the cellular cementum and the secondary dentine of Odense 533M, Zn and Ca have a positive correlation (File S1), also with Mg (see File S1). These trends are in agreement with clinical studies on the blood serum elemental levels of modern leprosy sufferers (e.g., [37,39,41]). Nonetheless, the correlations in dental hard tissues between Cu and Fe with the other elements (Zn, Ca, Mg) do not show the expected strength, direction, or significance compared to blood (e.g., [37,44,55]).

Considering the different homeostatic and metabolic factors that may influence the absorption and distribution of different elements from blood to body tissues, such as intestinal absorption [102,103], age [104], and pregnancy [105], it becomes evident that a direct and proportional link between blood and dental tissue elemental levels may not be applicable.

### 4.2. Exploring Evidence of Leprosy Treatment by Heavy Metals in Dental Tissues

Being influenced by the Hippocratic theory of humourism, medieval physicians believed that good health depended on a humoural balance in the body of the four principal substances (i.e., black bile, yellow bile, phlegm, blood), and, thus, the different types of treatment employed to cure an individual aimed at restoring the lost humoral equilibrium [106]. For example, bloodletting, which enabled the removal of excessive humours from the body [107], occurred four times every year at the leprosy hospital of Salle-aux-Puelles in Normandy, France, and was accompanied by a period of rest and special provisions [108]. Diet was also considered important for maintaining and restoring the humoral balance in the body. In medieval England, for example, mild and moist foods, such as poultry, eggs, and fresh fish, were considered appropriate for restoring the balance of the digestive system of leprosy sufferers [22], while in Spain, a special diet of chicken, mutton, sugar, dried fruits, and nuts proceeded the consultation by a physician [109].

The few historical sources that provide information about the treatment of leprosy in medieval Denmark date to the 16th century and mention mainly the use of different plants and animals [110,111,112]. An exception was the following recipe, mentioned in Christian Pedersen’s book En nøttelig legebog [110], specifically for wealthy individuals, which included gold as an ingredient. Nevertheless, medicinal preparations that included minerals were used in other areas (e.g., England) as a treatment for leprosy (as well as other diseases) towards the end of the medieval period when the Odense and Næstved leprosy hospitals were still in operation [22,113,114,115,116]. For instance, mercury and lead were mixed with animal products and plants and administered epidermically [114,117,118]. The use of minerals in medicinal preparations during that time is also attested to archaeologically, as mineral residues were discovered in an infirmary at the medieval priory and hospital of St Mary Spital in London [119]. Mineral preparations of mercury as well as arsenic were still used for the treatment of leprosy during the 19th and early 20th centuries; now administered also by fumigation, as well as orally and intravenously [20,21,120].

Previous work conducted by Rasmussen et al. [121] on bone samples of individuals from three medieval monastic sites in Denmark (Øm Kloster, Franciscan friaries in Odense and Svendborg) demonstrated a fourfold Hg increase in individuals with leprosy lesions (~200 ppb) compared to the control group (34 ± 15 ppb), suggesting that mercury-containing medicine was administered in 79% (11 out of 14) of the cases. Moreover, in medieval Danish rural populations, Rasmussen et al. [122] showed Hg background exposition levels at 80 and 300 ppb in cortical and trabecular bone, respectively. Regarding As concentrations, there are no published values measured in the bones or teeth of leprosy sufferers. Rasmussen [123] reports the range of the arsenic content in the enamel of individuals from medieval Denmark as being <0.001–0.239 ppm, while Hg ranges from 0.046–1.88 ppm. Moreover, Rasmussen et al. [124] interpret the 0.4–120 ppm As content of bones from medieval and post-medieval individuals from Denmark as resulting from diagenesis, while the 7–78,730 ppb Hg concentration is interpreted as reflecting medicinal treatments. Here, neither the SXRF nor LA-ICP-TOFMS setups were sensitive enough to detect Hg concentrations, while the low As concentrations visualized by LA-ICP-TOFMS in the cellular cementum of Næstved 211C as well as in the secondary dentine and in the cellular and acellular cementum of Odense 533M is not considered a reliable biological signal. If any Hg and/or As intake, therefore, was intentionally occurring for medicinal purposes by our scanned individuals, this remains well below the detection limit of the two techniques used here.

Lead concentrations, on the other hand, are well detected, with SXRF overview maps showing an accumulation of Pb in the slow-forming secondary dentine and cementum and high-resolution maps (SXRF and LA-ICP-TOFMS) depicting a well-defined pattern of alternating enriched and depleted Pb concentrations (Figure 5). Exposure to lead (Pb) was common during the medieval period [125,126,127,128], also in relation to medical treatment [115,129,130]. In medieval rural populations from Denmark, Rasmussen et al. [122] measured a Pb background level of 5 and 7 ppm in cortical and trabecular bone, respectively. Nielsen et al. [131], however, document much higher Pb concentrations in bones from two early medieval Danish sites (means of 30 and 105 ppm). In another study, Rasmussen et al. [124] interpreted the Pb content in human bone (range: 0.8–426 ppm) as being related to the increased use in Denmark, from 1400–1700 CE, of Pb-glazed ceramics for the storage of food. The skeleton is known to accumulate Pb due to the low solubility of lead phosphate; teeth and bones contain 94% of the total Pb body burden [87,131]. Because Pb^2+^ has the same chemical affinities as Ca^2+^, Zn^2+,^ and Sr^2+^, Pb is expected to substitute for Ca and P in the hydroxyapatite crystalline structure since it has a small enough ionic radius [132,133,134]. The storage and release of Pb involve the same molecular mechanisms and hormones as Ca metabolism [134].

Under constant exposure, Pb incorporation in permanent teeth increases with age until reaching a plateau [135,136]. There is substantial variation of Pb levels in modern urban human teeth (e.g., [97,137]), with the element being preferentially deposited in cementum [92,99], especially at the root apex, where the presence of cellular cementum and the larger surface area may favor Pb exchange from blood to tooth tissues [138]. Secondary dentine was also identified as the dental tissue storing most of the Pb [139,140] due to the proximity of blood in the pulp chamber [137,141]. This tissue, therefore, may provide direct insight into Pb exposure, Pb plasma levels, and Pb intake until the death of an individual or the cessation of tooth function [142,143]. This Pb enrichment may be favored by the slow growth or crystal size in secondary dentine [143].

Here, the overview SXRF maps show consistently higher Pb levels in both the secondary dentine and cementum of all scanned teeth. However, the greatest accumulation of Pb is seen in the modern Romanian teeth. According to the records of the “Francisc I. Rainer” Institute of Anthropology, individual R1386 was male and died in 1926 at 36 years of age at the Colentina Hospital (Bucharest, Romania). This individual was a blacksmith and, thus, experienced daily exposure to metals during his adult life (Pb is accumulated in cementum and secondary dentine) and probably as early as his adolescence (Pb increases from the mid-root of his M2; see Figure 5 and File S2). Pascu [144] reports that in the 1920s, industrial production was flourishing in Romania and was especially focused on exploiting Pb, among other metals. Although concentrations in Pb cannot be calculated from the SXRF data for R1386M, his Pb content is clearly higher than that of the other modern Romanian individual and well above that of all the Danish archaeological teeth.

**Figure 6:**
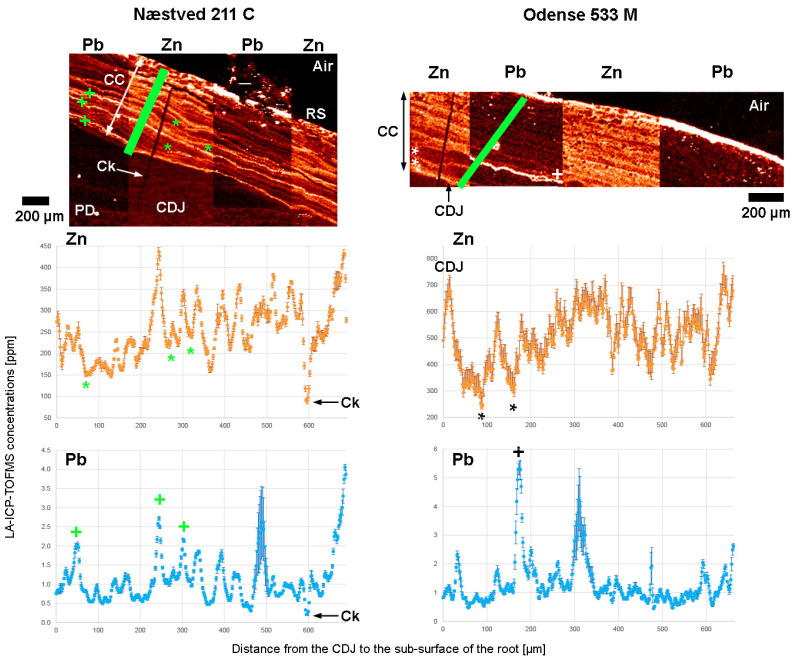
Covariation of Pb and Zn in the cellular cementum of Næstved 211C (**left**) and Odense 533M (**right**) (LA-ICP-TOFMS data used for the correlation tests, File S1). Note that two Zn depletions (black star on the graph, white star on the map) are followed by a Pb enrichment (black cross on the graph, white cross on the map) in Odense 533M, while three Pb enrichments (green cross) are followed by Zn depletions (green star) in Næstved 211C. The range of the *y*-axes has been adapted to best present the fluctuations of Zn and Pb in the two teeth. The root surface is enriched in Pb over the first ~30µm, which were avoided here to best represent the elemental covariations. Error bars represent 1 SD on either side. “CC” stands for “cellular cementum,” “CDJ” for “cementodentine junction,” “PD” for “primary dentine,” “RS” for “root surface,” “Ck” for “crack” in the cellular cementum of Næstved 211C. Graphic programs used: Adobe Photoshop CS6 and Adobe Illustrator CS6.

Quantitative Pb concentrations were measured by LA-ICP-TOFMS for two medieval teeth (Næstved 211C and Odense 533M). The results found a moderate to strong correlation between Pb and Zn (as well as Ca and Mg) variations within tooth tissues formed during adult life (File S1). In Odense 533M, a single strong Pb enrichment occurs close to the CDJ (at ~10 years of age; estimation from root formation stage in [79]). This Pb peak is preceded by two long and strong Zn depletions (Figure 6), also seen in Ca, P, Sr, Mg, and Na (File S3). These depletions in essential elements may have favored Pb incorporation and, thus, increased its toxicity. In Næstved 211C, a major Pb peak occurs at the CDJ at the time of formation of the first cellular cementum layers (~12 years of age; estimation from root formation stage in [79]) and is followed by two strong peaks registered a little later during adult life (~17 years and early 20s, rough estimation based on Zn map). These Pb enrichments seem to be systematically followed by Zn-depleted bands (Figure 6), which could result from a reduced Zn absorption due to the increased load and toxicity of Pb in the body.

Previous studies have also observed a positive correlation between Pb and Zn. This covariation has been suggested to represent a biological signal reflecting the bloodstream in the pulp cavity and the metabolism of dentine-forming cells [92,145,146]. Talpur et al. [147] showed that compared to well-nourished children, the blood and hair of malnourished children have Pb levels that are two times higher and levels of essentials elements (e.g., Ca, Fe, and Zn) that are one to twofold lower. When the diet is of insufficient quality, therefore, the correlation between Pb and Ca, Fe, or Zn becomes negative [147]. This may be explained by the increased urinary Zn excretion according to the duration and level of Pb exposure [148]. Bartón [149] also reported a strong negative correlation between hair Pb and Zn levels in preschool children. The incorporation of Zn and Pb in dental tissues may depend on formation rates and permeability [150]. Experiments found that Pb is incorporated almost instantaneously in forming dentine [151,152]. In modern humans, root formation (dentine) slows down towards completion [153]; this leaves a longer time period for incorporating Pb at the root apex, more specifically into the mineralizing matrix of the forming cementum [138].

The overall Pb background level measured in the cementum of the scanned medieval leprosy sufferers from Denmark may be related to daily Pb exposure through the consumption of contaminated water and food (lead-tiled roofs and glazed ceramics [124,154]). The few strong Pb enrichments, however, correspond to a marked exposure to the element. A possible explanation for the Pb peaks could potentially be the administration of Pb in the form of medication. Although no historical record survives that attests to the treatment of leprosy using Pb in medieval Denmark, this element was used in medicinal preparations during the later Middle Ages (cf., [22]). The administration of Pb-containing medicine would indicate that the mineral was used for medical purposes in Denmark already from the late 12th to mid-13th centuries, according to the radiocarbon dates of the two leprosy patients from Næstved (211) and Odense (533) (Table S2).

Nevertheless, during physiological events, such as pregnancy (e.g., [155,156]) or pathological events, such as severe illness [157], Pb (along with other major and trace elements) is mobilized from the bone matrix into the bloodstream due to disruptions in skeletal homeostasis. The elevated blood Pb levels are then incorporated into the mineralizing dental tissues and are recorded as Pb-enriched bands [157]. Consequently, the Pb enrichments imprinted in the dental tissues of the leprosy sufferers could indicate a period of intense physiological stress, possibly a period of exacerbated leprosy reactions. This could also be true for the cementum root surface Pb enrichment of Næstved 211C and Odense 533M, which is surprising by its intensity and duration, and which lasted until the death of both leprosy sufferers. Although it would be tempting to interpret this high Pb intoxication as being related to the occurrence of death, more research needs to be done to confirm this hypothesis. Nonetheless, the maximum Pb concentration that is found at the root surface of Odense 533M is ~100 ppm. Several public health studies report a positive correlation between Pb content in blood and teeth and further document a Pb blood concentration of 6 µg/dL for a Pb content in teeth of ~100 ppm [149,158,159]. The incorporation of Pb from the blood into dental tissues is a complex matter, yet one may speculate that following these clinical data, the degree of impairment induced by this Pb intoxication could have reached a level affecting development and health [160,161].

## 5. Conclusions

This study investigated markers of elemental blood fluctuations in the archaeological teeth of leprosy sufferers. The moderate to strong positive correlation between Zn, Ca, and Mg distributions seen in the dental tissues of Næstved 211C and Odense 533M corresponds to the results of clinical studies on blood samples of modern individuals suffering from leprosy. However, no distinctive pattern is observed in the concentration distributions of the elements that could be directly linked to the leprosy infection. Future studies on teeth and blood samples of modern leprosy sufferers with a recorded medical history could elucidate whether elemental blood imbalances related to leprosy are imprinted in dental tissues or if the elemental concentration and correlation patterns seen here indicate natural and physiological markers of tooth tissue incremental growth.

This is the first study using laser ablation to perform continuous mapping without having to extrapolate data from line scans, thus providing a true and reliable elemental mapping of the dental tissues. LA-ICP-TOFMS results reveal a constant exposure to Pb, which most likely reflects the ingestion of contaminated water and food. Although the administration of Pb in the form of medication cannot be excluded, the few strong Pb enrichments may be related to periods of intense physiological stress and to the enrichment of blood with Pb from bone stores. This is perhaps supported by the Pb enrichment seen in the last deposited cementum growth layers of Næstved 211C and Odense 533M. The positive correlation that is observed between Pb and Zn concentrations, as well as the patterns that emerge when there is relatively high incorporation of Pb in the dental tissues, may indicate underlying pathophysiological conditions that promote or are induced by an increased Pb incorporation. A similar study on a larger sample size as well as on a non-leprosy control group from the same geographical regions and time period, however, is needed in order to ascertain these interpretations.

This study is the first to investigate mineral element distributions in dental tissues of leprosy sufferers, demonstrating the potential of SXRF and LA-ICP-TOFMS for paleopathological investigations. By targeting all dental tissues and by employing a methodology that allows different degrees of resolution, we have explored elemental distributions spanning the entire life of the individuals under study.

## Figures and Tables

**Table 1:** Medieval and modern tooth samples for SXRF and LA-ICP-TOFMS multiscale imaging.

Site	Gr. Nr.	Teeth	SXRF Overview Scan		SXRF High-Resolution Scan			LA-ICP-TOFMS
			Spot Size (μm)	Exposure Time (ms)	Tissue	Spot Size (μm)	Exposure Time (ms)	
Odense	533	LLM1	10	3	AC	−	−	2 µm
					CC	1	1	1 µm
					SD	1	3	2 µm
Odense	896	LLC	10	3	−	−	−	−
		LLM1	10	2.5	TC	1.5	3	−
Odense	914	ULM1	10	3	CC	1	3	−
Odense	1149	URC	10	4	AC	1	10	−
		ULM1	10	4	CMSC?	1	3	−
Næstved	6	ULC	10	4	AC	1	10	−
Næstved	211	URC	10	3	CC	1	−	20 μm overview + 2 µm
		LRM1	10	2.5	AC	1.5	3	−
Næstved	268	LLM1	10	4	CC	1.5	3	−
Næstved	305	LLC	10	3	−	−	−	−
		LRM1	10	2.5	CC	1.5	3	−
Romania	A1651	URC	10	3	CC	1	3	−
		LRM1	10	2.5	TC	1.5	3	−
Romania	R1386	LRM2	10	2.5	CC	1.5	3	−

“Gr. Nr.” stands for “Grave Number.” “AC” stands for “acellular cementum” (on the cervical half of the root), “CC” for “cellular cementum” (apical half of the root), and “SD” stands for “secondary dentine.” “T” stands for “transition,” depicting a zone between acellular and cellular cementum, and “CMSC” for “cellular mixed stratified cementum.” Abbreviations of teeth are designed as follows: “L” or “U” for “lower” or “upper”; “L” or “R” for “left” or “right”; “C” or “M” for “canine” or “molar”; and finally, the tooth position in Arabic numerals. For instance, “LLM1” represents “lower left first molar.”

## Data Availability

The data used in this study are contained within the Supplementary Material or referenced within the text.

## Supplementary Materials

The following supporting information can be downloaded at: https://www.mdpi.com/article/10.3390/biology12020184/s1, Table S1: List of individuals and teeth included in the study from the two medieval Danish leprosy sites (Odense and Næstved); Table S2: Age and sex determination and 14C dates of the individuals of interest for SXRF and LA-ICP-TOFMS scanning (14C dates reported by Brozou et al. 2021, American Journal of Physical Anthropology); Table S3: Limits of detection (LOD) and of quantitation (LOQ) of the calibrated LA-ICP-TOFMS data; Table S4: LA-ICP-TOFMS concentrations (in ppm) along a transect (in µm; See page 5 of File S1) in the cellular cementum of the Næstved 211 C; Table S5: LA-ICP-TOFMS concentrations (in ppm) along a transect (in µm; See page 55 of File S1) in the cellular cementum of the Odense 533 M1; Table S6: LA-ICP-TOFMS concentrations (in ppm) along a transect (in µm; See page 30 of File S1) in the acellular cementum of the Odense 533 M1; Table S7: LA-ICP-TOFMS concentrations (in ppm) along a transect (in µm; See page 77 of File S1) in the secondary dentine of the Odense 533 M1). File S1. Methods and Statistical results. File S2. Synchrotron Fluorescence (SXRF) results. File S3. Laser Ablation (LA-ICP-TOFMS) results.

## References

[1] World Health Organization (2019). Global leprosy update, 2018: Moving towards a leprosy-free world–Situation de la lèpre dans le monde, 2018: Parvenir à un monde exempt de lèpre. Wkly. Epidemiol. Rec. Relev. Épidémiologique Hebd..

[2] Han X.Y., Sizer K.C., Thompson E.J., Kabanja J., Li J., Hu P., Gómez-Valero L., Silva F.J. (2009). Comparative Sequence Analysis of *Mycobacterium leprae* and the New Leprosy-Causing *Mycobacterium lepromatosis*. J. Bacteriol..

[3] Han X.Y., Seo Y.-H., Sizer K.C., Schoberle T., May G.S., Spencer J.S., Li W., Nair R.G. (2008). A New *Mycobacterium* Species Causing Diffuse Lepromatous Leprosy. Am. J. Clin. Pathol..

[4] Lynnerup N., Boldsen J.L., Grauer A.L. (2012). Leprosy (Hansen’s Disease). A Companion to Paleopathology.

[5] Ridley D., Jopling W. (1966). Classification of Leprosy According to Immunity. A Five-Group System. Int. J. Lepr. Other Mycobact. Dis..

[6] Dwivedi V.P., Banerjee A., Das I., Saha A., Dutta M., Bhardwaj B., Biswas S., Chattopadhyay D. (2019). Diet and Nutrition: An Important Risk Factor in Leprosy. Microb. Pathog..

[7] Feenstra S.G., Nahar Q., Pahan D., Oskam L., Richardus J.H. (2011). Recent Food Shortage Is Associated with Leprosy Disease in Bangladesh: A Case-Control Study. PLoS Negl. Trop. Dis..

[8] Kerr-Pontes L.R., Barreto M.L., Evangelista C.M., Rodrigues L.C., Heukelbach J., Feldmeier H. (2006). Socioeconomic, Environmental, and Behavioural Risk Factors for Leprosy in North-East Brazil: Results of a Case–Control Study. Int. J. Epidemiol..

[9] Oktaria S., Hurif N.S., Naim W., Thio H.B., Nijsten T., Richardus J. (2018). Dietary Diversity and Poverty as Risk Factors for Leprosy in Indonesia: A Case-Control Study. PLoS Negl. Trop. Dis..

[10] Wagenaar I., van Muiden L., Alam K., Bowers R., Hossain M.A., Kispotta K., Richardus J.H. (2015). Diet-Related Risk Factors for Leprosy: A Case-Control Study. PLoS Negl. Trop. Dis..

[11] Katona P., Katona-Apte J. (2008). The Interaction between Nutrition and Infection. Clin. Infect. Dis..

[12] Oz H. (2017). Nutrients, Infectious and Inflammatory Diseases. Nutrients.

[13] Sethi N., Madadi A.J., Bhandari S. (1996). Serum Zinc, Copper, Magnesium, Proteins and Superoxide Dismutase in Leprosy Patients on Multidrug Therapy–a Follow-up Study. Indian J. Lepr..

[14] Fatimah S., Rahfiludin M.Z. (2017). The Difference of BMI and Micronutrient Intake Between Multibacillary Leprosy and Non Leprosy (A Study in District Brondong, Lamongan 2013). Adv. Sci. Lett..

[15] Foster R., Sanchez A., Foulkes J., Cameron L.J. (1991). Profile of Blood Elements in Leprosy Patients. Indian J. Lepr..

[16] Khalid H.N., Mostafa M.I., Attia N.S., Bazid H.A.S.E. (2022). Serum Level of Selenium, Zinc, and Vitamin C and Their Relation to the Clinical Spectrum of Leprosy. J. Infect. Dev. Ctries.

[17] Nigam P., Dayal S., Sriwastava P., Joshi L. (1979). Serum Calcium and Magnesium in Leprosy. Asian J. Infect. Dis..

[18] Venkatesan K., Kannan K.B., Bharadwaj V.P., Sritharan V., Katoch K., Usha R., Ramu G. (1983). Serum Copper & Zinc in Leprosy & Effect of Oral Zinc Therapy. Indian J. Med. Res..

[19] World Health Organization (2020). Leprosy/Hansen Disease: Management of Reactions and Prevention of Disabilities. Technical Guidance.

[20] MacLeod J.M.H. (1927). Sections of Tropical Diseases and Parasitology, Dermatology, and Therapeutics and Pharmacology. Proc. R. Soc. Med..

[21] Muir E. (1933). Treatment of Leprosy. A Review. Int. J. Lepr..

[22] Rawcliffe C. (2006). Leprosy in Medieval England.

[23] Beaumont J., Montgomery J. (2016). The Great Irish Famine: Identifying Starvation in the Tissues of Victims Using Stable Isotope Analysis of Bone and Incremental Dentine Collagen. PLoS ONE.

[24] Cerrito P., Bailey S.E., Hu B., Bromage T.G. (2020). Parturitions, Menopause and Other Physiological Stressors Are Recorded in Dental Cementum Microstructure. Sci. Rep..

[25] Colard T., Bertrand B., Naji S., Delannoy Y., Bécart A. (2018). Toward the Adoption of Cementochronology in Forensic Context. Int. J. Legal Med..

[26] Dean M.C. (2010). Retrieving Chronological Age from Dental Remains of Early Fossil Hominins to Reconstruct Human Growth in the Past. Phil. Trans. R. Soc. B.

[27] Schwartz G.T., Reid D.J., Dean M.C., Zihlman A.L. (2006). A Faithful Record of Stressful Life Events Recorded in the Dental Developmental Record of a Juvenile Gorilla. Int. J. Primatol..

[28] Skinner M., Byra C. (2019). Signatures of Stress: Pilot Study of Accentuated Laminations in Porcine Enamel. Am. J. Phys. Anthropol..

[29] Fehrenbach M.J., Popowics T. (2020). Dental Embryology, Histology and Anatomy.

[30] Nanci A. (2018). Ten Cate’s Oral Histology. Development, Structure and Function.

[31] Gherase M.R., Fleming D.E.B. (2019). Probing Trace Elements in Human Tissues with Synchrotron Radiation. Crystals.

[32] National Research Council (1989). Diet and Health: Implications for Reducing Chronic Disease Risk.

[33] Prashanth L., Kattapagari K., Chitturi R.T., Baddam V.R.R., Prasad L.K. (2015). A Review on Role of Essential Trace Elements in Health and Disease. J. NTR Univ. Health Sci..

[34] Fairweather-Tait S., Hurrell R.F. (1996). Bioavailability of Minerals and Trace Elements: Members of EC Flair Concerted Action No. 10: Measurements of Micronutrient Absorption and Status. Nutr. Res. Rev..

[35] Soetan K., Olaiya C.O., Oyewole O. (2010). The Importance of Mineral Elements for Humans, Domestic Animals and Plants: A Review. Afr. J. Food Sci..

[36] Wada O. (2004). What Are Trace Elements? Their Deficiency and Excess States. JMAJ.

[37] Rao K., Gupta J.D., Sehgal V., Chakrabarti A., Saha K. (1985). Trace Elements in the Sera of Leprosy Spectrum. Indian J. Lepr..

[38] Arora P., Dhillon K., Rajan S., Sayal S., Das A. (2002). Serum Zinc Levels in Cutaneous Disorders. MJAFI.

[39] George J., Bhatia V.N., Balakrishnan S., Ramu G. (1991). Serum Zinc/Copper Ratio in Subtypes of Leprosy and Effect of Oral Zinc Therapy on Reactional States. Int. J. Lepr. Other Mycobact. Dis..

[40] Mathur N.K., Sharma M.L., Mangal H.N., Rai S.M. (1984). Serum Zinc Levels in Subtypes of Leprosy. Int. J. Lepr. Other Mycobact. Dis..

[41] Pradhan T., Kumari S. (2015). Evaluation of Oxidative Status and Zinc Level in Leprosy Patients after Zinc Supplementation. Int. Biol. Med. Res..

[42] Saxena N., Sharma R., Singh V.S. (1988). Study of Serum Zinc Level in Leprosy. Indian J. Lepr..

[43] Saxena N., Sharma R., Singh V.S. (1990). Serum Iron and Total Iron Binding Capacity in Leprosy Patients. Indian J. Lepr..

[44] Bhattacharya R.N., Goswami K., Bandyopadhyay A. (2020). Copper and Ascorbic Acid Status in Children Suffering from Leprosy. EJBPS.

[45] Jain P., Khare V., Koshti A., Malik R., Bhimte B. (2014). Serum Zinc Level Estimation- Comparison between Normal Control and in Leprosy Patients. Int. J. Biol. Med. Res..

[46] Oon B.B., Khong K.Y., Greaves M.W., Plummer V.M. (1974). Trophic Skin Ulceration of Leprosy: Skin and Serum Zinc Concentrations. BMJ.

[47] Mennen U., Howells C., Wiese A. (1993). Serum Zinc, Sodium, Calcium, Magnesium and Potassium Levels and Standard Diet in Leprosy Patients. Indian J. Lepr..

[48] Rao K.N., Saha K. (1986). Undernutrition in Lepromatous Leprosy, Part II. Altered Levels of Serum Elements. Their Association with the Disease and Not with Food Deprivation. Lepr. Rev..

[49] Sher R., Shulman G., Baily P., Politzer W.M. (1981). Serum Trace Elements and Vitamin A in Leprosy Subtypes. Am. J. Clin. Nutr..

[50] Wannemacher R.W., Pekarek R.S., Klainer A.S., Bartelloni P.J., Dupont H.L., Hornick R.B., Beisel W.R. (1975). Detection of a Leukocytic Endogenous Mediator-like Mediator of Serum Amino Acid and Zinc Depression during Various Infectious Illnesses. Infect. Immun..

[51] Kumar N., Malhotra V., Singh R., Vij J., Anand B. (1982). Structure and Function of the Small Bowel in Lepromatous Leprosy. Int. J. Lepr. Other Mycobact. Dis..

[52] Bhattacharya R.N., Goswami K., Bandyopadhyay A. (2020). Plasma Copper and Ascorbic Acid Status in Leprosy. EJBPS.

[53] Bharadwaj V.P., Venkatesan K., Ramu G., Desikan K.V. (1978). Serum Iron and Total Iron Binding Capacity in Leprosy Patients. Leprosy in India.

[54] Shwe T., Than-Toe, Tu A.T.B. (1976). Serum Iron and Total Iron Binding Capacity in Burmese Patients with Leprosy. Lepr. Rev..

[55] Tamara R., Muchtar S.V., Amin S., Seweng A., Sjahril R., Adam A.M. (2018). Serum Iron, Total Iron Binding Capacity and Transferrin Saturation Levels in Leprosy Patients before Multi Drug Therapy - World Health Organization (MDT-WHO) Compared with Healthy Control Group. Int. J. Med. Rev. Case Rep..

[56] Jain A., Mukherjee A., Chattopadhya D., Saha K. (1995). Biometals in Skin and Sera of Leprosy Patients and Their Correlation to Trace Element Contents of M. Leprae and Histological Types of the Disease, a Comparative Study with Cutaneous Tuberculosis. Int. J. Lepr. Other Mycobact. Dis..

[57] Swathi M., Tagore R. (2015). Study of Oxidative Stress in Different Forms of Leprosy. Indian J. Dermatol..

[58] Skaar E.P., Raffatellu M. (2015). Metals in Infectious Diseases and Nutritional Immunity. Metallomics.

[59] Weinberg E.D. (1975). Nutritional Immunity: Host’s Attempt to Withhold Iron from Microbial Invaders. JAMA.

[60] Botella H., Stadthagen G., Lugo-Villarino G., de Chastellier C., Neyrolles O. (2012). Metallobiology of Host–Pathogen Interactions: An Intoxicating New Insight. Trends in Microbiology.

[61] Hood M.I., Skaar E.P. (2012). Nutritional Immunity: Transition Metals at the Pathogen–Host Interface. Nat. Rev. Microbiol..

[62] Neyrolles O., Mintz E., Catty P. (2013). Zinc and Copper Toxicity in Host Defense against Pathogens: Mycobacterium Tuberculosis as a Model Example of an Emerging Paradigm. Front. Cell. Infect. Microbiol..

[63] Møller-Christensen V. (1954). Hvad de Døde Fortalte. Historisk Samfund for Præstø Amt.

[64] Madsen K. (1990). Spedalskhed Og Sct. Jørgensgård.

[65] Arentoft E. (1999). De spedalskes hospital: Udgravning af Sankt Jørgensgården i Odense.

[66] Nielsen E. (1981). Beretning for Udgravningen 1980-81 Af Sct. Jørgensgården, Odense.

[67] Michelsen F. (1954). St. Jørgensgården i Aaderup Ved Næstved. Årbog Hist. Samf. Præstø.

[68] Ehlers E. (1898). Danske St. Jørgensgaarde i Middelalderen..

[69] Erslev K. (1901). Testamenter fra Danmarks Middelalder indtil 1450.

[70] Møller-Christensen V. (1953). Location and Excavation of the First Danish Leper Graveyard from the Middle Ages – St. Jørgen’s Farm, Næstved. Bull. Hist. Med..

[71] Nielsen E. (1983). Sct. Jørgensgården i Odense. Fynske Minder 1982.

[72] Richards P. (1977). The Medieval Leper and His Northern Heirs.

[73] Boldsen J.L., Mollerup L. (2006). Outside St. Jørgen: Leprosy in the Medieval Danish City of Odense. Am. J. Phys. Anthropol..

[74] Møller-Christensen V. (1961). Bone Changes in Leprosy.

[75] Møller-Christensen V. (1953). Ten Lepers from Næstved in Denmark. A Study of Skeletons from a Medieval Danish Leper Hospital.

[76] Schuurs A. (2013). Pathology of the Hard Dental Tissues.

[77] Boesenberg U., Ryan C.G., Kirkham R., Siddons D.P., Alfeld M., Garrevoet J., Núñez T., Claussen T., Kracht T., Falkenberg G. (2016). Fast X-Ray Microfluorescence Imaging with Submicrometer-Resolution Integrating a Maia Detector at Beamline P06 at PETRA III. J. Synchrotron Rad..

[78] Schroer C., Boye P., Feldkamp J., Patommel J., Samberg D., Schropp A., Schwab A., Stephan S., Falkenberg G., Wellenreuther G. (2010). Hard X-Ray Nanoprobe at Beamline P06 at PETRA III. Nucl. Instrum. Meth. A.

[79] AlQahtani S.J., Hector M.P., Liversidge H.M. (2010). Brief Communication: The London Atlas of Human Tooth Development and Eruption. Am. J. Phys. Anthropol..

[80] Burger M., Gundlach-Graham A., Allner S., Schwarz G., Wang H.A.O., Gyr L., Burgener S., Hattendorf B., Grolimund D., Günther D. (2015). High-Speed, High-Resolution, Multielemental LA-ICP-TOFMS Imaging: Part II. Critical Evaluation of Quantitative Three-Dimensional Imaging of Major, Minor, and Trace Elements in Geological Samples. Anal. Chem..

[81] Gundlach-Graham A., Günther D. (2016). Toward Faster and Higher Resolution LA–ICPMS Imaging: On the Co-Evolution of LA Cell Design and ICPMS Instrumentation. Anal. Bioanal. Chem..

[82] Armbruster D.A., Pry T. (2008). Limit of Blank, Limit of Detection and Limit of Quantitation. Clin. Biochem. Rev..

[83] RStudio Team (2020). RStudio: Integrated Development Environment for R.

[84] Dean M.C., Spiers K.M., Garrevoet J., Le Cabec A. (2019). Synchrotron X-Ray Fluorescence Mapping of Ca, Sr and Zn at the Neonatal Line in Human Deciduous Teeth Reflects Changing Perinatal Physiology. Arch. Oral Biol..

[85] Humphrey L.T., Jeffries T.E., Dean M.C., Irish J.D., Nelson G.C. (2008). Micro Spatial Distributions of Lead and Zinc in Human Deciduous Tooth Enamel. Technique and application in Dental Anthropology.

[86] Müller W., Nava A., Evans D., Rossi P.F., Alt K.W., Bondioli L. (2019). Enamel Mineralization and Compositional Time-Resolution in Human Teeth Evaluated via Histologically-Defined LA-ICPMS Profiles. Geochim. Cosmochim. Acta.

[87] Barbosa F., Tanus-Santos J.E., Gerlach R.F., Parsons P.J. (2005). A Critical Review of Biomarkers Used for Monitoring Human Exposure to Lead: Advantages, Limitations, and Future Needs. Environ. Health Perspect..

[88] Bercovitz K., Laufer D. (1991). Age and Gender Influence on Lead Accumulation in Root Dentine of Human Permanent Teeth. Arch. Oral Biol..

[89] Mahajan P., Jadhav V., Patki A.H., Jogaikar D.G., Mehta J. (1994). Oral Zinc Therapy in Recurrent Erythema Nodosum Leprosum: A Clinical Study. Indian J. Lepr..

[90] Naafs B., Noto S., Nunzi E., Massone C. (2012). Reactions in Leprosy. Leprosy. A Practical Guide..

[91] Cool S.M., Forwood M.R., Campbell P., Bennett M.B. (2002). Comparisons between Bone and Cementum Compositions and the Possible Basis for Their Layered Appearances. Bone.

[92] Martin R.R., Naftel S.J., Nelson A.J., Feilen A.B., Narvaez A. (2007). Metal Distributions in the Cementum Rings of Human Teeth: Possible Depositional Chronologies and Diagenesis. J. Archaeol. Sci..

[93] Martin R.R., Naftel S.J., Nelson A.J., Feilen A.B., Narvaez A. (2004). Synchrotron X-Ray Fluorescence and Trace Metals in the Cementum Rings of Human Teeth. J. Environ. Monit..

[94] Dean C., Le Cabec A., Spiers K., Zhang Y., Garrevoet J. (2018). Incremental Distribution of Strontium and Zinc in Great Ape and Fossil Hominin Cementum Using Synchrotron X-Ray Fluorescence Mapping. J. R. Soc. Interface..

[95] Stock S.R., Finney L.A., Telser A., Maxey E., Vogt S., Okasinski J.S. (2017). Cementum Structure in Beluga Whale Teeth. Acta Biomater..

[96] Stock S.R., Deymier-Black A.C., Veis A., Telser A., Lux E., Cai Z. (2014). Bovine and Equine Peritubular and Intertubular Dentin. Acta Biomater..

[97] Cohen D.D., Clayton E., Ainsworth T. (1981). Preliminary Investigations of Trace Element Concentrations in Human Teeth. Nucl. Instrum. Methods Phys. Res., B.

[98] Dean C., Le Cabec A., Van Malderen S.J.M., Garrevoet J. (2020). Synchrotron X-Ray Fluorescence Imaging of Strontium Incorporated into the Enamel and Dentine of Wild-Shot Orangutan Canine Teeth. Arch. Oral Biol..

[99] Rautray T.R., Das S., Rautray A.C. (2010). *In Situ* Analysis of Human Teeth by External PIXE. Nucl. Instrum. Methods Phys. Res., B.

[100] Carvalho M.L., Casaca C., Marques J.P., Pinheiro T., Cunha A.S. (2001). Human Teeth Elemental Profiles Measured by Synchrotron X-Ray Fluorescence: Dietary Habits and Environmental Influence. X-Ray Spectrom..

[101] Bouillon R., Suda T. (2014). Vitamin D: Calcium and Bone Homeostasis during Evolution. BoneKEy Reports.

[102] King J.C., Shames D.M., Woodhouse L.R. (2000). Zinc Homeostasis in Humans. J. Nutr..

[103] Walczyk T., von Blanckenburg F. (2002). Natural Iron Isotope Variations in Human Blood. Science.

[104] Veldurthy V., Wei R., Oz L., Dhawan P., Jeon Y.H., Christakos S. (2016). Vitamin D, Calcium Homeostasis and Aging. Bone Research.

[105] Kovacs C.S. (2015). Calcium and Bone Metabolism during Pregnancy and Lactation. J. Mammary Gland Biol. Neoplasia.

[106] Rawcliffe C. (1999). Medicine and Society in Later Medieval England.

[107] Demaitre L. (2007). Leprosy in Premodern Medicine. A Malady of the Whole Body.

[108] Brenner E. (2015). Leprosy and Charity in Medieval Rouen.

[109] Jáuregui C., Connelly E., Künzel S. (2018). Inside the Leprosarium: Illness in the Daily Life of 14th Century Rouen. New Approaches to Disease, Disability, and Medicine in Medieval Europe.

[110] Pedersen C. (1533). En Nøttelig Legebog.

[111] Smit H. (1557). En Skøn Nyttelig Lægebog Ind Hollendis Atskillige Mange Skøne Forfarne Lægedommer Huilcke Som Tiæne Bartskerrerne, Oc Dem Som Ville Læge Ferske Oc Gamle Saar. Desligiste Oc Om Bad Aare Ladelse Oc Koppe Settelse, Och Om de Lagedomme Som Findis i Apotecken Fale..

[112] Norse Medical and Herbal Healing (2011). A Medical Book from Medieval Iceland.

[113] Arrizabalaga J., Henderson J., French R. (1997). The Great Pox. The French Disease in Renaissance Europe.

[114] Goldwater L.J. (1972). Mercury. A History of Quicksilver.

[115] Lev E. (2007). Drugs Held and Sold by Pharmacists of the Jewish Community of Medieval (11–14th Centuries) Cairo According to Lists of Materia Medica Found at the Taylor–Schechter Genizah Collection, Cambridge. J. Ethnopharmacol..

[116] Nriagu J.O., Needleman H.L. (1992). Saturnine Drugs and Medicinal Exposure to Lead: An Historical Outline. Human Lead Exposure.

[117] Dawson W.R. (1934). A Leechbook or Collection of Medical Recipes of the Fifteenth Century.

[118] Ogden M.S. (1969). The Liber de Diversis Medicinis.

[119] Harward C., Holder N., Phillpotts C., Thomas C. (2019). The Medieval Priory and Hospital of St Mary Spital and the Bishopsgate Suburb. Excavations at Spitalfields Market, London E1, 1991–2007.

[120] Buckingham J. (2002). Leprosy in Colonial South India: Medicine and Confinement..

[121] Rasmussen K.L., Boldsen J.L., Kristensen H.K., Skytte L., Hansen K.L., Mølholm L., Grootes P.M., Nadeau M.-J., Eriksen K.M.F. (2008). Mercury Levels in Danish Medieval Human Bones. J. Archaeol. Sci..

[122] Rasmussen K.L., Skytte L., Jensen A.J., Boldsen J.L. (2015). Comparison of Mercury and Lead Levels in the Bones of Rural and Urban Populations in Southern Denmark and Northern Germany during the Middle Ages. J. Archaeol. Sci. Rep..

[123] Rasmussen E.G. (1974). Antimony, Arsenic, Bromine and Mercury in Enamel from Human Teeth. Eur. J. Oral Sci..

[124] Rasmussen L.K., Skytte L., D’imporzano P., Thomsen O.P., Søvsø M., Lier Boldsen J. (2016). On the Distribution of Trace Element Concentrations in Multiple Bone Elements in 10 Danish Medieval and Post-Medieval Individuals. Am. J. Phys. Anthropol..

[125] Budd P., Millard A., Chenery C., Lucy S., Roberts C. (2004). Investigating Population Movement by Stable Isotope Analysis: A Report from Britain. Antiquity.

[126] Le Roux G., Weiss D., Grattan J., Givelet N., Krachler M., Cheburkin A., Rausch N., Kober B., Shotyk W. (2004). Identifying the Sources and Timing of Ancient and Medieval Atmospheric Lead Pollution in England Using a Peat Profile from Lindow Bog, Manchester. J. Environ. Monit..

[127] Lessler M.A. (1988). Lead and Lead Poisoning from Antiquity to Modern Times. Ohio J. Sci..

[128] Rasmussen K.L., Delbey T., d’Imporzano P., Skytte L., Schiavone S., Torino M., Tarp P., Thomsen P.O. (2020). Comparison of Trace Element Chemistry in Human Bones Interred in Two Private Chapels Attached to Franciscan Friaries in Italy and Denmark: An Investigation of Social Stratification in Two Medieval and Post-Medieval Societies. Herit. Sci..

[129] Alexandrovskaya E.I., Panova T. (2003). History of the Soil, Cultural Layer, and People in Medieval Moscow. Rev. Mex. Cienc..

[130] Francia S., Stobart A. (2014). Critical Approaches to the History of Western Herbal Medicine: From Classical Antiquity to the Early Modern Period.

[131] Nielsen O.V., Grandjean P., Bennike P. (1982). Chemical Analyses of Archaeological Bone-Samples: Evidence for High Lead Exposure on the Faroe Islands. J. Dan. Archaeol..

[132] Bigi A., Gandolfi M., Gazzano M., Ripamonti A., Roveri N., Thomas S.A. (1991). Structural Modifications of Hydroxyapatite Induced by Lead Substitution for Calcium. J. Chem. Soc. Dalton Trans..

[133] Carvalho M.L., Casaca C., Pinheiro T., Marques J.P., Chevallier P., Cunha A.S. (2000). Analysis of Human Teeth and Bones from the Chalcolithic Period by X-Ray Spectrometry. Nucl. Instrum. Methods Phys. Res. B.

[134] Pounds G.J., Long G.J., Rosen J.F. (1991). Cellular and Molecular Toxicity of Lead in Bone. Environ. Health Perspect..

[135] Rabinowitz M.B., Leviton A., Bellinger D. (1993). Relationships between Serial Blood Lead Levels and Exfoliated Tooth Dentin Lead Levels: Models of Tooth Lead Kinetics. Calcif. Tissue Int..

[136] Steenhout A. (1982). Kinetics of Lead Storage in Teeth and Bones: An Epidemiologic Approach. Arch. Environ. Health.

[137] Gulson B.L., Gillings B.R. (1997). Lead Exchange in Teeth and Bone – a Pilot Study Using Stable Lead Isotopes. Environ. Health Perspect..

[138] Arora M.Y., Chan S.W., Kennedy B.J., Sharma A., Crisante D., Murray Walker D. (2004). Spatial Distribution of Lead in the Roots of Human Primary Teeth. J. Trace Elem. Med. Biol..

[139] Cox A., Keenan F., Cooke M., Appleton J. (1996). Trace Element Profiling of Dental Tissues Using Laser Ablation-Inductively Coupled Plasma-Mass Spectrometry. Fresen. J. Anal. Chem..

[140] Gulson B.L. (1996). Tooth Analyses of Sources and Intensity of Lead Exposure in Children. Environ. Health Perspect..

[141] Steenhout A., Pourtois M. (1981). Lead Accumulation in Teeth as a Function of Age with Different Exposures. Br. J. Ind. Med..

[142] Shapiro I.M., Mitchell G., Davidson I., Katz S.H. (1975). The Lead Content of Teeth. Arch. Environ. Health.

[143] Shepherd T.J., Dirks W., Manmee C., Hodgson S., Banks D.A., Averley P., Pless-Mulloli T. (2012). Reconstructing the Life-Time Lead Exposure in Children Using Dentine in Deciduous Teeth. Sci. Total Environ..

[144] Pascu G. (2015). Le Patrimoine Industriel-Minier Facteur de Développement Territorial: Complexité et Enjeux en Roumanie, en Comparaison Avec la France et la Grande-Bretagne. Master’s Thesis.

[145] Ide-Ektessabi A., Shirasawa K., Koizumi A., Azechi M. (2004). Application of Synchrotron Radiation Microbeams to Environmental Monitoring. Nucl. Instrum. Methods Phys. Res. B.

[146] Wang Y., Specht A., Liu Y., Finney L., Maxey E., Vogt S., Zheng W., Weisskopf M., Nie L.H. (2016). Microdistribution of Lead in Human Teeth Using Microbeam Synchrotron Radiation X-Ray Fluorescence (μ-SRXRF): Microdistribution of Lead in Human Teeth Using μ-SRXRF. X-Ray Spectrom..

[147] Talpur S., Afridi H.I., Kazi T.G., Talpur F.N. (2018). Interaction of Lead with Calcium, Iron, and Zinc in the Biological Samples of Malnourished Children. Biol. Trace Elem. Res..

[148] Miller G.D., Massaro T., Massaro E.J. (1990). Interactions between Lead and Essential Elements: A Review. Neurotoxicology.

[149] Bartoń H. (2011). Advantages of the Use of Deciduous Teeth, Hair, and Blood Analysis for Lead and Cadmium Bio-Monitoring in Children. A Study of 6-Year-Old Children from Krakow (Poland). Biol. Trace Elem. Res..

[150] Brudevold F., Steadman L.T., Smith F.A. (1960). Inorganic and Organic Components of Tooth Structure. Ann. N. Y. Acad. Sci..

[151] Okada M. (1943). Hard Tissues of Animal Body: Highly Interesting Details of Nippon Studies in Periodic Patterns of Hard Tissues Are Described. Shanghai Evening Post.

[152] Papakyrikos A.M., Arora M., Austin C., Boughner J.C., Capellini T.D., Dingwall H.L., Greba Q., Howland J.G., Kato A., Wang X.-P. (2020). Biological Clocks and Incremental Growth Line Formation in Dentine. J. Anat..

[153] Macchiarelli R., Bondioli L., Debénath A., Mazurier A., Tournepiche J.-F., Birch W., Dean M.C. (2006). How Neanderthal Molar Teeth Grew. Nature.

[154] Grandjean P., Griffin T.B., Knelson J.H. (1975). Lead in Danes. Lead.

[155] Franklin C.A., Inskip M.J., Baccanale C.L., Edwards C.M., Manton W.I., Edwards E., O’Flaherty E.J. (1997). Use of Sequentially Administered Stable Lead Isotopes to Investigate Changes in Blood Lead during Pregnancy in a Nonhuman Primate (*Macaca Fascicularis*). Fundam. Appl. Toxicol..

[156] Gulson B., Mizon K., Korsch M., Taylor A. (2016). Revisiting Mobilisation of Skeletal Lead during Pregnancy Based on Monthly Sampling and Cord/Maternal Blood Lead Relationships Confirm Placental Transfer of Lead. Arch. Toxicol..

[157] Austin C., Smith T.M., Farahani R.M.Z., Hinde K., Carter E.A., Lee J., Lay P.A., Kennedy B.J., Sarrafpour B., Wright R.J. (2016). Uncovering System-Specific Stress Signatures in Primate Teeth with Multimodal Imaging. Sci. Rep..

[158] Cleymaet R., Collys K., Retief D.H., Michotte Y., Slop D., Taghon E., Maex W., Coomans D. (1991). Relation between Lead in Surface Tooth Enamel, Blood, and Saliva from Children Residing in the Vicinity of a Non-Ferrous Metal Plant in Belgium. Br. J. Ind. Med..

[159] Habercam J.W., Keil J.E., Routt Reigart J., Croft H.W. (1974). Lead Content of Human Blood, Hair, and Deciduous Teeth: Correlation with Environmental Factors and Growth. J. Dent. Res..

[160] Fewtrell L., Kaufmann R., Prüss-Üstün A. (2003). Lead: Assessing the Environmental Burden of Disease at National and Local Levels.

[161] (2010). World Health Organization Childhood Lead Poisoning.

