# Supplementary material for: Using SXRF and LA-ICP-TOFMS to Explore Evidence of Treatment and Physiological Responses to Leprosy in Medieval Denmark
Source: Biology (Basel). 2023 Jan 25;12(2):184. doi: 10.3390/biology12020184 (PMC9952905; doi:10.3390/biology12020184)

File S1

Methods and Statistical results

Using SXRF and LA-ICP-TOFMS to explore evidence of treatment and physiological responses to leprosy in medieval Denmark

Biology

Anastasia Brozou, Marcello A. Mannino, Stijn J.M. Van Malderen, Jan Garrevoet, Eric Pubert, Benjamin T. Fuller, M. Christopher Dean, Thomas Colard, Frédéric Santos, Niels Lynnerup, Jesper L. Boldsen, Marie Louise Jørkov, Andrei Dorian Soficaru, Laszlo Vincze, Adeline Le Cabec

Corresponding author:
Adeline Le Cabec
E-mail: adeline.le-cabec@u-bordeaux.fr

Content

1. Methods

Tooth thin section preparation 2

SXRF data acquisition and processing 2

LA-ICP-TOFMS imaging of Odense 533M and Næstved 211C 3

Statistical analysis 3

References 4

2. Results

Næstved 211C – Cellular Cementum 5

Odense 533M1 – Acellular Cementum 18

Odense 533M1 – Cellular Cementum 31

Odense 533M1 – Secondary Dentine 42

1. Methods

Tooth thin section preparation

To optimise the chances of visualising an area of cementum not affected by taphonomic damages (e.g., cracks, remineralisation, fungi), and avoid restricting the field of view to acellular cementum where increments are tightly packed, the tooth root was sectioned longitudinally through the root apices. Single-rooted teeth (i.e., canines) were sectioned labio-lingually. For lower molars, a mesio-distal section enables to involve both root apices, while for three-rooted maxillary molars, the plane of section passed through the apices of the lingual root and the mesio-buccal root. Note that for molars, the plane of section was oriented to pass through the cusp tips as much as possible, in order to access the earliest information recorded during tooth growth.

Prior to sectioning, the teeth were embedded in epoxy resin for a minimum of 12 hours. A Buehler Linear Precision Saw Isomet 5000 was used in the cementochronology laboratory at *PACEA* (University of Bordeaux, France) to slice the tooth a bit off the desired plane of section. Subsequently, the tooth surface of the thicker half was first briefly polished using an abrasive disk P240 (NAC, Presi) with 58 µm particles size and resin disks (Imax-R, Presi) with 54 µm or 18 µm diamond particles embedded, and then glued on a glass slide (30 x 45 mm or 45 x 60 mm based on the size of each tooth) using epoxy resin at 65°C, under pressure for one hour. A further sectioning followed to produce tooth slices of ~300-400 µm, which were ground and lapped down to between 80-100 µm, first as described above and then using a fine diamond abrasive suspension at 9 µm, 3 µm and 1 µm (LDP polycrystalline diamond suspension, Presi) deposited on polishing clothes (PAD MAG, Presi).

SXRF data acquisition and processing

Experiments were performed on the P06 Beamline (Boesenberg et al., 2016; Schroer et al., 2010), Petra III, at DESY (Deutsches Elektronen-Synchrotron, Hamburg, Germany). The storage ring was operated in 480-bunch mode using top-up filling mode with a current of 120 mA ± 0.5 mA, and an undulator gap of 11.07 mm. The primary X-ray beam was monochromatised to 16.6 keV using a double crystal Si111 monochromator and focused using a Kirkpatrick-Baez (KB) mirror system (JTEC, Japan) to 500×500 nm^2. The experimental configuration consisted of two Vortex EM silicon drift detectors, Hitachi High-Tech Science America, Inc., the second of which was collimated. Both detectors were positioned symmetrically at scattering angles of 135 degrees at a distance of 9 mm from the focal point. The use of dual-detector "backscatter" geometry enables maximising the solid angle during analysis of thin polished samples (∼100 µm-thick in this study), and imaging large area SXRF with micrometric resolution using millisecond dwell times (Falkenberg et al., 2017). This setup allowed capturing the Kα emission lines from Si to Sr and the Lα emission lines of Hg and Pb. The Mg signal, however, is drastically lowered by absorption through the sample itself and the air path. Furthermore, the Mg Kα emission line is at 1.254 keV, which is not well-suited for the primary energy (16.6 keV). To note that some interferences within the spectrum may affect the sensitivity for detecting elements, such as Pb or Hg.

Spectral peak deconvolution and integration was performed using the core of PyMca 5.5.0 (Solé et al., 2007), whilst calibration was performed by an in-house script. Image analysis was performed in HDIP v-1.3.3.1073 (Teledyne CETAC Technologies, Bozeman, MT, USA). The X-ray yield calculations were performed assuming a hydroxyapatite matrix ($Ca_{10}(PO_4)_6(OH)_2$) with density of 2.85 g.cm^{-3} for the enamel phase and of 1.6 g.cm^{-3} for the dentine phase (Djomehri et al., 2015). Elemental mass fractions were determined by calculating an areal density sensitivity from measurements of standard Ti, Fe, and Cu foils with areal density of 59.0, 55.0, and 47.9 µg.cm^{-2}, respectively (Micromatter Technologies Inc. Canada), and measured thickness of the samples. Tooth section thickness was measured throughout the whole surface of the specimen in five positions for canines and eight positions for molars. The average tooth section thickness was also taken into account in the x-ray mass attenuation coefficients of the hydroxyapatite phase during attenuation correction

(Szczerbowska-Boruchowska, 2012). Glass slides substrates were included in the overall sample model as appropriate (i.e., background subtraction). Normalisation to the incoming X-ray flux was applied. In the calibrated data, SXRF concentrations are reported by mass fraction ($\mu g.g^{-1}$, i.e., ppm), and/or areal density ($g.cm^{-3}$). Pb images contain no quantitative data (i.e., relative Pb content is expressed in arbitrary units 'a.u.'), as L-lines were not modelled.

LA-ICP-TOFMS imaging of Odense 533M and Næstved 211C

LA-ICP-TOFMS imaging was performed at the Department of Chemistry, Ghent University (Belgium) with a setup consisting of an Iridia 193 nm ArF* excimer-based laser ablation system (Teledyne Photon Machines, Bozeman, MT, U.S.A.) coupled to an icpTOF 2R (TOFWERK AG, Thun, Switzerland) TOF-based ICP-MS instrument. The laser ablation system was equipped with the Cobalt Long-Pulse ablation cell and the aerosol rapid introduction system (ARIS), which introduced an Ar make-up gas flow (∼1.05 $L.min^{-1}$) into an optimised He carrier gas flow of 0.60 $L.min^{-1}$ before entering the plasma. The LA-ICP-MS system was optimised via ablation of NIST SRM612 (National Institute for Standards and Technology, Gaithersburg, MD, U.S.A.), with tuning for the highest intensities for $^{24}Mg^{+}$, $^{115}In^{+}$ and $^{238}U^{+}$, whilst maintaining low oxide formation (<1% $^{238}U^{16}O^{+}/^{238}U^{+}$) and a ratio of $^{238}U^{+}/^{232}Th^{+}$ ratio ∼1. Imaging was performed in fixed dosage mode 1 and with an energy density of 3 $J.cm^{-2}$, at a repetition rate of 250 Hz, using a circular spot size of 4 or 2 µm, with a vertical interspacing between the lines between 20 and 2 µm. The images were recorded using bracketing with NIST SRM612. The icpTOF 2R ICP-TOFMS was operated in standard operation mode with a mass coverage range of 14-254 m/q. The read-out frames of 3.8 ms were synchronised with the laser ablation repetition rate, with a transfer delay of 53.7 ms. The iCAP Q was equipped with the LA injector of 2.5 mm inner diameter and nickel sample and skimmer cones with a skimmer cone insert of 2.8 mm in diameter. The RF power was set to 1500 W, and an auxiliary Ar gas flow rate of ∼0.90 $L.min^{-1}$ and a plasma Ar gas flow rate of 15 $L.min^{-1}$ were used.

Statistical analysis

The classical correlation tests involving Pearson's r and Spearman ρ (rho), (with α=0.05) were computed in RStudio using the function cor.test(), and adjusting the method to "pearson" or "spearman". However, the Pearson correlation is sensitive to outliers, which may weaken the strength and the significance of the results. A more robust correlation test involving the Minimum Covariance Determinant (MCD) was calculated using the corr.plot() function. The MCD is a highly robust estimator of multivariate location and scatter (Hubert et al., 2018; Rousseeuw and Driessen, 1999; Santos, 2020). This procedure runs the correlation test on a user-defined percentage of the data (here quan= 0.8, meaning 80% of the data are kept) representing the "good part" of the data, thus removing values detected as outliers. Some elements such as Fe, Mn or Pb showed a substantial root surface enrichment, which may considerably disturb the computation of the tests. However, the MCD procedure may not remove all of the datapoints related to the root surface, but also exclude hotspots or high concentrations occurring within the cementum thickness, which could be related to a biological signal and, thus, be meaningful. Therefore, the classical and robust tests were also run by excluding the datapoints of the root surface and sub-surface defined by a peak in Pb, Mn or Fe, to assess if the significance of the correlation tests would be affected and change. All detailed results are reported below, and the classical Pearson results are plotted in blue while the MCD correlation is plotted in red. Correlation coefficient matrices were computed using the Spearman method, and plotted using 'corr.plot', which enables displaying the rho values following a colour code, and crossing out rho values when p-values are not significant.

References

Boesenberg, U., Ryan, C.G., Kirkham, R., Siddons, D.P., Alfeld, M., Garrevoet, J., Núñez, T., Claussen, T., Kracht, T., Falkenberg, G., 2016. Fast X-ray microfluorescence imaging with submicrometer-resolution integrating a Maia detector at beamline P06 at PETRA III. J Synchrotron Rad 23, 1550–1560. https://doi.org/10.1107/S1600577516015289

Djomehri, S.I., Candell, S., Case, T., Browning, A., Marshall, G.W., Yun, W., Lau, S.H., Webb, S., Ho, S.P., 2015. Mineral density volume gradients in normal and diseased human tissues. PLoS ONE 10, e0121611. https://doi.org/10.1371/journal.pone.0121611

Falkenberg, G., Fleissner, Gerta, Fleissner, Guenther, Alraun, P., Boesenberg, U., Spiers, K., 2017. Large-scale high-resolution micro-XRF analysis of histological structures in the skin of the pigeon beak. X-Ray Spectrom. 46, 467–473. https://doi.org/10.1002/xrs.2769

Hubert, M., Debruyne, M., Rousseeuw, P.J., 2018. Minimum covariance determinant and extensions. WIREs Computational Statistics 10, e1421. https://doi.org/10.1002/wics.1421

Rousseeuw, P.J., Driessen, K.V., 1999. A fast algorithm for the minimum covariance determinant estimator. Technometrics 41, 212–223. https://doi.org/10.1080/00401706.1999.10485670

Santos, F., 2020. Modern methods for old data: An overview of some robust methods for outliers detection with applications in osteology. J. Archaeol. Sci. Rep. 32, 102423. https://doi.org/10.1016/j.jasrep.2020.102423

Schroer, C., Boye, P., Feldkamp, J., Patommel, J., Samberg, D., Schropp, A., Schwab, A., Stephan, S., Falkenberg, G., Wellenreuther, G., Reimers, N., 2010. Hard X-ray nanoprobe at beamline P06 at PETRA III. Nucl. Instrum. Meth. A 616, 93–97. https://doi.org/10.1063/1.4952830

Solé, V.A., Papillon, E., Cotte, M., Walter, P., Susini, J., 2007. A multiplatform code for the analysis of energy-dispersive X-ray fluorescence spectra. Spectrochim. Acta Part B 62, 63–68. https://doi.org/10.1016/j.sab.2006.12.002

Szczerbowska-Boruchowska, M., 2012. Sample thickness considerations for quantitative X-ray fluorescence analysis of the soft and skeletal tissues of the human body - theoretical evaluation and experimental validation. X-Ray Spectrom. 41, 328–337. https://doi.org/10.1002/xrs.2407

2. Results

Statistical analysis

Næstved 211C – Cellular Cementum (total thickness=721 µm)

Correlations between pairs of elements

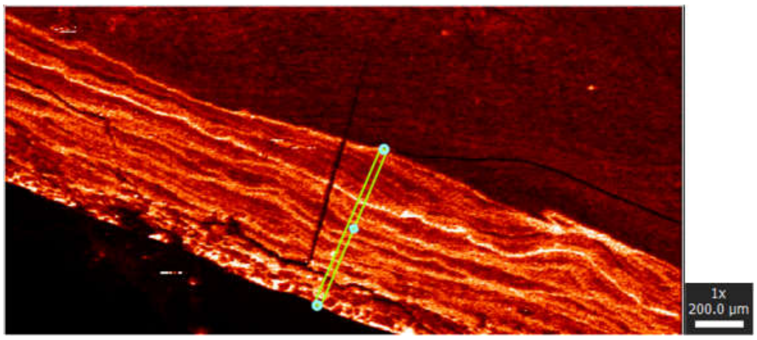

Zn map (yellow path indicates where the measurements are taken)

Summary: whole cementum thickness, Spearman correlations.

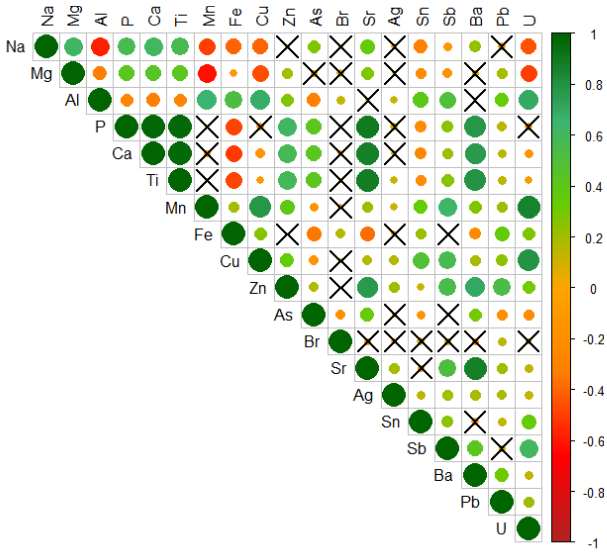

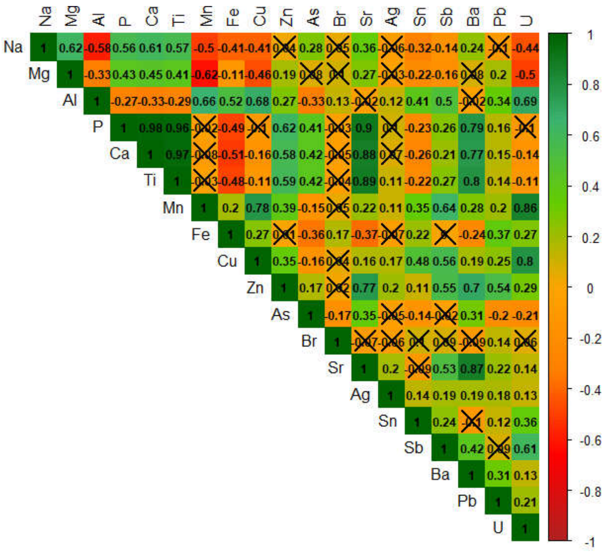

Summary: removing the first 30 µm of root surface, Spearman correlations.

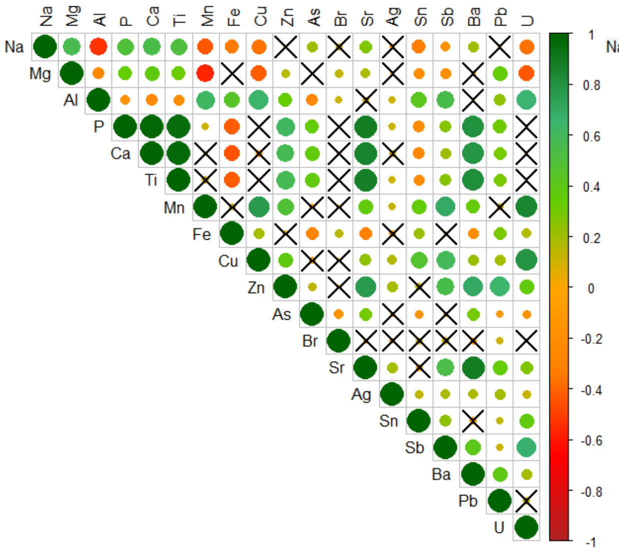

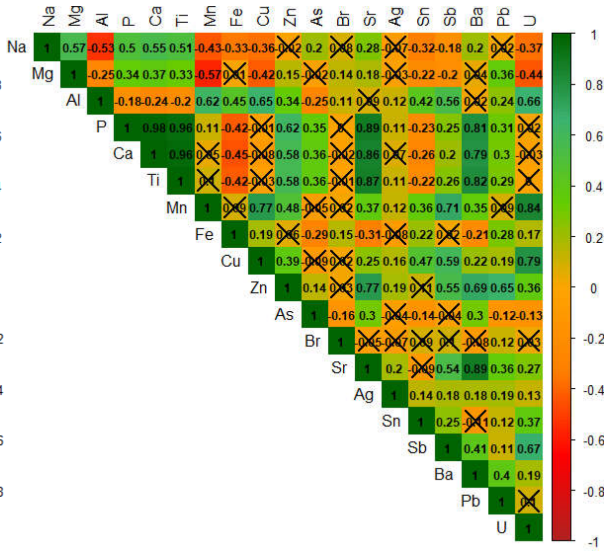

wRS: without Root surface [Pb = ~27 µm; Fe = ~15µm; Mn = ~200µm; Al = ~17 µm]

- **Ca vs Ba**

Classical test	Pearson's product-moment correlation<br><br>data: Aa211C_CC$Ca and Aa211C_CC$Ba<br>t = 16.927, df = 338, **p-value < 2.2e-16**<br>alternative hypothesis: true correlation is not equal to 0<br>95 percent confidence interval:<br>0.6153113 0.7310395<br>sample estimates:<br>**cor**<br>**0.6773442**	Spearman's rank correlation rho<br><br>data: Aa211C_CC$Ca and Aa211C_CC$Ba<br>S = 1527564, **p-value < 2.2e-16**<br>alternative hypothesis: true rho is not equal to 0<br>sample estimates:<br>**rho**<br>**0.7668058**
MCD (α=0.05; quant=0.8)	$cor.rob<br>**0.7691947**	

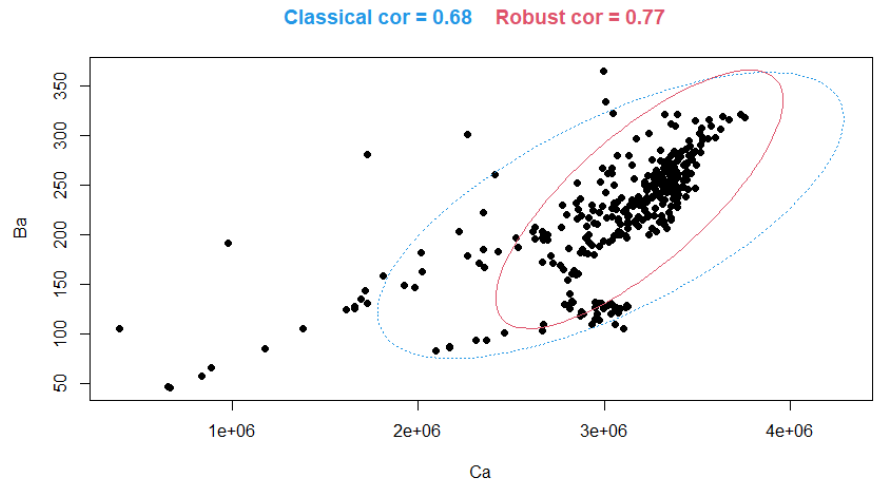

- **Ca vs Sr**

Classical test	Pearson's product-moment correlation<br><br>data: Aa211C_CC$Ca and Aa211C_CC$Sr<br>t = 43.337, df = 338, **p-value < 2.2e-16**<br>alternative hypothesis: true correlation is not equal to 0<br>95 percent confidence interval: 0.9026035 0.9353623<br>sample estimates:<br>**cor**<br>**0.9205867**	Spearman's rank correlation rho<br><br>data: Aa211C_CC$Ca and Aa211C_CC$Sr<br>S = 815088, **p-value < 2.2e-16**<br>alternative hypothesis: true rho is not equal to 0<br>sample estimates:<br>**rho**<br>**0.8755707**
MCD (α=0.05; quant=0.8)	$cor.rob<br>[1] **0.8612135**	

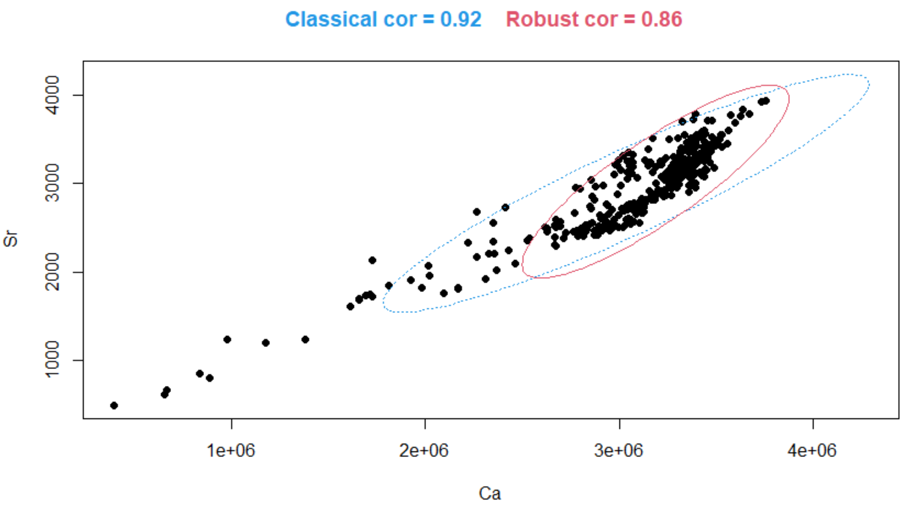

- **Ba vs Sr**

Classical test	Pearson's product-moment correlation<br><br>data: Aa211C_CC$Ba and Aa211C_CC$Sr<br>t = 29, df = 338, **p-value < 2.2e-16**<br>alternative hypothesis: true correlation is not equal to 0<br>95 percent confidence interval: 0.8110749 0.8725568<br>sample estimates:<br>**cor**<br>**0.8445774**	Spearman's rank correlation rho<br><br>data: Aa211C_CC$Ba and Aa211C_CC$Sr<br>S = 844406, **p-value < 2.2e-16**<br>alternative hypothesis: true rho is not equal to 0<br>sample estimates:<br>**rho**<br>**0.8710951**
MCD (α=0.05; quant=0.8)	$cor.rob<br>[1] **0.8781067**	

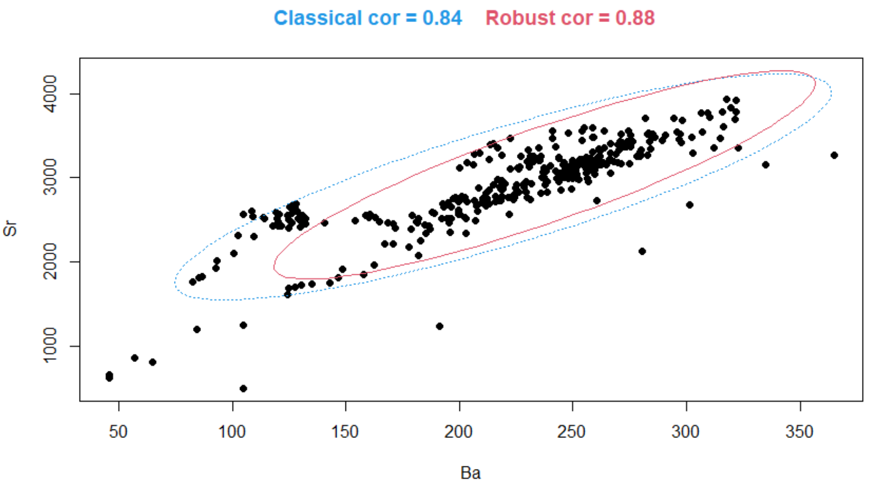

- **Zn vs Sr**

Classical test	Pearson's product-moment correlation<br><br>data: Aa211C_CC$Zn and Aa211C_CC$Sr<br>t = 20.166, df = 338, **p-value < 2.2e-16**<br>alternative hypothesis: true correlation is not equal to 0<br>95 percent confidence interval:<br>0.6865859 0.7837428<br>sample estimates:<br>**cor**<br>**0.7389826**	Spearman's rank correlation rho<br><br>data: Aa211C_CC$Zn and Aa211C_CC$Sr<br>S = 1506568, **p-value < 2.2e-16**<br>alternative hypothesis: true rho is not equal to 0<br>sample estimates:<br>**rho**<br>**0.770011**
MCD (α=0.05; quant=0.8)	$cor.rob<br>[1] **0.7459763**	

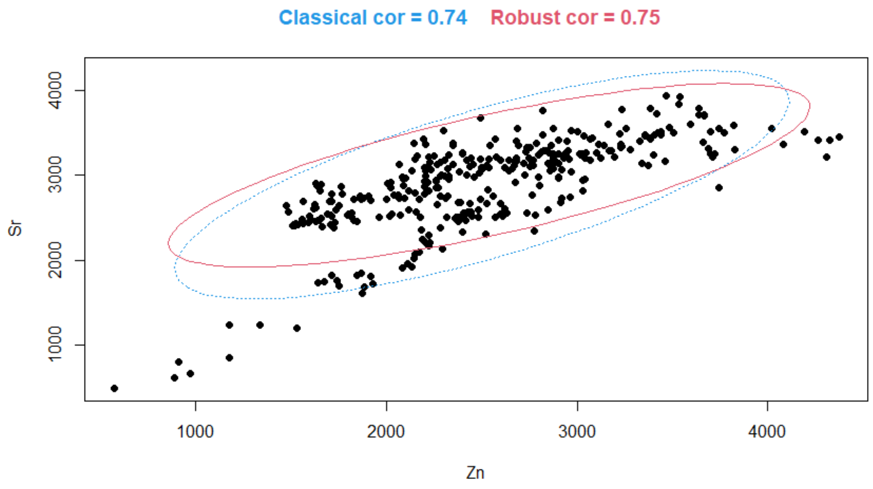

- **Mg vs Sr**

Classical test	Pearson's product-moment correlation<br><br>data: Aa211C_CC$Mg and Aa211C_CC$Sr<br>t = 9.5244, df = 338, **p-value < 2.2e-16**<br>alternative hypothesis: true correlation is not equal to 0<br>95 percent confidence interval:<br>0.3718262 0.5399413<br>sample estimates:<br>**cor**<br>**0.4599964**	Spearman's rank correlation rho<br><br>data: Aa211C_CC$Mg and Aa211C_CC$Sr<br>S = 4795796, **p-value = 5.916e-07**<br>alternative hypothesis: true rho is not equal to 0<br>sample estimates:<br>**rho**<br>**0.2678856**
MCD (α=0.05; quant=0.8)	$cor.rob<br>[1] **0.607433**	

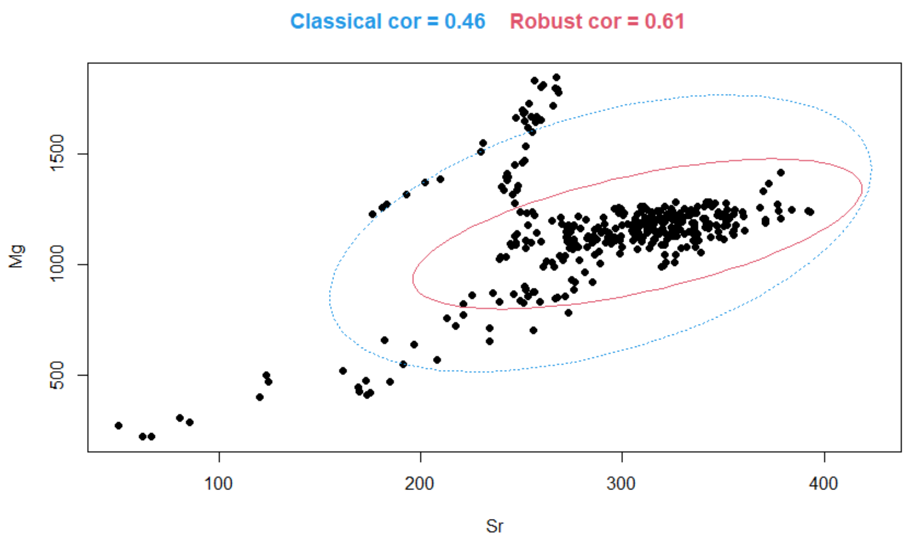

- **Pb vs Sr**

Whole cementum thickness:

Classical test	Pearson's product-moment correlation<br><br>data: Aa211C_CC$Sr and Aa211C_CC$Pb<br>t = -1.9403, df = 338, **p-value = 0.05318**<br>alternative hypothesis: true correlation is not equal to 0<br>95 percent confidence interval:<br>-0.20898408 0.00142329<br>sample estimates:<br>**cor**<br>**-0.1049548**	Spearman's rank correlation rho<br><br>data: Aa211C_CC$Sr and Aa211C_CC$Pb<br>S = 5138998, **p-value = 6.42e-05**<br>alternative hypothesis: true rho is not equal to 0<br>sample estimates:<br>**rho**<br>**0.2154932**
MCD (α=0.05; quant=0.8)	$cor.rob<br>[1] **0.2704948**	

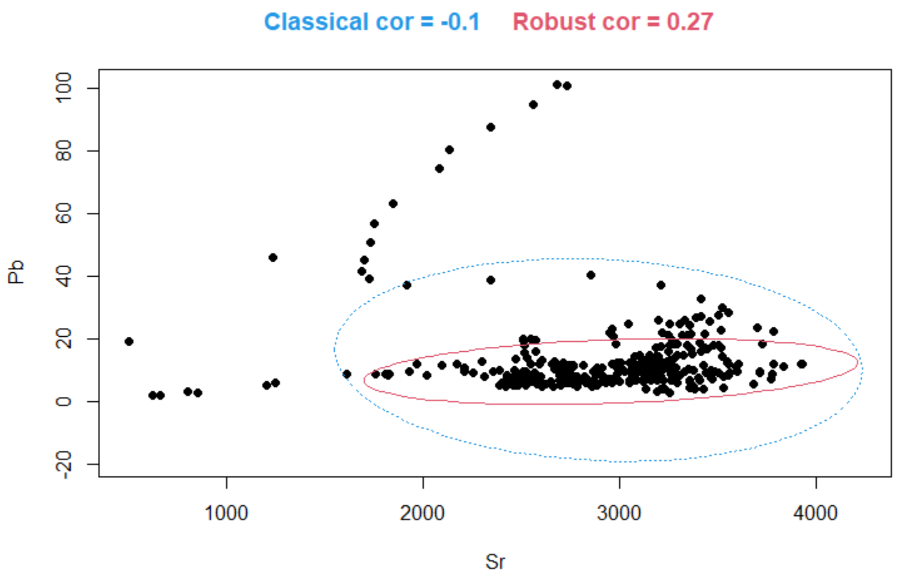

Removing 27 µm of Pb-enriched root surface:

Classical test	Pearson's product-moment correlation<br><br>data: Aa211C_CC_wRS$Sr and Aa211C_CC_wRS$Pb<br>t = 4.4666, df = 325, **p-value = 1.098e-05**<br>alternative hypothesis: true correlation is not equal to 0<br>95 percent confidence interval:<br>0.1355669 0.3400778<br>sample estimates:<br>**cor**<br>**0.2404895**	Spearman's rank correlation rho<br><br>data: Aa211C_CC_wRS$Zn and Aa211C_CC_wRS$Pb<br>S = 2159708, **p-value < 2.2e-16**<br>alternative hypothesis: true rho is not equal to 0<br>sample estimates:<br>**rho**<br>**0.6293986**
MCD (α=0.05; quant=0.8)	$cor.rob<br>[1] **0.2800147**	

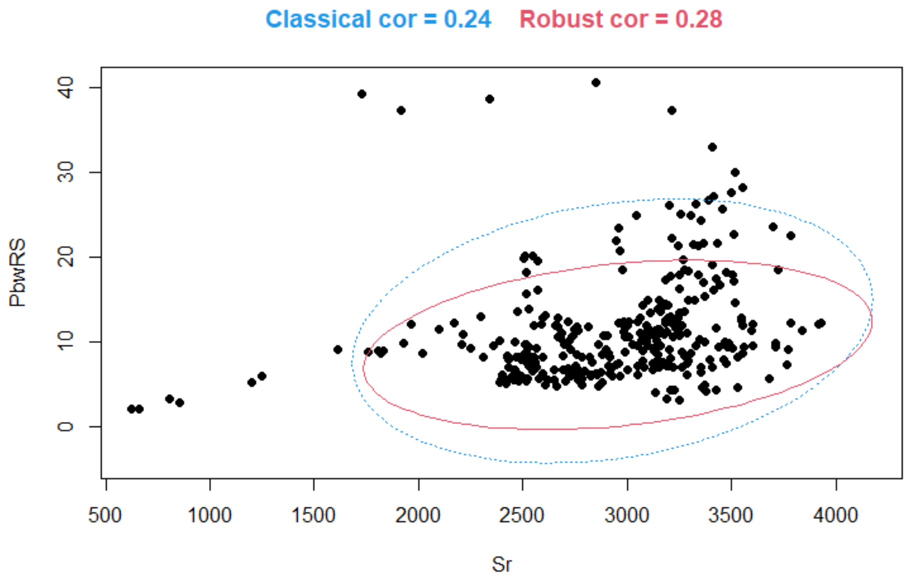

- **Sr vs Zn**

Classical test	Pearson's product-moment correlation<br><br>data: Aa211C_CC$Zn and Aa211C_CC$Sr<br>t = 20.166, df = 338, **p-value < 2.2e-16**<br>alternative hypothesis: true correlation is not equal to 0<br>95 percent confidence interval:<br>0.6865859 0.7837428<br>sample estimates:<br>**cor**<br>**0.7389826**	Spearman's rank correlation rho<br><br>data: Aa211C_CC$Zn and Aa211C_CC$Sr<br>S = 1506568, **p-value < 2.2e-16**<br>alternative hypothesis: true rho is not equal to 0<br>sample estimates:<br>**rho**<br>**0.770011**
MCD (α=0.05; quant=0.8)	$cor.rob<br>[1] **0.7459763**	

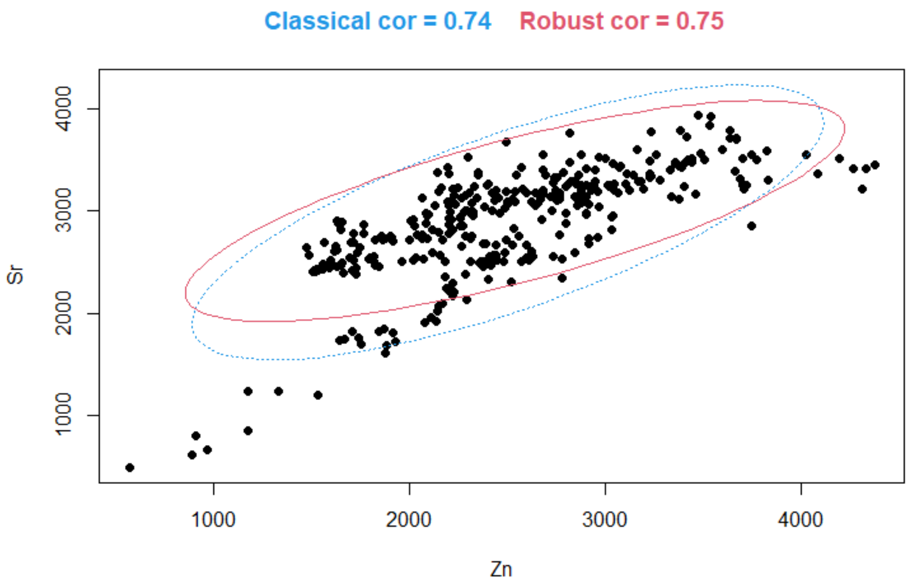

- **Pb vs Zn**

Whole cementum thickness:

Classical test	Pearson's product-moment correlation<br><br>data: Aa211C_CC$Zn and Aa211C_CC$Pb<br>t = 4.2406, df = 338, **p-value = 2.88e-05**<br>alternative hypothesis: true correlation is not equal to 0<br>95 percent confidence interval:<br>0.1212964 0.3233902<br>sample estimates:<br>**cor**<br>**0.2247589**	Spearman's rank correlation rho<br><br>data: Aa211C_CC$Zn and Aa211C_CC$Pb<br>S = 3010790, **p-value < 2.2e-16**<br>alternative hypothesis: true rho is not equal to 0<br>sample estimates:<br>**rho**<br>**0.5403802**
MCD (α=0.05; quant=0.8)	$cor.rob<br>[1] **0.5789923**	

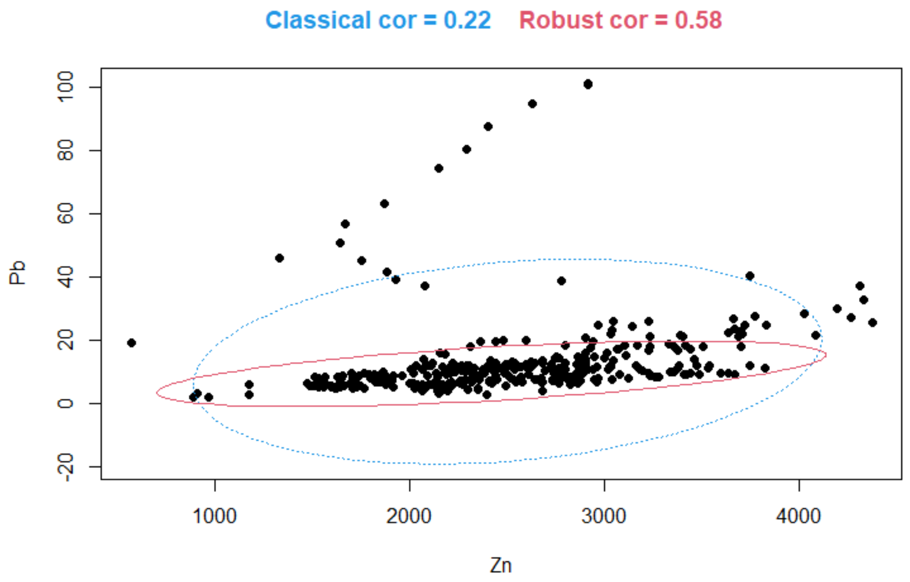

Removing 27 µm of Pb-enriched root surface:

	Pearson's product-moment correlation	Spearman's rank correlation rho
Classical test	data: Aa211C_CC_wRS$Zn and Aa211C_CC_wRS$Pb<br>t = 13.887, df = 325, **p-value < 2.2e-16**<br>alternative hypothesis: true correlation is not equal to 0<br>95 percent confidence interval:<br>0.5373619 0.6740960<br>sample estimates:<br>**cor**<br>**0.610254**	data: Aa211C_CC_wRS$Zn and Aa211C_CC_wRS$Pb<br>S = 2159708, **p-value < 2.2e-16**<br>alternative hypothesis: true rho is not equal to 0<br>sample estimates:<br>**rho**<br>**0.6293986**
MCD (α=0.05; quant=0.8)	$cor.rob<br>[1] **0.5752824**	

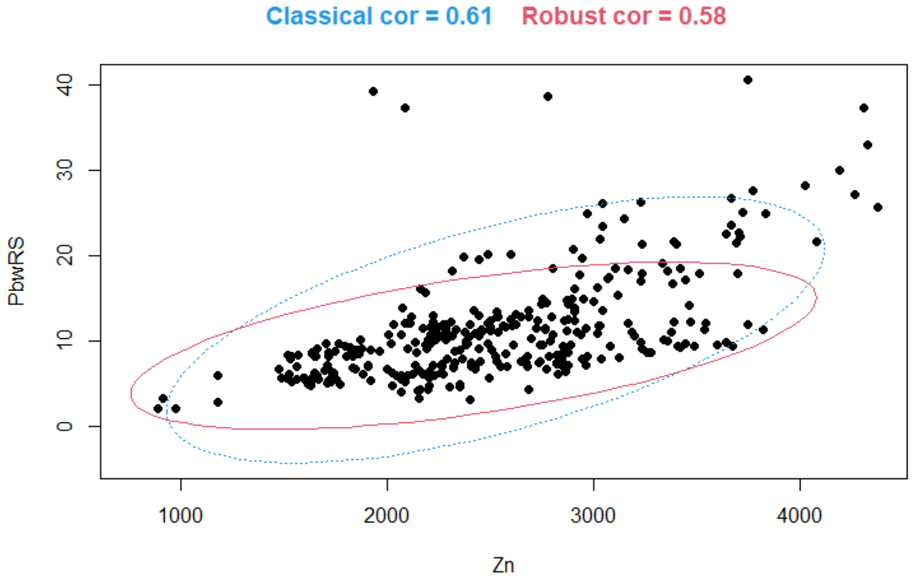

- **Zn vs Ca**

	Pearson's product-moment correlation	Spearman's rank correlation rho
Classical test	data: Aa211C_CC$Zn and Aa211C_CC$Ca<br>t = 12.248, df = 338, **p-value < 2.2e-16**<br>alternative hypothesis: true correlation is not equal to 0<br>95 percent confidence interval:<br>0.4761334 0.6239848<br>sample estimates:<br>**cor**<br>**0.5544184**	data: Aa211C_CC$Zn and Aa211C_CC$Ca<br>S = 2718964, **p-value < 2.2e-16**<br>alternative hypothesis: true rho is not equal to 0<br>sample estimates:<br>**rho**<br>**0.5849296**
MCD (α=0.05; quant=0.8)	$cor.rob<br>[1] **0.5666424**	

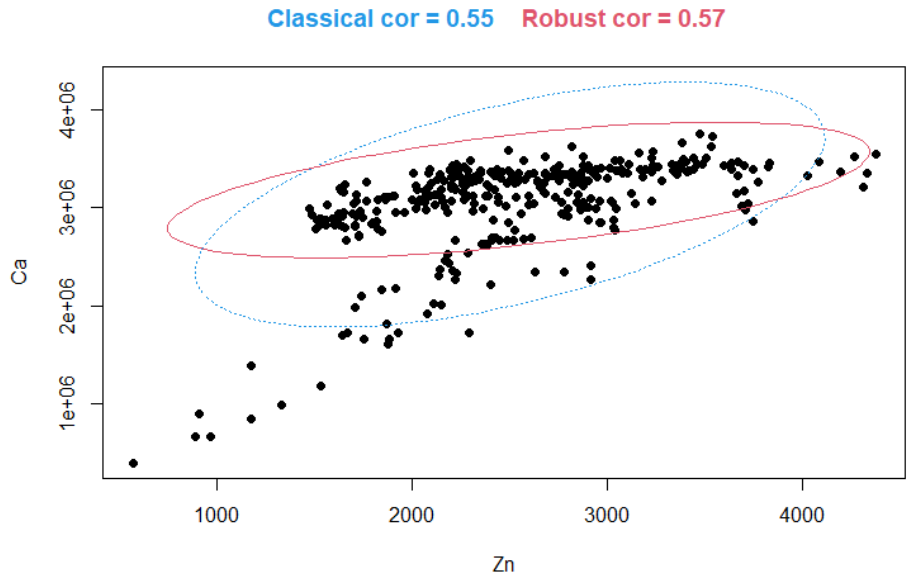

- **Zn vs Fe**

Whole cementum thickness:

Classical test	Pearson's product-moment correlation<br><br>data: Aa211C_CC$Zn and Aa211C_CC$Fe<br>t = -0.79887, df = 338, **p-value = 0.4249**<br>alternative hypothesis: true correlation is not equal to 0<br>95 percent confidence interval:<br>-0.14908558 0.06324246<br>sample estimates:<br>**cor**<br>**-0.04341176**	Spearman's rank correlation rho<br><br>data: Aa211C_CC$Zn and Aa211C_CC$Fe<br>S = 6501830, **p-value = 0.8911**<br>alternative hypothesis: true rho is not equal to 0<br>sample estimates:<br>**rho**<br>**0.007446635**
MCD (α=0.05; quant=0.8)	$cor.rob<br>[1] **-0.1496604**	

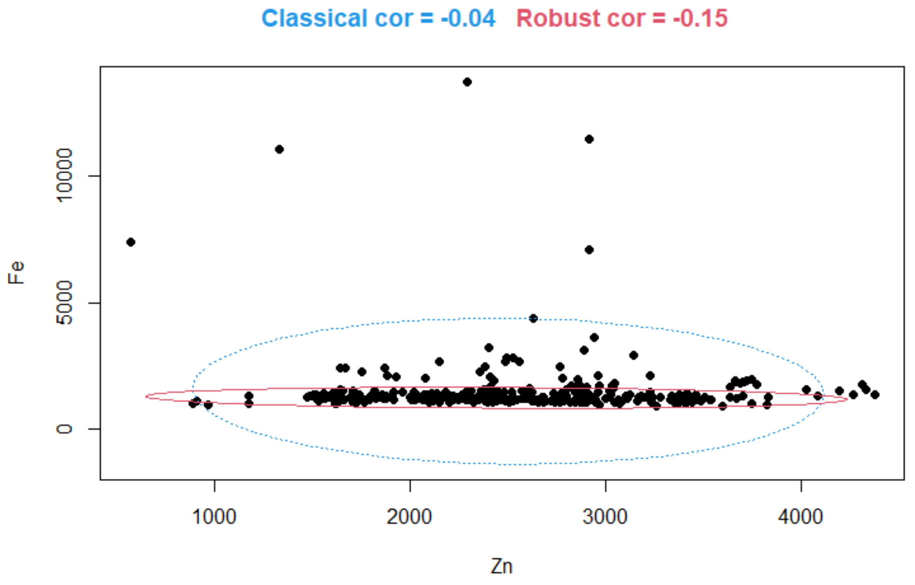

Removing 15 µm of Fe-enriched root surface:

Classical test	Pearson's product-moment correlation<br><br>data: Aa211C_CC$Zn and Aa211C_CC$Fe<br>t = -0.79887, df = 338, **p-value = 0.4249**<br>alternative hypothesis: true correlation is not equal to 0<br>95 percent confidence interval:<br>-0.14908558 0.06324246<br>sample estimates:<br>**cor**<br>**-0.04341176**	Spearman's rank correlation rho<br><br>data: Aa211C_CC$Zn and Aa211C_CC$Fe<br>S = 6501830, **p-value = 0.8911**<br>alternative hypothesis: true rho is not equal to 0<br>sample estimates:<br>**rho**<br>**0.007446635**
MCD (α=0.05; quant=0.8)	$cor.rob<br>[1] **-0.1496604**	

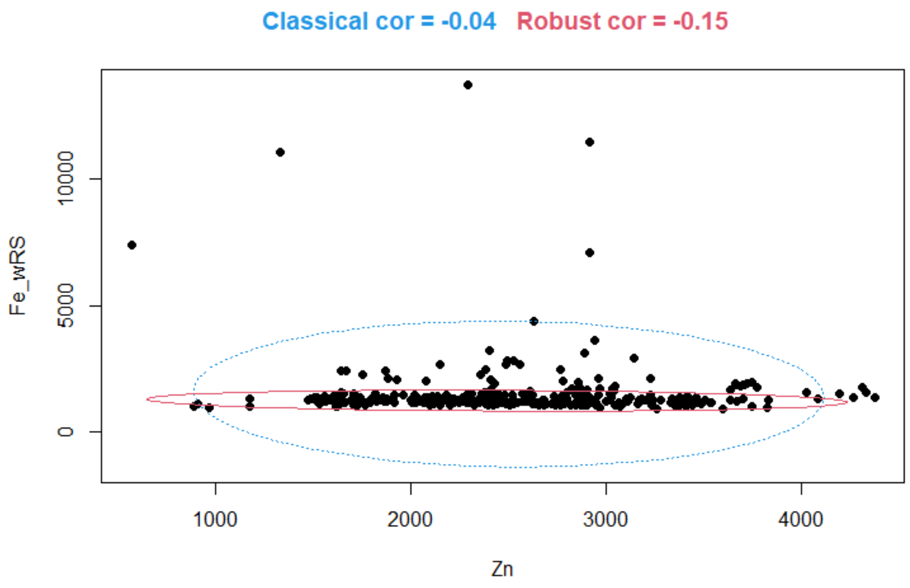

- **Zn vs Cu**

Classical test	Pearson's product-moment correlation<br><br>data: Aa211C_CC$Zn and Aa211C_CC$Cu<br>t = 3.6814, df = 338, **p-value = 0.0002699**<br>alternative hypothesis: true correlation is not equal to 0<br>95 percent confidence interval:<br>0.09190018 0.29651317<br>sample estimates:<br>**cor**<br>**0.1963432**	Spearman's rank correlation rho<br><br>data: Aa211C_CC$Zn and Aa211C_CC$Cu<br>S = 4278960, **p-value = 6.446e-11**<br>alternative hypothesis: true rho is not equal to 0<br>sample estimates:<br>**rho**<br>**0.3467845**
MCD (α=0.05; quant=0.8)	$cor.rob<br>[1] **0.4401857**	

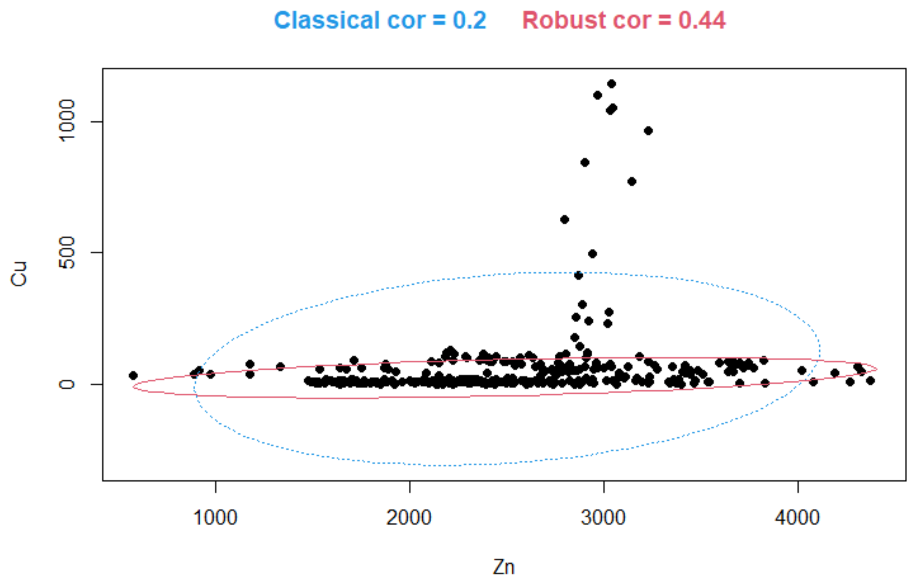

- **Zn vs Mg**

Classical test	Pearson's product-moment correlation<br><br>data: Aa211C_CC$Zn and Aa211C_CC$Mg<br>t = 4.7487, df = 338, **p-value = 3.03e-06**<br>alternative hypothesis: true correlation is not equal to 0<br>95 percent confidence interval:<br>0.1476523 0.3472134<br>sample estimates:<br>**cor**<br>**0.250087**	Spearman's rank correlation rho<br><br>data: Aa211C_CC$Zn and Aa211C_CC$Mg<br>S = 5277330, **p-value = 0.0003195**<br>alternative hypothesis: true rho is not equal to 0<br>sample estimates:<br>**rho**<br>**0.1943758**
MCD (α=0.05; quant=0.8)	$cor.rob<br>[1] **0.08211205**	

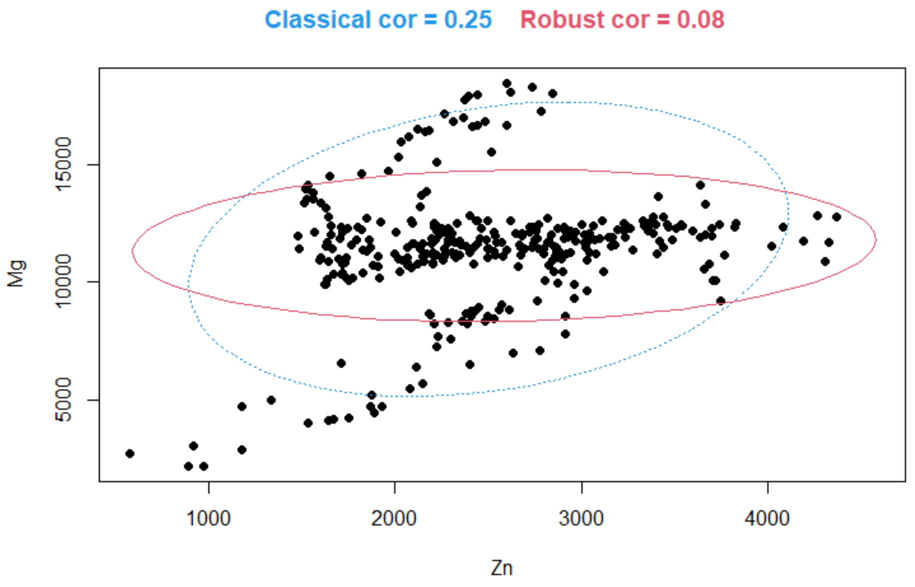

- **Ca vs Fe**

Whole cementum thickness:

Classical test	Pearson's product-moment correlation<br><br>data: Aa211C_CC$Ca and Aa211C_CC$Fe<br>t = -8.4272, df = 338, **p-value = 1.033e-15**<br>alternative hypothesis: true correlation is not equal to 0<br>95 percent confidence interval:<br>-0.5008532 -0.3247181<br>sample estimates:<br>**cor**<br>**-0.4166888**	Spearman's rank correlation rho<br><br>data: Aa211C_CC$Ca and Aa211C_CC$Fe<br>S = 9906092, **p-value < 2.2e-16**<br>alternative hypothesis: true rho is not equal to 0<br>sample estimates:<br>**rho**<br>**-0.5122396**
MCD (α=0.05; quant=0.8)	$cor.rob<br>[1] **-0.4999543**	

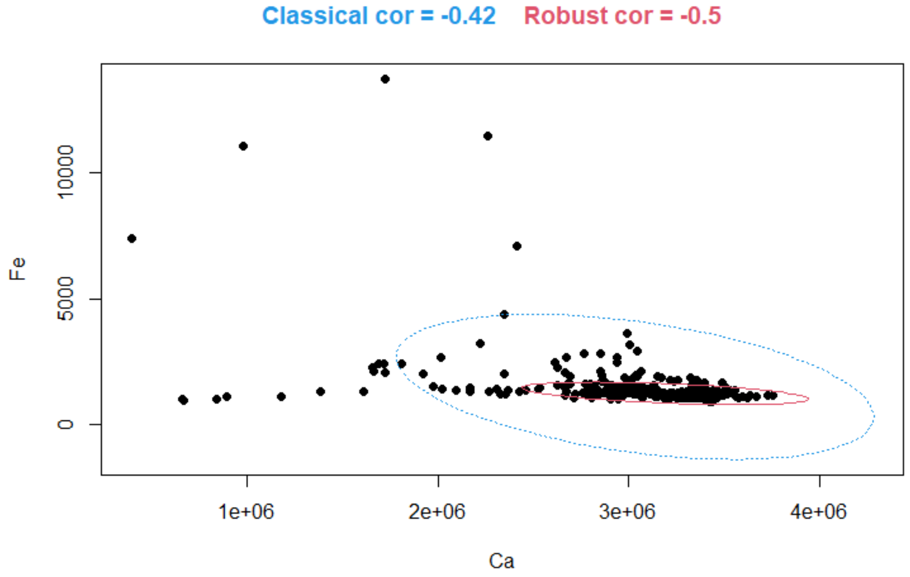

Removing 15 µm of Fe-enriched root surface:

Classical test	Pearson's product-moment correlation<br><br>data: Aa211C_CC$Ca and Aa211C_CC$Fe<br>t = -8.4272, df = 338, **p-value = 1.033e-15**<br>alternative hypothesis: true correlation is not equal to 0<br>95 percent confidence interval:<br>-0.5008532 -0.3247181<br>sample estimates:<br>**cor**<br>**-0.4166888**	Spearman's rank correlation rho<br><br>data: Aa211C_CC$Ca and Aa211C_CC$Fe<br>S = 9906092, **p-value < 2.2e-16**<br>alternative hypothesis: true rho is not equal to 0<br>sample estimates:<br>**rho**<br>**-0.5122396**
MCD (α=0.05; quant=0.8)	$cor.rob<br>[1] **-0.4999543**	

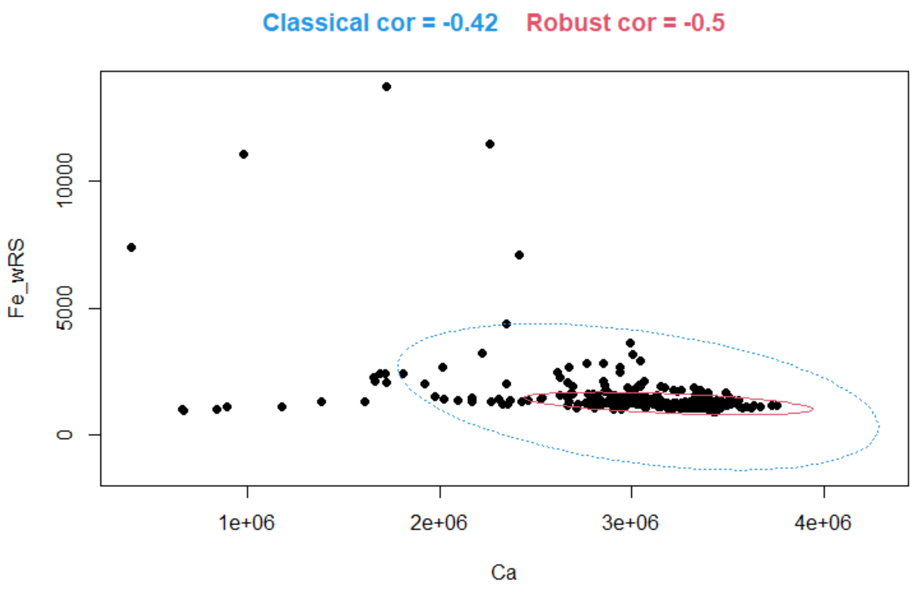

- **Ca vs Mg**

Classical test	Pearson's product-moment correlation<br><br>data: Aa211C_CC$Ca and Aa211C_CC$Mg<br>t = 16.104, df = 338, **p-value < 2.2e-16**<br>alternative hypothesis: true correlation is not equal to 0<br>95 percent confidence interval:<br>0.5941831 0.7151465<br>sample estimates:<br>**cor**<br>**0.6589035**	Spearman's rank correlation rho<br><br>data: Aa211C_CC$Ca and Aa211C_CC$Mg<br>S = 3592892, **p-value < 2.2e-16**<br>alternative hypothesis: true rho is not equal to 0<br>sample estimates:<br>**rho**<br>**0.451518**
MCD (α=0.05; quant=0.8)	$cor.rob<br>[1] **0.7879131**	

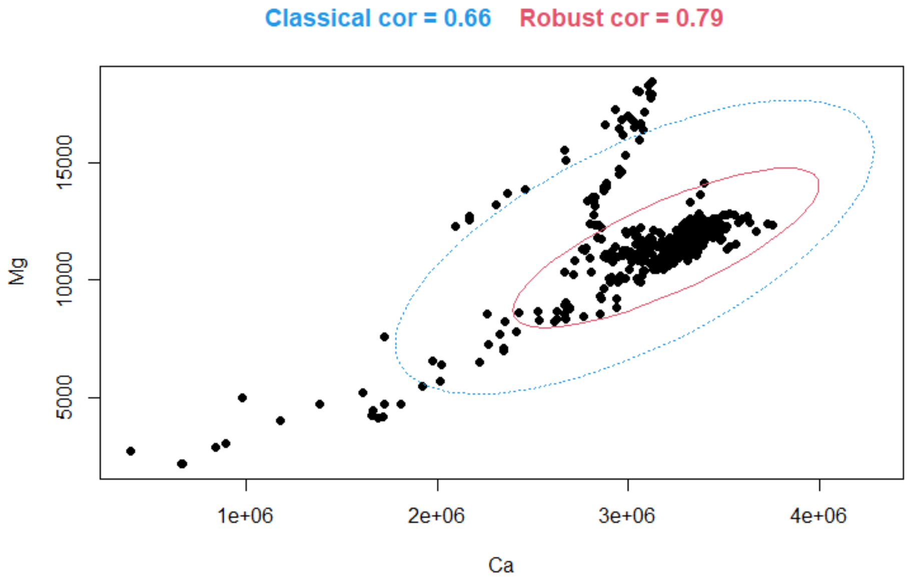

- **Ca vs P**

Classical test	Pearson's product-moment correlation<br><br>data: Aa211C_CC$Ca and Aa211C_CC$P<br>t = 175.03, df = 338, **p-value < 2.2e-16**<br>alternative hypothesis: true correlation is not equal to 0<br>95 percent confidence interval:<br>0.9932310 0.9955785<br>sample estimates:<br>**cor**<br>**0.9945289**	Spearman's rank correlation rho<br><br>data: Aa211C_CC$Ca and Aa211C_CC$P<br>S = 112302, **p-value < 2.2e-16**<br>alternative hypothesis: true rho is not equal to 0<br>sample estimates:<br>**rho**<br>**0.9828563**
MCD (α=0.05; quant=0.8)	$cor.rob<br>[1] **0.9880191**	

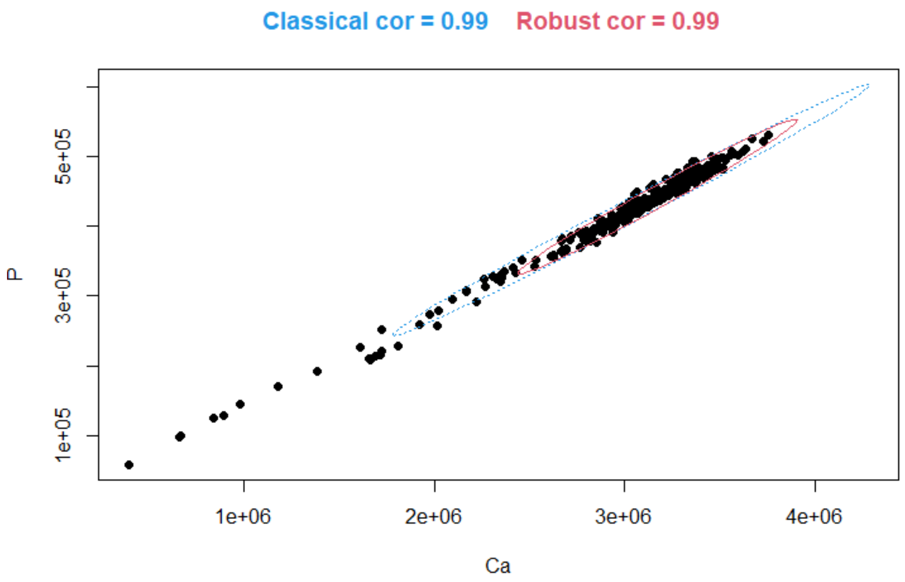

- **Cu vs Fe**

Whole cementum thickness:

Classical test	Pearson's product-moment correlation<br><br>data: Aa211C_CC$Cu and Aa211C_CC$Fe<br>t = 2.5683, df = 338, **p-value = 0.01065**<br>alternative hypothesis: true correlation is not equal to 0<br>95 percent confidence interval:<br>0.03246759 0.24116538<br>sample estimates:<br>**cor**<br>**0.138352**	Spearman's rank correlation rho<br><br>data: Aa211C_CC$Cu and Aa211C_CC$Fe<br>S = 4806092, **p-value = 6.913e-07**<br>alternative hypothesis: true rho is not equal to 0<br>sample estimates:<br>**rho**<br>**0.2663138**
MCD (α=0.05; quant=0.8)	$cor.rob<br>[1] **-0.2079025**	

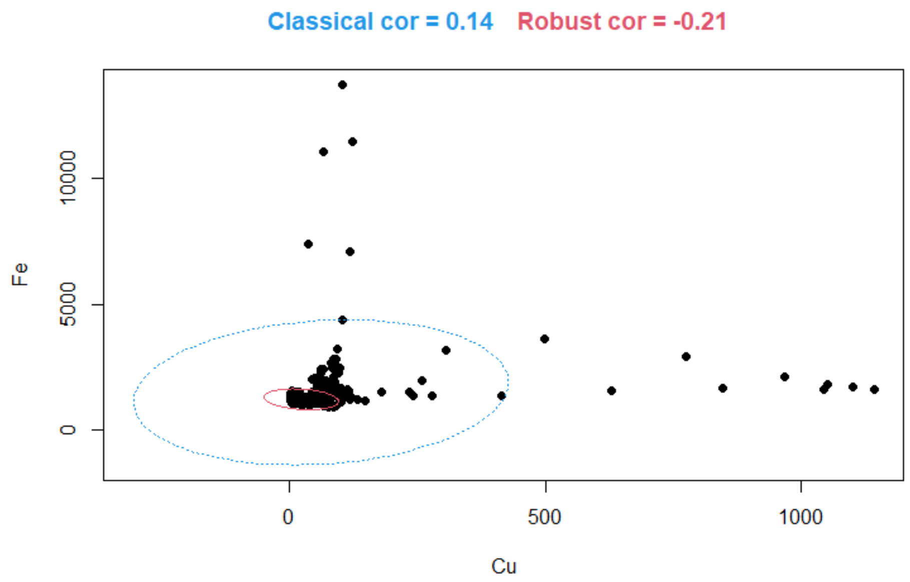

Removing 15 µm of Fe-enriched root surface:

Classical test	Pearson's product-moment correlation<br><br>data: Aa211C_CC$Cu and Aa211C_CC$Fe<br>t = 2.5683, df = 338, **p-value = 0.01065**<br>alternative hypothesis: true correlation is not equal to 0<br>95 percent confidence interval:<br>0.03246759 0.24116538<br>sample estimates:<br>**cor**<br>**0.138352**	Spearman's rank correlation rho<br><br>data: Aa211C_CC$Cu and Aa211C_CC$Fe<br>S = 4806092, **p-value = 6.913e-07**<br>alternative hypothesis: true rho is not equal to 0<br>sample estimates:<br>**rho**<br>**0.2663138**
MCD (α=0.05; quant=0.8)	$cor.rob<br>[1] **-0.2079025**	

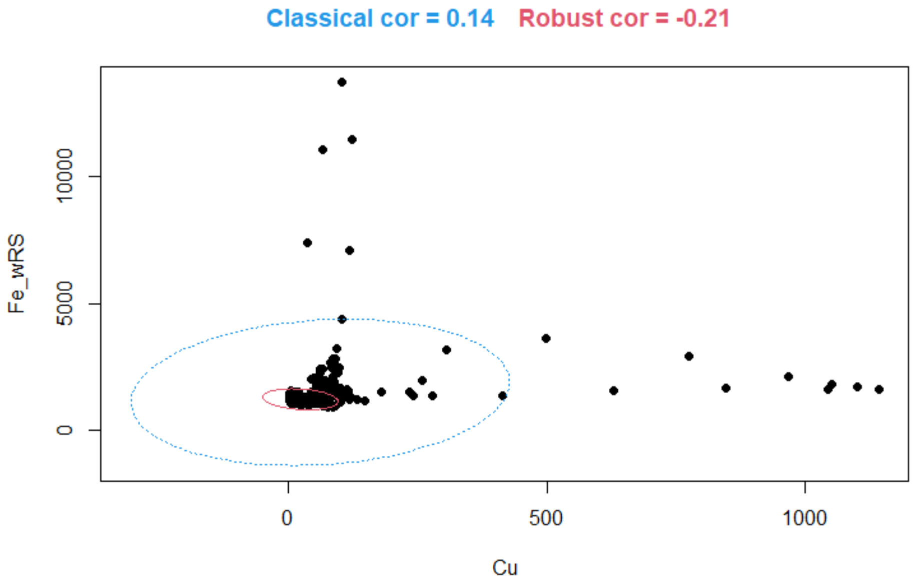

- **Mg vs Fe**

Whole cementum thickness:

Classical test	Pearson's product-moment correlation<br><br>data: Aa211C_CC$Mg and Aa211C_CC$Fe<br>t = -5.8506, df = 338, **p-value = 1.157e-08**<br>alternative hypothesis: true correlation is not equal to 0<br>95 percent confidence interval:<br>-0.3968103 -0.2034466<br>sample estimates:<br>**cor**<br>**-0.3032469**	Spearman's rank correlation rho<br><br>data: Aa211C_CC$Mg and Aa211C_CC$Fe<br>S = 7273132, **p-value = 0.04214**<br>alternative hypothesis: true rho is not equal to 0<br>sample estimates:<br>**rho**<br>**-0.1102984**
MCD (α=0.05; quant=0.8)	$cor.rob<br>[1] **-0.02840057**	

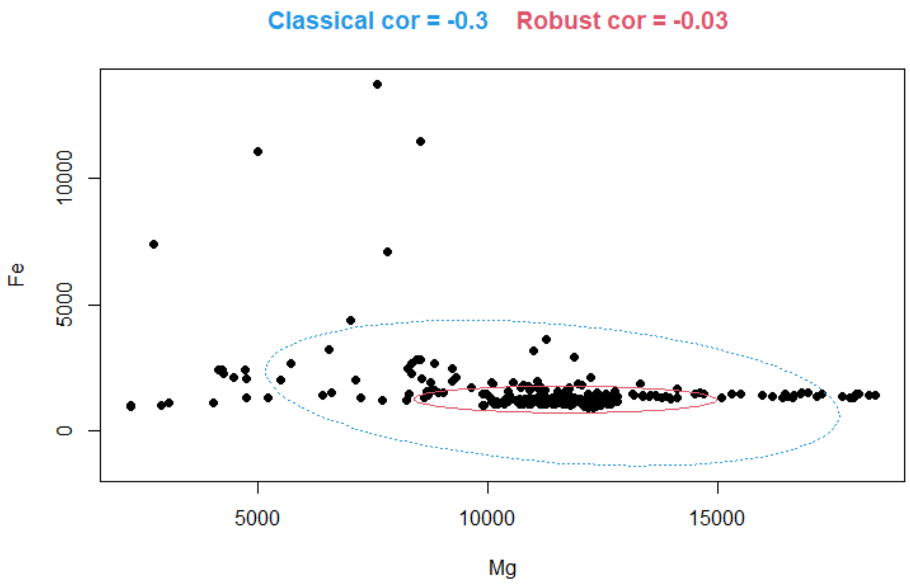

Removing 15 µm of Fe-enriched root surface:

Classical test	Pearson's product-moment correlation<br><br>data: Aa211C_CC$Mg and Aa211C_CC$Fe<br>t = -5.8506, df = 338, **p-value = 1.157e-08**<br>alternative hypothesis: true correlation is not equal to 0<br>95 percent confidence interval:<br>-0.3968103 -0.2034466<br>sample estimates:<br>**cor**<br>**-0.3032469**	Spearman's rank correlation rho<br><br>data: Aa211C_CC$Mg and Aa211C_CC$Fe<br>S = 7273132, **p-value = 0.04214**<br>alternative hypothesis: true rho is not equal to 0<br>sample estimates:<br>**rho**<br>**-0.1102984**
MCD (α=0.05; quant=0.8)	$cor.rob<br>[1] **-0.02840057**	

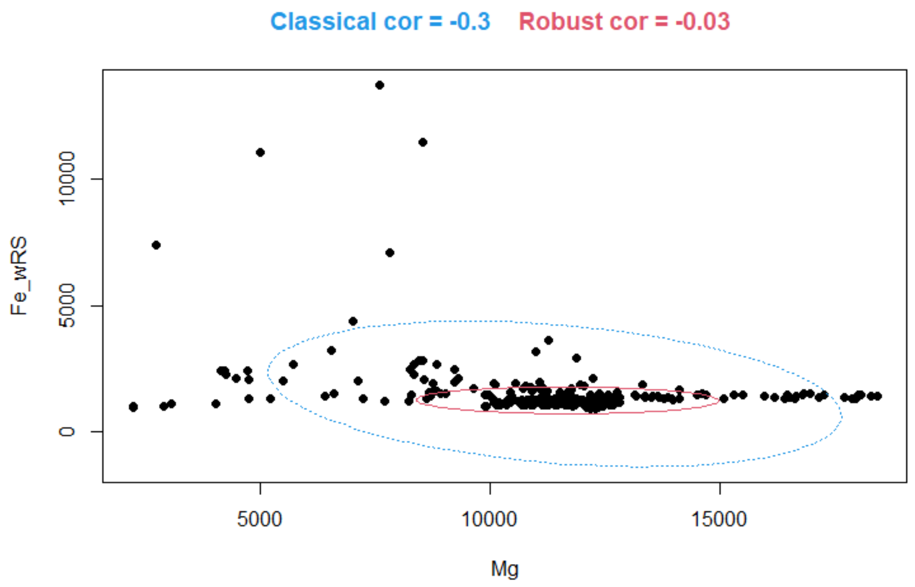

- **Cu vs Mg**

Classical test	Pearson's product-moment correlation<br><br>data: Aa211C_CC$Mg and Aa211C_CC$Cu<br>t = -1.8016, df = 338, **p-value = 0.0725**<br>alternative hypothesis: true correlation is not equal to 0<br>95 percent confidence interval:<br>-0.201796969 0.008926746<br>sample estimates:<br>**cor**<br>**-0.09752806**	Spearman's rank correlation rho<br><br>data: Aa211C_CC$Mg and Aa211C_CC$Cu<br>S = 9576908, **p-value < 2.2e-16**<br>alternative hypothesis: true rho is not equal to 0<br>sample estimates:<br>**rho**<br>**-0.4619872**
MCD (α=0.05; quant=0.8)	$cor.rob<br>[1] **-0.5469069**	

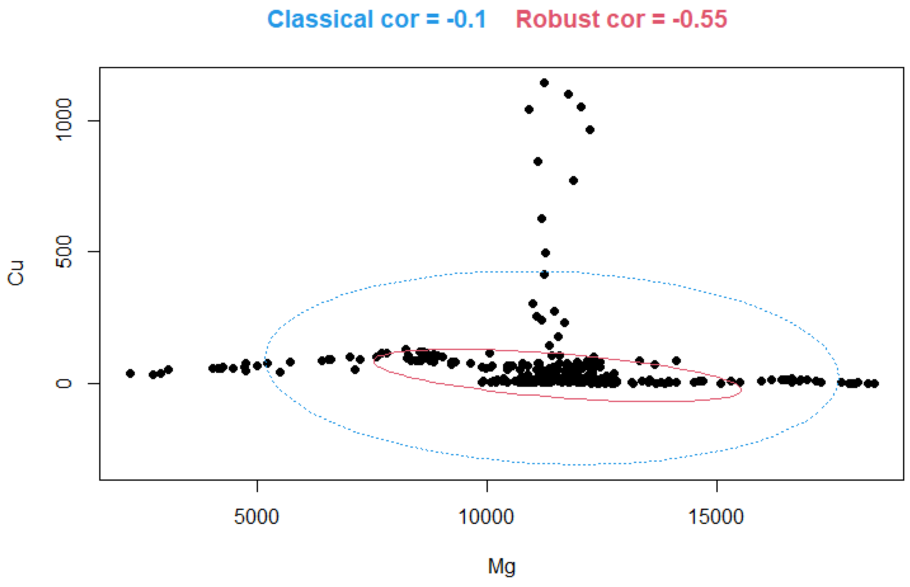

- **As vs Hg, Pb vs Hg (no Hg at all). => Not Applicable.**

- **Pb vs As**

Whole cementum thickness:

	Pearson's product-moment correlation	Spearman's rank correlation rho
Classical test	data: Aa211C_CC$As and Aa211C_CC$Pb<br>t = -2.565, df = 338, **p-value = 0.01075**<br>alternative hypothesis: true correlation is not equal to 0<br>95 percent confidence interval:<br>-0.24099712 -0.03228913<br>sample estimates:<br>**cor**<br>**-0.1381768**	data: Aa211C_CC$As and Aa211C_CC$Pb<br>S = 7893065, **p-value = 0.0001416**<br>alternative hypothesis: true rho is not equal to 0<br>sample estimates:<br>**rho**<br>**-0.2049359**<br>*Impossible to calculate the exact p-value with ex-aequos*
MCD (α=0.05; quant=0.8)	$cor.rob<br>[1] **-0.07379289**	

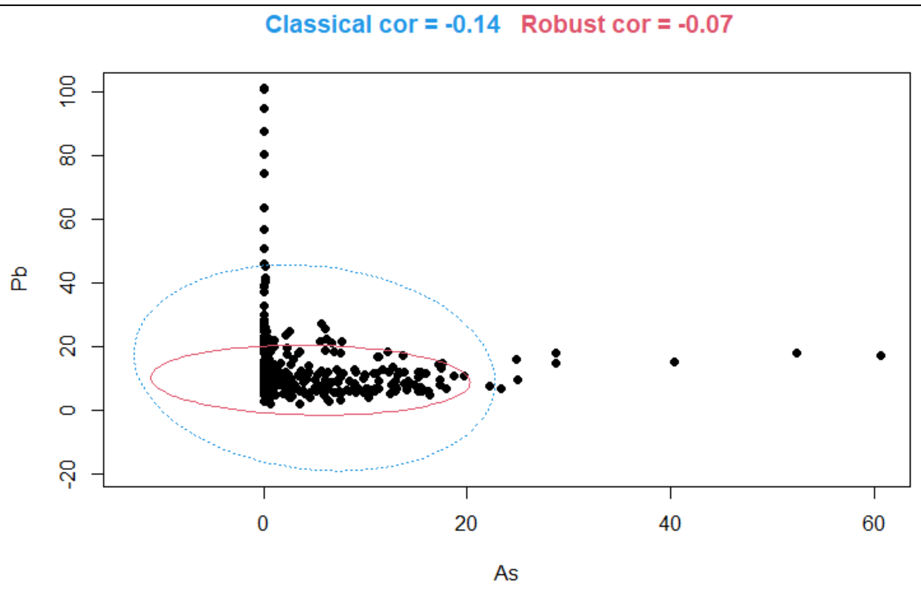

Removing 27 µm of Pb-enriched root surface:

	Pearson's product-moment correlation	Spearman's rank correlation rho
Classical test	data: Aa211C_CC_wRS$As and Aa211C_CC_wRS$Pb<br>t = -1.1211, df = 325, **p-value = 0.2631**<br>alternative hypothesis: true correlation is not equal to 0<br>95 percent confidence interval:<br>-0.16938326 0.04670812<br>sample estimates:<br>**cor**<br>**-0.06206488**	data: Aa211C_CC_wRS$As and Aa211C_CC_wRS$Pb<br>S = 6591902, **p-value = 0.01765**<br>alternative hypothesis: true rho is not equal to 0<br>sample estimates:<br>**rho**<br>**-0.1311567**<br>*Impossible to calculate the exact p-value with ex-aequos*
MCD (α=0.05; quant=0.8)	$cor.rob<br>[1] **-0.08793329**	

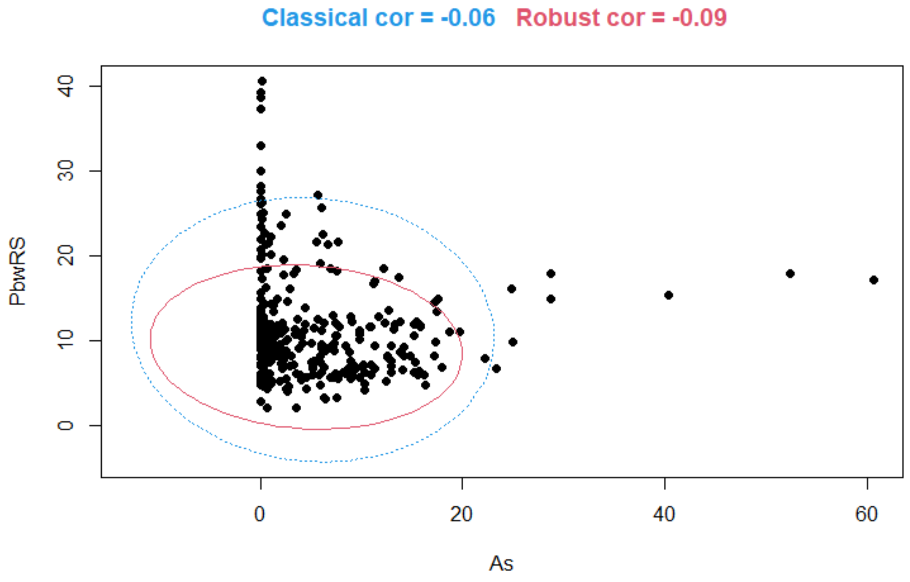

- **Sb vs Sn**

Classical test	Pearson's product-moment correlation<br><br>data: Aa211C_CC$Sn and Aa211C_CC$Sb<br>t = 6.6484, df = 338, **p-value = 1.191e-10**<br>alternative hypothesis: true correlation is not equal to 0<br>95 percent confidence interval:<br>0.2424784 0.4308482<br>sample estimates:<br>**cor**<br>**0.34007**	Spearman's rank correlation rho<br><br>data: Aa211C_CC$Sn and Aa211C_CC$Sb<br>S = 4952632, **p-value = 5.724e-06**<br>alternative hypothesis: true rho is not equal to 0<br>sample estimates:<br>**rho**<br>**0.2439434**
MCD (α=0.05; quant=0.8)	$cor.rob<br>[1] **0.1804737**	

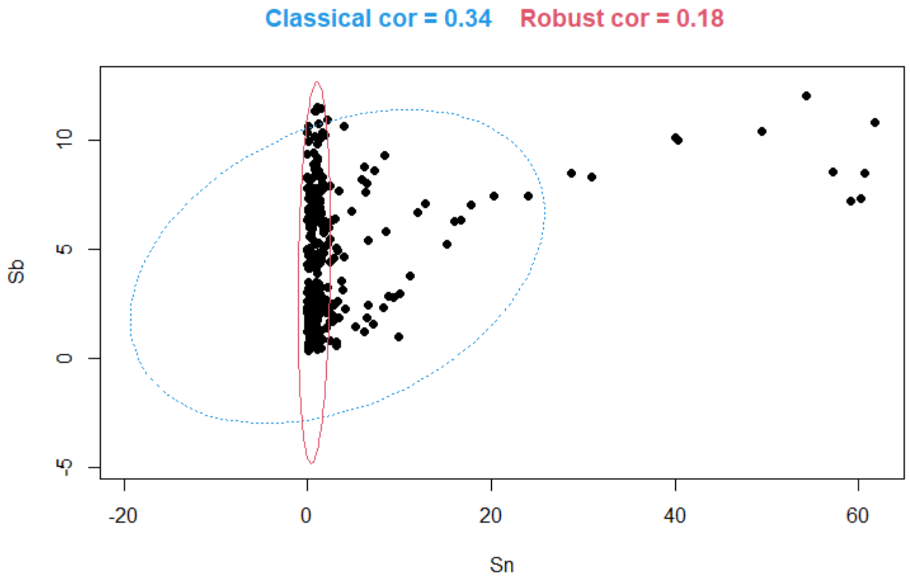

- **Pb vs Sb**

Whole cementum thickness:

Classical test	Pearson's product-moment correlation<br><br>data: Aa211C_CC$Sb and Aa211C_CC$Pb<br>t = 0.31289, df = 338, **p-value = 0.7546**<br>alternative hypothesis: true correlation is not equal to 0<br>95 percent confidence interval:<br>-0.08950772 0.12315580<br>sample estimates:<br>**cor**<br>**0.01701649**	Spearman's rank correlation rho<br><br>data: Aa211C_CC$Sb and Aa211C_CC$Pb<br>S = 5975776, **p-value = 0.1062**<br>alternative hypothesis: true rho is not equal to 0<br>sample estimates:<br>**rho**<br>**0.08775274**
MCD (α=0.05; quant=0.8)	$cor.rob<br>[1] **-0.05452402**	

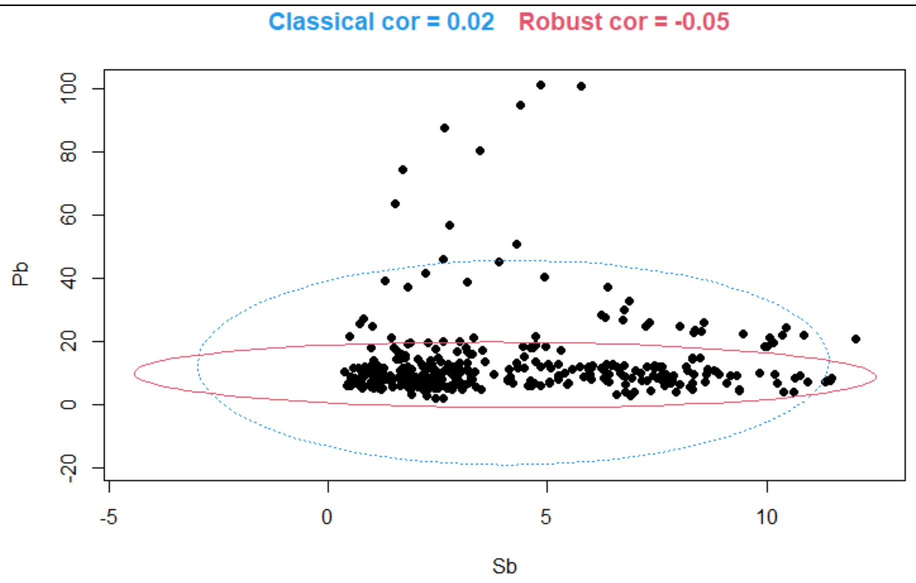

Removing 27 µm of Pb-enriched root surface:

Classical test	Pearson's product-moment correlation<br><br>data: Aa211C_CC_wRS$Sb and Aa211C_CC_wRS$Pb<br>t = 2.0427, df = 325, **p-value = 0.04189**<br>alternative hypothesis: true correlation is not equal to 0<br>95 percent confidence interval:<br>0.004181806 0.218381198<br>sample estimates:<br>**cor**<br>**0.1125893**	Spearman's rank correlation rho<br><br>data: Aa211C_CC_wRS$Sb and Aa211C_CC_wRS$Pb<br>S = 5227674, **p-value = 0.063**<br>alternative hypothesis: true rho is not equal to 0<br>sample estimates:<br>**rho**<br>**0.1029419**
MCD (α=0.05; quant=0.8)	$cor.rob<br>[1] **-0.04749927**	

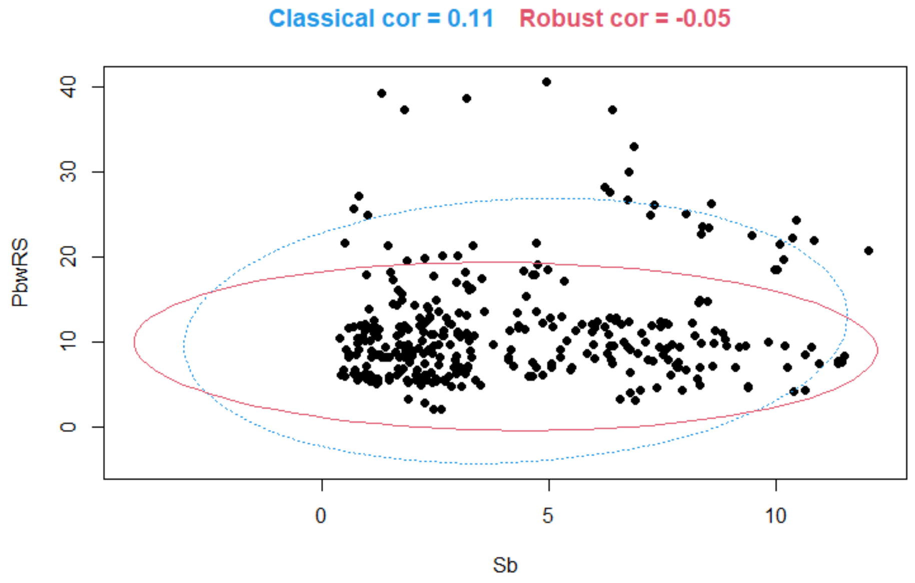

- **Pb vs Sn**

Whole cementum thickness:

Classical test	Pearson's product-moment correlation<br><br>data: Aa211C_CC$Sn and Aa211C_CC$Pb<br>t = 1.7675, df = 338, **p-value = 0.07805**<br>alternative hypothesis: true correlation is not equal to 0<br>95 percent confidence interval:<br>-0.01077634 0.20002186<br>sample estimates:<br>**cor**<br>**0.09569555**	Spearman's rank correlation rho<br><br>data: Aa211C_CC$Sn and Aa211C_CC$Pb<br>S = 5741980, **p-value = 0.02287**<br>alternative hypothesis: true rho is not equal to 0<br>sample estimates:<br>**rho**<br>**0.1234435**
MCD (α=0.05; quant=0.8)	$cor.rob<br>[1] **0.02039293**	

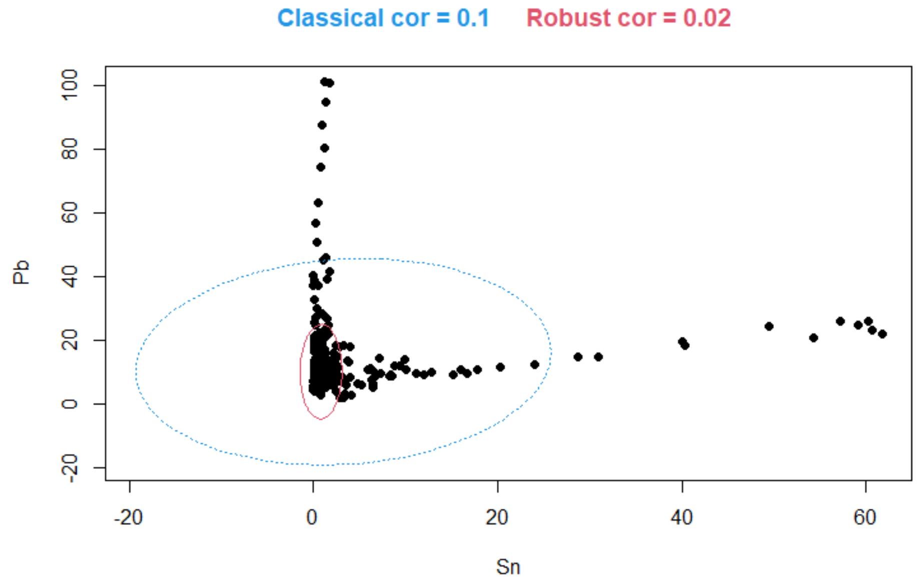

Removing 27 µm of Pb-enriched root surface:

Classical test	Pearson's product-moment correlation<br><br>data: Aa211C_CC_wRS$Sn and Aa211C_CC_wRS$Pb<br>t = 5.3054, df = 325, **p-value = 2.084e-07**<br>alternative hypothesis: true correlation is not equal to 0<br>95 percent confidence interval:<br>0.1793539 0.3791690<br>sample estimates:<br>**cor**<br>**0.2823206**	Spearman's rank correlation rho<br><br>data: Aa211C_CC_wRS$Sn and Aa211C_CC_wRS$Pb<br>S = 5124926, **p-value = 0.02931**<br>alternative hypothesis: true rho is not equal to 0<br>sample estimates:<br>**rho**<br>**0.1205733**
MCD (α=0.05; quant=0.8)	$cor.rob<br>[1] **0.05272294**	

Classical cor = 0.28   Robust cor = 0.05

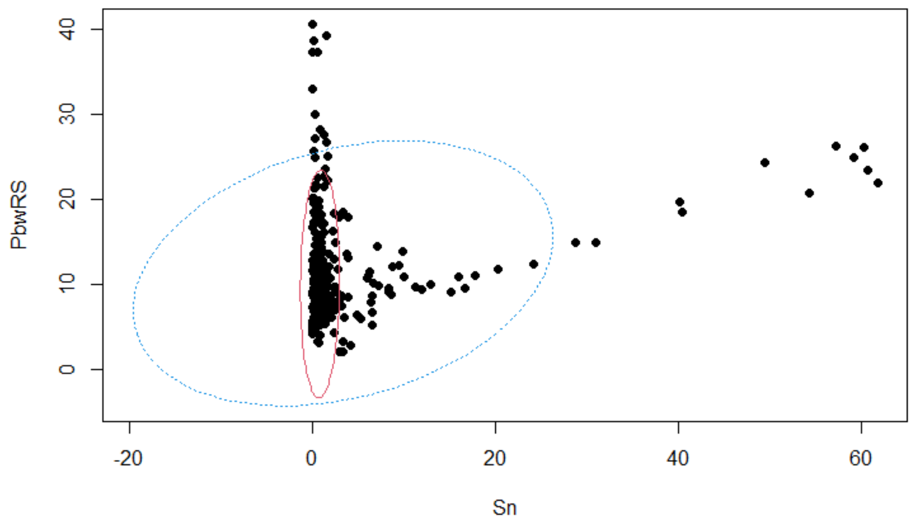

- **Br vs Ba**

Classical test	Pearson's product-moment correlation<br><br>data: Aa211C_CC$Ba and Aa211C_CC$Br<br>t = -2.2626, df = 338, **p-value = 0.0243**<br>alternative hypothesis: true correlation is not equal to 0<br>95 percent confidence interval:<br>-0.22557786 -0.01599186<br>sample estimates:<br>**cor**<br>**-0.1221463**	Spearman's rank correlation rho<br><br>data: Aa211C_CC$Ba and Aa211C_CC$Br<br>S = 7161164, **p-value = 0.08615**<br>alternative hypothesis: true rho is not equal to 0<br>sample estimates:<br>**rho**<br>**-0.09320567**
MCD (α=0.05; quant=0.8)	$cor.rob<br>[1] **0.0757424**	

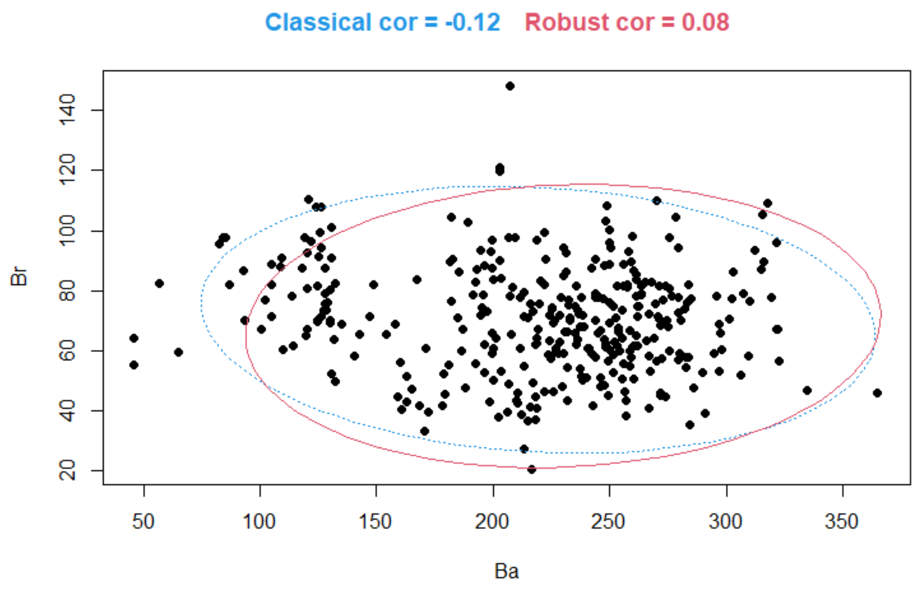

- **Br vs Sr**

Classical test	Pearson's product-moment correlation<br><br>data: Aa211C_CC$Sr and Aa211C_CC$Br<br>t = -1.3668, df = 338, **p-value = 0.1726**<br>alternative hypothesis: true correlation is not equal to 0<br>95 percent confidence interval:<br>-0.17908762 0.03248073<br>sample estimates:<br>**cor**<br>**-0.0741376**	Spearman's rank correlation rho<br><br>data: Aa211C_CC$Sr and Aa211C_CC$Br<br>S = 7018806, **p-value = 0.1885**<br>alternative hypothesis: true rho is not equal to 0<br>sample estimates:<br>**rho**<br>**-0.07147365**
MCD (α=0.05; quant=0.8)	$cor.rob<br>[1] **-0.02307787**	

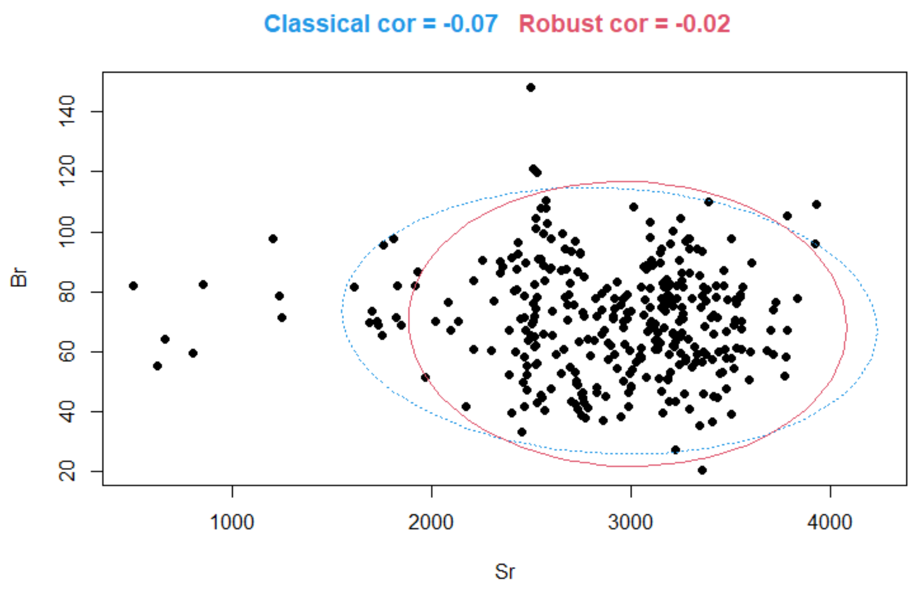

- **Br vs Zn**

Classical test	Pearson's product-moment correlation<br><br>data: Aa211C_CC$Zn and Aa211C_CC$Br<br>t = 0.19553, df = 338, **p-value = 0.8451**<br>alternative hypothesis: true correlation is not equal to 0<br>95 percent confidence interval:<br>-0.09583573 0.11686490<br>sample estimates:<br>**cor**<br>**0.01063488**	Spearman's rank correlation rho<br><br>data: Aa211C_CC$Zn and Aa211C_CC$Br<br>S = 6435608, **p-value = 0.7469**<br>alternative hypothesis: true rho is not equal to 0<br>sample estimates:<br>**rho**<br>**0.01755592**
MCD (α=0.05; quant=0.8)	$cor.rob<br>[1] **0.03940319**	

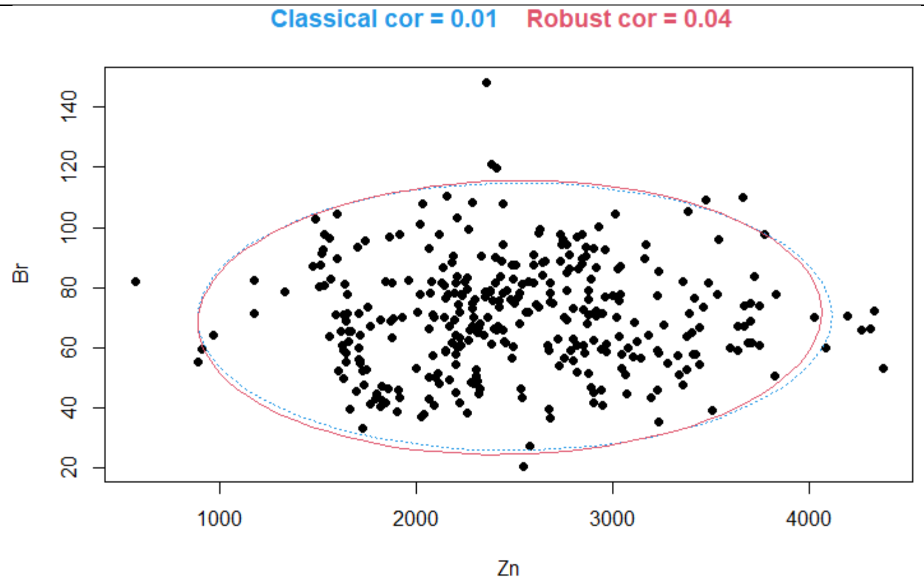

- **Br vs Ca**

Classical test	Pearson's product-moment correlation<br><br>data: Aa211C_CC$Ca and Aa211C_CC$Br<br>t = -1.3833, df = 338, **p-value = 0.1675**<br>alternative hypothesis: true correlation is not equal to 0<br>95 percent confidence interval:<br>-0.17995719 0.03158313<br>sample estimates:<br>**cor**<br>**-0.07503112**	Spearman's rank correlation rho<br><br>data: Aa211C_CC$Ca and Aa211C_CC$Br<br>S = 6896554, **p-value = 0.3314**<br>alternative hypothesis: true rho is not equal to 0<br>sample estimates:<br>**rho**<br>**-0.05281096**
MCD (α=0.05; quant=0.8)	$cor.rob<br>[1] **-0.02307787**	

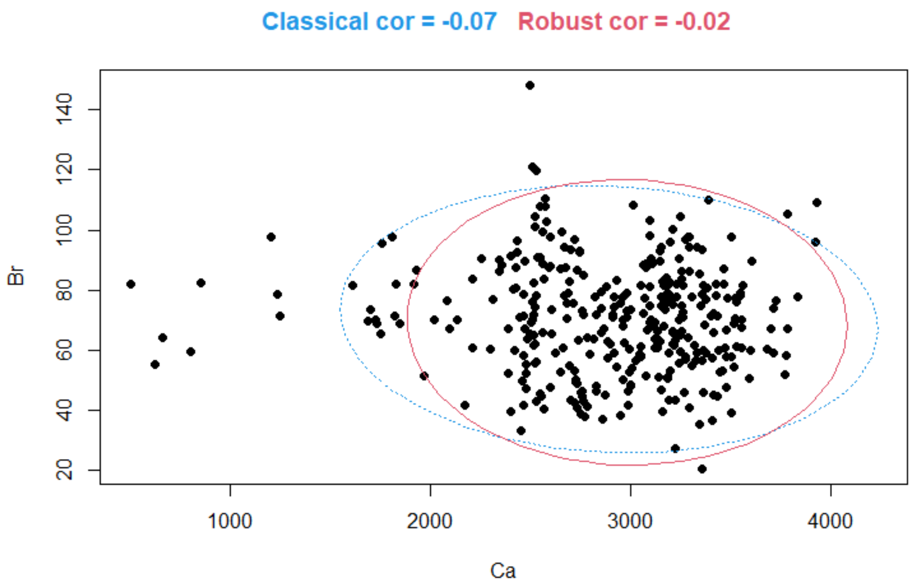
Classical cor = -0.07   Robust cor = -0.02
Br
Ca
20
40
60
80
100
120
140
1000
2000
3000
4000

Odense 533M1 – Acellular Cementum (total thickness=122 µm)

Correlations between pairs of elements

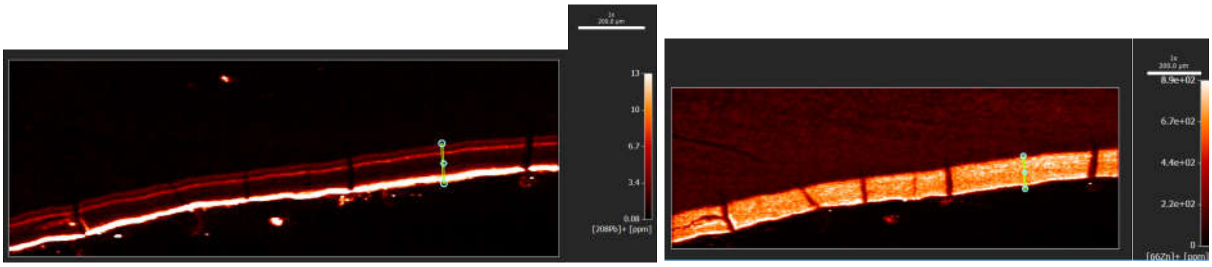

Pb (left) and Zn (right) maps (green path indicates where the measurements are taken)

Summary: whole cementum thickness, Spearman correlations.

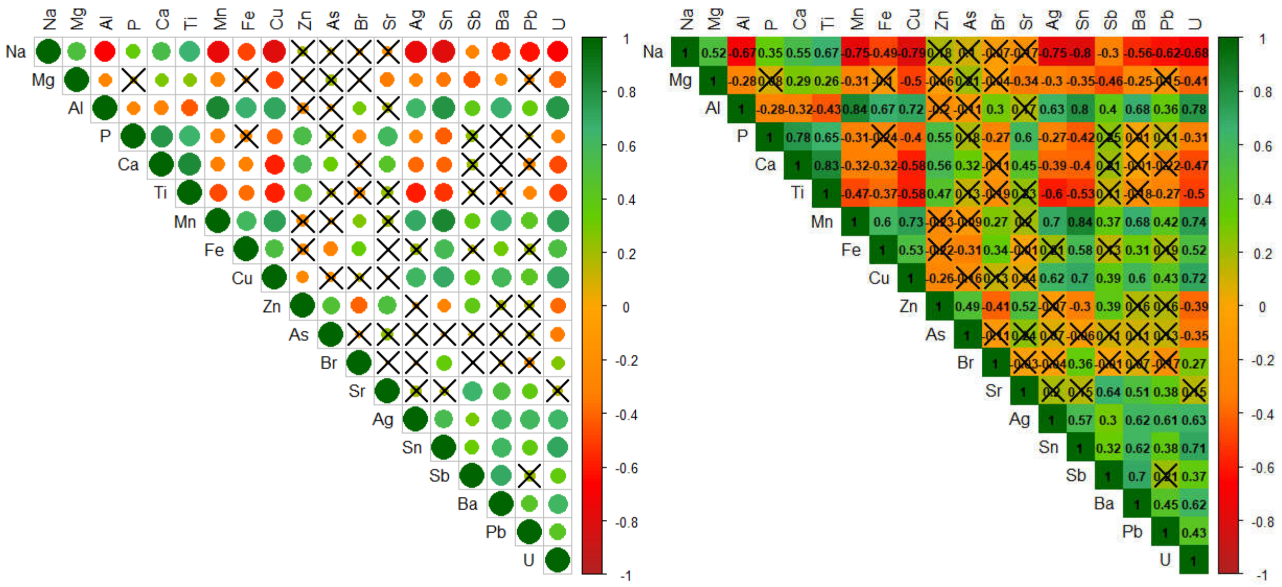

Summary: removing the first 25 µm of root surface, Spearman correlations.

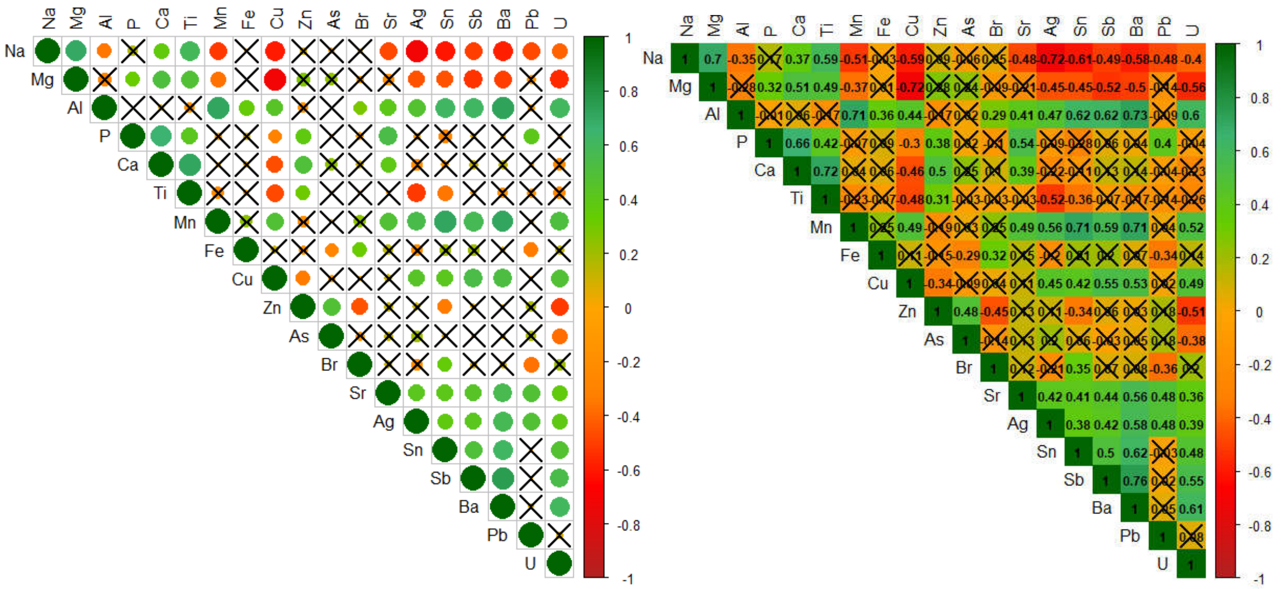

wRS: without Root surface [Pb = ~25 µm; Fe = ~20 µm; Mn = ~25 µm; Al = ~18 µm]

- **Ca vs Ba**

<table>
<tr><td rowspan="1">Classical test</td><td>Pearson's product-moment correlation

data: Od533M_AC$Ca and Od533M_AC$Ba
t = -1.3231, df = 60, p-value = 0.1908
alternative hypothesis: true correlation is not equal to 0
95 percent confidence interval:
-0.40126689 0.08496775
sample estimates:
cor
-0.1683737</td><td>Spearman's rank correlation rho

data: Od533M_AC$Ca and Od533M_AC$Ba
S = 40122, p-value = 0.9363
alternative hypothesis: true rho is not equal to 0
sample estimates:
rho
-0.01034978</td></tr>
<tr><td>MCD (α=0.05; quant=0.8)</td><td colspan="2">$cor.rob
[1] 0.1322351</td></tr>
</table>

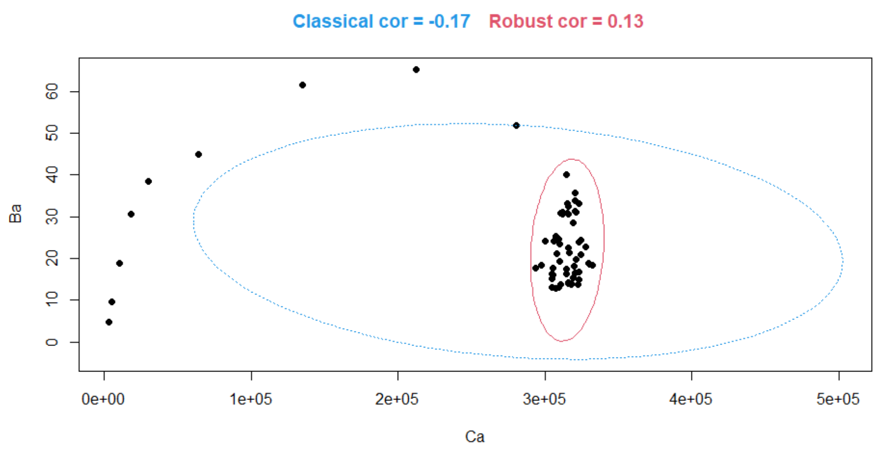

- **Ca vs Sr**

<table>
<tr><td>Classical test</td><td>Pearson's product-moment correlation

data: Od533M_AC$Ca and Od533M_AC$Sr
t = 10.43, df = 60, p-value = 4.231e-15
alternative hypothesis: true correlation is not equal to 0
95 percent confidence interval:
0.6917803 0.8767852
sample estimates:
cor
0.8028315</td><td>Spearman's rank correlation rho

data: Od533M_AC$Ca and Od533M_AC$Sr
S = 21722, p-value = 0.0002536
alternative hypothesis: true rho is not equal to 0
sample estimates:
rho
0.4529979</td></tr>
<tr><td>MCD (α=0.05; quant=0.8)</td><td colspan="2">$cor.rob
[1] 0.3525441</td></tr>
</table>

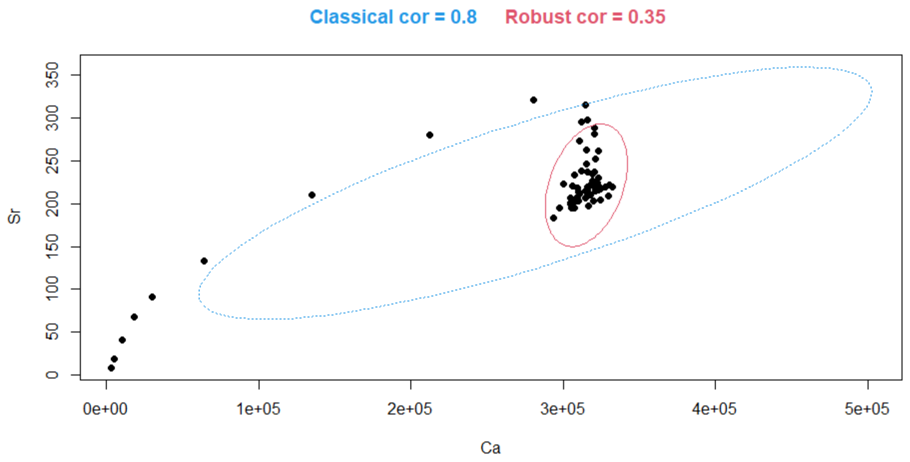

- **Ba vs Sr**

Classical test	Pearson's product-moment correlation<br><br>data: Od533M_AC$Ba and Od533M_AC$Sr<br>t = 3.2487, df = 60, **p-value = 0.001901**<br>alternative hypothesis: true correlation is not equal to 0<br>95 percent confidence interval:<br>0.1516530 0.5804646<br>sample estimates:<br>**cor**<br>**0.3867711**	Spearman's rank correlation rho<br><br>data: Od533M_AC$Ba and Od533M_AC$Sr<br>S = 19484, **p-value = 3.066e-05**<br>alternative hypothesis: true rho is not equal to 0<br>sample estimates:<br>**rho**<br>**0.5093551**
MCD (α=0.05; quant=0.8)	$cor.rob<br>[1] **0.773796**	

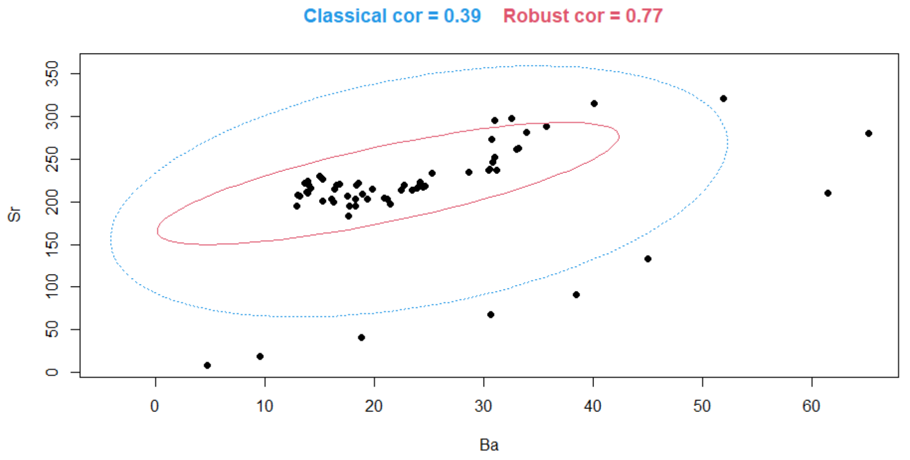

- **Zn vs Sr**

Classical test	Pearson's product-moment correlation<br><br>data: Od533M_AC$Zn and Od533M_AC$Sr<br>t = 12.962, df = 60, **p-value < 2.2e-16**<br>alternative hypothesis: true correlation is not equal to 0<br>95 percent confidence interval:<br>0.7747542 0.9125301<br>sample estimates:<br>**cor**<br>**0.858412**	Spearman's rank correlation rho<br><br>data: Od533M_AC$Zn and Od533M_AC$Sr<br>S = 19190, **p-value = 2.263e-05**<br>alternative hypothesis: true rho is not equal to 0<br>sample estimates:<br>**rho**<br>**0.5167586**
MCD (α=0.05; quant=0.8)	$cor.rob<br>[1] **0.1622059**	

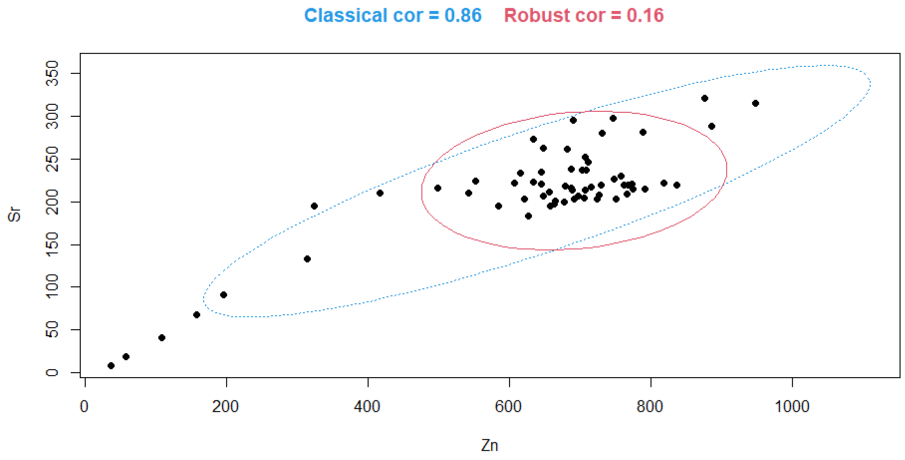

- **Mg vs Sr**

Classical test	Pearson's product-moment correlation<br><br>data: Od533M_AC$Sr and Od533M_AC$Mg<br>t = -0.74167, df = 60, **p-value = 0.4612**<br>alternative hypothesis: true correlation is not equal to 0<br>95 percent confidence interval:<br>-0.3370577 0.1582213<br>sample estimates:<br>**cor**<br>**-0.09531356**	Spearman's rank correlation rho<br><br>data: Od533M_AC$Sr and Od533M_AC$Mg<br>S = 53034, **p-value = 0.00795**<br>alternative hypothesis: true rho is not equal to 0<br>sample estimates:<br>**rho**<br>**0**
MCD (α=0.05; quant=0.8)	$cor.rob<br>[1] **-0.5588916**	

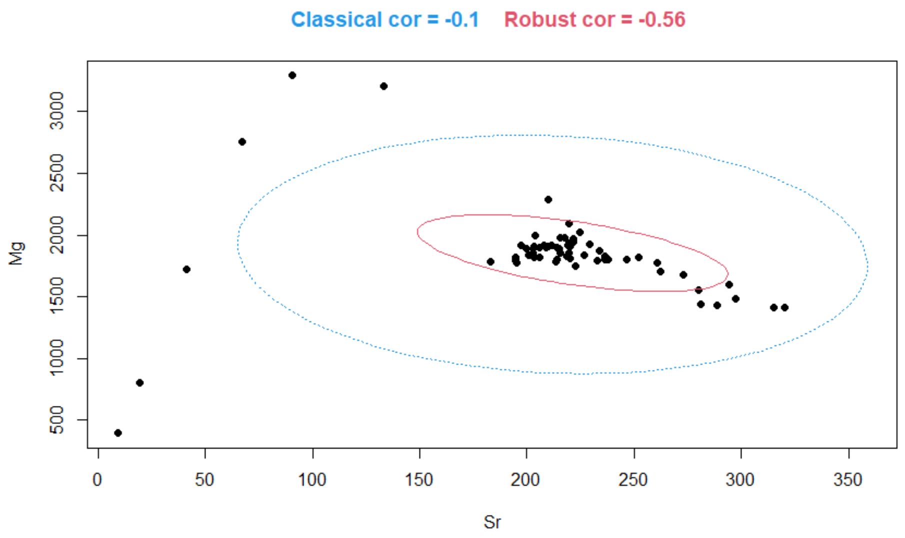

- **Pb vs Sr**

Whole cementum thickness:

Classical test	Pearson's product-moment correlation<br><br>data: Od533M_AC$Sr and Od533M_AC$Pb<br>t = 2.1467, df = 60, **p-value = 0.03587**<br>alternative hypothesis: true correlation is not equal to 0<br>95 percent confidence interval:<br>0.0185453 0.4845234<br>sample estimates:<br>**cor**<br>**0.2670764**	Spearman's rank correlation rho<br><br>data: Od533M_AC$Sr and Od533M_AC$Pb<br>S = 24506, **p-value = 0.002278**<br>alternative hypothesis: true rho is not equal to 0<br>sample estimates:<br>rho<br>0.3828914
MCD (α=0.05; quant=0.8)	$cor.rob<br>[1] **0.6354589**	

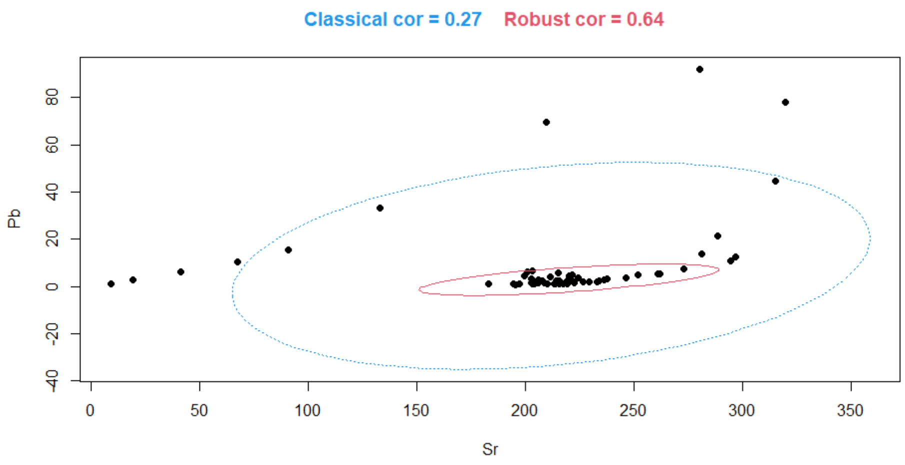

Removing 25 µm of Pb-enriched root surface:

Classical test	Pearson's product-moment correlation<br><br>data: Od533M_AC_wRS$Sr and Od533M_AC_wRS$Pb<br>t = 5.6421, df = 47, **p-value = 9.329e-07**<br>alternative hypothesis: true correlation is not equal to 0<br>95 percent confidence interval:<br>0.4313376 0.7776908<br>sample estimates:<br>**cor**<br>**0.6354589**	Spearman's rank correlation rho<br><br>data: Od533M_AC_wRS$Zn and Od533M_AC_wRS$Pb<br>S = 16154, **p-value = 0.2262**<br>alternative hypothesis: true rho is not equal to 0<br>sample estimates:<br>**rho**<br>**0.1758163**
MCD (α=0.05; quant=0.8)	$cor.rob<br>[1] **0.5718473**	

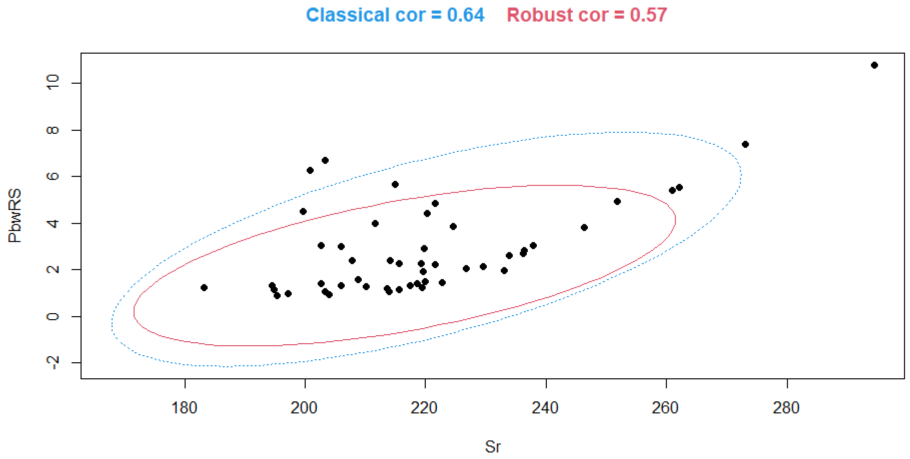

- **Sr vs Zn**

Classical test	Pearson's product-moment correlation<br><br>data: Od533M_AC$Zn and Od533M_AC$Sr<br>t = 12.962, df = 60, **p-value < 2.2e-16**<br>alternative hypothesis: true correlation is not equal to 0<br>95 percent confidence interval:<br>0.7747542 0.9125301<br>sample estimates:<br>**cor**<br>**0.858412**	Spearman's rank correlation rho<br><br>data: Od533M_AC$Zn and Od533M_AC$Sr<br>S = 19190, **p-value = 2.263e-05**<br>alternative hypothesis: true rho is not equal to 0<br>sample estimates:<br>**rho**<br>**0.5167586**
MCD (α=0.05; quant=0.8)	$cor.rob<br>[1] **0.1622059**	

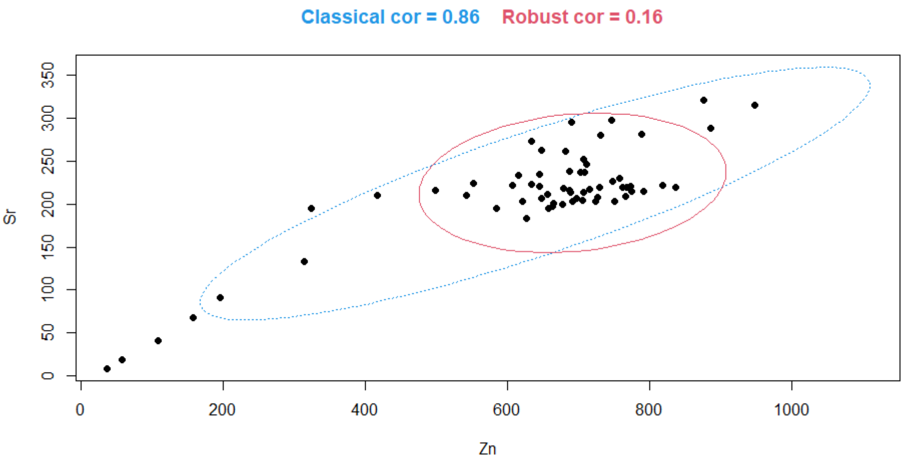

- **Pb vs Zn**

Whole cementum thickness:

Classical test	Pearson's product-moment correlation<br><br>data: Od533M_AC$Zn and Od533M_AC$Pb<br>t = 0.78748, df = 60, **p-value = 0.4341**<br>alternative hypothesis: true correlation is not equal to 0<br>95 percent confidence interval:<br>-0.1524783 0.3422638<br>sample estimates:<br>**cor**<br>**0.1011418**	Spearman's rank correlation rho<br><br>data: Od533M_AC$Zn and Od533M_AC$Pb<br>S = 33264, **p-value = 0.2069**<br>alternative hypothesis: true rho is not equal to 0<br>sample estimates:<br>**rho**<br>**0.162348**
MCD (α=0.05; quant=0.8)	$cor.rob<br>[1] **0.1101698**	

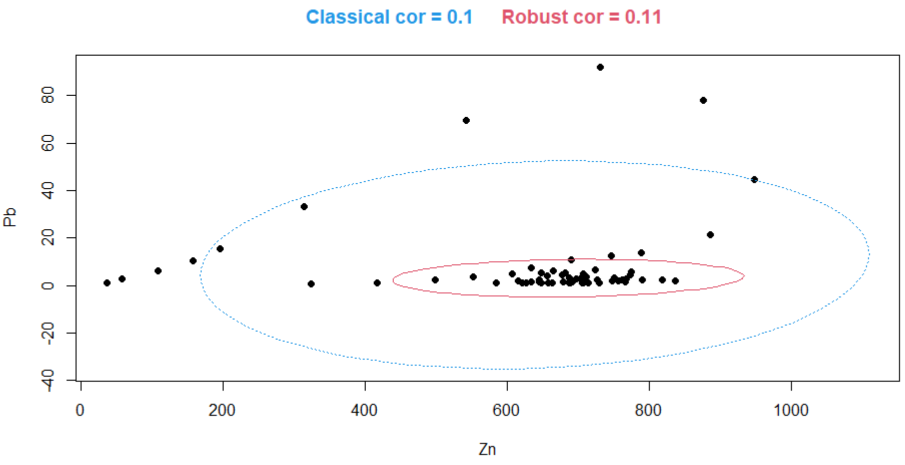

Removing 25 µm of Pb-enriched root surface:

Classical test	Pearson's product-moment correlation<br><br>data: Od533M_AC_wRS$Zn and Od533M_AC_wRS$Pb<br>t = 0.8646, df = 47, **p-value = 0.3916**<br>alternative hypothesis: true correlation is not equal to 0<br>95 percent confidence interval:<br>-0.1617640 0.3925105<br>sample estimates:<br>**cor**<br>**0.1251242**	Spearman's rank correlation rho<br><br>data: Od533M_AC_wRS$Zn and Od533M_AC_wRS$Pb<br>S = 16154, **p-value = 0.2262**<br>alternative hypothesis: true rho is not equal to 0<br>sample estimates:<br>**rho**<br>**0.1758163**
MCD (α=0.05; quant=0.8)	$cor.rob<br>[1] **0.03672209**	

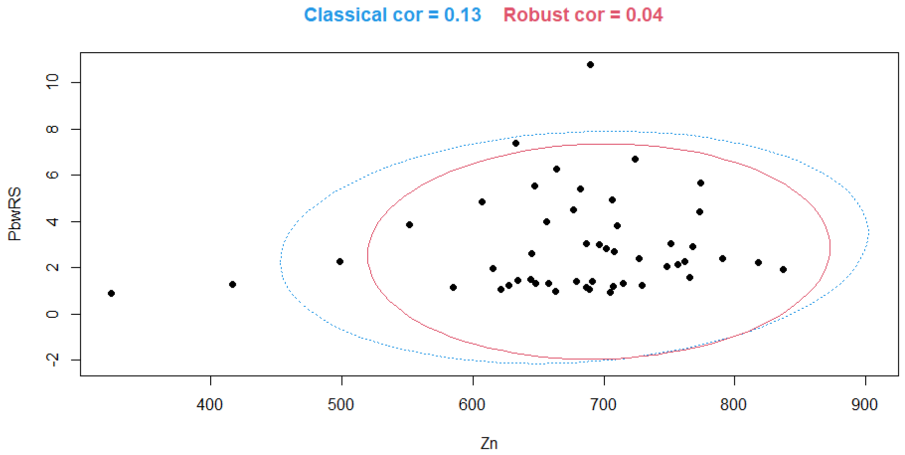

- **Zn vs Ca**

Classical test	Pearson's product-moment correlation<br><br>data: Od533M_AC$Zn and Od533M_AC$Ca<br>t = 12.292, df = 60, **p-value < 2.2e-16**<br>alternative hypothesis: true correlation is not equal to 0<br>95 percent confidence interval:<br>0.7560046 0.9046328<br>sample estimates:<br>**cor**<br>**0.846022**	Spearman's rank correlation rho<br><br>data: Od533M_AC$Zn and Od533M_AC$Ca<br>S = 17394, **p-value = 3.074e-06**<br>alternative hypothesis: true rho is not equal to 0<br>sample estimates:<br>**rho**<br>**0.5619853**
MCD (α=0.05; quant=0.8)	$cor.rob<br>[1] **0.495149**	

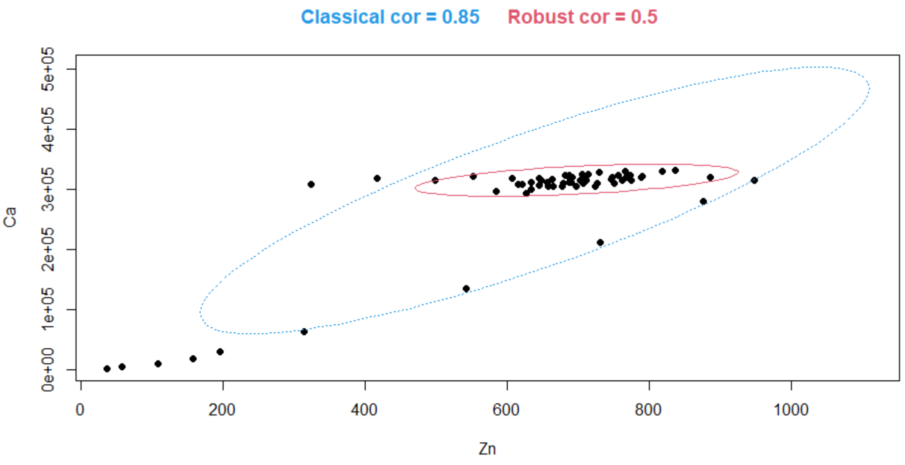

- **Zn vs Fe**

Whole cementum thickness:

	Pearson's product-moment correlation	Spearman's rank correlation rho
Classical test	data: Od533M_AC$Zn and Od533M_AC$Fe<br>t = -5.7009, df = 60, **p-value = 3.858e-07**<br>alternative hypothesis: true correlation is not equal to 0<br>95 percent confidence interval:<br>-0.7338670 -0.4025811<br>sample estimates:<br>**cor**<br>**-0.5927474**	data: Od533M_AC$Zn and Od533M_AC$Fe<br>S = 48500, **p-value = 0.08392**<br>alternative hypothesis: true rho is not equal to 0<br>sample estimates:<br>**rho**<br>**-0.2213241**
MCD (α=0.05; quant=0.8)	$cor.rob<br>[1] **0.05539145**	

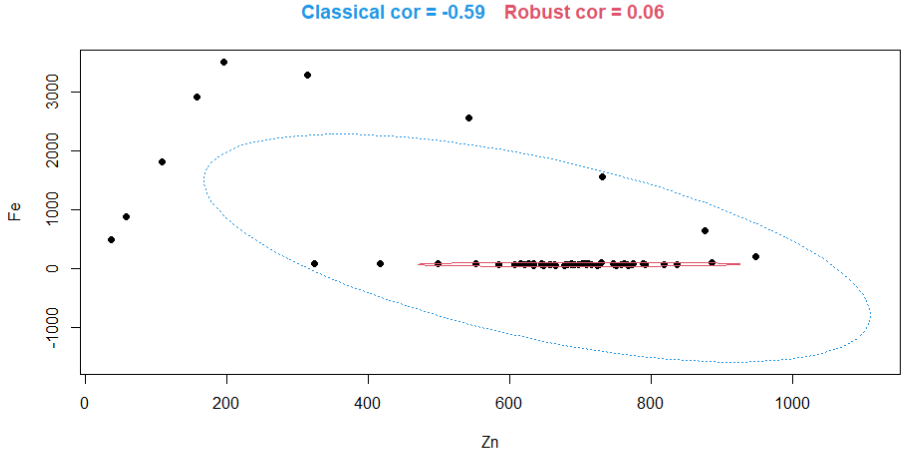

Removing 20 µm of Fe-enriched root surface:

Classical test	Pearson's product-moment correlation<br><br>data: Od533M_AC$Zn and Od533M_AC$Fe<br>t = -5.7009, df = 60, **p-value = 3.858e-07**<br>alternative hypothesis: true correlation is not equal to 0<br>95 percent confidence interval:<br>-0.7338670 -0.4025811<br>sample estimates:<br>**cor**<br>**-0.5927474**	Spearman's rank correlation rho<br><br>data: Od533M_AC$Zn and Od533M_AC$Fe<br>S = 48500, **p-value = 0.08392**<br>alternative hypothesis: true rho is not equal to 0<br>sample estimates:<br>**rho**<br>**-0.2213241**
MCD (α=0.05; quant=0.8)	$cor.rob<br>[1] **0.05539145**	

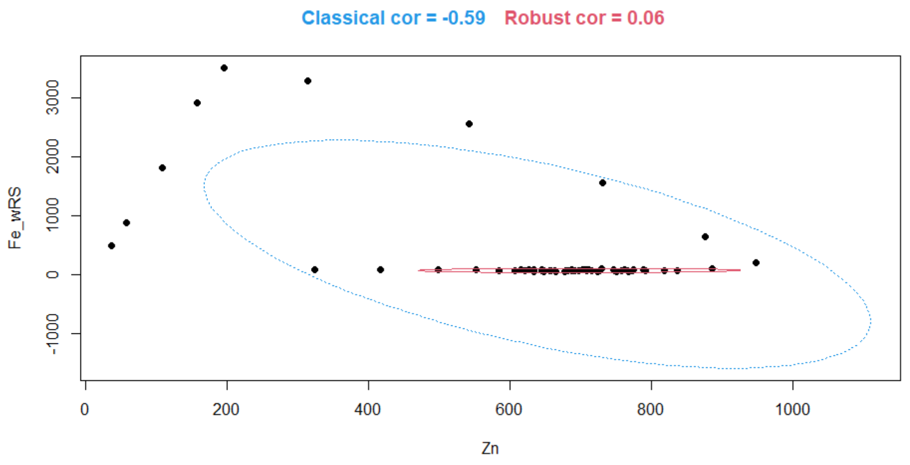

- **Zn vs Cu**

Classical test	Pearson's product-moment correlation<br><br>data: Od533M_AC$Zn and Od533M_AC$Cu<br>t = -2.3242, df = 60, **p-value = 0.02352**<br>alternative hypothesis: true correlation is not equal to 0<br>95 percent confidence interval:<br>-0.5011898 -0.0405404<br>sample estimates:<br>**cor**<br>**-0.2873985**	Spearman's rank correlation rho<br><br>data: Od533M_AC$Zn and Od533M_AC$Cu<br>S = 50118, **p-value = 0.03992**<br>alternative hypothesis: true rho is not equal to 0<br>sample estimates:<br>**rho**<br>**-0.2620684**
MCD (α=0.05; quant=0.8)	$cor.rob<br>[1] **-0.199509**	

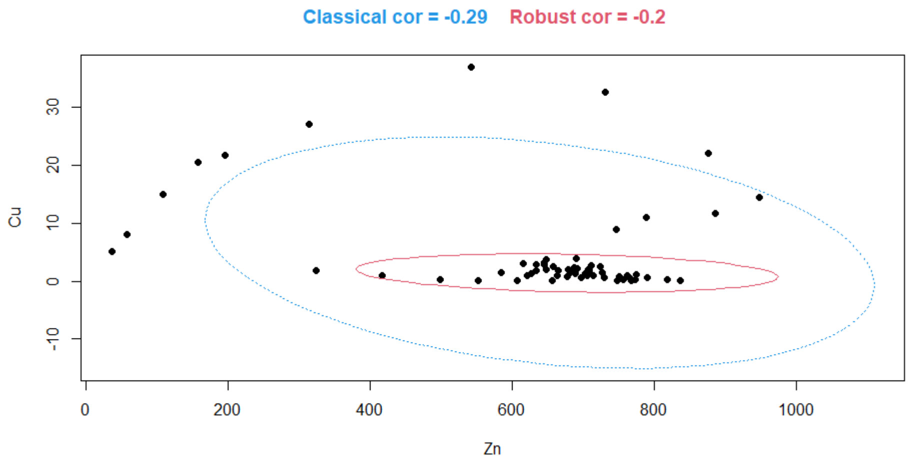

- **Zn vs Mg**

Classical test	Pearson's product-moment correlation<br><br>data: Od533M_AC$Zn and Od533M_AC$Mg<br>t = -0.67332, df = 60, **p-value = 0.5033**<br>alternative hypothesis: true correlation is not equal to 0<br>95 percent confidence interval:<br>-0.3292453 0.1667769<br>sample estimates:<br>**cor**<br>**-0.08659857**	Spearman's rank correlation rho<br><br>data: Od533M_AC$Zn and Od533M_AC$Mg<br>S = 42232, **p-value = 0.6232**<br>alternative hypothesis: true rho is not equal to 0<br>sample estimates:<br>**rho**<br>**-0.06348367**
MCD (α=0.05; quant=0.8)	$cor.rob<br>[1] **-0.08098037**	

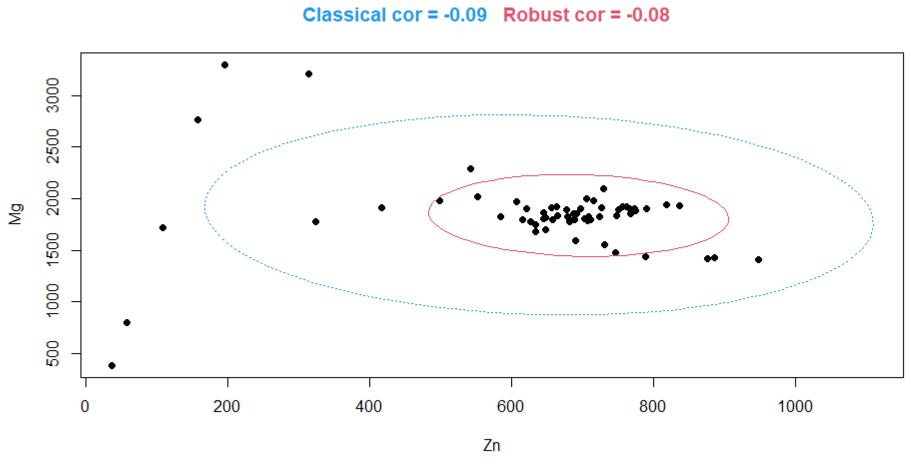

- **Ca vs Fe**

Whole cementum thickness:

Classical test	Pearson's product-moment correlation<br><br>data: Od533M_AC$Ca and Od533M_AC$Fe<br>t = -11.254, df = 60, **p-value < 2.2e-16**<br>alternative hypothesis: true correlation is not equal to 0<br>95 percent confidence interval:<br>-0.8903300 -0.7226572<br>sample estimates:<br>**cor**<br>**-0.8237425**	Spearman's rank correlation rho<br><br>data: Od533M_AC$Ca and Od533M_AC$Fe<br>S = 52262, **p-value = 0.01264**<br>alternative hypothesis: true rho is not equal to 0<br>sample estimates:<br>**rho**<br>**-0.3160585**
MCD (α=0.05; quant=0.8)	$cor.rob<br>[1] **0.1122484**	

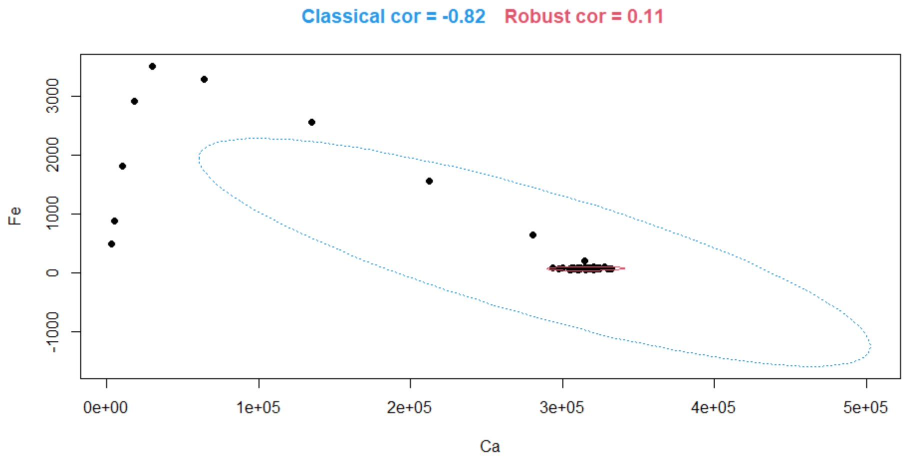

Removing 20 µm of Fe-enriched root surface:

Classical test	Pearson's product-moment correlation<br><br>data: Od533M_AC$Ca and Od533M_AC$Fe<br>t = -11.254, df = 60, **p-value < 2.2e-16**<br>alternative hypothesis: true correlation is not equal to 0<br>95 percent confidence interval:<br>-0.8903300 -0.7226572<br>sample estimates:<br>**cor**<br>**-0.8237425**	Spearman's rank correlation rho<br><br>data: Od533M_AC$Ca and Od533M_AC$Fe<br>S = 52262, **p-value = 0.01264**<br>alternative hypothesis: true rho is not equal to 0<br>sample estimates:<br>**rho**<br>**-0.3160585**
MCD (α=0.05; quant=0.8)	$cor.rob<br>[1] **0.1122484**	

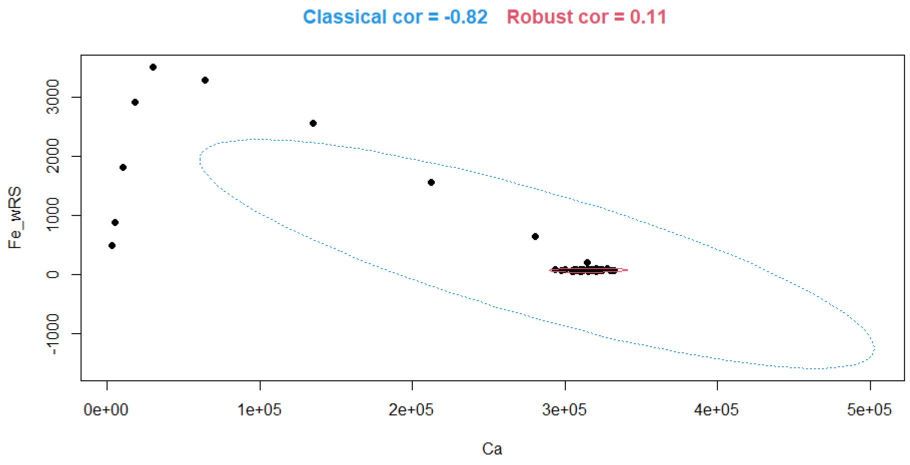

- **Ca vs Mg**

Classical test	Pearson's product-moment correlation<br><br>data: Od533M_AC$Ca and Od533M_AC$Mg<br>t = -0.84899, df = 60, **p-value = 0.3993**<br>alternative hypothesis: true correlation is not equal to 0<br>95 percent confidence interval:<br>-0.3492168 0.1447556<br>sample estimates:<br>**cor**<br>**-0.1089518**	Spearman's rank correlation rho<br><br>data: Od533M_AC$Ca and Od533M_AC$Mg<br>S = 28360, **p-value = 0.02465**<br>alternative hypothesis: true rho is not equal to 0<br>sample estimates:<br>**rho**<br>**0.2858402**
MCD (α=0.05; quant=0.8)	$cor.rob<br>[1] **0.4769753**	

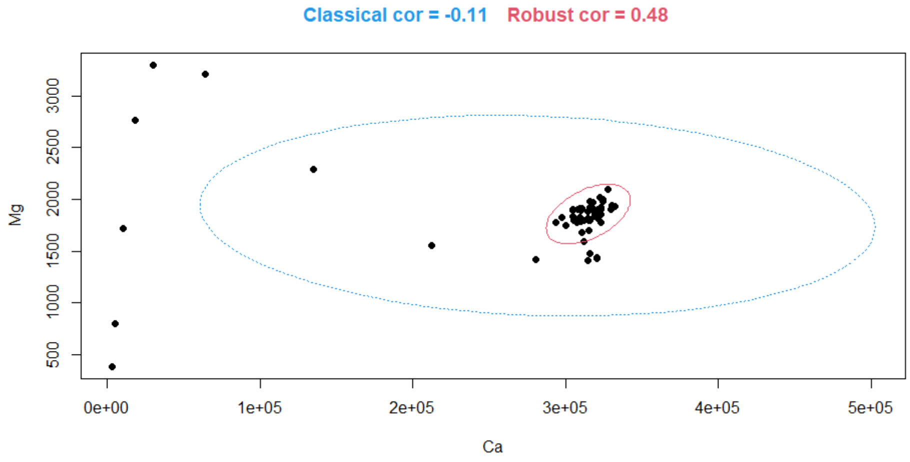

- **Ca vs P**

Classical test	Pearson's product-moment correlation<br><br>data: Od533M_AC$Ca and Od533M_AC$P<br>t = 119.66, df = 60, **p-value < 2.2e-16**<br>alternative hypothesis: true correlation is not equal to 0<br>95 percent confidence interval:<br>0.9965231 0.9987457<br>sample estimates:<br>**cor**<br>**0.9979114**	Spearman's rank correlation rho<br><br>data: Od533M_AC$Ca and Od533M_AC$P<br>S = 8874, **p-value < 2.2e-16**<br>alternative hypothesis: true rho is not equal to 0<br>sample estimates:<br>rho<br>**0.7765355**
MCD (α=0.05; quant=0.8)	$cor.rob<br>[1] **0.6936723**	

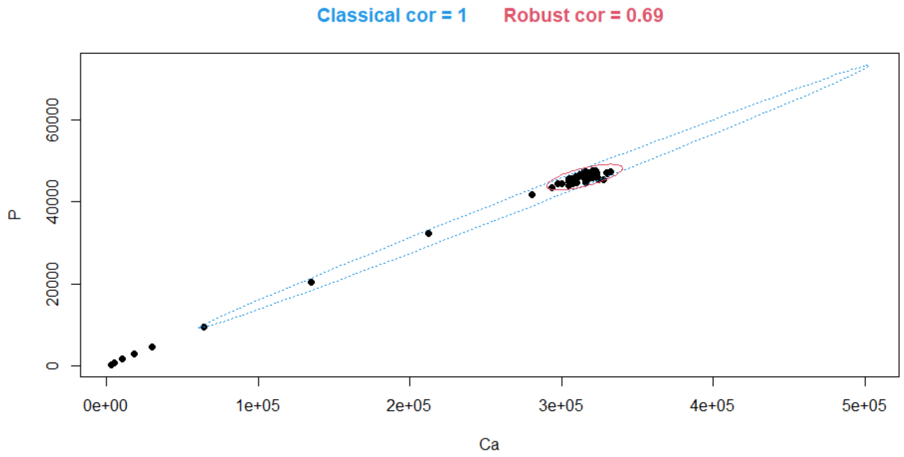

- **Cu vs Fe**

Whole cementum thickness:

Classical test	Pearson's product-moment correlation<br><br>data: Od533M_AC$Cu and Od533M_AC$Fe<br>t = 11.496, df = 60, **p-value < 2.2e-16**<br>alternative hypothesis: true correlation is not equal to 0<br>95 percent confidence interval:<br>0.7309596 0.8939221<br>sample estimates:<br>**cor**<br>**0.8293187**	Spearman's rank correlation rho<br><br>data: Od533M_AC$Cu and Od533M_AC$Fe<br>S = 18542, **p-value = 1.133e-05**<br>alternative hypothesis: true rho is not equal to 0<br>sample estimates:<br>**rho**<br>**0.5330765**
MCD (α=0.05; quant=0.8)	$cor.rob<br>[1] **0.05517098**	

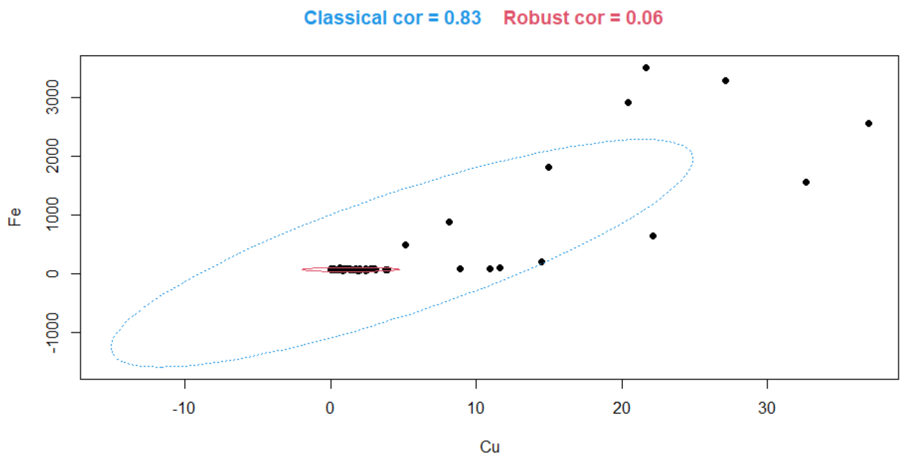

Removing 15 µm of Fe-enriched root surface:

Classical test	Pearson's product-moment correlation<br><br>data: Od533M_AC$Cu and Od533M_AC$Fe<br>t = 11.496, df = 60, **p-value < 2.2e-16**<br>alternative hypothesis: true correlation is not equal to 0<br>95 percent confidence interval:<br>0.7309596 0.8939221<br>sample estimates:<br>**cor**<br>**0.8293187**	Spearman's rank correlation rho<br><br>data: Od533M_AC$Cu and Od533M_AC$Fe<br>S = 18542, **p-value = 1.133e-05**<br>alternative hypothesis: true rho is not equal to 0<br>sample estimates:<br>**rho**<br>**0.5330765**
MCD (α=0.05; quant=0.8)	$cor.rob<br>[1] **0.05517098**	

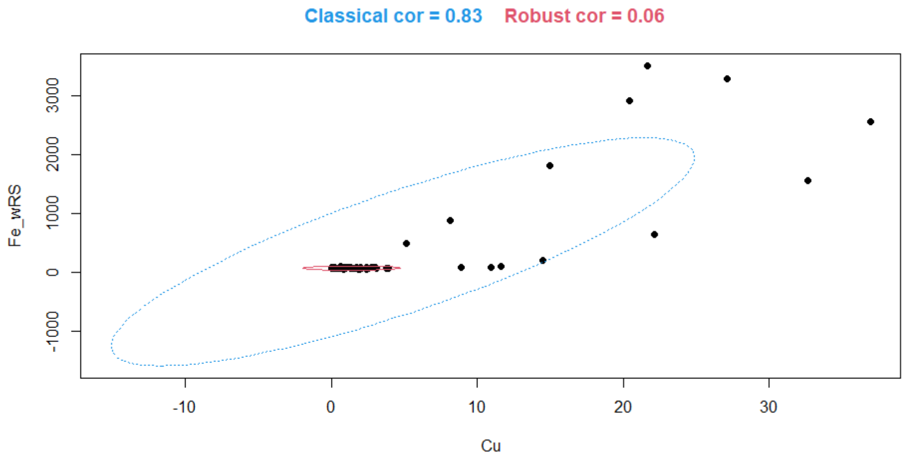

- **Mg vs Fe**

Whole cementum thickness:

Classical test	Pearson's product-moment correlation<br><br>data: Od533M_AC$Mg and Od533M_AC$Fe<br>t = 5.2596, df = 60, **p-value = 2.03e-06**<br>alternative hypothesis: true correlation is not equal to 0<br>95 percent confidence interval:<br>0.3628988 0.7116660<br>sample estimates:<br>**cor**<br>**0.5617497**	Spearman's rank correlation rho<br><br>data: Od533M_AC$Mg and Od533M_AC$Fe<br>S = 43784, **p-value = 0.4266**<br>alternative hypothesis: true rho is not equal to 0<br>sample estimates:<br>**rho**<br>**-0.102566**
MCD (α=0.05; quant=0.8)	$cor.rob<br>[1] **0.1951158**	

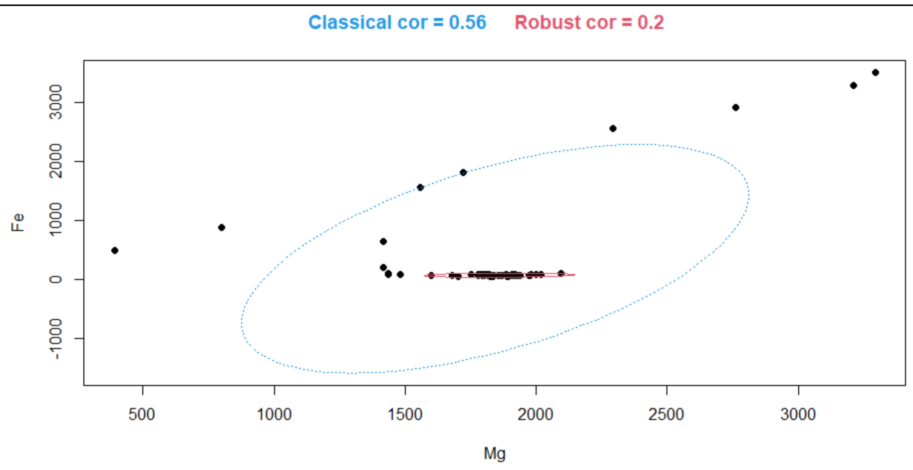

Removing 20 µm of Fe-enriched root surface:

Classical test	Pearson's product-moment correlation<br><br>data: Od533M_AC$Mg and Od533M_AC$Fe<br>t = 5.2596, df = 60, **p-value = 2.03e-06**<br>alternative hypothesis: true correlation is not equal to 0<br>95 percent confidence interval:<br>0.3628988 0.7116660<br>sample estimates:<br>**cor**<br>**0.5617497**	Spearman's rank correlation rho<br><br>data: Od533M_AC$Mg and Od533M_AC$Fe<br>S = 43784, **p-value = 0.4266**<br>alternative hypothesis: true rho is not equal to 0<br>sample estimates:<br>**rho**<br>**-0.102566**
MCD (α=0.05; quant=0.8)	$cor.rob<br>[1] **0.1951158**	

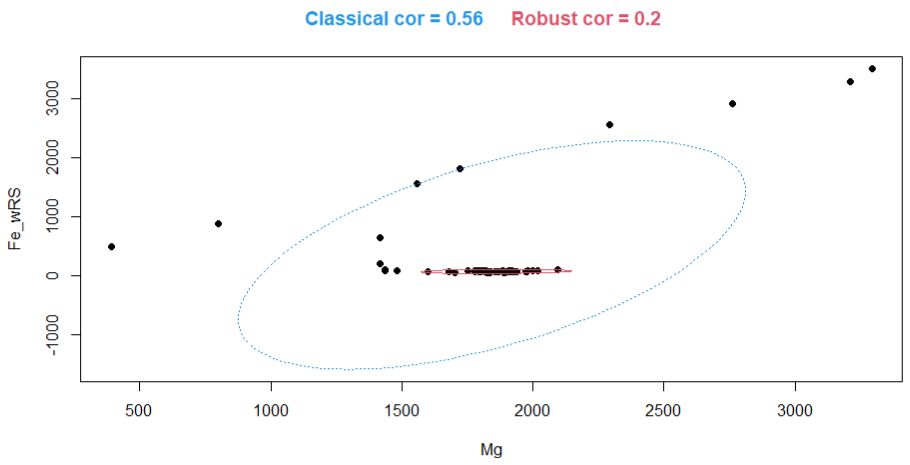

- **Cu vs Mg**

Classical test	Pearson's product-moment correlation<br><br>data: Od533M_AC$Mg and Od533M_AC$Cu<br>t = 1.8879, df = 60, **p-value = 0.06388**<br>alternative hypothesis: true correlation is not equal to 0<br>95 percent confidence interval:<br>-0.01379117 0.45939099<br>sample estimates:<br>**cor**<br>**0.2367927**	Spearman's rank correlation rho<br><br>data: Od533M_AC$Mg and Od533M_AC$Cu<br>S = 59714, **p-value = 3.847e-05**<br>alternative hypothesis: true rho is not equal to 0<br>sample estimates:<br>**rho**<br>**-0.5037143**
MCD (α=0.05; quant=0.8)	$cor.rob<br>[1] **-0.6742311**	

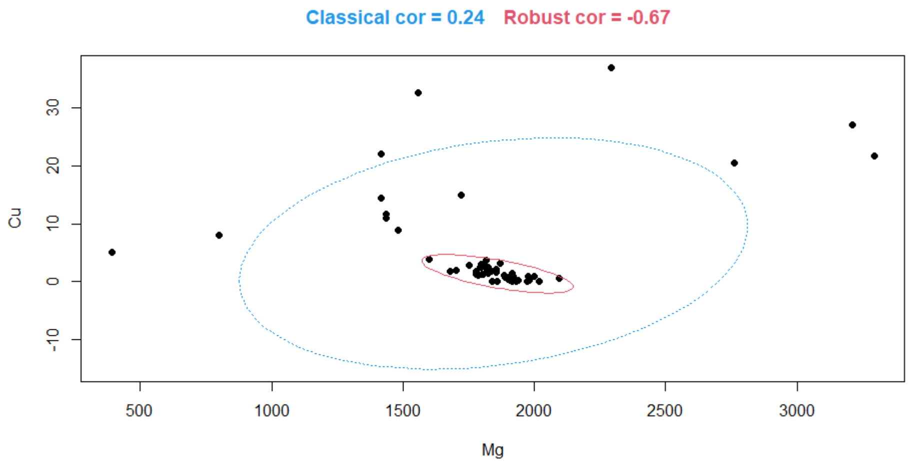

- **As vs Hg, Pb vs Hg (no Hg at all). => Not Applicable.**

- **Pb vs As**

Whole cementum thickness:

Classical test	Pearson's product-moment correlation<br><br>data: Od533M_AC$As and Od533M_AC$Pb<br>t = -0.36151, df = 60, **p-value = 0.719**<br>alternative hypothesis: true correlation is not equal to 0<br>95 percent confidence interval:<br>-0.2929768 0.2055414<br>sample estimates:<br>**cor**<br>**-0.04662013**	Spearman's rank correlation rho<br><br>data: Od533M_AC$As and Od533M_AC$Pb<br>S = 34662, **p-value = 0.3239**<br>alternative hypothesis: true rho is not equal to 0<br>sample estimates:<br>**rho**<br>**0.1271436**
MCD (α=0.05; quant=0.8)	$cor.rob<br>[1] **0.05151116**	

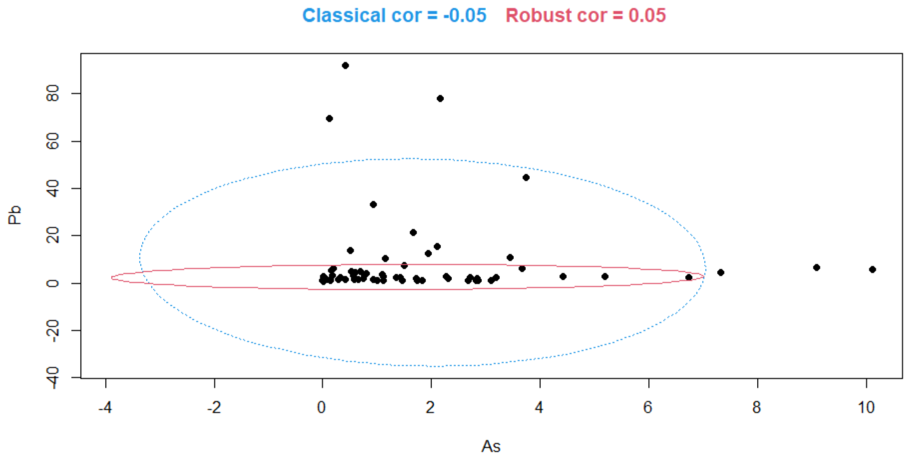

Removing 25 µm of Pb-enriched root surface:

Classical test	Pearson's product-moment correlation<br><br>data: Od533M_AC_wRS$As and Od533M_AC_wRS$Pb<br>t = 2.4433, df = 47, **p-value = 0.01836**<br>alternative hypothesis: true correlation is not equal to 0<br>95 percent confidence interval:<br>0.06020137 0.56369734<br>sample estimates:<br>**cor**<br>**0.3357148**	Spearman's rank correlation rho<br><br>data: Od533M_AC_wRS$As and Od533M_AC_wRS$Pb<br>S = 15974, **p-value = 0.2025**<br>alternative hypothesis: true rho is not equal to 0<br>sample estimates:<br>**rho**<br>**0.185**
MCD (α=0.05; quant=0.8)	$cor.rob<br>[1] **-0.2204023**	

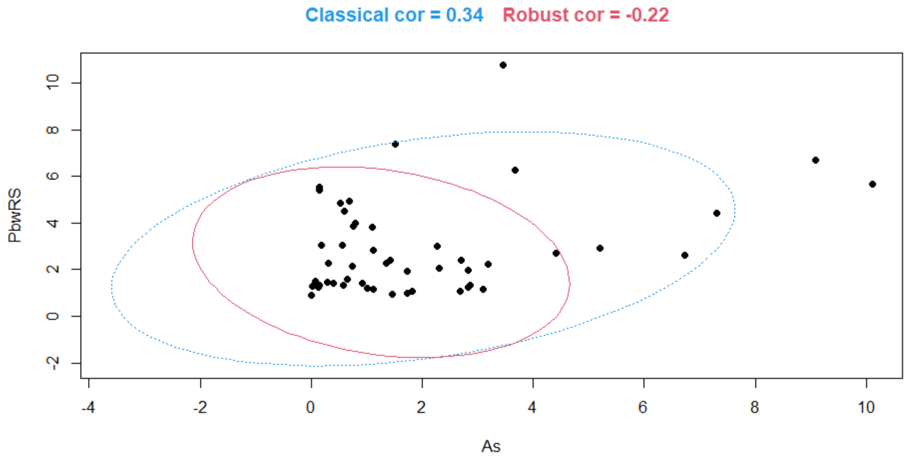

- **Sb vs Sn**

Classical test	Pearson's product-moment correlation<br><br>data: Od533M_AC$Sn and Od533M_AC$Sb<br>t = 0.082198, df = 60, **p-value = 0.9348**<br>alternative hypothesis: true correlation is not equal to 0<br>95 percent confidence interval:<br>-0.2397926 0.2596911<br>sample estimates:<br>**cor**<br>**0.01061118**	Spearman's rank correlation rho<br><br>data: Od533M_AC$Sn and Od533M_AC$Sb<br>S = 26930, **p-value = 0.01104**<br>alternative hypothesis: true rho is not equal to 0<br>sample estimates:<br>**rho**<br>**0.3218504**
MCD (α=0.05; quant=0.8)	$cor.rob<br>[1] **0.4795098**	

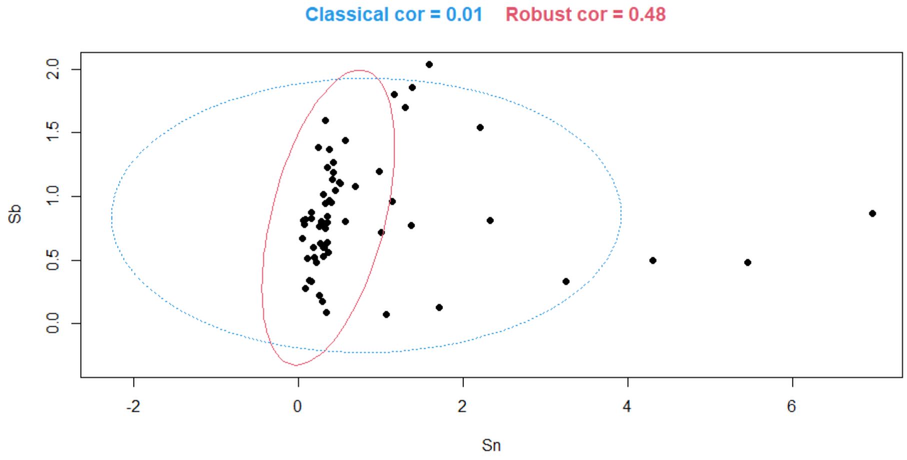

- **Pb vs Sb**

Whole cementum thickness:

Classical test	Pearson's product-moment correlation<br><br>data: Od533M_AC$Sb and Od533M_AC$Pb<br>t = 3.6301, df = 60, **p-value = 0.0005873**<br>alternative hypothesis: true correlation is not equal to 0<br>95 percent confidence interval:<br>0.1952898 0.6095230<br>sample estimates:<br>**cor**<br>**0.424359**	Spearman's rank correlation rho<br><br>data: Od533M_AC$Sb and Od533M_AC$Pb<br>S = 31236, **p-value = 0.09582**<br>alternative hypothesis: true rho is not equal to 0<br>sample estimates:<br>**rho**<br>**0.2134169**
MCD (α=0.05; quant=0.8)	$cor.rob<br>[1] **-0.002296206**	

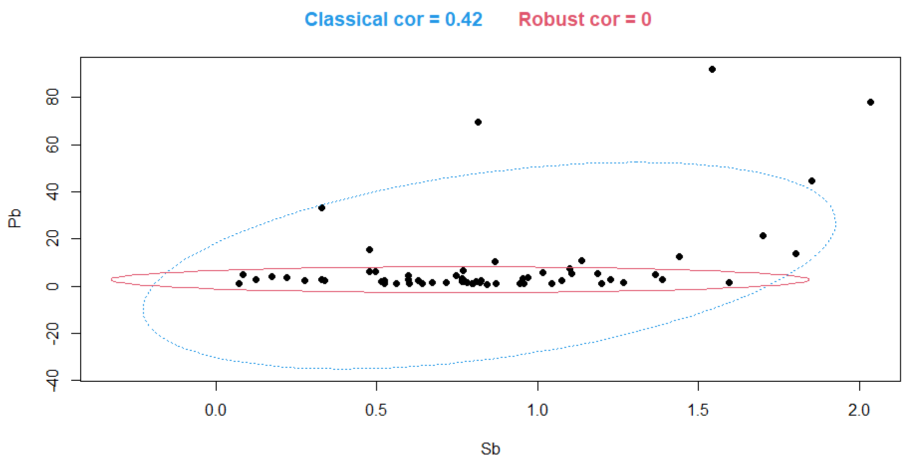

Removing 25 µm of Pb-enriched root surface:

Classical test	Pearson's product-moment correlation<br><br>data: Od533M_AC_wRS$Sb and Od533M_AC_wRS$Pb<br>t = 0.5351, df = 47, **p-value = 0.5951**<br>alternative hypothesis: true correlation is not equal to 0<br>95 percent confidence interval:<br>-0.2079310 0.3513245<br>sample estimates:<br>**cor**<br>**0.07781542**	Spearman's rank correlation rho<br><br>data: Od533M_AC_wRS$Sb and Od533M_AC_wRS$Pb<br>S = 19266, **p-value = 0.9074**<br>alternative hypothesis: true rho is not equal to 0<br>sample estimates:<br>**rho**<br>**0.01704082**
MCD (α=0.05; quant=0.8)	$cor.rob<br>[1] **-0.3605865**	

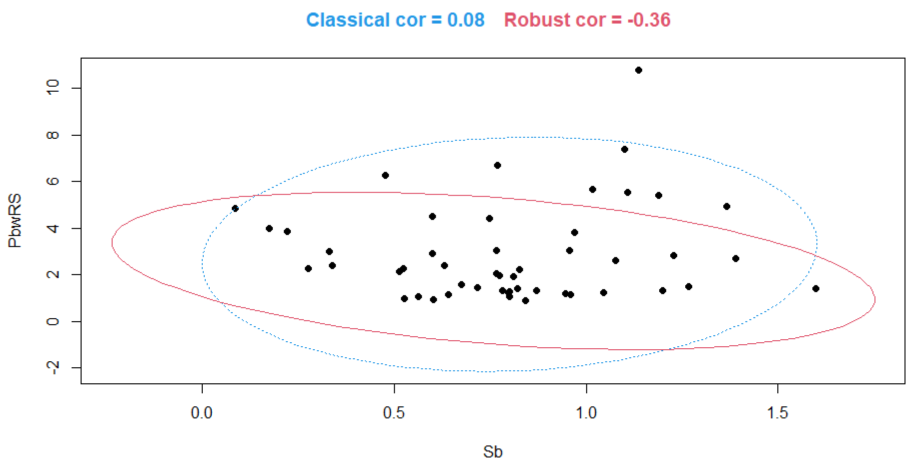

- **Pb vs Sn**

Whole cementum thickness:

Classical test	Pearson's product-moment correlation<br><br>data: Od533M_AC$Sn and Od533M_AC$Pb<br>t = 3.0305, df = 60, **p-value = 0.0036**<br>alternative hypothesis: true correlation is not equal to 0<br>95 percent confidence interval:<br>0.1260418 0.5628855<br>sample estimates:<br>**cor**<br>**0.3643402**	Spearman's rank correlation rho<br><br>data: Od533M_AC$Sn and Od533M_AC$Pb<br>S = 24746, **p-value = 0.002698**<br>alternative hypothesis: true rho is not equal to 0<br>sample estimates:<br>**rho**<br>**0.3768477**
MCD (α=0.05; quant=0.8)	$cor.rob<br>[1] **-0.1243023**	

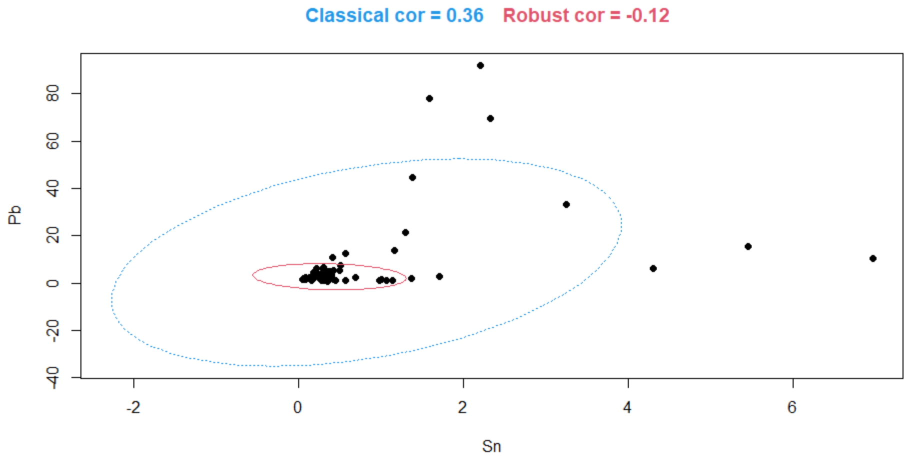

Removing 25 µm of Pb-enriched root surface:

Classical test	Pearson's product-moment correlation<br><br>data: Od533M_AC_wRS$Sn and Od533M_AC_wRS$Pb<br>t = -0.3783, df = 47, **p-value = 0.7069**<br>alternative hypothesis: true correlation is not equal to 0<br>95 percent confidence interval:<br>-0.3311624 0.2296582<br>sample estimates:<br>**cor**<br>**-0.05509644**	Spearman's rank correlation rho<br><br>data: Od533M_AC_wRS$Sn and Od533M_AC_wRS$Pb<br>S = 20154, **p-value = 0.8468**<br>alternative hypothesis: true rho is not equal to 0<br>sample estimates:<br>**rho**<br>**-0.02826531**
MCD (α=0.05; quant=0.8)	$cor.rob<br>[1] **0.2403129**	

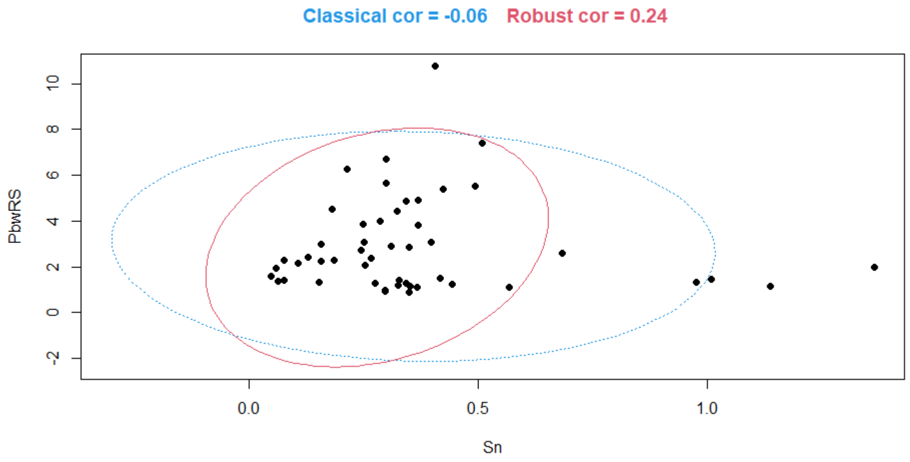

- **Br vs Ba**

Classical test	Pearson's product-moment correlation<br><br>data: Od533M_AC$Ba and Od533M_AC$Br<br>t = 0.18099, df = 60, **p-value = 0.857**<br>alternative hypothesis: true correlation is not equal to 0<br>95 percent confidence interval:<br>-0.2277374 0.2715435<br>sample estimates:<br>**cor**<br>**0.02335957**	Spearman's rank correlation rho<br><br>data: Od533M_AC$Ba and Od533M_AC$Br<br>S = 36878, **p-value = 0.5807**<br>alternative hypothesis: true rho is not equal to 0<br>sample estimates:<br>**rho**<br>**0.07134043**
MCD (α=0.05; quant=0.8)	$cor.rob<br>[1] **0.1710217**	

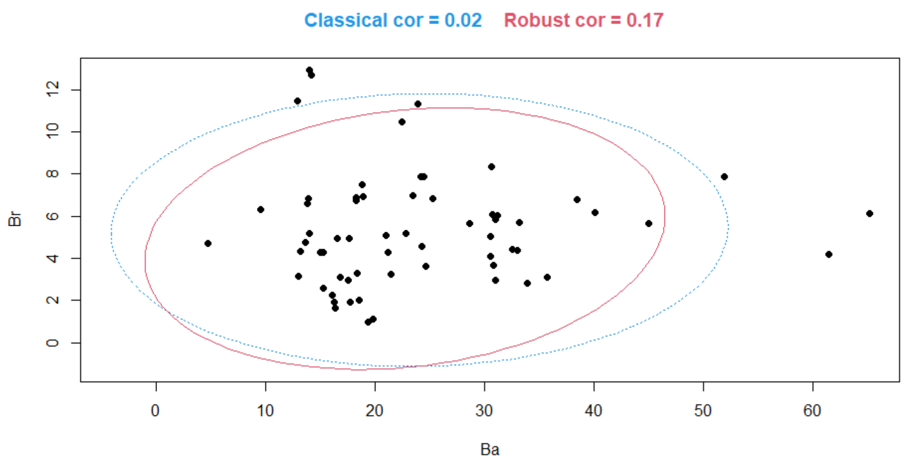

- **Br vs Sr**

Classical test	Pearson's product-moment correlation<br><br>data: Od533M_AC$Sr and Od533M_AC$Br<br>t = -0.85807, df = 60, **p-value = 0.3943**<br>alternative hypothesis: true correlation is not equal to 0<br>95 percent confidence interval:<br>-0.3502397 0.1436145<br>sample estimates:<br>**cor**<br>**-0.1101032**	Spearman's rank correlation rho<br><br>data: Od533M_AC$Sr and Od533M_AC$Br<br>S = 41026, **p-value = 0.7979**<br>alternative hypothesis: true rho is not equal to 0<br>sample estimates:<br>**rho**<br>**-0.03311425**
MCD (α=0.05; quant=0.8)	$cor.rob<br>[1] **0.1626547**	

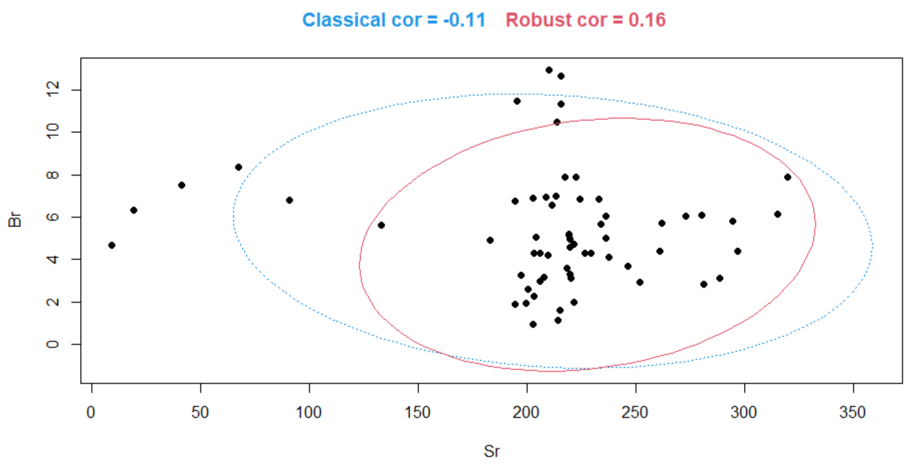

- **Br vs Zn**

Classical test	Pearson's product-moment correlation<br><br>data: Od533M_AC$Zn and Od533M_AC$Br<br>t = -3.1715, df = 60, **p-value = 0.00239**<br>alternative hypothesis: true correlation is not equal to 0<br>95 percent confidence interval:<br>-0.5743247 -0.1426418<br>sample estimates:<br>**cor**<br>**-0.3789104**	Spearman's rank correlation rho<br><br>data: Od533M_AC$Zn and Od533M_AC$Br<br>S = 55904, **p-value = 0.001099**<br>alternative hypothesis: true rho is not equal to 0<br>sample estimates:<br>**rho**<br>**-0.4077711**
MCD (α=0.05; quant=0.8)	$cor.rob<br>[1] **-0.3694156**	

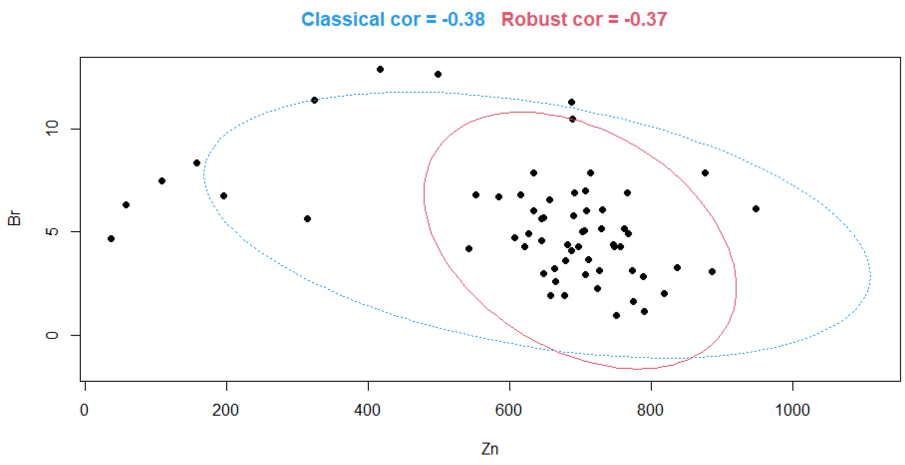

- **Br vs Ca**

Classical test	Pearson's product-moment correlation<br><br>data: Od533M_AC$Ca and Od533M_AC$Br<br>t = -1.1183, df = 60, **p-value = 0.2679**<br>alternative hypothesis: true correlation is not equal to 0<br>95 percent confidence interval:<br>-0.3791260 0.1108354<br>sample estimates:<br>**cor**<br>**-0.1428884**	Spearman's rank correlation rho<br><br>data: Od533M_AC$Ca and Od533M_AC$Br<br>S = 44066, **p-value = 0.3952**<br>alternative hypothesis: true rho is not equal to 0<br>sample estimates:<br>**rho**<br>**-0.1096673**
MCD (α=0.05; quant=0.8)	$cor.rob<br>[1] **0.1626547**	

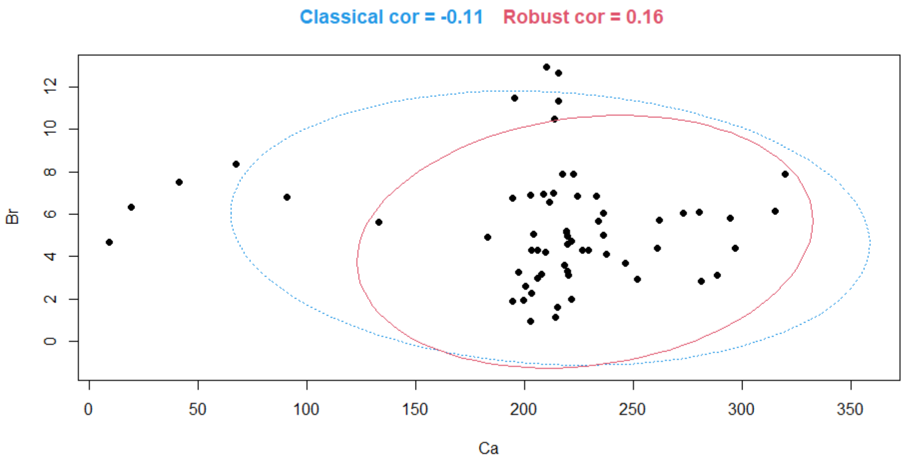
Classical cor = -0.11   Robust cor = 0.16
Br
0
2
4
6
8
10
12
0
50
100
150
200
250
300
350
Ca

Odense 533M1 – Cellular Cementum (total thickness=694 µm)

Correlations between pairs of elements

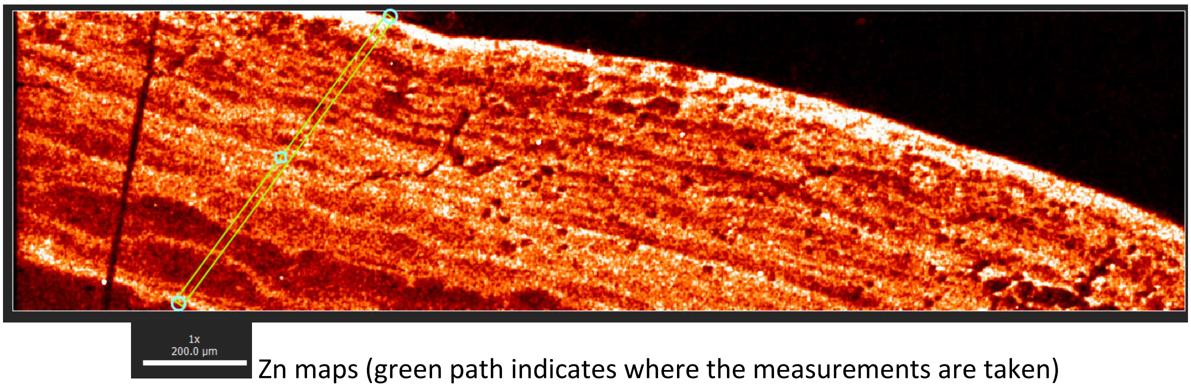

Zn maps (green path indicates where the measurements are taken)

Summary : <u>whole cementum</u> thickness, Spearman correlations.

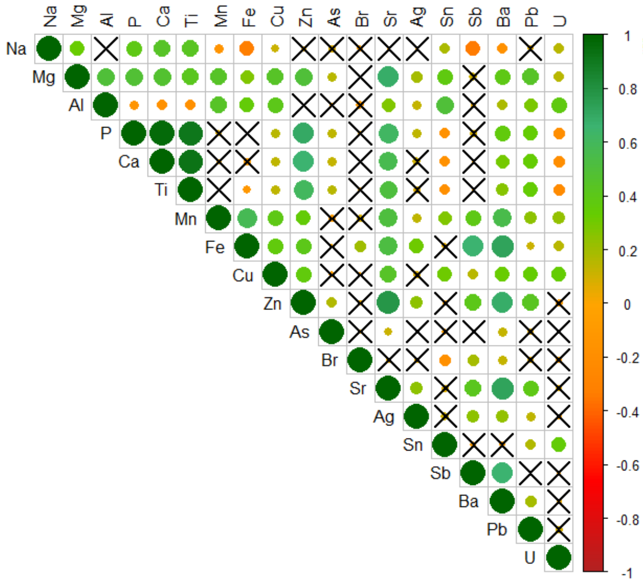

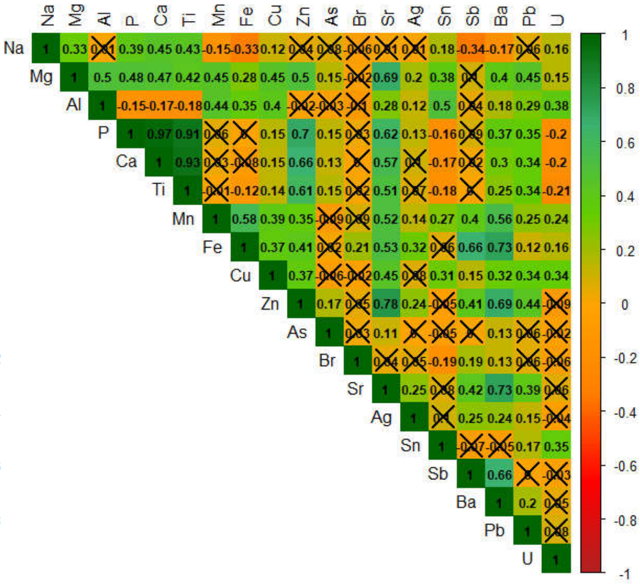

Summary : removing the first 15 µm of root surface, Spearman correlations.

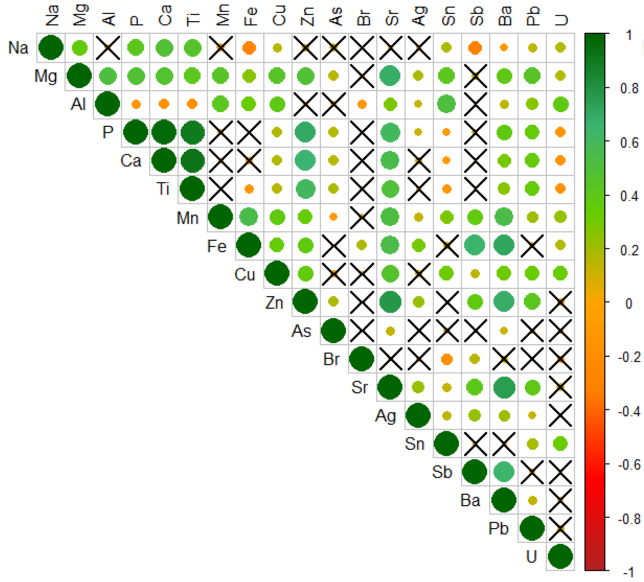

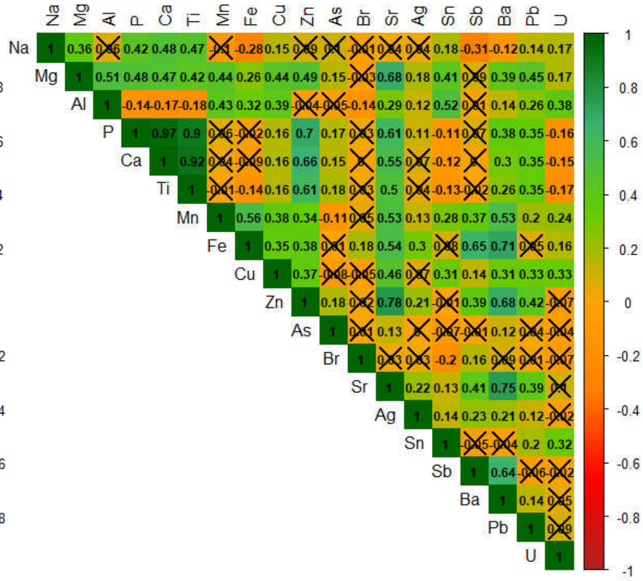

wRS: without Root surface [Pb = ~15 µm; Fe, Mn, Al not included in the path]

- **Ca vs Ba**

Classical test	Pearson's product-moment correlation<br><br>data: Od533M_CC$Ca and Od533M_CC$Ba<br>t = -1.192, df = 346, **p-value = 0.2341**<br>alternative hypothesis: true correlation is not equal to 0<br>95 percent confidence interval:<br>-0.16795239 0.04145918<br>sample estimates:<br>**cor**<br>**-0.06395056**	Spearman's rank correlation rho<br><br>data: Od533M_CC$Ca and Od533M_CC$Ba<br>S = 4937240, **p-value = 1.881e-08**<br>alternative hypothesis: true rho is not equal to 0<br>sample estimates:<br>**rho**<br>**0.2970874**
MCD (α=0.05; quant=0.8)	$cor.rob<br>[1] **0.4222214**	

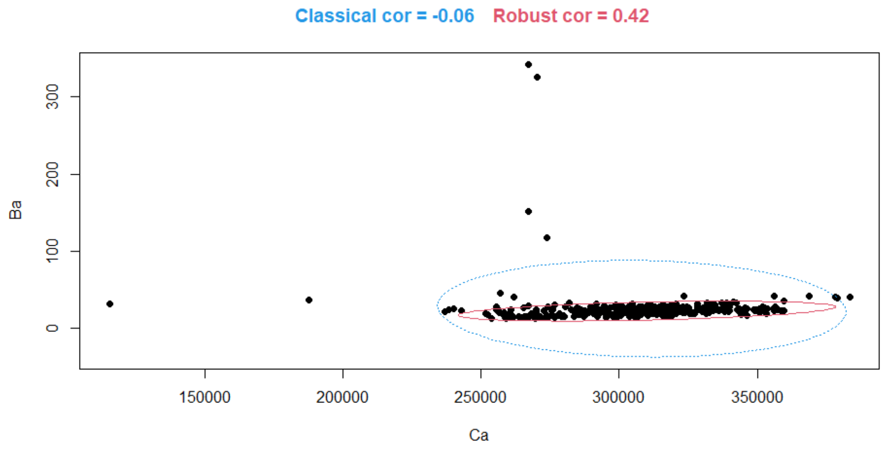

- **Ca vs Sr**

Classical test	Pearson's product-moment correlation<br><br>data: Od533M_CC$Ca and Od533M_CC$Sr<br>t = 8.5503, df = 346, **p-value = 4.012e-16**<br>alternative hypothesis: true correlation is not equal to 0<br>95 percent confidence interval:<br>0.3268786 0.5007984<br>sample estimates:<br>**cor**<br>**0.4176568**	Spearman's rank correlation rho<br><br>data: Od533M_CC$Ca and Od533M_CC$Sr<br>S = 3051902, **p-value < 2.2e-16**<br>alternative hypothesis: true rho is not equal to 0<br>sample estimates:<br>**rho**<br>**0.5655021**
MCD (α=0.05; quant=0.8)	$cor.rob<br>[1] **0.7877556**	

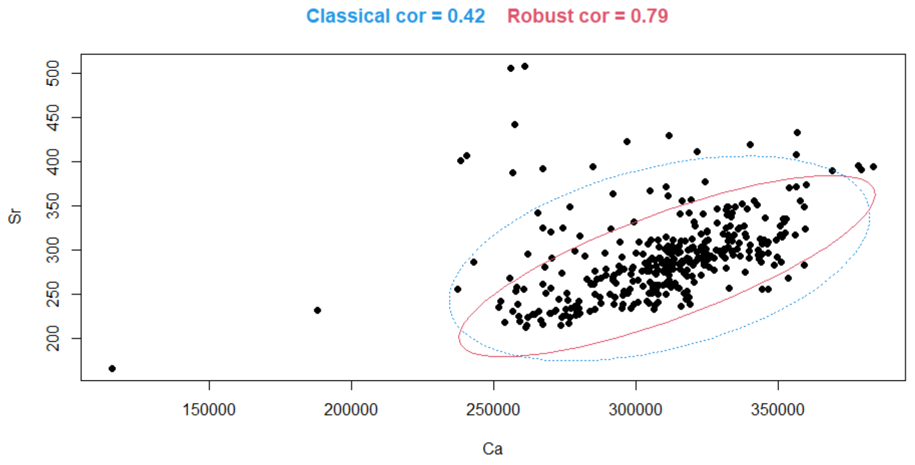

- **Ba vs Sr**

<table>
<tr><td rowspan="1">Classical test</td><td>Pearson's product-moment correlation

data: Od533M_CC$Ba and Od533M_CC$Sr
t = 3.768, df = 346, p-value = 0.0001934
alternative hypothesis: true correlation is not equal to 0
95 percent confidence interval:
0.09539602 0.29745827
sample estimates:
cor
0.1985359</td><td>Spearman's rank correlation rho

data: Od533M_CC$Ba and Od533M_CC$Sr
S = 1899564, p-value < 2.2e-16
alternative hypothesis: true rho is not equal to 0
sample estimates:
rho
0.7295599</td></tr>
<tr><td>MCD (α=0.05; quant=0.8)</td><td colspan="2">$cor.rob
[1] 0.8230075</td></tr>
</table>

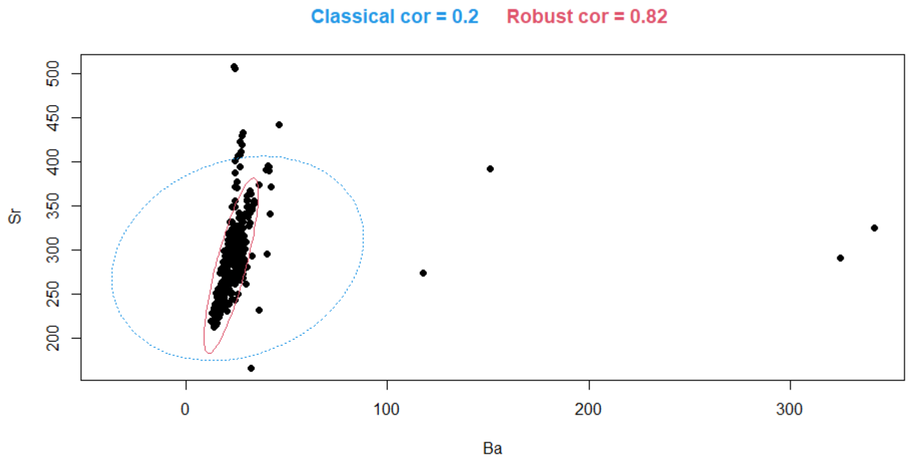

- **Zn vs Sr**

Classical test	Pearson's product-moment correlation<br><br>data: Od533M_CC$Zn and Od533M_CC$Sr<br>t = 17.291, df = 346, **p-value < 2.2e-16**<br>alternative hypothesis: true correlation is not equal to 0<br>95 percent confidence interval:<br>0.6201049 0.7334794<br>sample estimates:<br>**cor**<br>**0.6808497**	Spearman's rank correlation rho<br><br>data: Od533M_CC$Zn and Od533M_CC$Sr<br>S = 1538210, **p-value < 2.2e-16**<br>alternative hypothesis: true rho is not equal to 0<br>sample estimates:<br>**rho**<br>**0.7810057**
MCD (α=0.05; quant=0.8)	$cor.rob<br>[1] **0.8659534**	

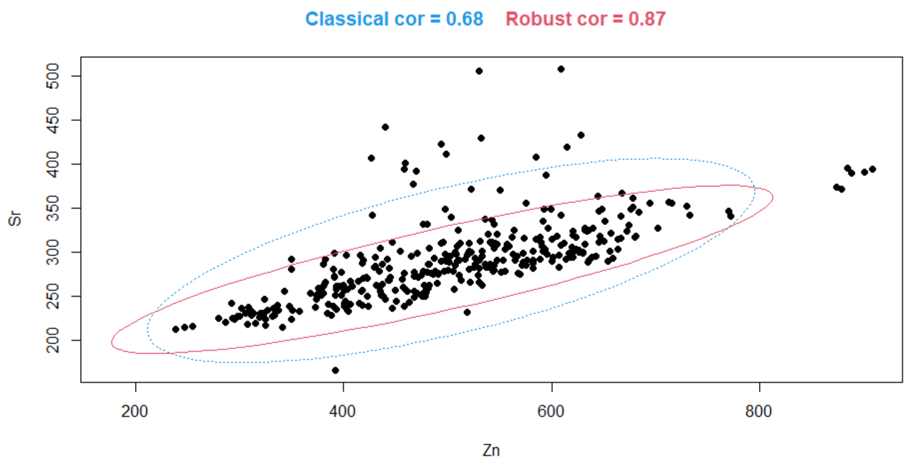

- **Mg vs Sr**

Classical test	Pearson's product-moment correlation<br><br>data: Od533M_CC$Mg and Od533M_CC$Sr<br>t = 14.517, df = 346, **p-value < 2.2e-16**<br>alternative hypothesis: true correlation is not equal to 0<br>95 percent confidence interval:<br>0.5453866 0.6766088<br>sample estimates:<br>**cor**<br>**0.6152415**	Spearman's rank correlation rho<br><br>data: Od533M_CC$Mg and Od533M_CC$Sr<br>S = 2212532, **p-value < 2.2e-16**<br>alternative hypothesis: true rho is not equal to 0<br>sample estimates:<br>**rho**<br>**0.6850028**
MCD (α=0.05; quant=0.8)	$cor.rob<br>[1] **0.6402011**	

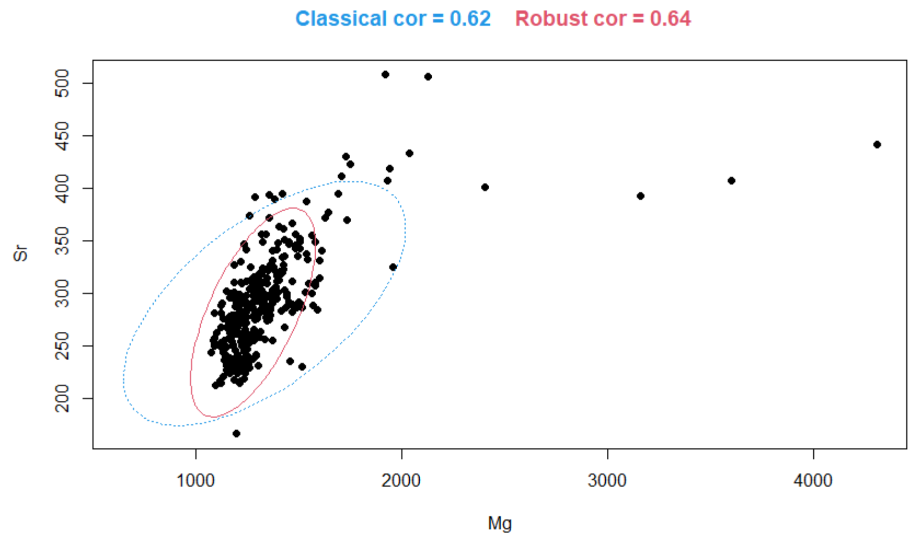

- **Pb vs Sr**

Whole cementum thickness:

Classical test	Pearson's product-moment correlation<br><br>data: Od533M_CC$Sr and Od533M_CC$Pb<br>t = 0.55828, df = 346, **p-value = 0.577**<br>alternative hypothesis: true correlation is not equal to 0<br>95 percent confidence interval:<br>-0.07536916 0.13470571<br>sample estimates:<br>**cor**<br>**0.02999955**	Spearman's rank correlation rho<br><br>data: Od533M_CC$Sr and Od533M_CC$Pb<br>S = 4282912, **p-value = 4.798e-14**<br>alternative hypothesis: true rho is not equal to 0<br>sample estimates:<br>**rho**<br>**0.3902438**
MCD (α=0.05; quant=0.8)	$cor.rob<br>[1] **0.4150683**	

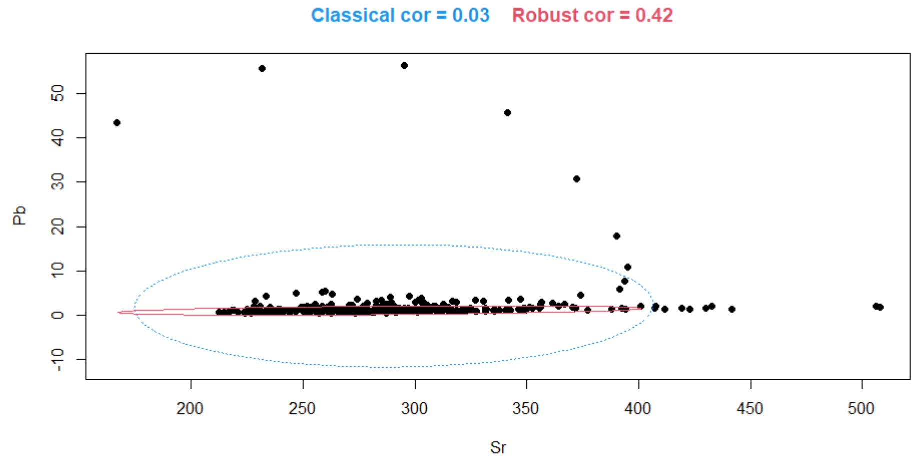

Removing 15 µm of Pb-enriched root surface:

Classical test	Pearson's product-moment correlation<br><br>data: Od533M_CC_wRS$Sr and Od533M_CC_wRS$Pb<br>t = 4.1906, df = 338, **p-value = 3.556e-05**<br>alternative hypothesis: true correlation is not equal to 0<br>95 percent confidence interval:<br>0.1186810 0.3210121<br>sample estimates:<br>**cor**<br>**0.2222379**	Spearman's rank correlation rho<br><br>data: Od533M_CC_wRS$Zn and Od533M_CC_wRS$Pb<br>S = 3812334, **p-value < 2.2e-16**<br>alternative hypothesis: true rho is not equal to 0<br>sample estimates:<br>**rho**<br>**0.4180185**
MCD (α=0.05; quant=0.8)	$cor.rob<br>[1] **0.435669**	

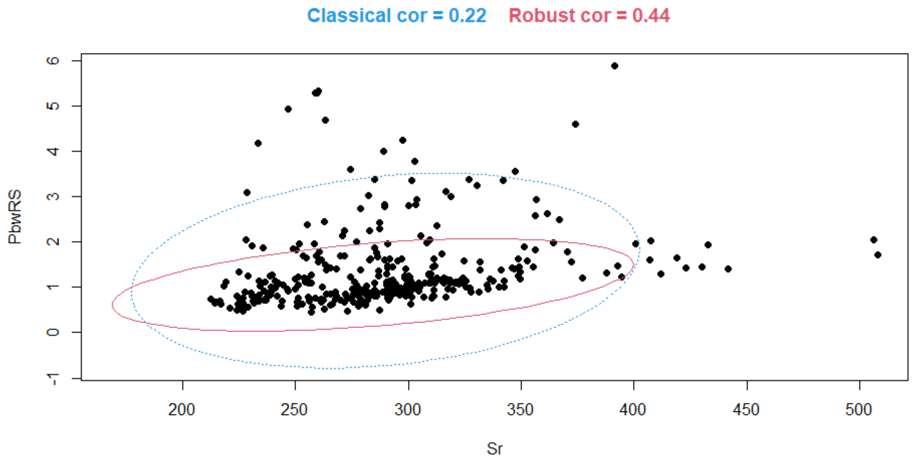

- **Sr vs Zn**

Classical test	Pearson's product-moment correlation<br><br>data: Od533M_CC$Zn and Od533M_CC$Sr<br>t = 17.291, df = 346, **p-value < 2.2e-16**<br>alternative hypothesis: true correlation is not equal to 0<br>95 percent confidence interval:<br>0.6201049 0.7334794<br>sample estimates:<br>**cor**<br>**0.6808497**	Spearman's rank correlation rho<br><br>data: Od533M_CC$Zn and Od533M_CC$Sr<br>S = 1538210, **p-value < 2.2e-16**<br>alternative hypothesis: true rho is not equal to 0<br>sample estimates:<br>**rho**<br>**0.7810057**
MCD (α=0.05; quant=0.8)	$cor.rob<br>[1] **0.8659534**	

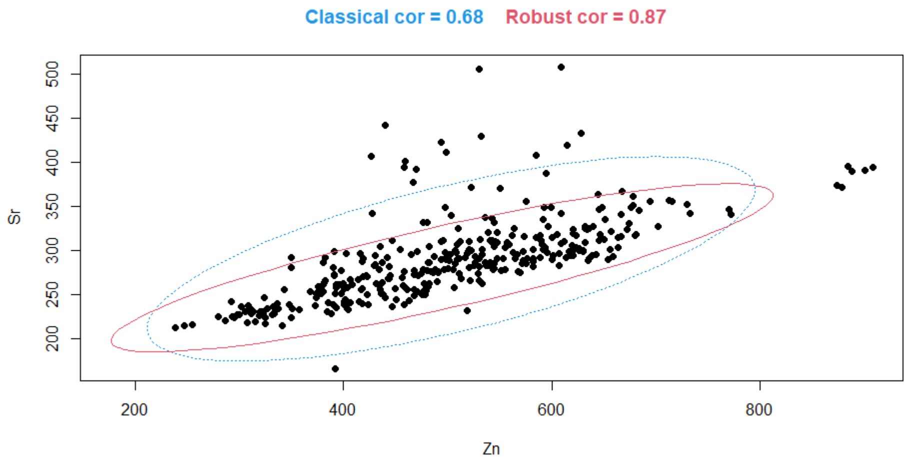

- **Pb vs Zn**

Whole cementum thickness:

Classical test	Pearson's product-moment correlation<br><br>data: Od533M_CC$Zn and Od533M_CC$Pb<br>t = 4.2036, df = 346, **p-value = 3.348e-05**<br>alternative hypothesis: true correlation is not equal to 0<br>95 percent confidence interval:<br>0.1180325 0.3181857<br>sample estimates:<br>**cor**<br>**0.2204283**	Spearman's rank correlation rho<br><br>data: Od533M_CC$Zn and Od533M_CC$Pb<br>S = 3949120, **p-value < 2.2e-16**<br>alternative hypothesis: true rho is not equal to 0<br>sample estimates:<br>**rho**<br>**0.4377656**
MCD (α=0.05; quant=0.8)	$cor.rob<br>[1] **0.3801596**	

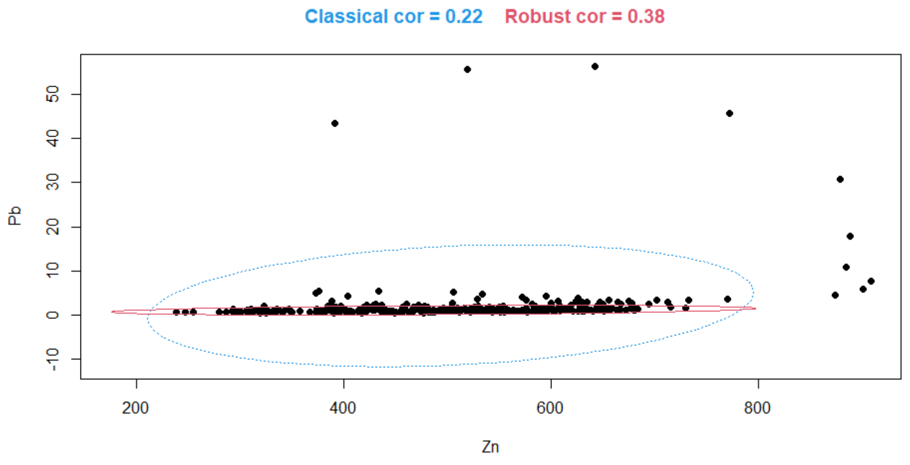

Removing 15 µm of Pb-enriched root surface:

Classical test	Pearson's product-moment correlation<br><br>data: Od533M_CC_wRS$Zn and Od533M_CC_wRS$Pb<br>t = 7.2751, df = 338, **p-value = 2.435e-12**<br>alternative hypothesis: true correlation is not equal to 0<br>95 percent confidence interval:<br>0.2722422 0.4564486<br>sample estimates:<br>**cor**<br>**0.3679499**	Spearman's rank correlation rho<br><br>data: Od533M_CC_wRS$Zn and Od533M_CC_wRS$Pb<br>S = 3812334, **p-value < 2.2e-16**<br>alternative hypothesis: true rho is not equal to 0<br>sample estimates:<br>**rho**<br>**0.4180185**
MCD (α=0.05; quant=0.8)	$cor.rob<br>[1] **0.4035045**	

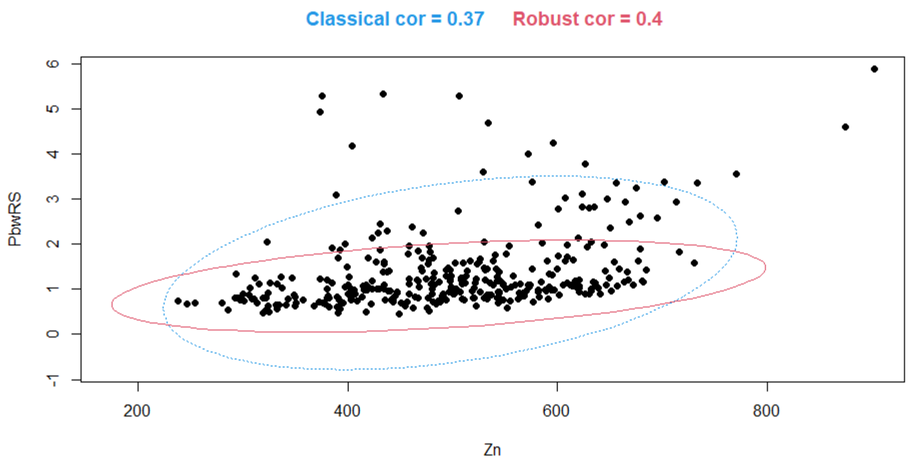

- **Zn vs Ca**

Classical test	Pearson's product-moment correlation<br><br>data: Od533M_CC$Zn and Od533M_CC$Ca<br>t = 15.055, df = 346, **p-value < 2.2e-16**<br>alternative hypothesis: true correlation is not equal to 0<br>95 percent confidence interval:<br>0.5611166 0.6887129<br>sample estimates:<br>**cor**<br>**0.6291345**	Spearman's rank correlation rho<br><br>data: Od533M_CC$Zn and Od533M_CC$Ca<br>S = 2381548, **p-value < 2.2e-16**<br>alternative hypothesis: true rho is not equal to 0<br>sample estimates:<br>**rho**<br>**0.6609401**
MCD (α=0.05; quant=0.8)	$cor.rob<br>[1] **0.710546**	

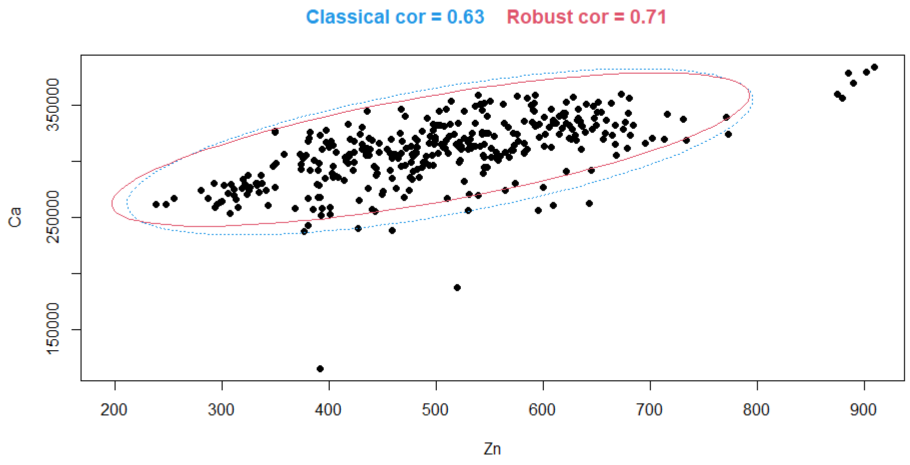

- **Zn vs Fe**

Classical test	Pearson's product-moment correlation<br><br>data: Od533M_CC$Zn and Od533M_CC$Fe<br>t = 4.0683, df = 346, **p-value = 5.869e-05**<br>alternative hypothesis: true correlation is not equal to 0<br>95 percent confidence interval:<br>0.1110267 0.3117911<br>sample estimates:<br>**cor**<br>**0.2136638**	Spearman's rank correlation rho<br><br>data: Od533M_CC$Zn and Od533M_CC$Fe<br>S = 4179194, **p-value = 1.234e-15**<br>alternative hypothesis: true rho is not equal to 0<br>sample estimates:<br>**rho**<br>**0.40501**
MCD (α=0.05; quant=0.8)	$cor.rob<br>[1] **0.4823483**	

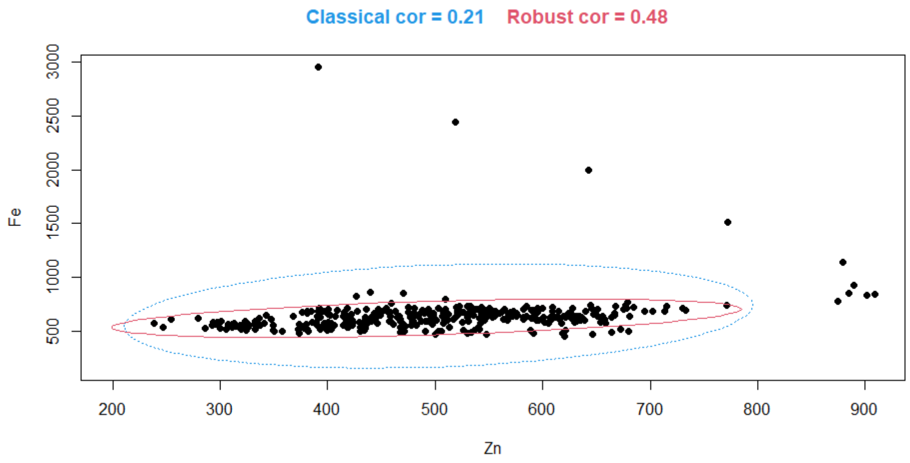

- **Zn vs Cu**

Classical test	Pearson's product-moment correlation<br><br>data: Od533M_CC$Zn and Od533M_CC$Cu<br>t = 5.3372, df = 346, **p-value = 1.712e-07**<br>alternative hypothesis: true correlation is not equal to 0<br>95 percent confidence interval:<br>0.1757652 0.3701970<br>sample estimates:<br>**cor**<br>**0.2757999**	Spearman's rank correlation rho<br><br>data: Od533M_CC$Zn and Od533M_CC$Cu<br>S = 4437736, **p-value = 1.792e-12**<br>alternative hypothesis: true rho is not equal to 0<br>sample estimates:<br>**rho**<br>**0.3682015**
MCD (α=0.05; quant=0.8)	$cor.rob<br>[1] **0.2541785**	

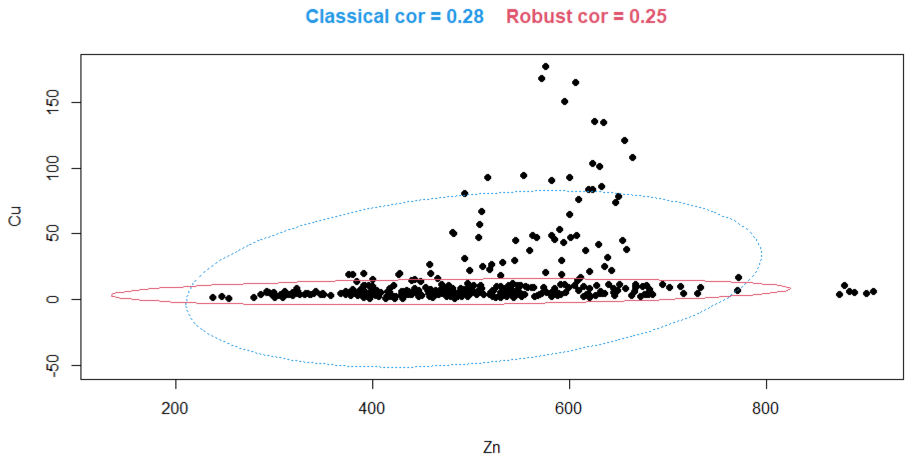

- **Zn vs Mg**

Classical test	Pearson's product-moment correlation<br><br>data: Od533M_CC$Zn and Od533M_CC$Mg<br>t = 3.2595, df = 346, **p-value = 0.001227**<br>alternative hypothesis: true correlation is not equal to 0<br>95 percent confidence interval:<br>0.06871983 0.27278489<br>sample estimates:<br>**cor**<br>**0.1726038**	Spearman's rank correlation rho<br><br>data: Od533M_CC$Zn and Od533M_CC$Mg<br>S = 3506054, **p-value < 2.2e-16**<br>alternative hypothesis: true rho is not equal to 0<br>sample estimates:<br>**rho**<br>**0.5008447**
MCD (α=0.05; quant=0.8)	$cor.rob<br>[1] **0.5550434**	

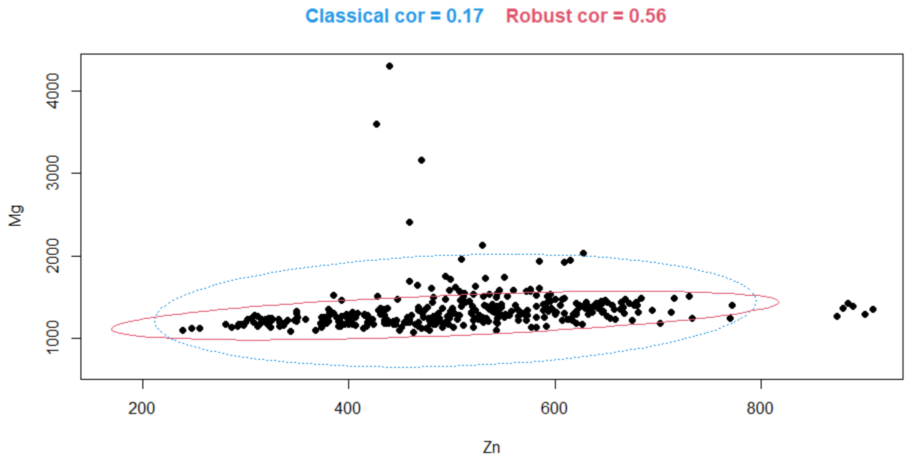

- **Ca vs Fe**

Classical test	Pearson's product-moment correlation<br><br>data: Od533M_CC$Ca and Od533M_CC$Fe<br>t = -6.9981, df = 346, **p-value = 1.352e-11**<br>alternative hypothesis: true correlation is not equal to 0<br>95 percent confidence interval:<br>-0.4409332 -0.2564893<br>sample estimates:<br>**cor**<br>**-0.3521252**	Spearman's rank correlation rho<br><br>data: Od533M_CC$Ca and Od533M_CC$Fe<br>S = 7553372, **p-value = 0.1606**<br>alternative hypothesis: true rho is not equal to 0<br>sample estimates:<br>**rho**<br>**-0.07537015**
MCD (α=0.05; quant=0.8)	$cor.rob<br>[1] **-0.04928535**	

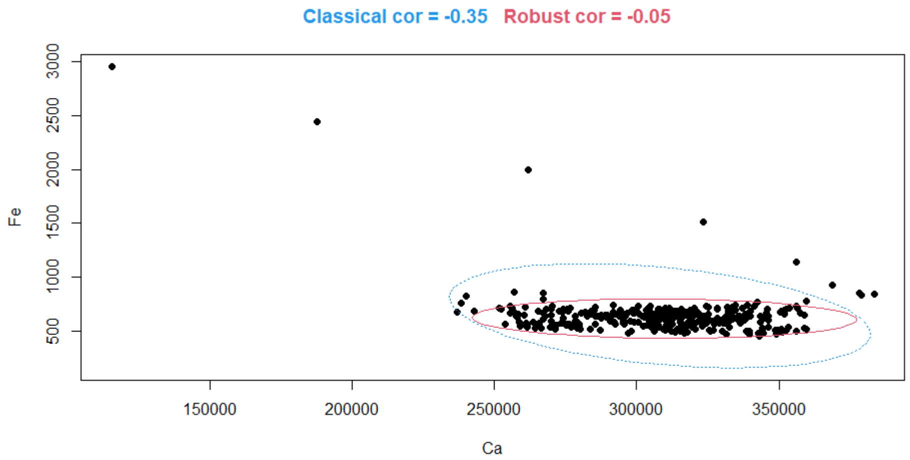

- **Ca vs Mg**

Classical test	Pearson's product-moment correlation<br><br>data: Od533M_CC$Ca and Od533M_CC$Mg<br>t = 0.28031, df = 346, **p-value = 0.7794**<br>alternative hypothesis: true correlation is not equal to 0<br>95 percent confidence interval:<br>-0.09020595 0.12000885<br>sample estimates:<br>**cor**<br>**0.01506795**	Spearman's rank correlation rho<br><br>data: Od533M_CC$Ca and Od533M_CC$Mg<br>S = 3704688, **p-value < 2.2e-16**<br>alternative hypothesis: true rho is not equal to 0<br>sample estimates:<br>**rho**<br>**0.4725652**
MCD (α=0.05; quant=0.8)	$cor.rob<br>[1] **0.6241344**	

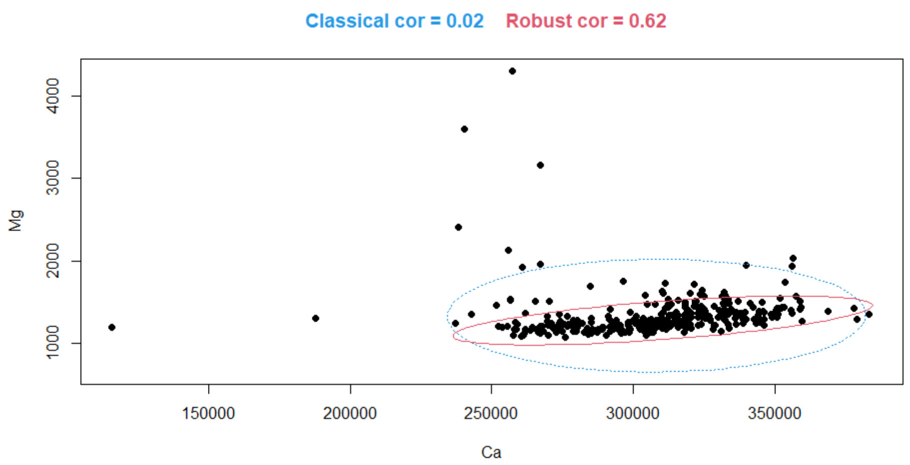

- **Ca vs P**

Classical test	Pearson's product-moment correlation<br><br>data: Od533M_CC$Ca and Od533M_CC$P<br>t = 83.901, df = 346, **p-value < 2.2e-16**<br>alternative hypothesis: true correlation is not equal to 0<br>95 percent confidence interval:<br>0.9708054 0.9807610<br>sample estimates:<br>**cor**<br>**0.9762941**	Spearman's rank correlation rho<br><br>data: Od533M_CC$Ca and Od533M_CC$P<br>S = 200644, **p-value < 2.2e-16**<br>alternative hypothesis: true rho is not equal to 0<br>sample estimates:<br>**rho**<br>**0.9714344**
MCD (α=0.05; quant=0.8)	$cor.rob<br>[1] **0.9763796**	

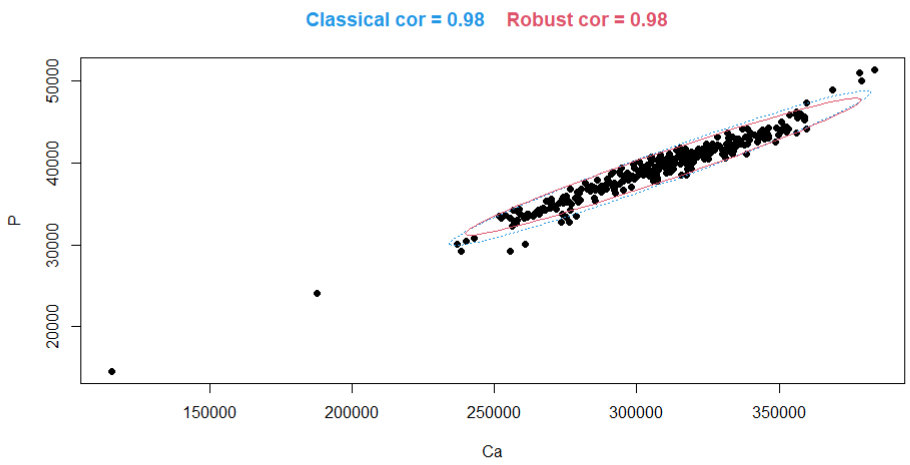

- **Cu vs Fe**

Classical test	Pearson's product-moment correlation<br><br>data: Od533M_CC$Cu and Od533M_CC$Fe<br>t = 0.9357, df = 346, **p-value = 0.3501**<br>alternative hypothesis: true correlation is not equal to 0<br>95 percent confidence interval:<br>-0.05518252 0.15455463<br>sample estimates:<br>**cor**<br>**0.05023995**	Spearman's rank correlation rho<br><br>data: Od533M_CC$Cu and Od533M_CC$Fe<br>S = 4457044, **p-value = 2.702e-12**<br>alternative hypothesis: true rho is not equal to 0<br>sample estimates:<br>**rho**<br>**0.3654527**
MCD (α=0.05; quant=0.8)	$cor.rob<br>[1] **0.3913524**	

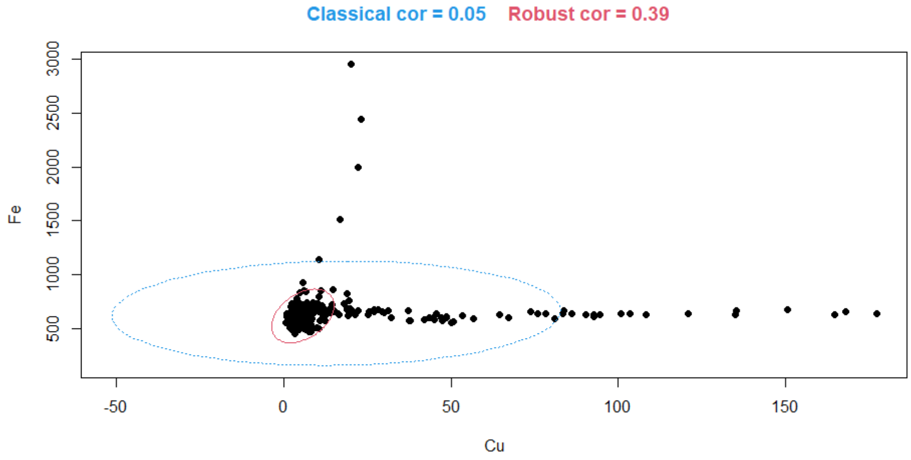

- **Mg vs Fe**

Classical test	Pearson's product-moment correlation<br><br>data: Od533M_CC$Mg and Od533M_CC$Fe<br>t = 2.6355, df = 346, **p-value = 0.00878**<br>alternative hypothesis: true correlation is not equal to 0<br>95 percent confidence interval:<br>0.03567788 0.24184694<br>sample estimates:<br>**cor**<br>**0.1402827**	Spearman's rank correlation rho<br><br>data: Od533M_CC$Mg and Od533M_CC$Fe<br>S = 5072070, **p-value = 1.543e-07**<br>alternative hypothesis: true rho is not equal to 0<br>sample estimates:<br>**rho**<br>**0.2778917**
MCD (α=0.05; quant=0.8)	$cor.rob<br>[1] **0.1465132**	

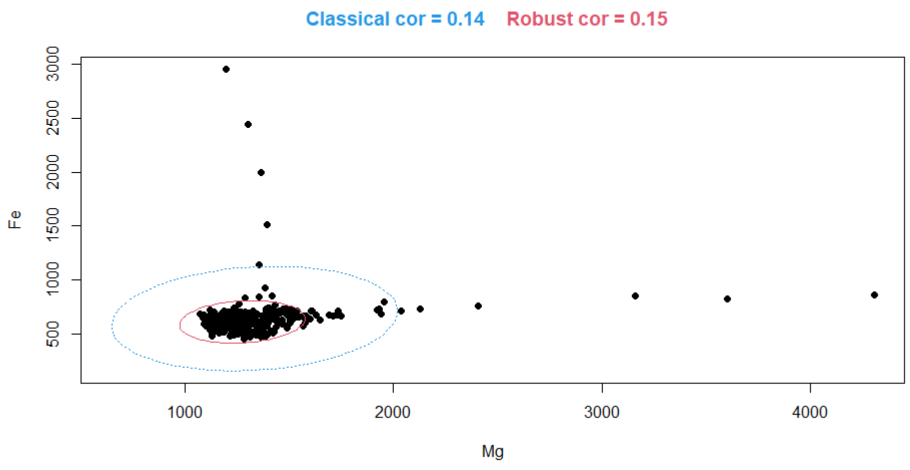

- **Cu vs Mg**

Classical test	Pearson's product-moment correlation<br><br>data: Od533M_CC$Mg and Od533M_CC$Cu<br>t = 3.1556, df = 346, **p-value = 0.001742**<br>alternative hypothesis: true correlation is not equal to 0<br>95 percent confidence interval:<br>0.06323774 0.26768091<br>sample estimates:<br>**cor**<br>**0.1672568**	Spearman's rank correlation rho<br><br>data: Od533M_CC$Mg and Od533M_CC$Cu<br>S = 3894676, **p-value < 2.2e-16**<br>alternative hypothesis: true rho is not equal to 0<br>sample estimates:<br>**rho**<br>**0.4455167**
MCD (α=0.05; quant=0.8)	$cor.rob<br>[1] **0.2762911**	

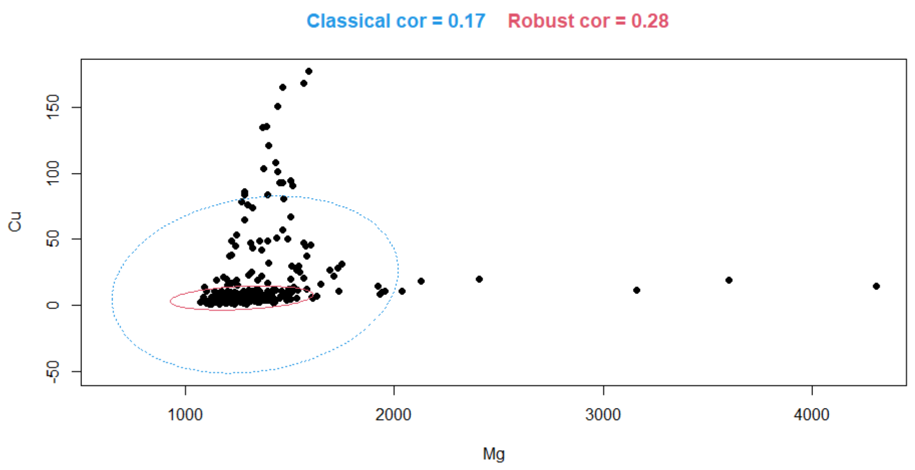

- **As vs Hg, Pb vs Hg (no Hg at all). => Not Applicable.**

- **Pb vs As**

Whole cementum thickness:

	Pearson's product-moment correlation	Spearman's rank correlation rho
Classical test	data: Od533M_CC$As and Od533M_CC$Pb<br>t = 1.2663, df = 346, **p-value = 0.2063**<br>alternative hypothesis: true correlation is not equal to 0<br>95 percent confidence interval:<br>-0.03747891 0.17182381<br>sample estimates:<br>**cor**<br>**0.06791971**	data: Od533M_CC$As and Od533M_CC$Pb<br>S = 6636242, **p-value = 0.3045**<br>alternative hypothesis: true rho is not equal to 0<br>sample estimates:<br>**rho**<br>**0.05520125**<br>*Impossible to calculate the exact p-value with ex-aequos*
MCD (α=0.05; quant=0.8)	$cor.rob<br>[1] **-0.01183972**	

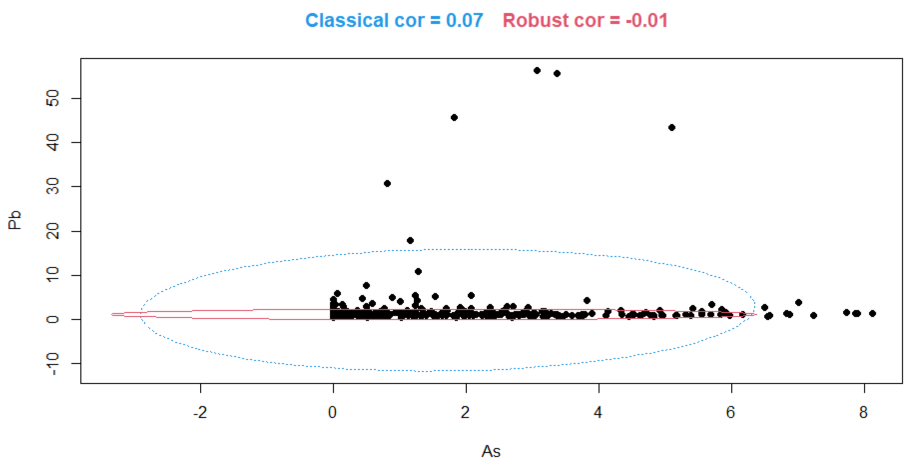

Removing 15 µm of Pb-enriched root surface:

Classical test	Pearson's product-moment correlation<br><br>data: Od533M_CC_wRS$As and Od533M_CC_wRS$Pb<br>t = -0.33905, df = 338, **p-value = 0.7348**<br>alternative hypothesis: true correlation is not equal to 0<br>95 percent confidence interval:<br>-0.12455677 0.08809614<br>sample estimates:<br>**cor**<br>**-0.01843885**	Spearman's rank correlation rho<br><br>data: Od533M_CC_wRS$As and Od533M_CC_wRS$Pb<br>S = 6300312, **p-value = 0.4825**<br>alternative hypothesis: true rho is not equal to 0<br>sample estimates:<br>**rho**<br>**0.03820989**<br>*Impossible to calculate the exact p-value with ex-aequos*
MCD (α=0.05; quant=0.8)	$cor.rob<br>[1] **-0.01645062**	

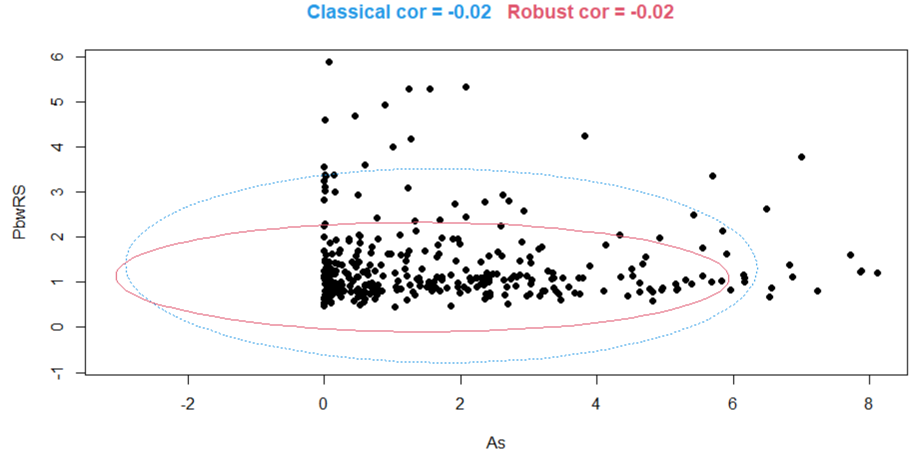

- **Sb vs Sn**

Classical test	Pearson's product-moment correlation<br><br>data: Od533M_CC$Sn and Od533M_CC$Sb<br>t = -1.5008, df = 346, **p-value = 0.1343**<br>alternative hypothesis: true correlation is not equal to 0<br>95 percent confidence interval:<br>-0.18399834 0.02491866<br>sample estimates:<br>**cor**<br>**-0.08042303**	Spearman's rank correlation rho<br><br>data: Od533M_CC$Sn and Od533M_CC$Sb<br>S = 7514274, **p-value = 0.1938**<br>alternative hypothesis: true rho is not equal to 0<br>sample estimates:<br>**rho**<br>**-0.06980379**
MCD (α=0.05; quant=0.8)	$cor.rob<br>[1] **-0.07359742**	

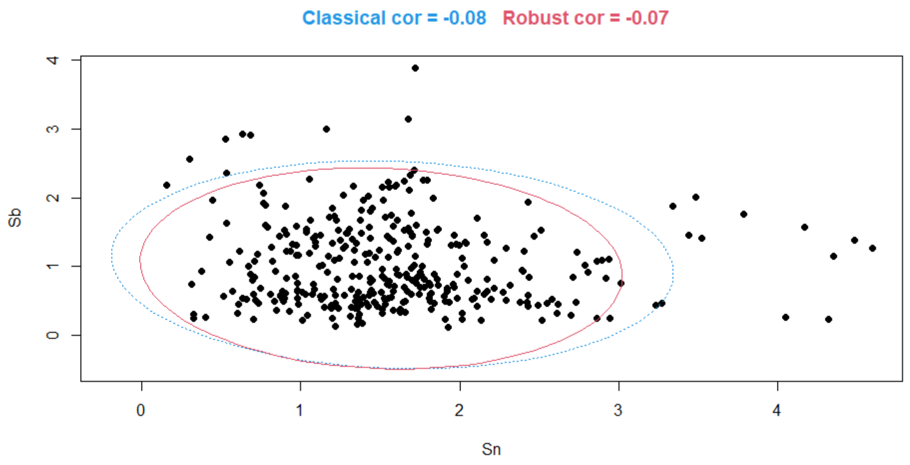

- **Pb vs Sb**

Whole cementum thickness:

Classical test	Pearson's product-moment correlation<br><br>data: Od533M_CC$Sb and Od533M_CC$Pb<br>t = 3.0237, df = 346, **p-value = 0.002684**<br>alternative hypothesis: true correlation is not equal to 0<br>95 percent confidence interval:<br>0.05626567 0.26117312<br>sample estimates:<br>**cor**<br>**0.1604476**	Spearman's rank correlation rho<br><br>data: Od533M_CC$Sb and Od533M_CC$Pb<br>S = 7033012, **p-value = 0.9809**<br>alternative hypothesis: true rho is not equal to 0<br>sample estimates:<br>**rho**<br>**-0.001286736**
MCD (α=0.05; quant=0.8)	$cor.rob<br>[1] **-0.1667674**	

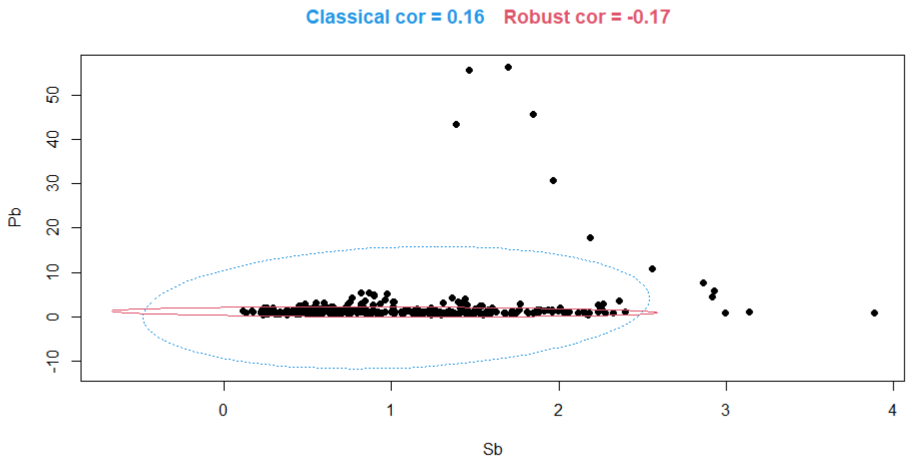

Removing 15 µm of Pb-enriched root surface:

Classical test	Pearson's product-moment correlation<br><br>data: Od533M_CC_wRS$Sb and Od533M_CC_wRS$Pb<br>t = 0.99995, df = 338, **p-value = 0.3181**<br>alternative hypothesis: true correlation is not equal to 0<br>95 percent confidence interval:<br>-0.05235506 0.15974899<br>sample estimates:<br>**cor**<br>**0.05430957**	Spearman's rank correlation rho<br><br>data: Od533M_CC_wRS$Sb and Od533M_CC_wRS$Pb<br>S = 6929224, **p-value = 0.2878**<br>alternative hypothesis: true rho is not equal to 0<br>sample estimates:<br>**rho**<br>**-0.05779828**
MCD (α=0.05; quant=0.8)	$cor.rob<br>[1] **-0.1805594**	

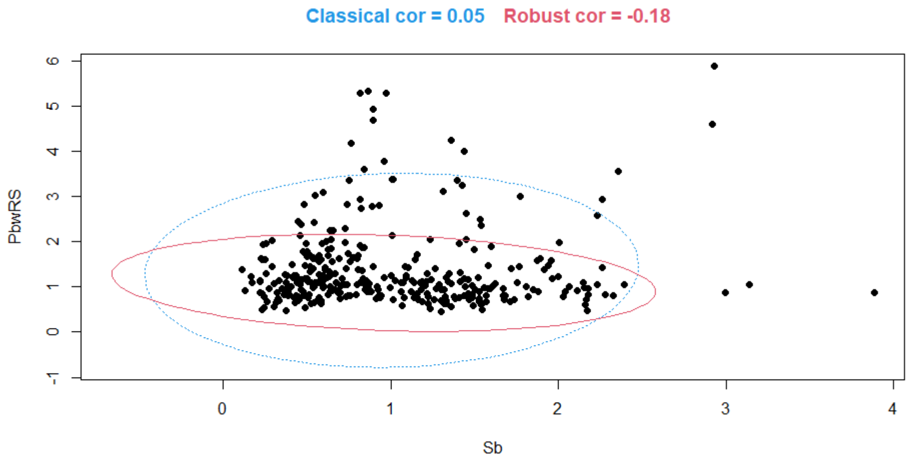

- **Pb vs Sn**

Whole cementum thickness:

Classical test	Pearson's product-moment correlation<br><br>data: Od533M_CC$Sn and Od533M_CC$Pb<br>t = 2.3224, df = 346, **p-value = 0.02079**<br>alternative hypothesis: true correlation is not equal to 0<br>95 percent confidence interval:<br>0.01900917 0.22607892<br>sample estimates:<br>**cor**<br>**0.1238926**	Spearman's rank correlation rho<br><br>data: Od533M_CC$Sn and Od533M_CC$Pb<br>S = 5821180, **p-value = 0.001362**<br>alternative hypothesis: true rho is not equal to 0<br>sample estimates:<br>**rho**<br>**0.1712412**
MCD (α=0.05; quant=0.8)	$cor.rob<br>[1] **0.1078794**	

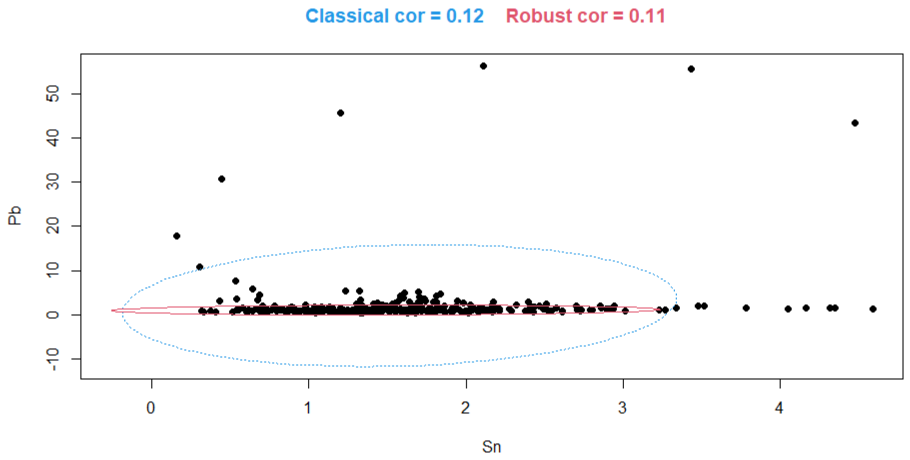

Removing 15 µm of Pb-enriched root surface:

Classical test	Pearson’s product-moment correlation<br><br>data: Od533M_CC_wRS$Sn and Od533M_CC_wRS$Pb<br>t = 1.2308, df = 338, **p-value = 0.2192**<br>alternative hypothesis: true correlation is not equal to 0<br>95 percent confidence interval:<br>-0.03984622 0.17193969<br>sample estimates:<br>**cor**<br>**0.0667991**	Spearman’s rank correlation rho<br><br>data: Od533M_CC_wRS$Sn and Od533M_CC_wRS$Pb<br>S = 5262186, **p-value = 0.0002701**<br>alternative hypothesis: true rho is not equal to 0<br>sample estimates:<br>**rho**<br>**0.1966876**
MCD (α=0.05; quant=0.8)	$cor.rob<br>[1] **0.08510237**	

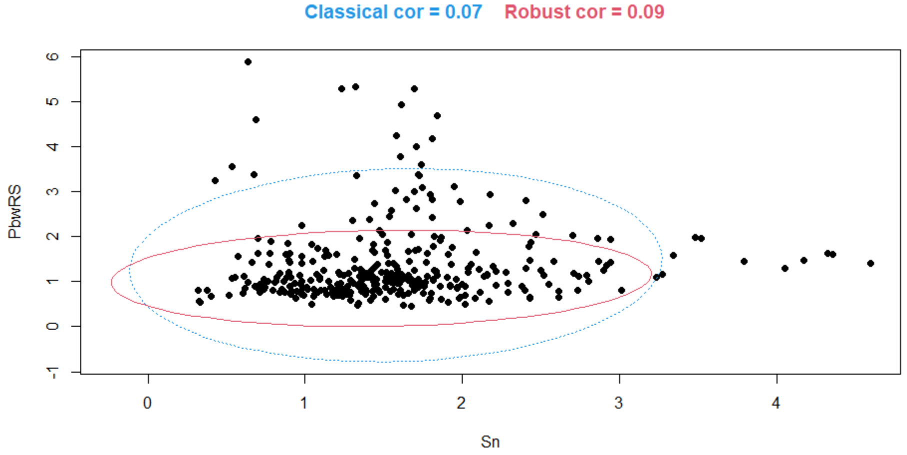

- **Br vs Ba**

Classical test	Pearson's product-moment correlation<br><br>data: Od533M_CC$Ba and Od533M_CC$Br<br>t = -0.030879, df = 346, **p-value = 0.9754**<br>alternative hypothesis: true correlation is not equal to 0<br>95 percent confidence interval:<br>-0.1067725 0.1034890<br>sample estimates:<br>**cor**<br>**-0.001660083**	Spearman's rank correlation rho<br><br>data: Od533M_CC$Ba and Od533M_CC$Br<br>S = 6124820, **p-value = 0.01693**<br>alternative hypothesis: true rho is not equal to 0<br>sample estimates:<br>**rho**<br>**0.1280121**
MCD (α=0.05; quant=0.8)	$cor.rob<br>[1] **0.107534**	

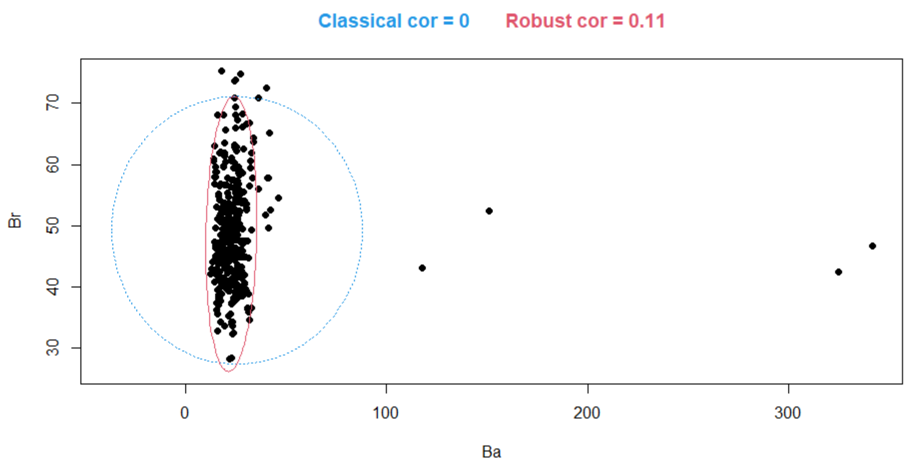

- **Br vs Sr**

Classical test	Pearson's product-moment correlation<br><br>data: Od533M_CC$Sr and Od533M_CC$Br<br>t = 1.1968, df = 346, **p-value = 0.2322**<br>alternative hypothesis: true correlation is not equal to 0<br>95 percent confidence interval:<br>-0.04120406 0.16820073<br>sample estimates:<br>**cor**<br>**0.06420507**	Spearman's rank correlation rho<br><br>data: Od533M_CC$Sr and Od533M_CC$Br<br>S = 6739054, **p-value = 0.4505**<br>alternative hypothesis: true rho is not equal to 0<br>sample estimates:<br>**rho**<br>**0.04056393**
MCD (α=0.05; quant=0.8)	$cor.rob<br>[1] **0.01599081**	

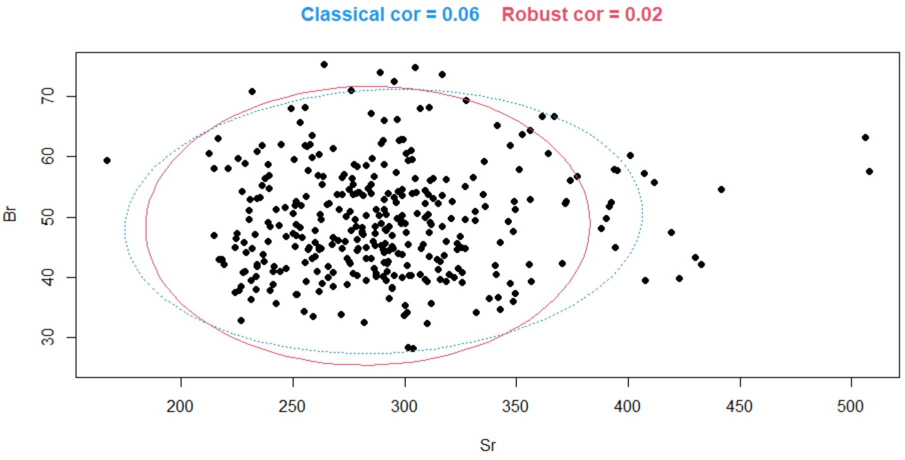

- **Br vs Zn**

Classical test	Pearson's product-moment correlation<br><br>data: Od533M_CC$Zn and Od533M_CC$Br<br>t = 1.2881, df = 346, **p-value = 0.1986**<br>alternative hypothesis: true correlation is not equal to 0<br>95 percent confidence interval:<br>-0.03631413 0.17295552<br>sample estimates:<br>**cor**<br>**0.06908061**	Spearman's rank correlation rho<br><br>data: Od533M_CC$Zn and Od533M_CC$Br<br>S = 6704876, **p-value = 0.398**<br>alternative hypothesis: true rho is not equal to 0<br>sample estimates:<br>**rho**<br>**0.04542984**
MCD (α=0.05; quant=0.8)	$cor.rob<br>[1] **-0.001427677**	

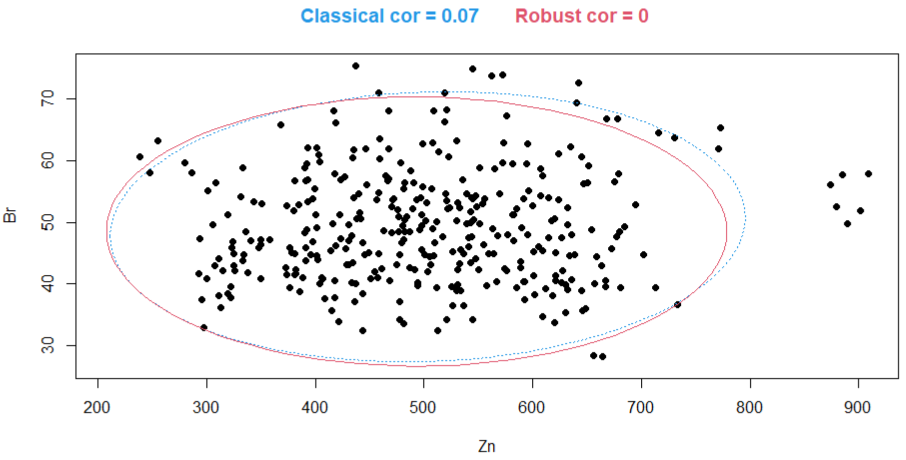

- **Br vs Ca**

Classical test	Pearson's product-moment correlation<br><br>data: Od533M_CC$Ca and Od533M_CC$Br<br>t = -0.45575, df = 346, **p-value = 0.6489**<br>alternative hypothesis: true correlation is not equal to 0<br>95 percent confidence interval:<br>-0.12929178 0.08084547<br>sample estimates:<br>**cor**<br>**-0.02449371**	Spearman's rank correlation rho<br><br>data: Od533M_CC$Ca and Od533M_CC$Br<br>S = 7026418, **p-value = 0.9948**<br>alternative hypothesis: true rho is not equal to 0<br>sample estimates:<br>**rho**<br>**-0.0003479512**
MCD (α=0.05; quant=0.8)	$cor.rob<br>[1] **0.01599081**	

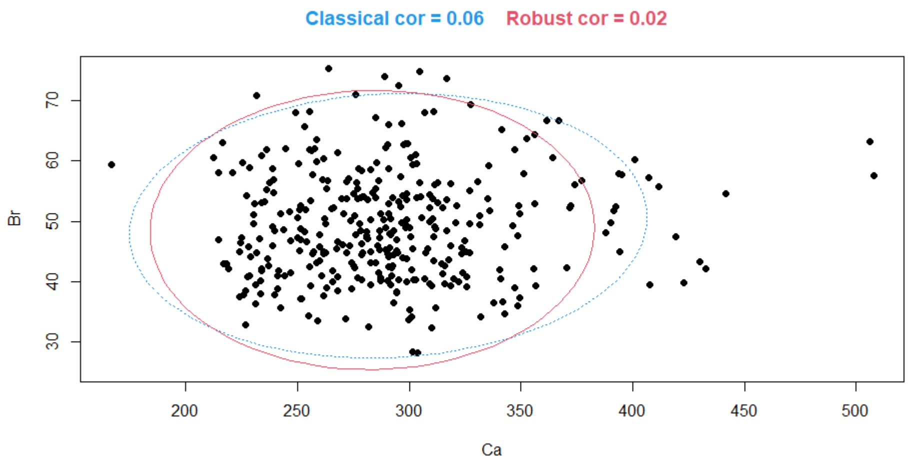

Odense 533M1 – Secondary Dentine (total thickness=396 µm)

Correlations between pairs of elements

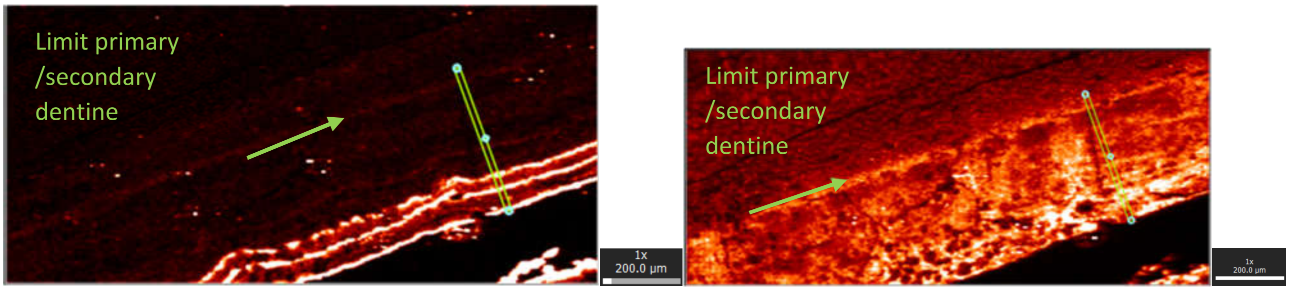

Pb (left) and Zn (right) maps (green path indicates where the measurements are taken)

Summary: whole secondary dentinee thickness, Spearman correlations.

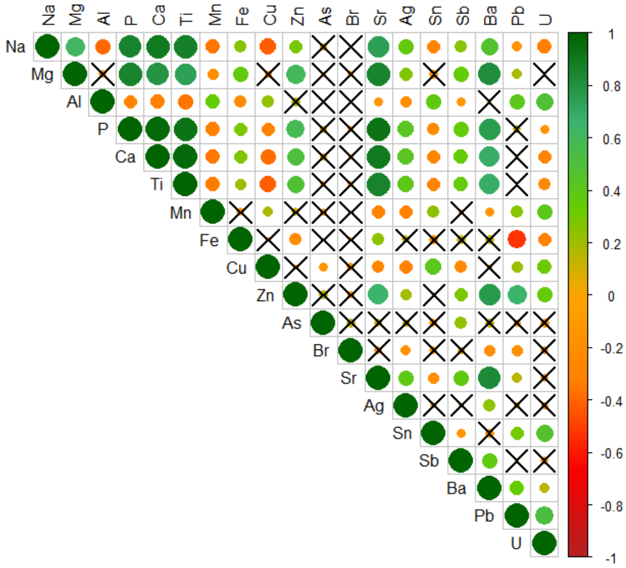

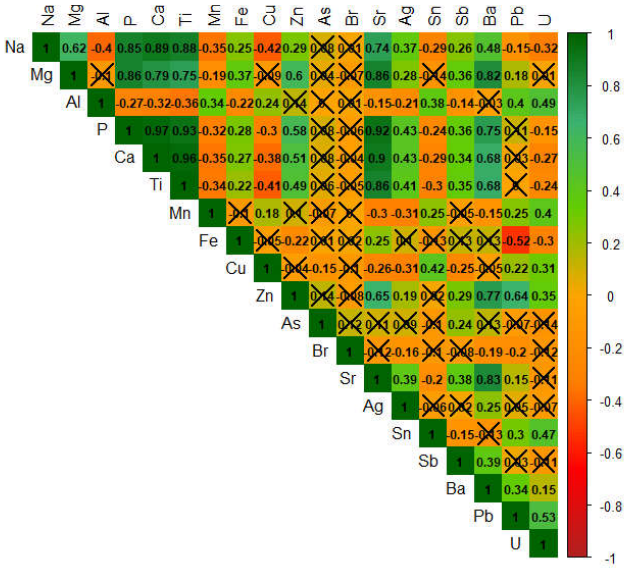

Summary: removing the first 110 µm of secondary dentinee surface, Spearman correlations.

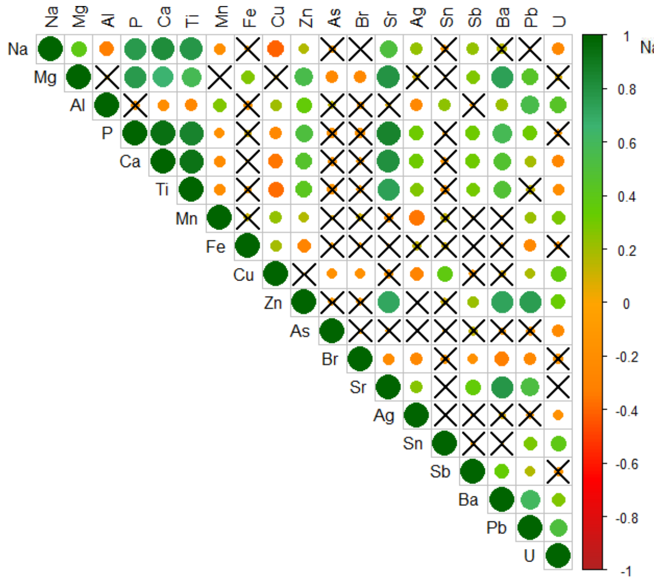

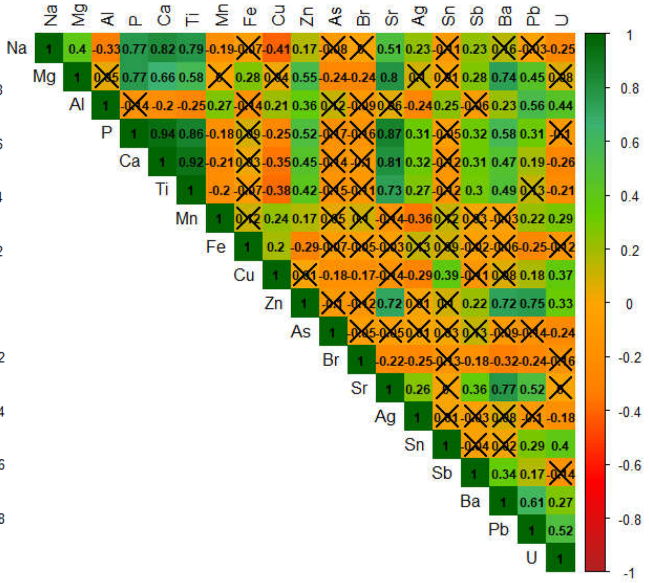

wSDS: without Secondary Dentinee surface [Pb = ~109 µm containing 3 strong (biological) accentuated lines; no significant surface enrichment for Fe, Mn, Cu, Al]

- **Ca vs Ba**

Classical test	Pearson's product-moment correlation<br><br>data: Od533M_SD$Ca and Od533M_SD$Ba<br>t = 21.276, df = 188, **p-value < 2.2e-16**<br>alternative hypothesis: true correlation is not equal to 0<br>95 percent confidence interval:<br>0.7931194 0.8778780<br>sample estimates:<br>**cor**<br>**0.8405697**	Spearman's rank correlation rho<br><br>data: Od533M_SD$Ca and Od533M_SD$Ba<br>S = 361716, **p-value < 2.2e-16**<br>alternative hypothesis: true rho is not equal to 0<br>sample estimates:<br>**rho**<br>**0.6835754**
MCD (α=0.05; quant=0.8)	$cor.rob<br>[1] **0.7271962**	

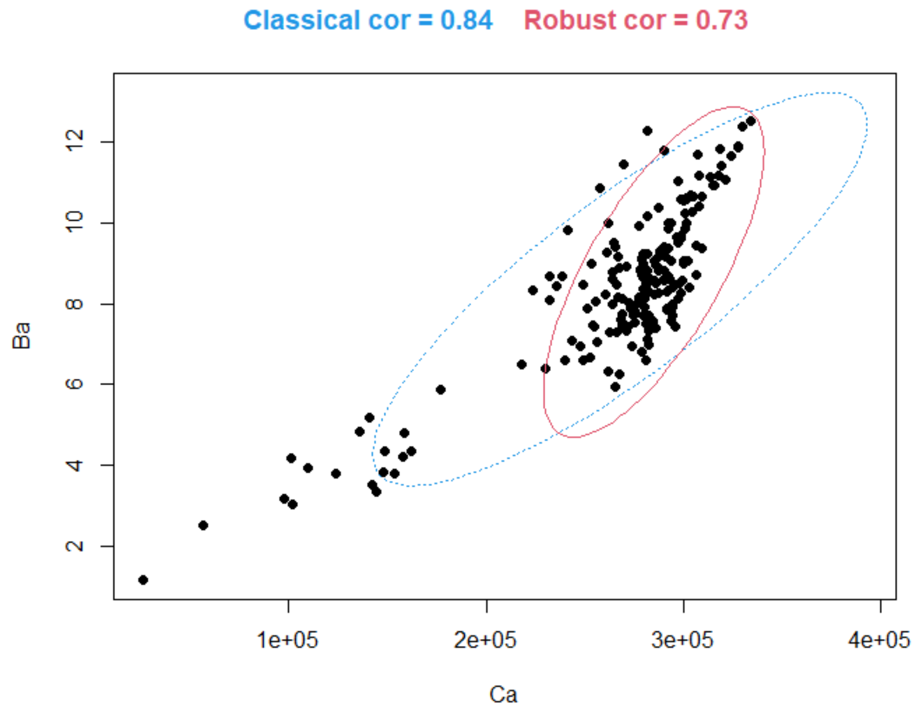

- **Ca vs Sr**

Classical test	Pearson's product-moment correlation<br><br>data: Od533M_SD$Ca and Od533M_SD$Sr<br>t = 47.692, df = 188, **p-value < 2.2e-16**<br>alternative hypothesis: true correlation is not equal to 0<br>95 percent confidence interval:<br>0.9484792 0.9706298<br>sample estimates:<br>**cor**<br>**0.9610697**	Spearman's rank correlation rho<br><br>data: Od533M_SD$Ca and Od533M_SD$Sr<br>S = 111092, **p-value < 2.2e-16**<br>alternative hypothesis: true rho is not equal to 0<br>sample estimates:<br>**rho**<br>**0.9028181**
MCD (α=0.05; quant=0.8)	$cor.rob<br>[1] **0.8939784**	

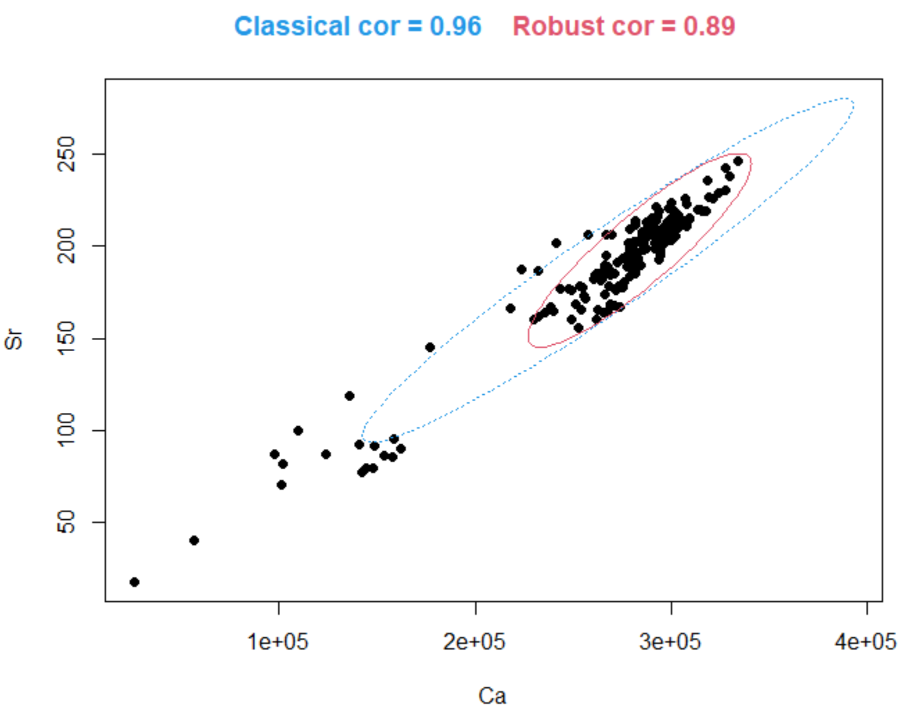

- **Ba vs Sr**

Classical test	Pearson's product-moment correlation<br><br>data: Od533M_SD$Ba and Od533M_SD$Sr<br>t = 28.16, df = 188, **p-value < 2.2e-16**<br>alternative hypothesis: true correlation is not equal to 0<br>95 percent confidence interval:<br>0.8678047 0.9232742<br>sample estimates:<br>**cor**<br>**0.8990891**	Spearman's rank correlation rho<br><br>data: Od533M_SD$Ba and Od533M_SD$Sr<br>S = 197406, **p-value < 2.2e-16**<br>alternative hypothesis: true rho is not equal to 0<br>sample estimates:<br>**rho**<br>**0.8273117**
MCD (α=0.05; quant=0.8)	$cor.rob<br>[1] **0.8067172**	

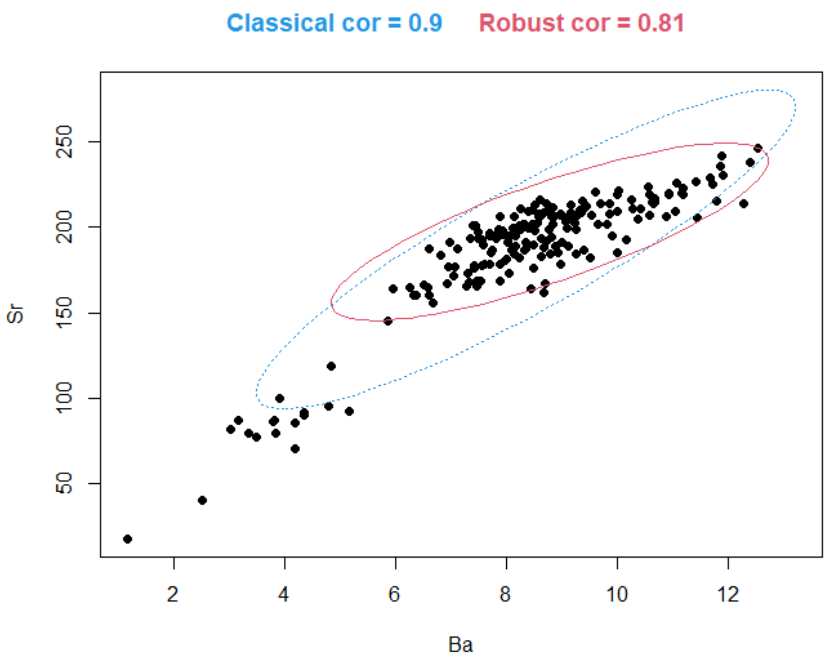

- **Zn vs Sr**

Classical test	Pearson's product-moment correlation<br><br>data: Od533M_SD$Zn and Od533M_SD$Sr<br>t = 9.0389, df = 188, **p-value < 2.2e-16**<br>alternative hypothesis: true correlation is not equal to 0<br>95 percent confidence interval:<br>0.4427294 0.6424145<br>sample estimates:<br>**cor**<br>**0.5503947**	Spearman's rank correlation rho<br><br>data: Od533M_SD$Zn and Od533M_SD$Sr<br>S = 405396, **p-value < 2.2e-16**<br>alternative hypothesis: true rho is not equal to 0<br>sample estimates:<br>**rho**<br>**0.6453647**
MCD (α=0.05; quant=0.8)	$cor.rob<br>[1] **0.7399384**	

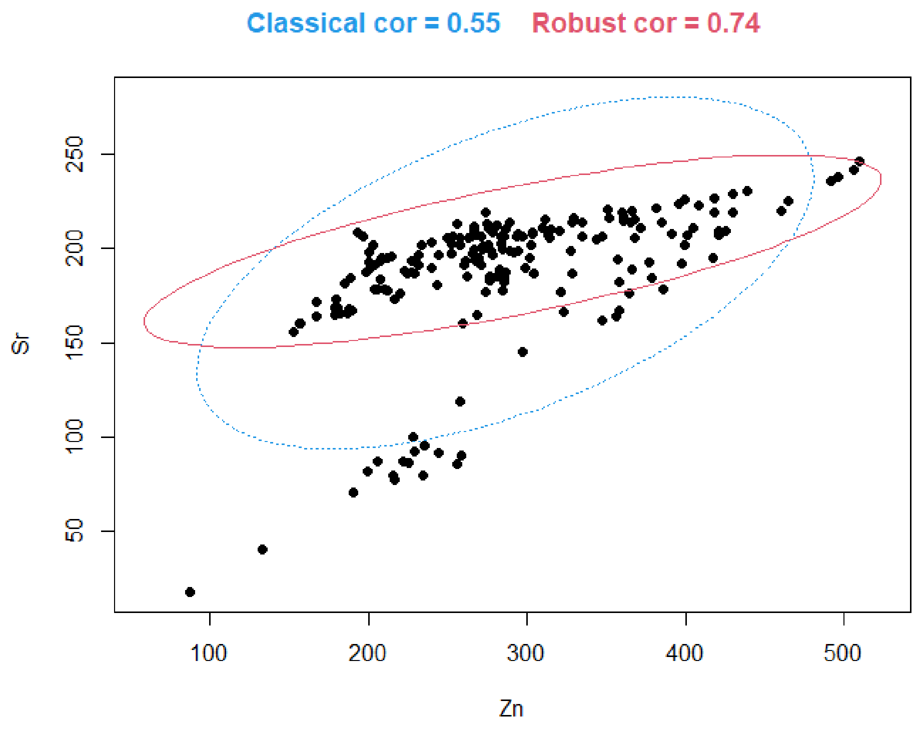

- **Mg vs Sr**

Classical test	Pearson's product-moment correlation<br><br>data: Od533M_SD$Mg and Od533M_SD$Sr<br>t = 39.904, df = 188, **p-value < 2.2e-16**<br>alternative hypothesis: true correlation is not equal to 0<br>95 percent confidence interval:<br>0.9283560 0.9589761<br>sample estimates:<br>**cor**<br>**0.9457272**	Spearman's rank correlation rho<br><br>data: Od533M_SD$Mg and Od533M_SD$Sr<br>S = 160422, **p-value < 2.2e-16**<br>alternative hypothesis: true rho is not equal to 0<br>sample estimates:<br>**rho**<br>**0.8596649**
MCD (α=0.05; quant=0.8)	$cor.rob<br>[1] **0.8330961**	

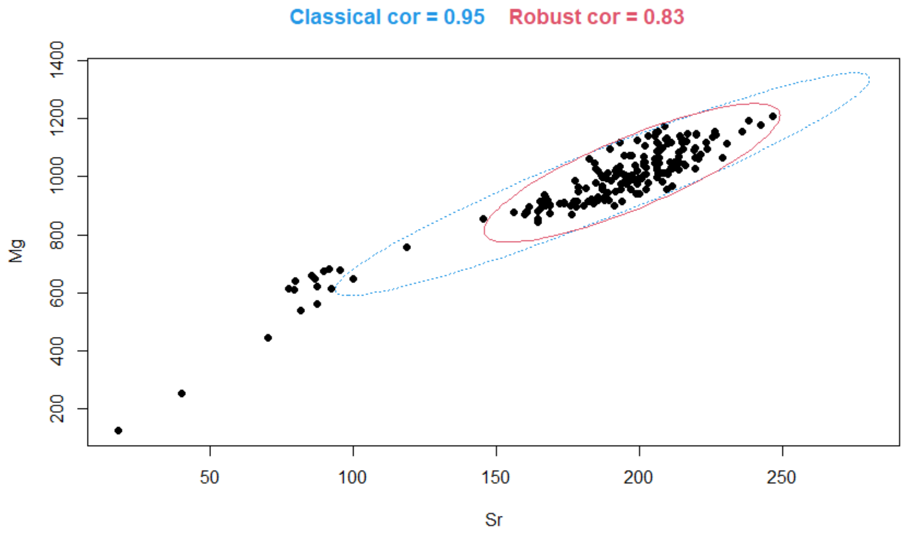

- **Pb vs Sr**

Whole secondary dentinee thickness:

Classical test	Pearson's product-moment correlation<br><br>data: Od533M_SD$Sr and Od533M_SD$Pb<br>t = -3.0443, df = 188, **p-value = 0.002666**<br>alternative hypothesis: true correlation is not equal to 0<br>95 percent confidence interval:<br>-0.34835595 -0.07676657<br>sample estimates:<br>**cor**<br>**-0.2167512**	Spearman's rank correlation rho<br><br>data: Od533M_SD$Sr and Od533M_SD$Pb<br>S = 967754, **p-value = 0.03466**<br>alternative hypothesis: true rho is not equal to 0<br>sample estimates:<br>**rho**<br>**0.1534211**
MCD (α=0.05; quant=0.8)	$cor.rob<br>[1] **0.1233295**	

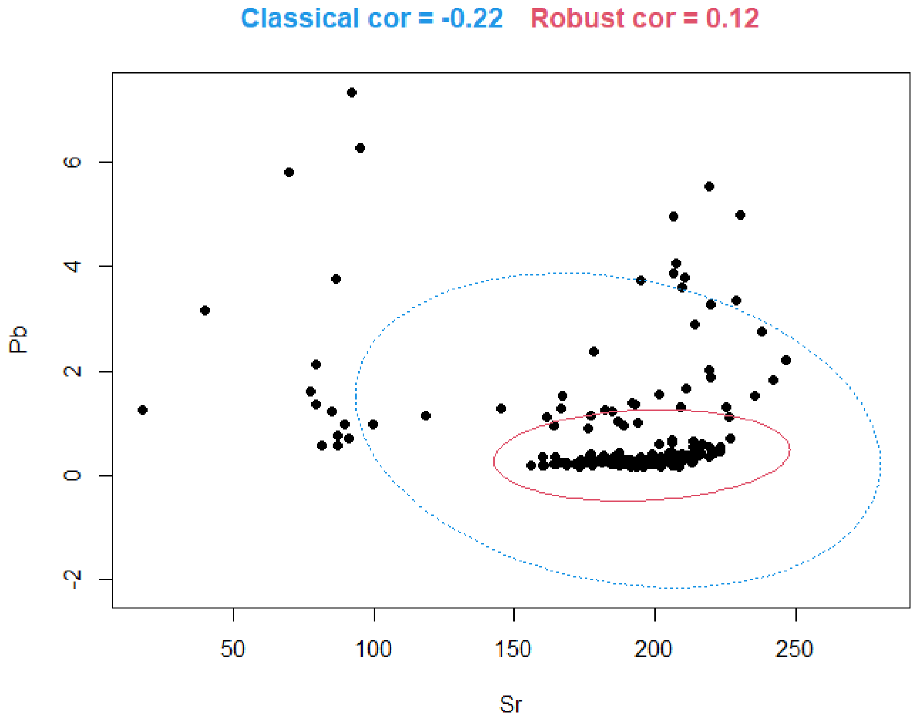

Removing 110 µm of Pb-enriched secondary dentinee surface:

Classical test	Pearson's product-moment correlation<br><br>data: Od533M_SD_wSDS$Sr and Od533M_SD_wSDS$Pb<br>t = 6.8029, df = 135, **p-value = 3.039e-10**<br>alternative hypothesis: true correlation is not equal to 0<br>95 percent confidence interval:<br>0.3688022 0.6204068<br>sample estimates:<br>**cor**<br>**0.5052651**	Spearman's rank correlation rho<br><br>data: Od533M_SD_wSDS$Zn and Od533M_SD_wSDS$Pb<br>S = 106274, **p-value < 2.2e-16**<br>alternative hypothesis: true rho is not equal to 0<br>sample estimates:<br>**rho**<br>**0.7520068**
MCD (α=0.05; quant=0.8)	$cor.rob<br>[1] **0.4864732**	

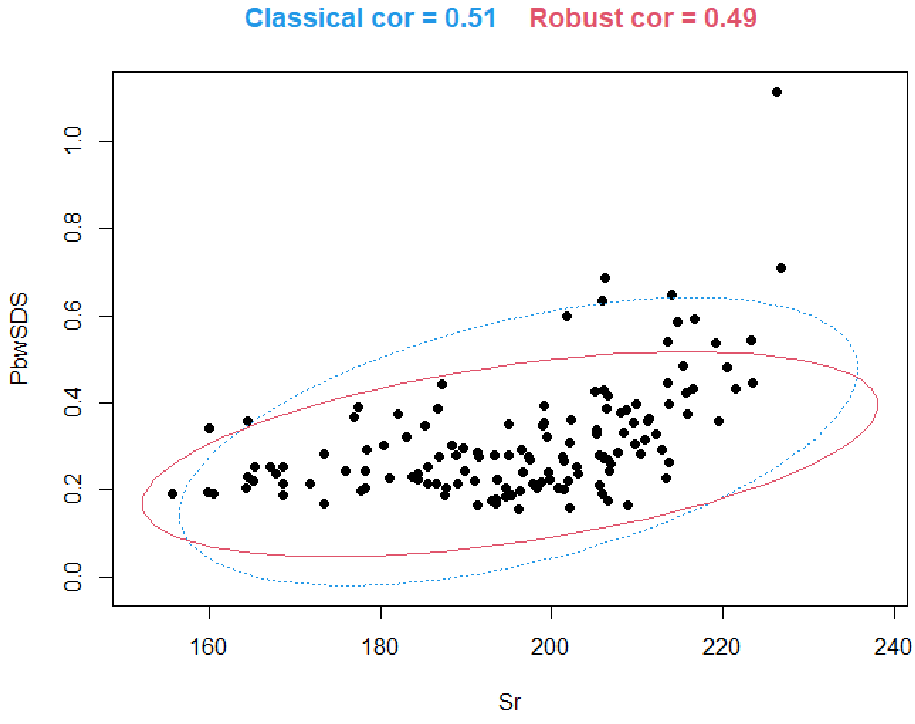

- **Sr vs Zn**

Classical test	Pearson's product-moment correlation<br><br>data: Od533M_SD$Zn and Od533M_SD$Sr<br>t = 9.0389, df = 188, **p-value < 2.2e-16**<br>alternative hypothesis: true correlation is not equal to 0<br>95 percent confidence interval:<br>0.4427294 0.6424145<br>sample estimates:<br>**cor**<br>**0.5503947**	Spearman's rank correlation rho<br><br>data: Od533M_SD$Zn and Od533M_SD$Sr<br>S = 405396, **p-value < 2.2e-16**<br>alternative hypothesis: true rho is not equal to 0<br>sample estimates:<br>**rho**<br>**0.6453647**
MCD (α=0.05; quant=0.8)	$cor.rob<br>[1] **0.7399384**	

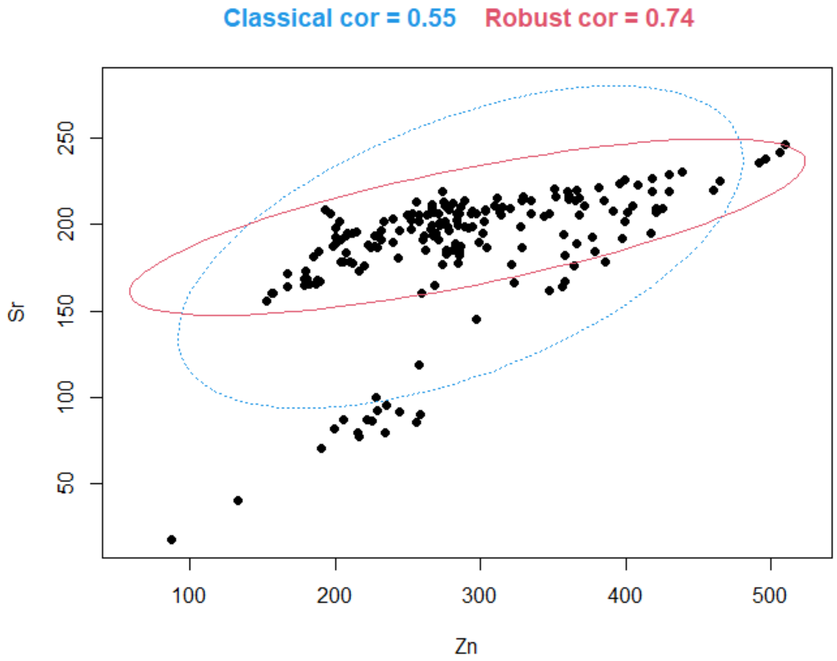

- **Pb vs Zn**

Whole secondary dentinee thickness:

Classical test	Pearson's product-moment correlation<br><br>data: Od533M_SD$Zn and Od533M_SD$Pb<br>t = 5.5906, df = 188, **p-value = 7.874e-08**<br>alternative hypothesis: true correlation is not equal to 0<br>95 percent confidence interval:<br>0.2485614 0.4933909<br>sample estimates:<br>**cor**<br>**0.3775555**	Spearman's rank correlation rho<br><br>data: Od533M_SD$Zn and Od533M_SD$Pb<br>S = 411004, **p-value < 2.2e-16**<br>alternative hypothesis: true rho is not equal to 0<br>sample estimates:<br>**rho**<br>**0.6404589**
MCD (α=0.05; quant=0.8)	$cor.rob<br>[1] **0.6087622**	

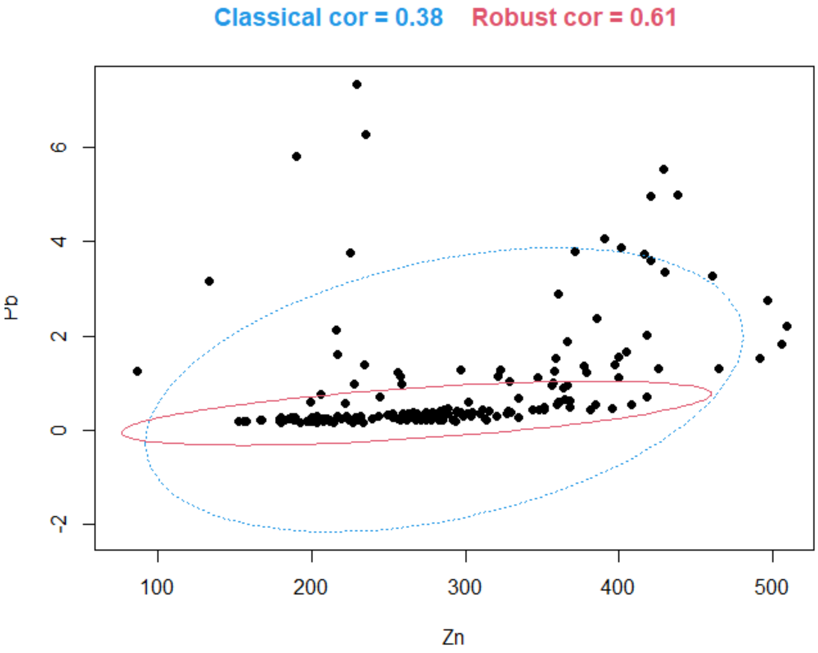

Removing 110 µm of Pb-enriched secondary dentinee surface:

Classical test	Pearson's product-moment correlation<br><br>data: Od533M_SD_wSDS$Zn and Od533M_SD_wSDS$Pb<br>t = 13.47, df = 135, **p-value < 2.2e-16**<br>alternative hypothesis: true correlation is not equal to 0<br>95 percent confidence interval:<br>0.6752670 0.8207112<br>sample estimates:<br>**cor**<br>**0.7572247**	Spearman's rank correlation rho<br><br>data: Od533M_SD_wSDS$Zn and Od533M_SD_wSDS$Pb<br>S = 106274, **p-value < 2.2e-16**<br>alternative hypothesis: true rho is not equal to 0<br>sample estimates:<br>**rho**<br>**0.7520068**
MCD (α=0.05; quant=0.8)	$cor.rob<br>[1] **0.7244165**	

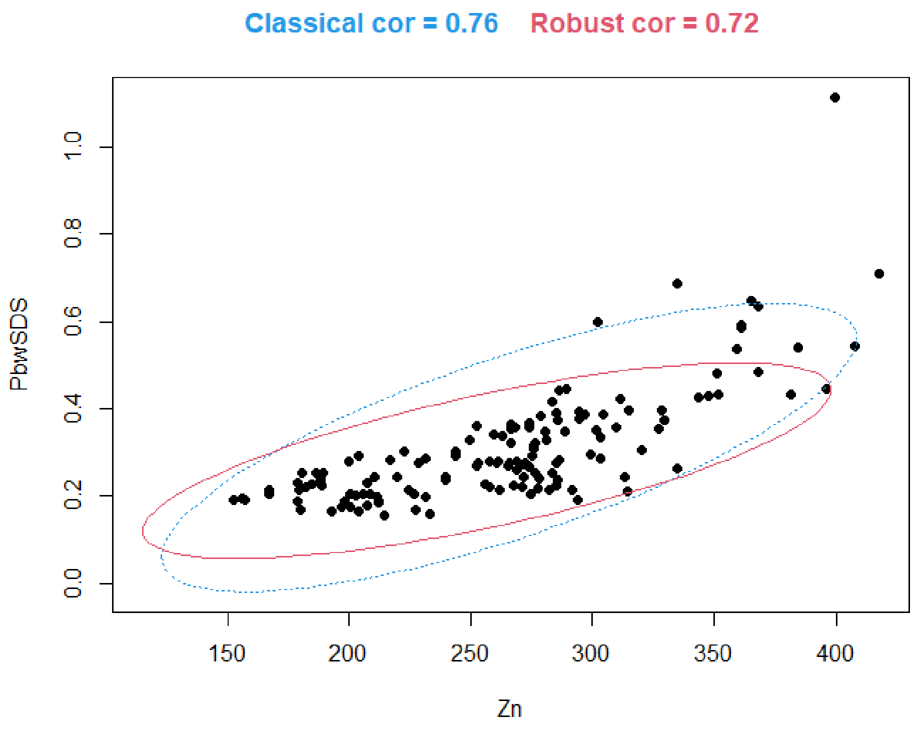

- **Zn vs Ca**

Classical test	Pearson's product-moment correlation<br><br>data: Od533M_SD$Zn and Od533M_SD$Ca<br>t = 7.0081, df = 188, **p-value = 4.196e-11**<br>alternative hypothesis: true correlation is not equal to 0<br>95 percent confidence interval:<br>0.3344273 0.5611145<br>sample estimates:<br>**cor**<br>**0.4551141**	Spearman's rank correlation rho<br><br>data: Od533M_SD$Zn and Od533M_SD$Ca<br>S = 560218, **p-value < 2.2e-16**<br>alternative hypothesis: true rho is not equal to 0<br>sample estimates:<br>**rho**<br>**0.5099284**
MCD (α=0.05; quant=0.8)	$cor.rob<br>[1] **0.6601464**	

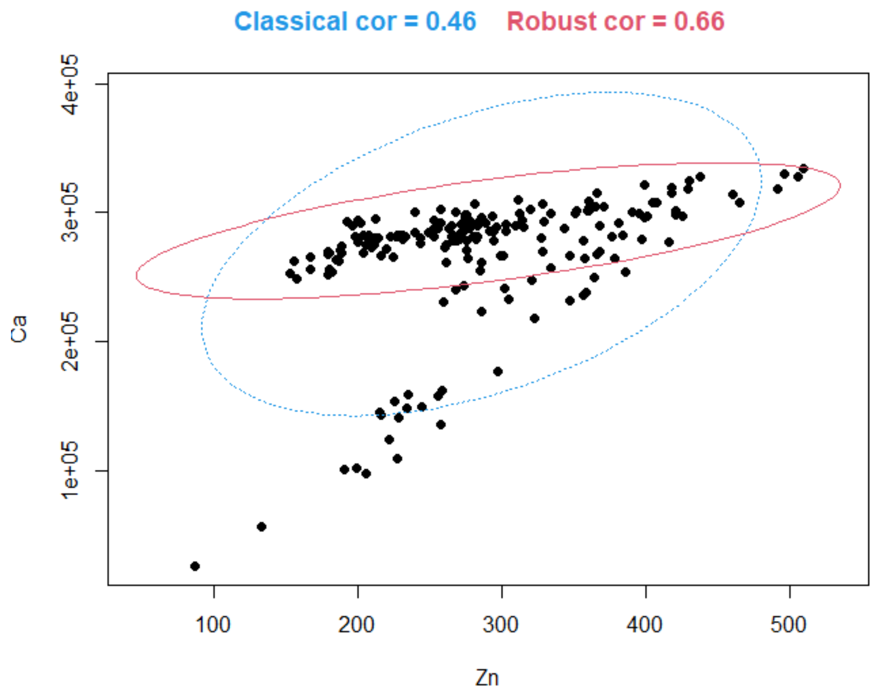

- **Zn vs Fe**

Classical test	Pearson's product-moment correlation<br><br>data: Od533M_SD$Zn and Od533M_SD$Fe<br>t = -2.455, df = 188, **p-value = 0.015**<br>alternative hypothesis: true correlation is not equal to 0<br>95 percent confidence interval:<br>-0.31080458 -0.03476824<br>sample estimates:<br>**cor**<br>**-0.1762492**	Spearman's rank correlation rho<br><br>data: Od533M_SD$Zn and Od533M_SD$Fe<br>S = 1399172, **p-value = 0.001936**<br>alternative hypothesis: true rho is not equal to 0<br>sample estimates:<br>**rho**<br>**-0.2239779**
MCD (α=0.05; quant=0.8)	$cor.rob<br>[1] **-0.3170197**	

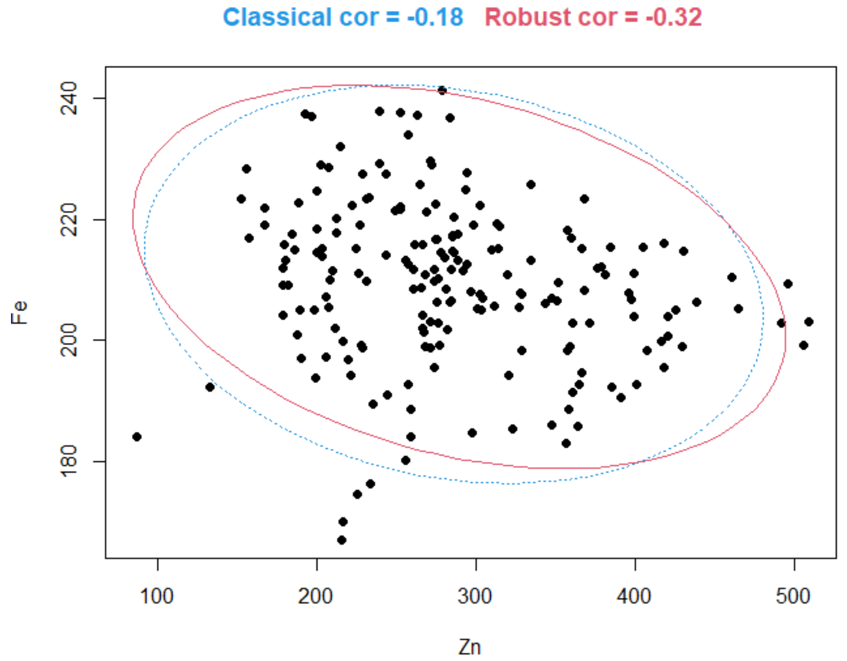

- **Zn vs Cu**

Classical test	Pearson's product-moment correlation<br><br>data: Od533M_SD$Zn and Od533M_SD$Cu<br>t = -1.4594, df = 188, **p-value = 0.1461**<br>alternative hypothesis: true correlation is not equal to 0<br>95 percent confidence interval:<br>-0.24450631 0.03707514<br>sample estimates:<br>**cor**<br>**-0.1058368**	Spearman's rank correlation rho<br><br>data: Od533M_SD$Zn and Od533M_SD$Cu<br>S = 1193286, **p-value = 0.5475**<br>alternative hypothesis: true rho is not equal to 0<br>sample estimates:<br>**rho**<br>**-0.04387146**
MCD (α=0.05; quant=0.8)	$cor.rob<br>[1] **0.009811241**	

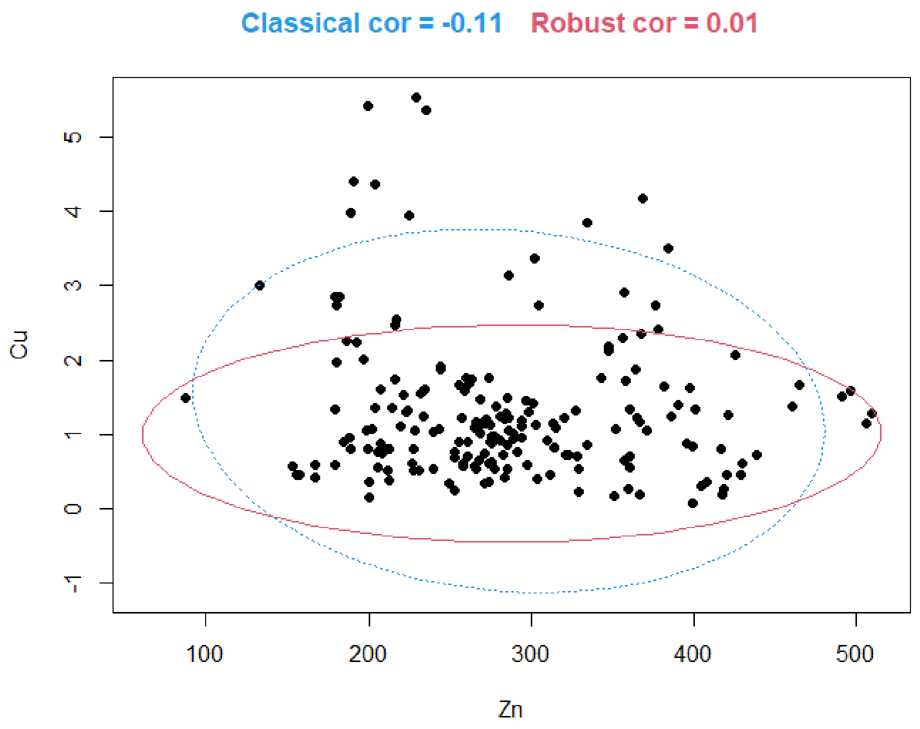

- **Zn vs Mg**

Classical test	Pearson's product-moment correlation<br><br>data: Od533M_SD$Zn and Od533M_SD$Mg<br>t = 9.3848, df = 188, **p-value < 2.2e-16**<br>alternative hypothesis: true correlation is not equal to 0<br>95 percent confidence interval:<br>0.4594057 0.6545460<br>sample estimates:<br>**cor**<br>**0.5648209**	Spearman's rank correlation rho<br><br>data: Od533M_SD$Zn and Od533M_SD$Mg<br>S = 457680, **p-value < 2.2e-16**<br>alternative hypothesis: true rho is not equal to 0<br>sample estimates:<br>**rho**<br>**0.5996273**
MCD (α=0.05; quant=0.8)	$cor.rob<br>[1] **0.6538013**	

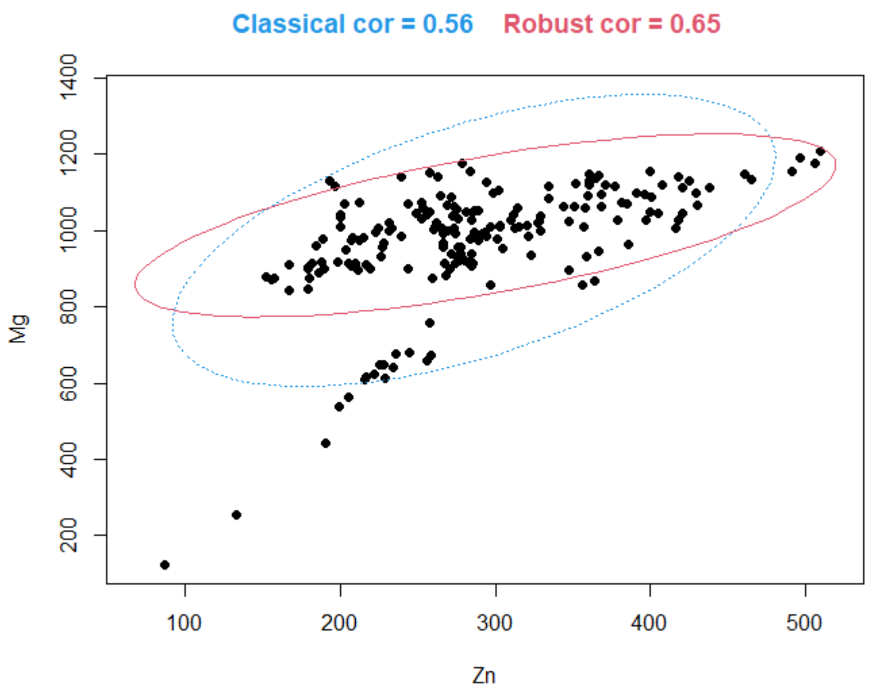

- **Ca vs Fe**

Classical test	Pearson's product-moment correlation<br><br>data: Od533M_SD$Ca and Od533M_SD$Fe<br>t = 8.0258, df = 188, **p-value = 1.063e-13**<br>alternative hypothesis: true correlation is not equal to 0<br>95 percent confidence interval:<br>0.3909232 0.6040775<br>sample estimates:<br>**cor**<br>**0.5051645**	Spearman's rank correlation rho<br><br>data: Od533M_SD$Ca and Od533M_SD$Fe<br>S = 832466, **p-value = 0.0001572**<br>alternative hypothesis: true rho is not equal to 0<br>sample estimates:<br>**rho**<br>**0.2717693**
MCD (α=0.05; quant=0.8)	$cor.rob<br>[1] **-0.05379779**	

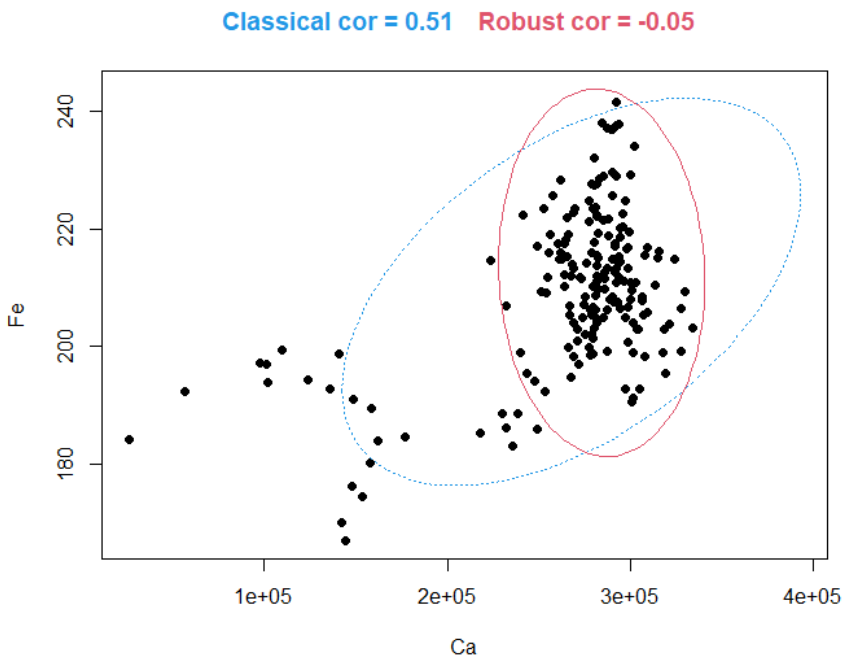

- **Ca vs Mg**

Classical test	Pearson's product-moment correlation<br><br>data: Od533M_SD$Ca and Od533M_SD$Mg<br>t = 31.118, df = 188, **p-value < 2.2e-16**<br>alternative hypothesis: true correlation is not equal to 0<br>95 percent confidence interval:<br>0.8884944 0.9355818<br>sample estimates:<br>**cor**<br>**0.9151051**	Spearman's rank correlation rho<br><br>data: Od533M_SD$Ca and Od533M_SD$Mg<br>S = 236192, **p-value < 2.2e-16**<br>alternative hypothesis: true rho is not equal to 0<br>sample estimates:<br>**rho**<br>**0.7933822**
MCD (α=0.05; quant=0.8)	$cor.rob<br>[1] **0.7510065**	

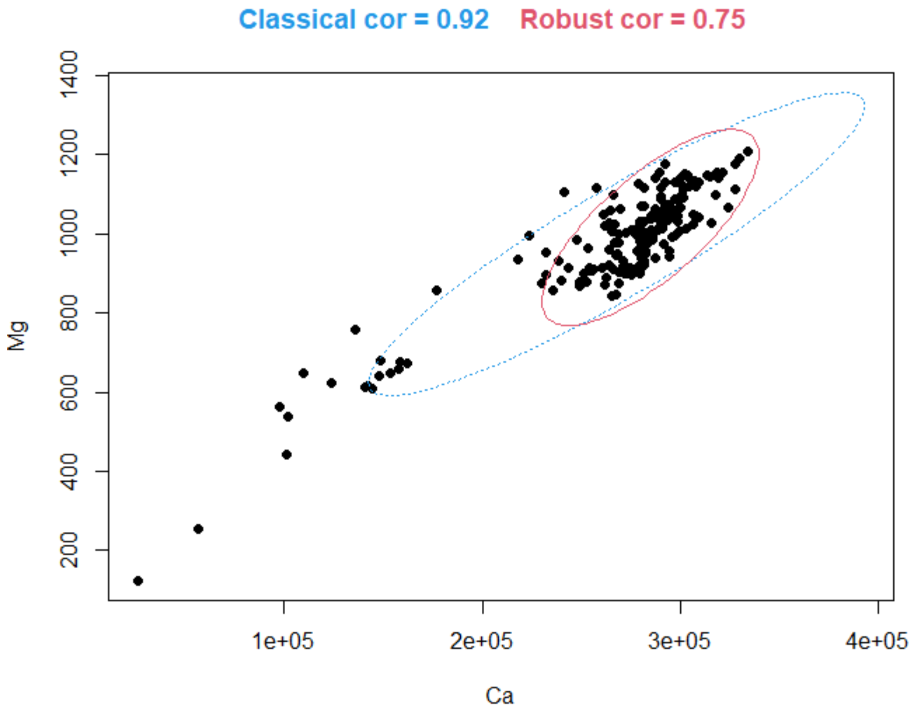

- **Ca vs P**

Classical test	Pearson's product-moment correlation<br><br>data: Od533M_SD$Ca and Od533M_SD$P<br>t = 113.46, df = 188, **p-value < 2.2e-16**<br>alternative hypothesis: true correlation is not equal to 0<br>95 percent confidence interval:<br>0.9903906 0.9945722<br>sample estimates:<br>**cor**<br>**0.9927769**	Spearman's rank correlation rho<br><br>data: Od533M_SD$Ca and Od533M_SD$P<br>S = 36556, **p-value < 2.2e-16**<br>alternative hypothesis: true rho is not equal to 0<br>sample estimates:<br>**rho**<br>**0.9680213**
MCD (α=0.05; quant=0.8)	$cor.rob<br>[1] **0.9609519**	

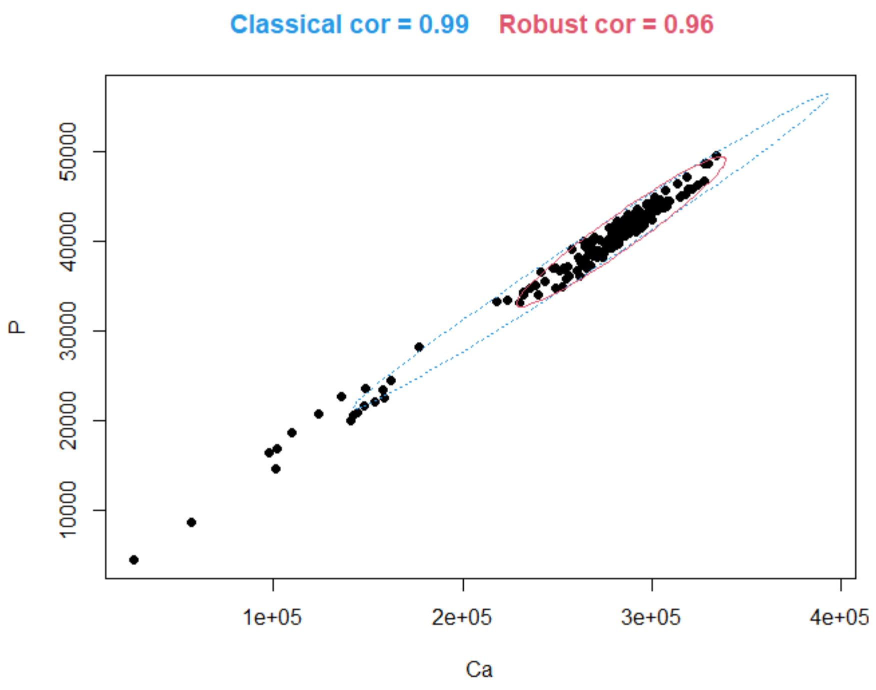

- **Cu vs Fe**

Classical test	Pearson's product-moment correlation<br><br>data: Od533M_SD$Cu and Od533M_SD$Fe<br>t = -0.99885, df = 188, **p-value = 0.3192**<br>alternative hypothesis: true correlation is not equal to 0<br>95 percent confidence interval:<br>-0.21280835 0.07042566<br>sample estimates:<br>**cor**<br>**-0.07265607**	Spearman's rank correlation rho<br><br>data: Od533M_SD$Cu and Od533M_SD$Fe<br>S = 1199798, **p-value = 0.4967**<br>alternative hypothesis: true rho is not equal to 0<br>sample estimates:<br>**rho**<br>**-0.04956807**
MCD (α=0.05; quant=0.8)	$cor.rob<br>[1] **-0.1037514**	

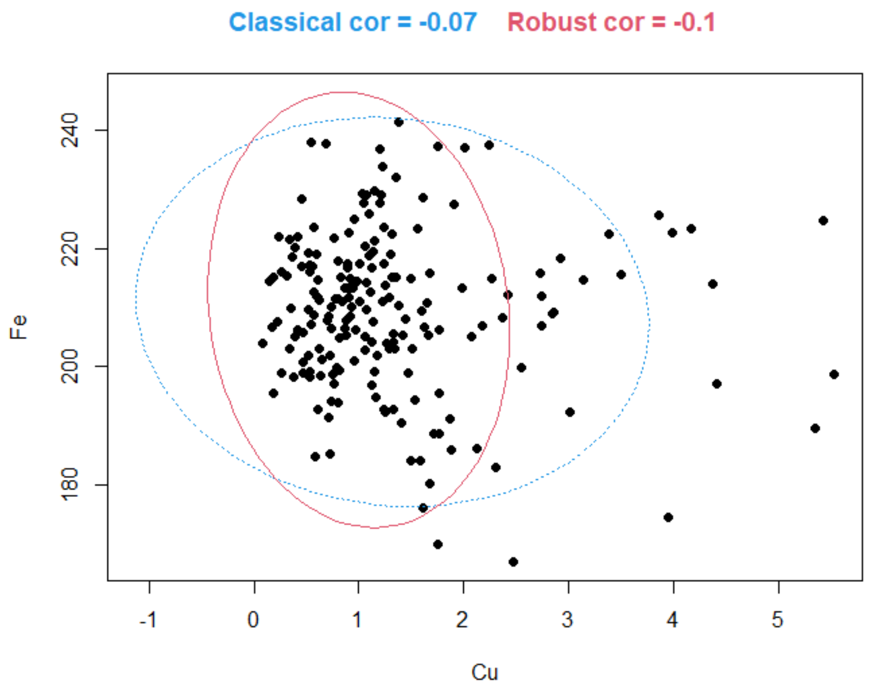

- **Mg vs Fe**

Classical test	Pearson's product-moment correlation<br><br>data: Od533M_SD$Mg and Od533M_SD$Fe<br>t = 8.243, df = 188, **p-value = 2.823e-14**<br>alternative hypothesis: true correlation is not equal to 0<br>95 percent confidence interval:<br>0.4024041 0.6126594<br>sample estimates:<br>**cor**<br>**0.5152425**	Spearman's rank correlation rho<br><br>data: Od533M_SD$Mg and Od533M_SD$Fe<br>S = 717378, **p-value = 1.513e-07**<br>alternative hypothesis: true rho is not equal to 0<br>sample estimates:<br>**rho**<br>**0.3724468**
MCD (α=0.05; quant=0.8)	$cor.rob<br>[1] **0.2092387**	

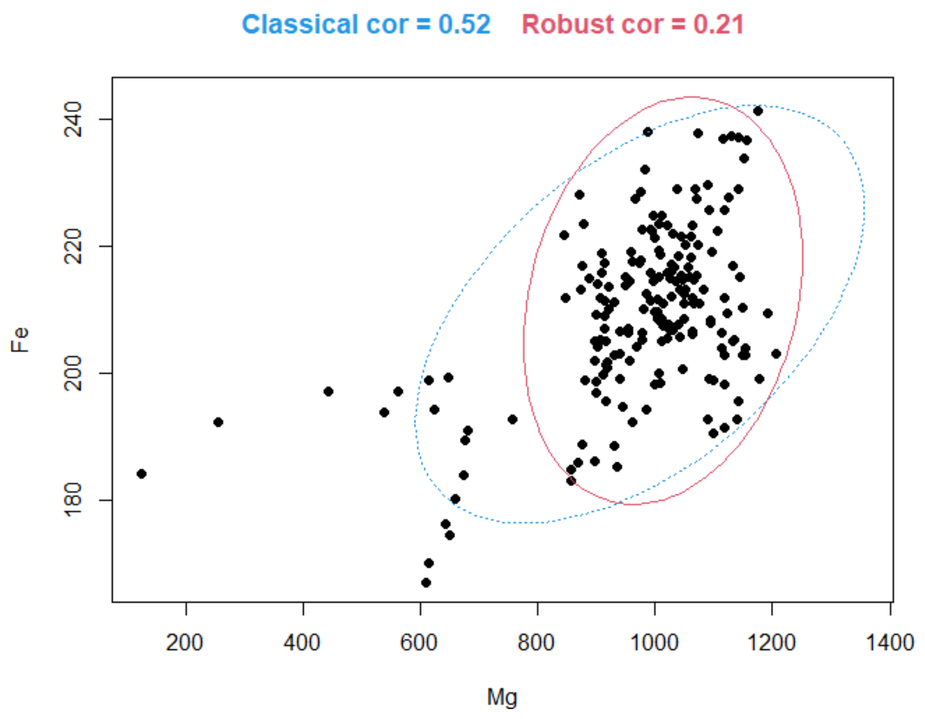

- **Cu vs Mg**

Classical test	Pearson's product-moment correlation<br><br>data: Od533M_SD$Mg and Od533M_SD$Cu<br>t = -3.5432, df = 188, **p-value = 0.0004986**<br>alternative hypothesis: true correlation is not equal to 0<br>95 percent confidence interval:<br>-0.3790493 -0.1118258<br>sample estimates:<br>**cor**<br>**-0.2501963**	Spearman's rank correlation rho<br><br>data: Od533M_SD$Mg and Od533M_SD$Cu<br>S = 1249146, **p-value = 0.203**<br>alternative hypothesis: true rho is not equal to 0<br>sample estimates:<br>**rho**<br>**-0.09273708**
MCD (α=0.05; quant=0.8)	$cor.rob<br>[1] **0.08609909**	

Classical cor = -0.25 Robust cor = 0.09

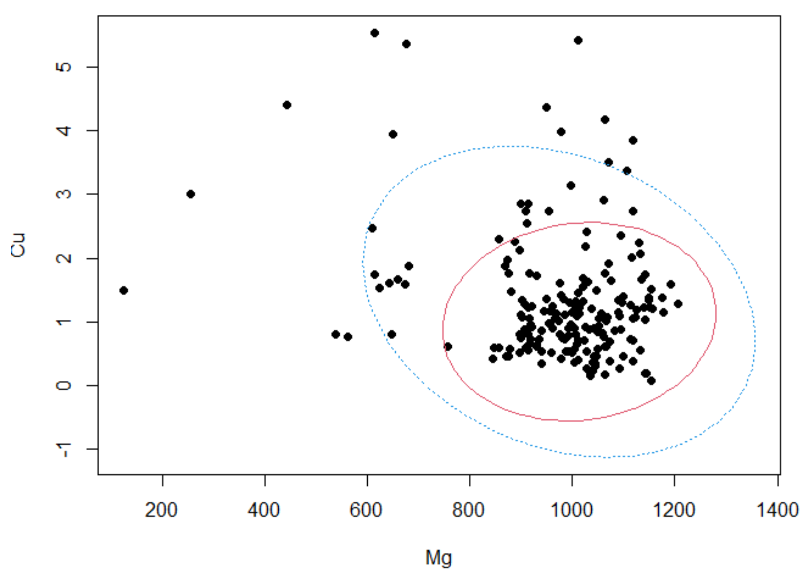

- **As vs Hg, Pb vs Hg (no Hg at all). => Not Applicable.**

- **Pb vs As**

Whole secondary dentinee thickness:

Classical test	Pearson's product-moment correlation<br><br>data: Od533M_SD$As and Od533M_SD$Pb<br>t = -0.57847, df = 188, **p-value = 0.5636**<br>alternative hypothesis: true correlation is not equal to 0<br>95 percent confidence interval:<br>-0.1834048 0.1008063<br>sample estimates:<br>**cor**<br>**-0.04215196**	Spearman's rank correlation rho<br><br>data: Od533M_SD$As and Od533M_SD$Pb<br>S = 1228634, **p-value = 0.3048**<br>alternative hypothesis: true rho is not equal to 0<br>sample estimates:<br>**rho**<br>**-0.07479344**
MCD (α=0.05; quant=0.8)	$cor.rob<br>[1] **-0.05585069**	

Classical cor = -0.04 Robust cor = -0.06

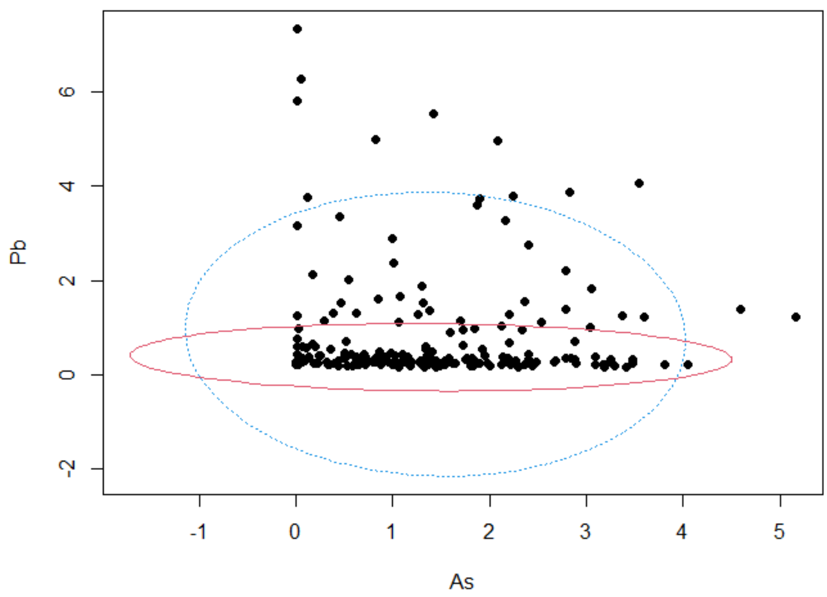

Removing 110 µm of Pb-enriched secondary dentinee surface:

Classical test	Pearson's product-moment correlation<br><br>data: Od533M_SD_wSDS$As and Od533M_SD_wSDS$Pb<br>t = -0.79052, df = 135, **p-value = 0.4306**<br>alternative hypothesis: true correlation is not equal to 0<br>95 percent confidence interval:<br>-0.2329440 0.1009847<br>sample estimates:<br>**cor**<br>**-0.0678805**	Spearman's rank correlation rho<br><br>data: Od533M_SD_wSDS$As and Od533M_SD_wSDS$Pb<br>S = 490286, **p-value = 0.09296**<br>alternative hypothesis: true rho is not equal to 0<br>sample estimates:<br>**rho**<br>**-0.1440952**
MCD (α=0.05; quant=0.8)	$cor.rob<br>[1] **-0.1582813**	

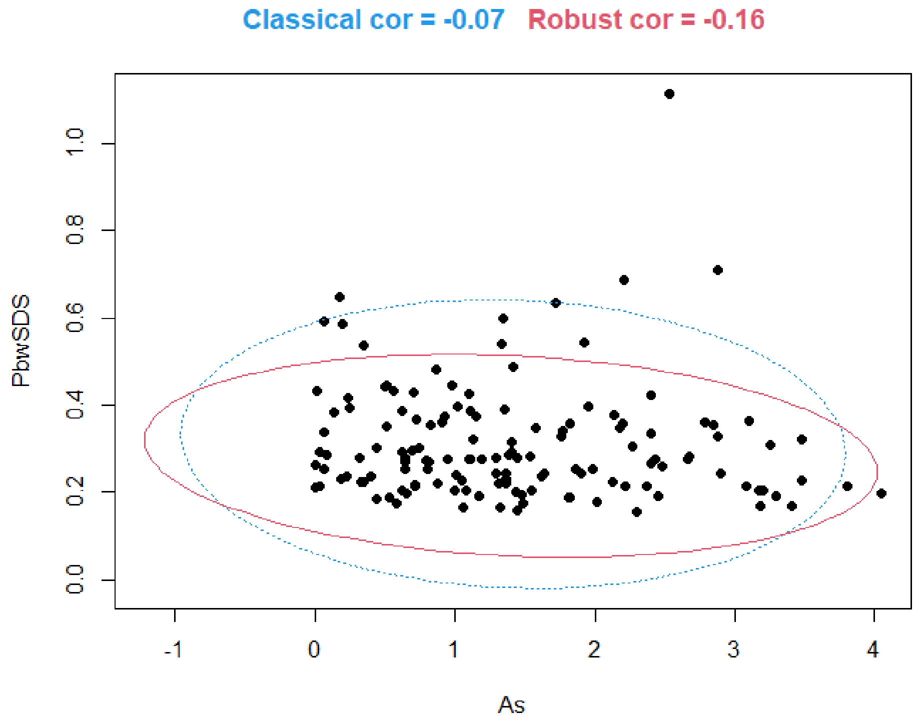

- **Sb vs Sn**

Classical test	Pearson's product-moment correlation<br><br>data: Od533M_SD$Sn and Od533M_SD$Sb<br>t = -3.4518, df = 188, **p-value = 0.0006878**<br>alternative hypothesis: true correlation is not equal to 0<br>95 percent confidence interval:<br>-0.3735037 -0.1054417<br>sample estimates:<br>**cor**<br>**-0.2441307**	Spearman's rank correlation rho<br><br>data: Od533M_SD$Sn and Od533M_SD$Sb<br>S = 1315916, **p-value = 0.03746**<br>alternative hypothesis: true rho is not equal to 0<br>sample estimates:<br>**rho**<br>**-0.1511466**
MCD (α=0.05; quant=0.8)	$cor.rob<br>[1] **0.023168**	

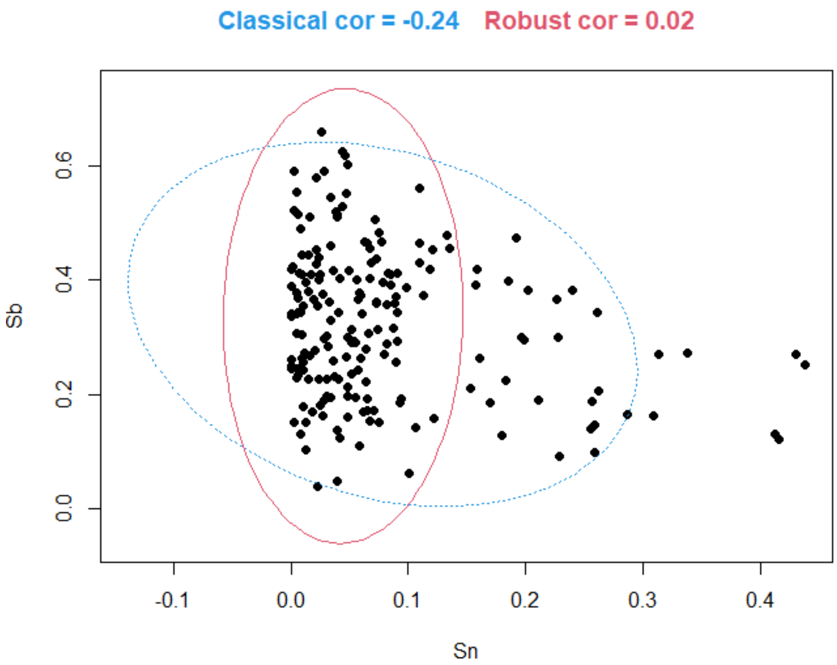

- **Pb vs Sb**

Whole secondary dentinee thickness:

	Pearson's product-moment correlation	Spearman's rank correlation rho
Classical test	data: Od533M_SD$Sb and Od533M_SD$Pb<br>t = -0.93918, df = 188, **p-value = 0.3488**<br>alternative hypothesis: true correlation is not equal to 0<br>95 percent confidence interval:<br>-0.20866014 0.07474378<br>sample estimates:<br>**cor**<br>**-0.06833665**	data: Od533M_SD$Sb and Od533M_SD$Pb<br>S = 1110492, **p-value = 0.6955**<br>alternative hypothesis: true rho is not equal to 0<br>sample estimates:<br>**rho**<br>**0.02855568**
MCD (α=0.05; quant=0.8)	$cor.rob<br>[1] **0.03371059**	

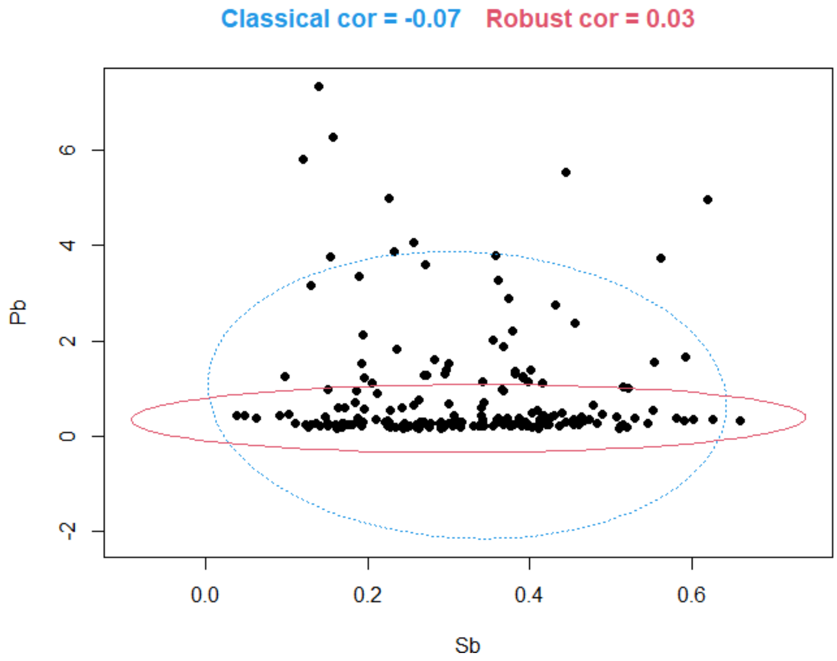

Removing 110 µm of Pb-enriched secondary dentinee surface:

Classical test	Pearson's product-moment correlation<br><br>data: Od533M_SD_wSDS$Sb and Od533M_SD_wSDS$Pb<br>t = 1.284, df = 135, **p-value = 0.2013**<br>alternative hypothesis: true correlation is not equal to 0<br>95 percent confidence interval:<br>-0.05896091 0.27253560<br>sample estimates:<br>**cor**<br>**0.1098408**	Spearman's rank correlation rho<br><br>data: Od533M_SD_wSDS$Sb and Od533M_SD_wSDS$Pb<br>S = 356020, **p-value = 0.04817**<br>alternative hypothesis: true rho is not equal to 0<br>sample estimates:<br>**rho**<br>**0.169218**
MCD (α=0.05; quant=0.8)	$cor.rob<br>[1] **0.340878**	

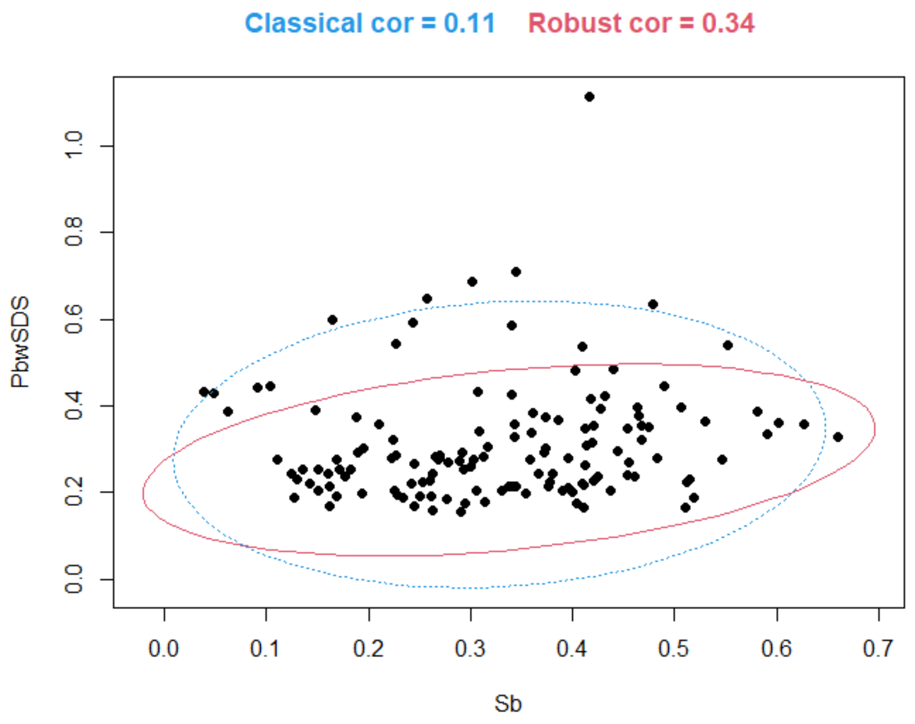

- **Pb vs Sn**

Whole secondary dentinee thickness:

Classical test	Pearson's product-moment correlation<br><br>data: Od533M_SD$Sn and Od533M_SD$Pb<br>t = 2.8353, df = 188, **p-value = 0.005079**<br>alternative hypothesis: true correlation is not equal to 0<br>95 percent confidence interval:<br>0.06193476 0.33519342<br>sample estimates:<br>**cor**<br>**0.2025027**	Spearman's rank correlation rho<br><br>data: Od533M_SD$Sn and Od533M_SD$Pb<br>S = 805144, **p-value = 3.737e-05**<br>alternative hypothesis: true rho is not equal to 0<br>sample estimates:<br>**rho**<br>**0.2956702**
MCD (α=0.05; quant=0.8)	$cor.rob<br>[1] **0.424453**	

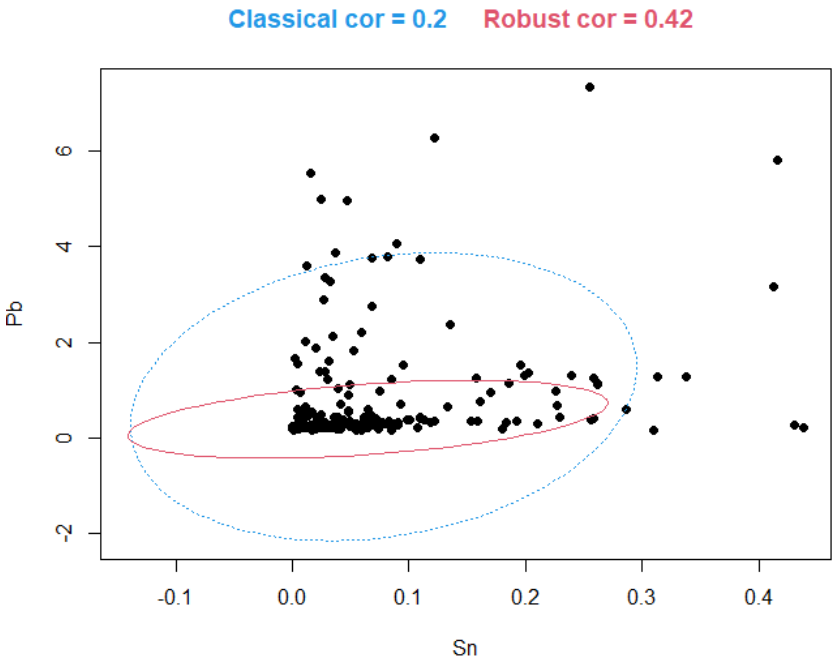

Removing 110 µm of Pb-enriched secondary dentinee surface:

	Pearson's product-moment correlation	Spearman's rank correlation rho
Classical test	data: Od533M_SD_wSDS$Sn and Od533M_SD_wSDS$Pb<br>t = 1.5529, df = 135, **p-value = 0.1228**<br>alternative hypothesis: true correlation is not equal to 0<br>95 percent confidence interval:<br>-0.0360418 0.2936653<br>sample estimates:<br>**cor**<br>**0.1324745**	data: Od533M_SD_wSDS$Sn and Od533M_SD_wSDS$Pb<br>S = 304920, **p-value = 0.0006611**<br>alternative hypothesis: true rho is not equal to 0<br>sample estimates:<br>**rho**<br>**0.2884612**
MCD (α=0.05; quant=0.8)	$cor.rob<br>[1] **0.3515933**	

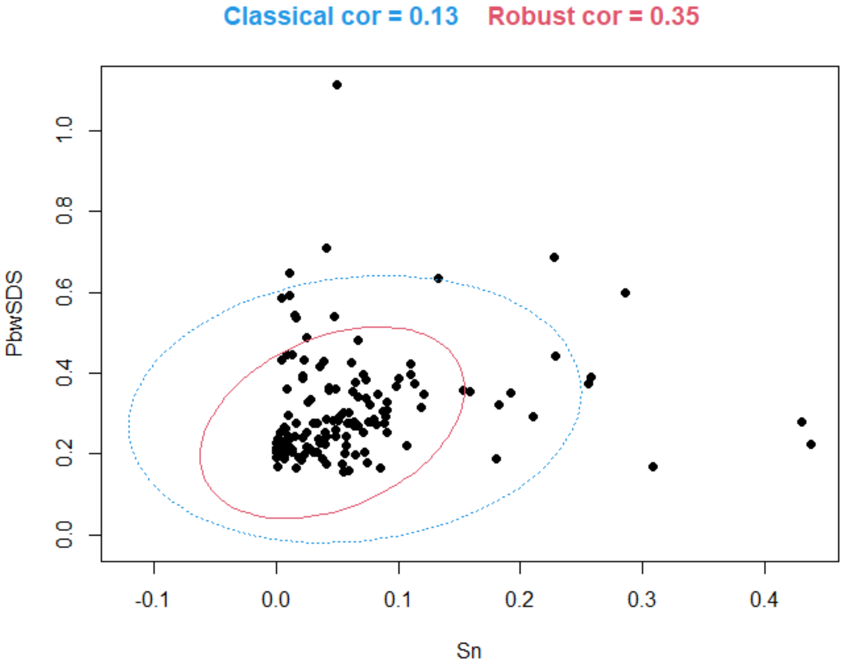

- **Br vs Ba**

Classical test	Pearson's product-moment correlation<br><br>data: Od533M_SD$Ba and Od533M_SD$Br<br>t = -1.6732, df = 188, **p-value = 0.09595**<br>alternative hypothesis: true correlation is not equal to 0<br>95 percent confidence interval:<br>-0.2590202 0.0215923<br>sample estimates:<br>**cor**<br>**-0.1211334**	Spearman's rank correlation rho<br><br>data: Od533M_SD$Ba and Od533M_SD$Br<br>S = 1364608, **p-value = 0.007479**<br>alternative hypothesis: true rho is not equal to 0<br>sample estimates:<br>**rho**<br>**-0.1937418**
MCD (α=0.05; quant=0.8)	$cor.rob<br>[1] **-0.3389472**	

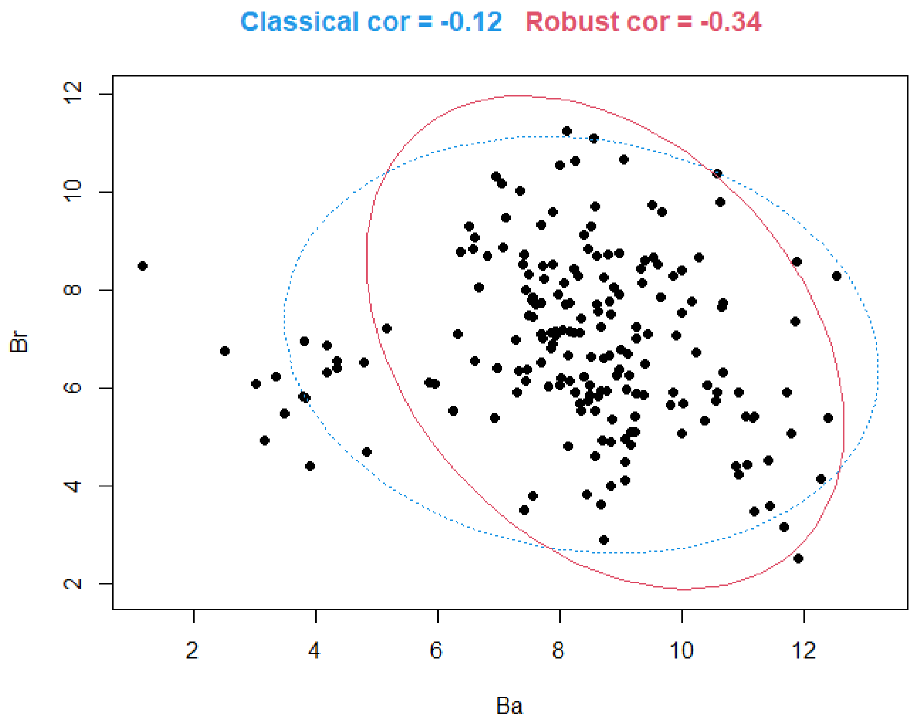

- **Br vs Sr**

Classical test	Pearson's product-moment correlation<br><br>data: Od533M_SD$Sr and Od533M_SD$Br<br>t = 0.19459, df = 188, **p-value = 0.8459**<br>alternative hypothesis: true correlation is not equal to 0<br>95 percent confidence interval:<br>-0.1284221 0.1562284<br>sample estimates:<br>**cor**<br>**0.01419062**	Spearman's rank correlation rho<br><br>data: Od533M_SD$Sr and Od533M_SD$Br<br>S = 1275574, **p-value = 0.1114**<br>alternative hypothesis: true rho is not equal to 0<br>sample estimates:<br>**rho**<br>**-0.115856**
MCD (α=0.05; quant=0.8)	$cor.rob<br>[1] **-0.2620026**	

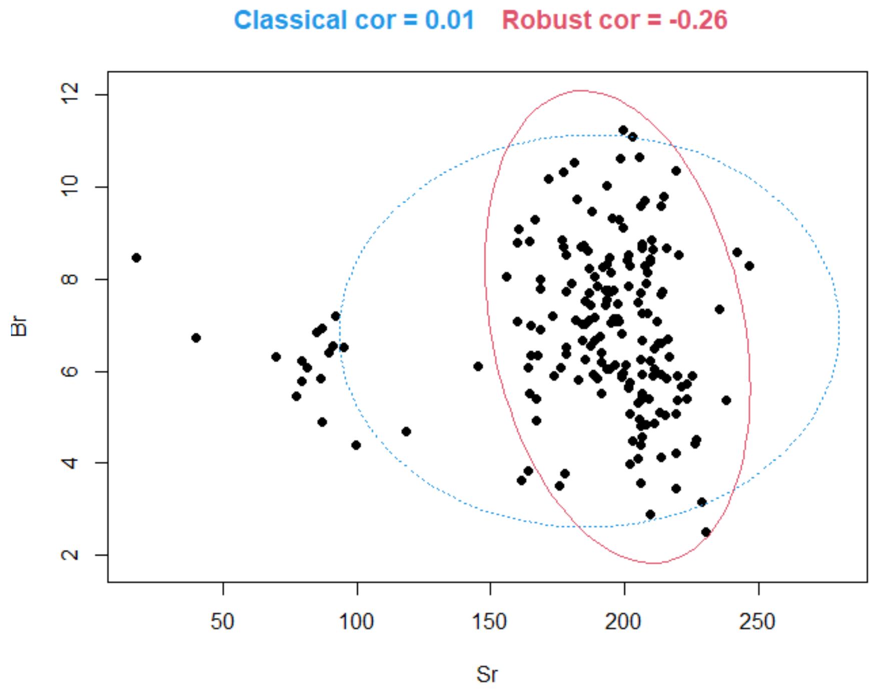

- **Br vs Zn**

Classical test	Pearson's product-moment correlation<br><br>data: Od533M_SD$Zn and Od533M_SD$Br<br>t = -1.6138, df = 188, **p-value = 0.1083**<br>alternative hypothesis: true correlation is not equal to 0<br>95 percent confidence interval:<br>-0.25499849 0.02589603<br>sample estimates:<br>**cor**<br>**-0.1168882**	Spearman's rank correlation rho<br><br>data: Od533M_SD$Zn and Od533M_SD$Br<br>S = 1232840, **p-value = 0.2816**<br>alternative hypothesis: true rho is not equal to 0<br>sample estimates:<br>**rho**<br>**-0.0784728**
MCD (α=0.05; quant=0.8)	$cor.rob<br>[1] **-0.08039107**	

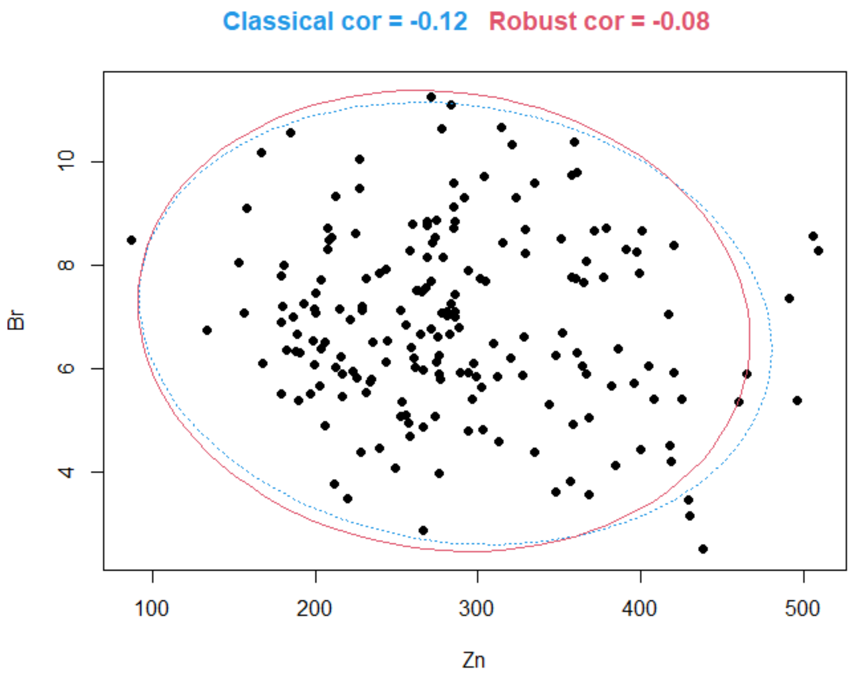

- **Br vs Ca**

Classical test	Pearson's product-moment correlation<br><br>data: Od533M_SD$Ca and Od533M_SD$Br<br>t = 0.59652, df = 188, **p-value = 0.5515**<br>alternative hypothesis: true correlation is not equal to 0<br>95 percent confidence interval:<br>-0.09950427 0.18467540<br>sample estimates:<br>**cor**<br>**0.04346472**	Spearman's rank correlation rho<br><br>data: Od533M_SD$Ca and Od533M_SD$Br<br>S = 1193966, **p-value = 0.5421**<br>alternative hypothesis: true rho is not equal to 0<br>sample estimates:<br>**rho**<br>**-0.04446631**
MCD (α=0.05; quant=0.8)	$cor.rob<br>[1] **-0.2620026**	

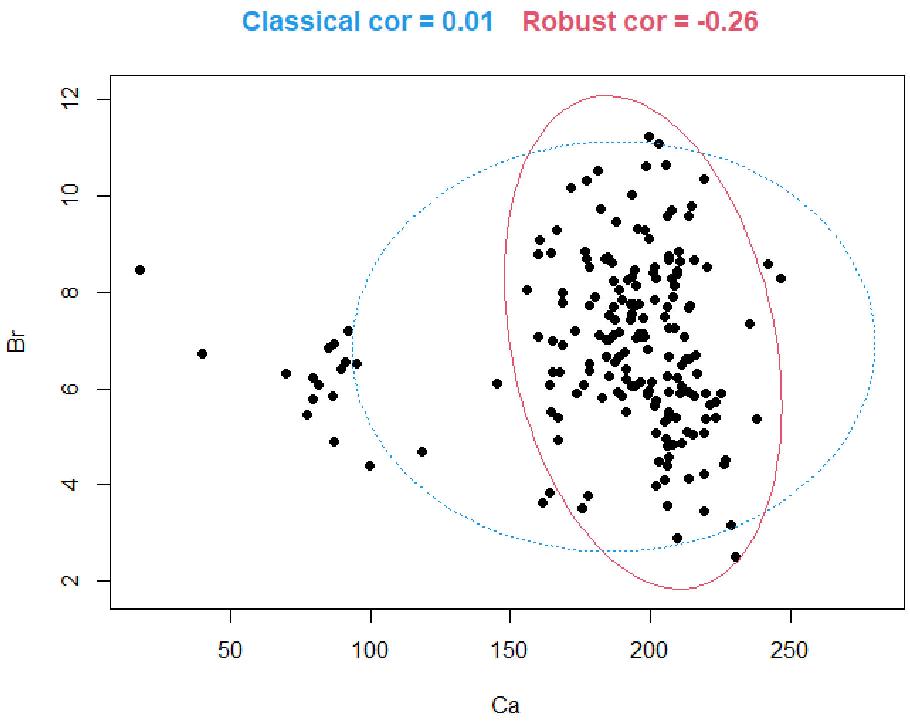

Supplement: Supplementary file 1 [file biology-12-00184-s001.zip › biology-2121048-Supplementary Material/Brozou_et_al_File_S1.pdf]
