# Supplementary material for: Using SXRF and LA-ICP-TOFMS to Explore Evidence of Treatment and Physiological Responses to Leprosy in Medieval Denmark
Source: Biology (Basel). 2023 Jan 25;12(2):184. doi: 10.3390/biology12020184 (PMC9952905; doi:10.3390/biology12020184)

File S2

Synchrotron Fluorescence (SXRF) results

Using SXRF and LA-ICP-TOFMS to explore evidence of treatment and physiological responses to leprosy in medieval Denmark

Biology

Anastasia Brozou, Marcello A. Mannino, Stijn J.M. Van Malderen, Jan Garrevoet, Eric Pubert, Benjamin T. Fuller, M. Christopher Dean, Thomas Colard, Frédéric Santos, Niels Lynnerup, Jesper L. Boldsen, Marie Louise Jørkov, Andrei Dorian Soficaru, Laszlo Vincze, Adeline Le Cabec

Corresponding author:

Adeline Le Cabec
E-mail: adeline.le-cabec@u-bordeaux.fr

Data reconstruction

- Raw data were fitted and calibrated.
- The calculation takes into account the **average thickness of the tooth section** measured by subtracting the thickness of the glass slide from {tooth section + epoxy bond ~negligible thickness + glass slide}.
- The **background noise has been subtracted** from the sample for each element.

In the next slides:
- *"Scanning"*: actual areas scanned.
- Images reported in this ppt are "Mass diffraction", in $mg.kg^{-1}$ (ppm).
- It is also possible to report "areal density", in $g.cm^{-3}$
- To save space, the elemental maps of some teeth may have been rotated by 90°.
- Maps are displayed using the "Gistheat" colormap.
- To enhance the banding/stress pattern while reducing overall noise, a 2D Gaussian filter was applied to the datasets. The kernel size (=strength of the filter) used is indicated for x and y. This will induce a slight smoothing of any curve generated from a transect. The curve may be saved from the same transect on the unfiltered dataset.
- To improve the visibility of distribution patterns, the contrast is adjusted on the maps, thus not displaying the full range of concentrations recorded in the scan. Numbers cited in the text have been directly measured on the scans, with the full range of concentrations.

K emission lines.

- For elements which Kα < 17 keV, the detectors capture the K emission lines within the remitted fluorescent light: Ca, Zn, Sr, Cu, Fe, Mn.
- Data are fitted and calibrated, and were processed (open .nxs, colormap, 2D Gauss filter) in HDIP.

L emission lines.

- For elements which Kα > 17 keV, the detectors only capture the L emission lines within the remitted fluorescent light: Pb and Ba.
- For these elements, data were NOT calibrated.
- For each element, the 32 bit images from Detector 0 and Detector 2 were added in Image J (Process> Image Calculator), and then filtered using a Gaussian blur (Process> Filters> Gaussian blur) with a kernel size of 0.7-0.9.

Romania – R1386 LRM2

♂ 36 yrs. 1890 – 1926 CE

Blacksmith deceased in Colentina Hospital (Bucharest)

Average tooth section thickness (µm): 112.5

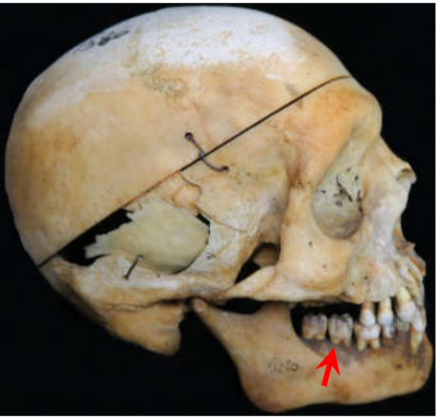

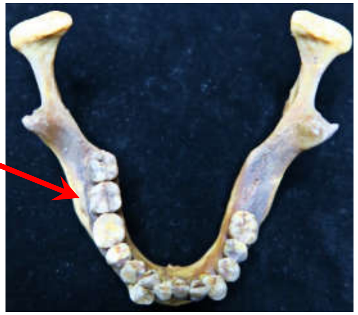

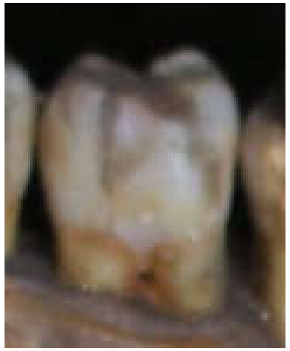

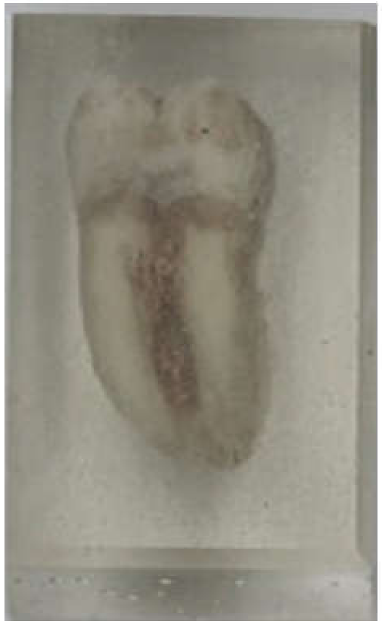

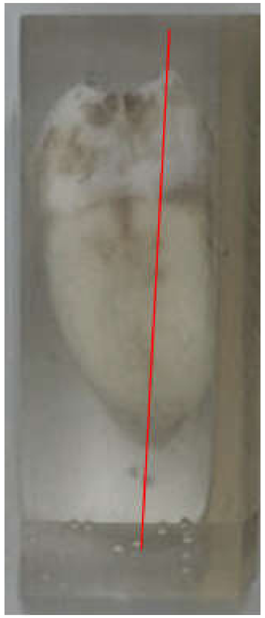

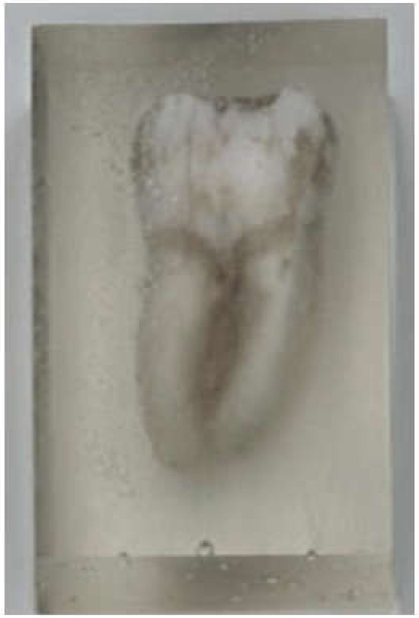

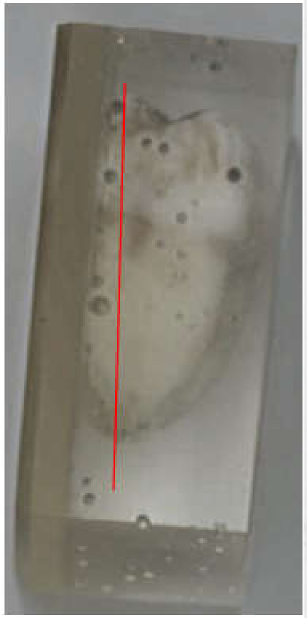

Distal

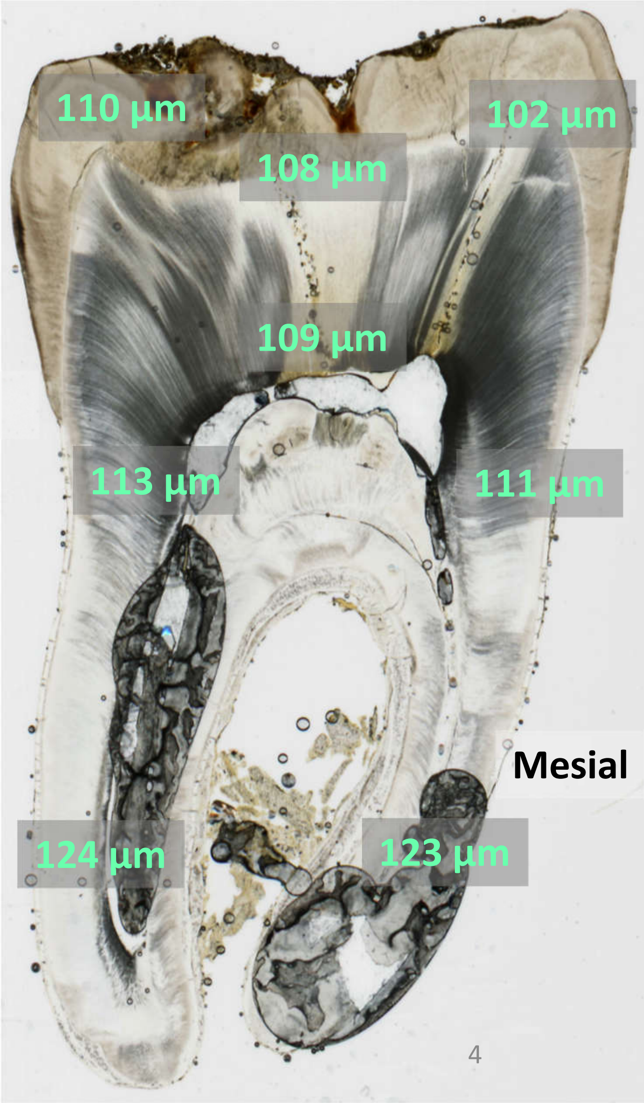

R1386 LRM2

Scanning

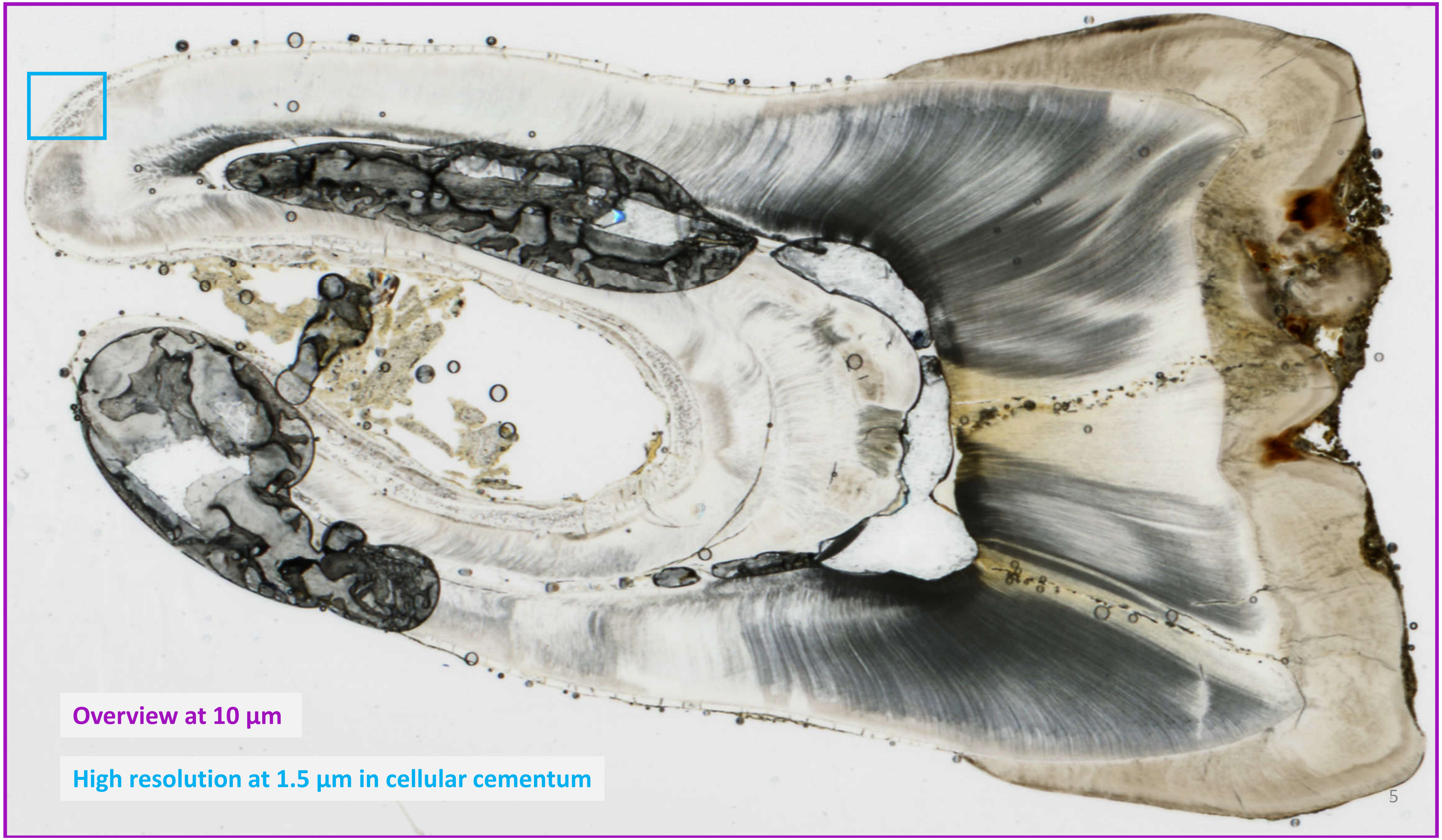

Overview at 10 µm

High resolution at 1.5 µm in cellular cementum

R1386 LRM2

Overview at 10 µm

Gauss (1x1)

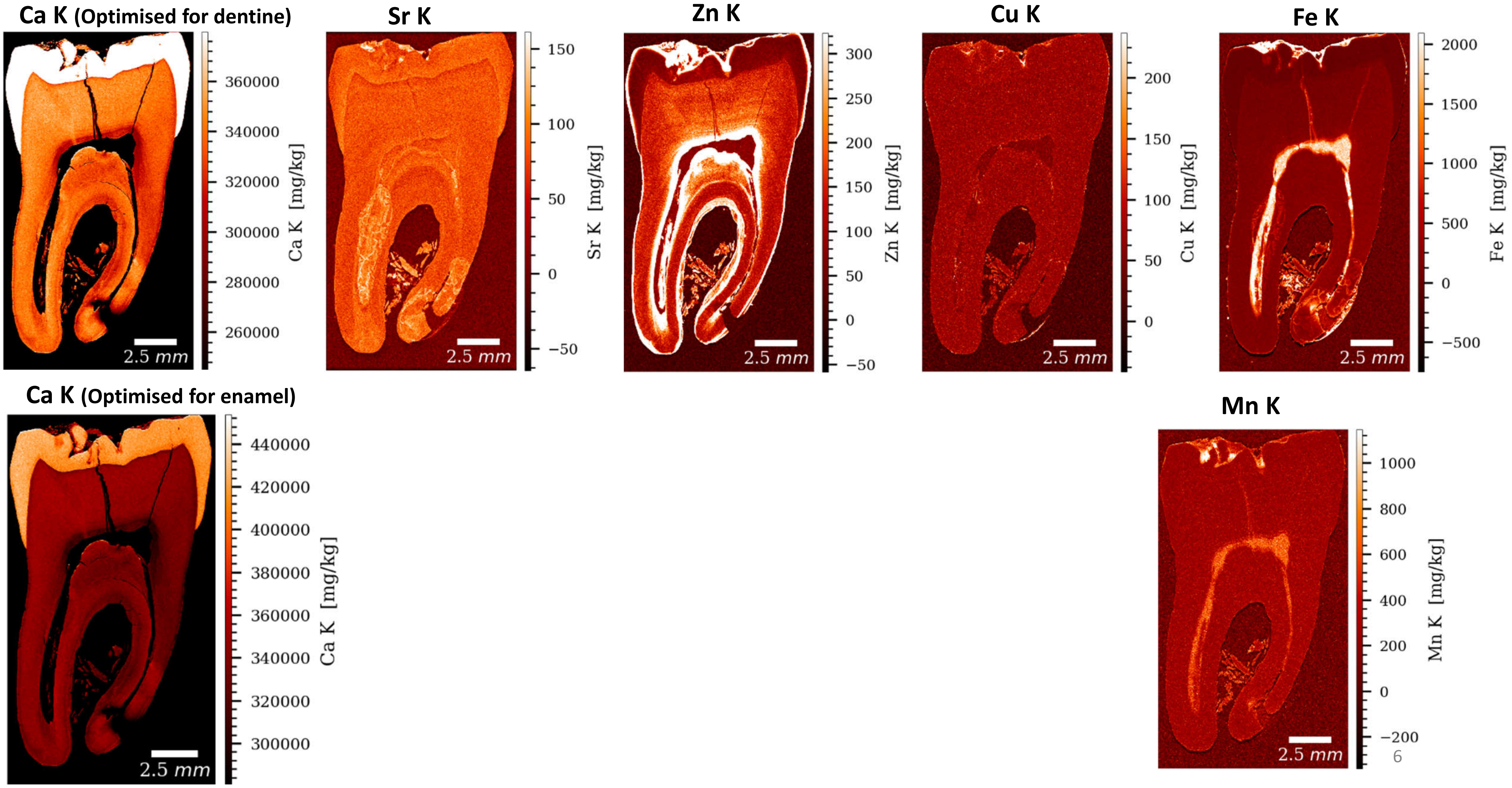

R1386 LRM2

Overview at 10 µm

Gauss (1x1)

Close-ups for elemental variations in the dentine

Ca K

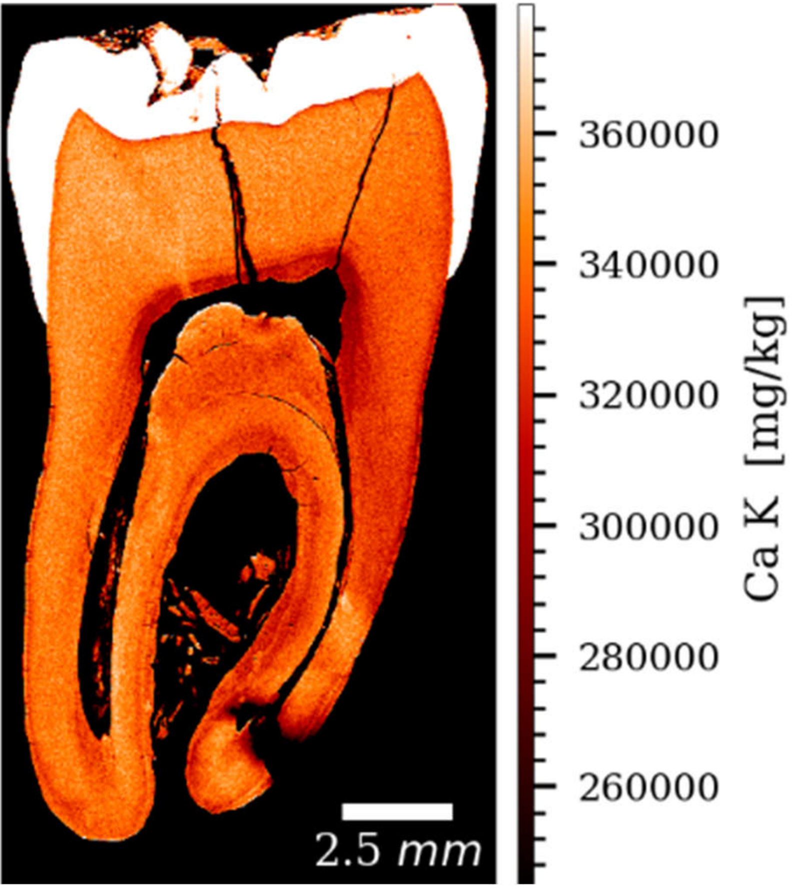

Sr K

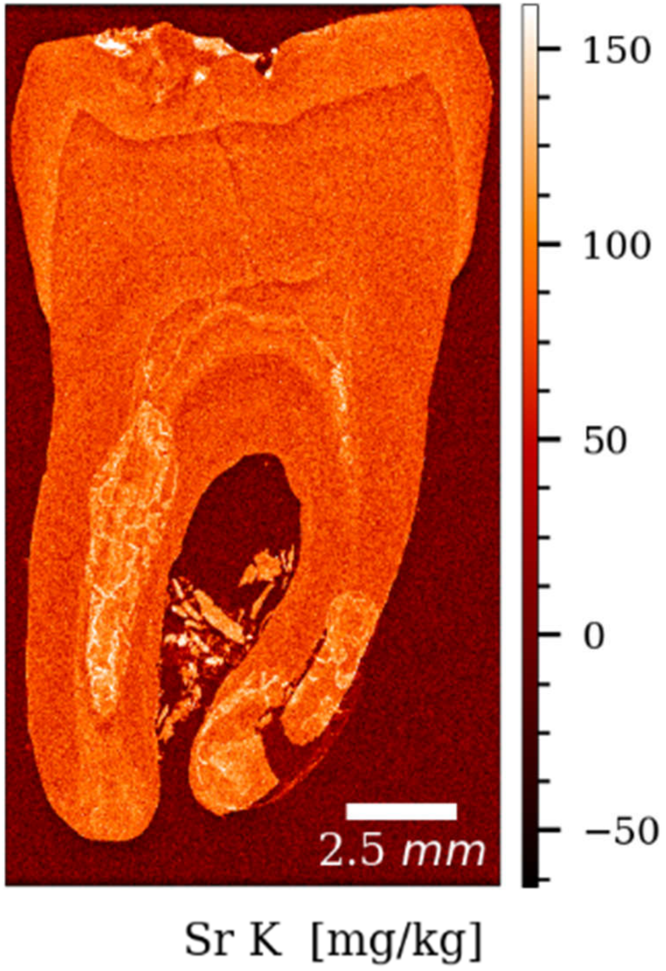

Zn K

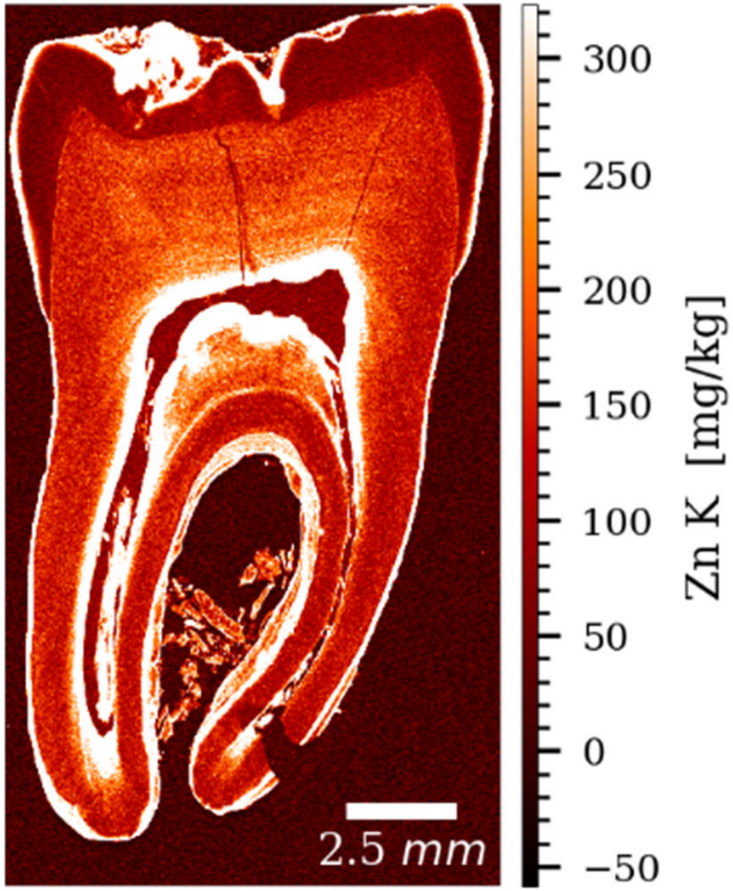

R1386 LRM2

Overview at 10 µm

Uncalibrated data (arbitrary units)

Gauss (1x1)

Br K

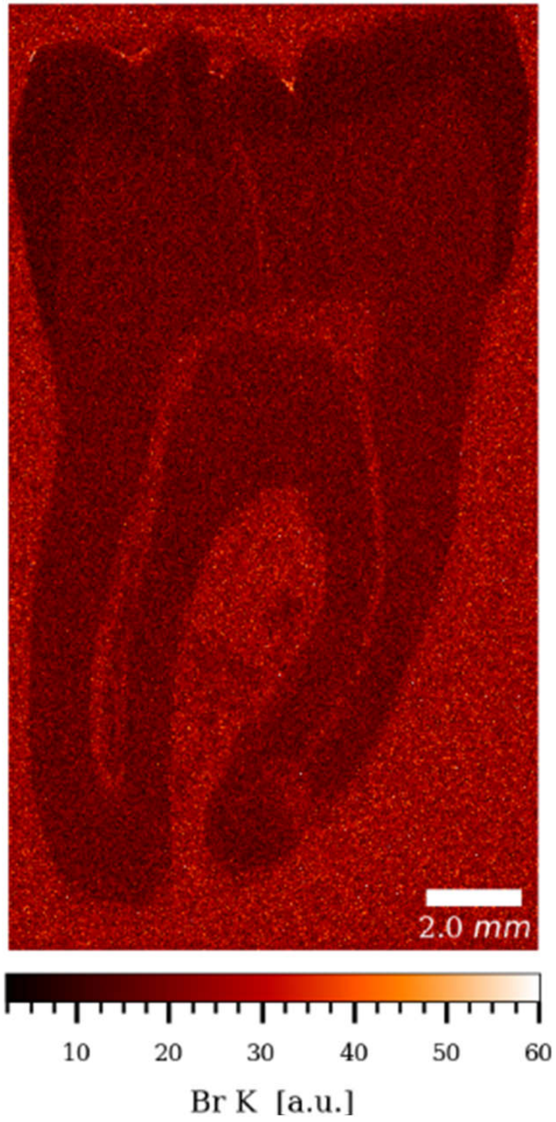

Pb L

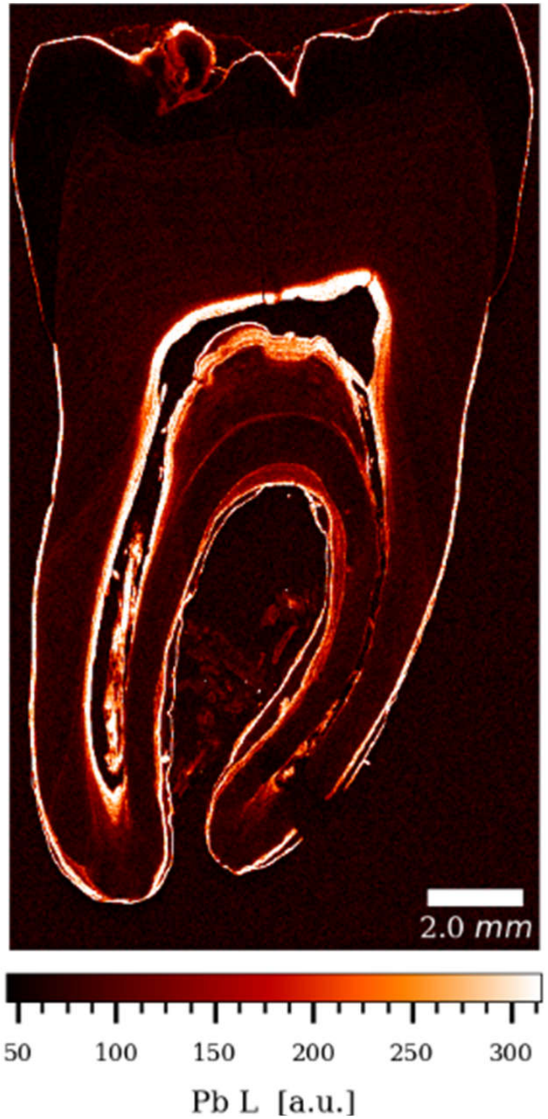

Optimised for cementum and secondary dentine

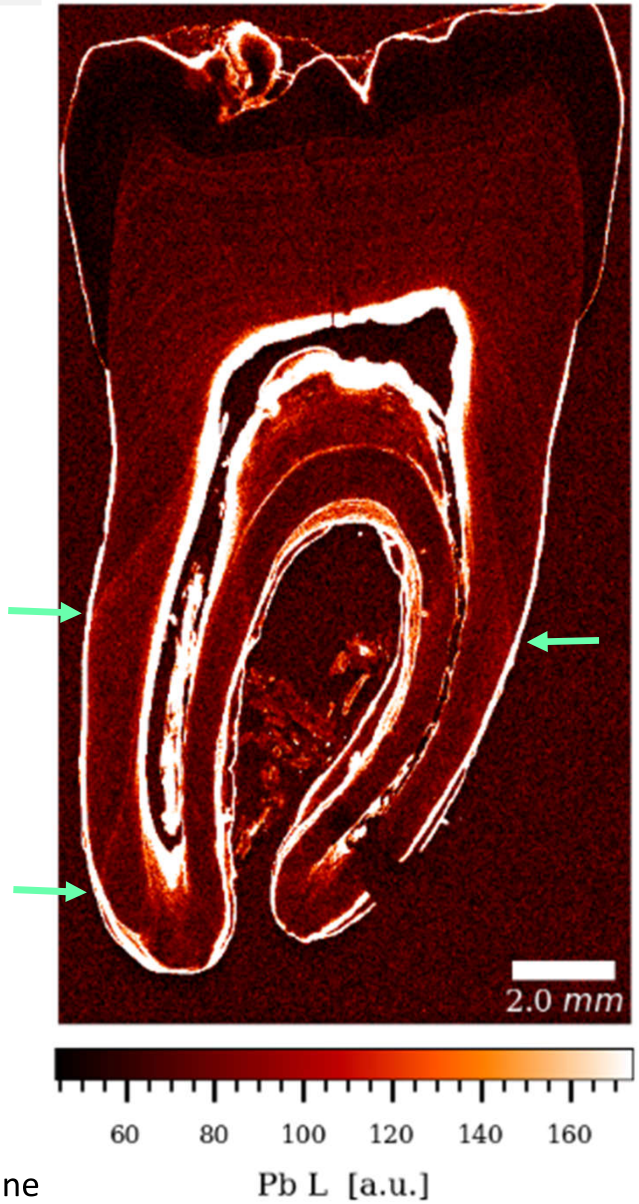

- Optimised for primary dentine
- Significant increase in Pb at mid-root (~adolescence)

Overview at 10 µm

Gauss (1x1)

Close-ups for elemental variations in the cementum and secondary dentine

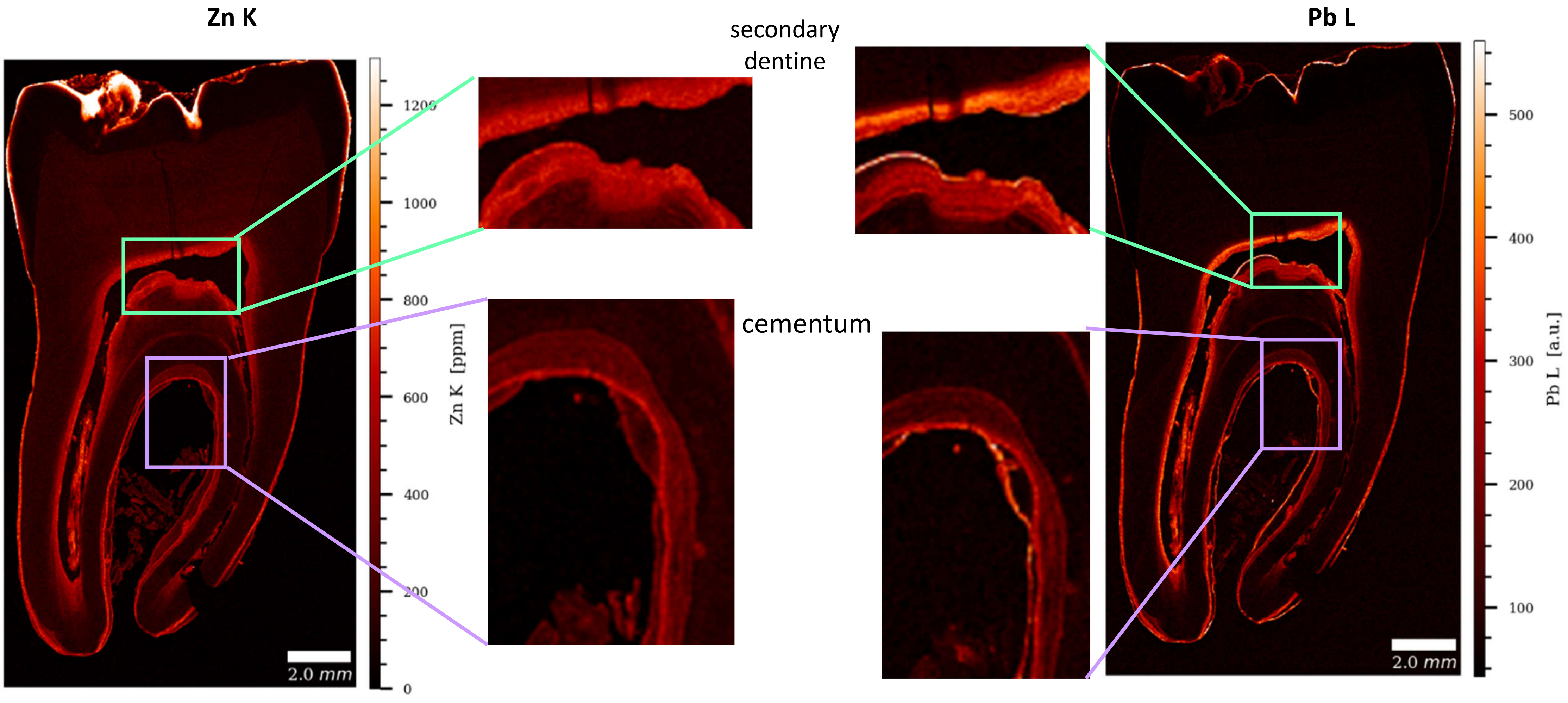

R1386 LRM2

High resolution at 1.5 µm

Gauss (0.8x0.8)

Ca K

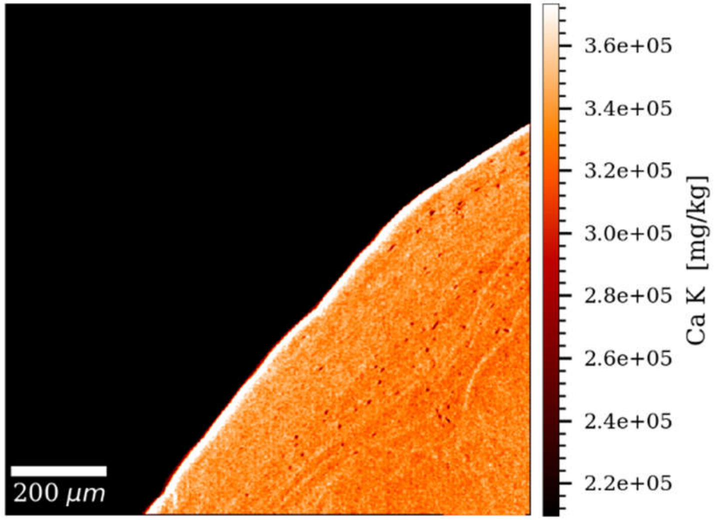

Sr K

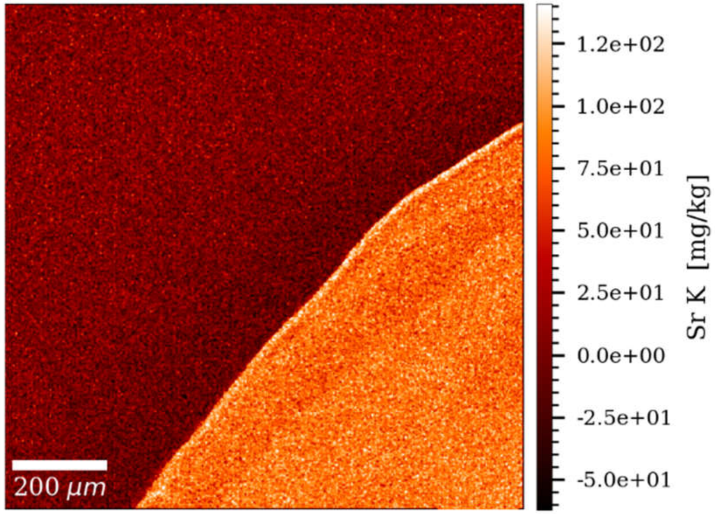

Zn K

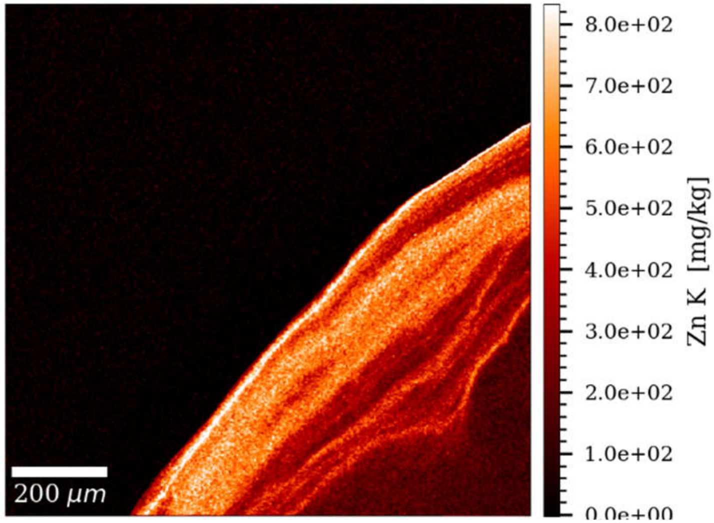

Cu K

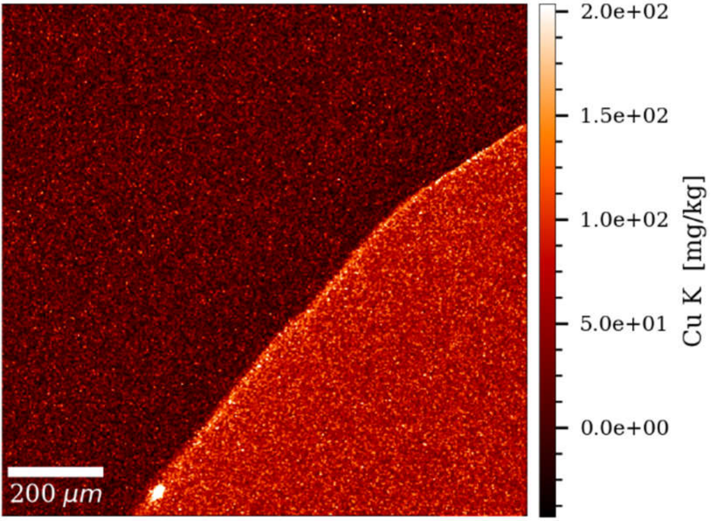

Fe K

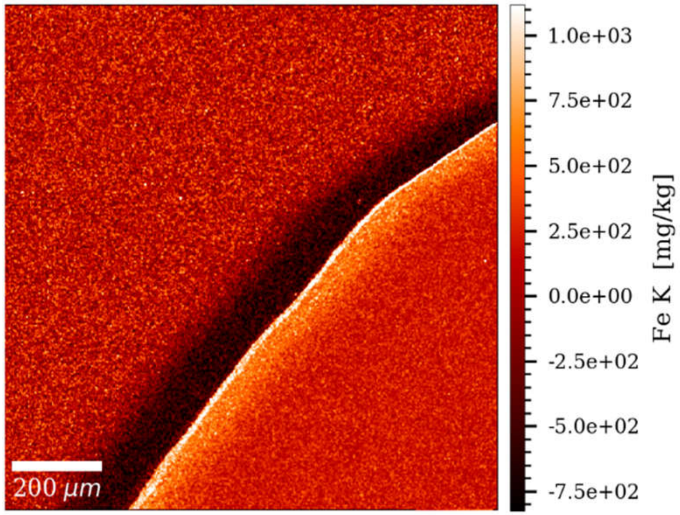

Mn K

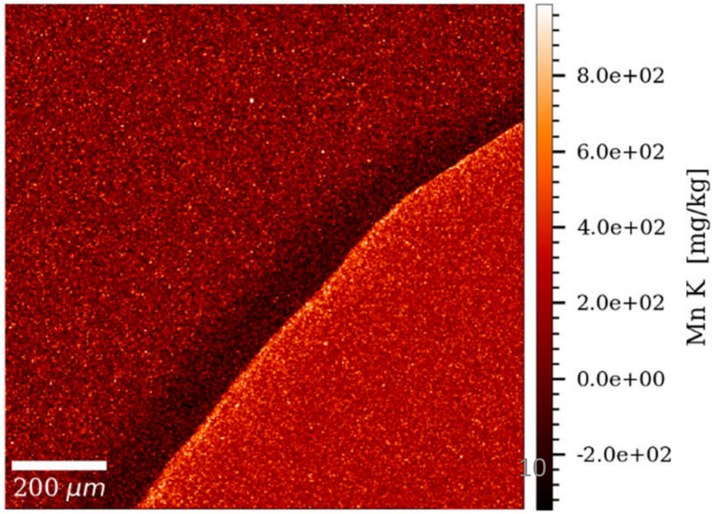

R1386 LRM2

High resolution at 1.5 µm

Gauss (0.8x0.8)

Pb L

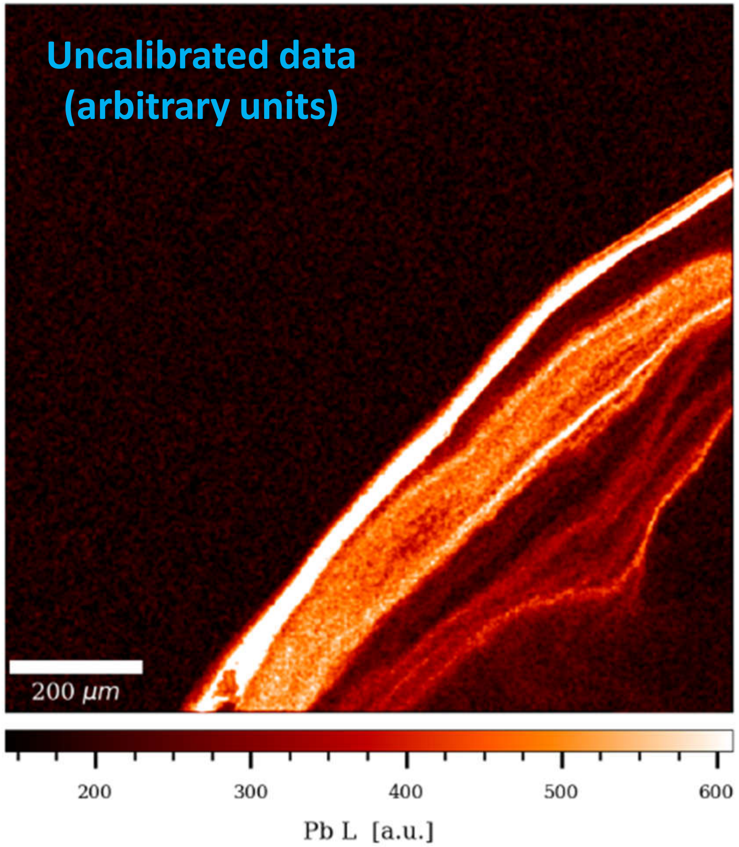

Mn K

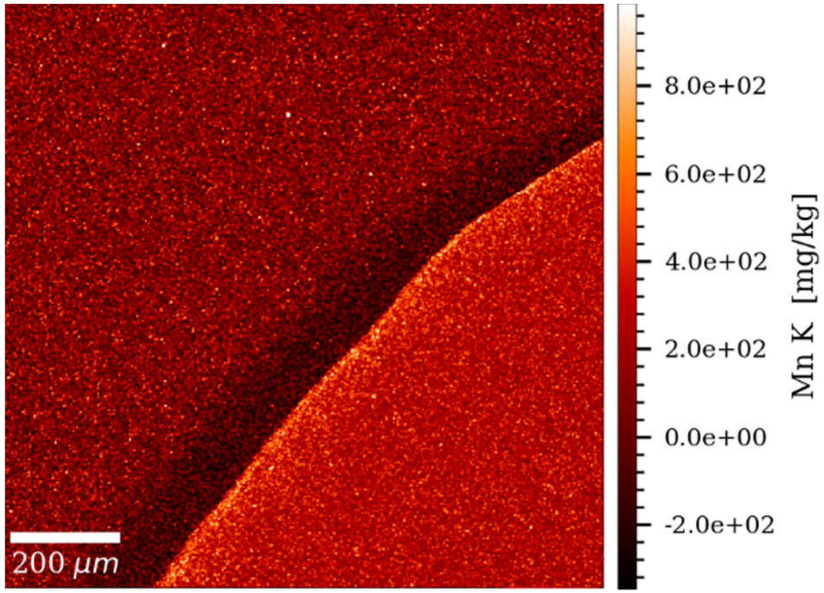

Romania – A1651 LRM1

♂ **Beginning of 20th c.**

Average tooth section thickness (µm): 121.75

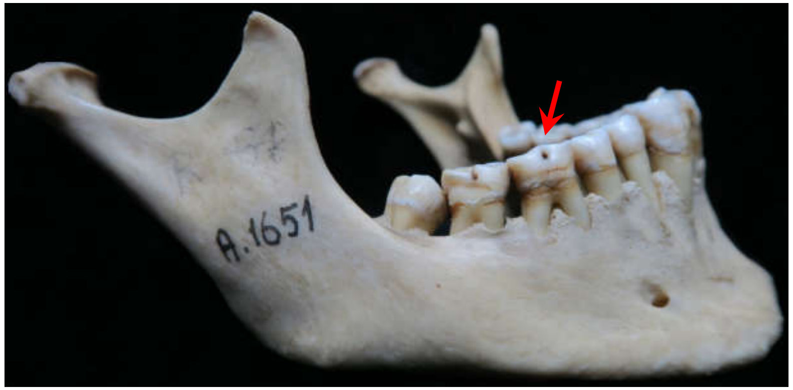

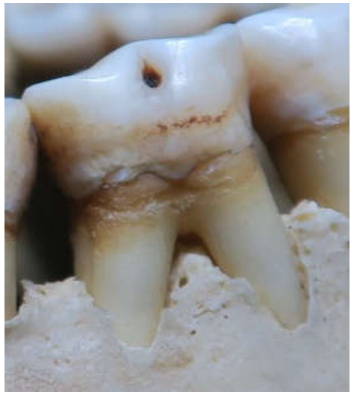

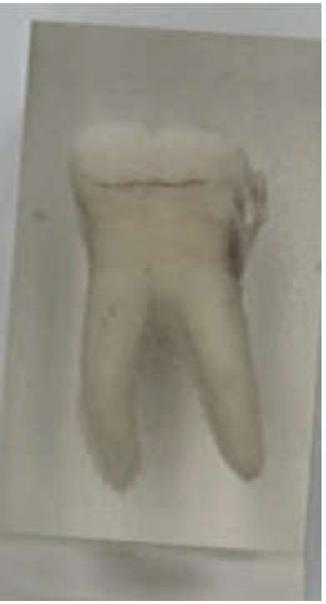

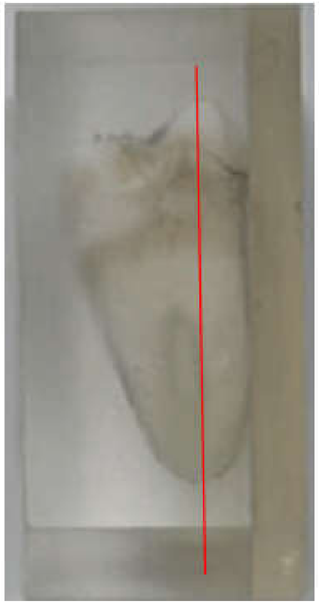

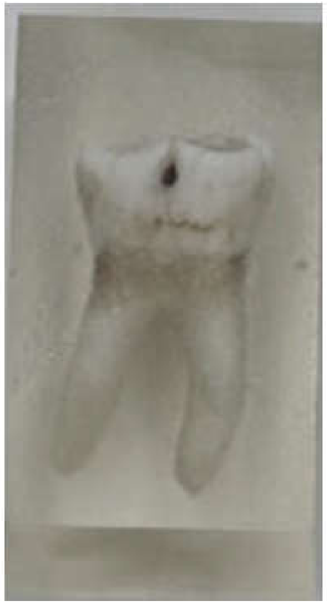

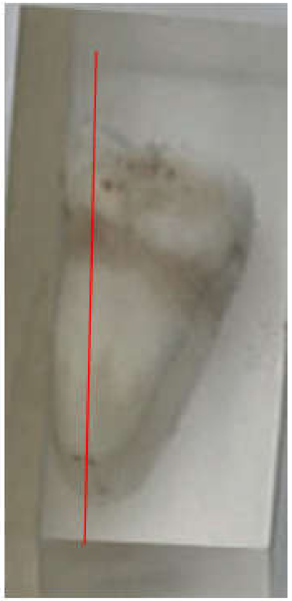

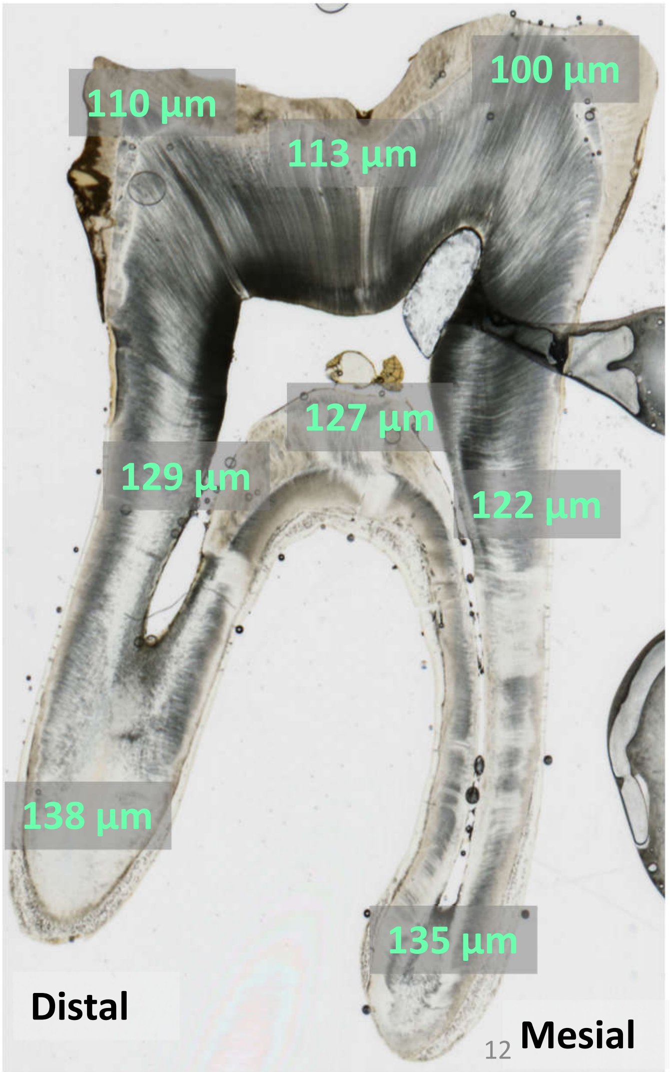

A1651 LRM1

Scanning

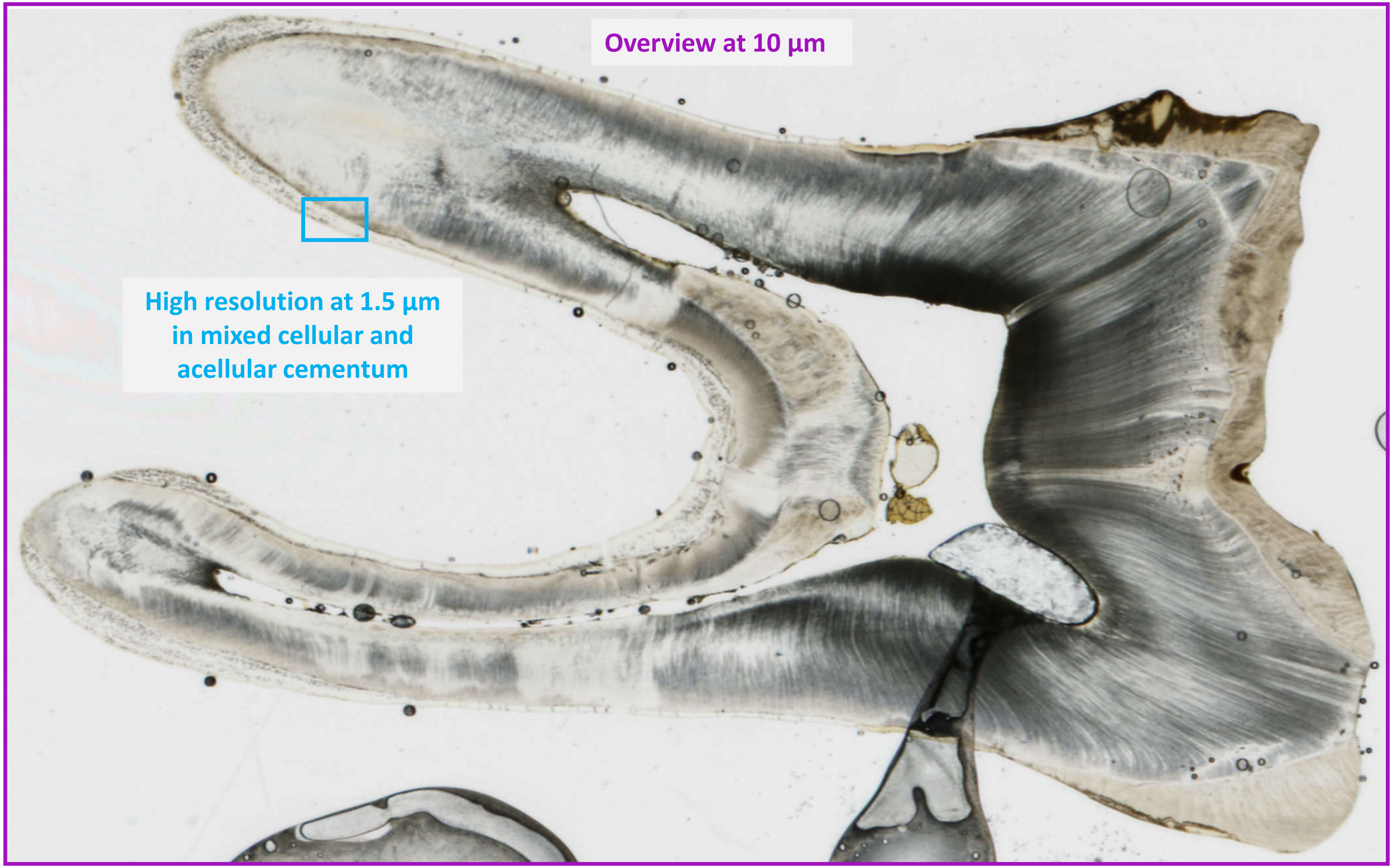

A1651 LRM1

Overview at 10 µm

Gauss (0.7x0.7)

Ca K Sr K Zn K

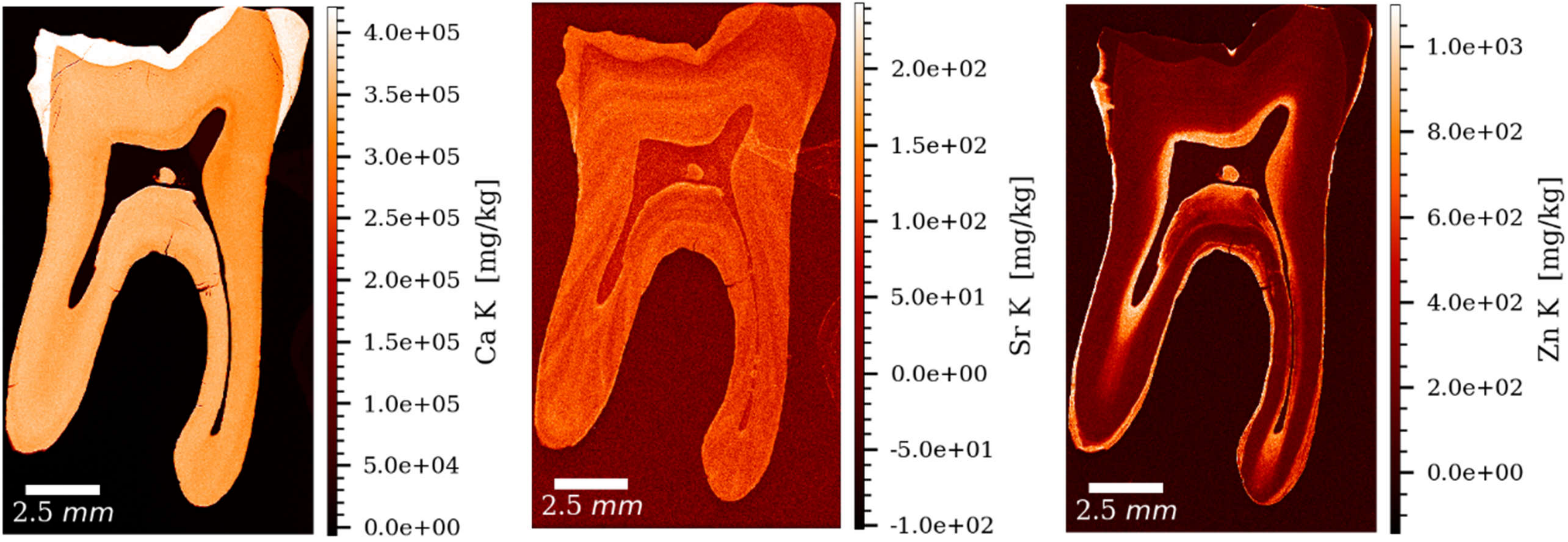

A1651 LRM1

Overview at 10 µm

Gauss (0.7x0.7)

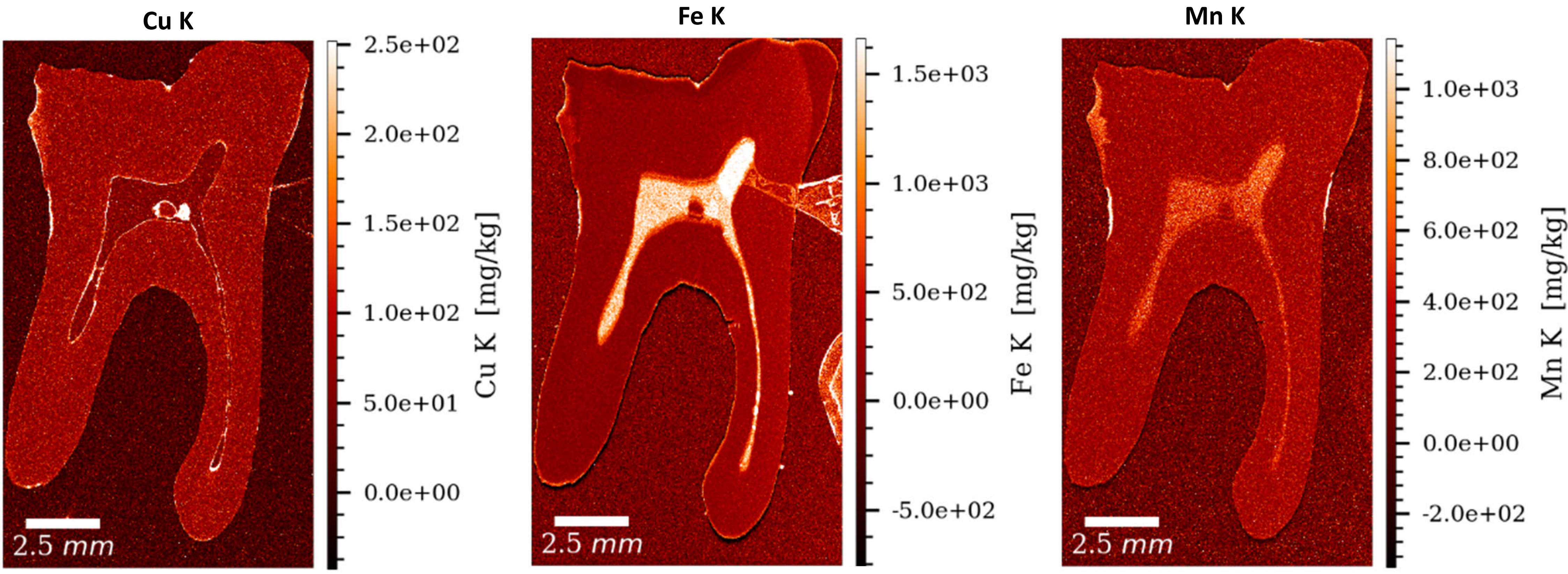

A1651 LRM1

Overview at 10 µm

Uncalibrated data
(arbitrary units)

Gauss (0.7x0.7)

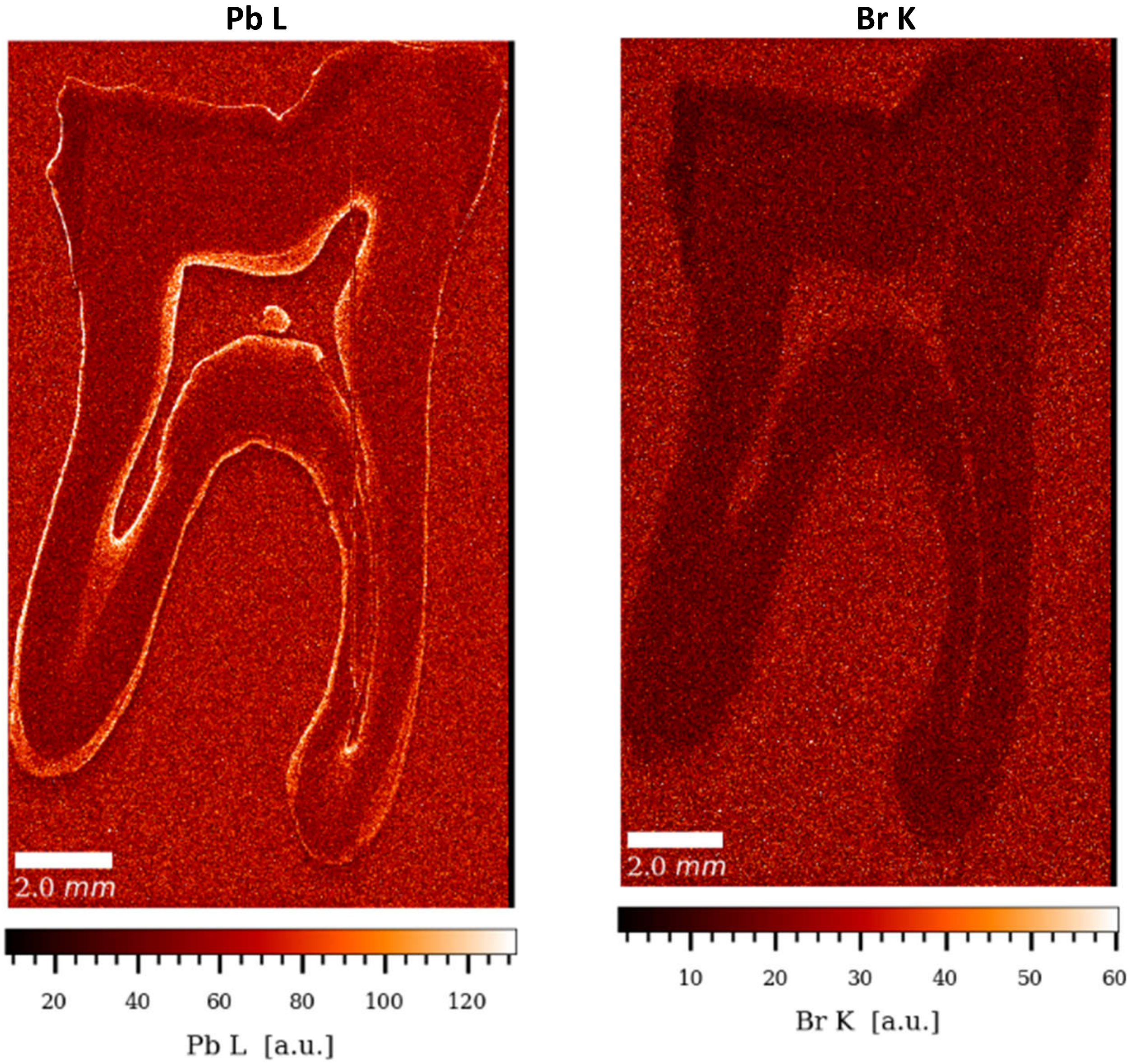

A1651 LRM1

High resolution at 1.5 µm

Gauss (0.85 x0.85)

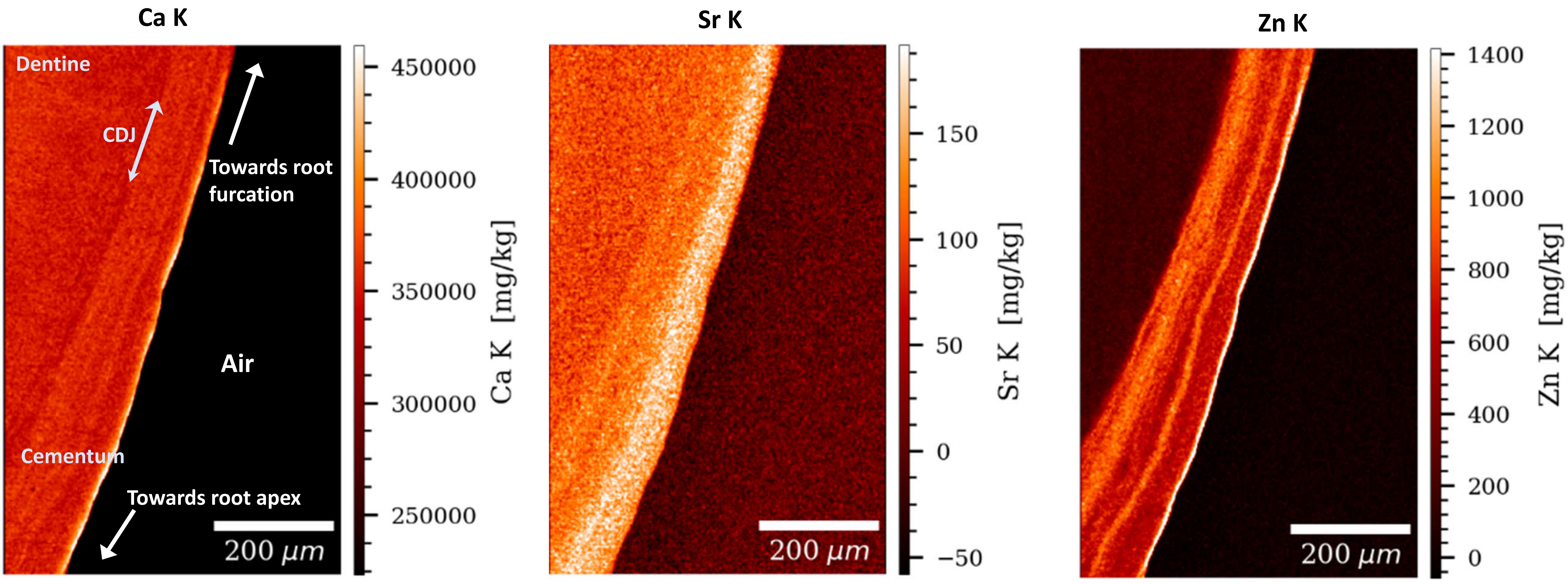

A1651 LRM1

High resolution at 1.5 µm

Gauss (0.85 x0.85)

Cu K

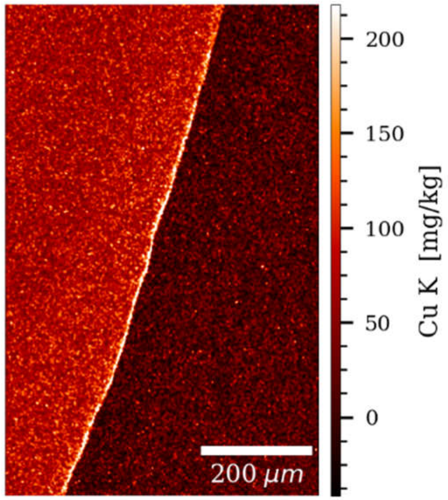

Fe K

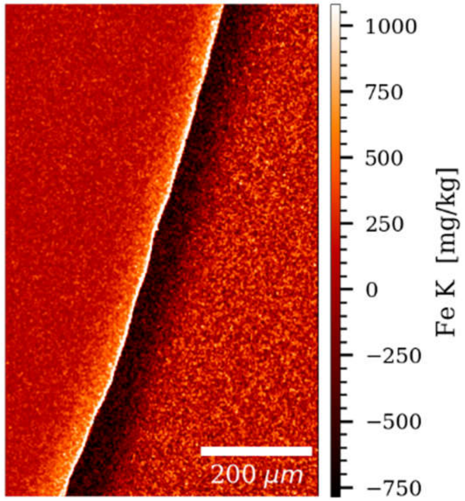

Mn K

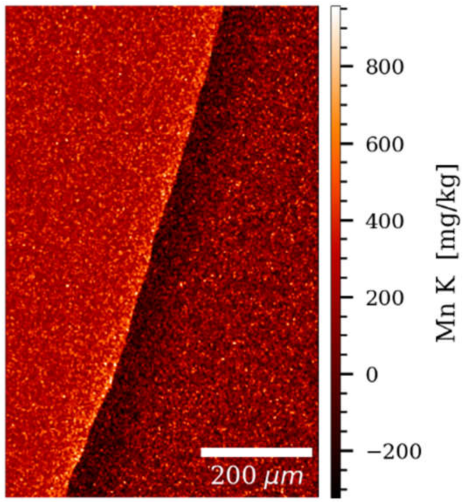

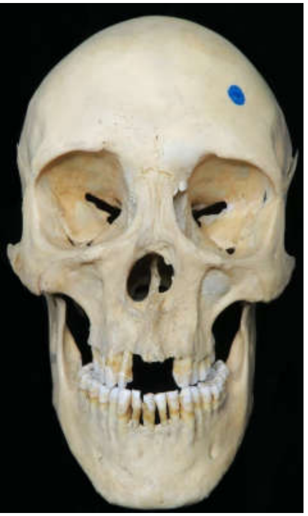

Romania – A1651 URC

♂ **Beginning of 20th c.**

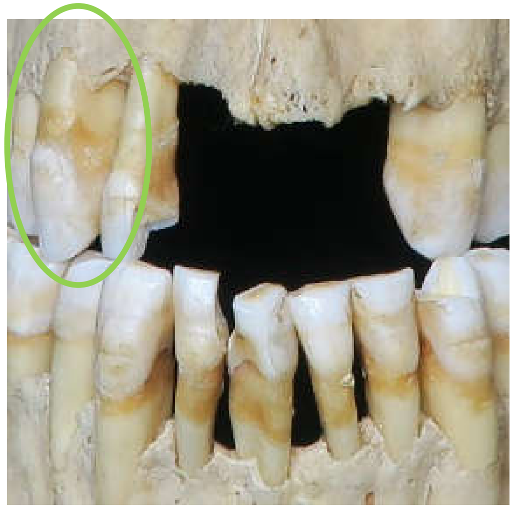

Average tooth section thickness (µm): 86.2

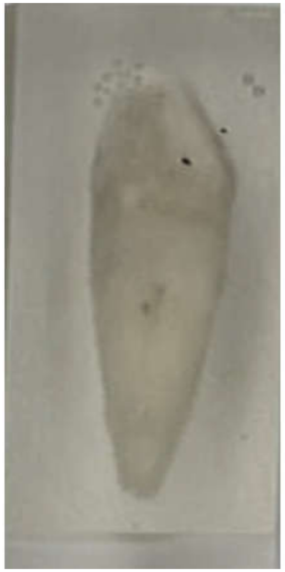

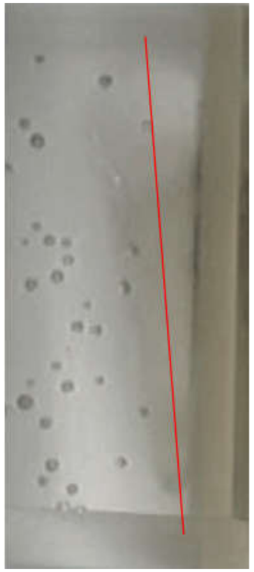

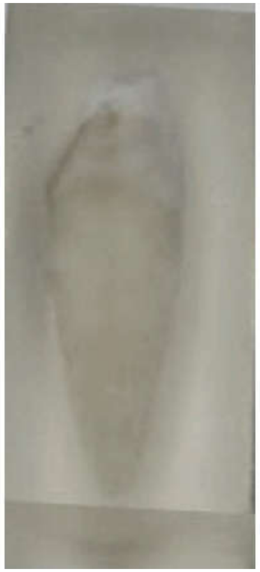

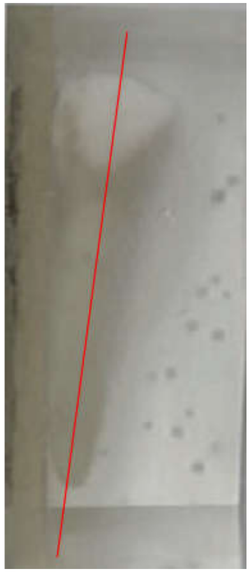

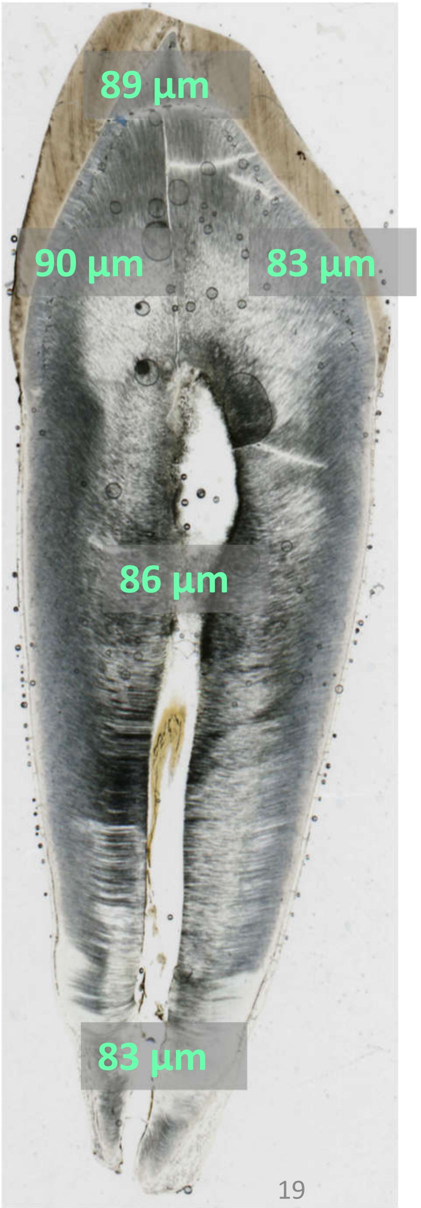

A1651 URC

Scanning

Overview at 10 µm

High resolution at 1 µm in cellular cementum

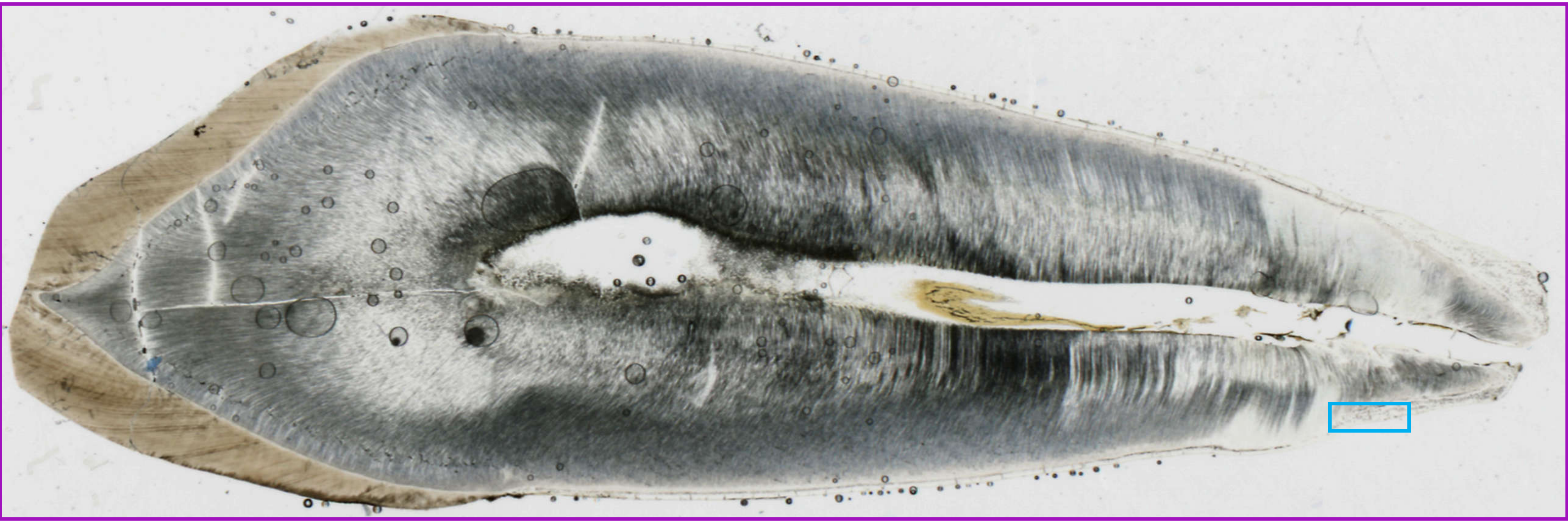

A1651 URC

Overview at 10 µm

Gauss (0.9x0.9)

Ca K   Sr K   Zn K   Fe K

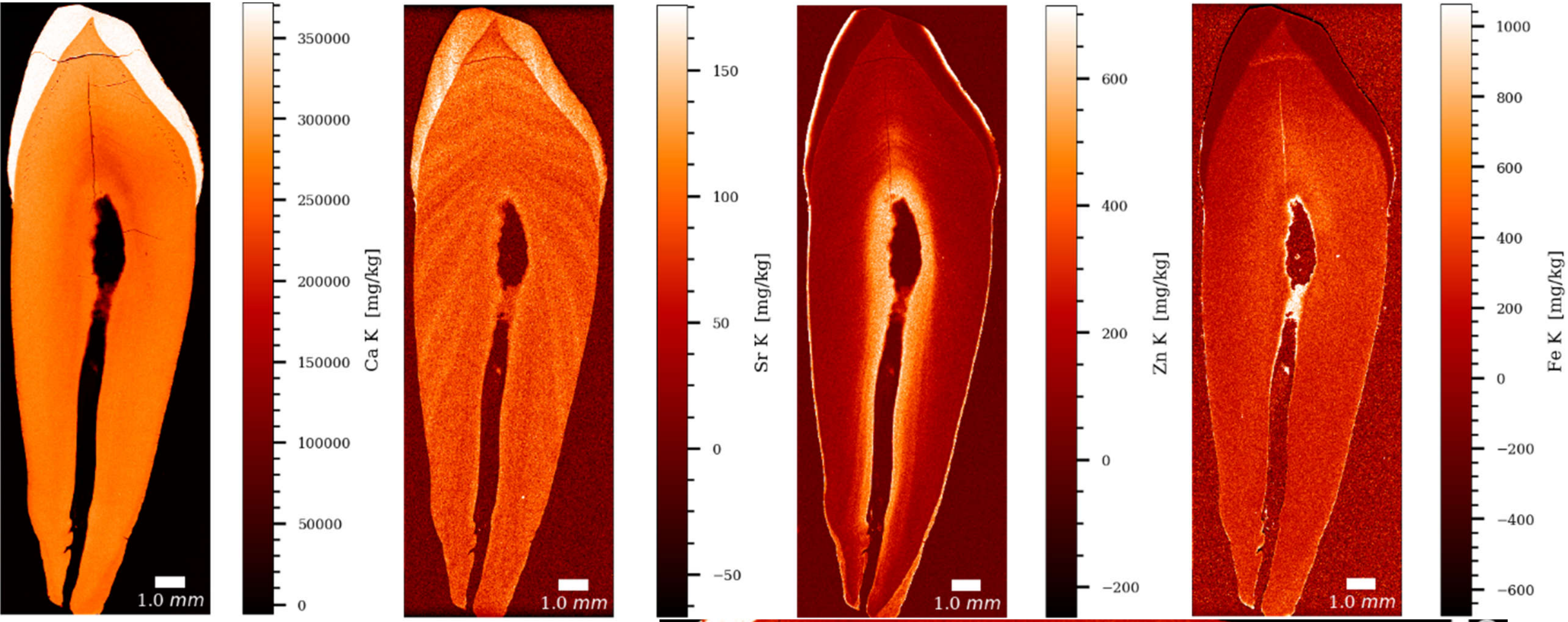

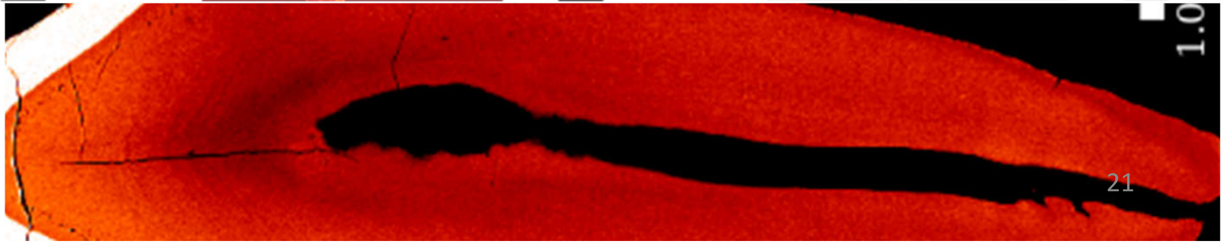

Ca K (optimised for dentine for elemental variations)

A1651 URC

Overview at 10 µm

Gauss (0.9x0.9)

Cu K

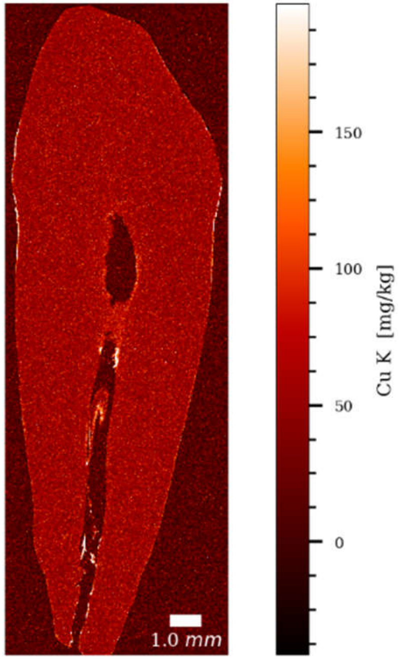

Fe K

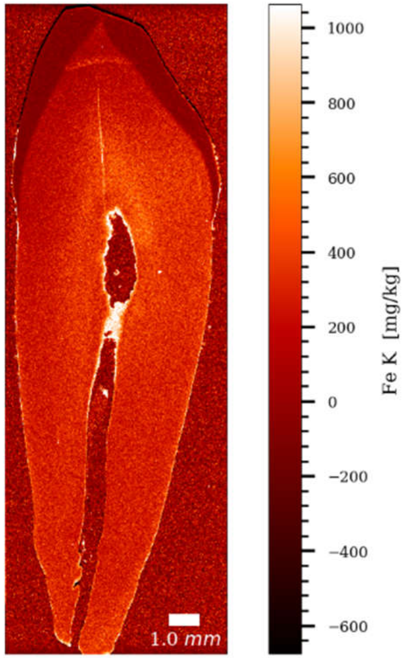

Mn K

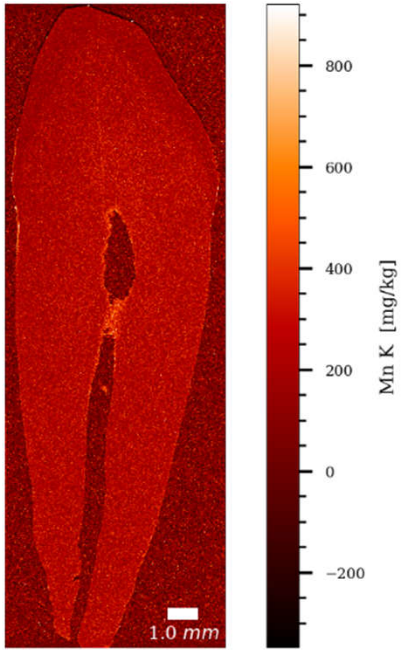

A1651 URC

High resolution at 1 µm

Gauss (1x1)

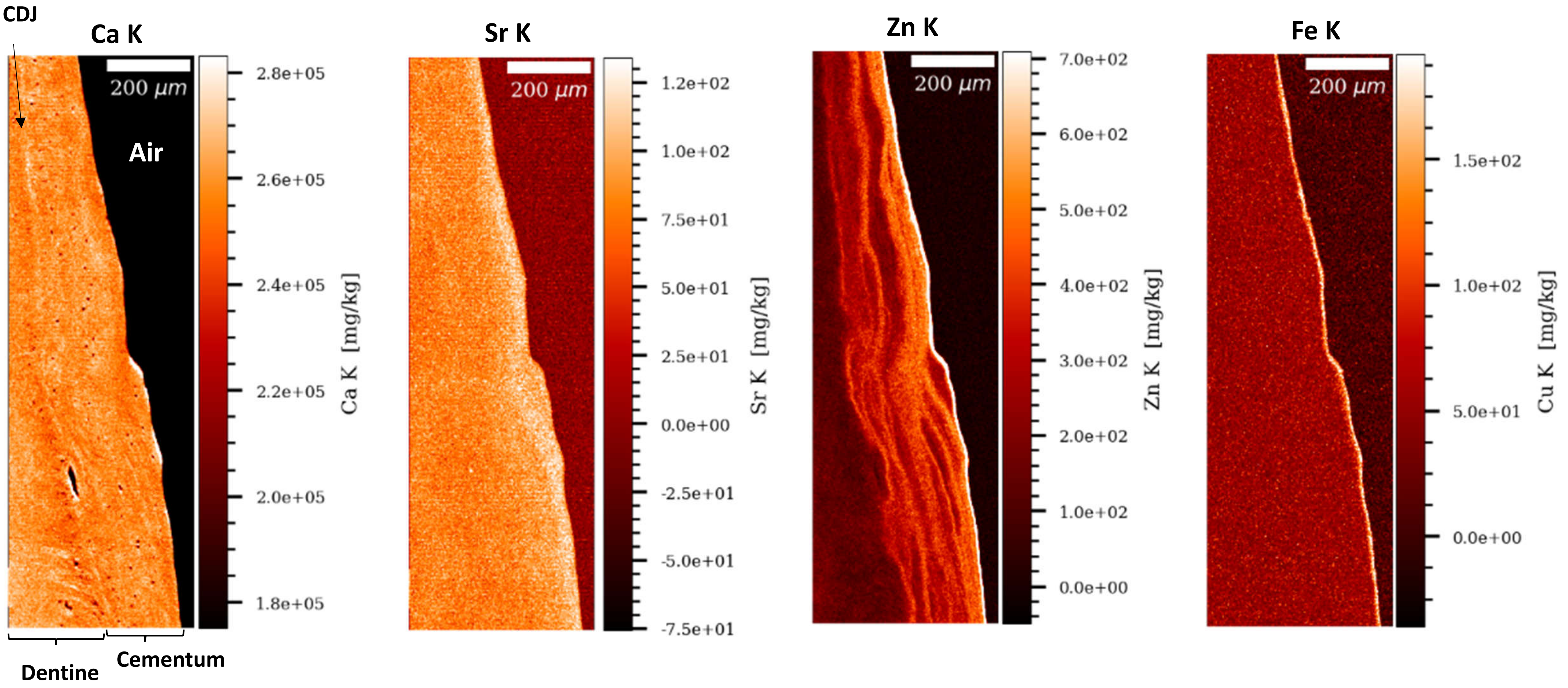

High resolution at 1 μm

Gauss (1x1)

Mn K

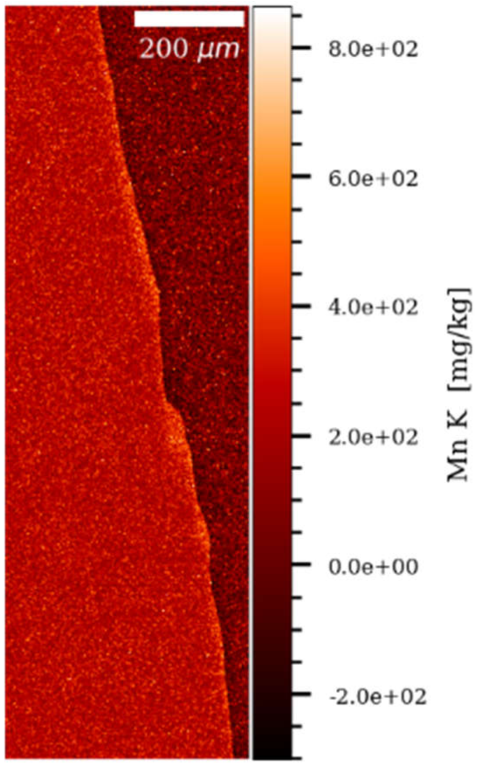

Cu K

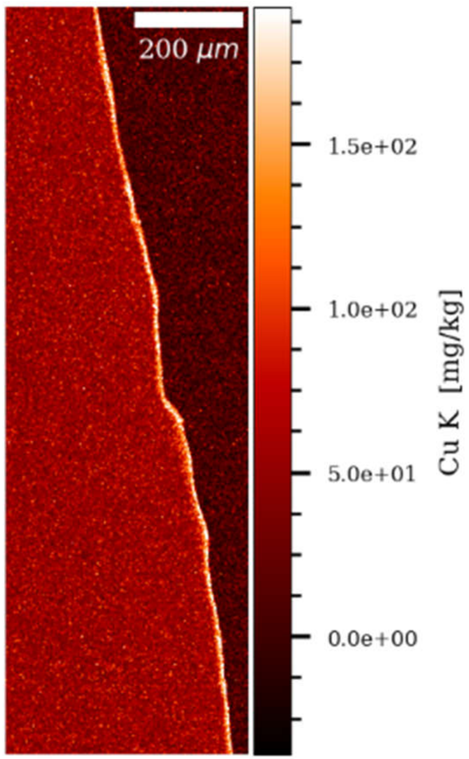

Odense – 533 LLM1

♀ 25-35 yrs. 1191 – 1269 cal. CE

Average tooth section thickness (µm): 104.0

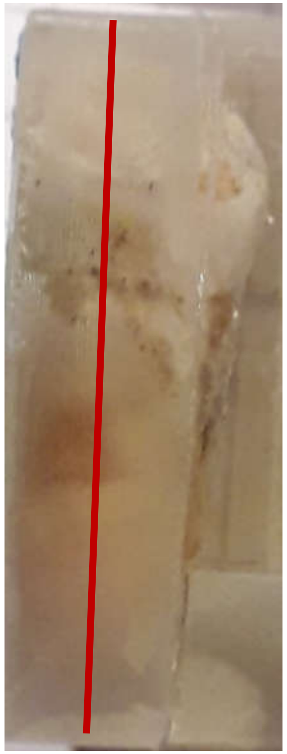

mesial

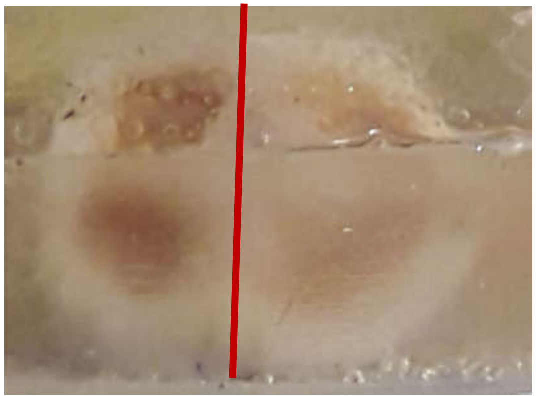

distal

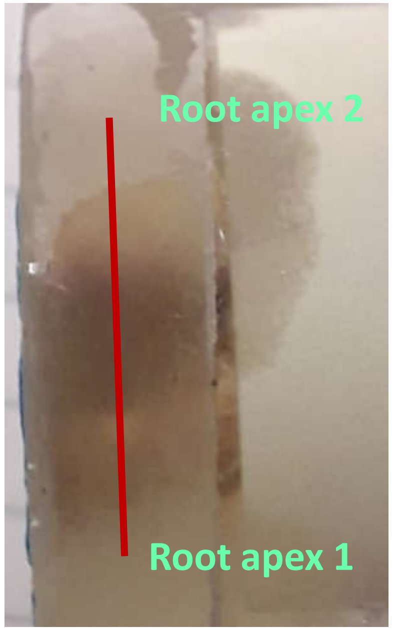

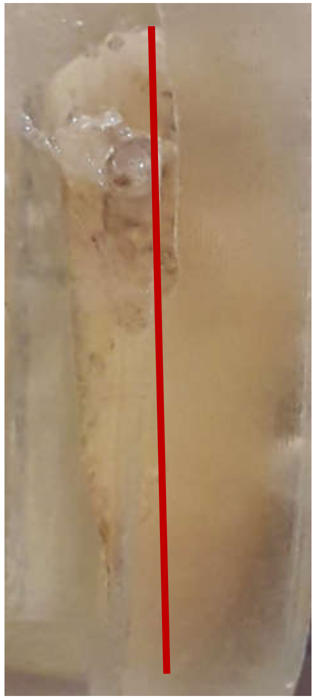

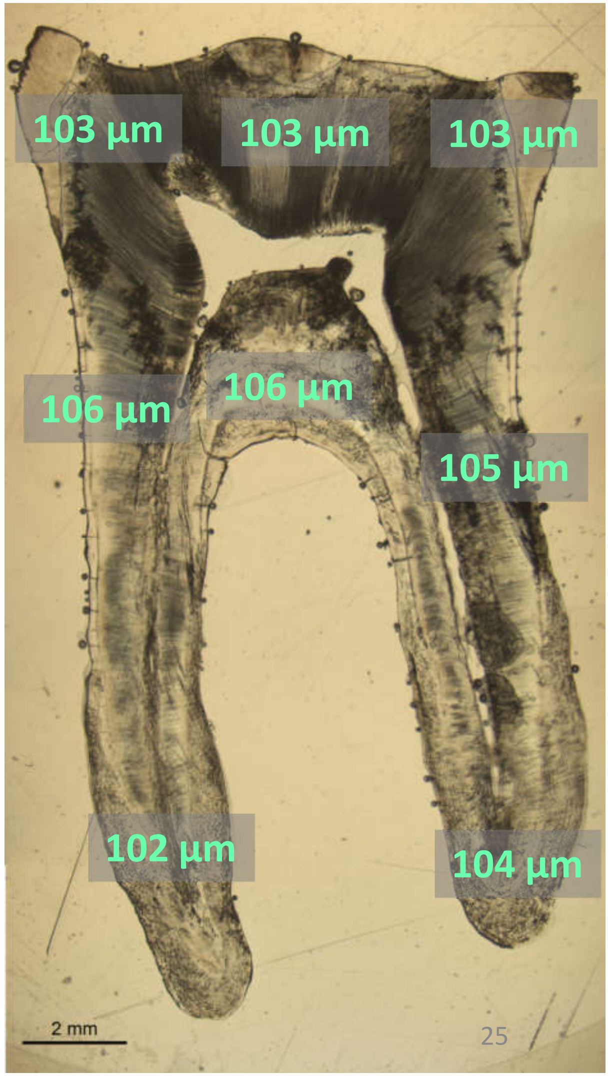

Odense 533 LLM1

Scanning

Overview at 10 µm

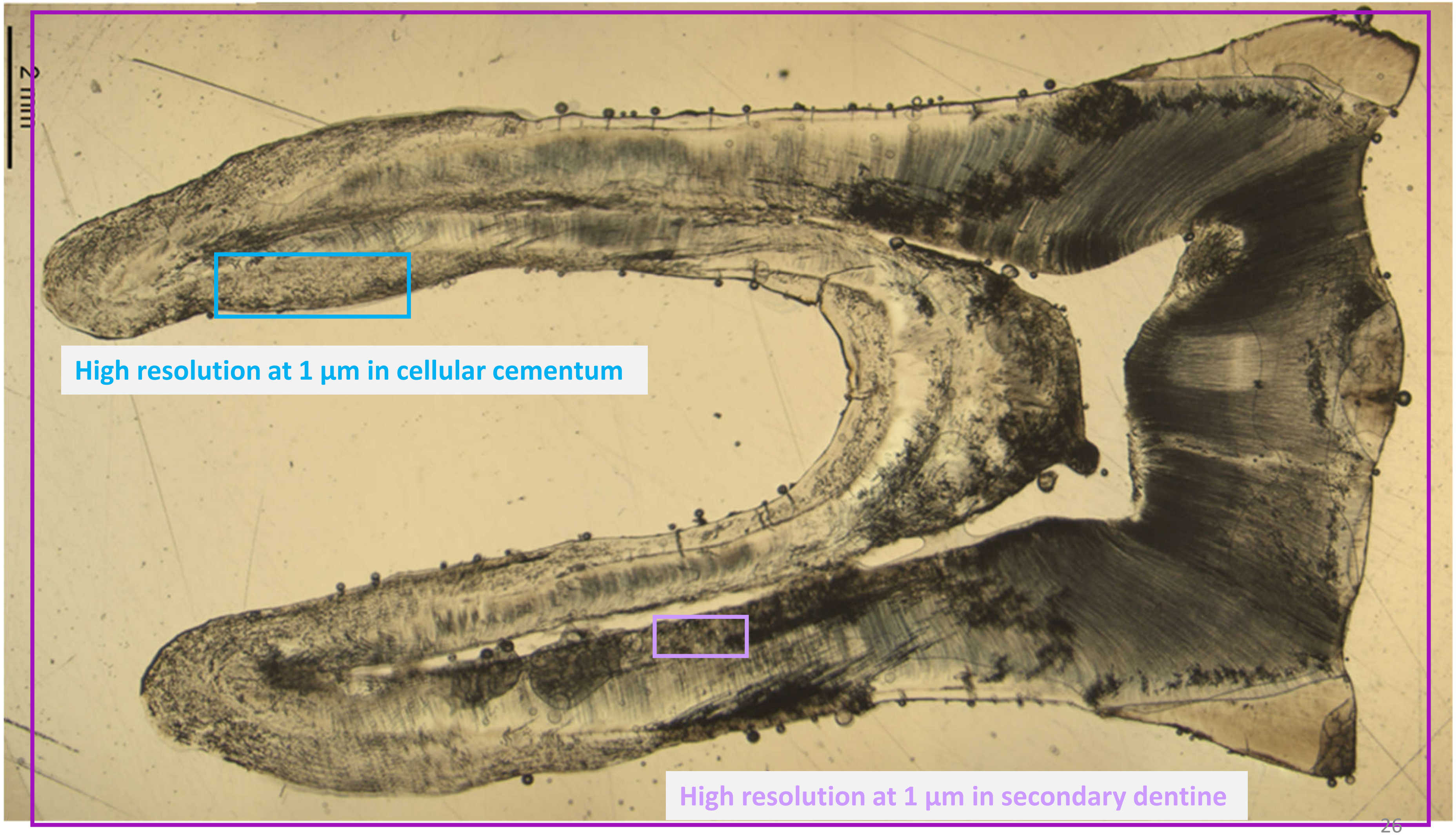

Odense 533 LLM1

Overview at 10 µm

Gauss (0.9x0.9)

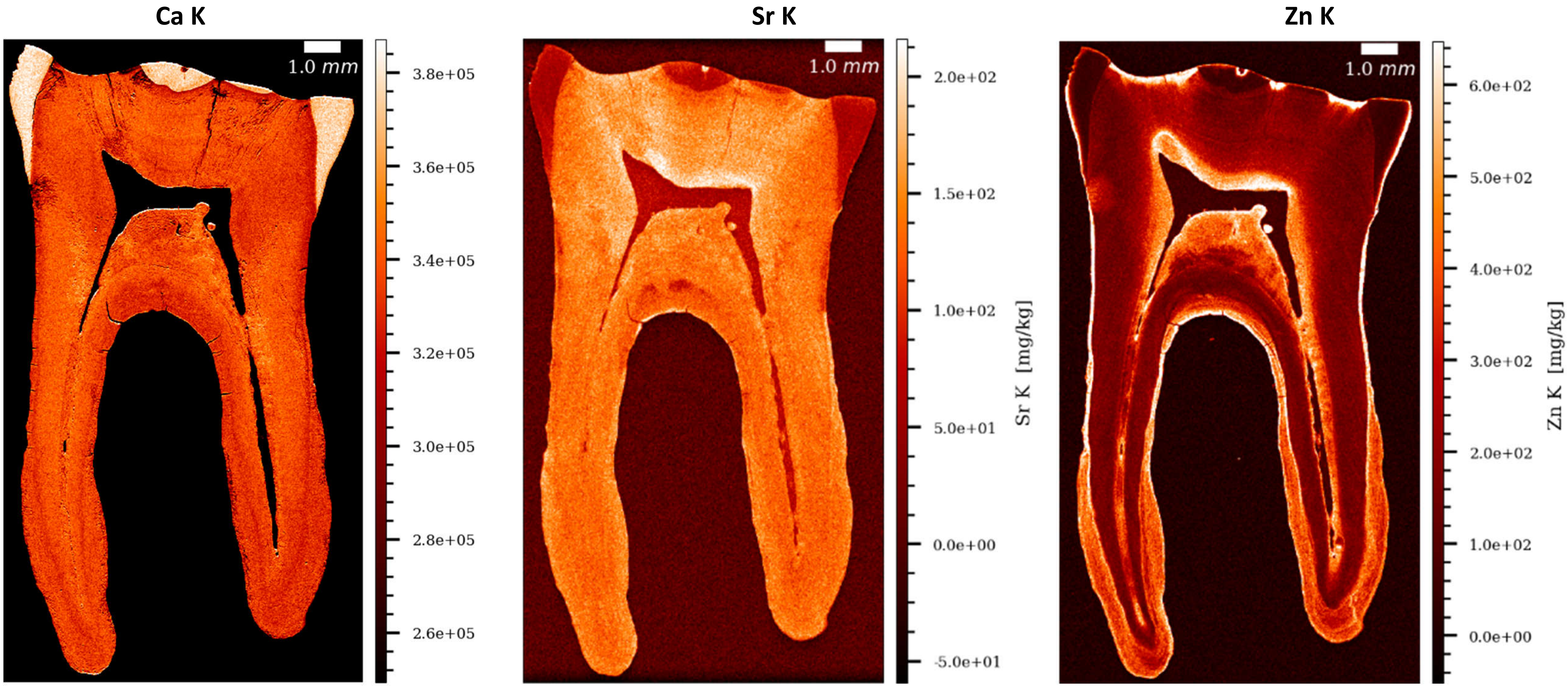

Odense 533 LLM1

Overview at 10 µm

Gauss (0.9x0.9)

Cu K

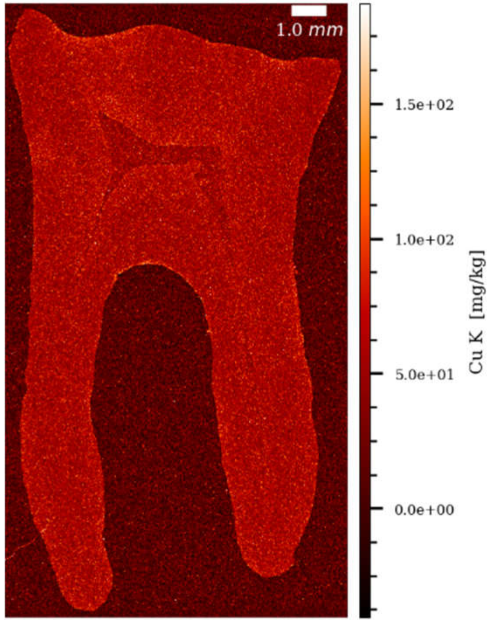

Fe K

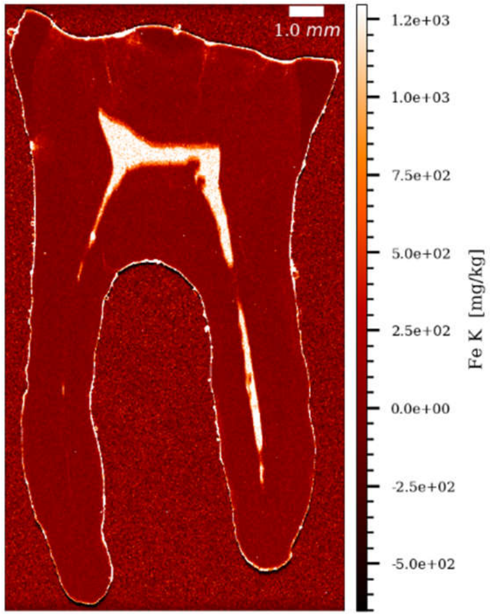

Mn K

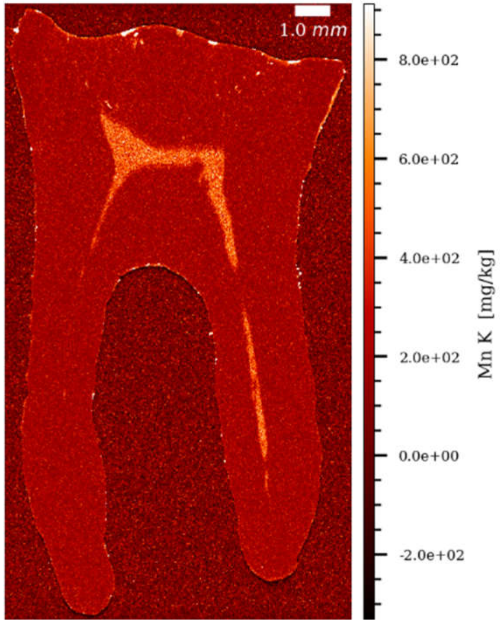

Odense 533 LLM1

High resolution at 1 µm in cellular cementum

Gauss (1x1)

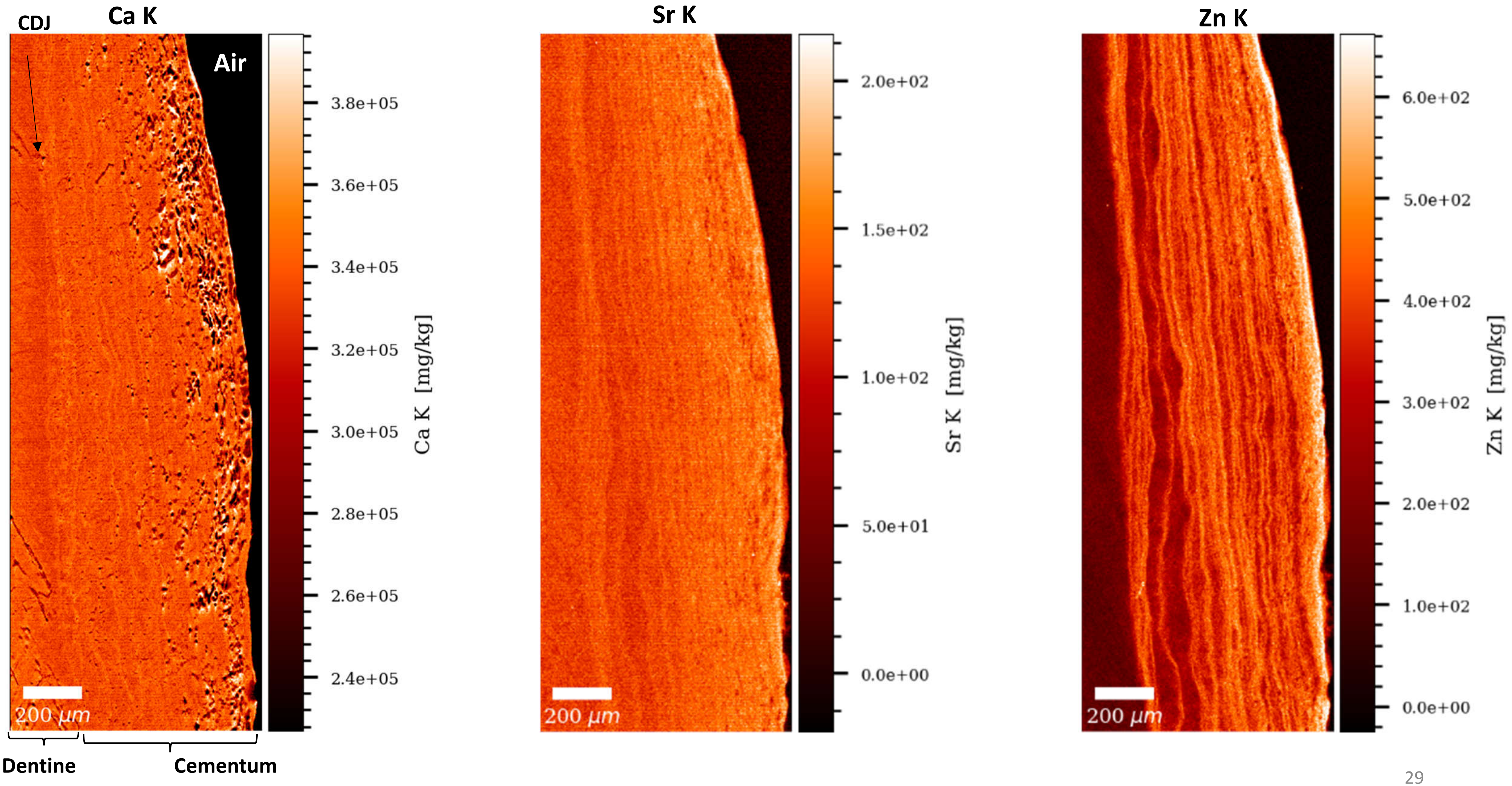

Odense 533 LLM1

High resolution at 1 µm in cellular cementum

Gauss (1x1)

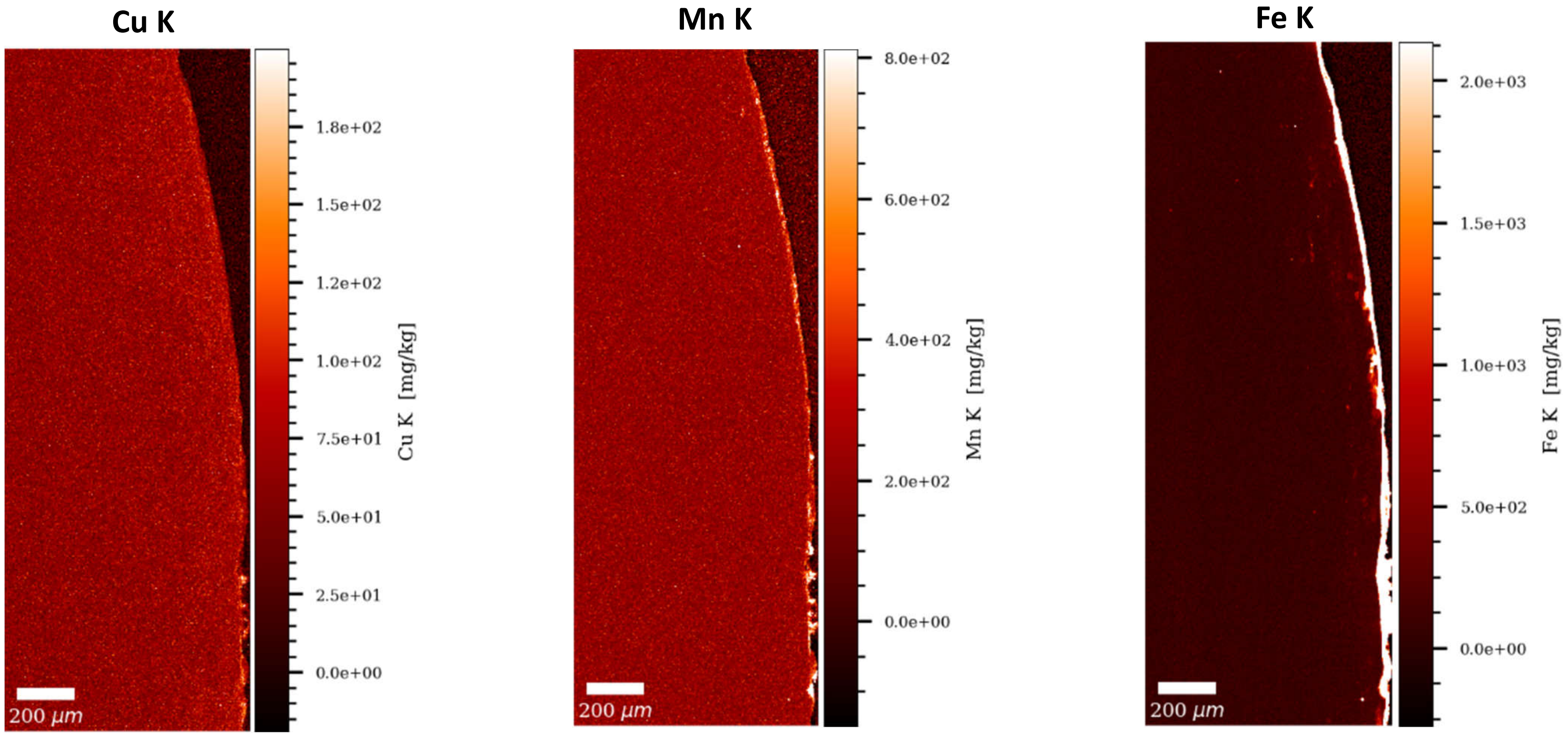

Odense 533 LLM1

High resolution at 1 µm in secondary dentine

Gauss (1x1)

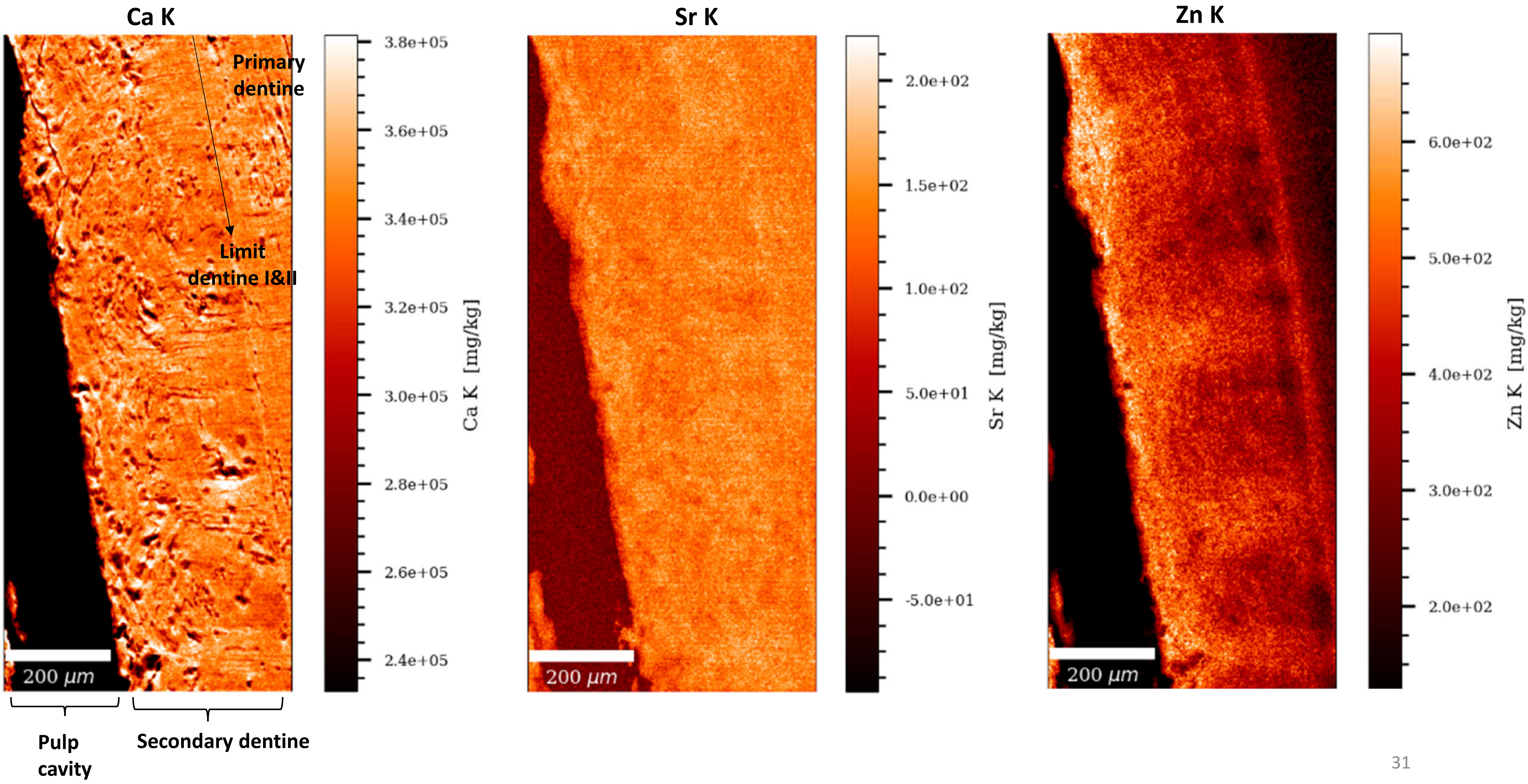

Odense 533 LLM1

High resolution at 1 µm in secondary dentine

Gauss (1x1)

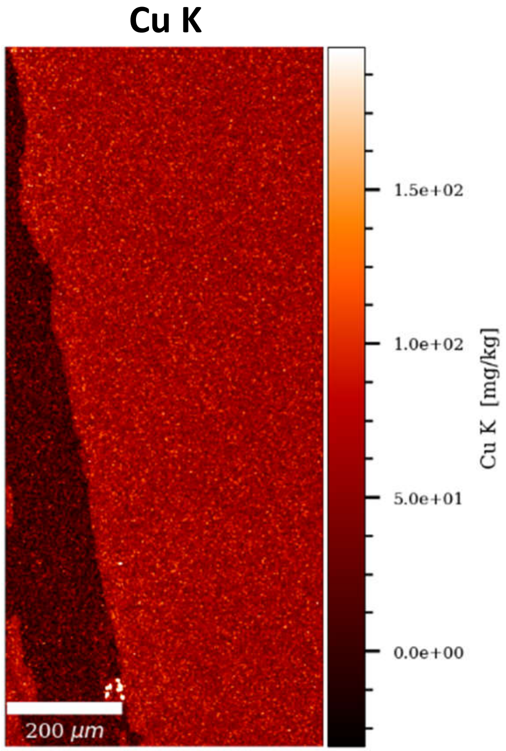

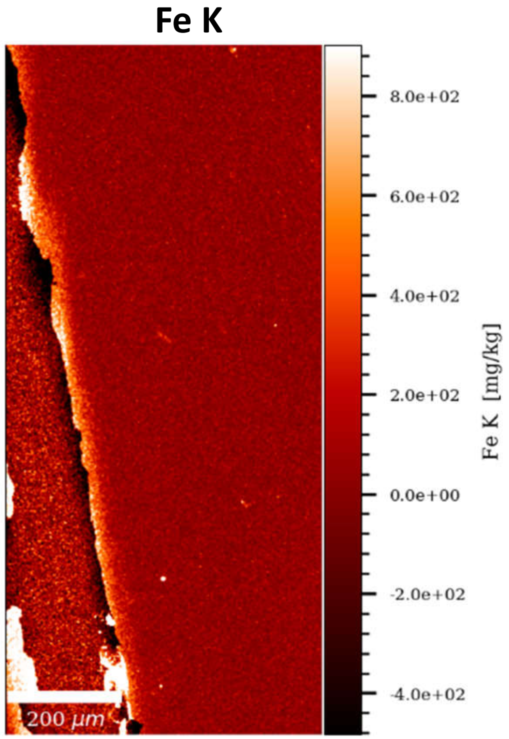

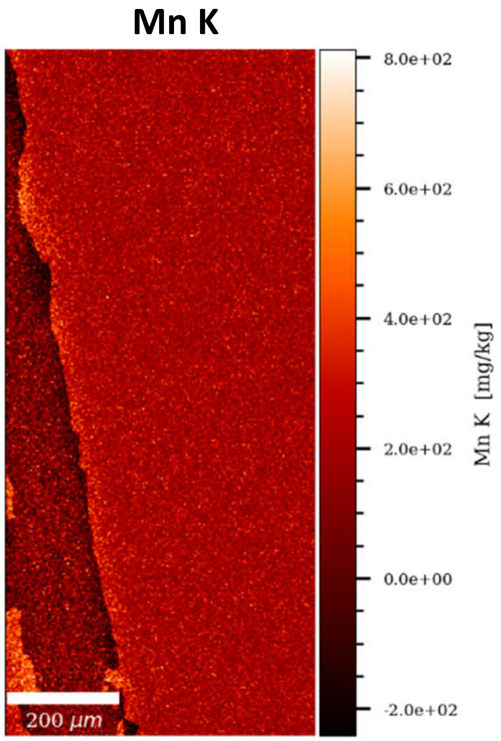

Odense – 896 LLC

♂ 35-45 yrs. 1183 – 1265 cal. CE

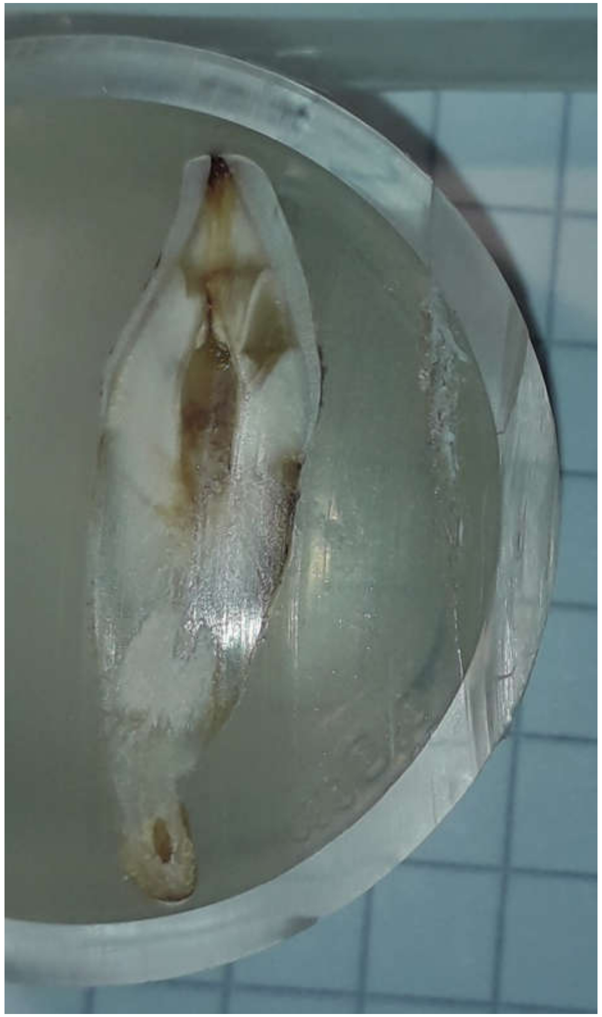

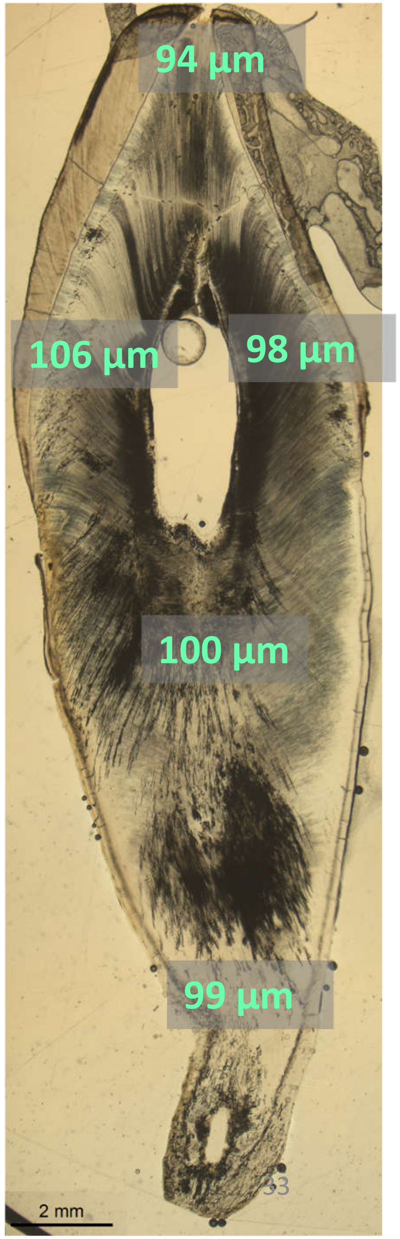

Average tooth section thickness (µm): 99.4

Odense 896 LLC

Scanning

Overview at 10 μm

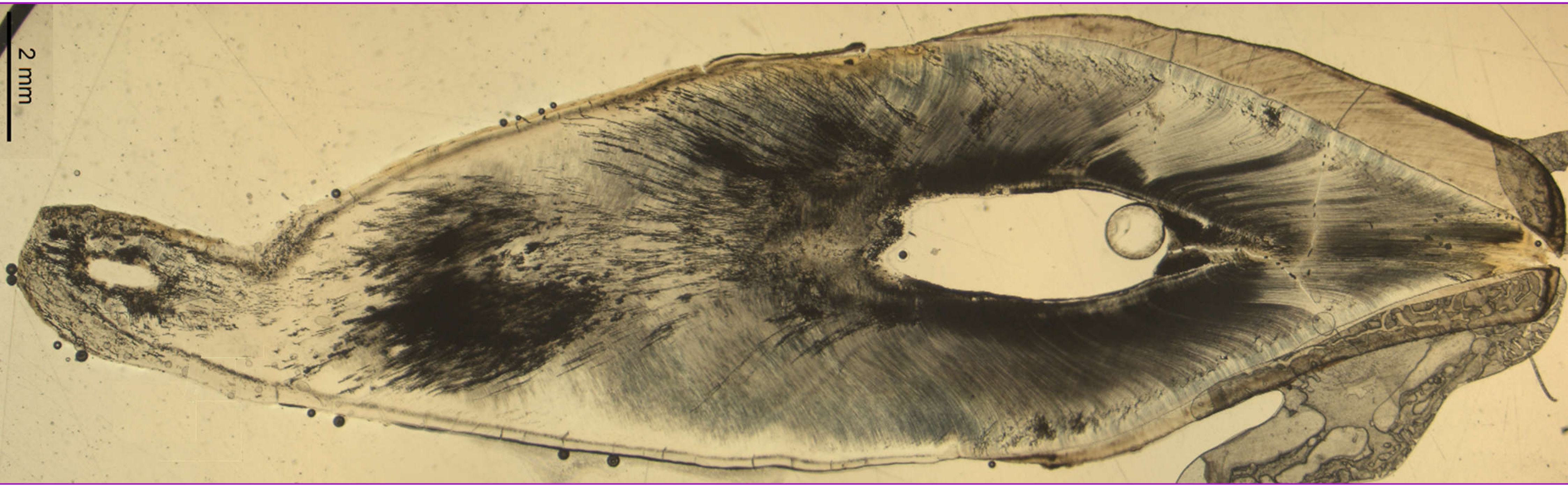

Odense 896 LLC

Overview at 10 µm

Gauss (0.8x0.8)

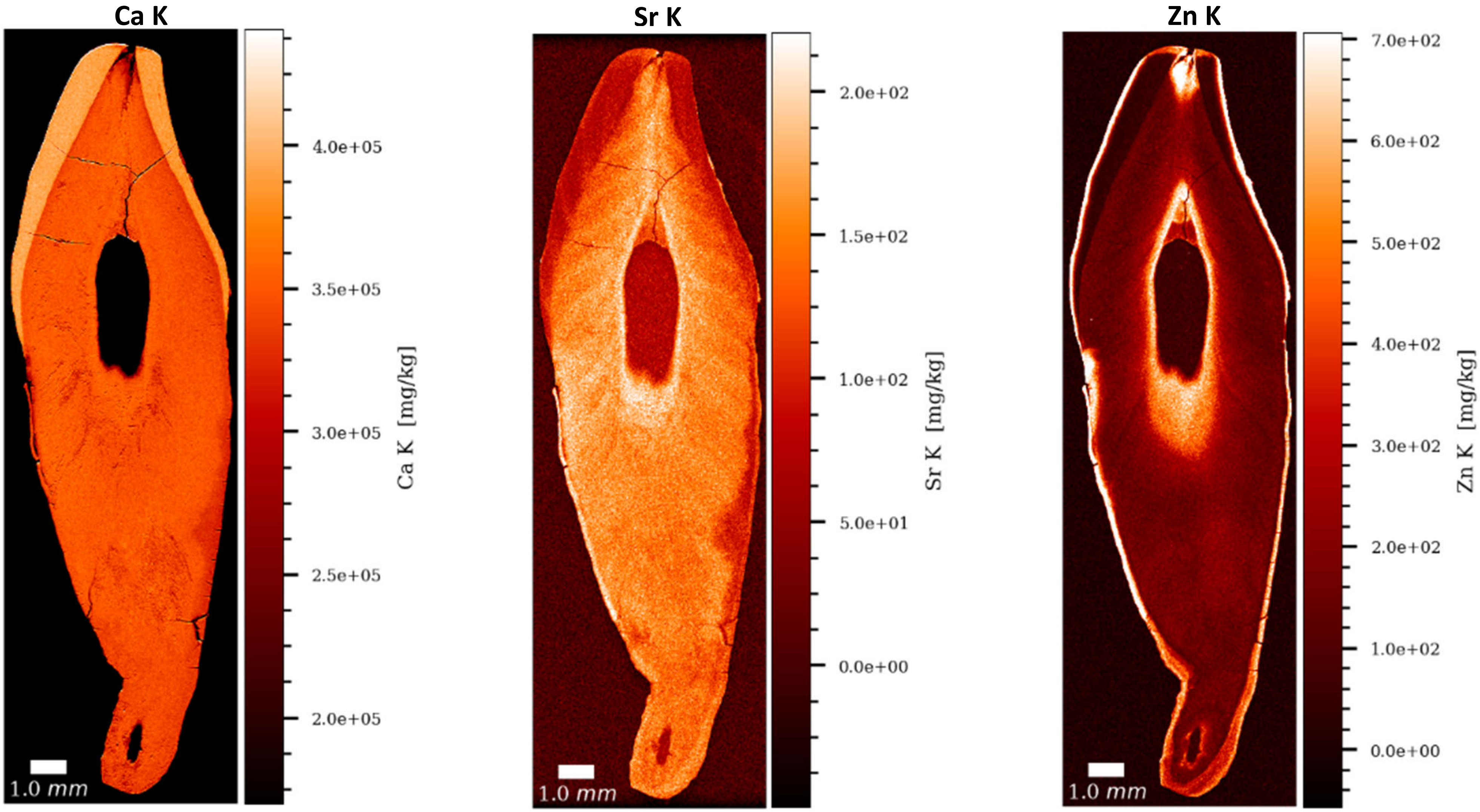

Odense 896 LLC

Overview at 10 µm

Gauss (0.8x0.8)

Cu K

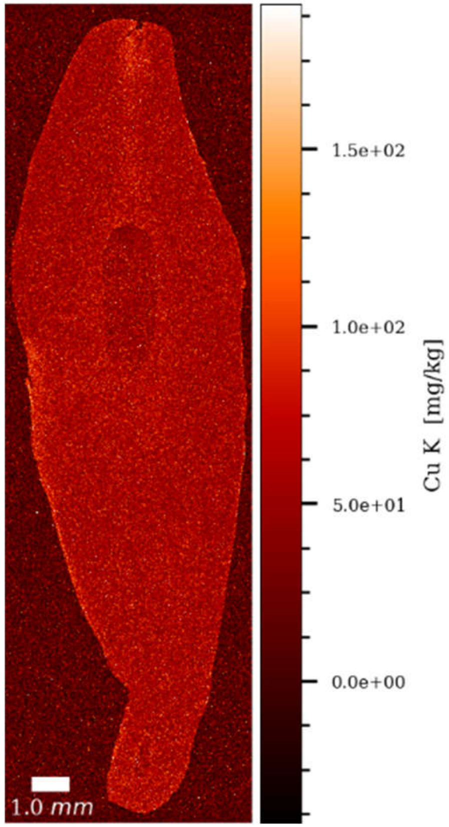

Fe K

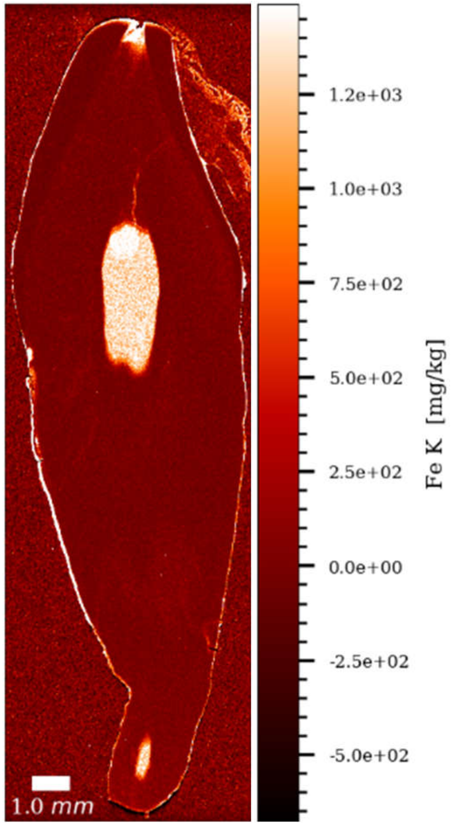

Mn K

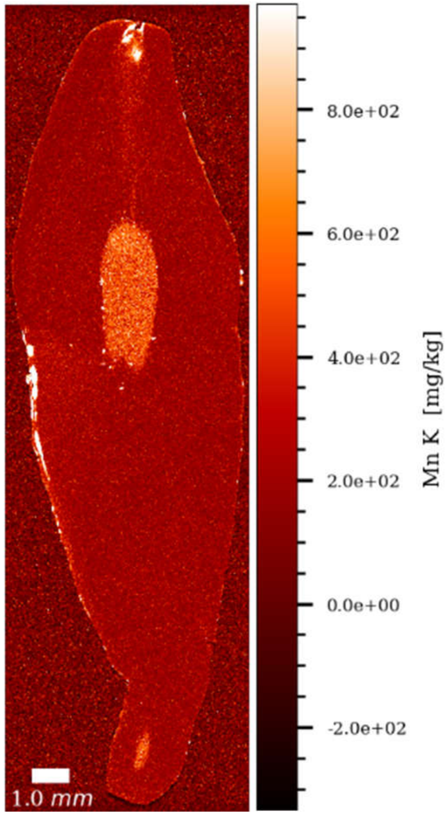

Odense – 896 LLM1

♂ 35-45 yrs. 1183 – 1265 cal. CE

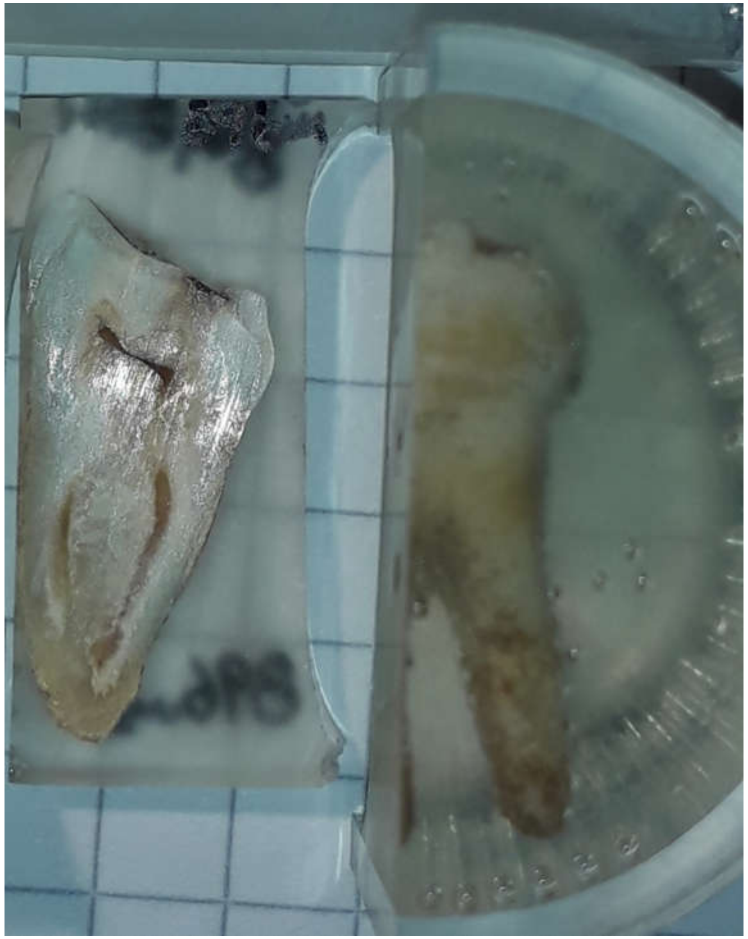

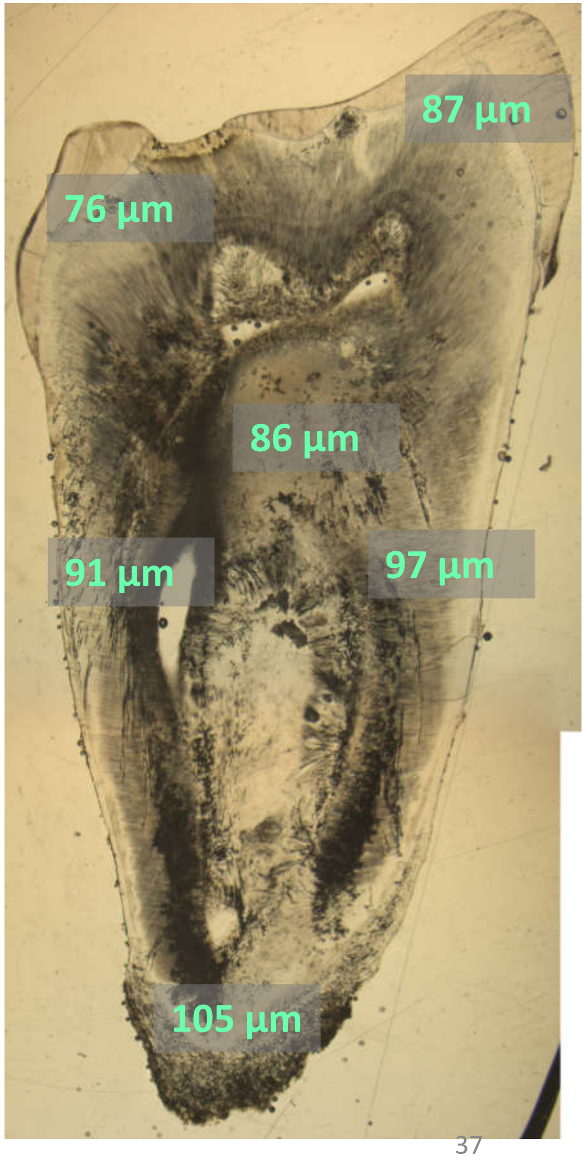

Average tooth section thickness (µm): 99.4

Odense 896 LLM1

Scanning

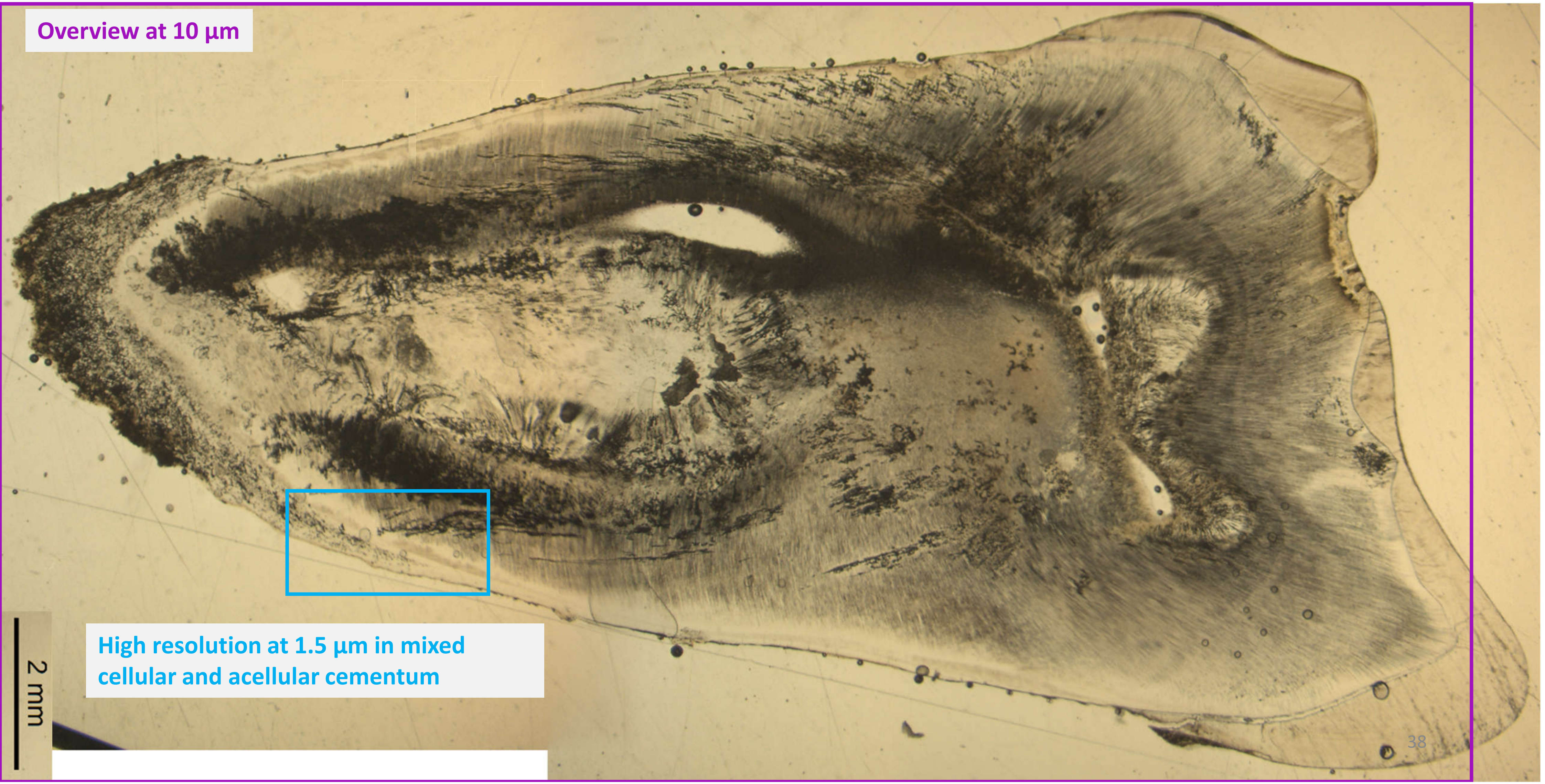

Odense 896 LLM1

Overview at 10 µm

Gauss (1x1)

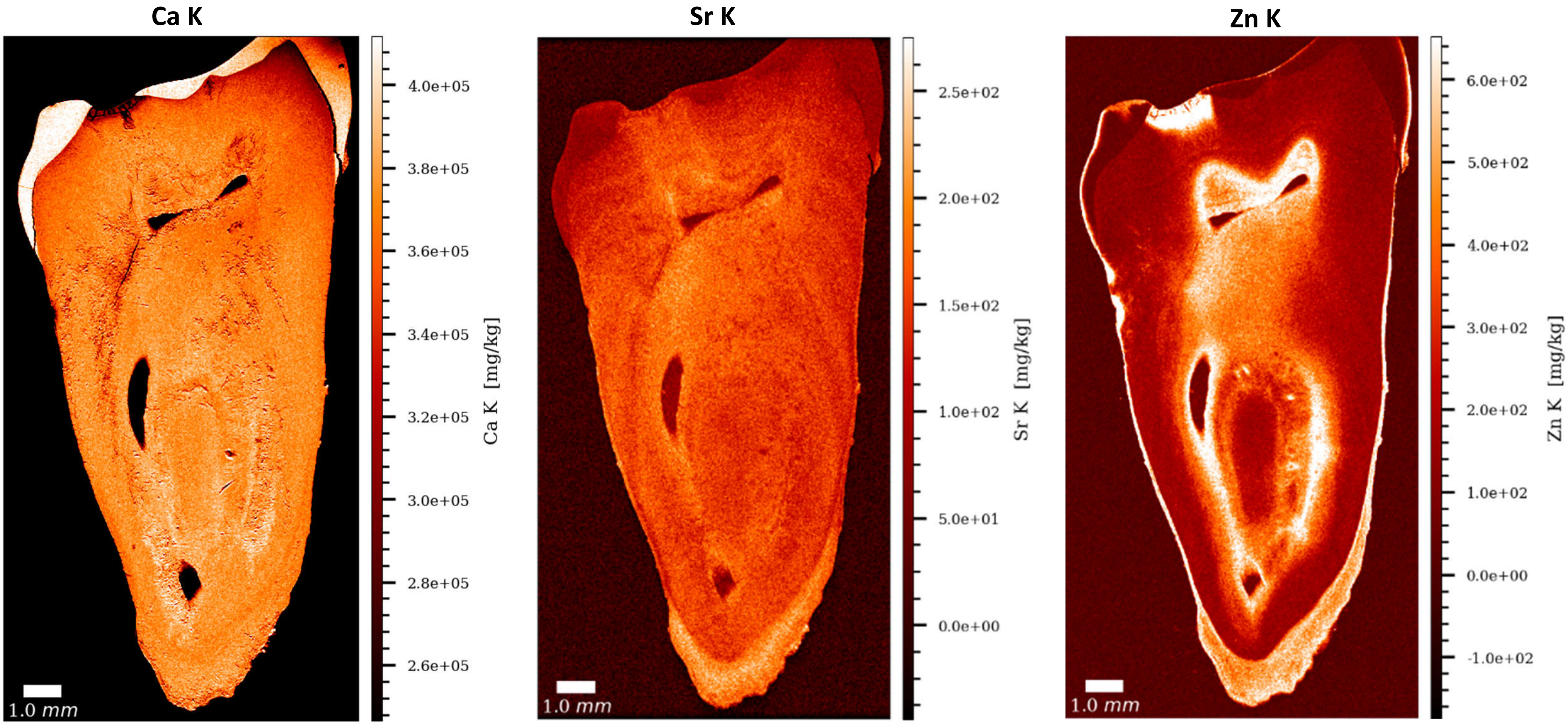

Odense 896 LLM1

Overview at 10 µm

Gauss (1x1)

Cu K

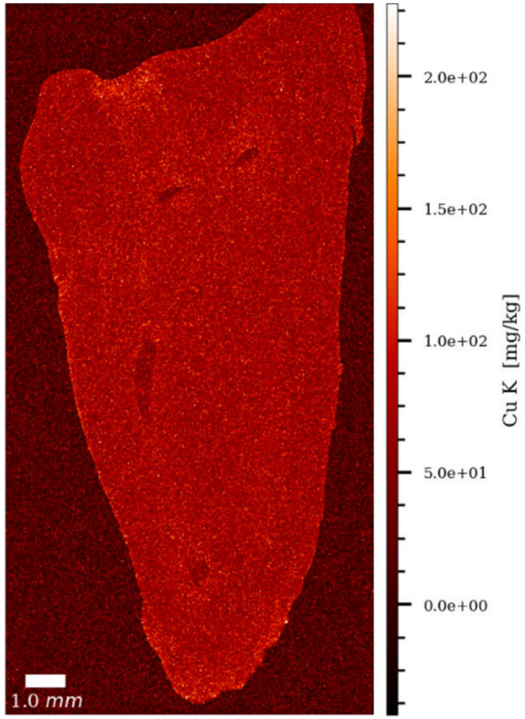

Fe K

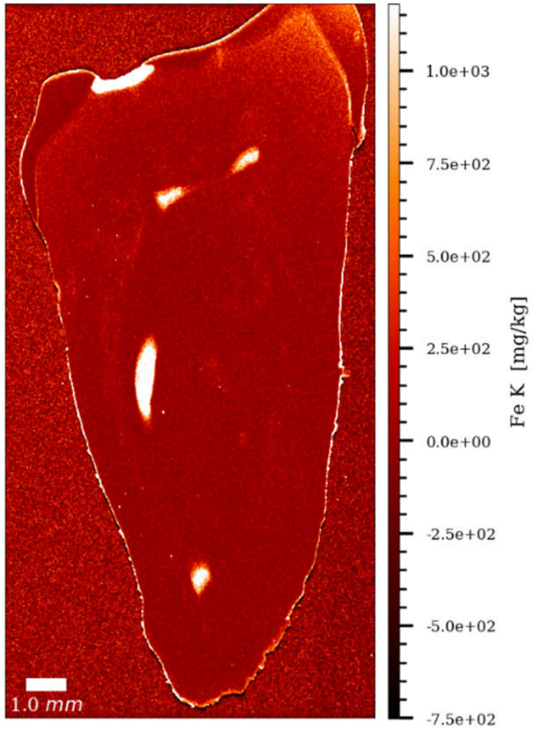

Mn K

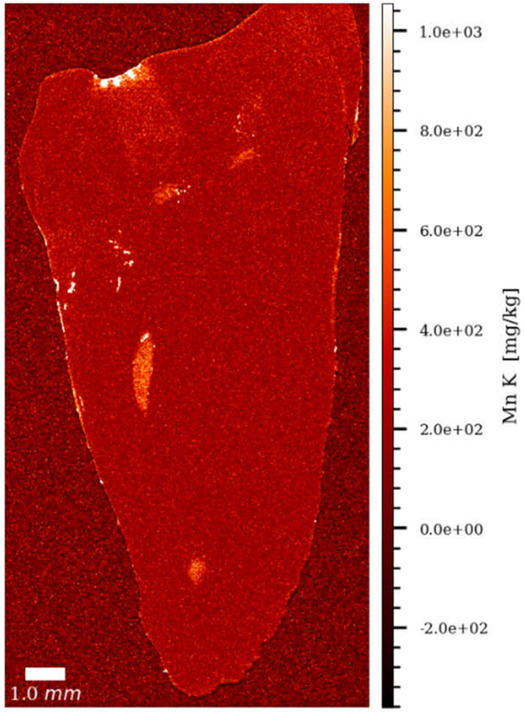

Odense 896 LLM1

High resolution at 1.5 µm

Gauss (1x1)

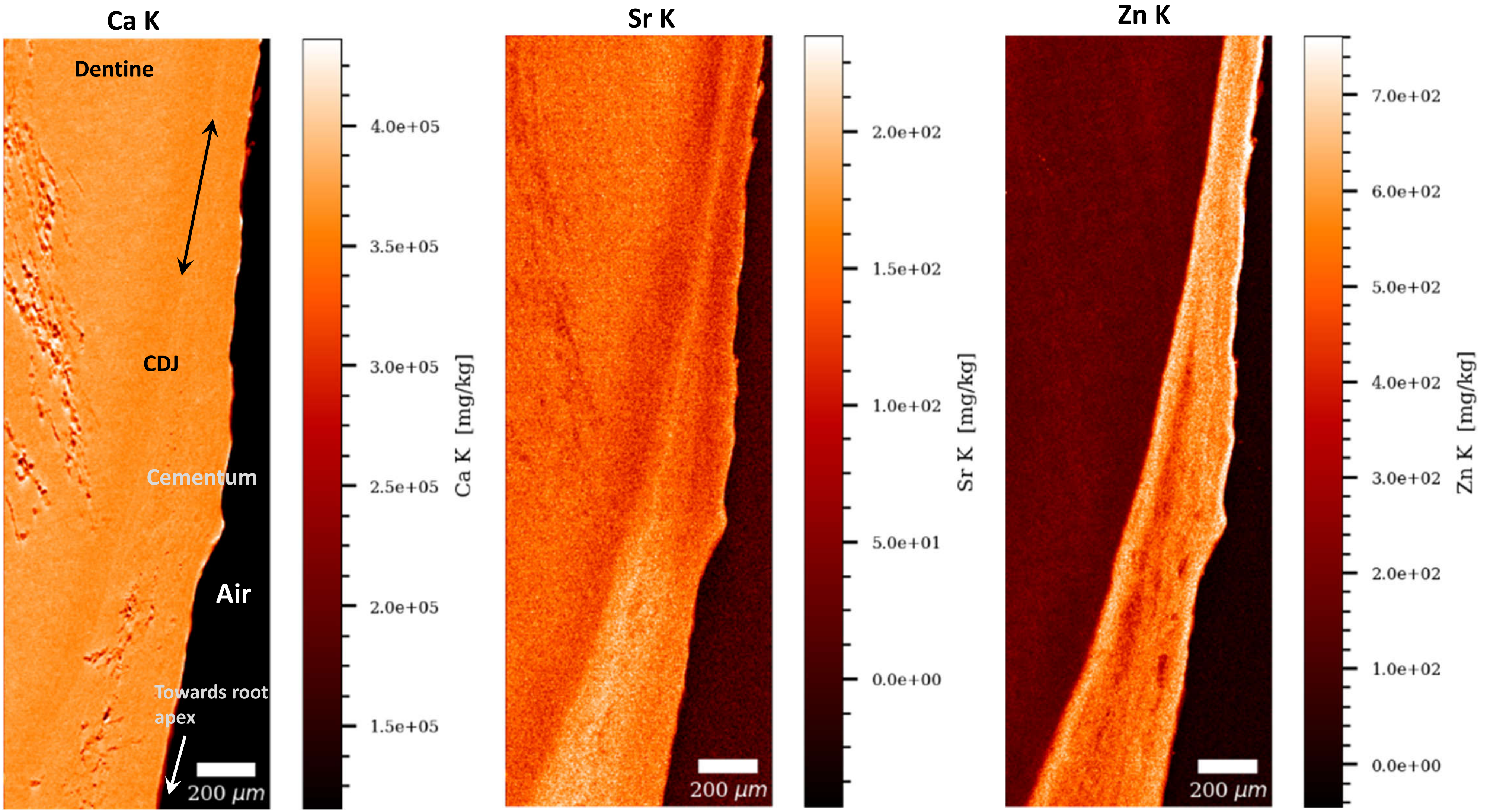

Odense 896 LLM1

High resolution at 1.5 µm

Gauss (1x1)

Cu K

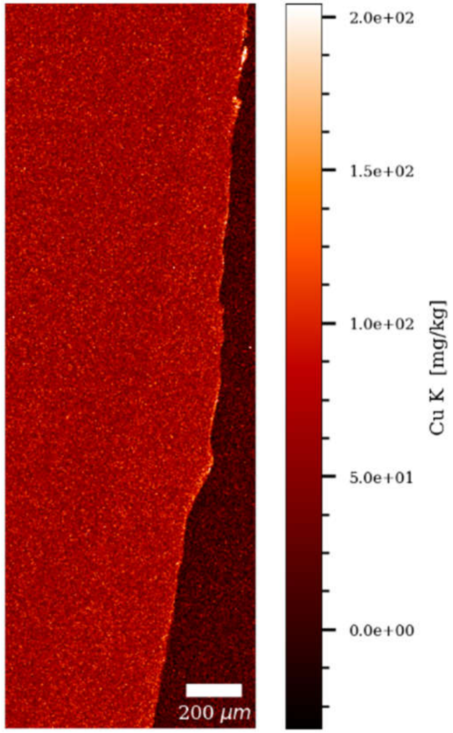

Fe K

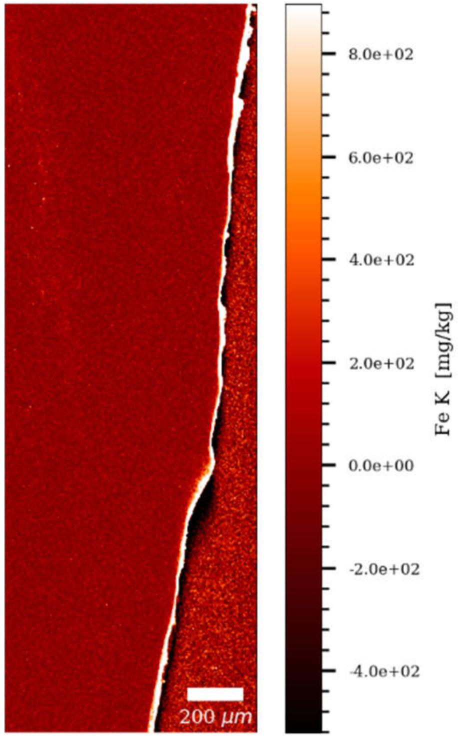

Mn K

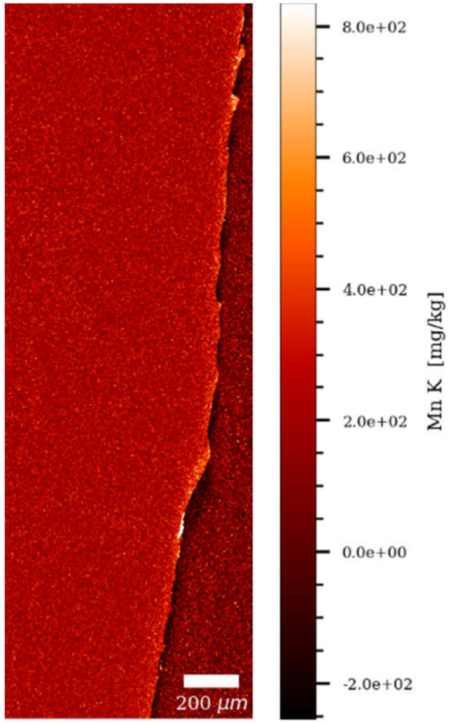

Odense – 914 ULM1

♀ 25-35 yrs. 1459 – 1566 cal. CE

Average tooth section thickness (µm): 95.9

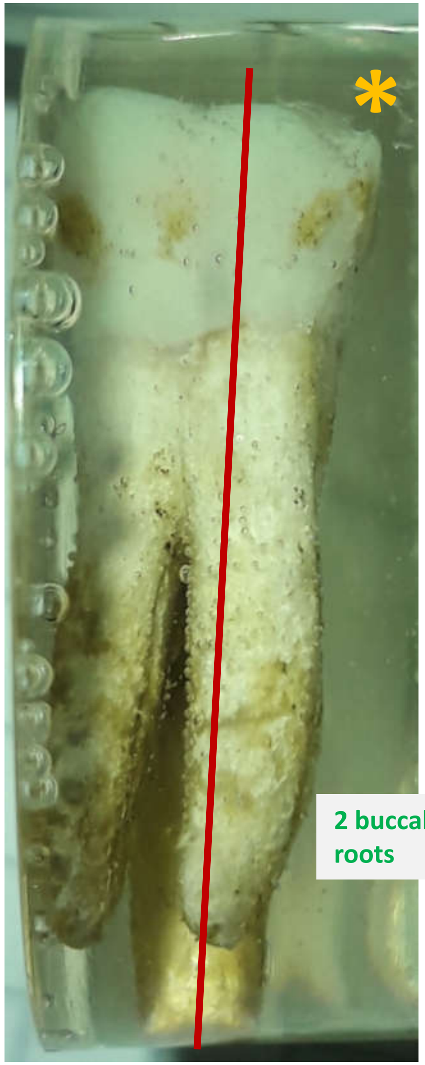

2 buccal roots

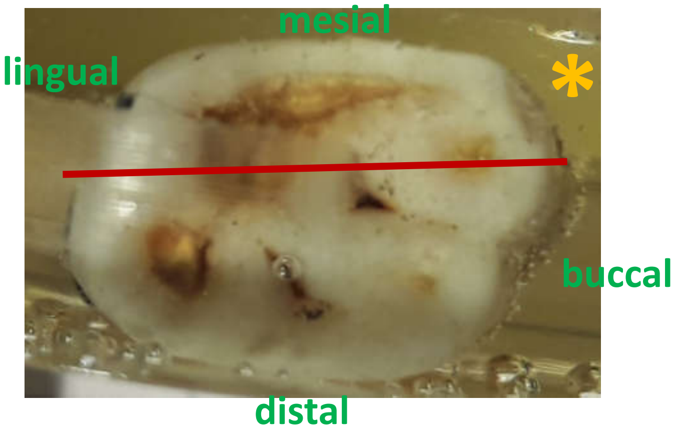

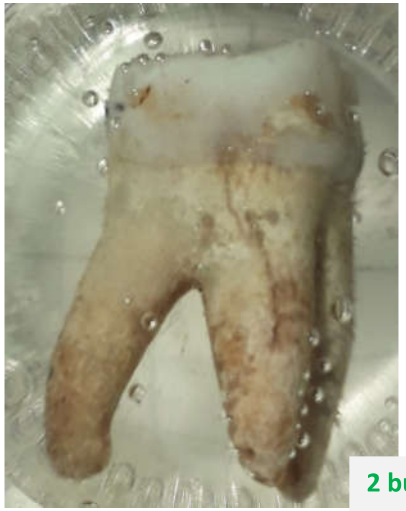

2 buccal roots

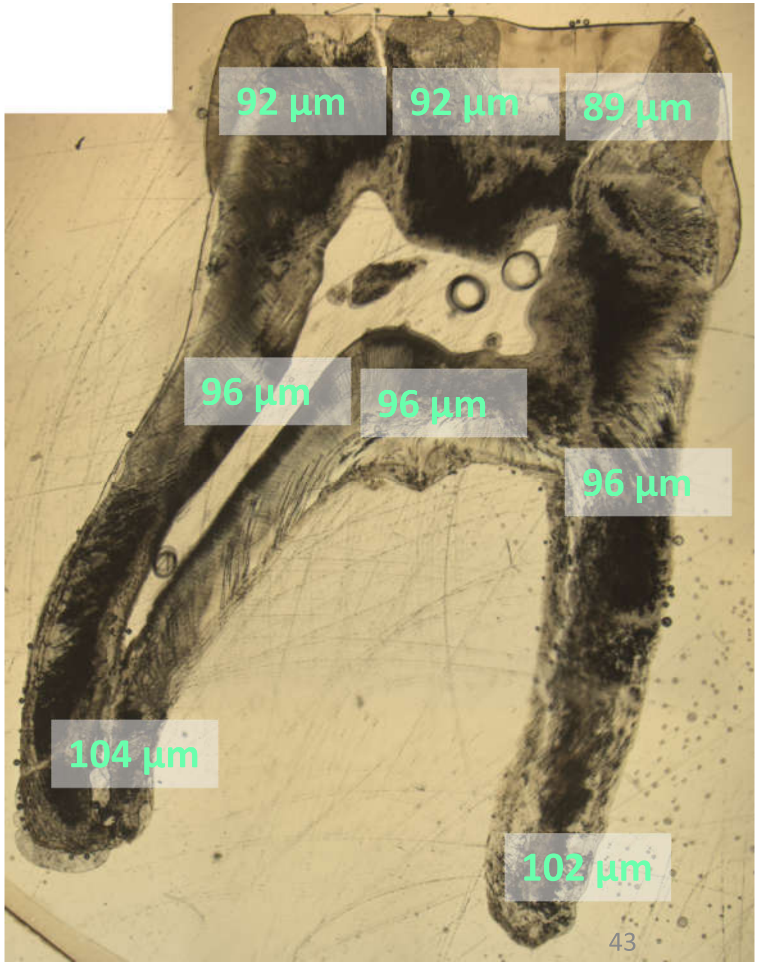

Odense 914 ULM1

Scanning

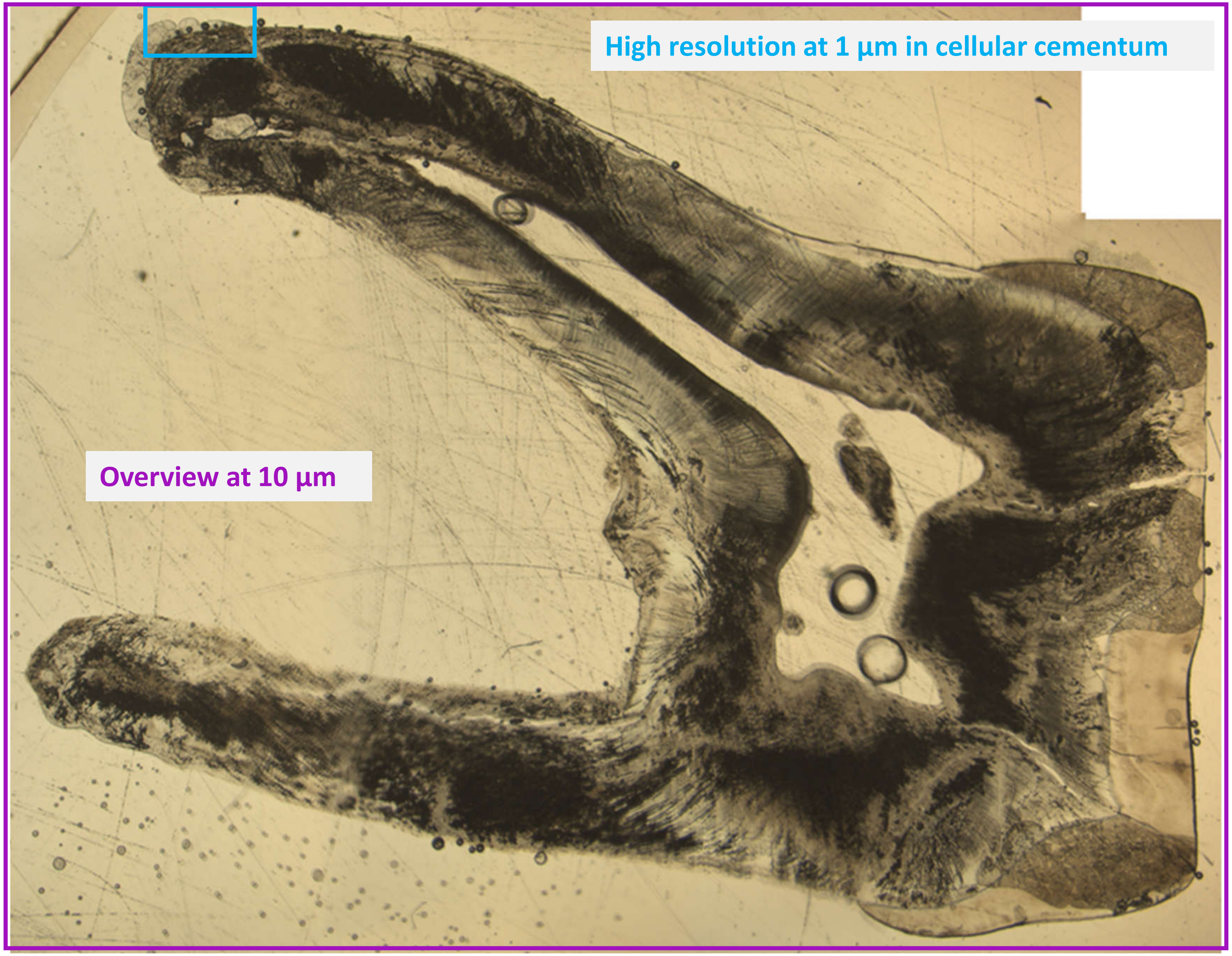

Odense 914 ULM1

Overview at 10 µm

Gauss (1x1)

Ca K

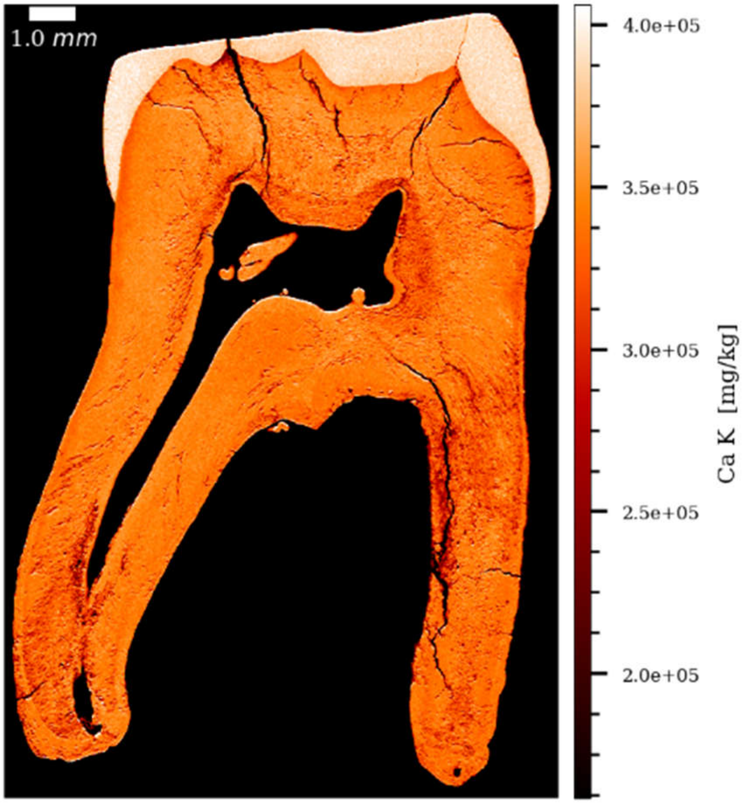

Sr K

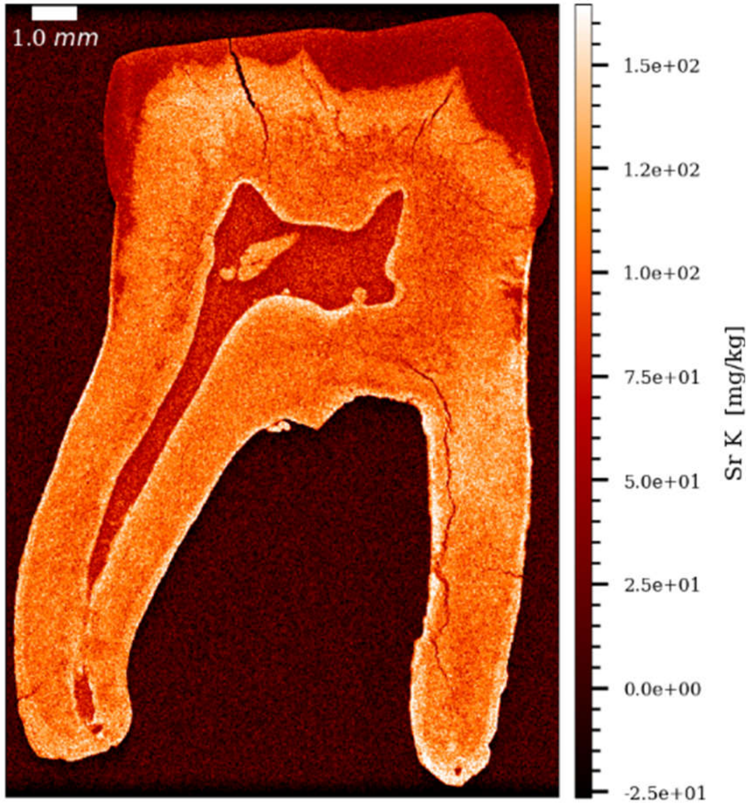

Zn K

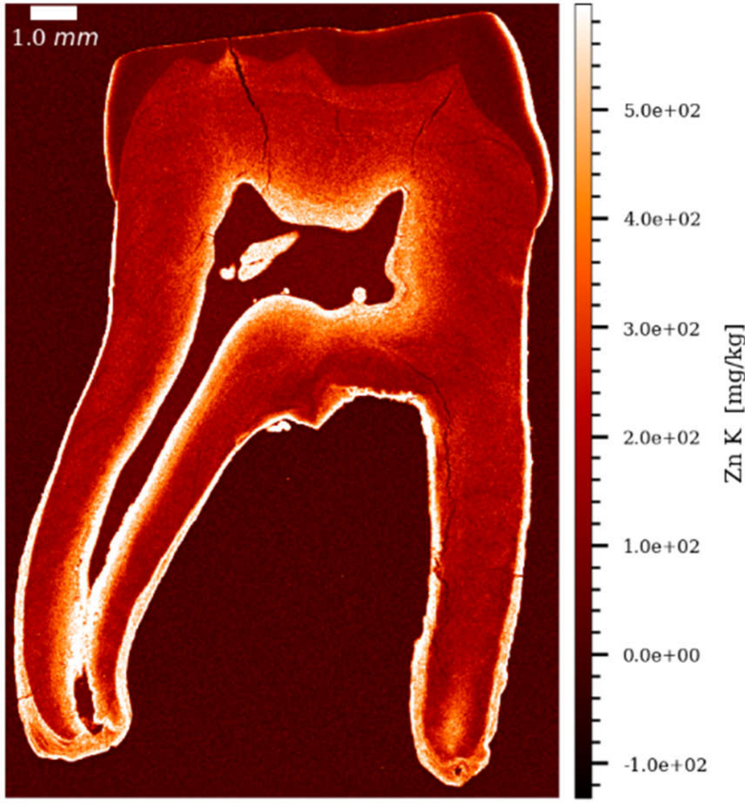

Odense 914 ULM1

Overview at 10 µm

Gauss (1x1)

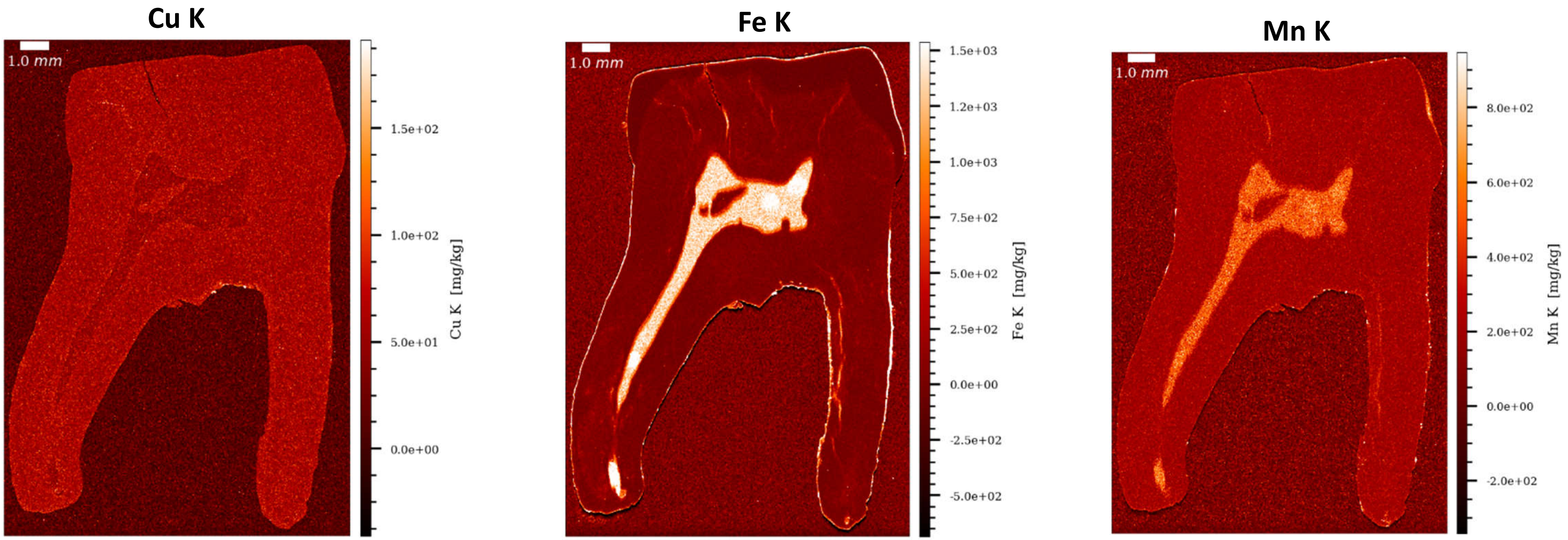

Odense 914 ULM1

Overview at 10 µm

Uncalibrated data (arbitrary units)

Gauss (1x1)

Br K

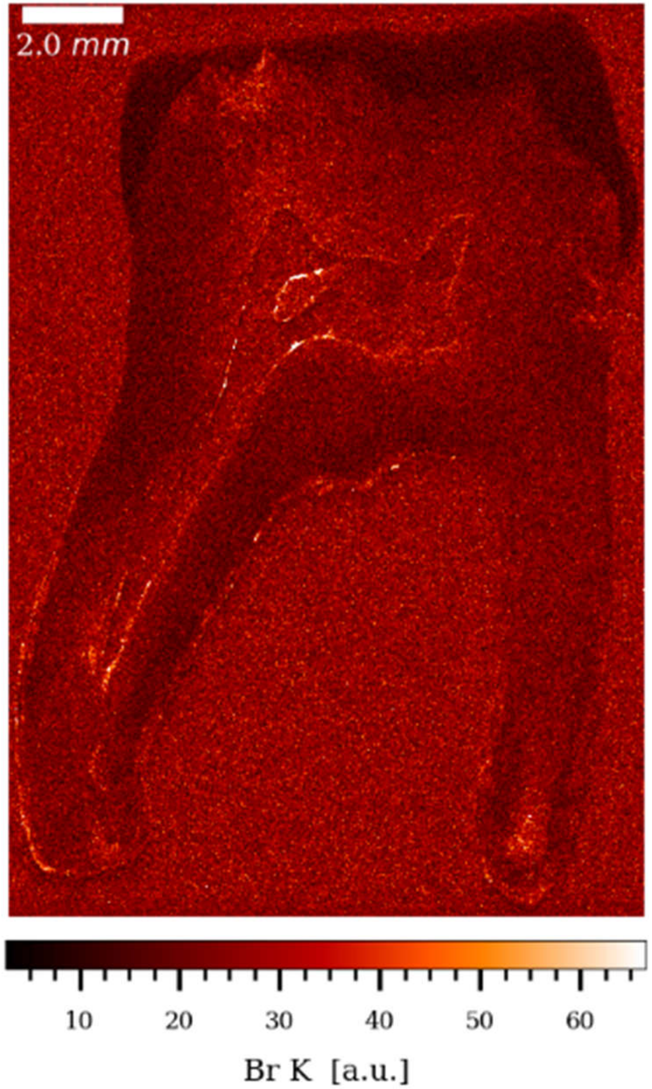

Pb L

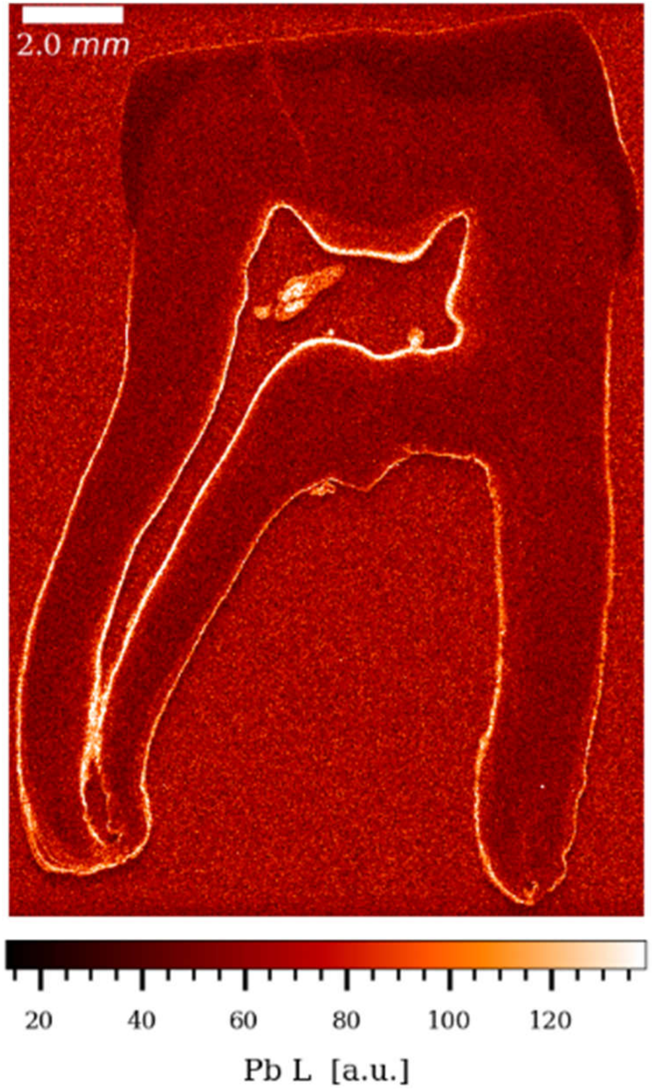

Odense 914 ULM1

High resolution at 1 µm

Gauss (0.9x0.9)

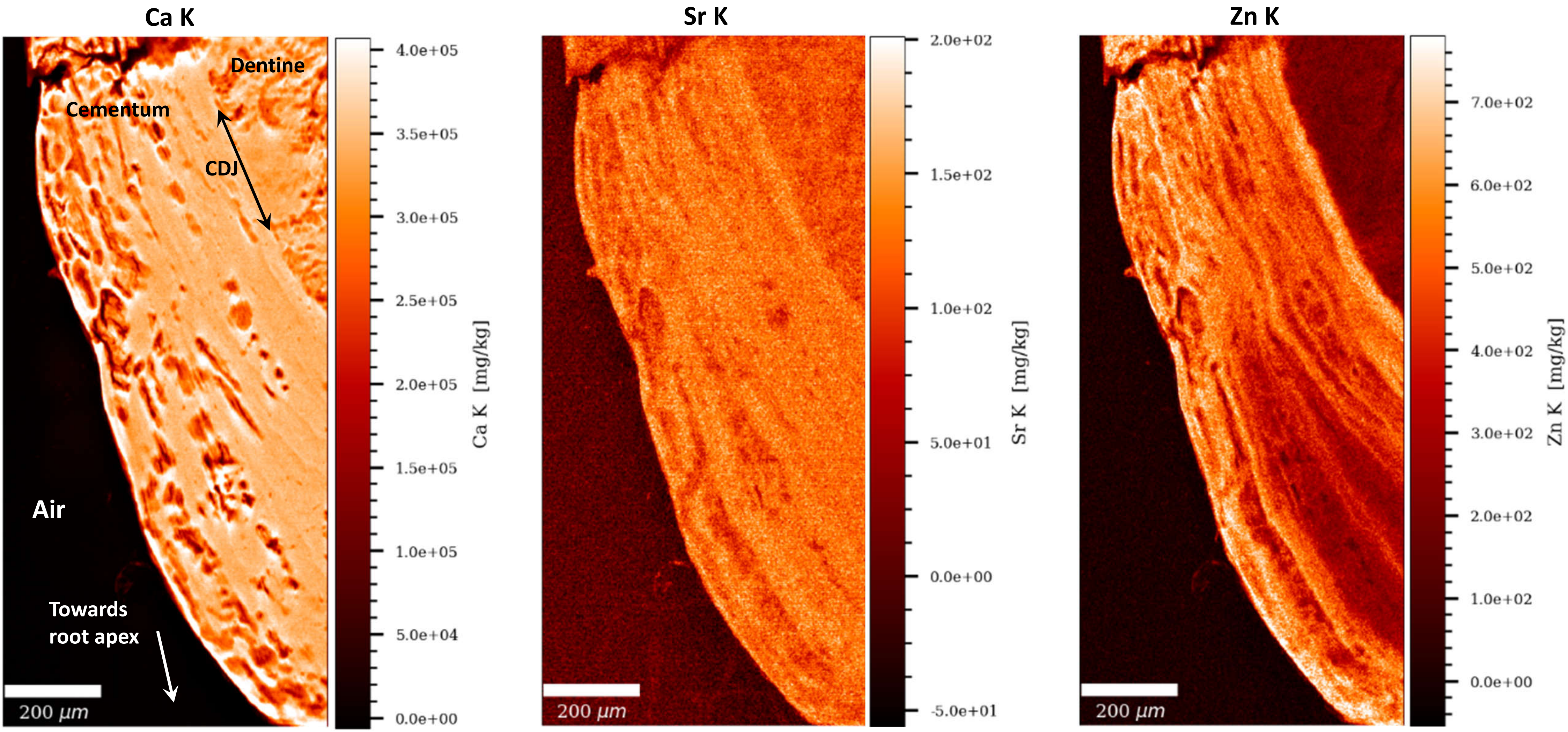

Odense 914 ULM1

High resolution at 1 µm

Gauss (0.9x0.9)

Cu K

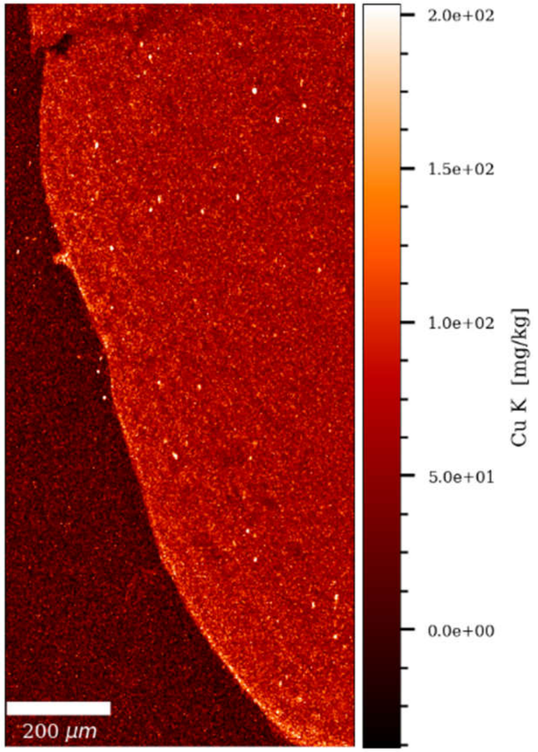

Fe K

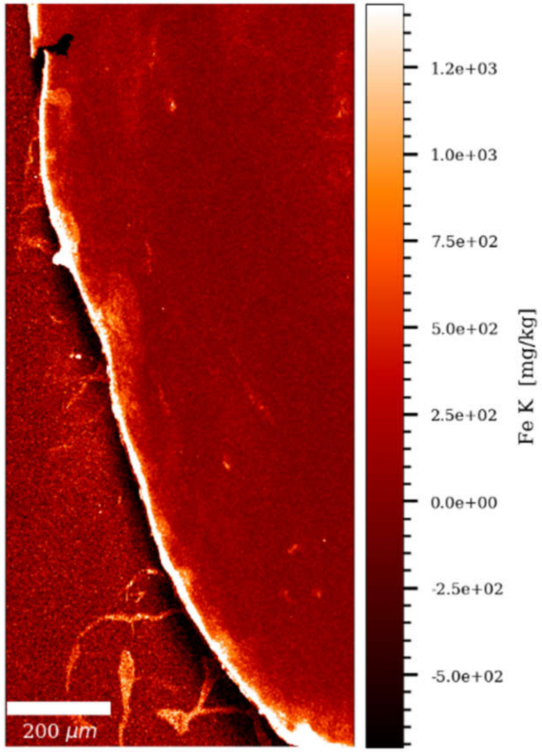

Mn K

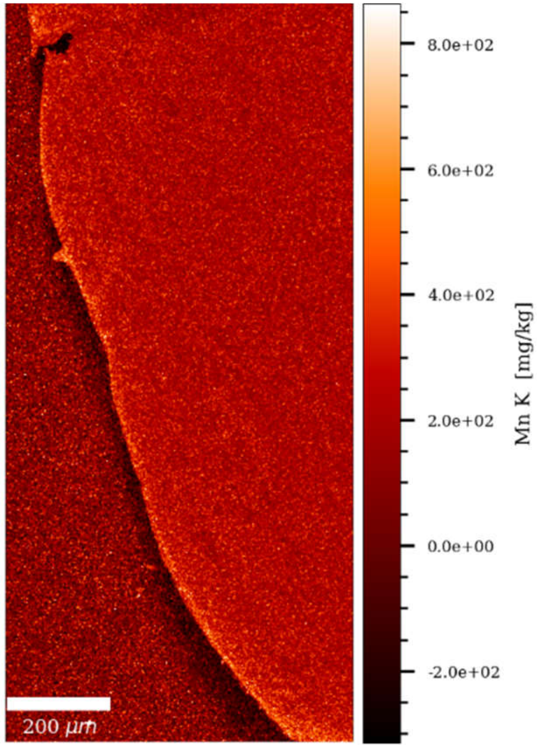

Odense – 1149 URC

♀ 20-30 yrs. 1301 – 1415 cal. CE

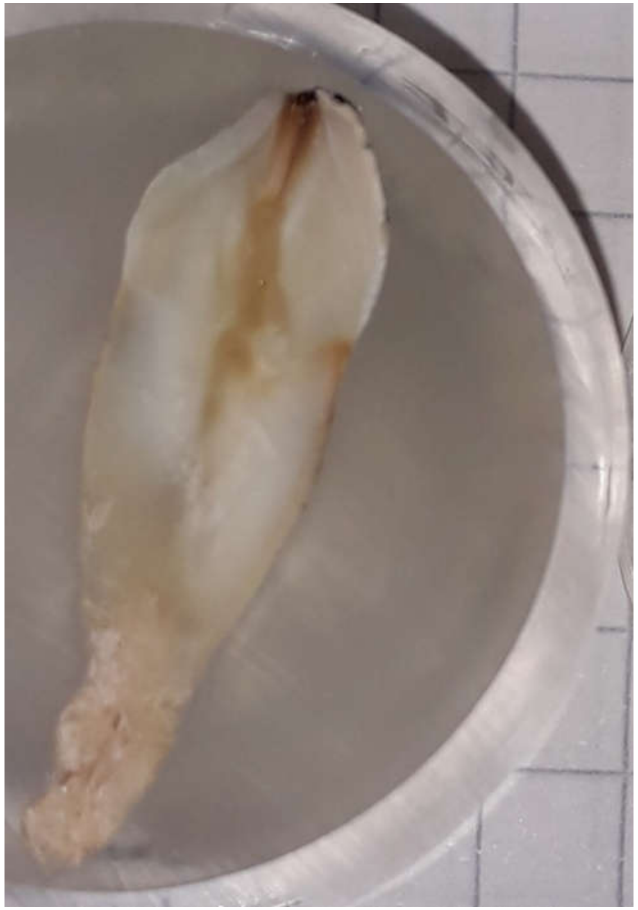

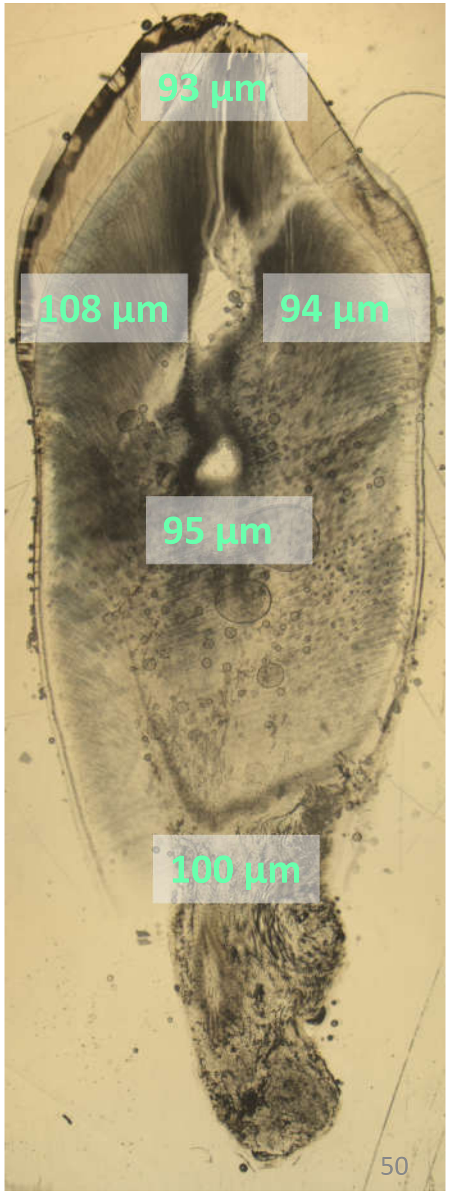

Average tooth section thickness (µm): 98.0

Odense 1149 URC

Scanning

Overview at 10 µm

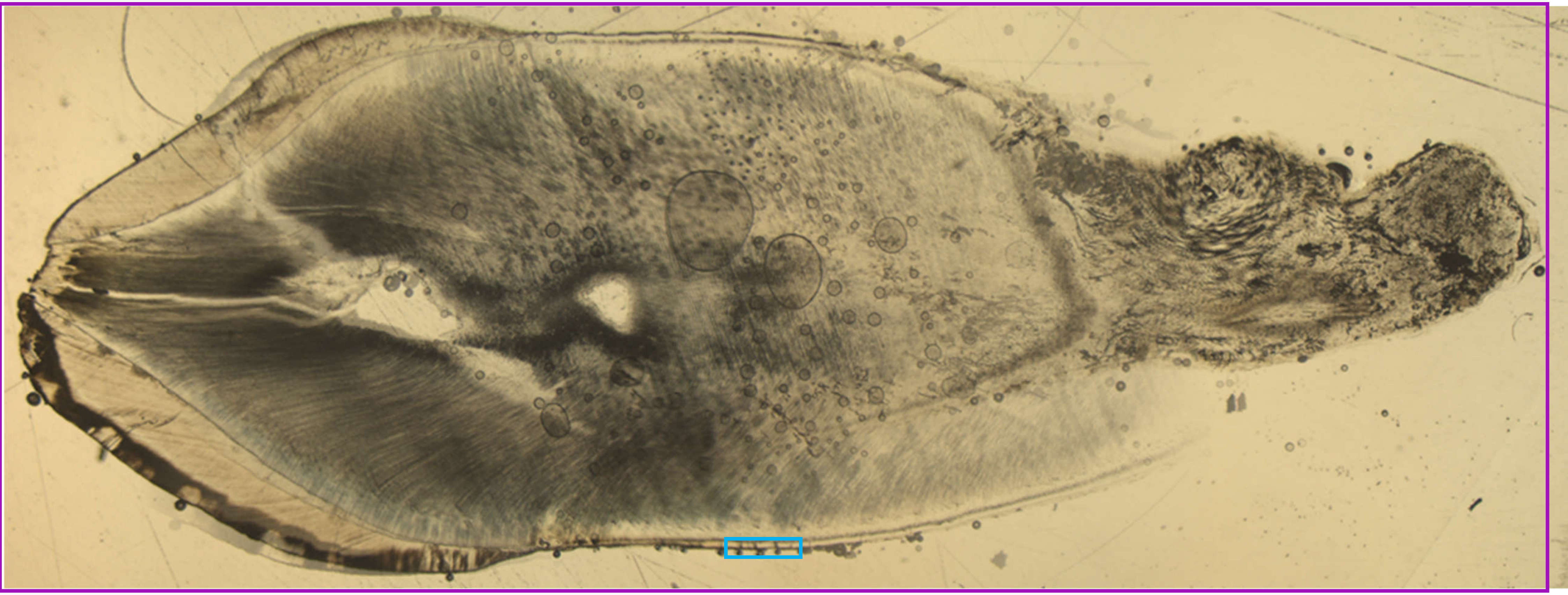

High resolution at 1 µm in acellular cementum

Odense 1149 URC

Overview at 10 µm

Gauss (1.2x1.2)

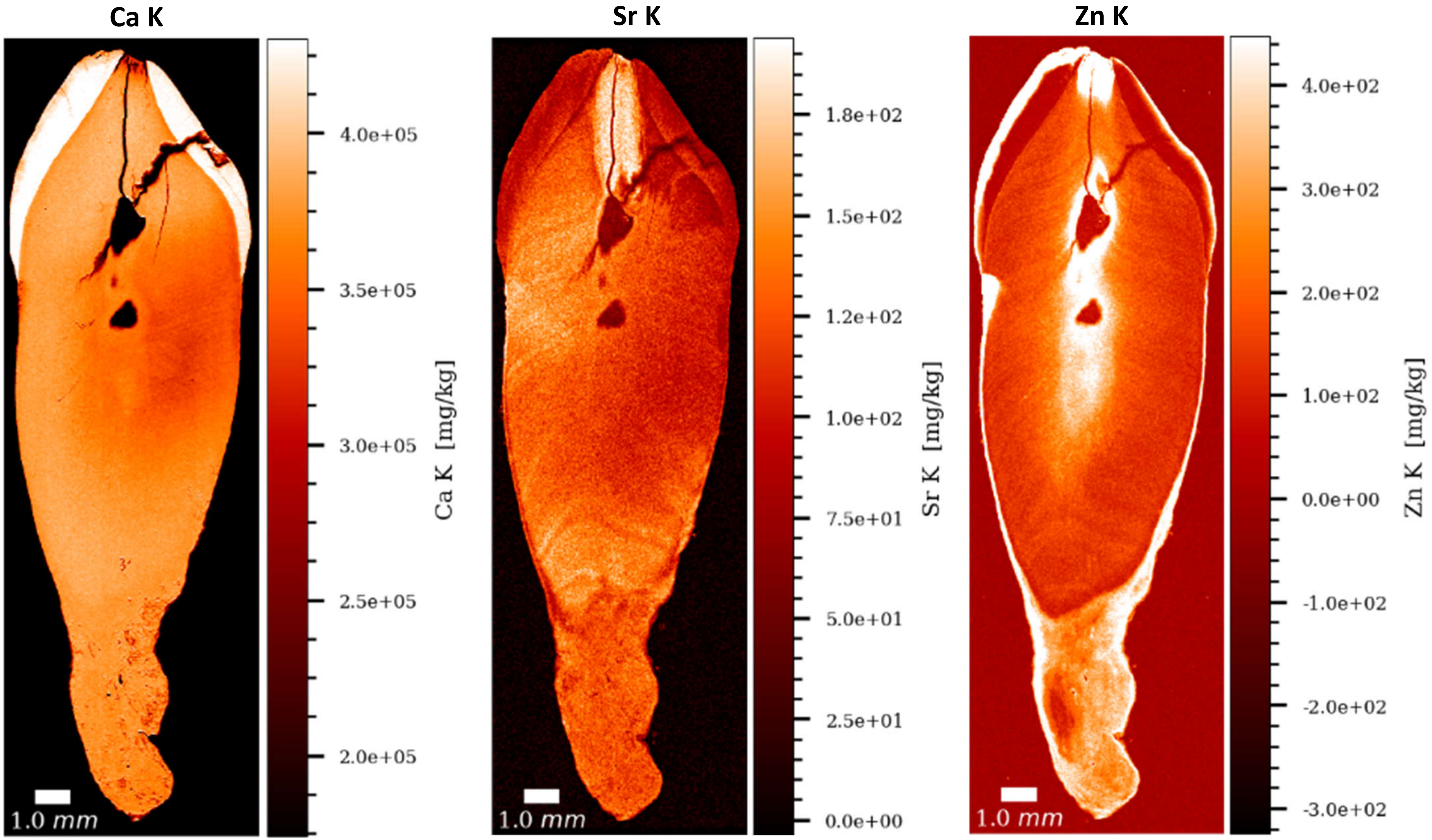

Odense 1149 URC

Overview at 10 µm

Gauss (1.2x1.2)

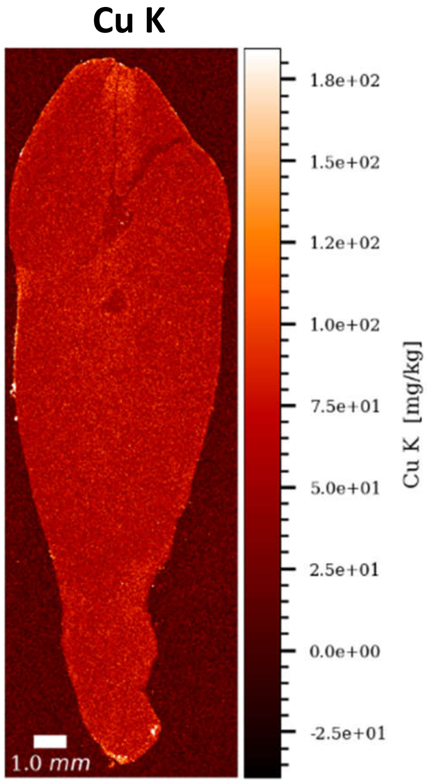

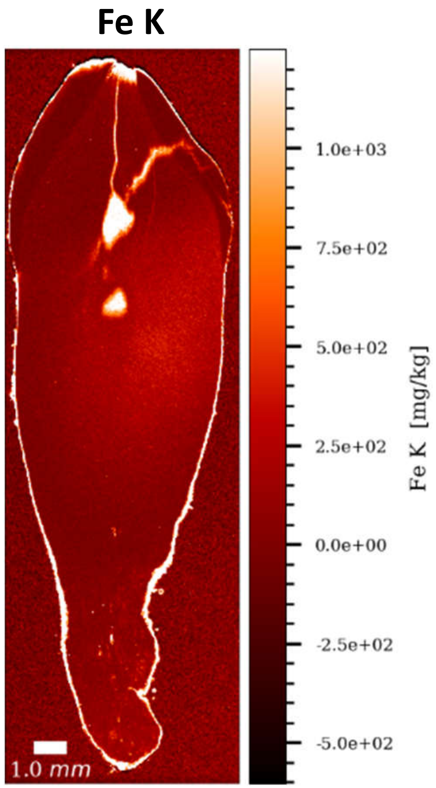

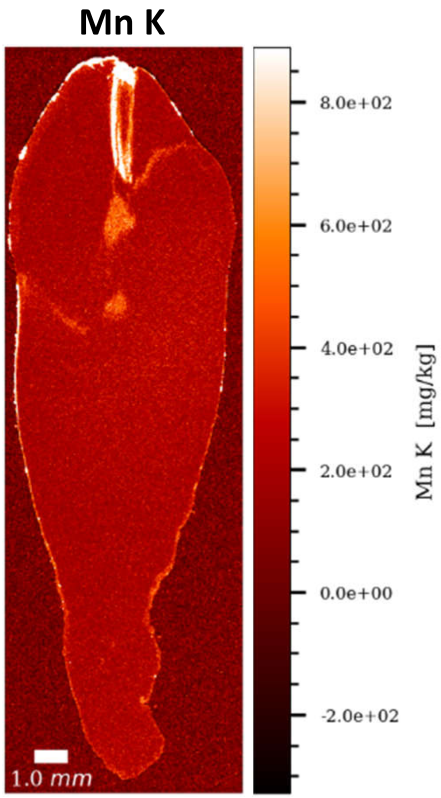

Odense 1149 URC

Overview at 10 µm

Uncalibrated data
(arbitrary units)

Gauss (1.2x1.2)

Br K

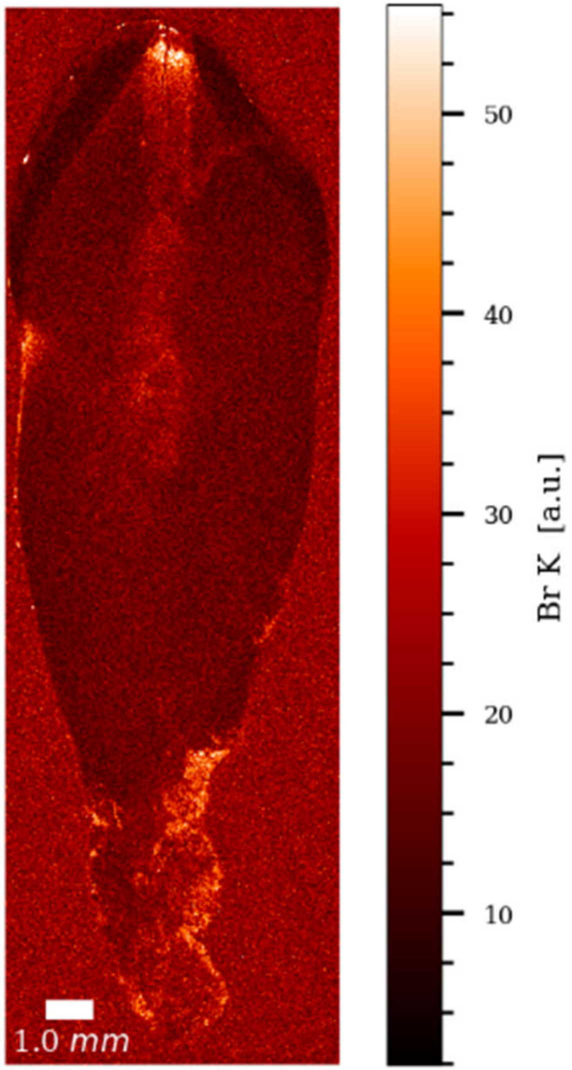

Pb L

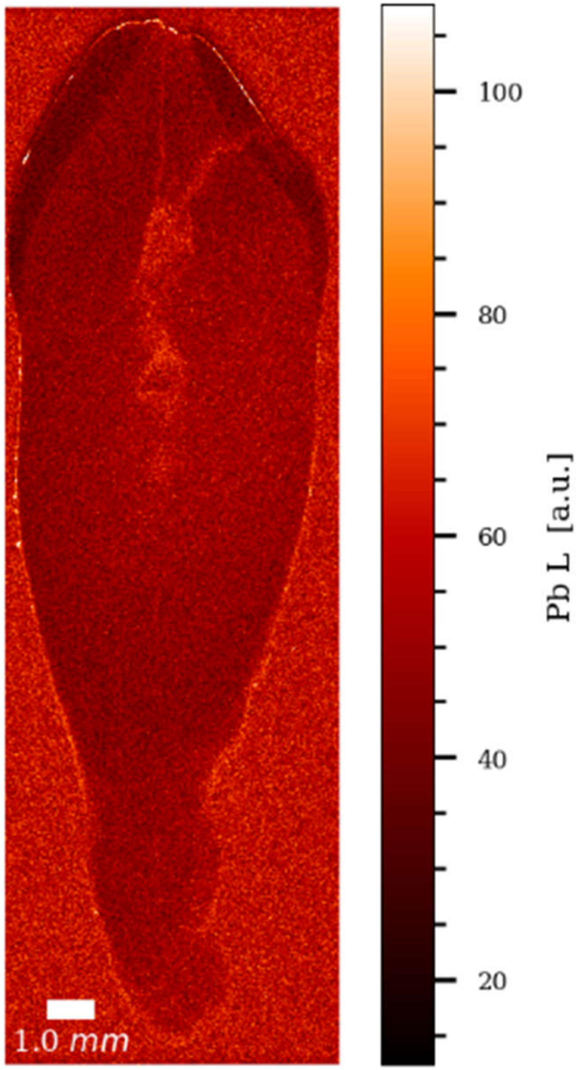

Odense 1149 URC

High resolution at 1 µm in acellular cementum

Gauss (1x1)

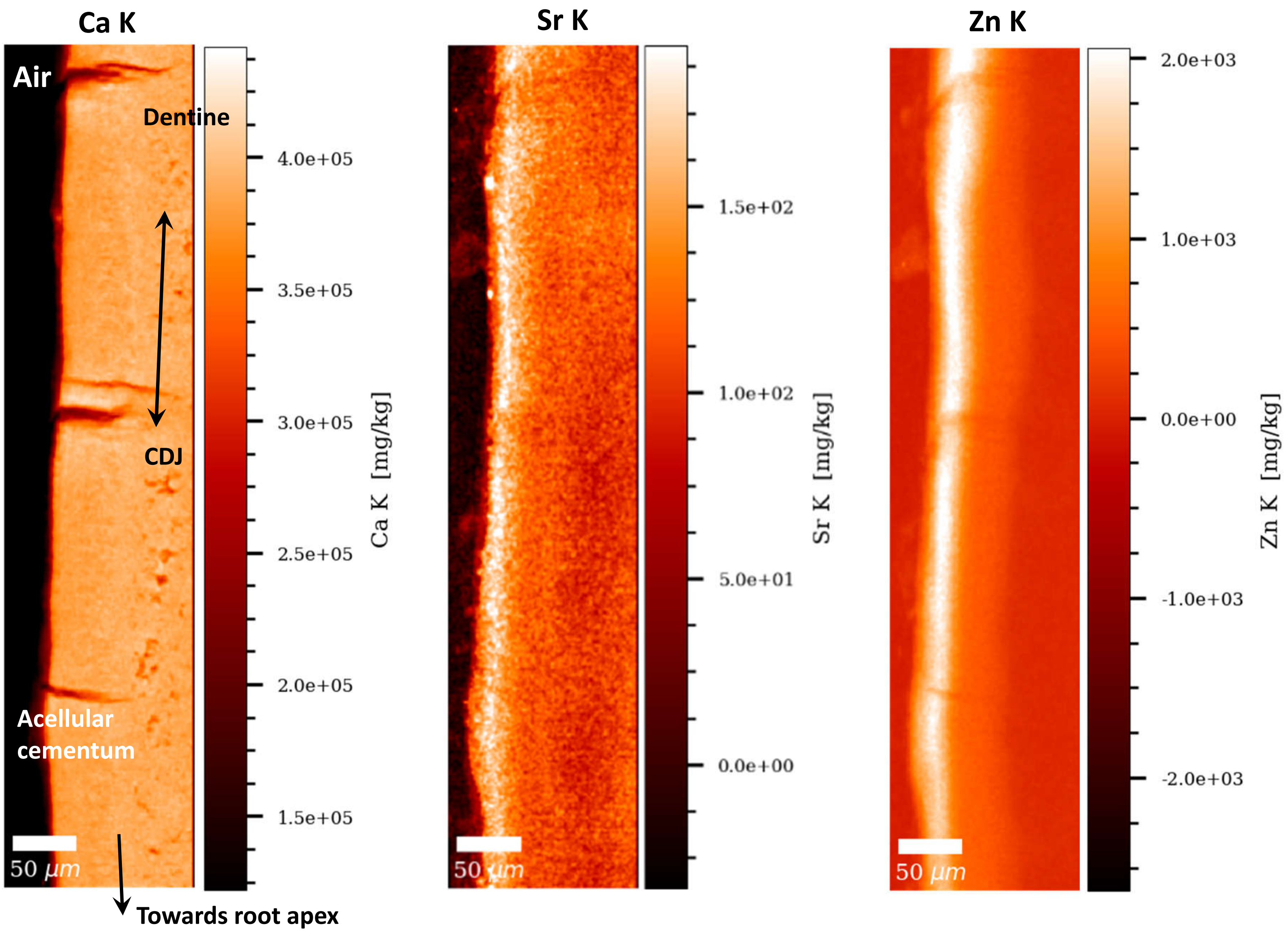

Odense 1149 URC

High resolution at 1 μm in acellular cementum

Gauss (1x1)

Cu K

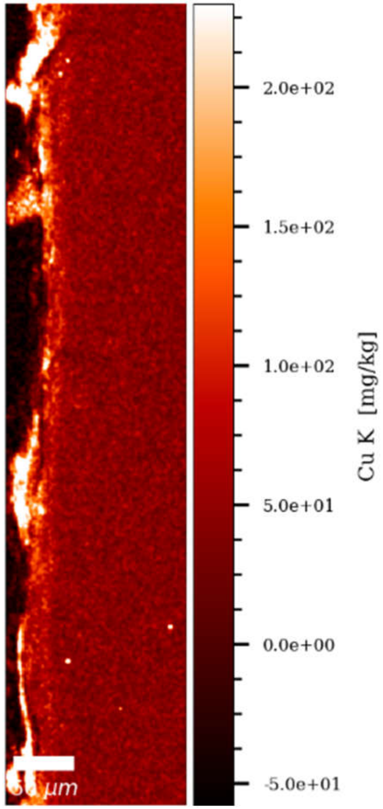

Fe K

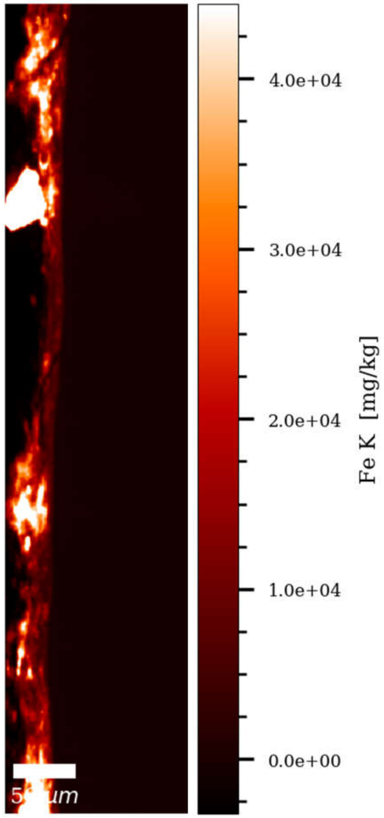

Mn K

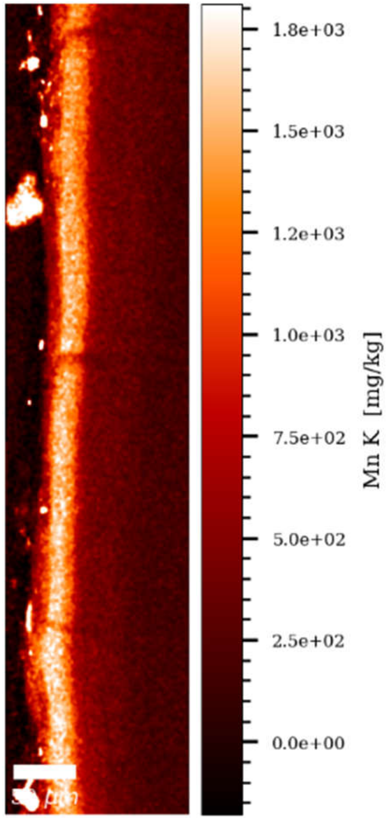

Odense – 1149 ULM1

♀ 20-30 yrs. 1301 – 1415 cal. CE

Average tooth section thickness (µm): 103.5

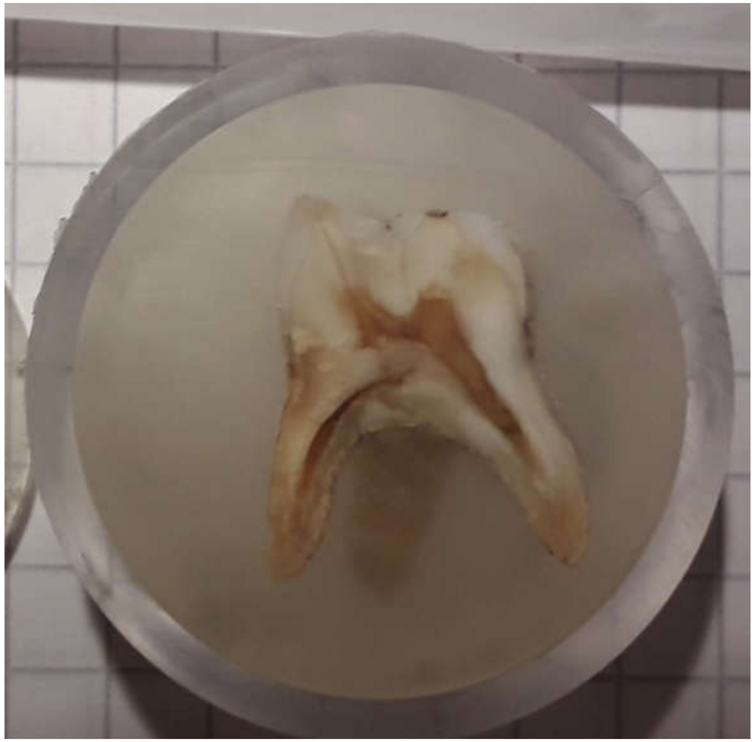

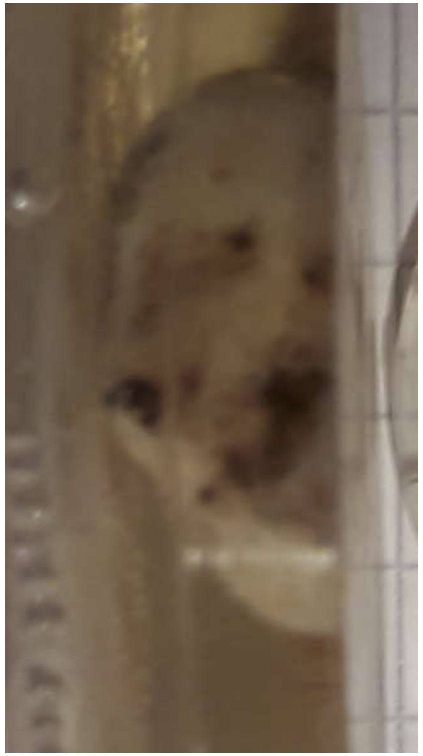

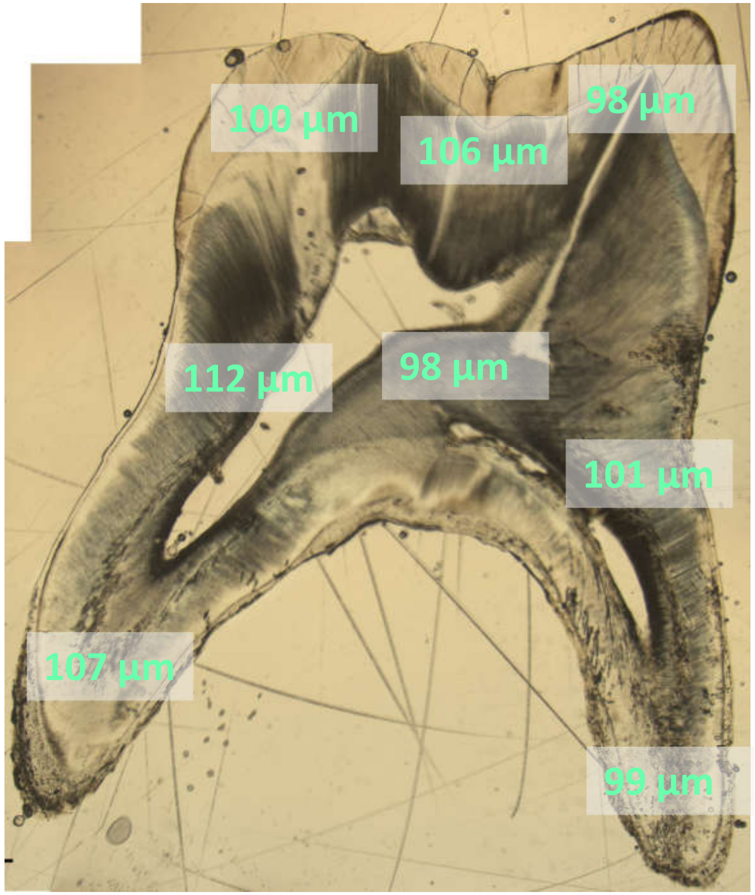

Odense 1149 ULM1

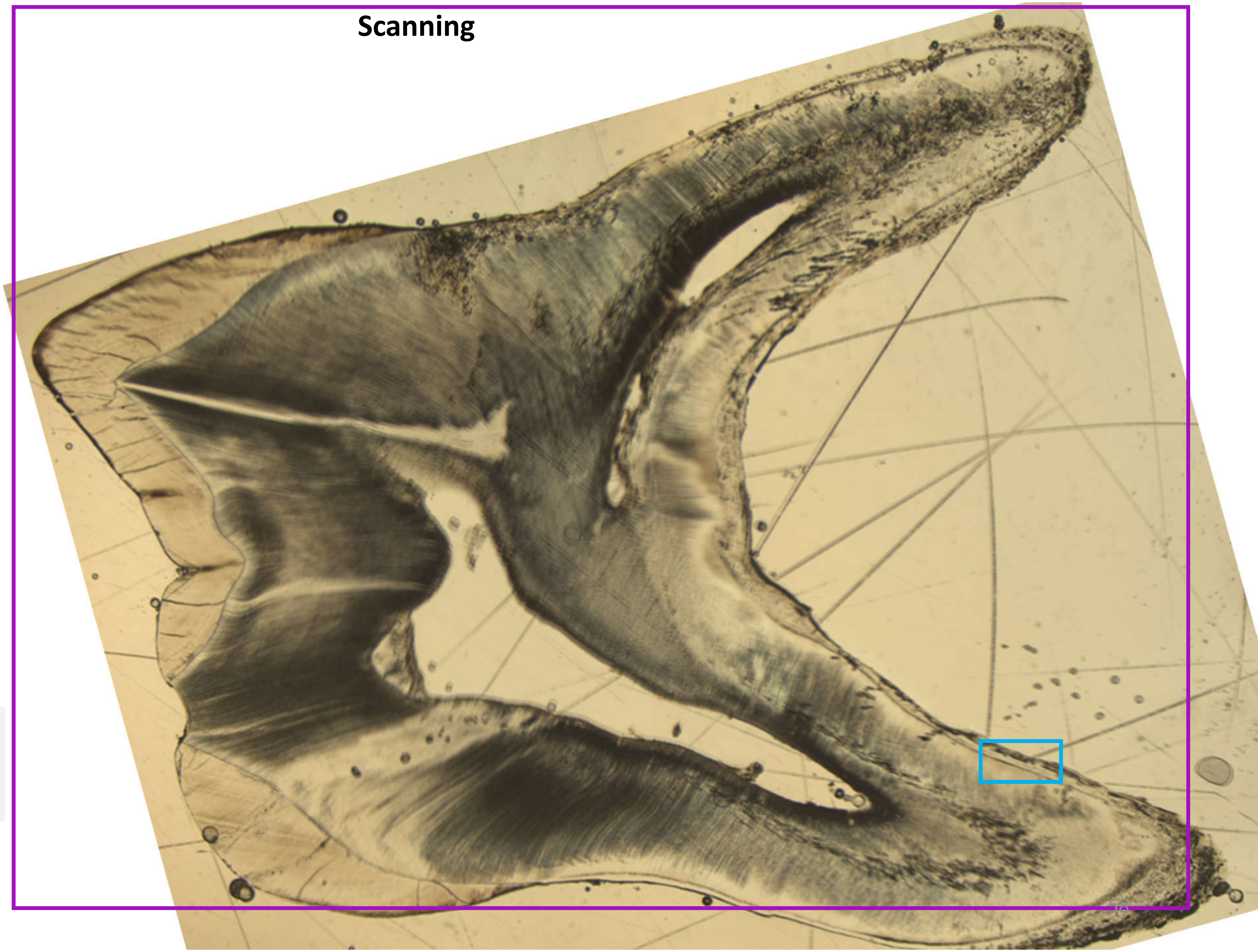

Overview at 10 µm
Problem of offset when centering sample

High resolution at 1 µm in mixed cellular and acellular cementum

Odense 1149 ULM1

Overview at 10 µm

Gauss (1x1)

Ca K

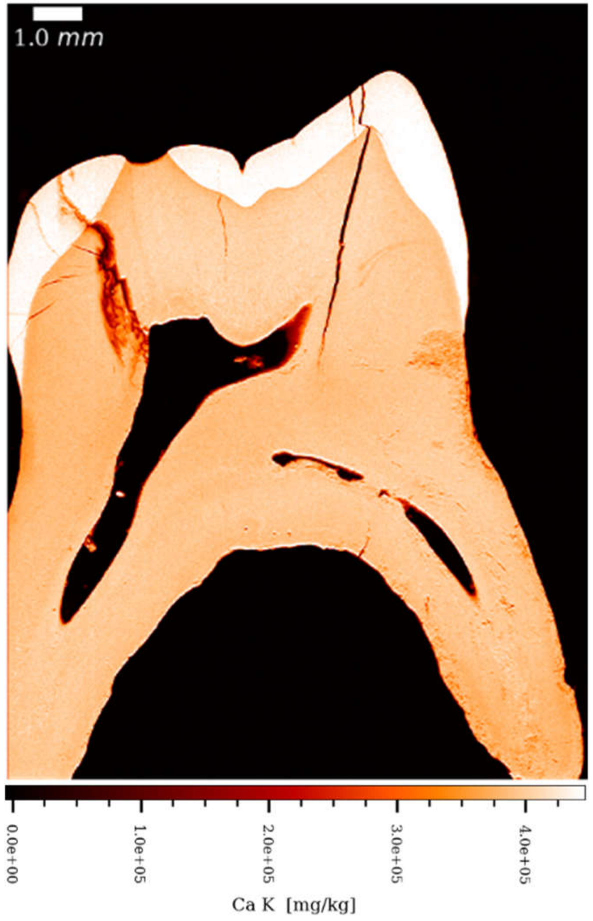

Sr K

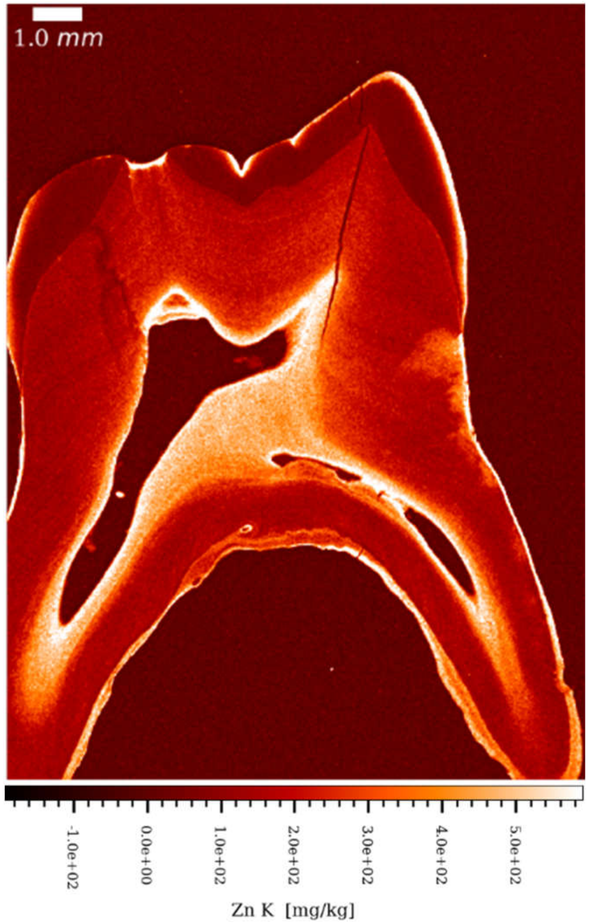

Zn K

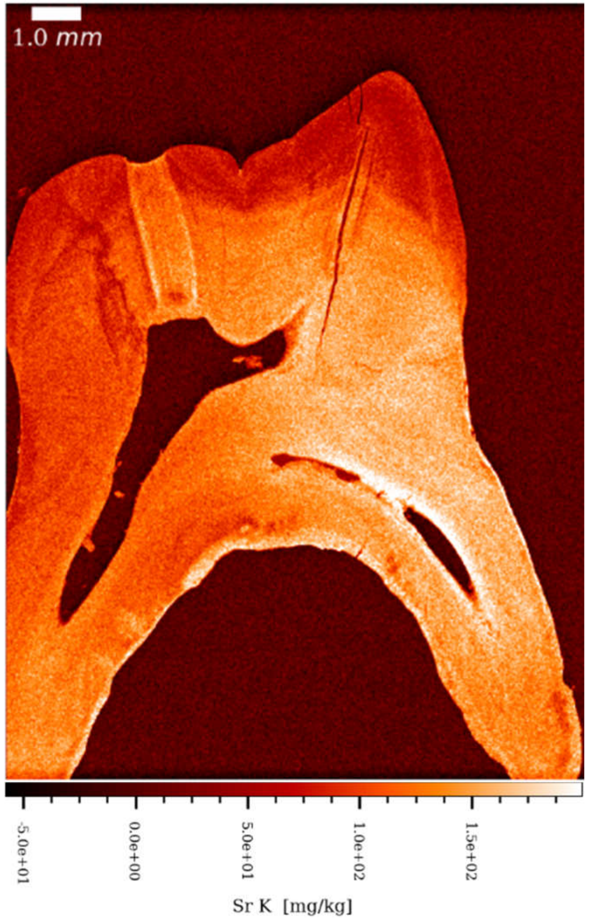

Odense 1149 ULM1

Overview at 10 µm

Gauss (1x1)

Cu K

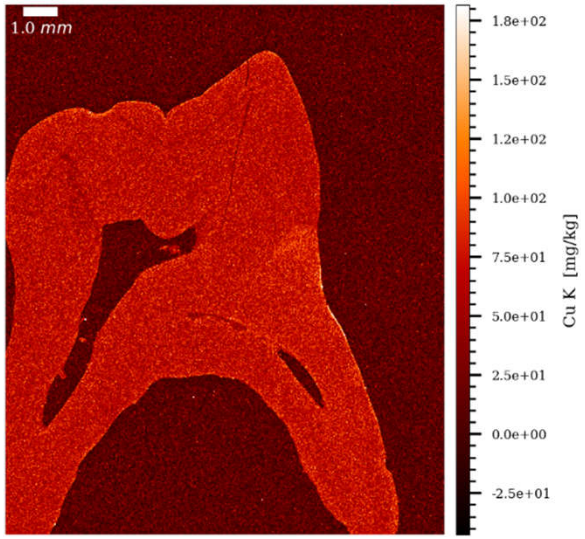

Fe K

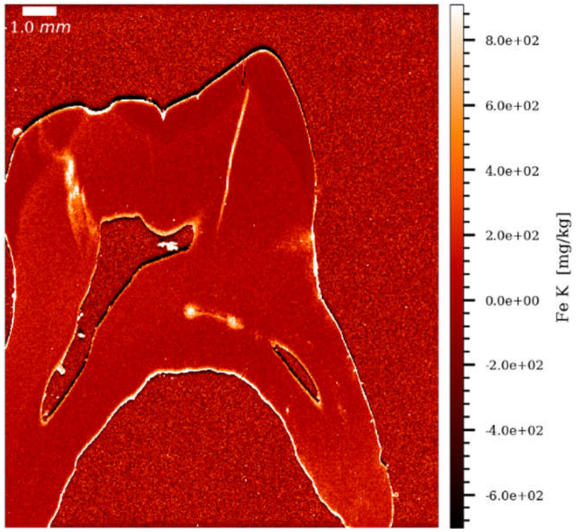

Mn K

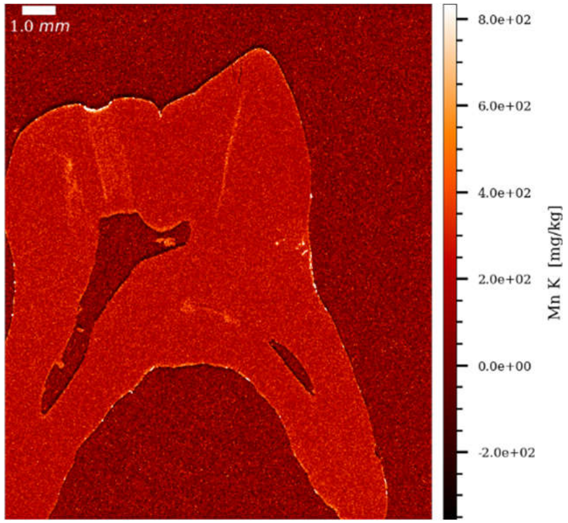

Odense 1149 ULM1

Overview at 10 µm

Uncalibrated data (arbitrary units)

Gauss (1x1)

Br K

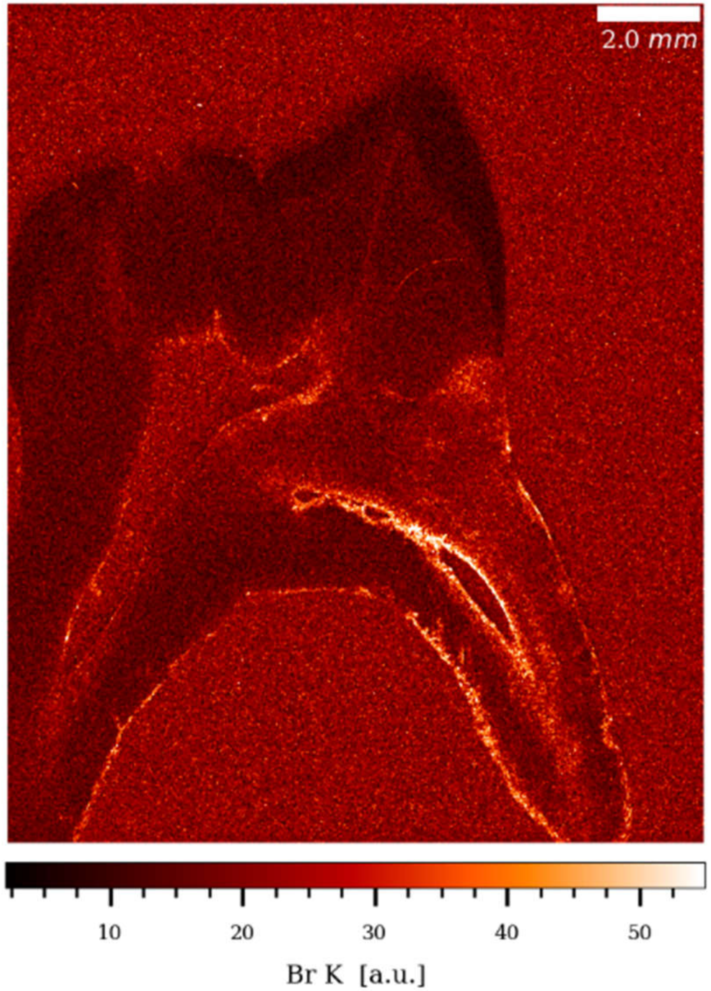

Pb L

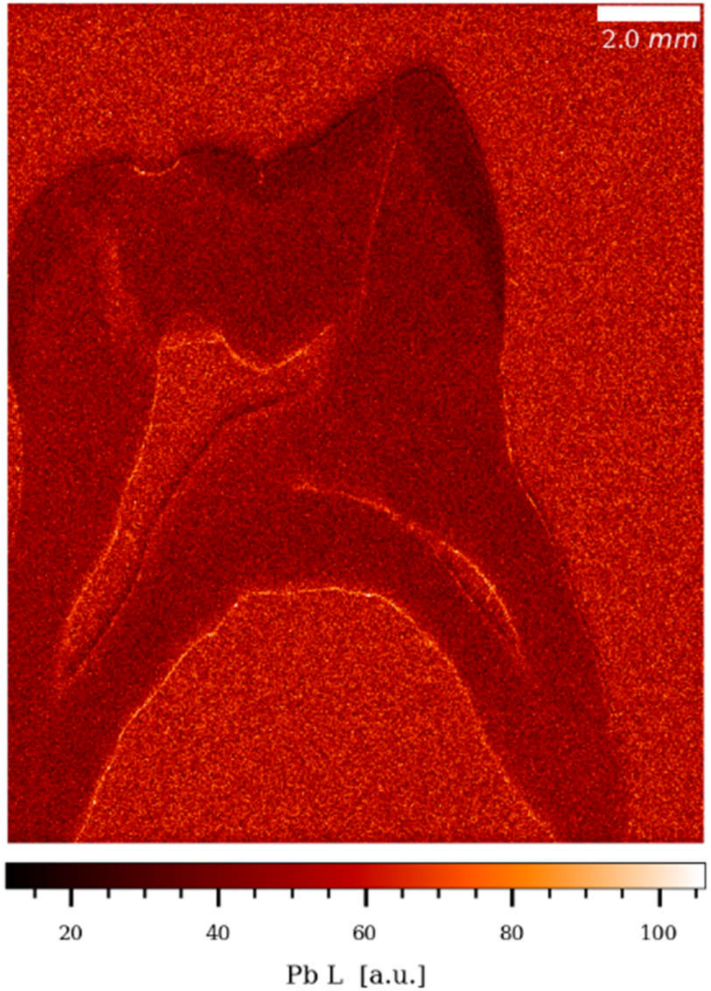

Odense 1149 ULM1

High resolution at 1 µm

Gauss (1.2x1.2)

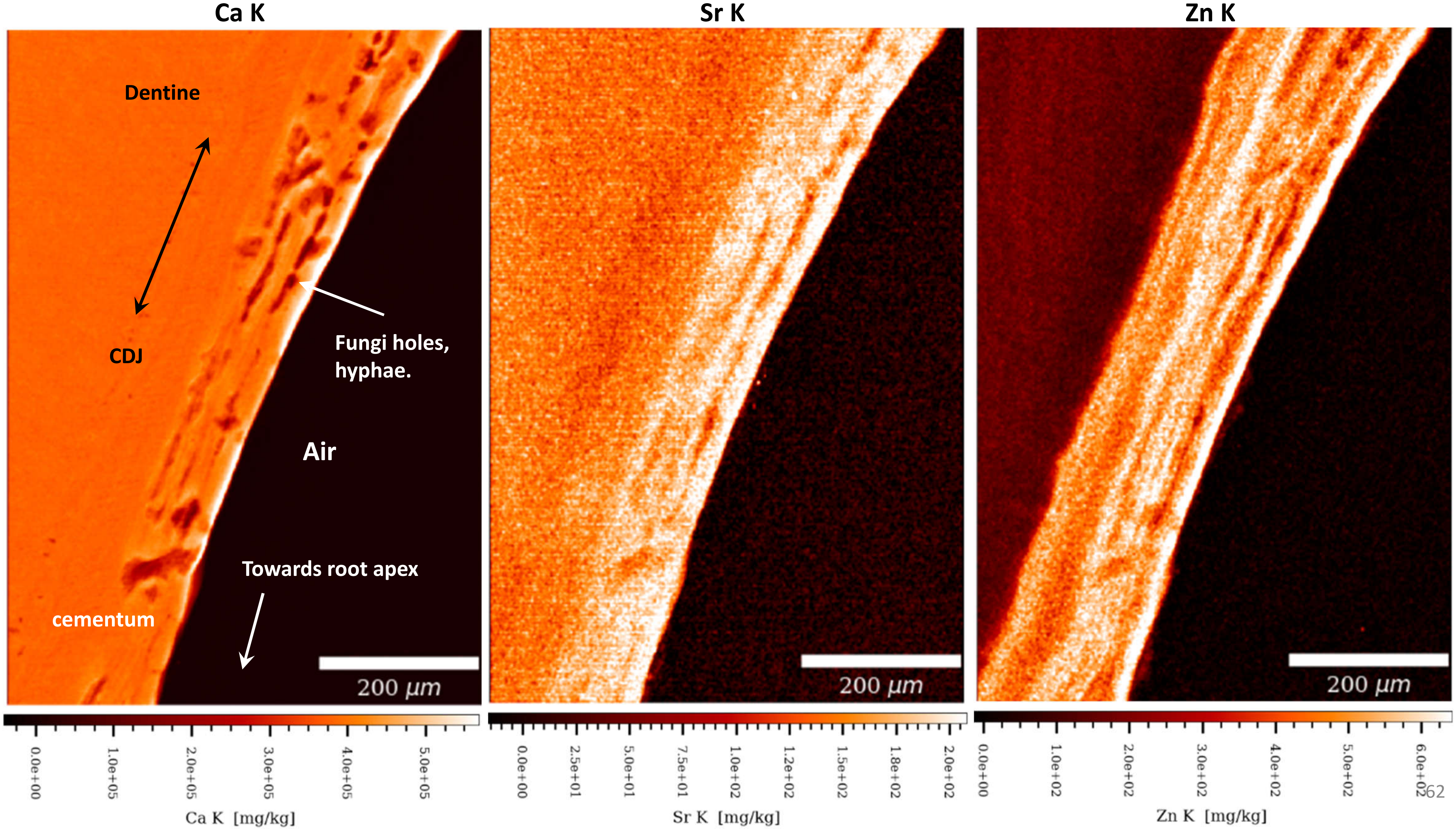

Odense 1149 ULM1

High resolution at 1 µm

Gauss (1.2x1.2)

Cu K

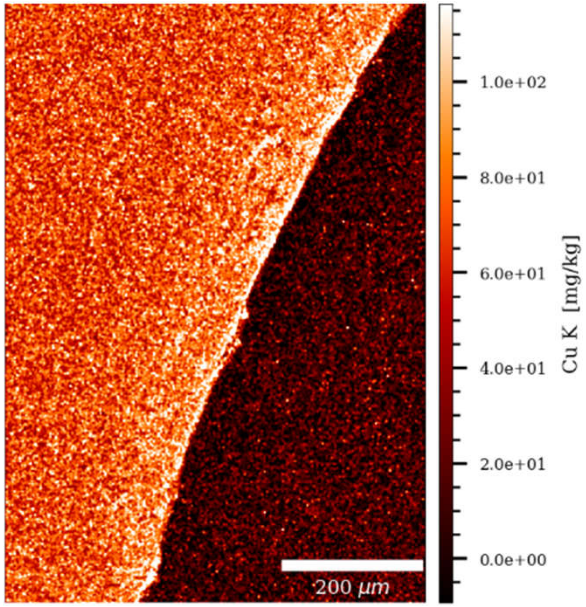

Fe K

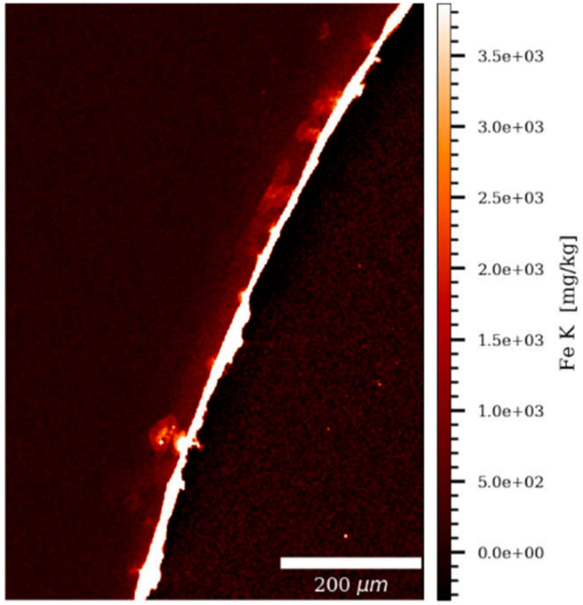

Mn K

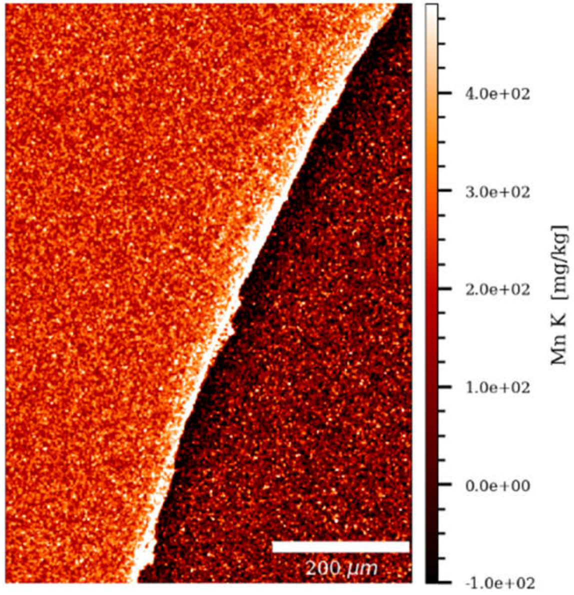

Næstved – 6 ULC

♀ ~20 yrs. mid-13th – mid-16th c. CE

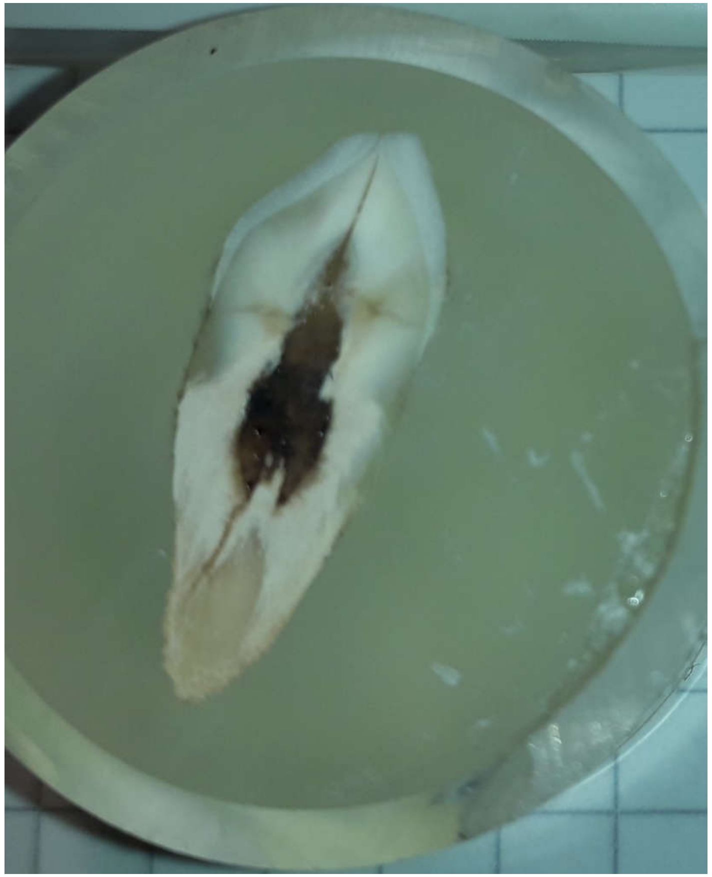

Average tooth section thickness (µm): 115.0

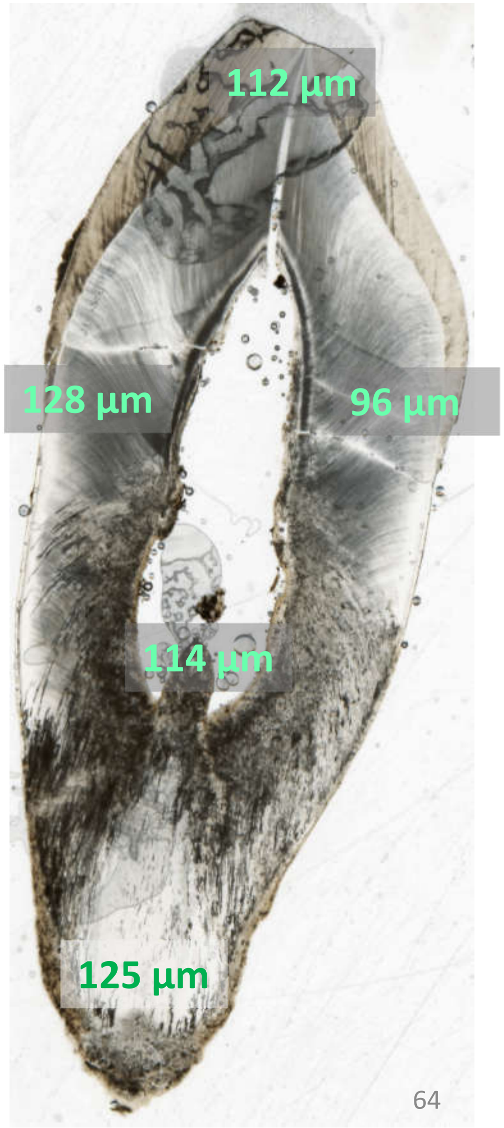

Næstved 6 ULC

Scanning

Overview at 10 µm

High resolution at 1 µm in acellular cementum

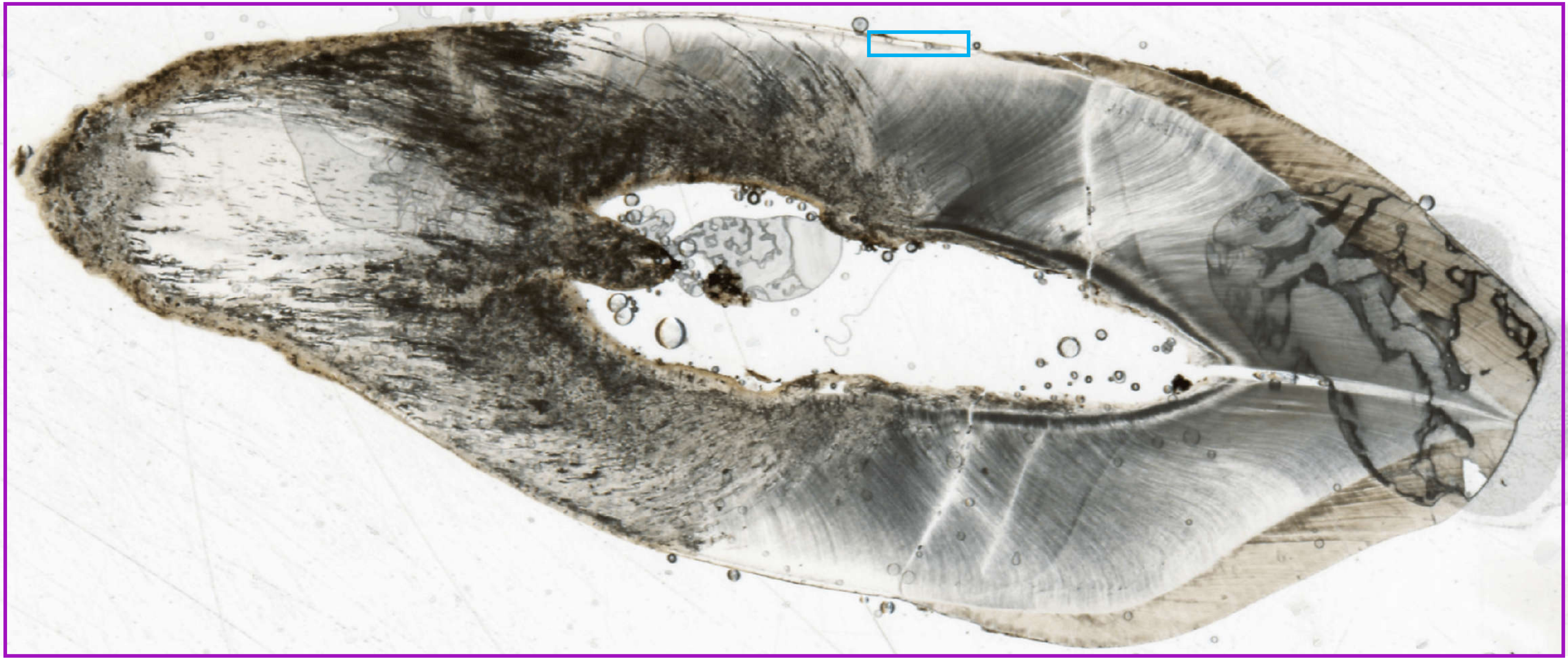

Næstved 6 ULC

Overview at 10 µm

Gauss (1x1)

Ca K

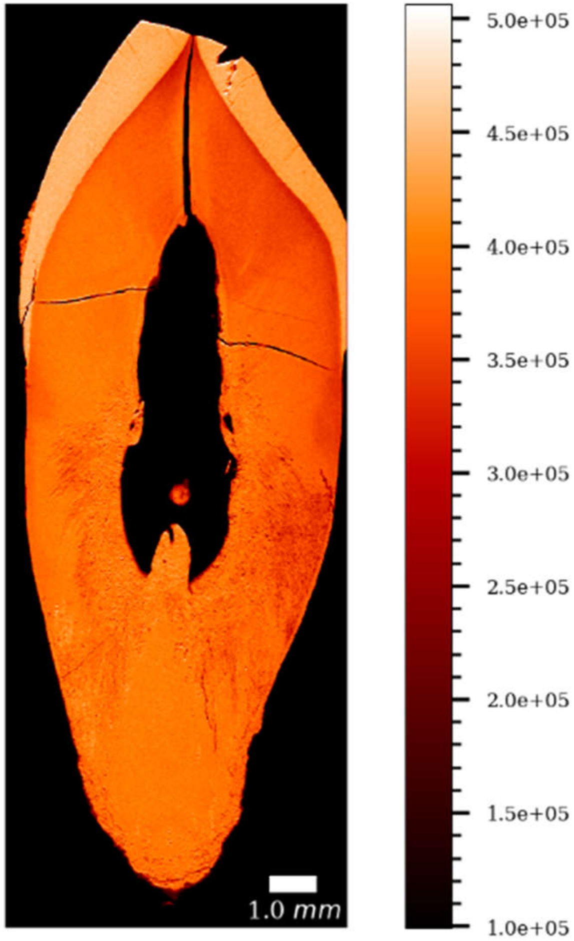

Sr K

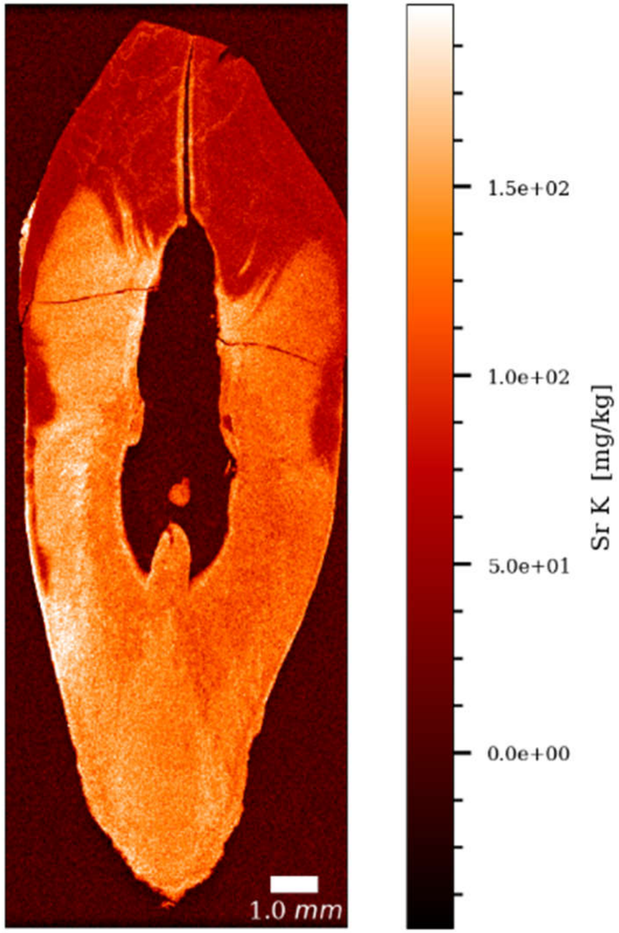

Zn K

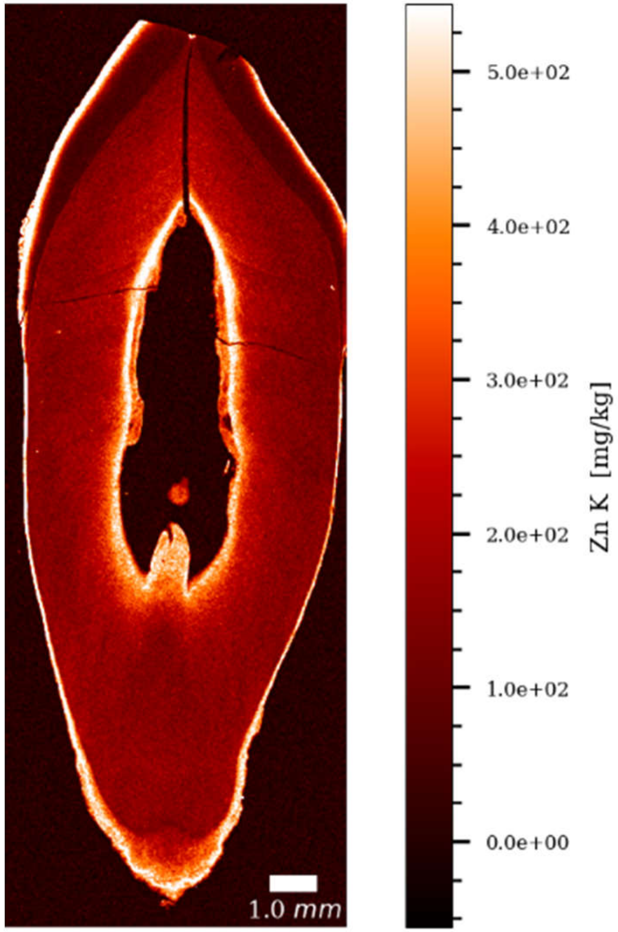

Næstved 6 ULC

Overview at 10 µm

Gauss (1x1)

Cu K

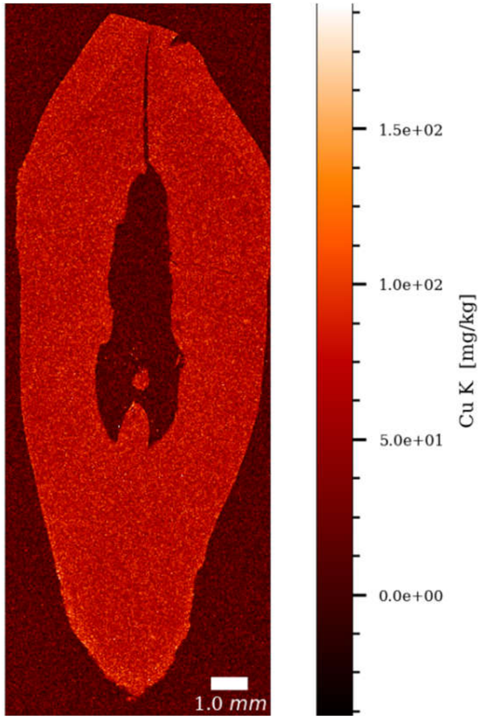

Fe K

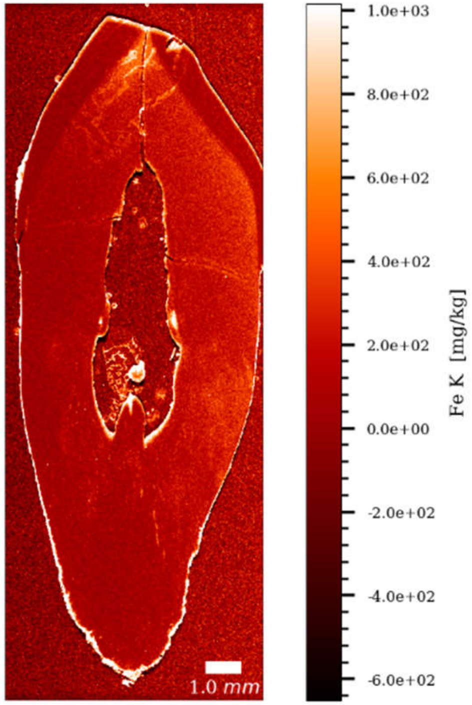

Mn K

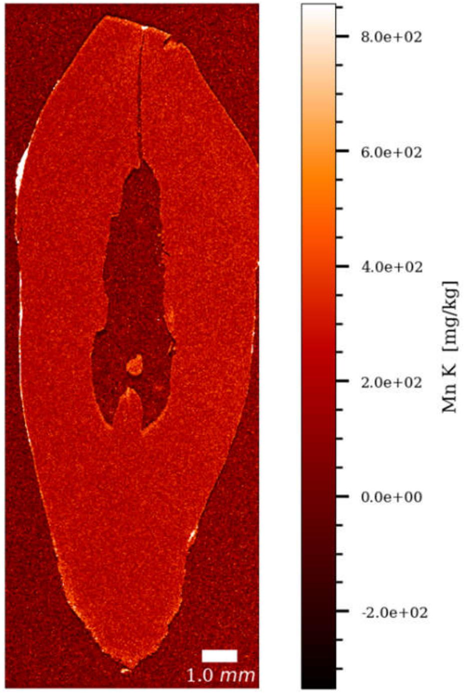

Næstved 6 ULC

Overview at 10 µm

Uncalibrated data
(arbitrary units)

Gauss (1x1)

Br K

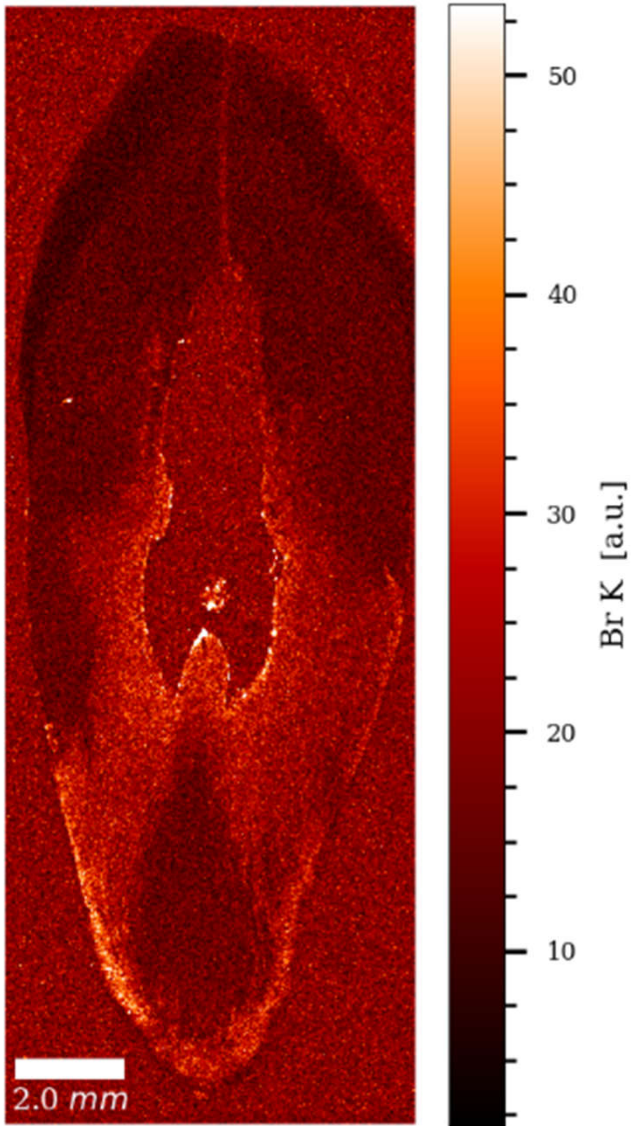

Pb L

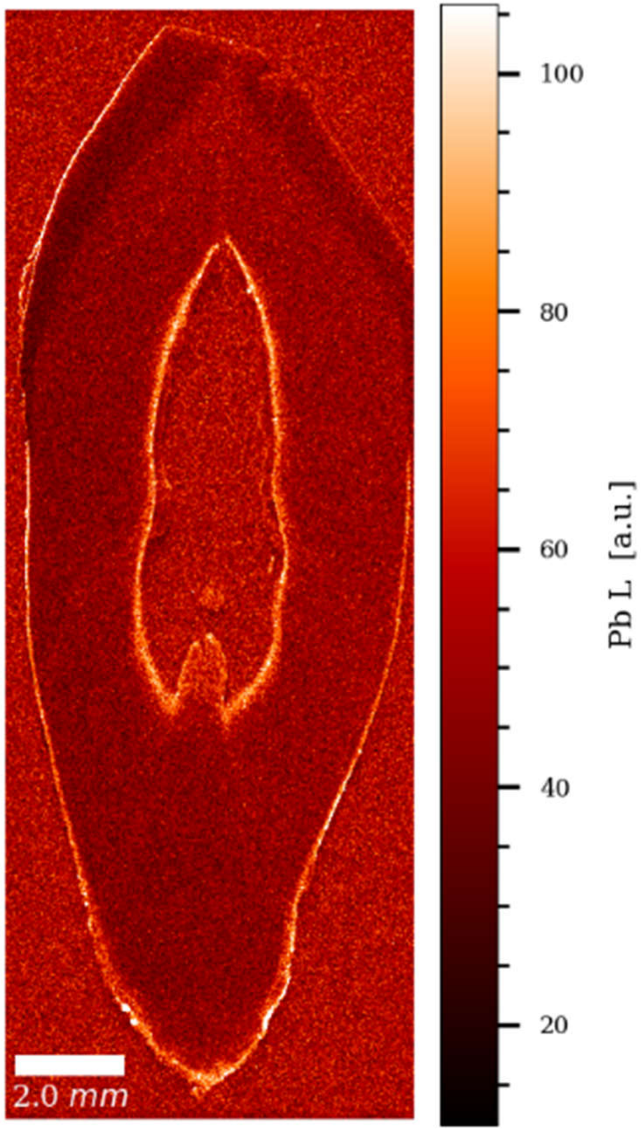

Næstved 6 ULC

High resolution at 1 µm

Gauss (1x1)

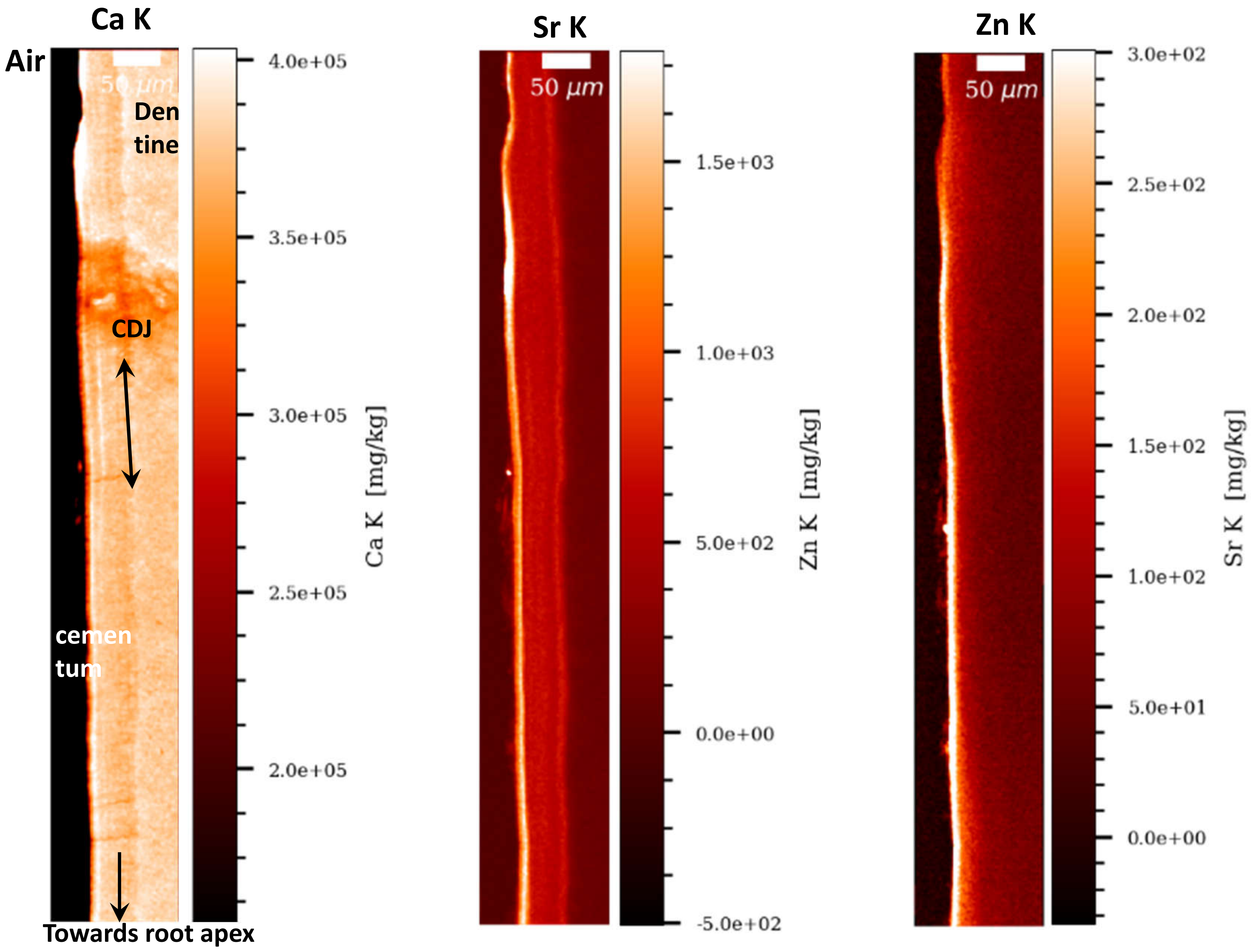

Næstved 6 ULC

High resolution at 1 µm

Gauss (1x1)

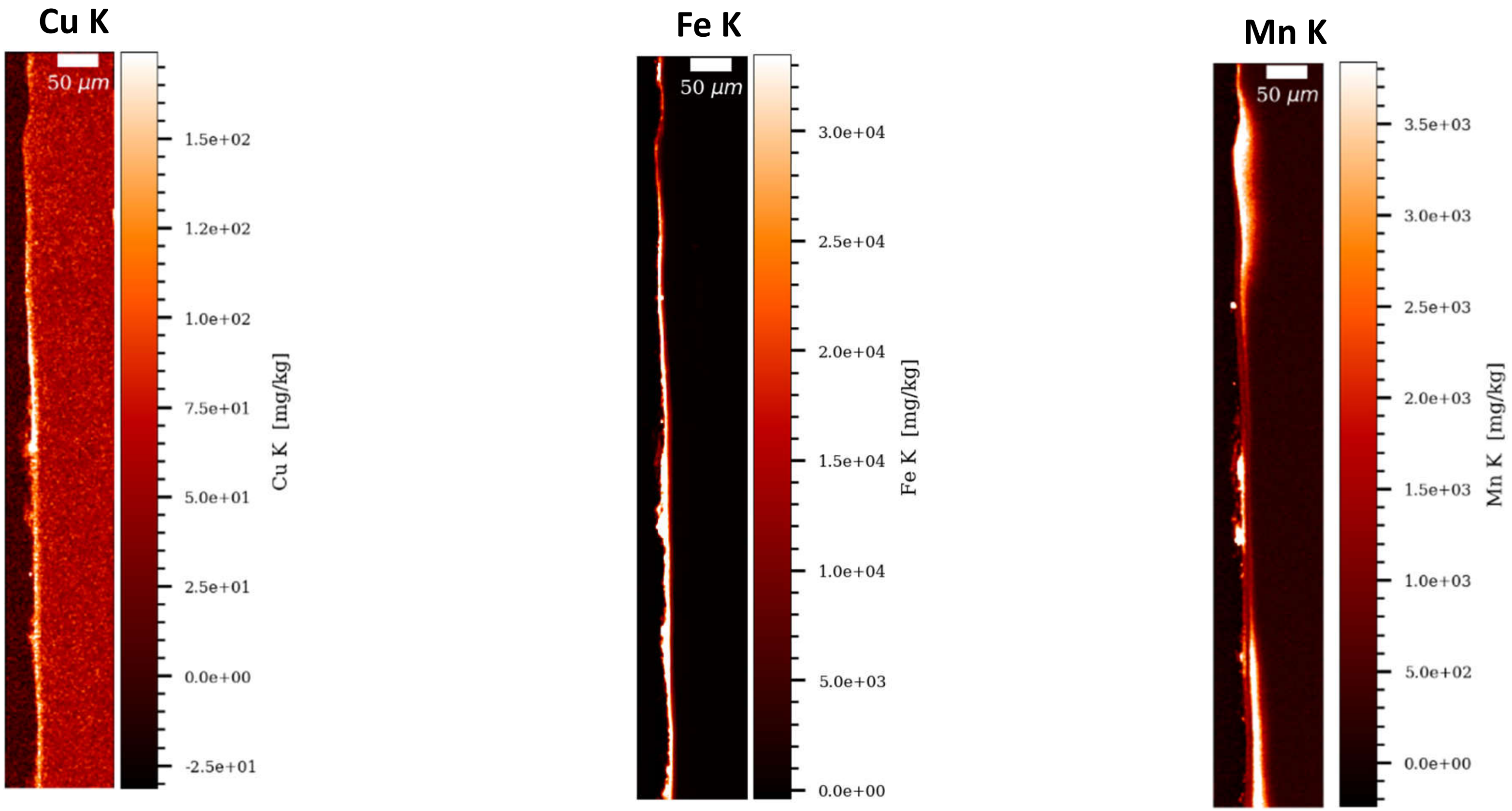

Næstved – 211 URC

♀ **40-45 yrs. 1184 – 1266 cal. CE**

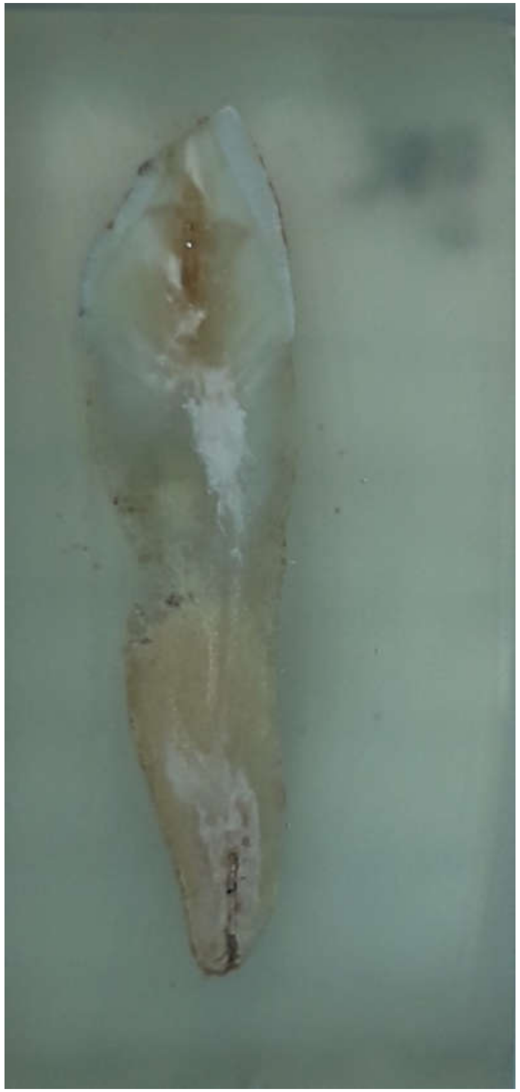

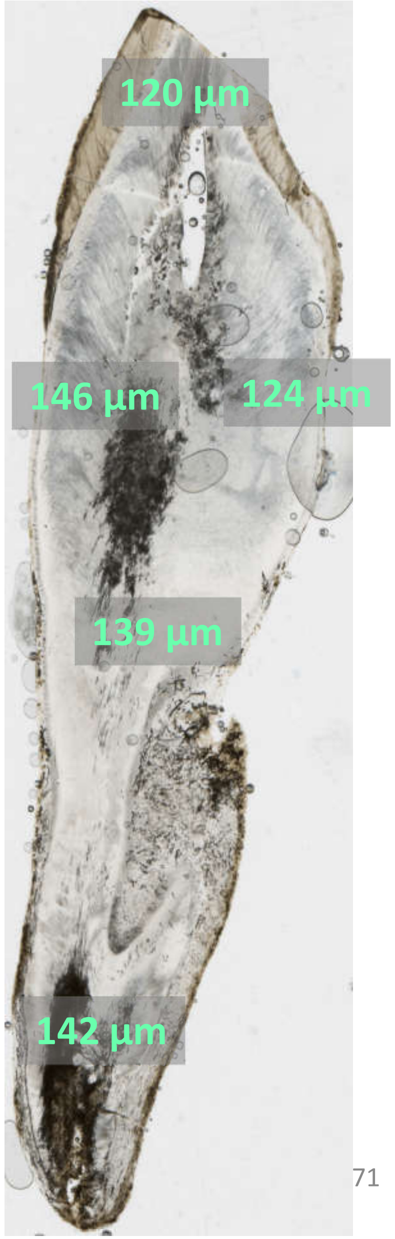

Average tooth section thickness (µm): 134.2

Næstved 211 URC

Scanning

Overview at 10 µm

High resolution at 1 µm

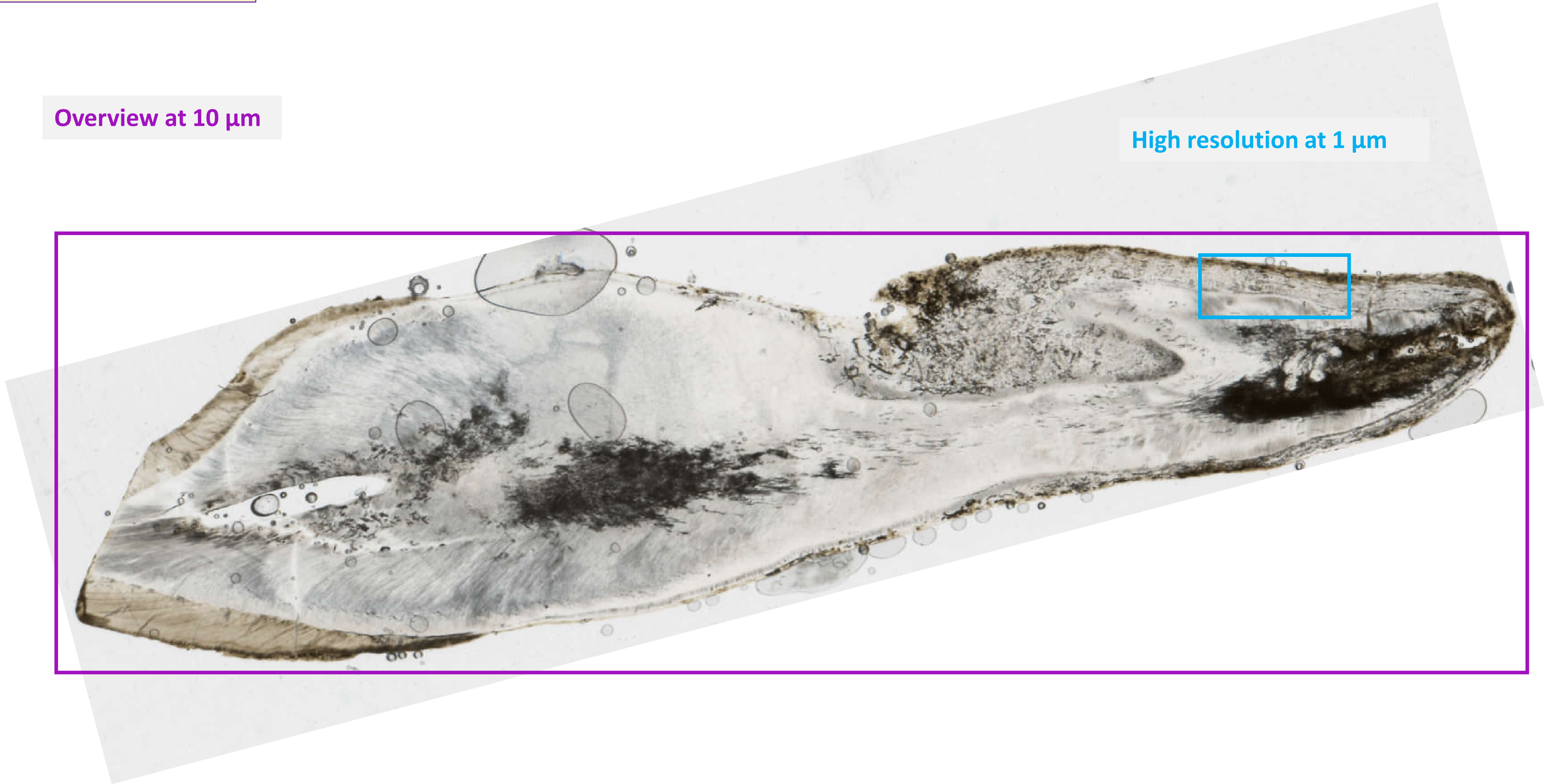

Næstved 211 URC

Overview at 10 µm

Gauss (1.2x1.2)

Ca K

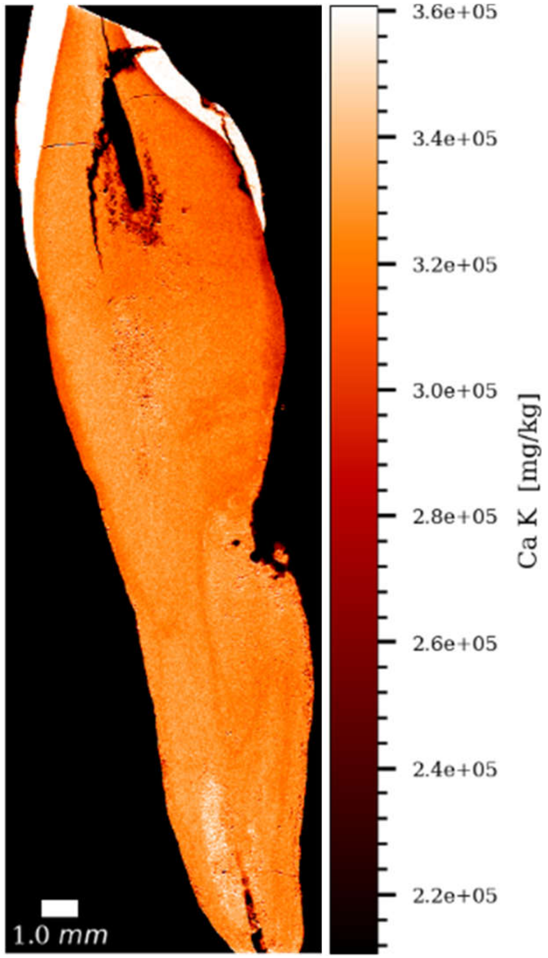

Sr K

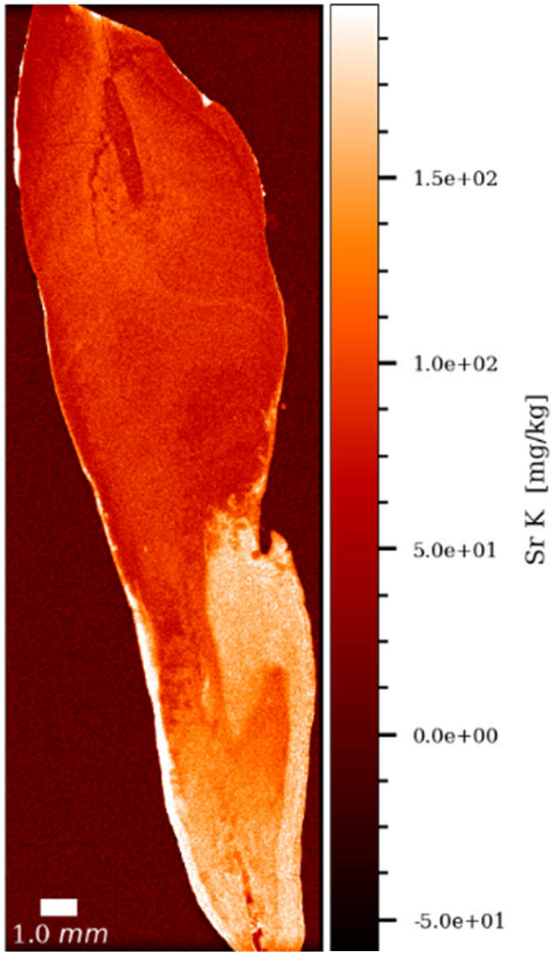

Zn K

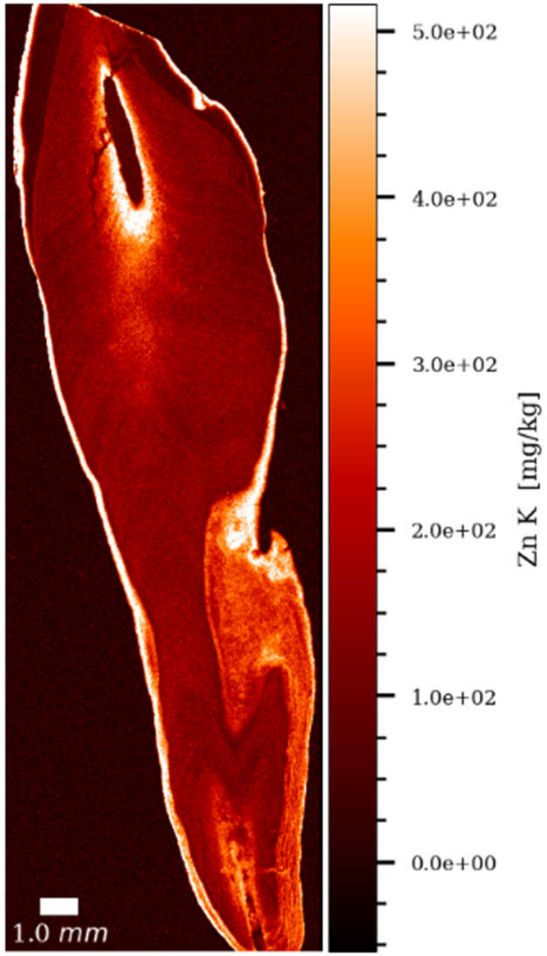

Næstved 211 URC

Overview at 10 µm

Gauss (1.2x1.2)

Cu K

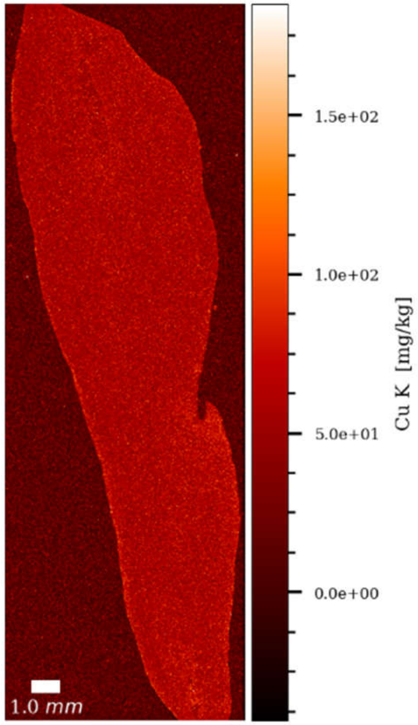

Fe K

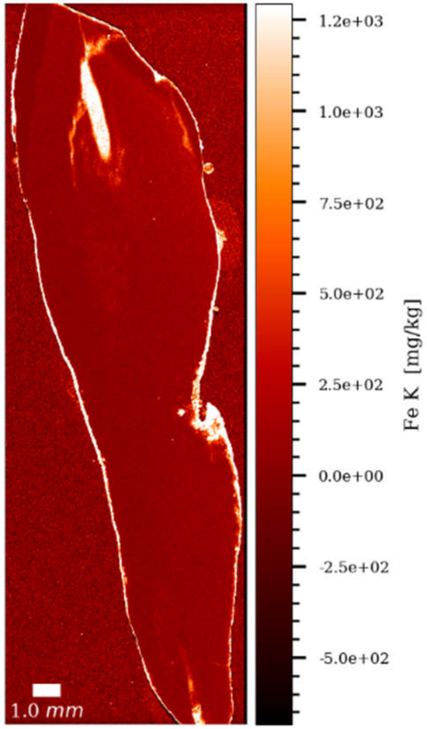

Mn K

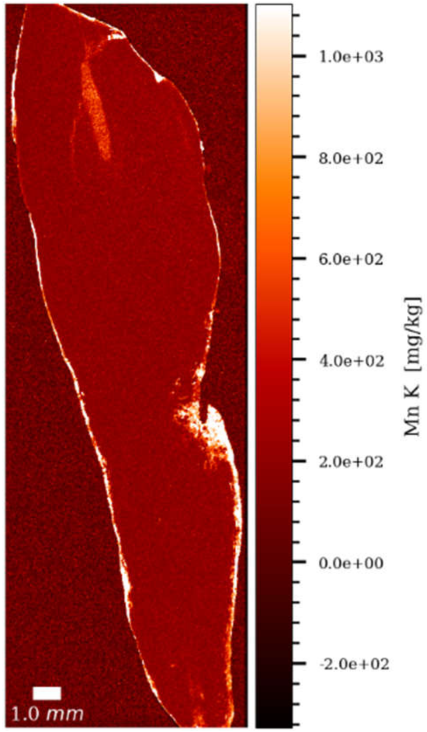

Næstved 211 URC

Overview at 10 µm

Uncalibrated data (arbitrary units)

Gauss (1.2x1.2)

Br K

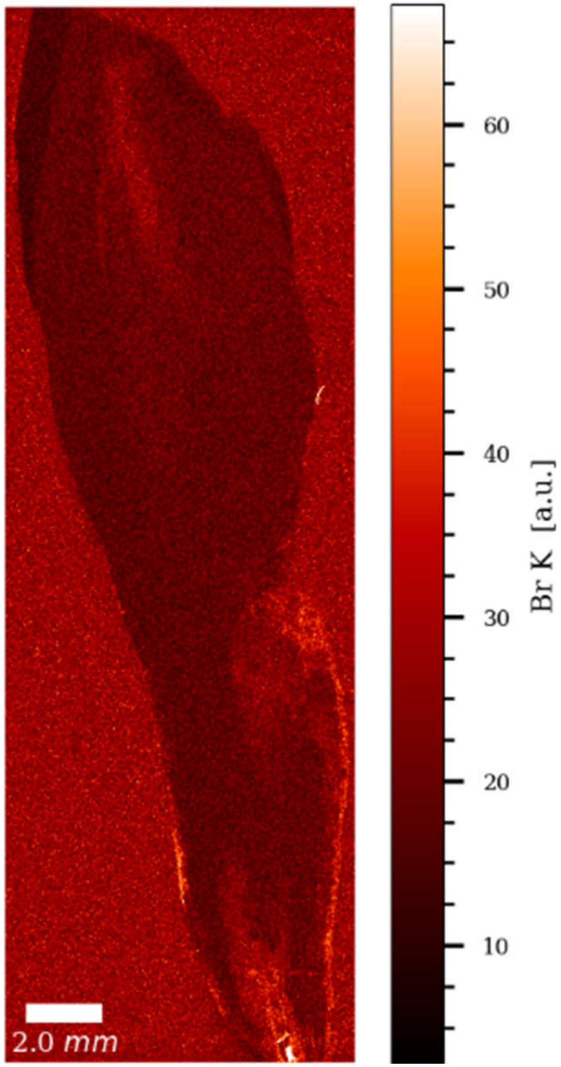

Pb L

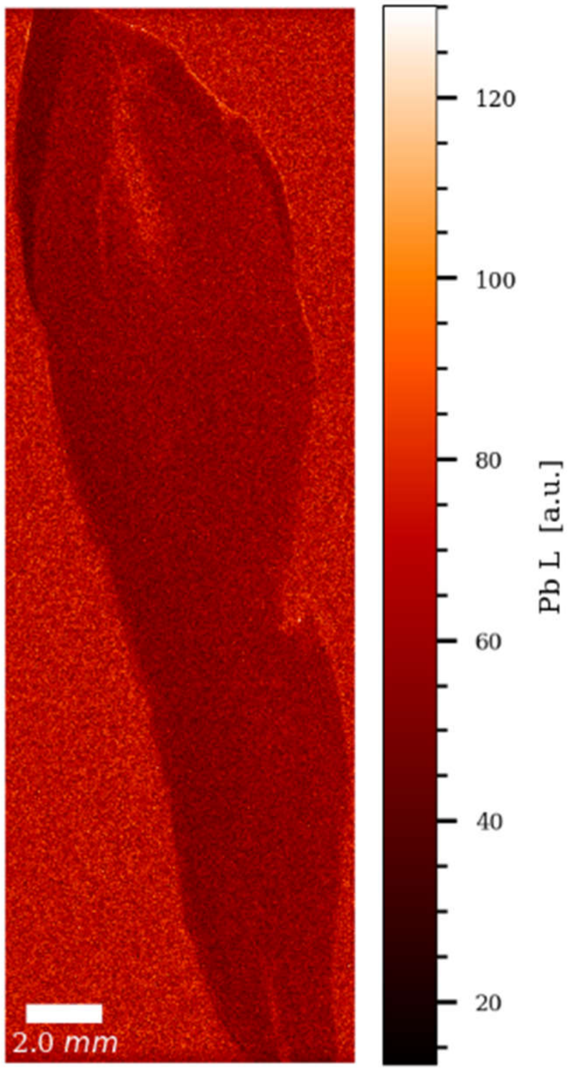

Næstved 211 URC

High resolution at 1 µm

Uncalibrated data (arbitrary units)

Gauss (1x1)

Ca K

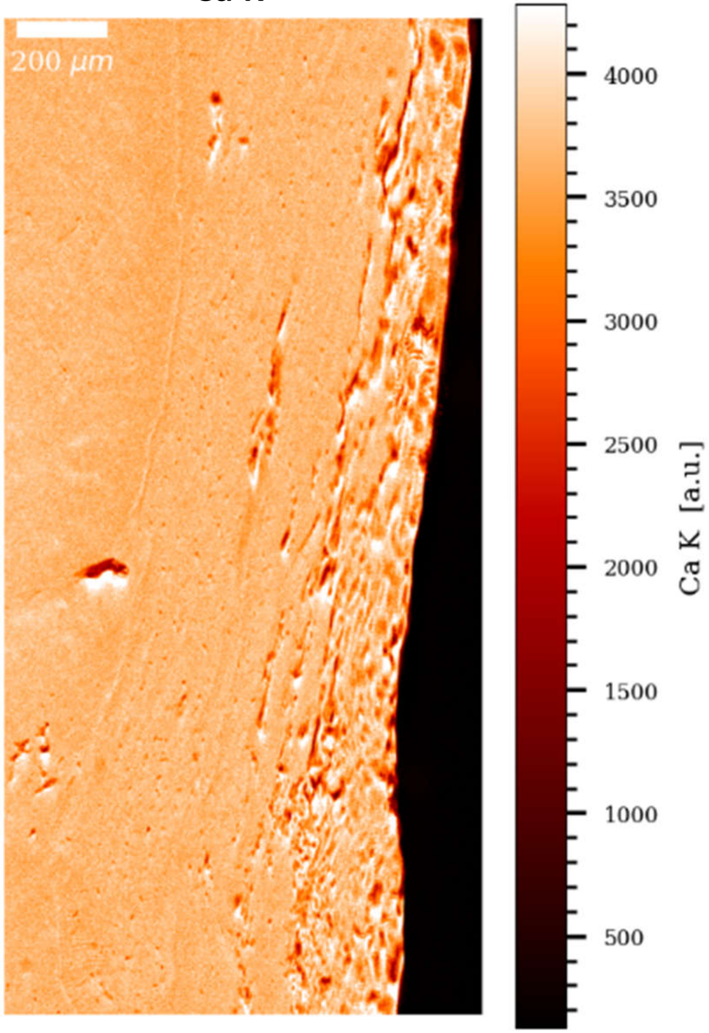

Sr K

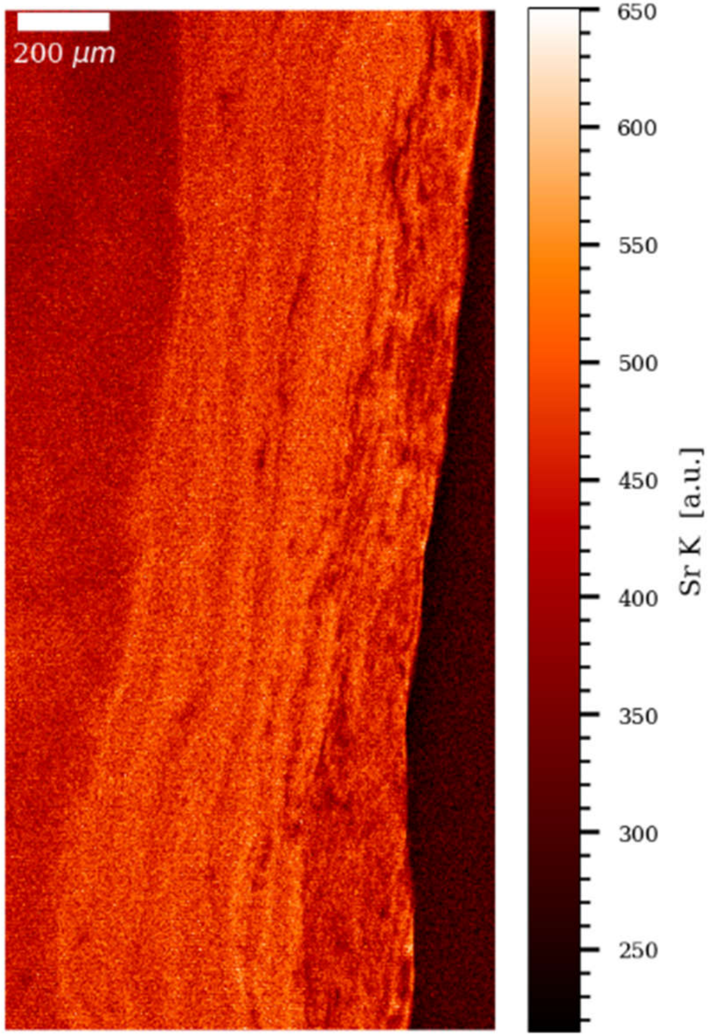

Zn K

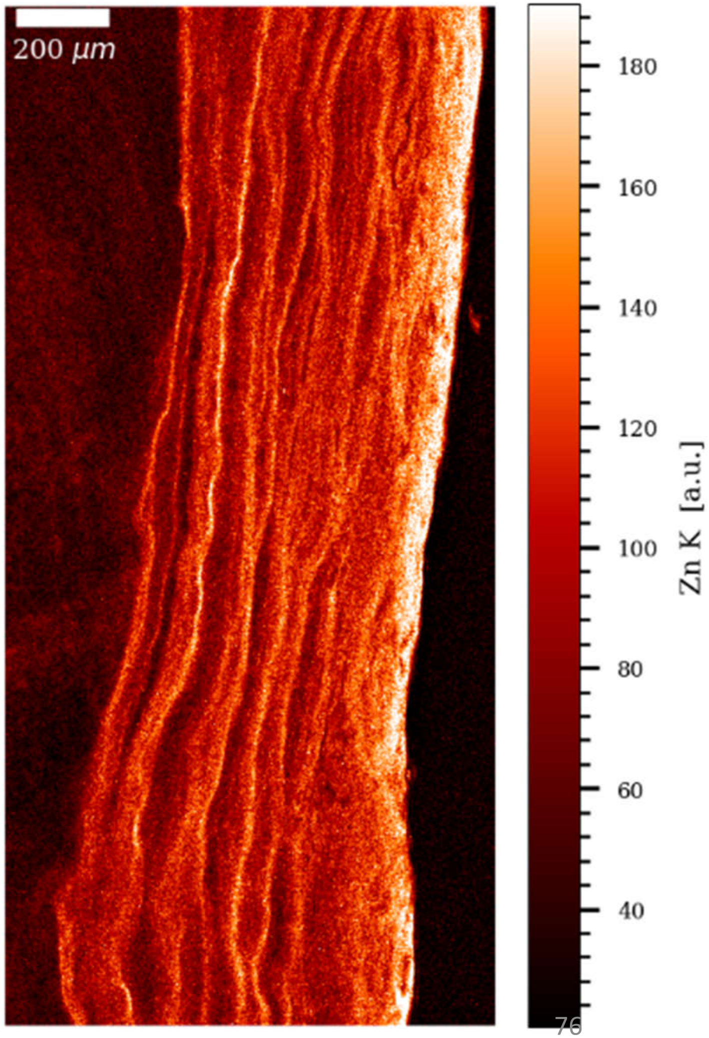

Næstved 211 URC

High resolution at 1 μm

Uncalibrated data (arbitrary units)

Gauss (1x1)

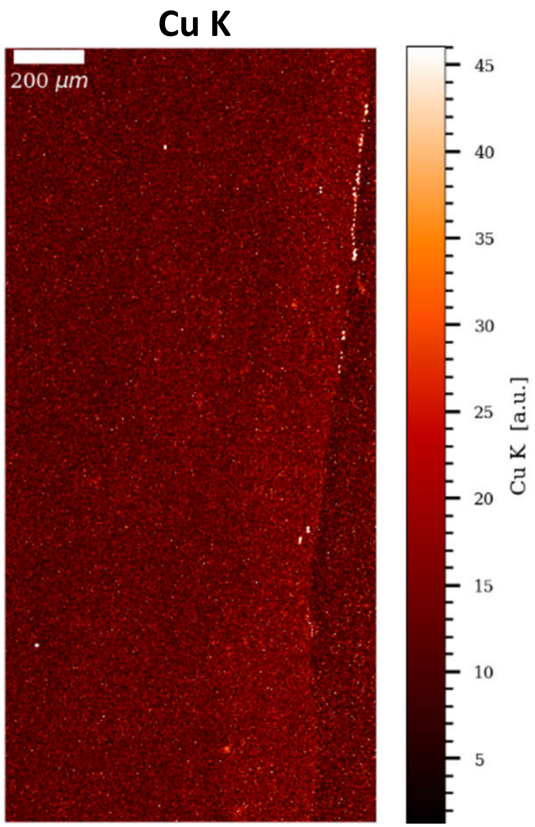

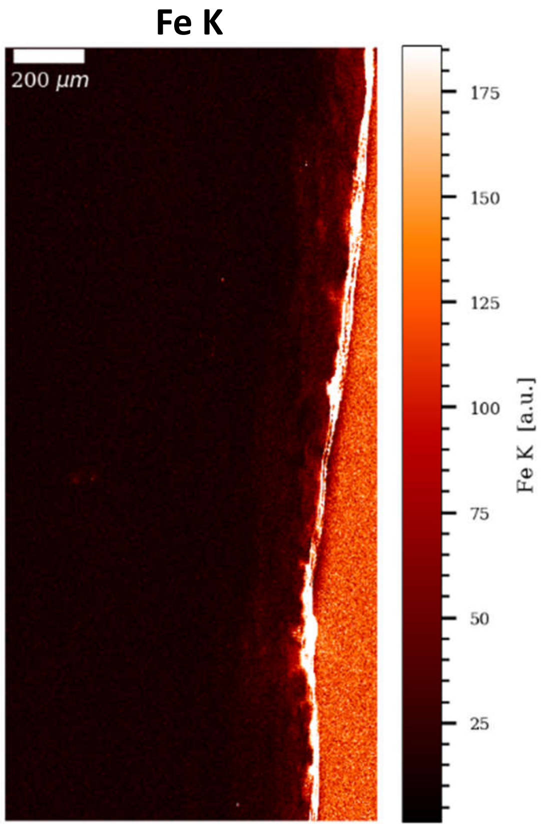

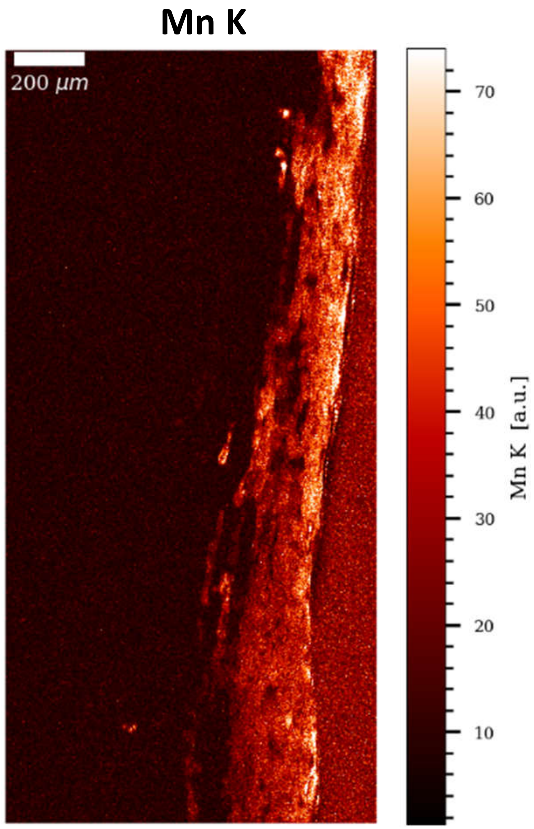

Næstved 211 URC

High resolution at 1 µm

Uncalibrated data
(arbitrary units)

Gauss (1x1)

Br K

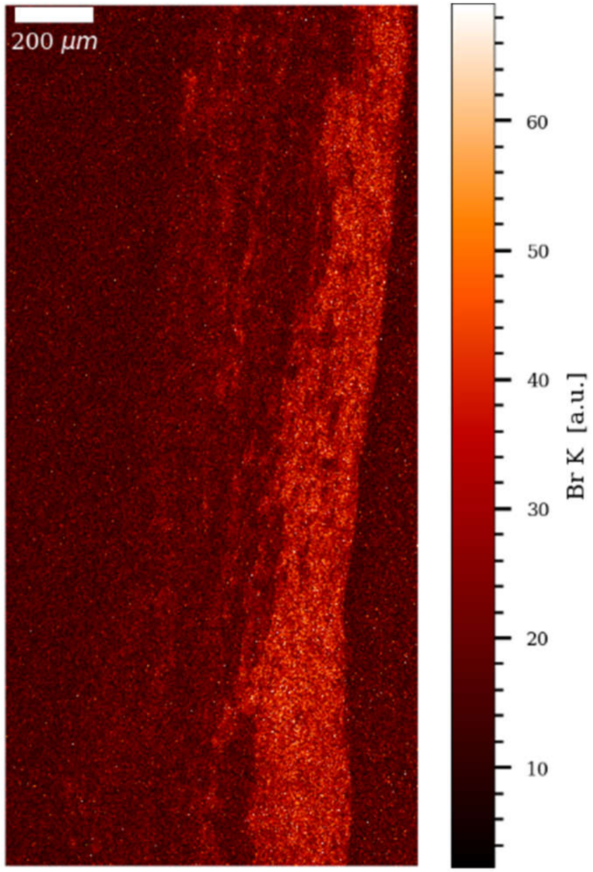

Pb L

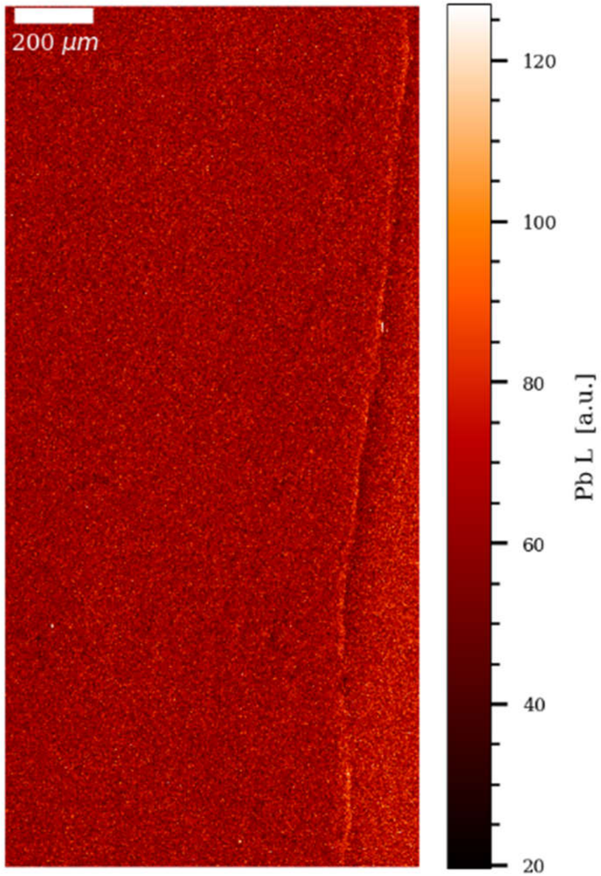

Næstved – 211 LRM1

♀ 40-45 yrs. 1184 – 1266 cal. CE

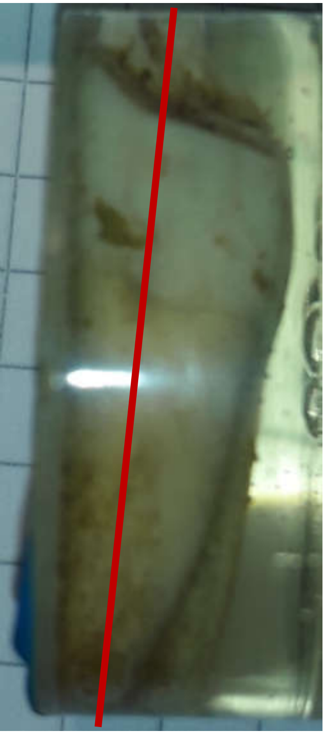

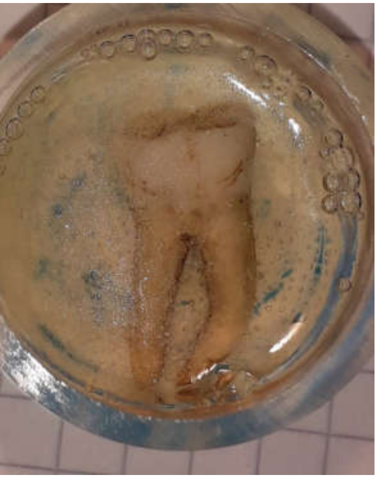

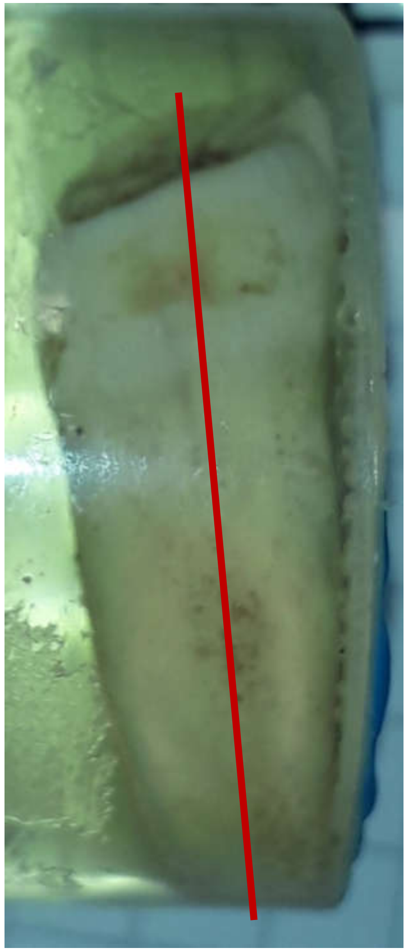

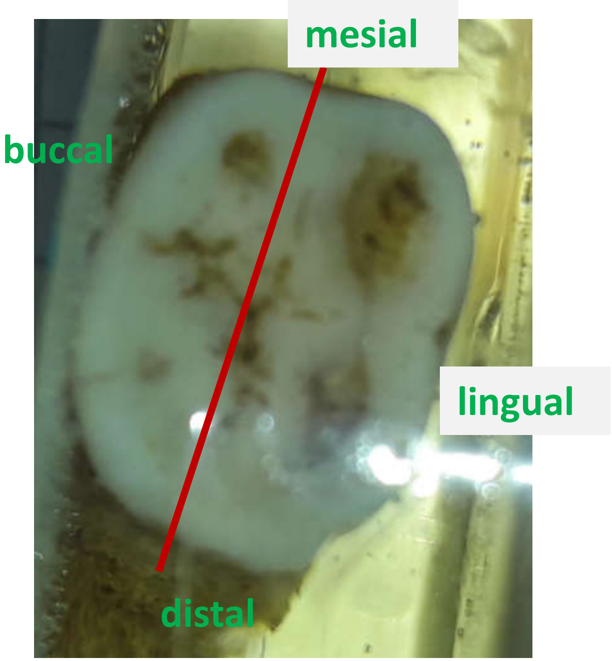

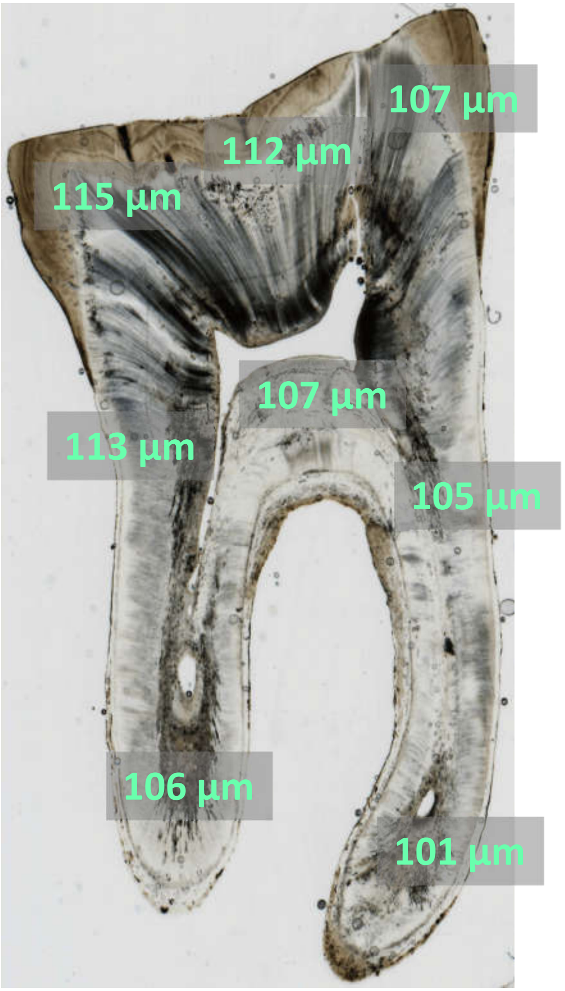

Average tooth section thickness (µm): 108.3

Næstved 211 LRM1

Scanning

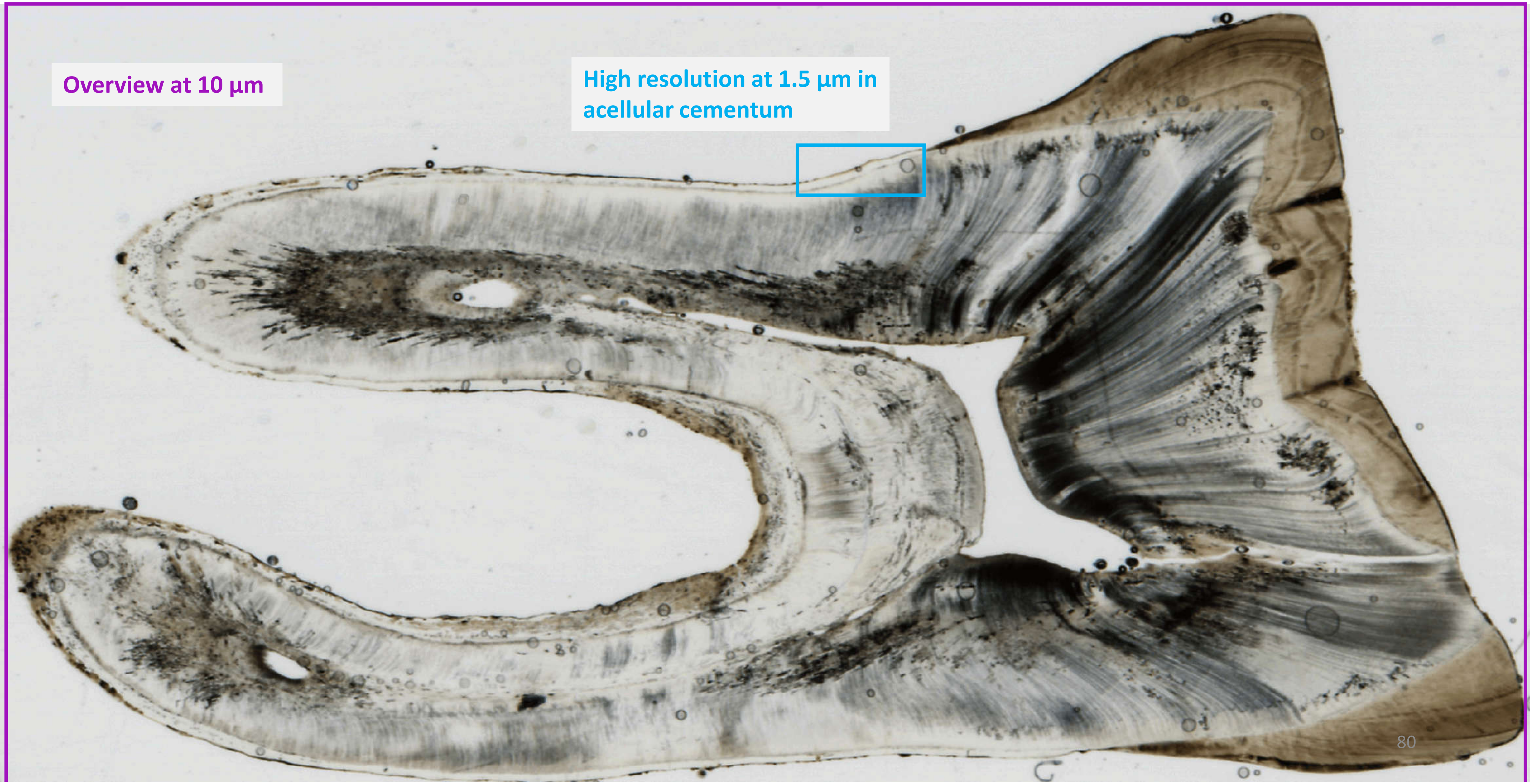

Næstved 211 LRM1

Overview at 10 µm

Gauss (1x1)

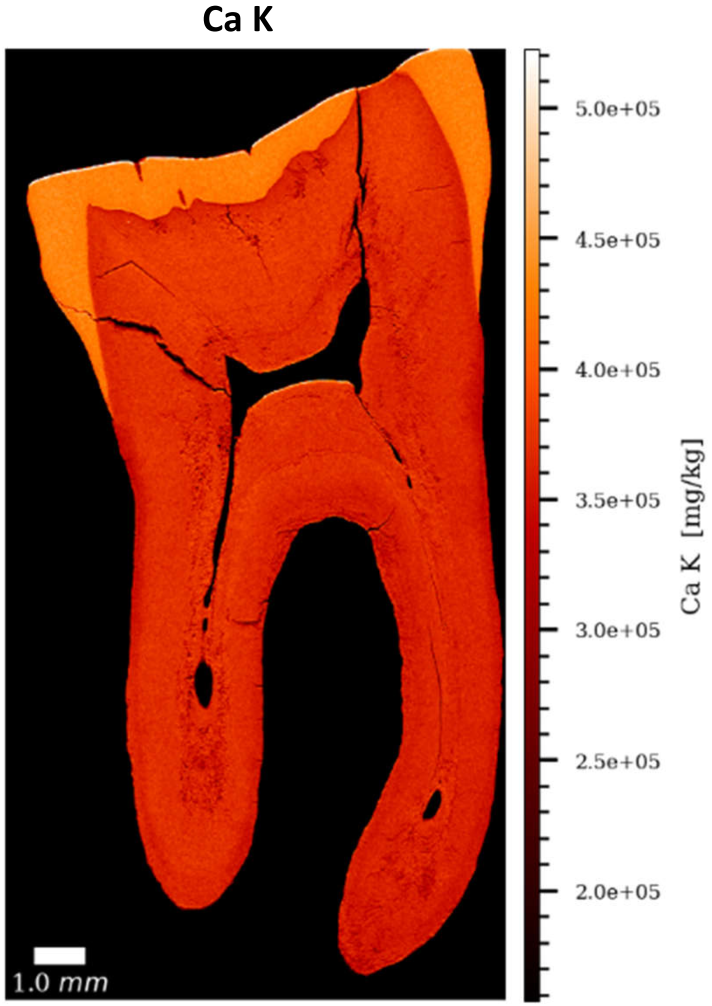

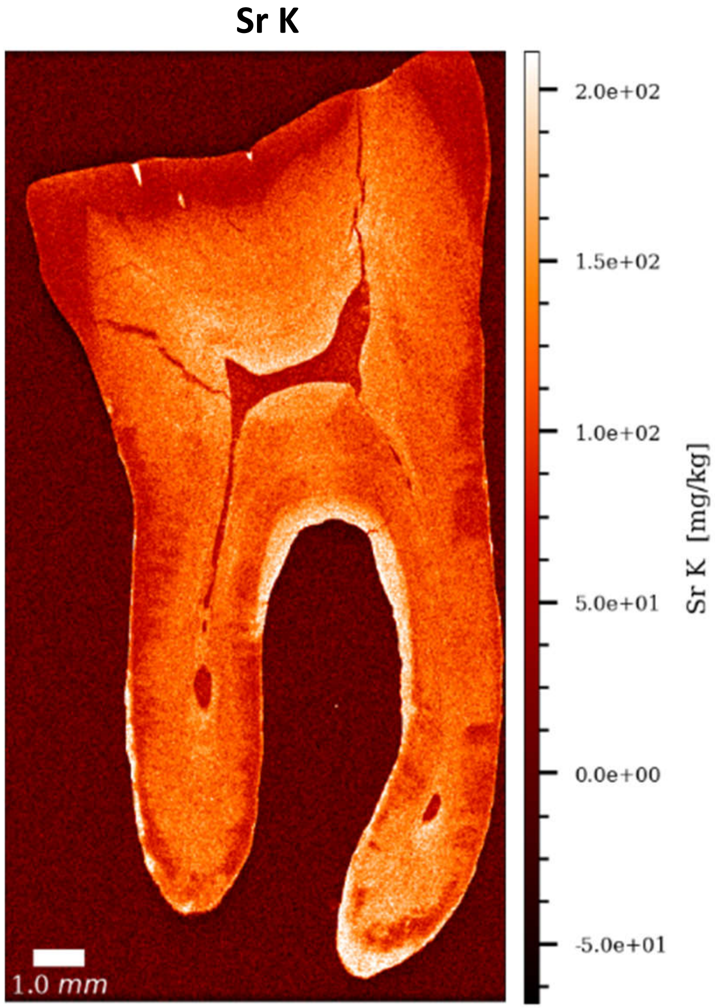

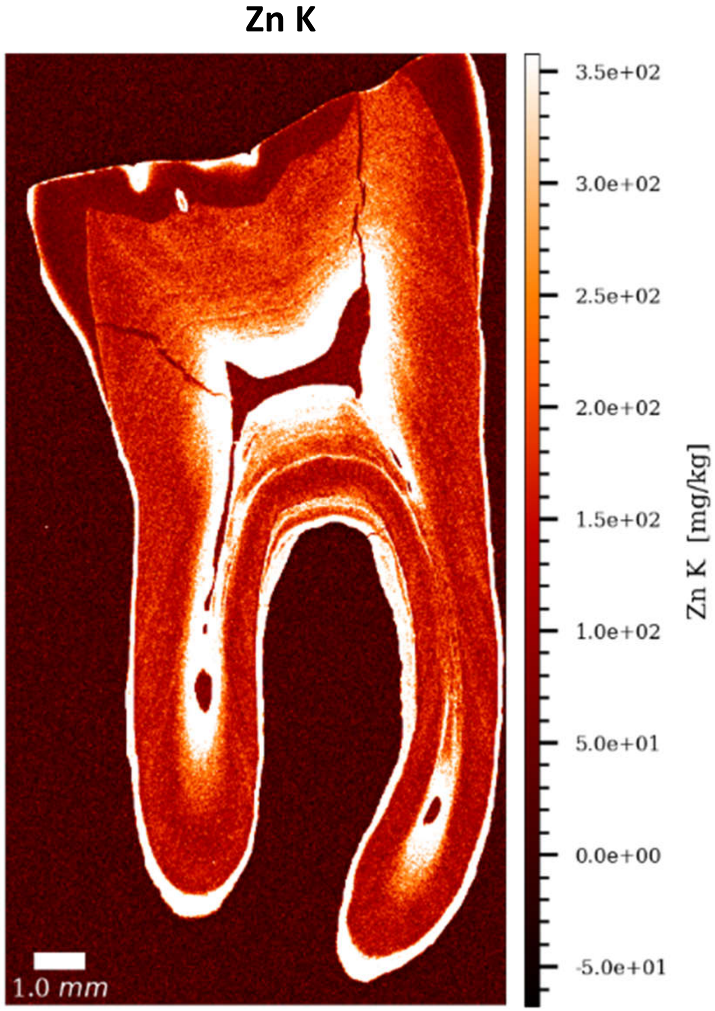

Næstved 211 LRM1

Overview at 10 µm

Gauss (1x1)

Cu K

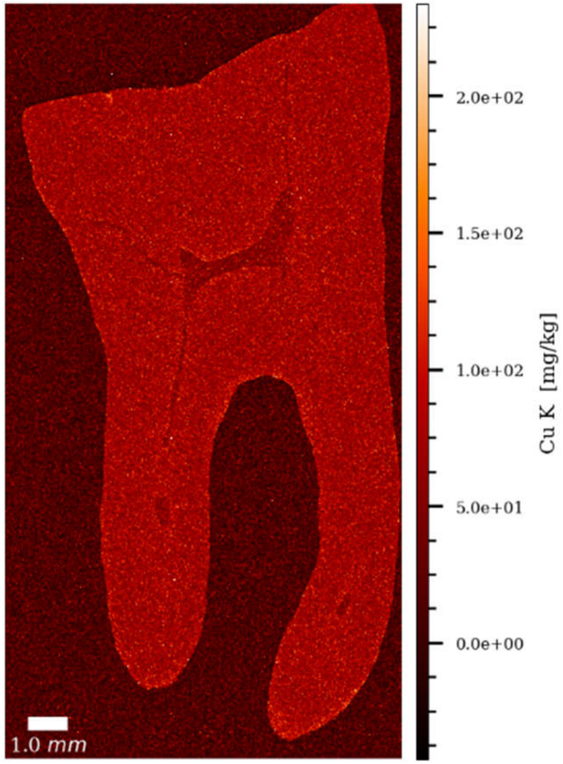

Fe K

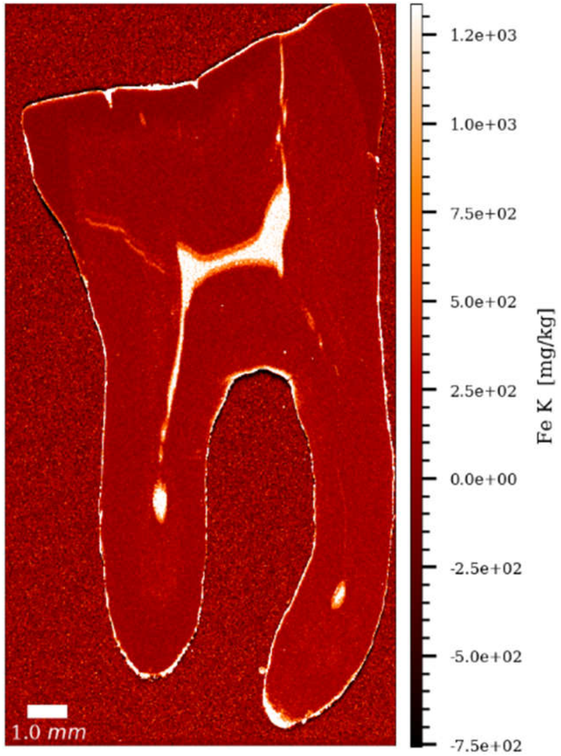

Mn K

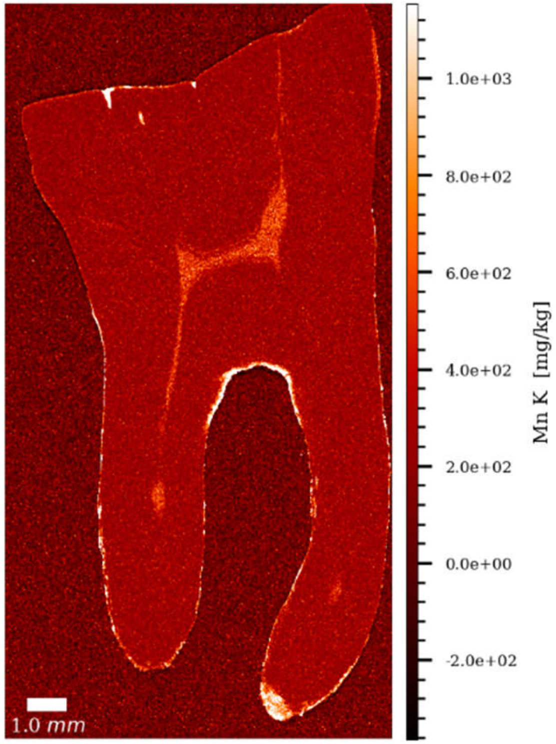

Næstved 211 LRM1

Overview at 10 µm

Uncalibrated data (arbitrary units)

Gauss (1x1)

Br K

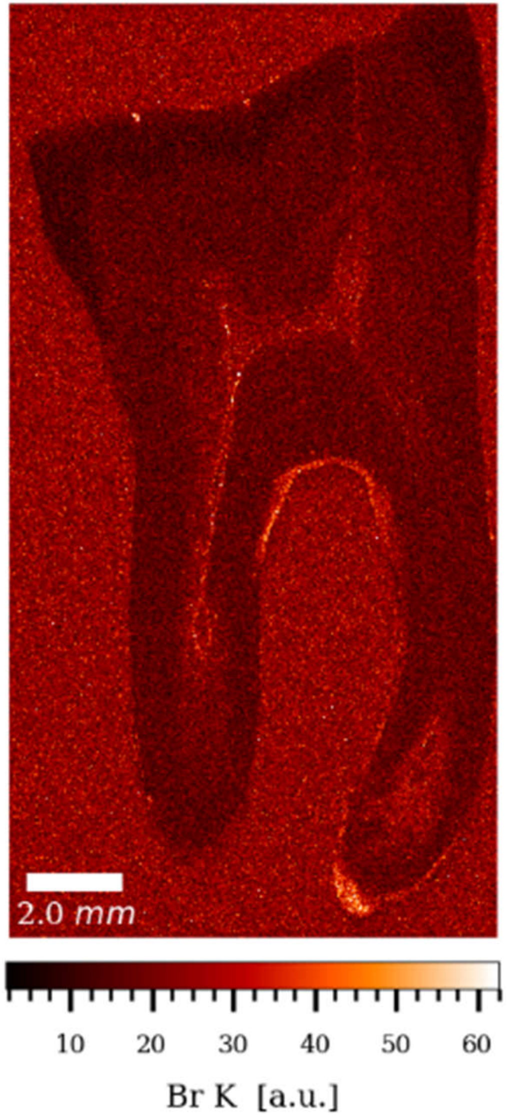

Pb L

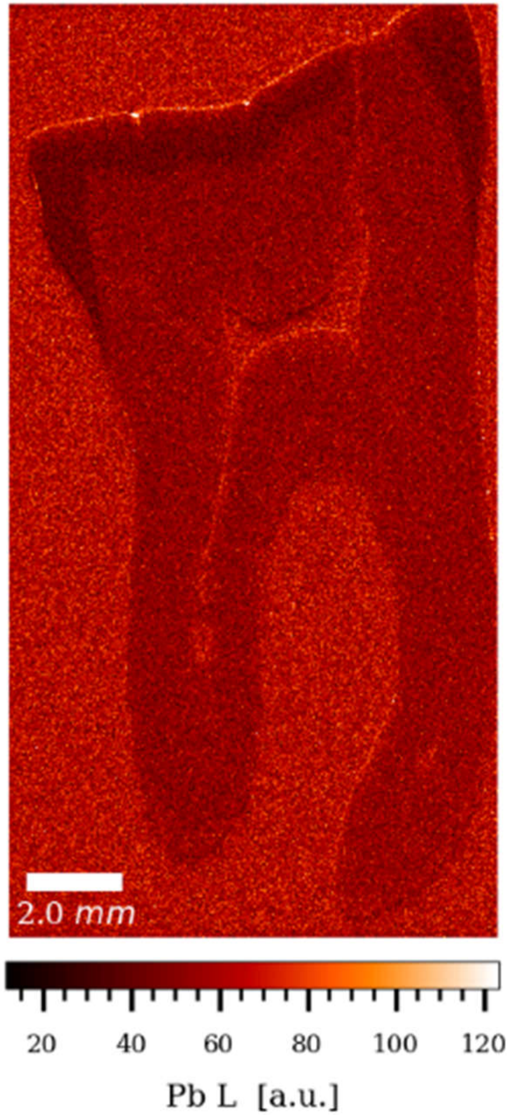

Næstved 211 LRM1

High resolution at 1.5 µm

Gauss (1x1)

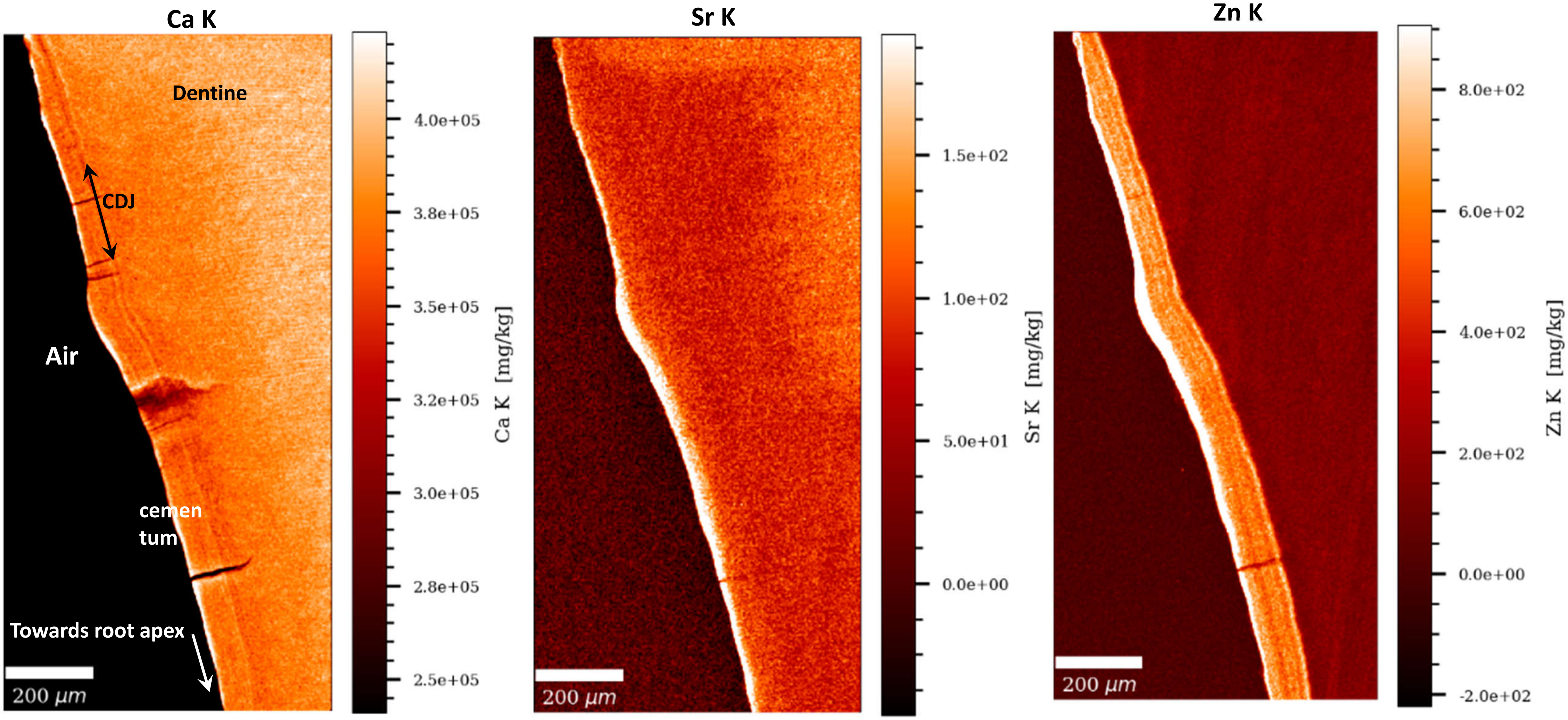

Næstved 211 LRM1

High resolution at 1.5 µm

Gauss (1x1)

Cu K

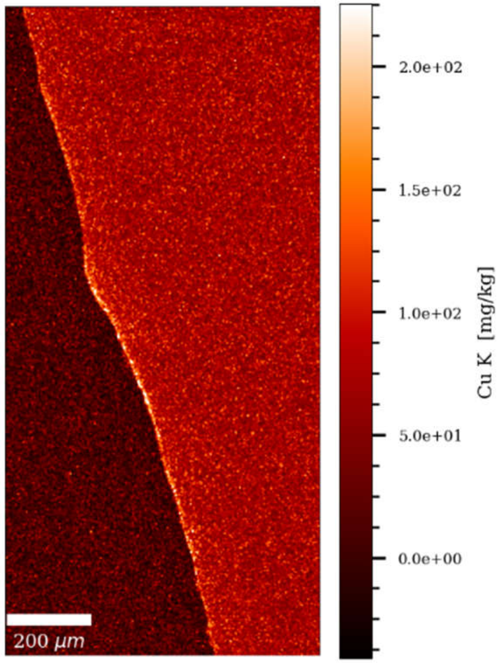

Fe K

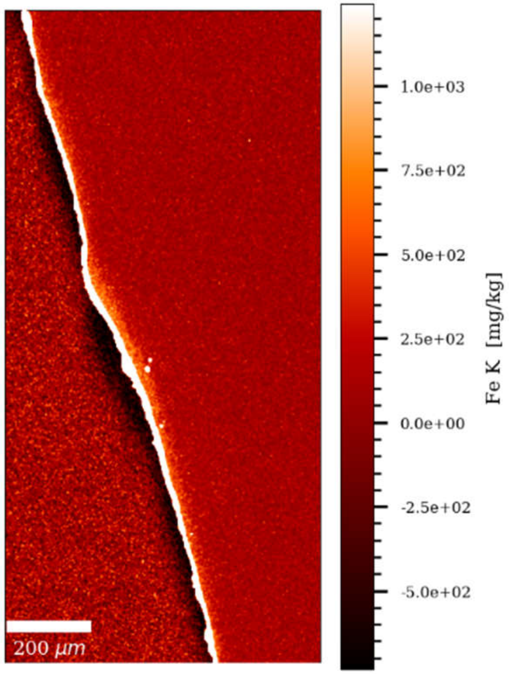

Mn K

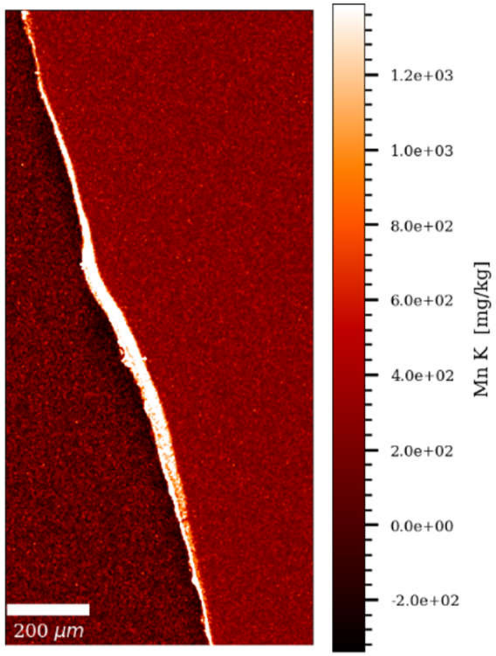

Næstved – 268 LLM1

♂ 30-40 yrs. 1441 – 1522 cal. CE

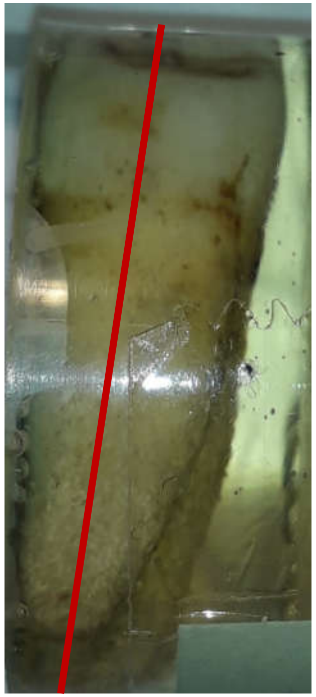

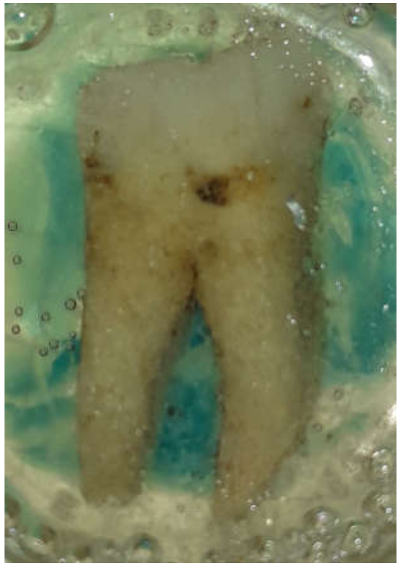

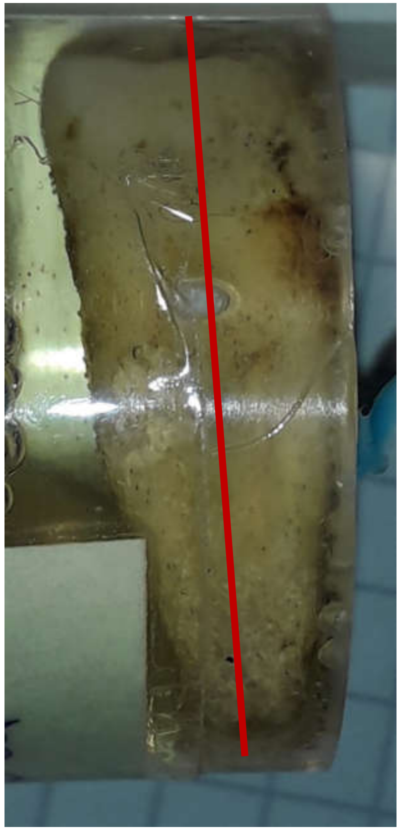

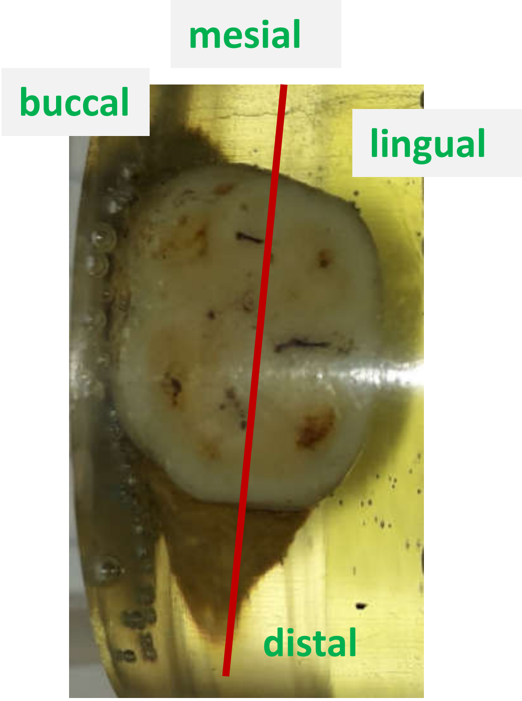

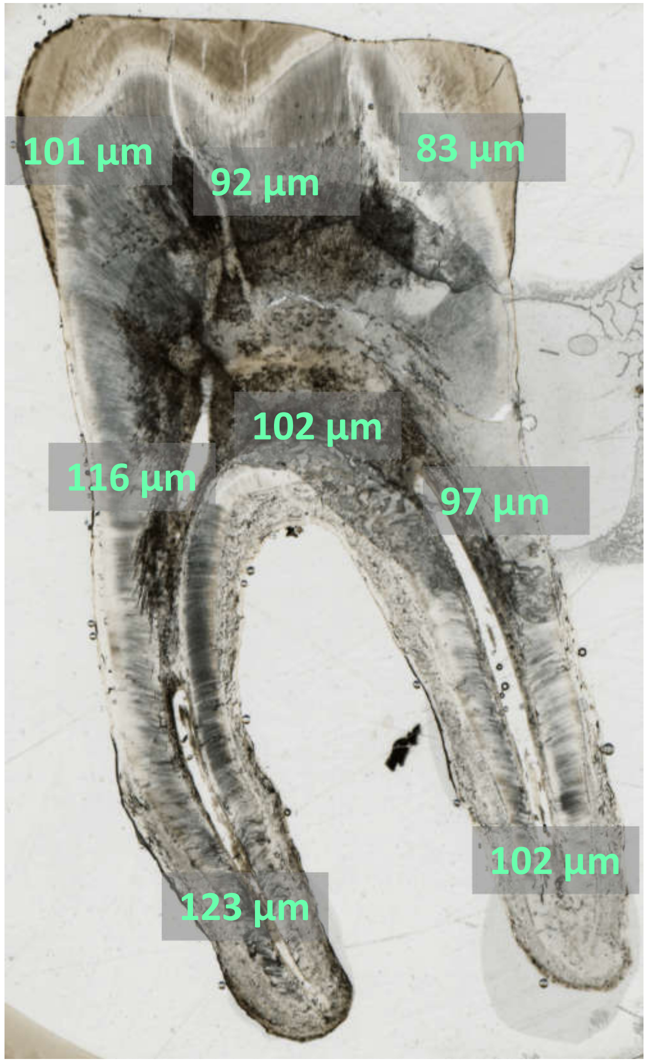

Average tooth section thickness (µm): 102.0

Næstved 268 LLM1

Scanning

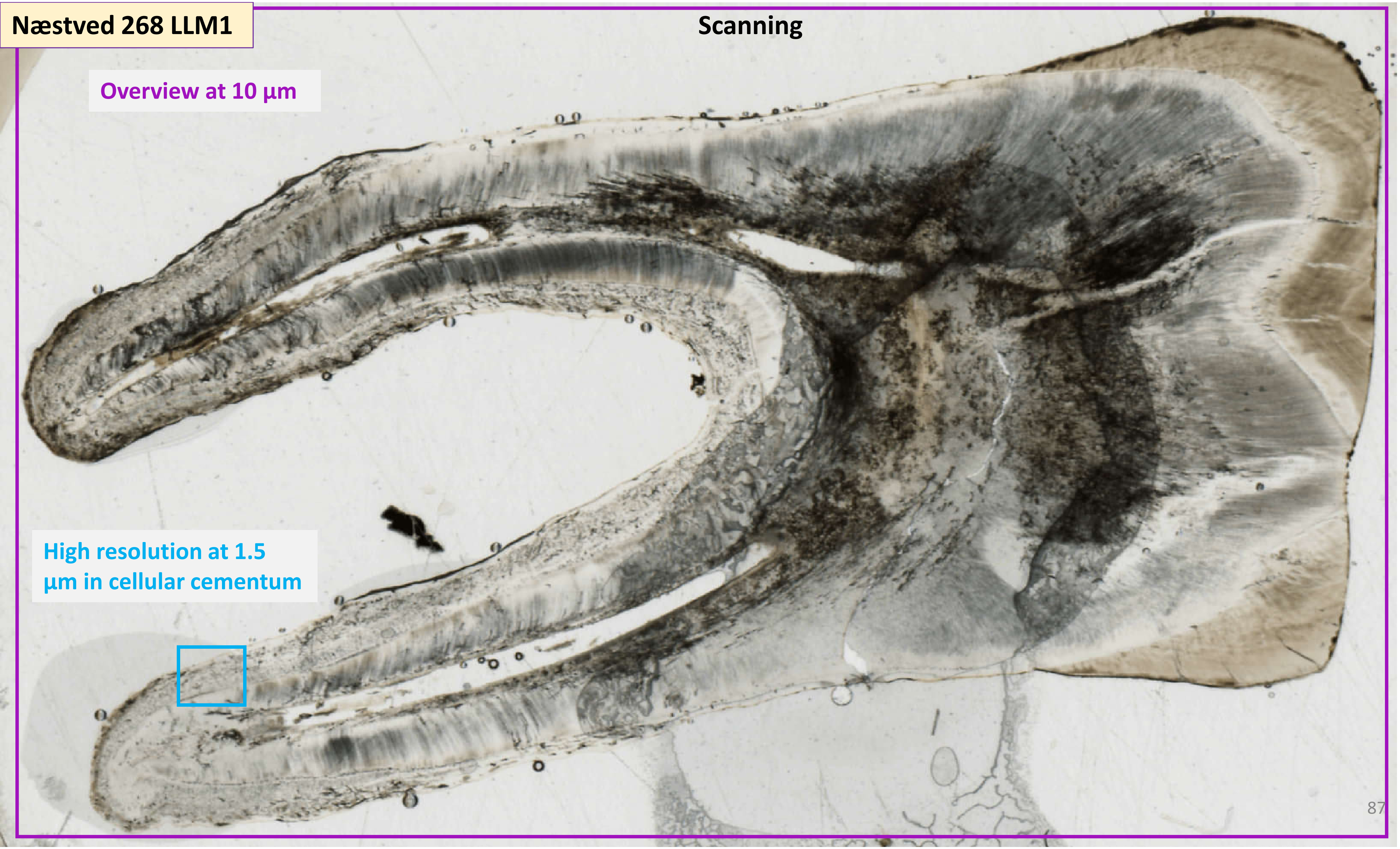

Næstved 268 LLM1

Overview at 10 µm

Gauss (1.2x1.2)

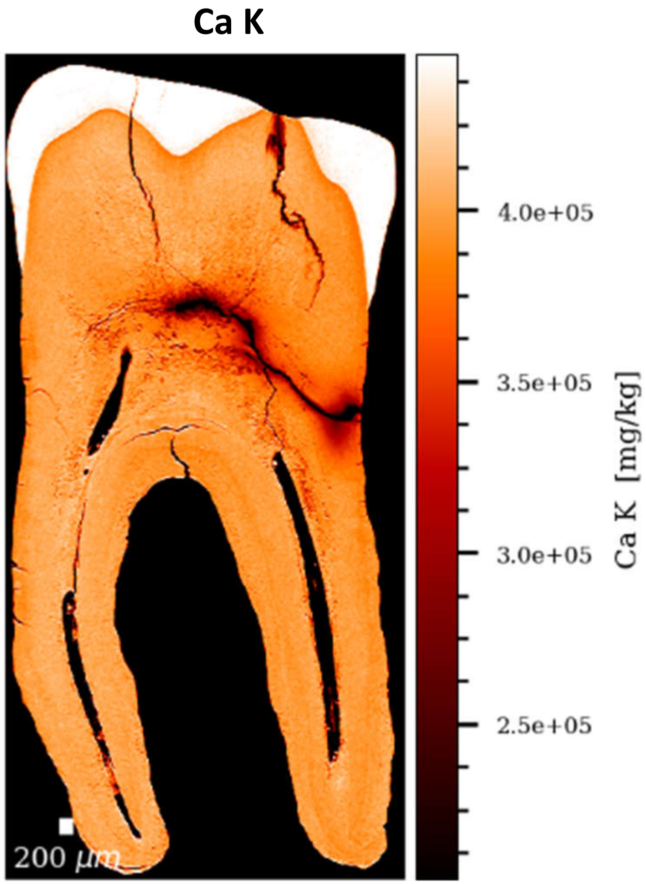

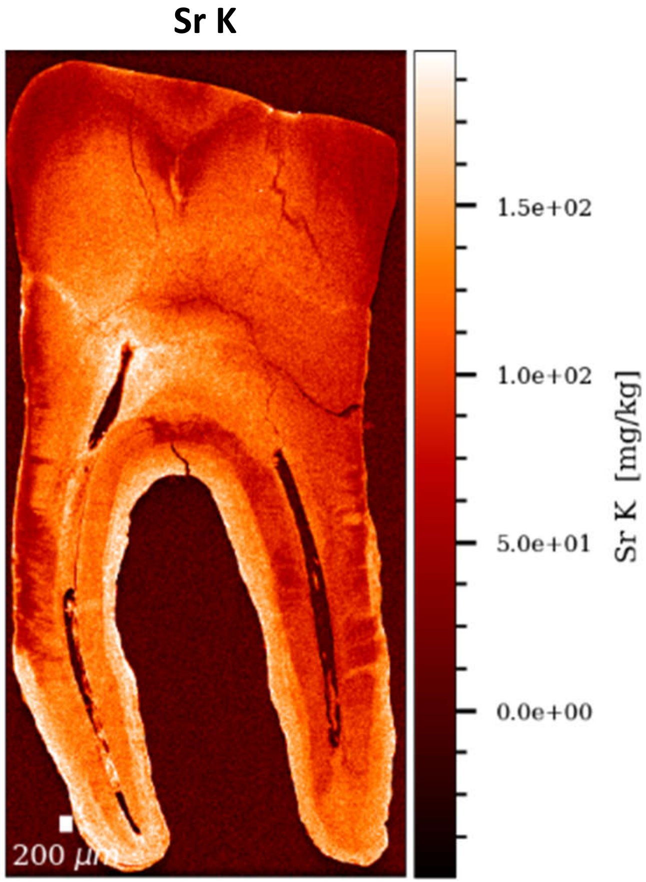

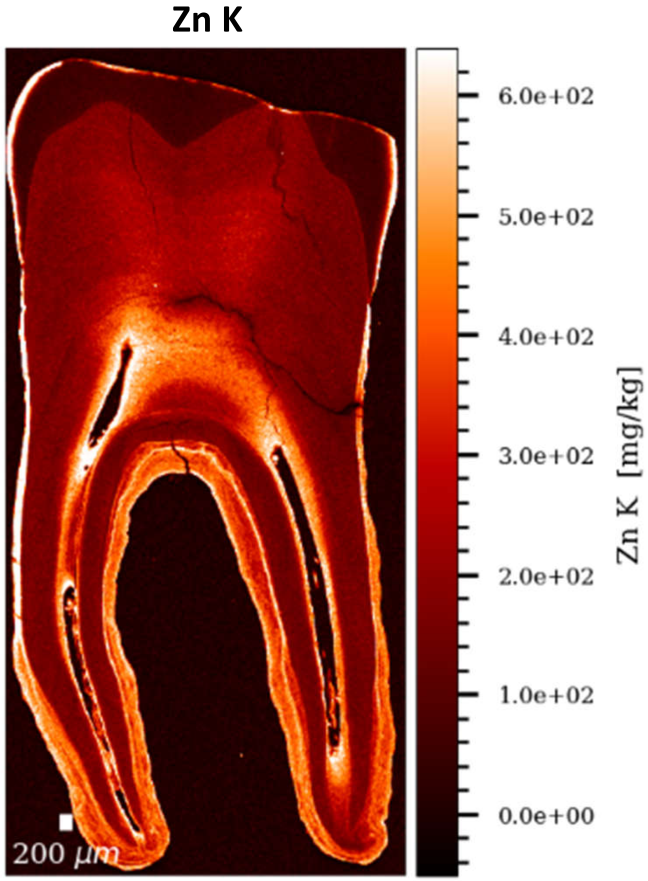

Næstved 268 LLM1

Overview at 10 µm

Gauss (1.2x1.2)

Cu K

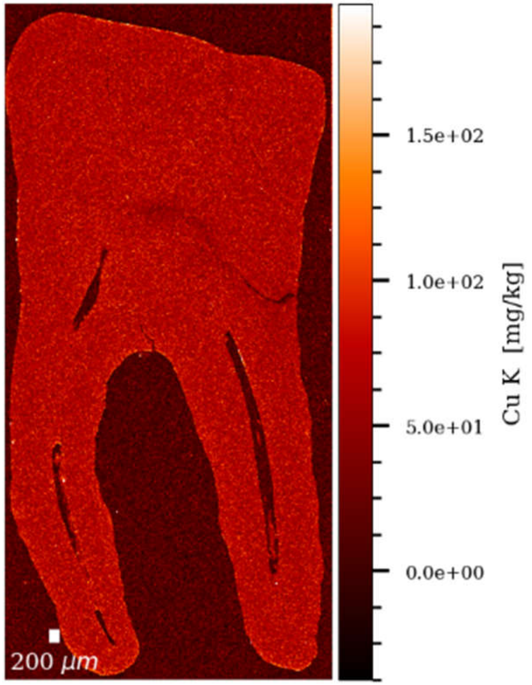

Fe K

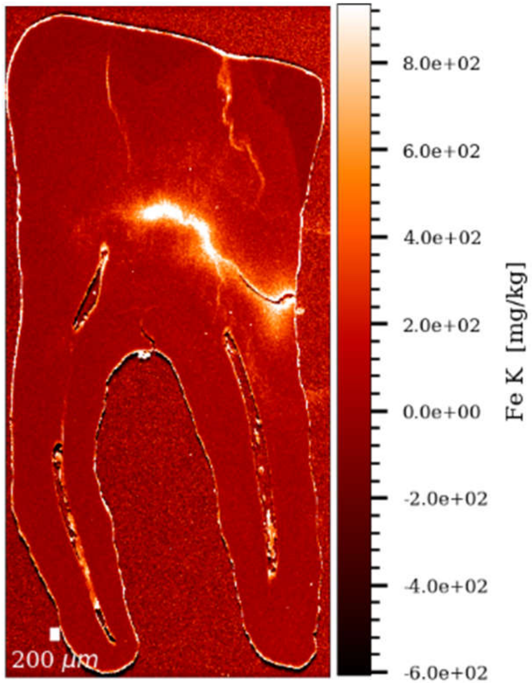

Mn K

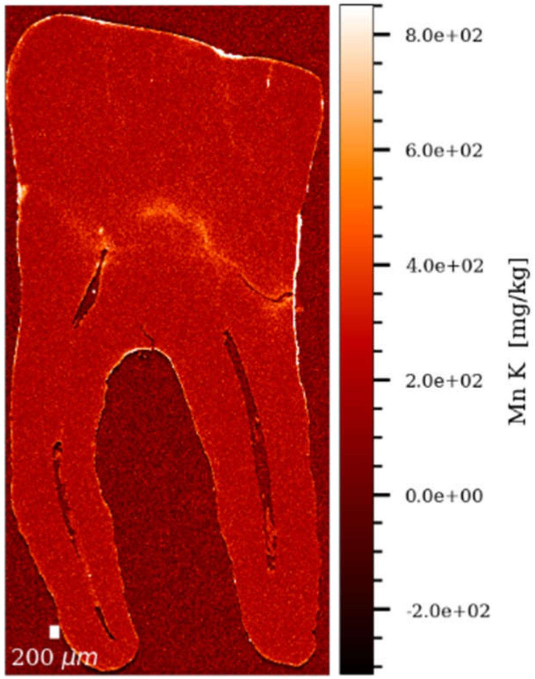

Næstved 268 LLM1

High resolution at 1 µm

Gauss (1x1)

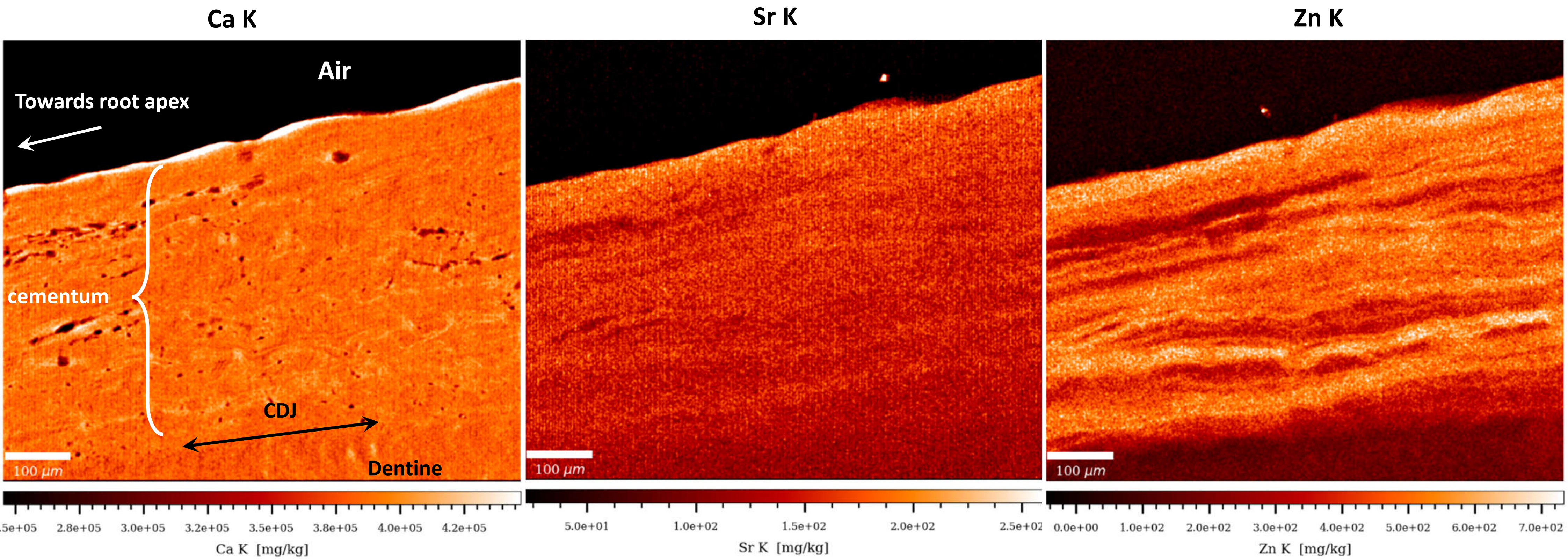

Næstved 268 LLM1

High resolution at 1 µm

Gauss (1x1)

Cu K

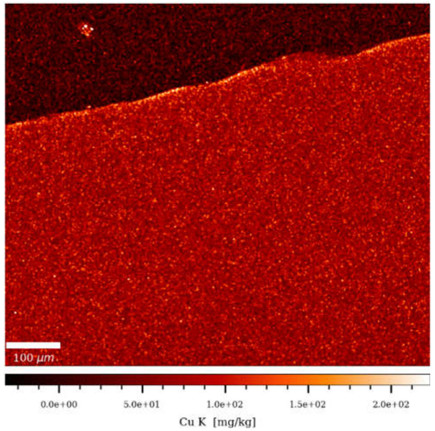

Mn K

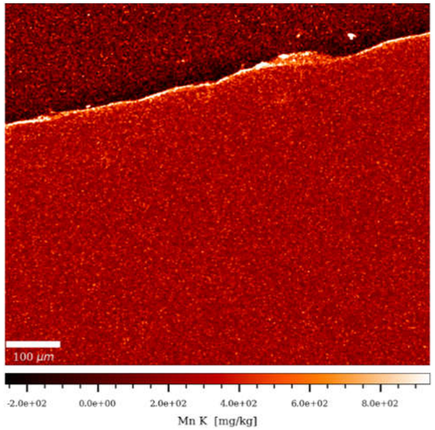

Fe K

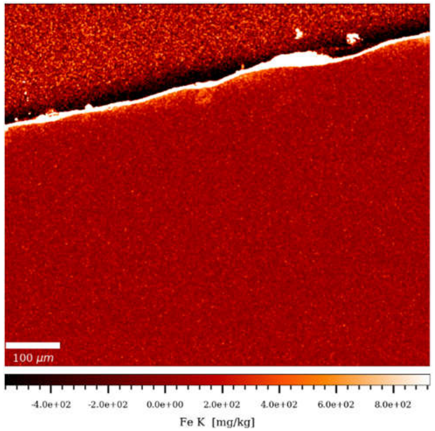

Pb L

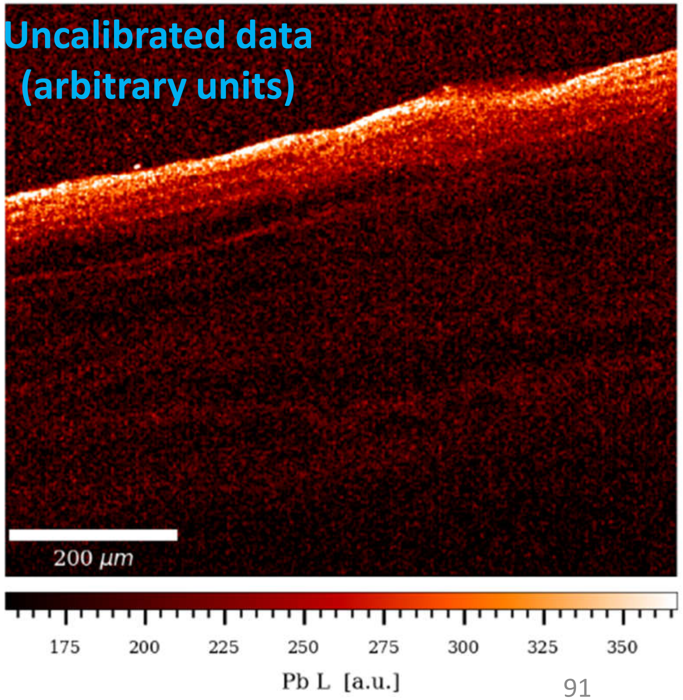

Næstved – 305 LLC

♂ 30-35 yrs. mid-13th – mid-16th c. CE

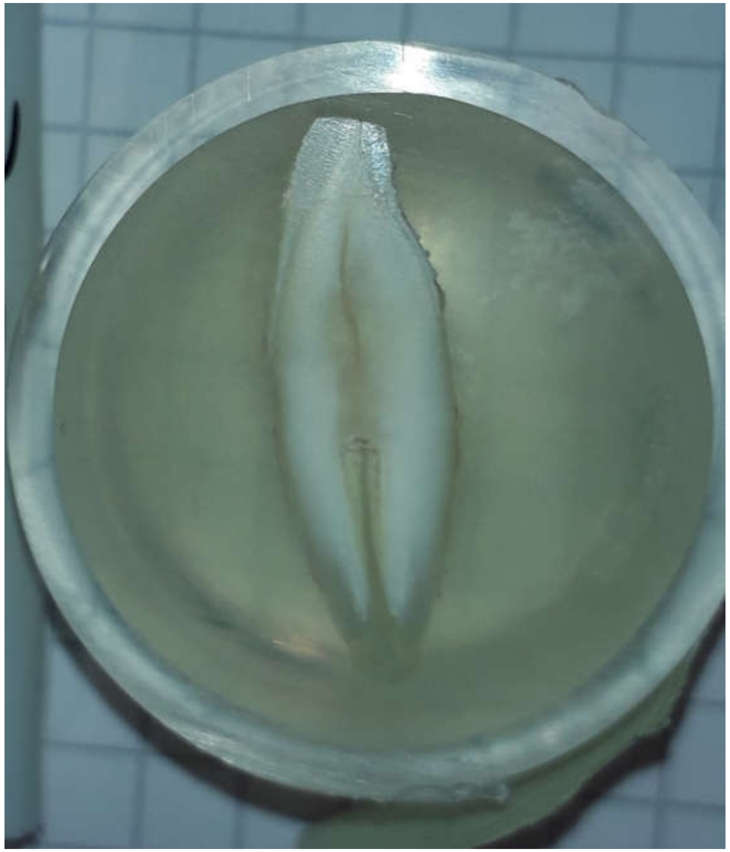

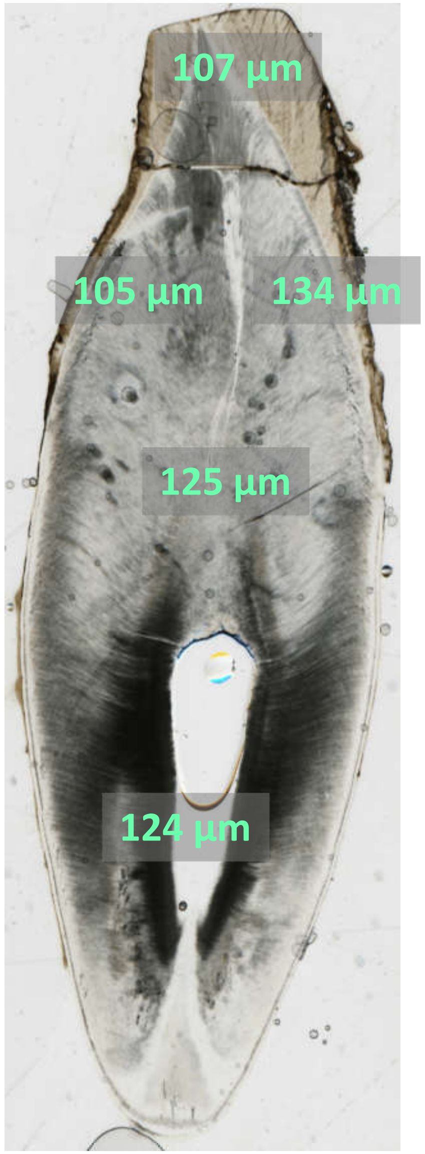

Average tooth section thickness (µm): 119.0

Næstved 305 LLC

Scanning

Overview at 10 µm

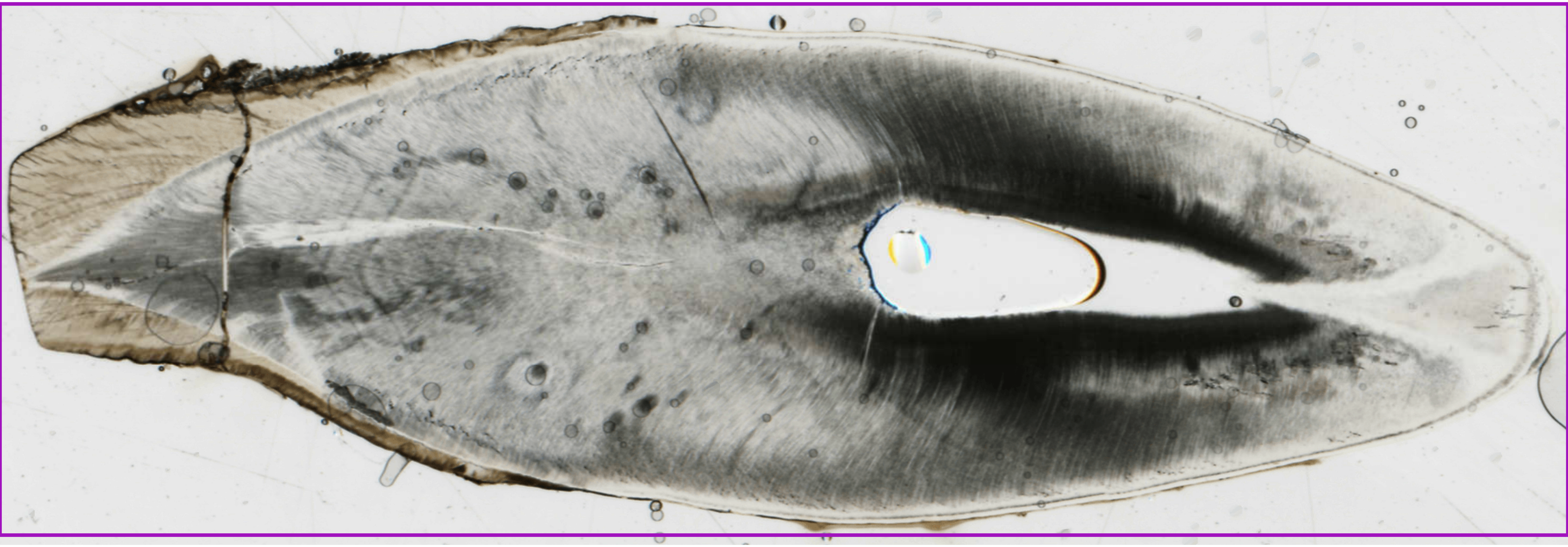

Næstved 305 LLC

Overview at 10 µm

Gauss (1.2x1.2)

Ca K

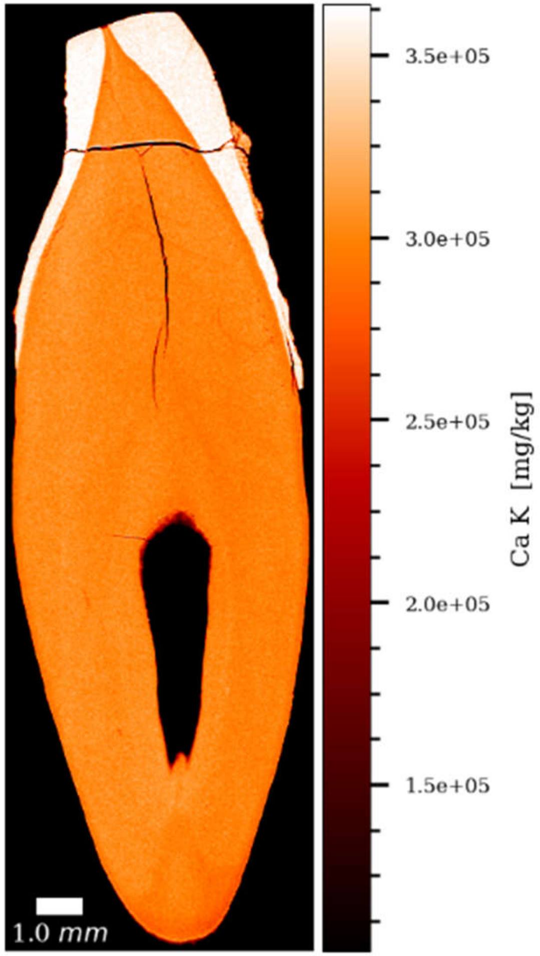

Sr K

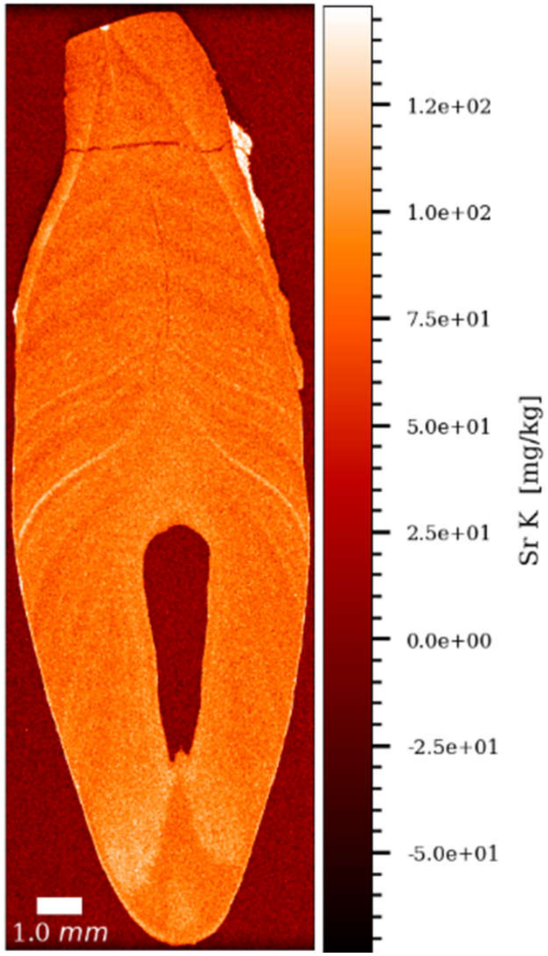

Zn K

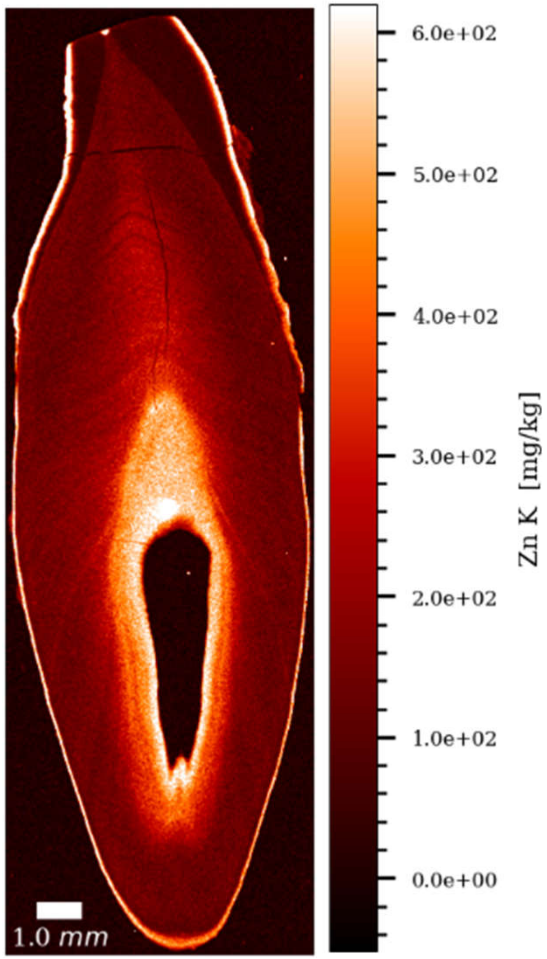

Næstved 305 LLC

Overview at 10 µm

Gauss (1.2x1.2)

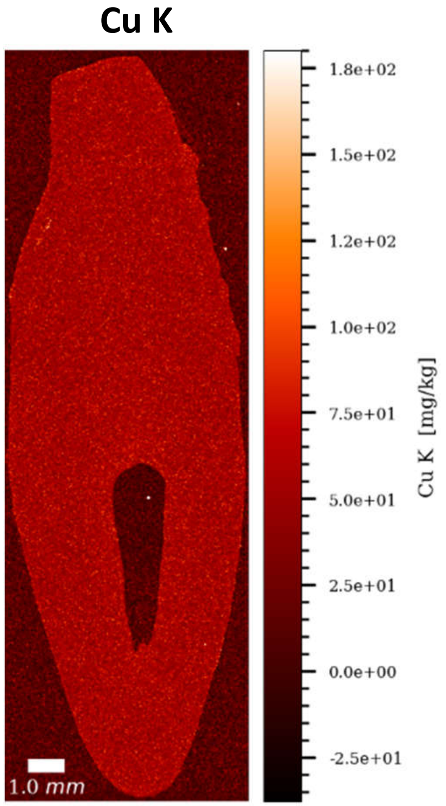

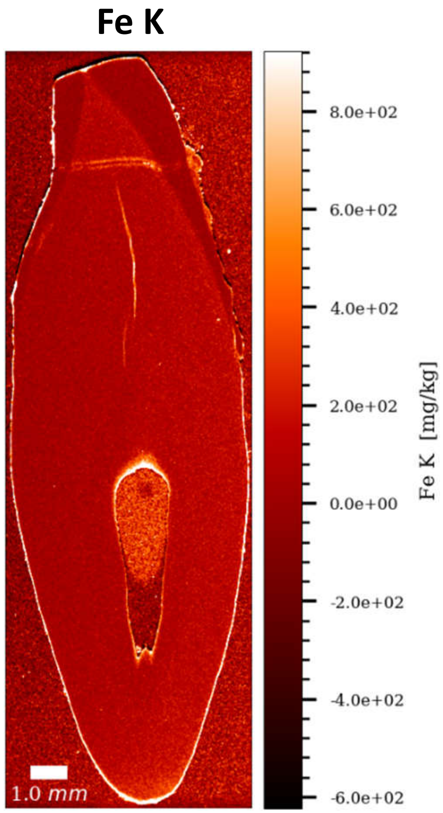

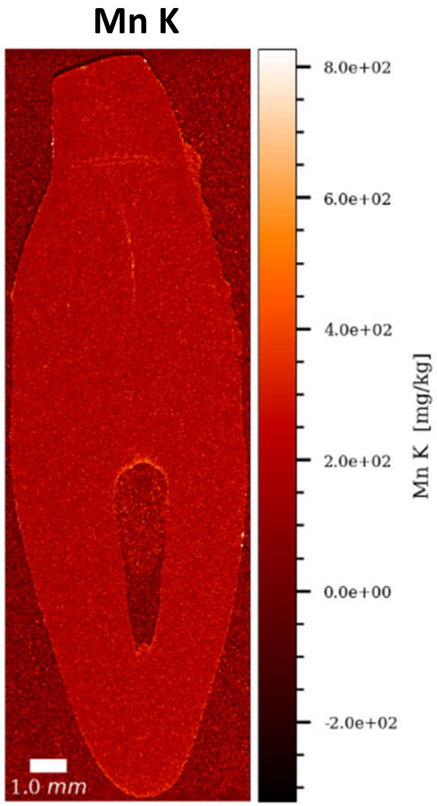

Næstved 305 LLC

Overview at 10 µm

Uncalibrated data
(arbitrary units)

Gauss (1.2x1.2)

Br K

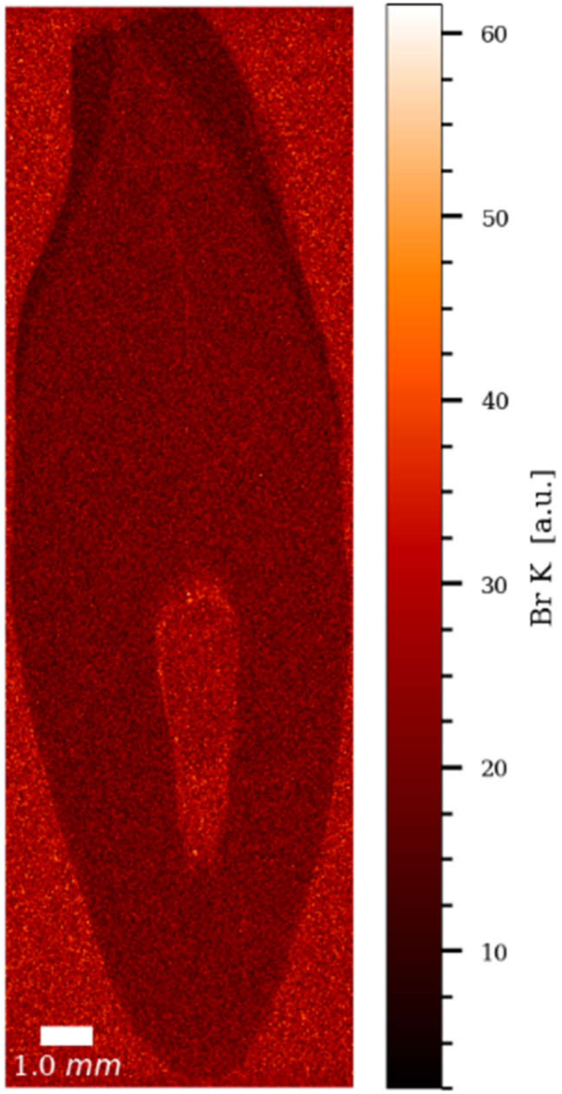

Pb L

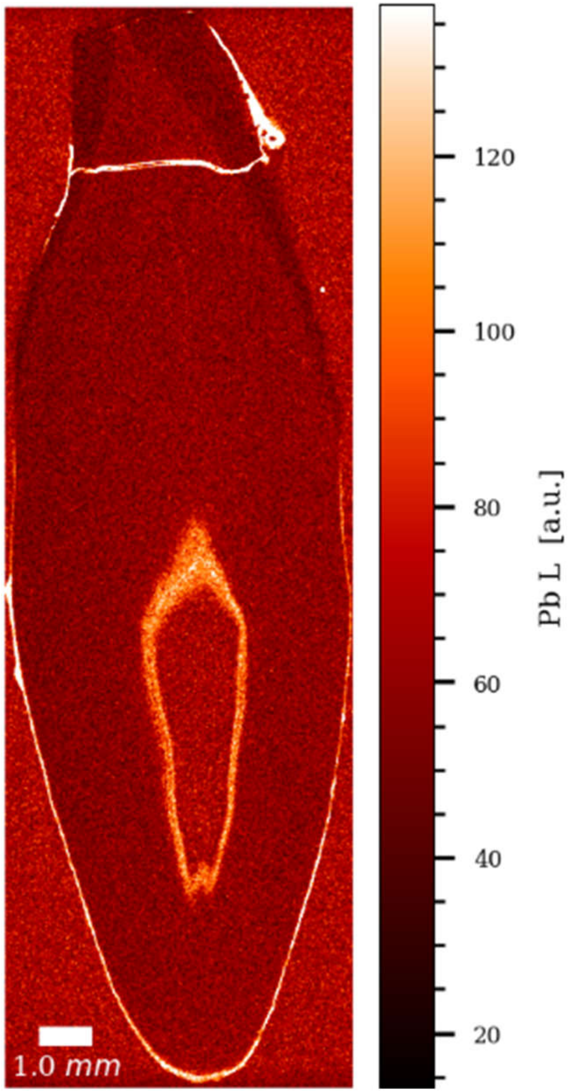

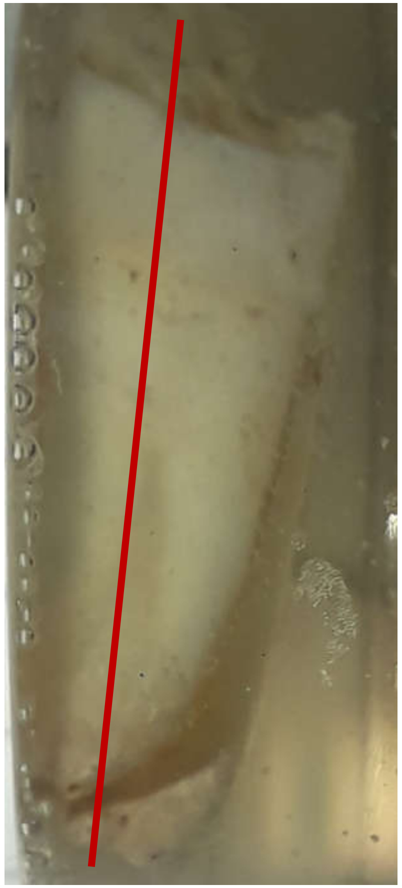

Næstved – 305 LRM1

♂ 30-35 yrs. mid-13th – mid-16th c. CE

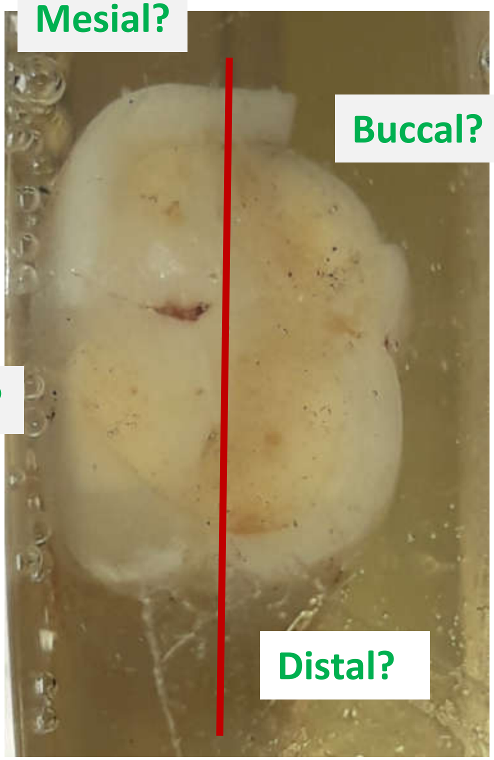

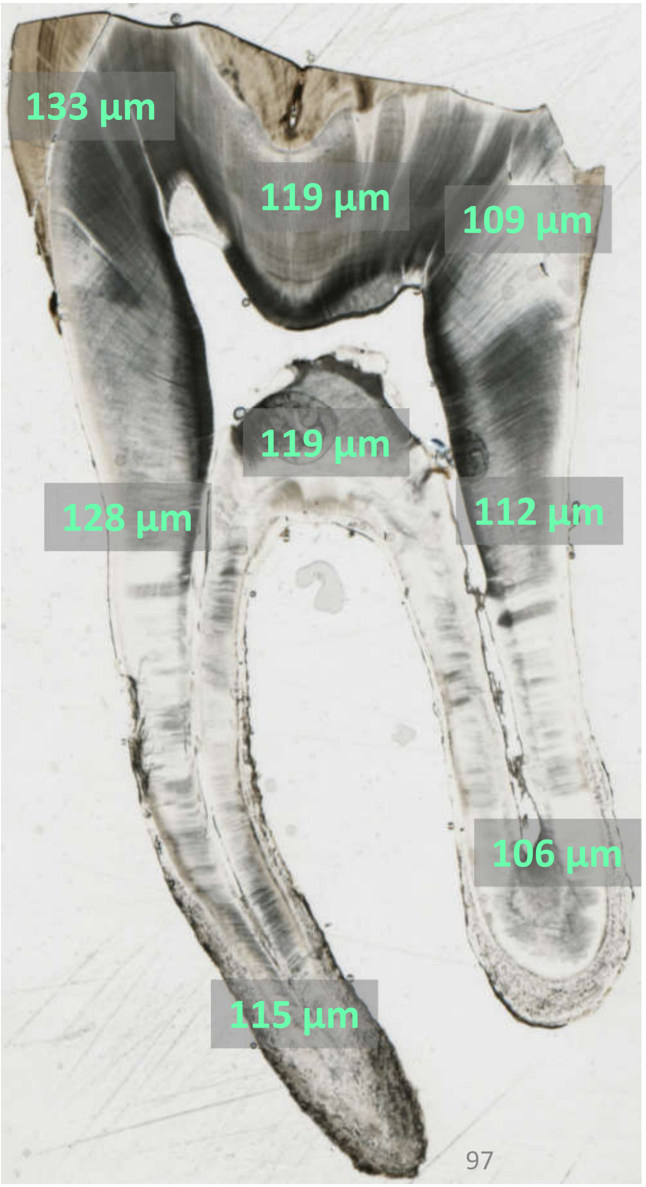

Average tooth section thickness (µm): 117.6

Næstved 305 LRM1

Scanning

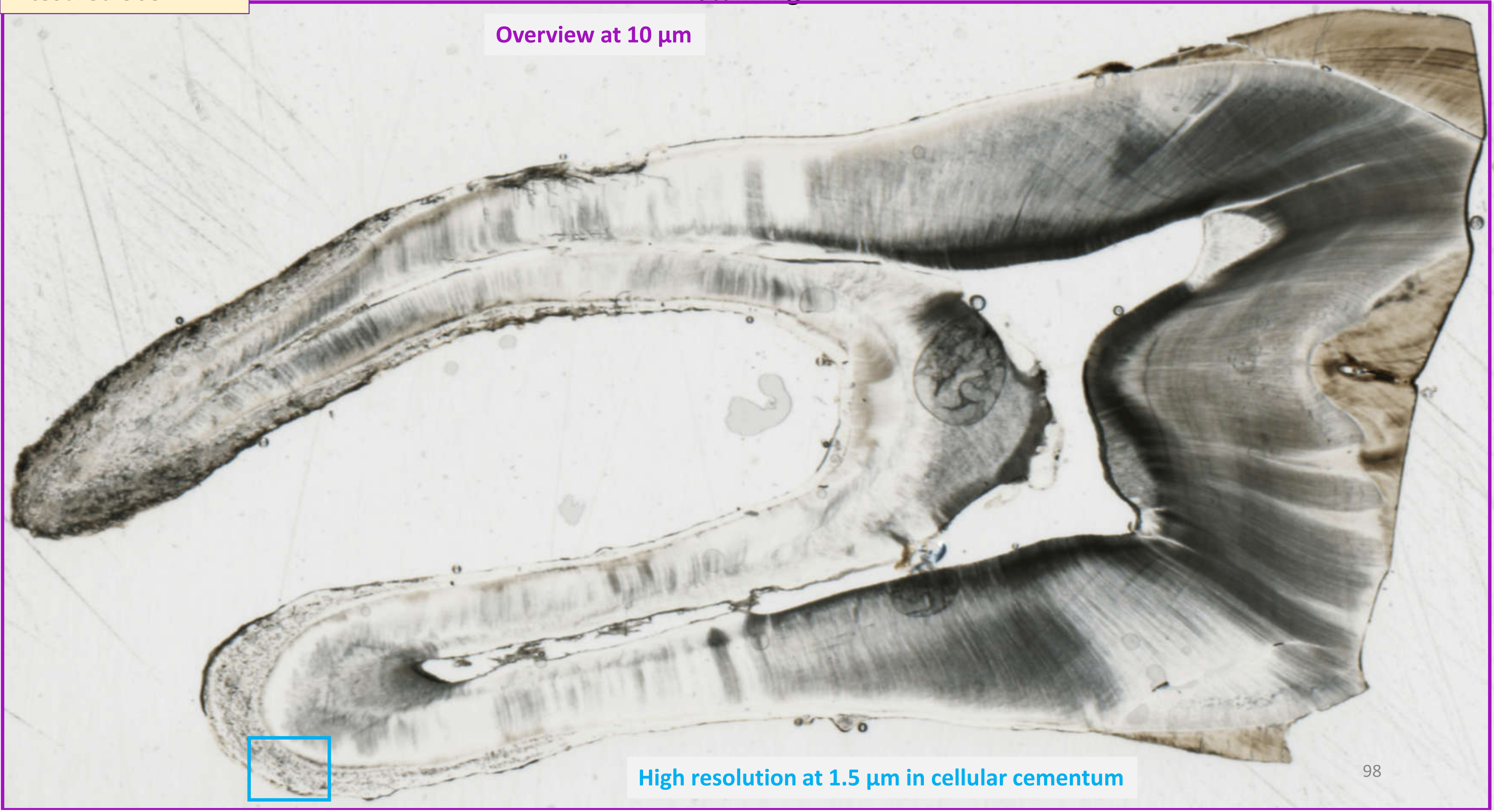

Næstved 305 LRM1

Overview at 10 µm

Gauss (1x1)

Ca K **Sr K** **Zn K**

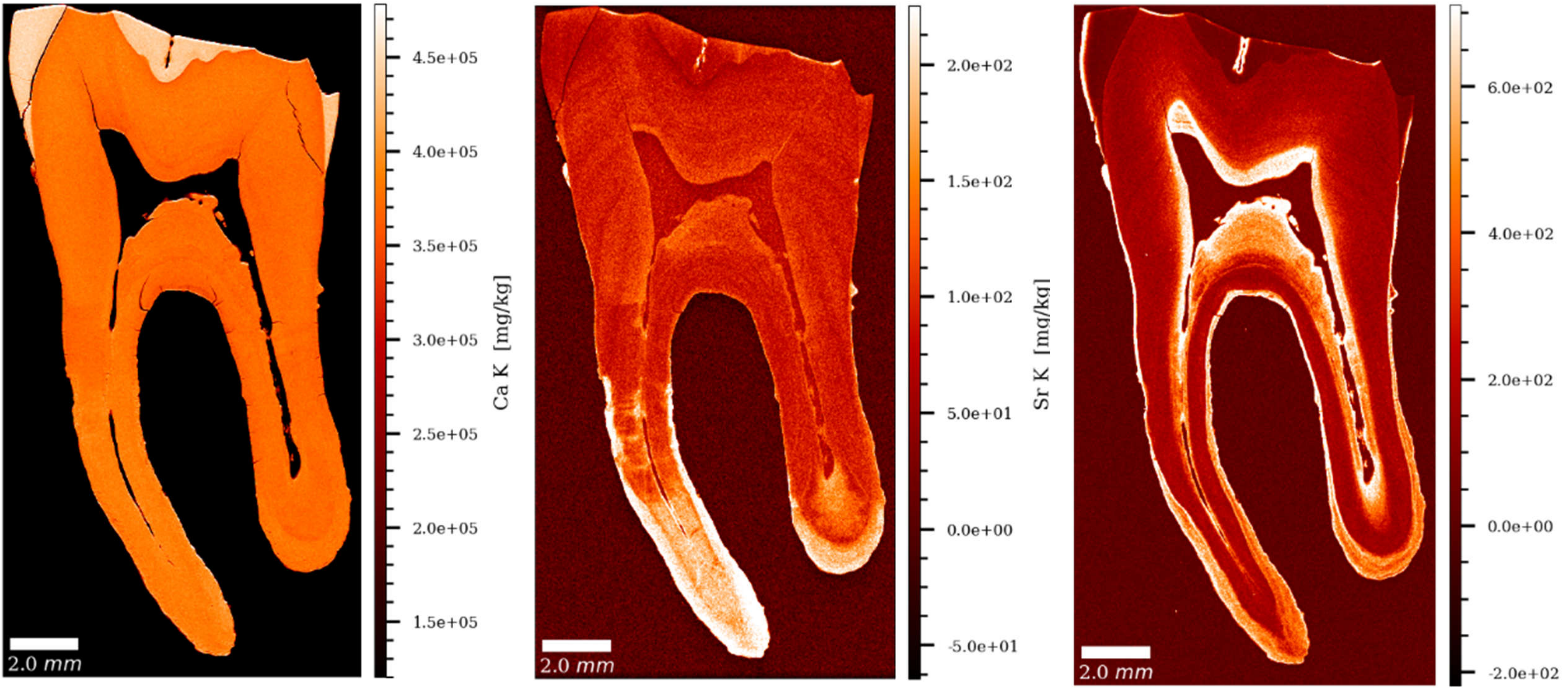

Næstved 305 LRM1

Overview at 10 µm

Gauss (1x1)

Cu K

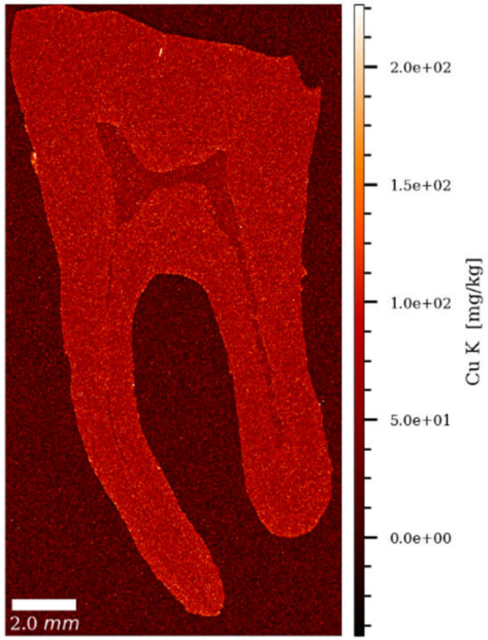

Fe K

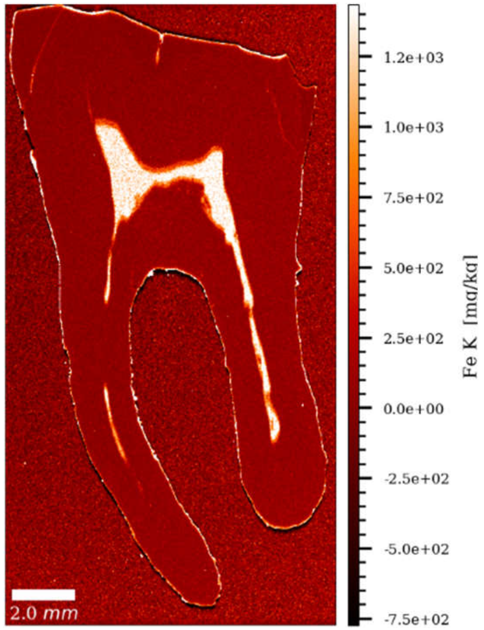

Mn K

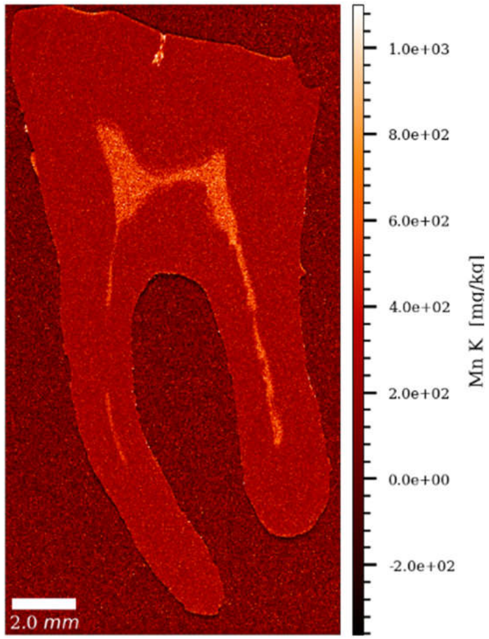

Næstved 305 LRM1

Overview at 10 µm

Uncalibrated data
(arbitrary units)

Gauss (1x1)

Pb L

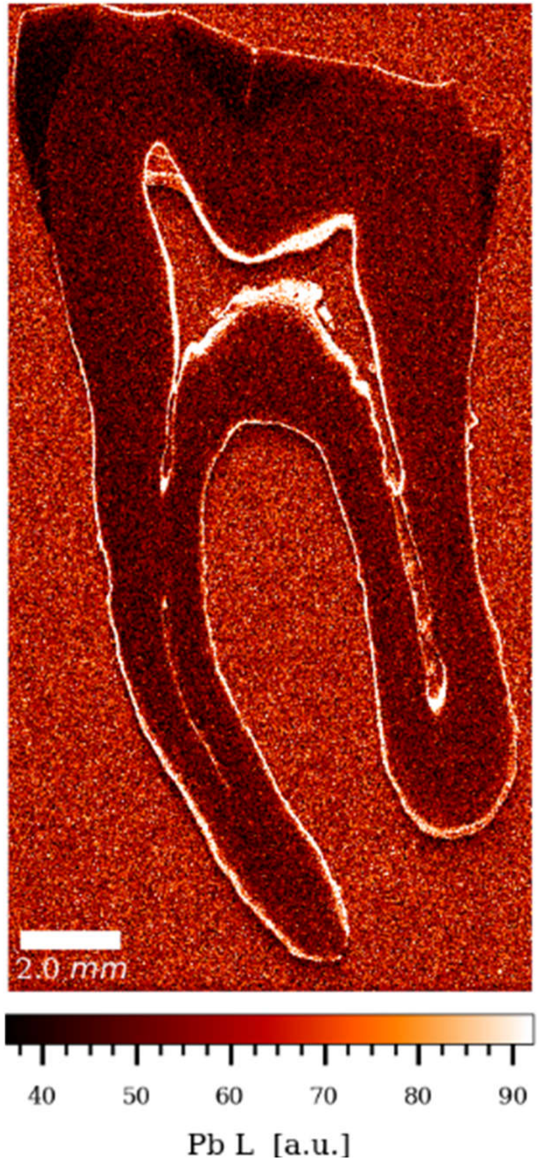

Br K

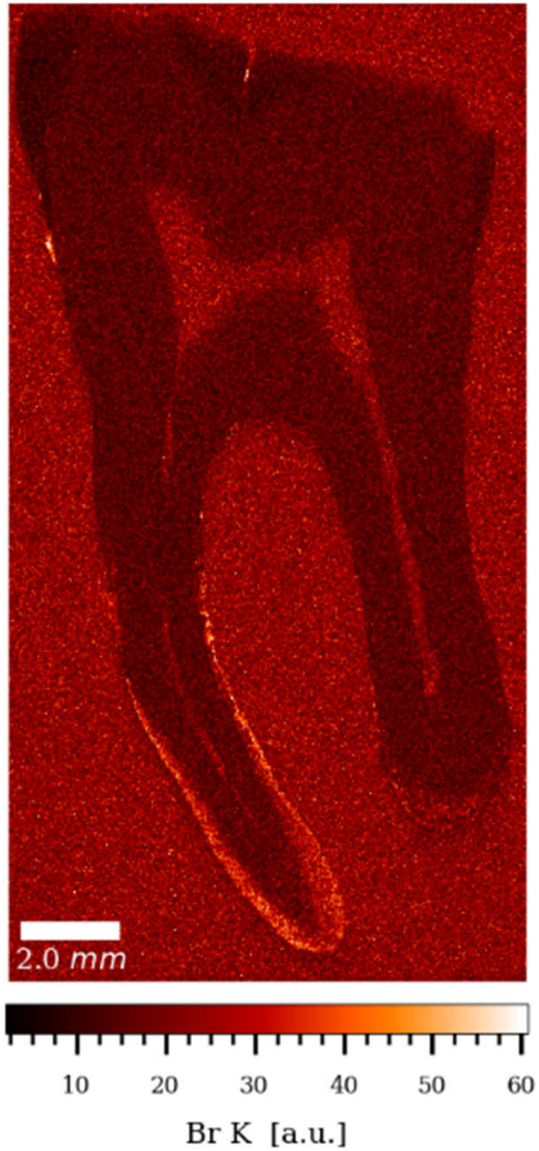

Næstved 305 LRM1

High resolution at 1.5 µm

Gauss (1x1)

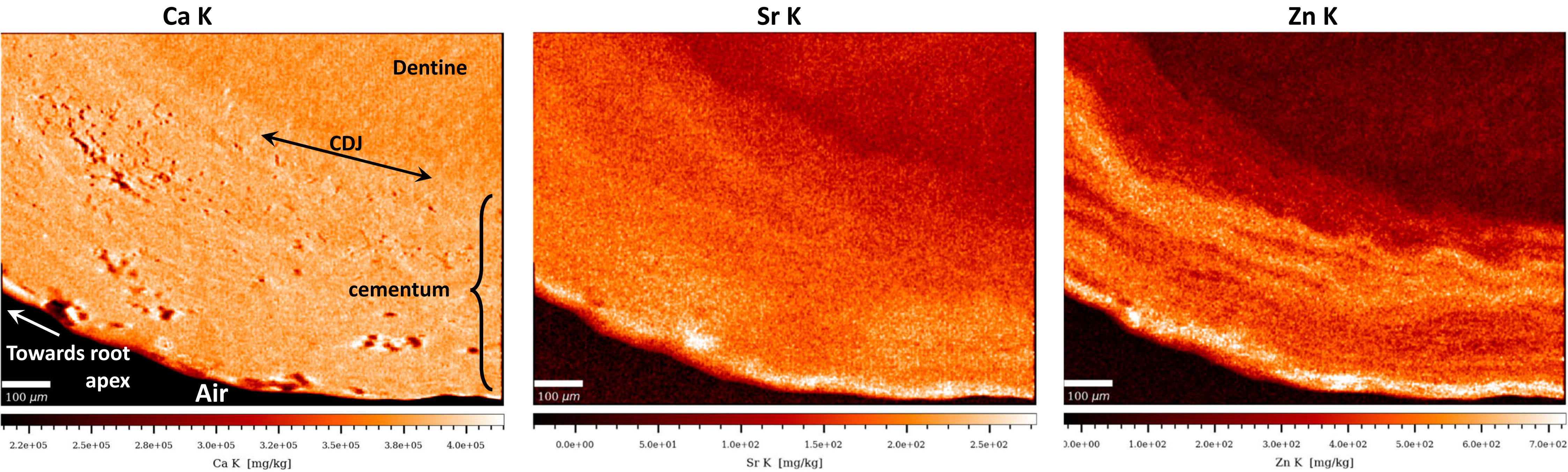

Næstved 305 LRM1

High resolution at 1.5 µm

Gauss (1x1)

Cu K

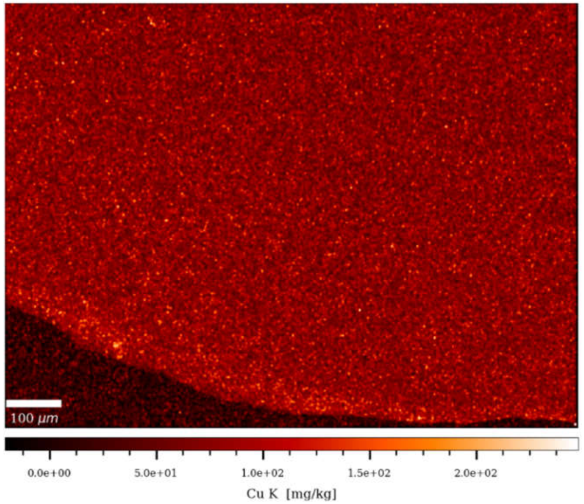

Fe K

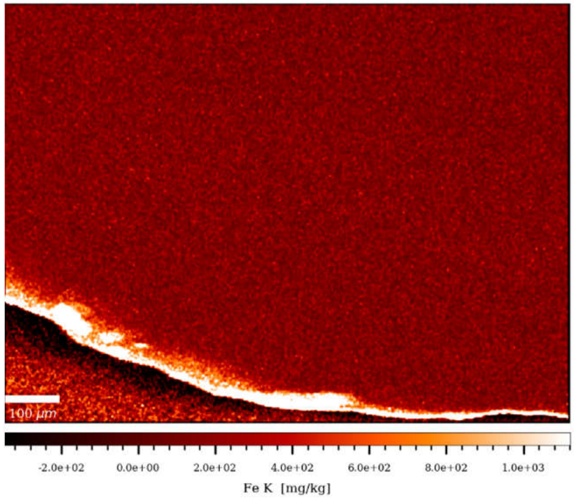

Mn K

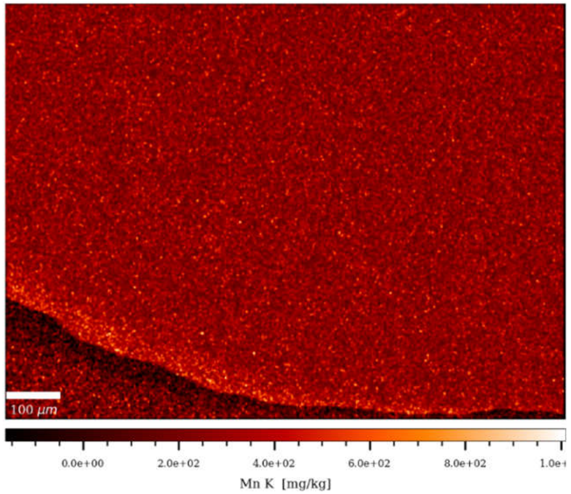

Supplement: Supplementary file 1 [file biology-12-00184-s001.zip › biology-2121048-Supplementary Material/Brozou_et_al_File_S2.pdf]
