# Supplementary material for: Using SXRF and LA-ICP-TOFMS to Explore Evidence of Treatment and Physiological Responses to Leprosy in Medieval Denmark
Source: Biology (Basel). 2023 Jan 25;12(2):184. doi: 10.3390/biology12020184 (PMC9952905; doi:10.3390/biology12020184)

File S3

Laser Ablation (LA-ICP-TOFMS) results

Using SXRF and LA-ICP-TOFMS to explore evidence of treatment and physiological responses to leprosy in medieval Denmark

Biology

Anastasia Brozou, Marcello A. Mannino, Stijn J.M. Van Malderen, Jan Garrevoet, Eric Pubert, Benjamin T. Fuller, M. Christopher Dean, Thomas Colard, Frédéric Santos, Niels Lynnerup, Jesper L. Boldsen, Marie Louise Jørkov, Andrei Dorian Soficaru, Laszlo Vincze, Adeline Le Cabec

Corresponding author:

Adeline Le Cabec
E-mail: adeline.le-cabec@u-bordeaux.fr

Teeth with the best SXRF signal in cellular cementum.

Odense 533 LLM1:
The cellular cementum and the secondary dentine are also imaged with SXRF.
The acellular cementum is scanned here for comparison.

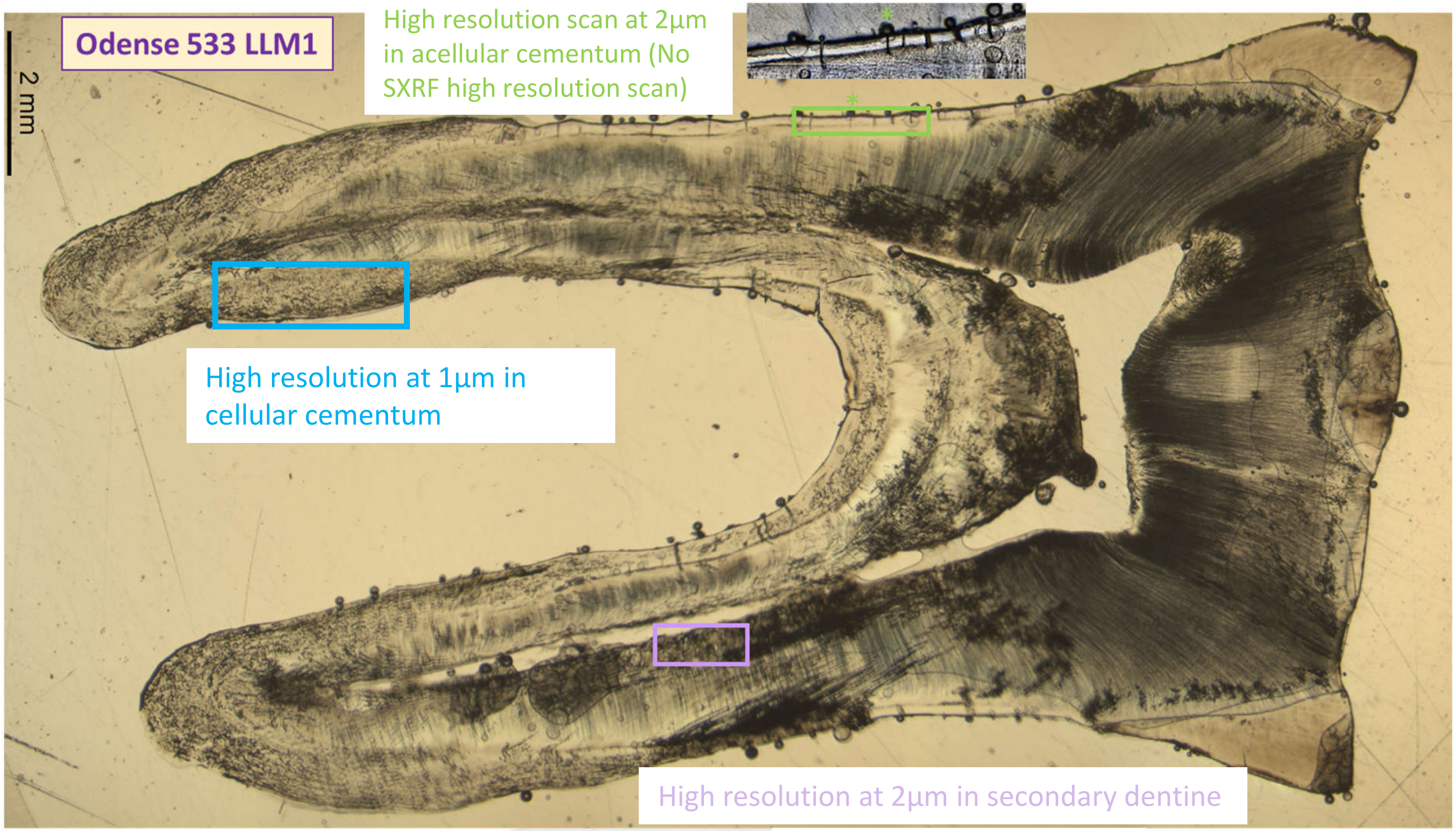

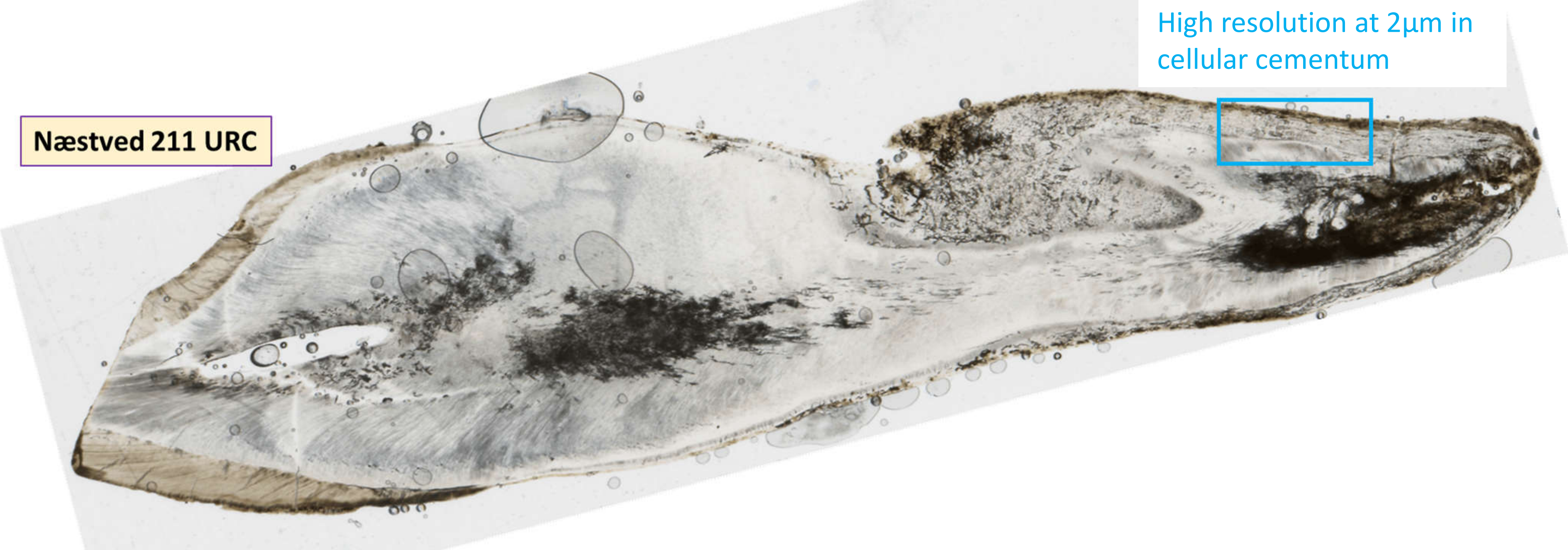

Naestved 211 URC – Cellular Cementum

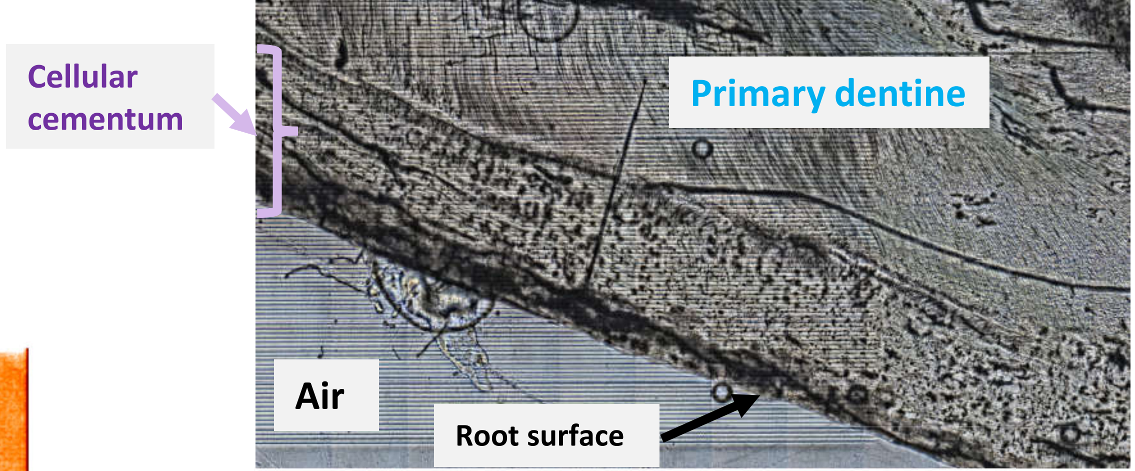

Gauss 0.8 x 0.8

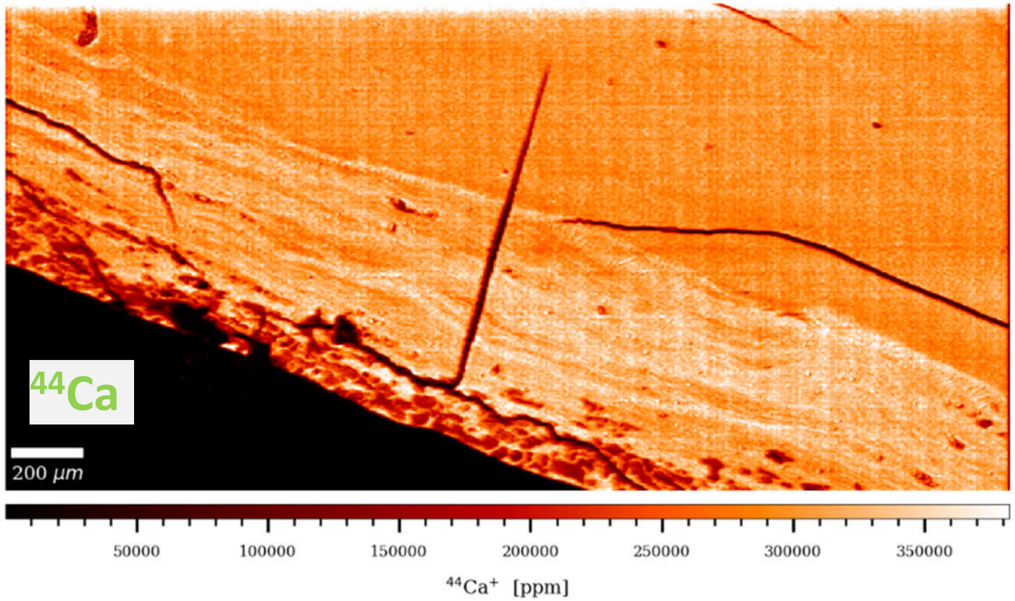

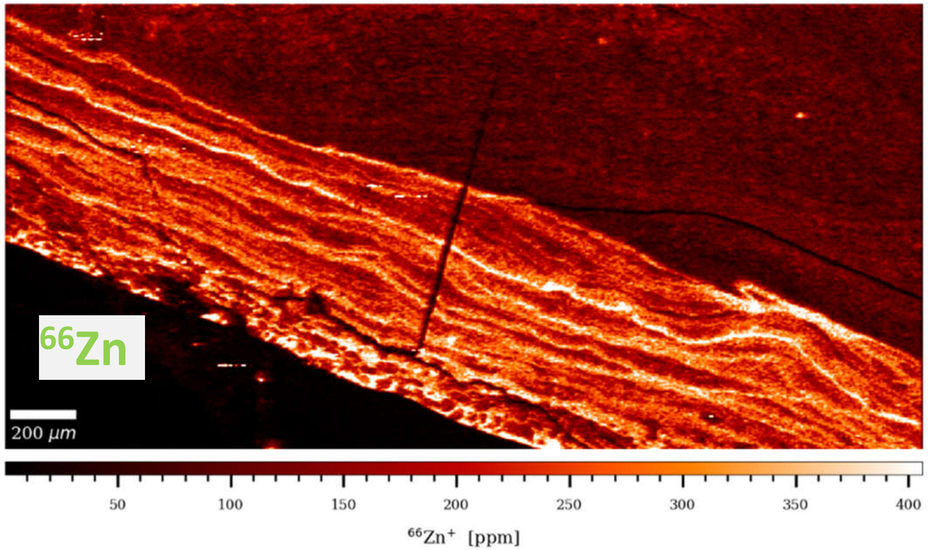

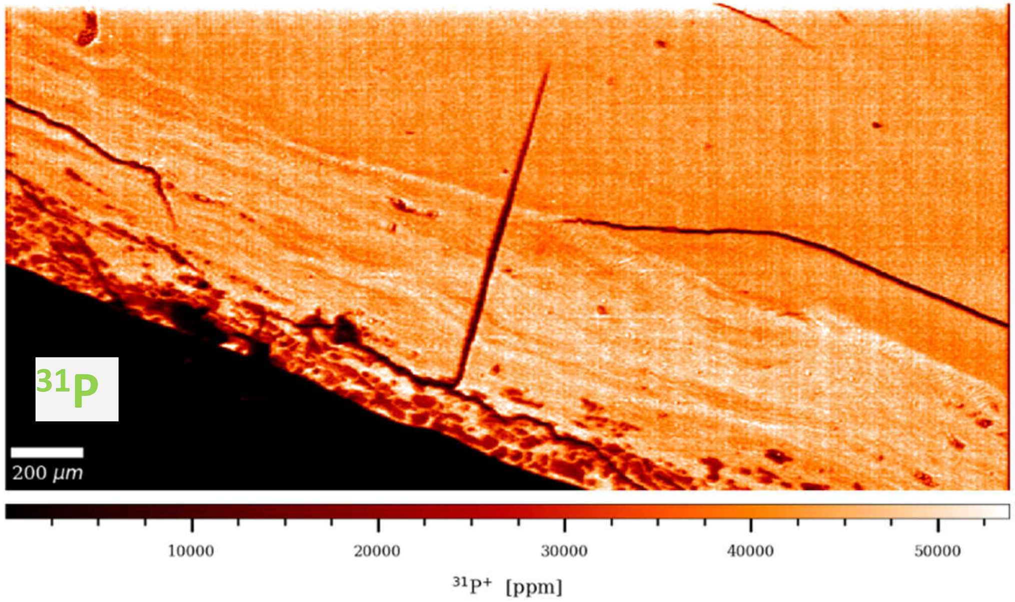

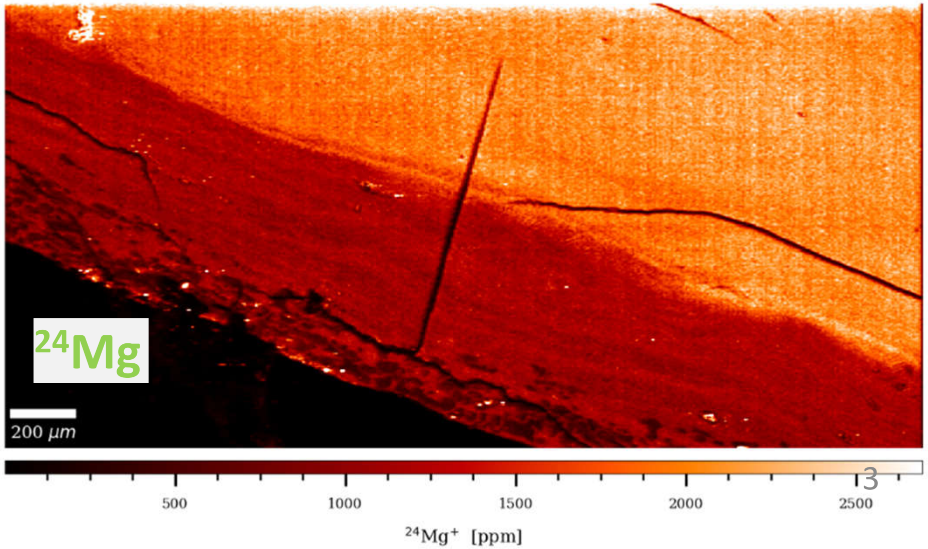

Næstved 211C – Cellular Cementum

^{23}Na

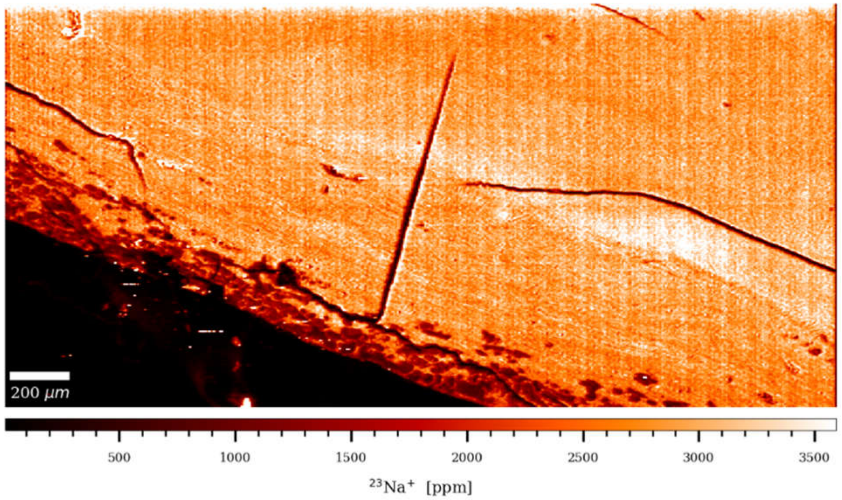

^{63}Cu

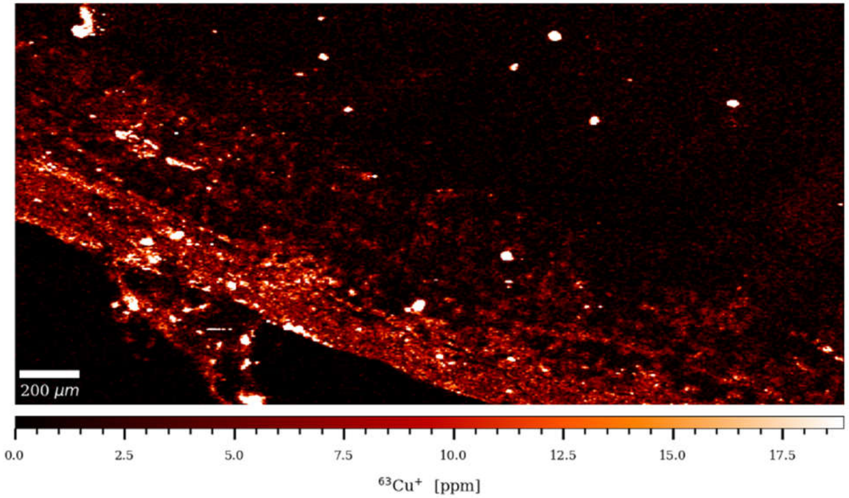

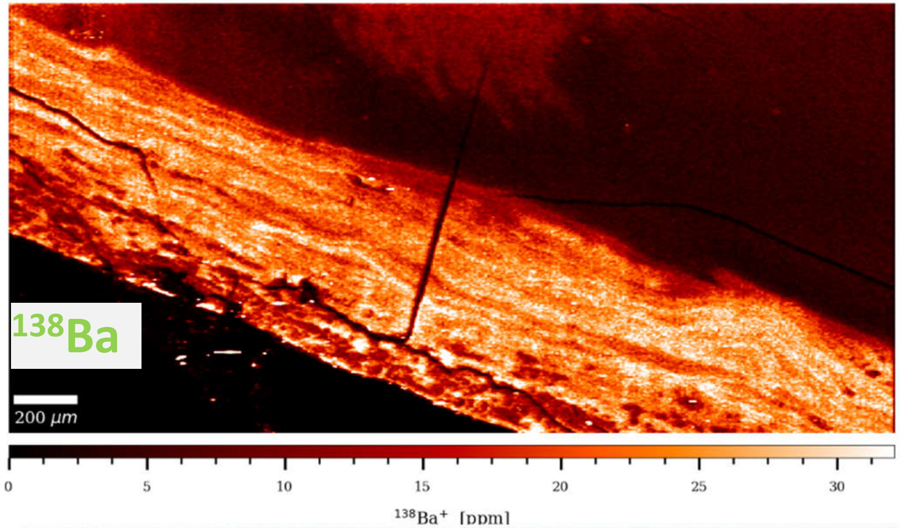

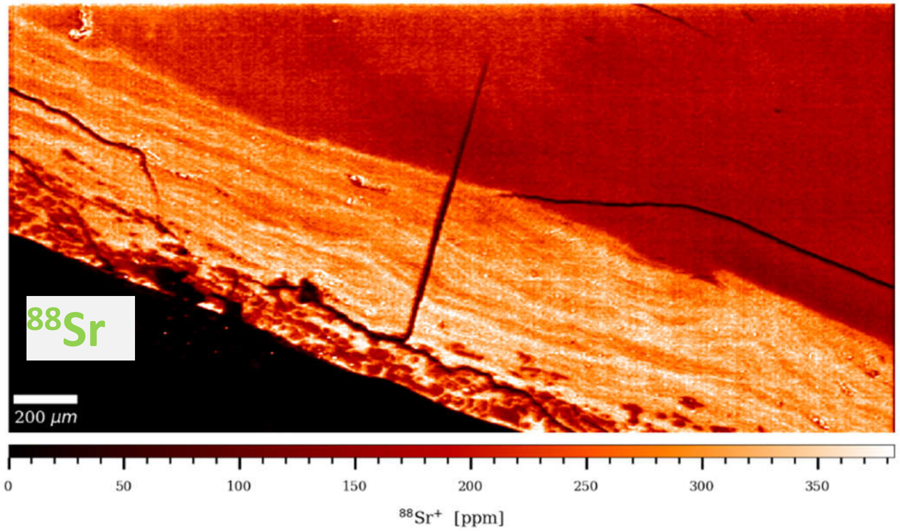

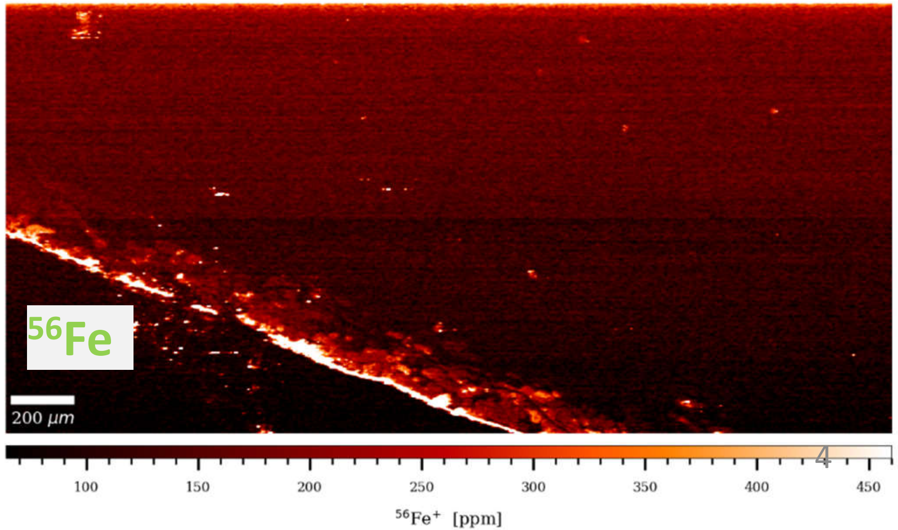

Næstved 211C – Cellular Cementum

^{75}As

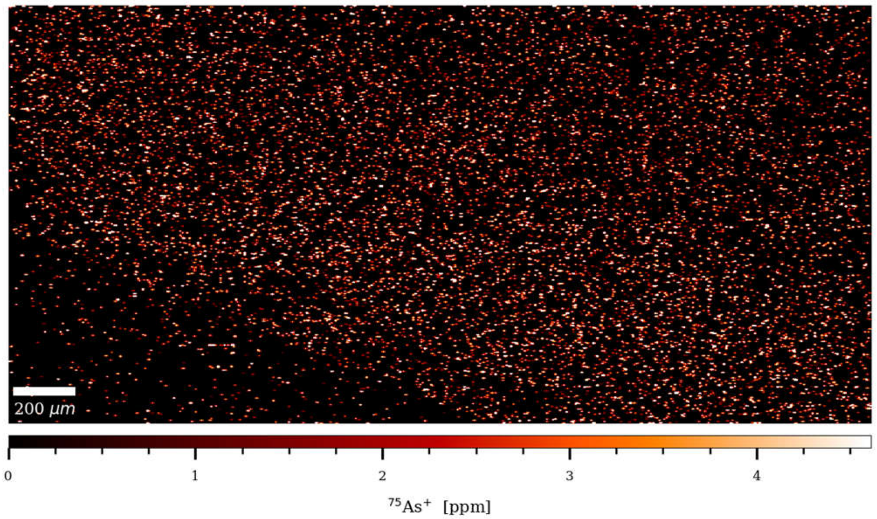

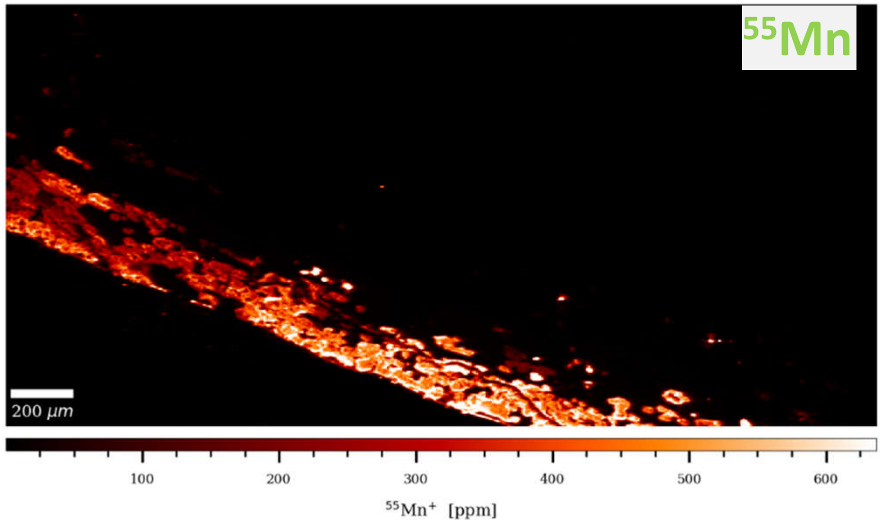

^{202}Hg

No detectable

mercury

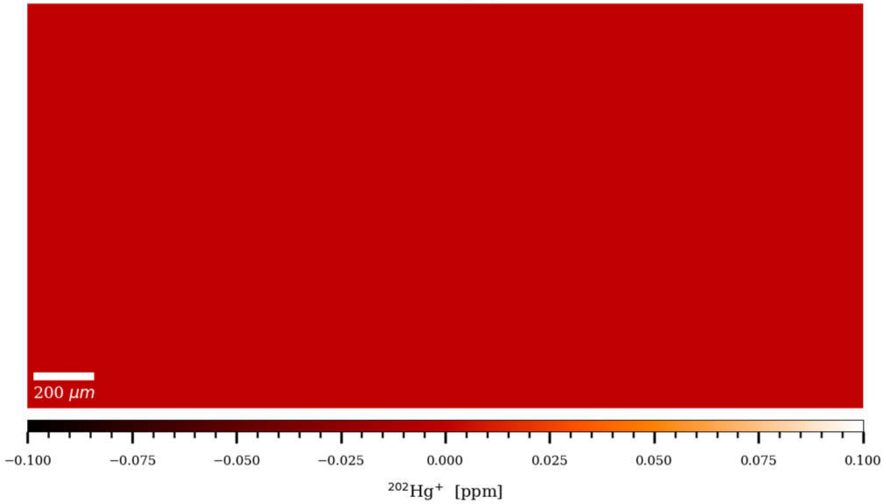

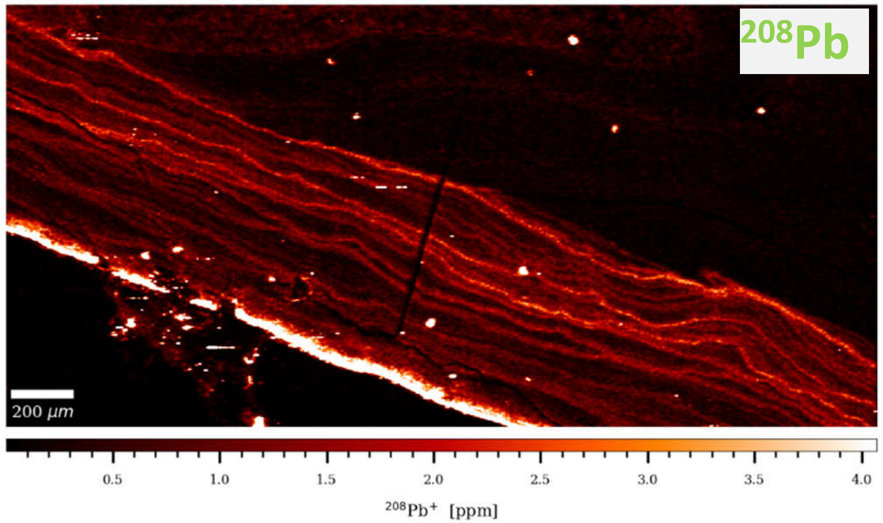

Odense 533 LLM1 – Cellular Cementum

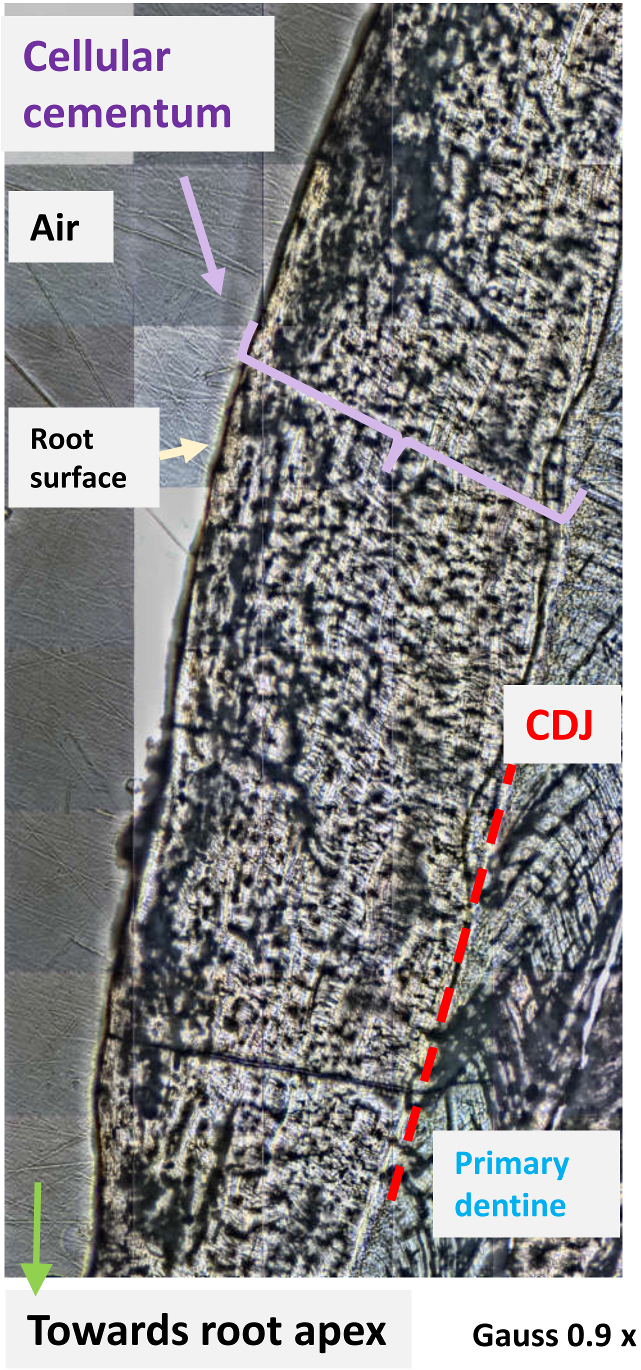

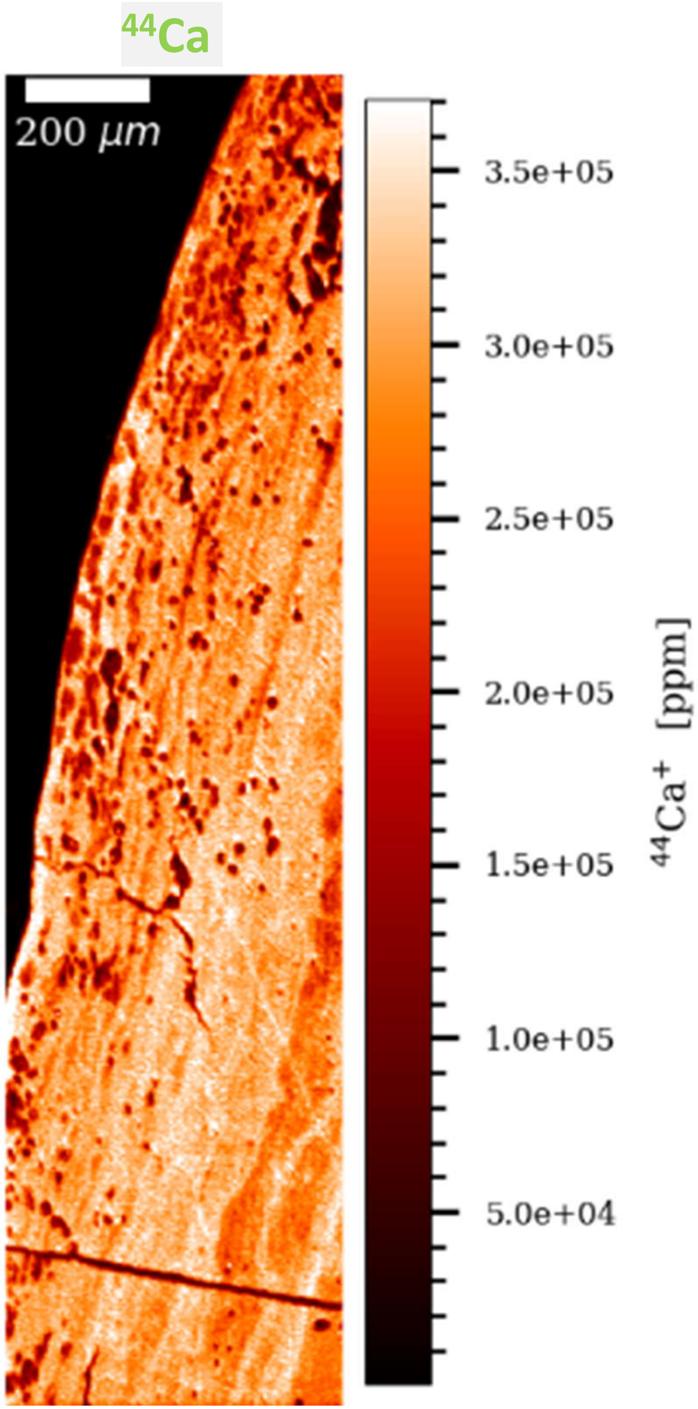

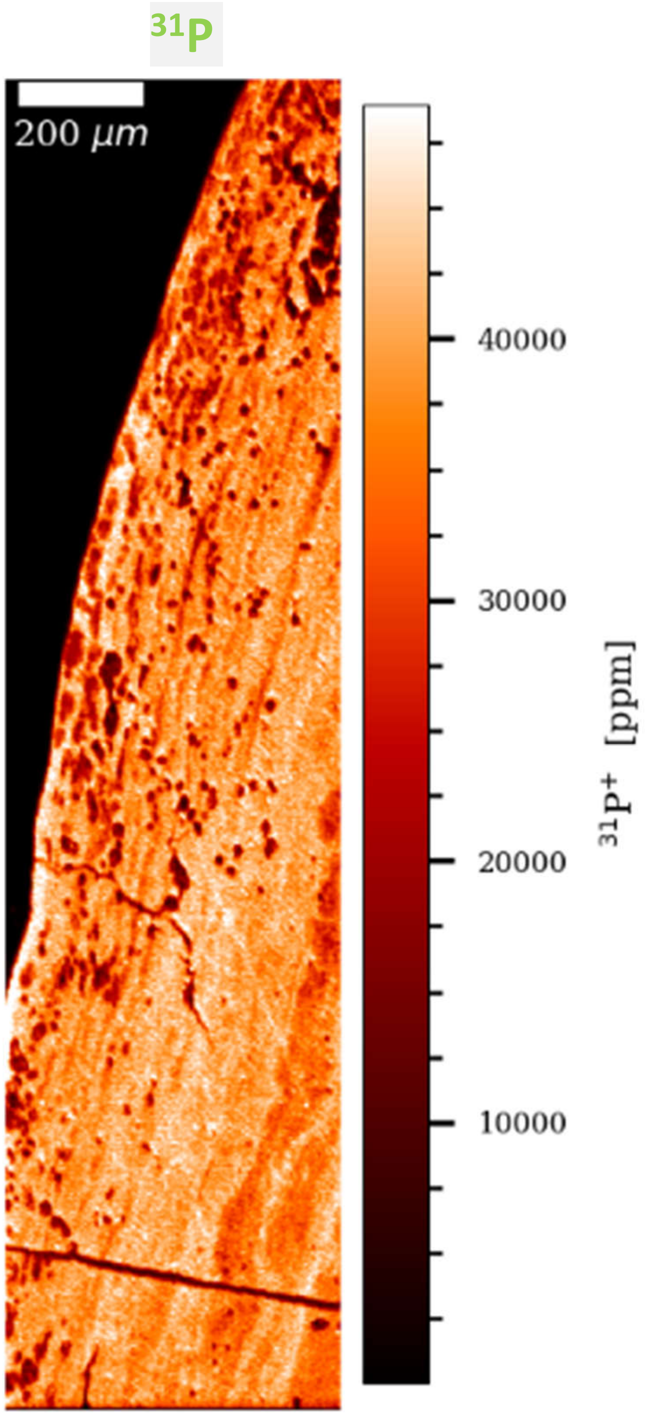

Gauss 0.9 x 0.9

Odense 533M – Cellular Cementum

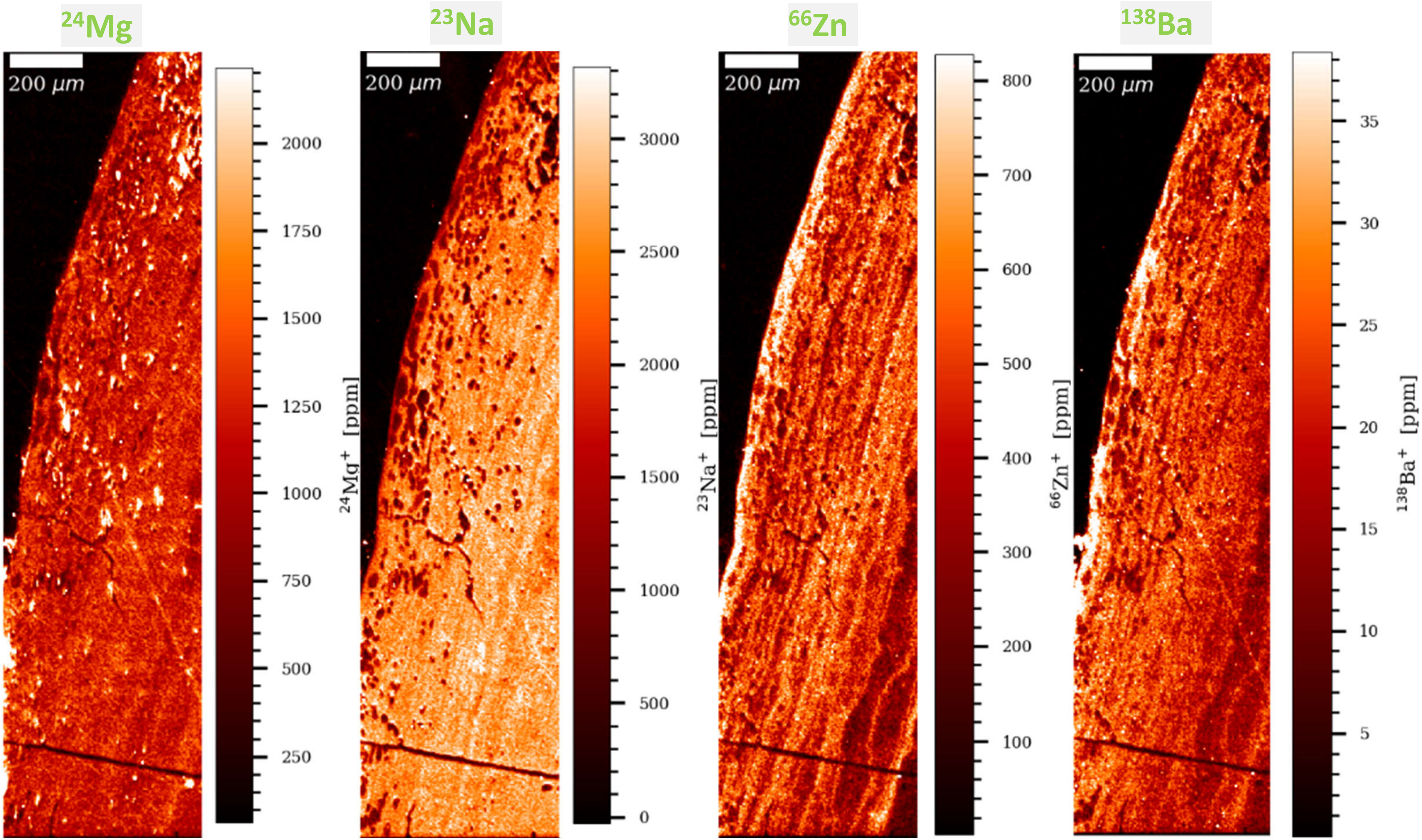

Odense 533M – Cellular Cementum

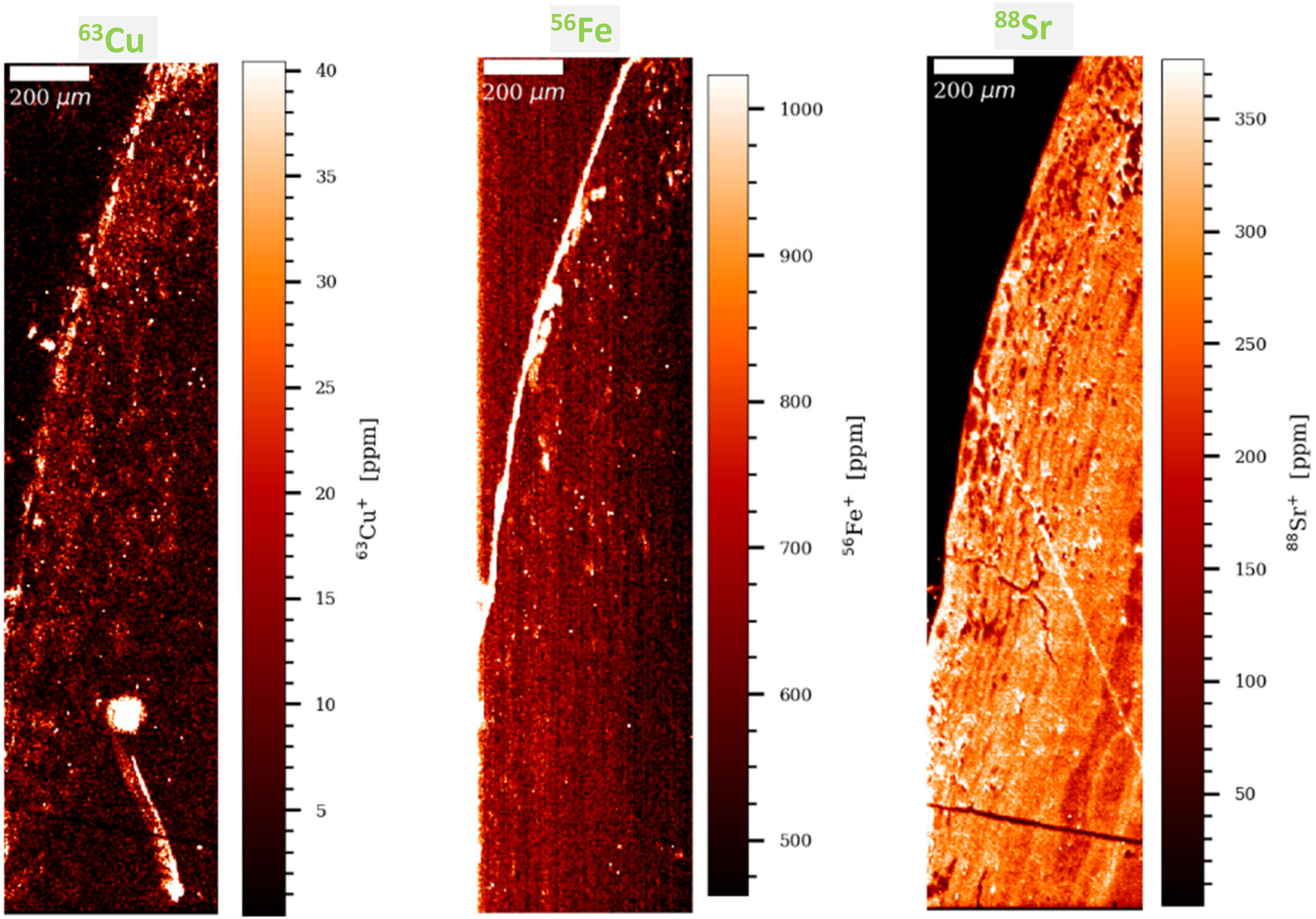

Odense 533M – Cellular Cementum

No detectable mercury or arsenic

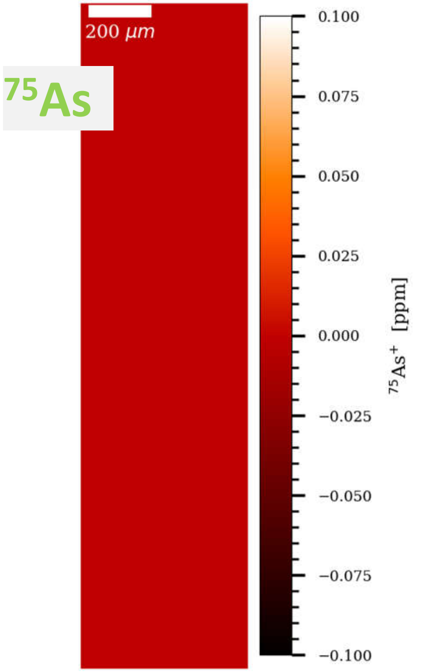

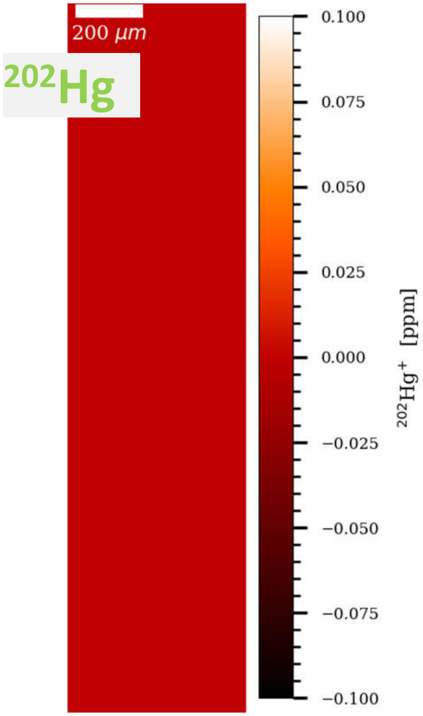

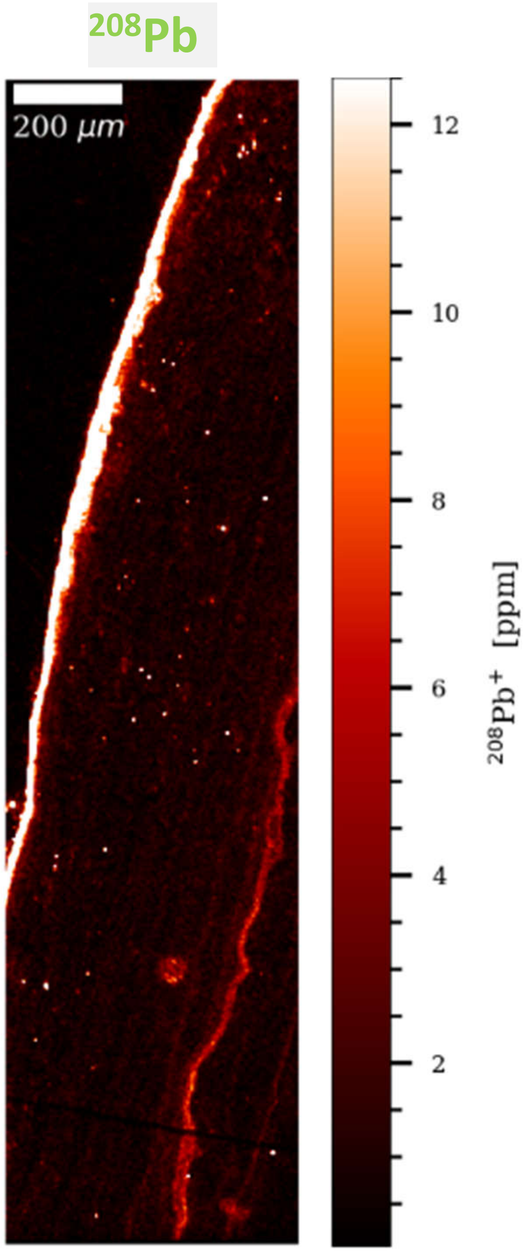

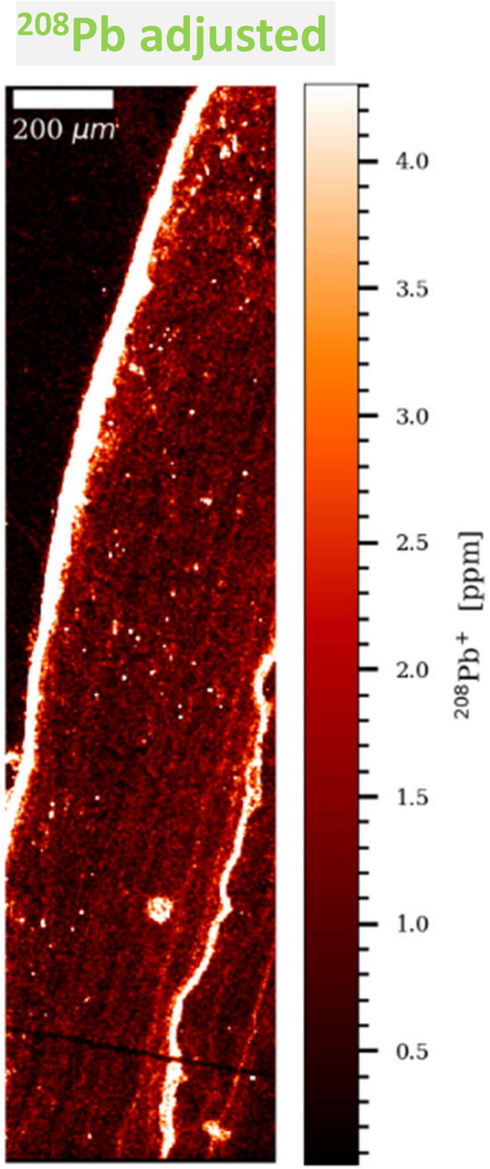

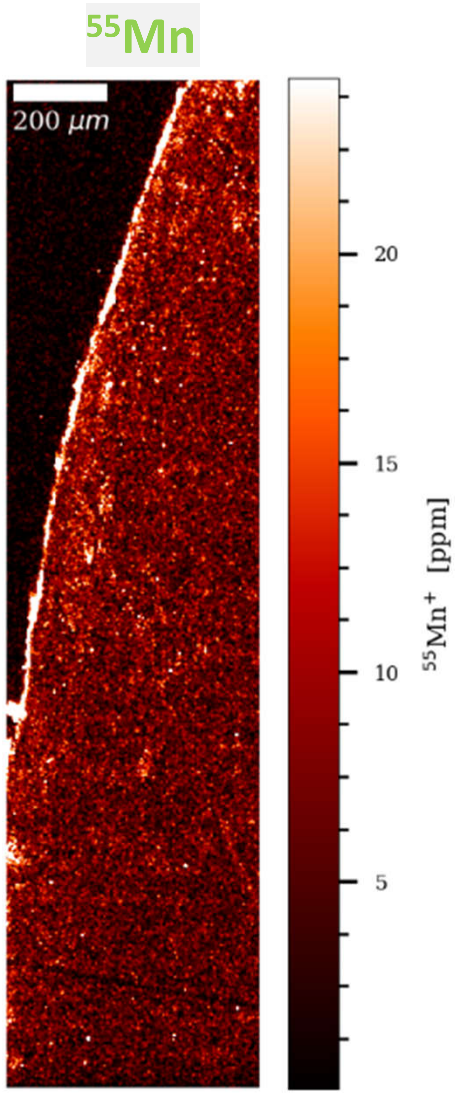

Odense 533 LLM1 – Acellular Cementum

Gauss 0.8 x 0.8

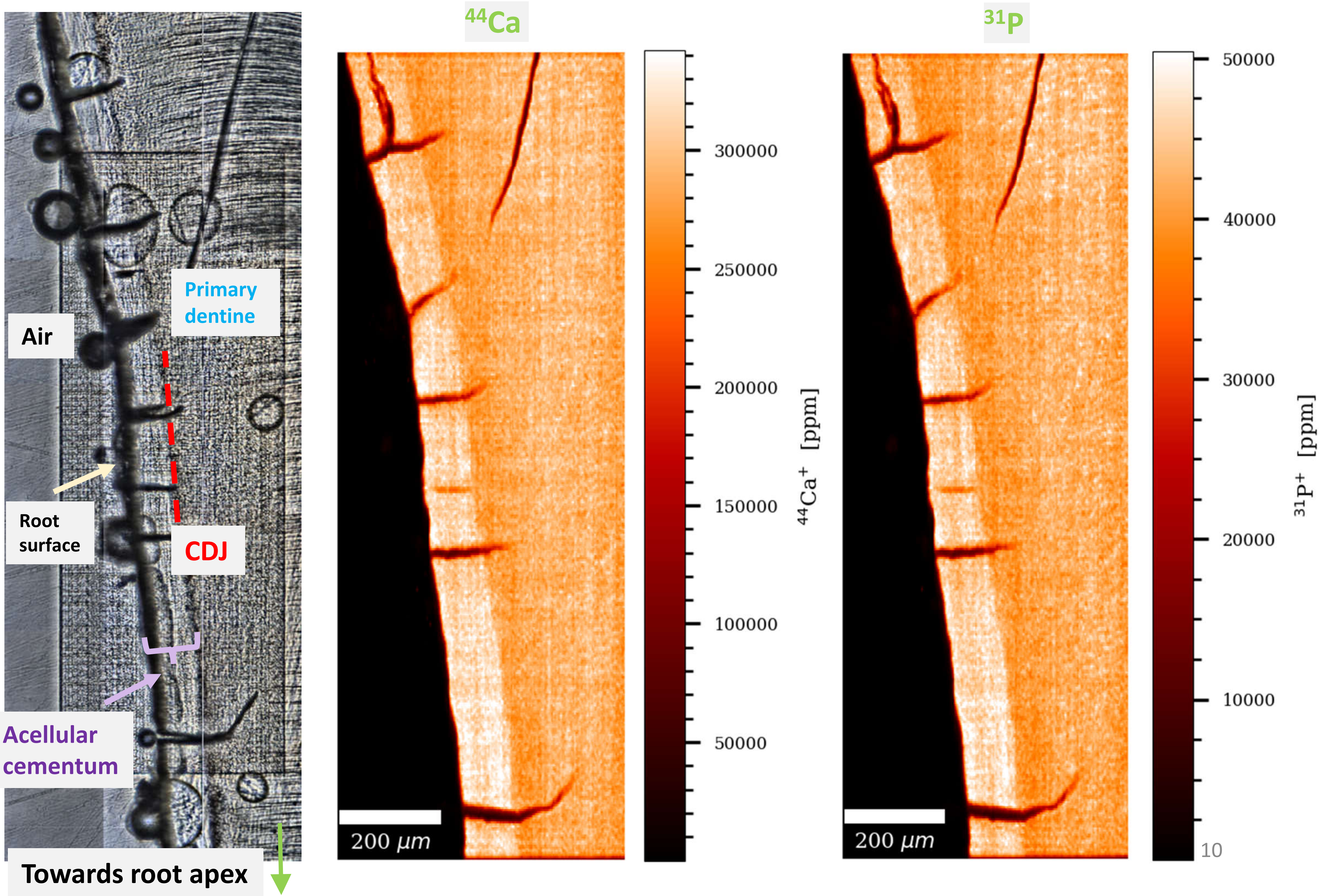

Odense 533M – Acellular Cementum

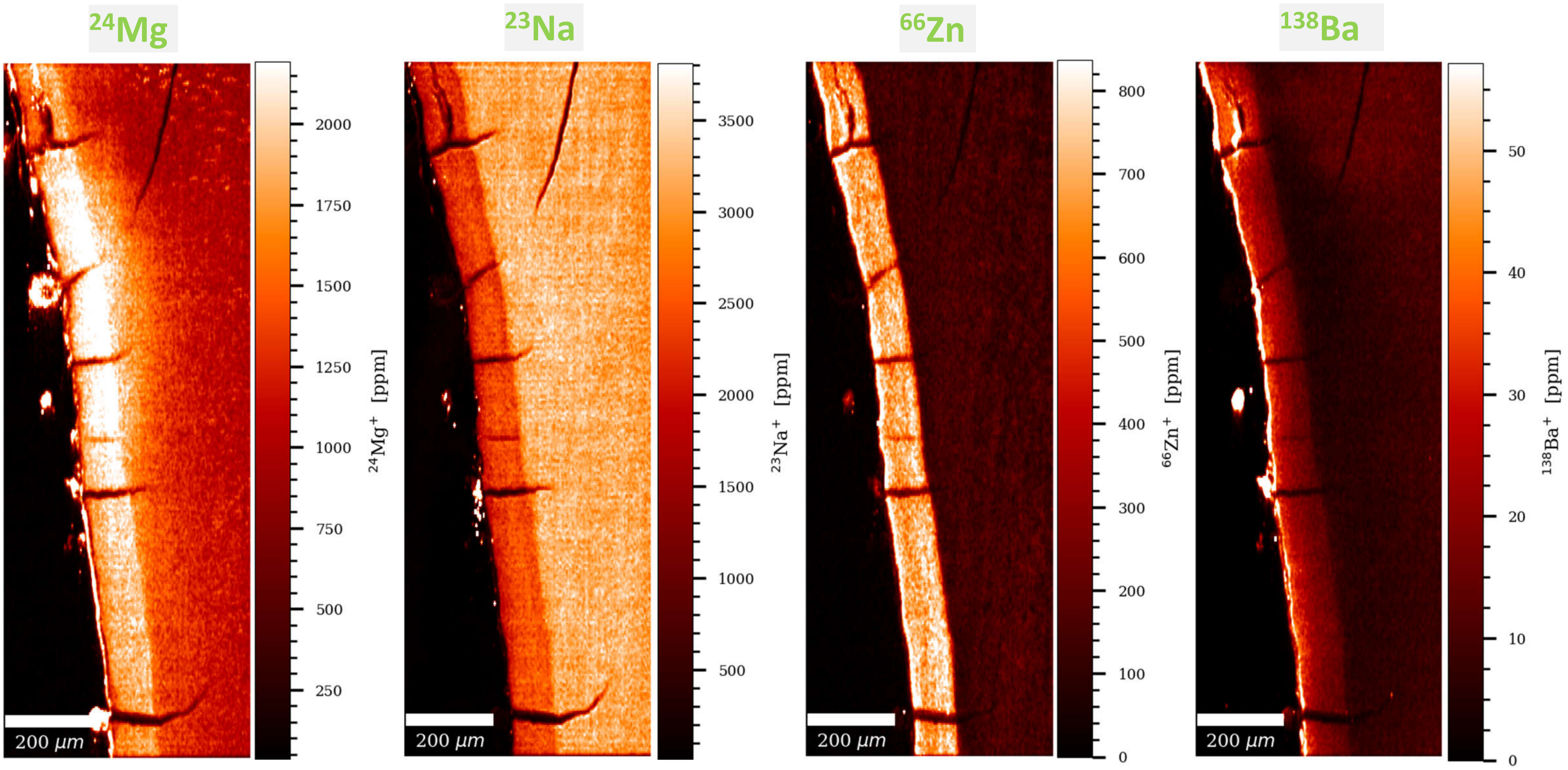

Odense 533M – Acellular Cementum

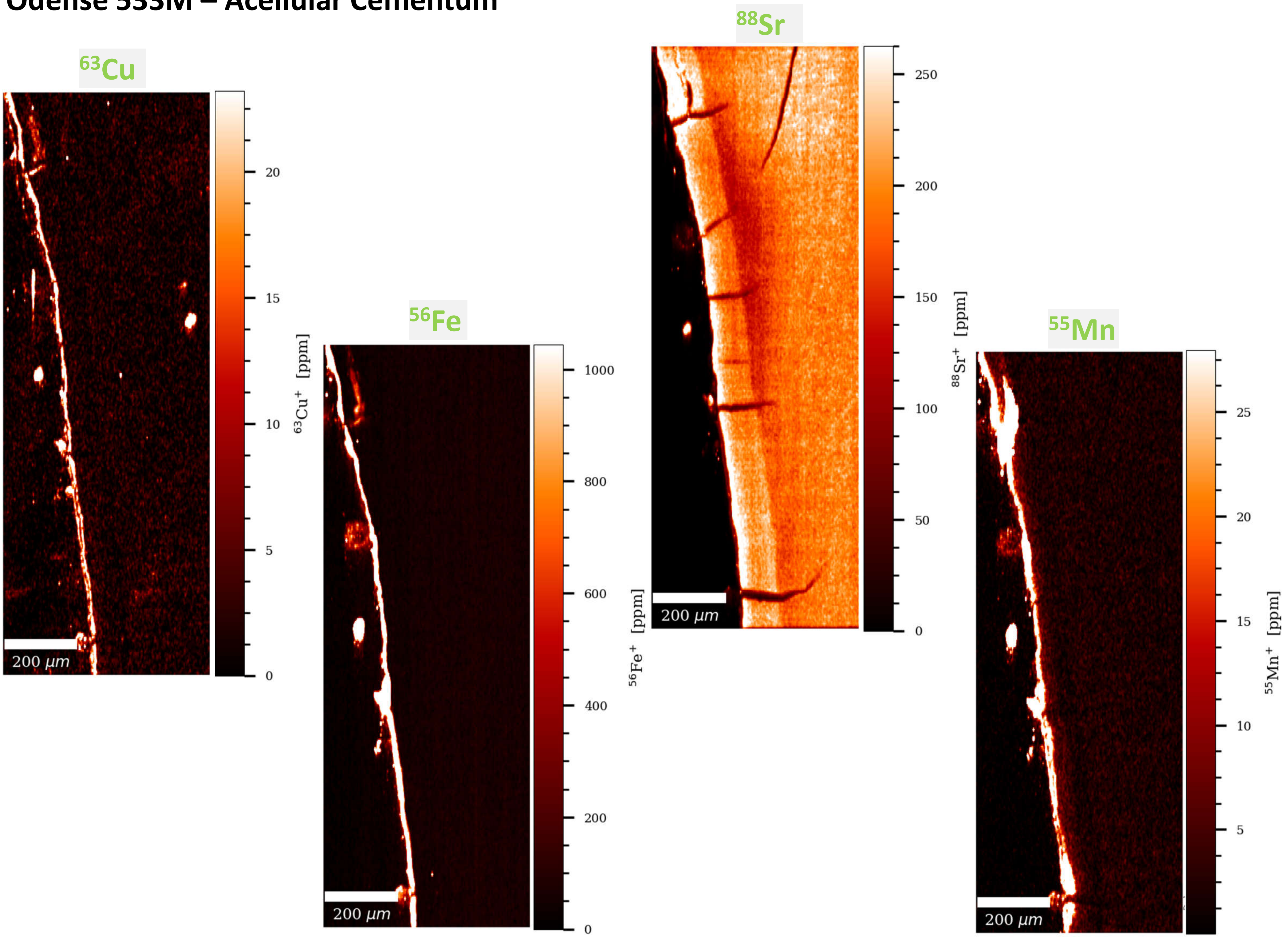

Odense 533M – Acellular Cementum

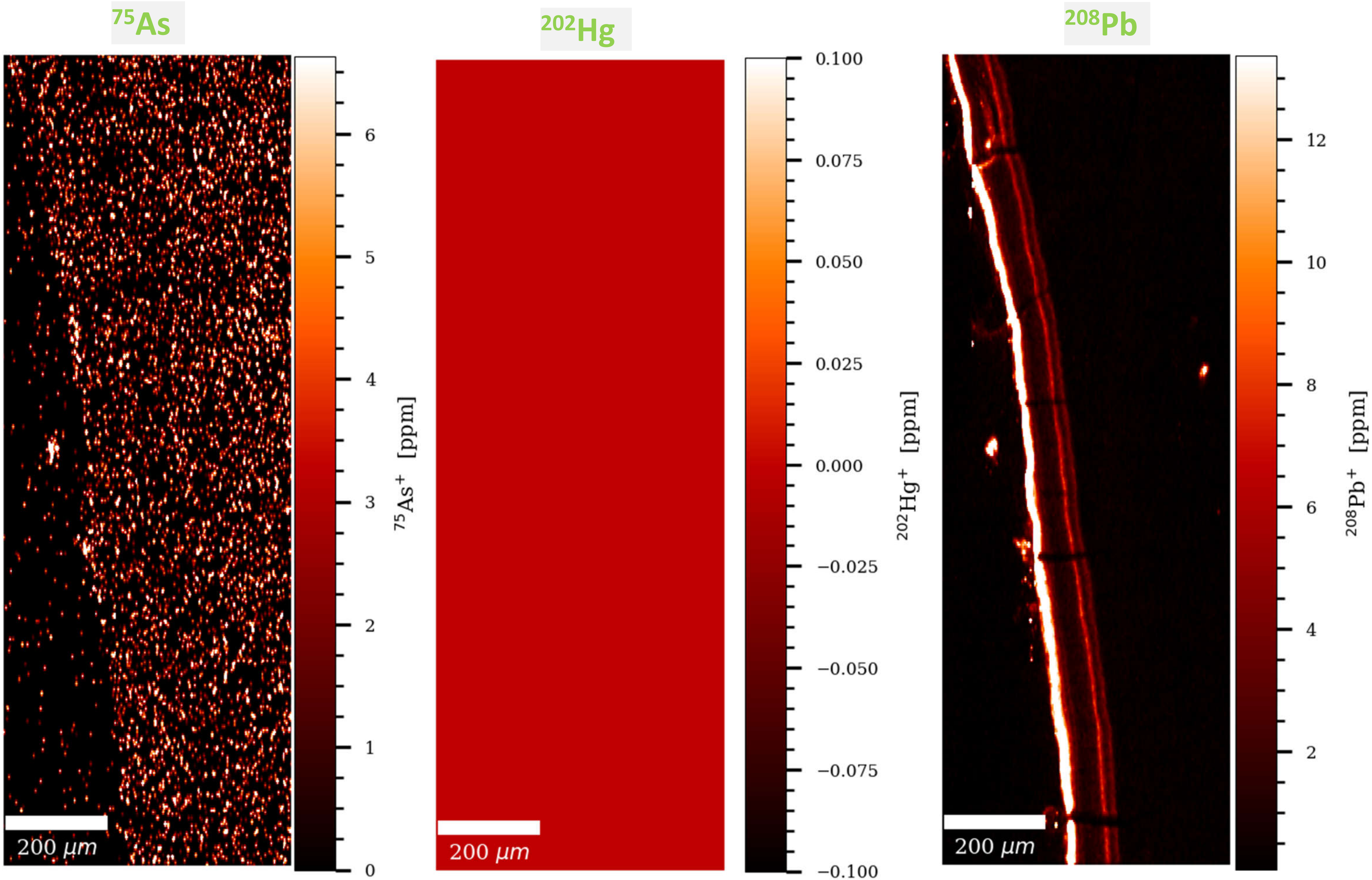

No detectable mercury

Odense 533 LLM1 – Secondary Dentine

Gauss 0.8 x 0.8

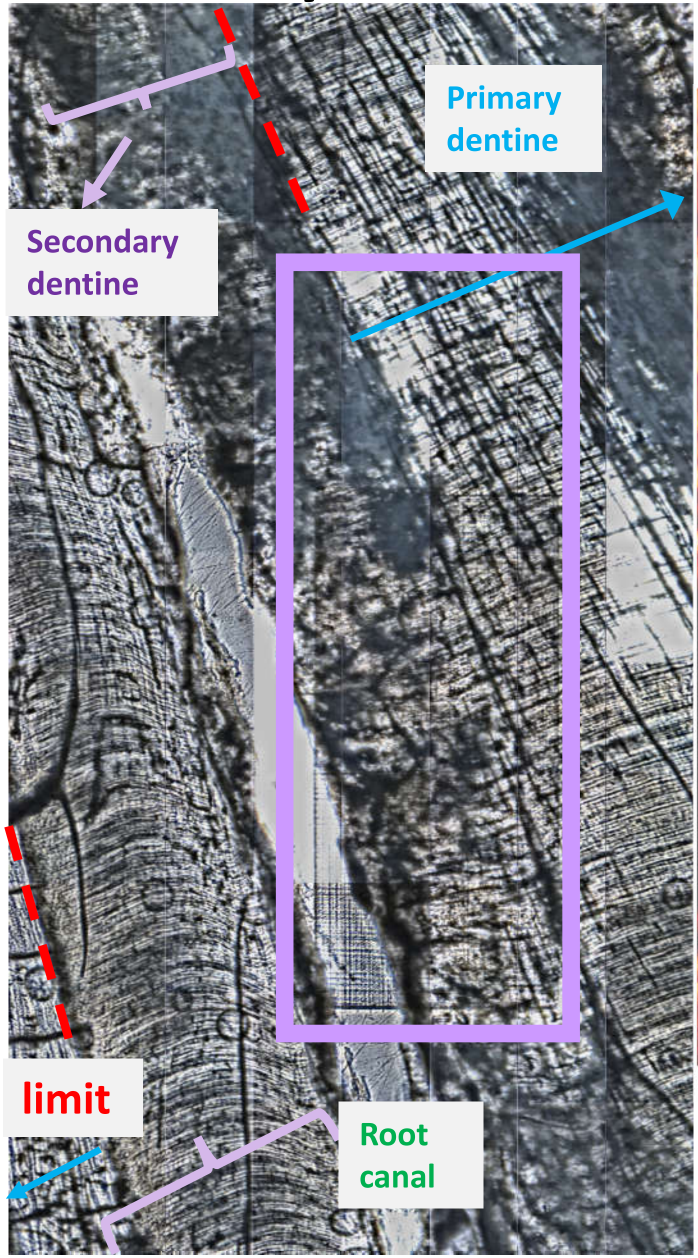

Towards root apex

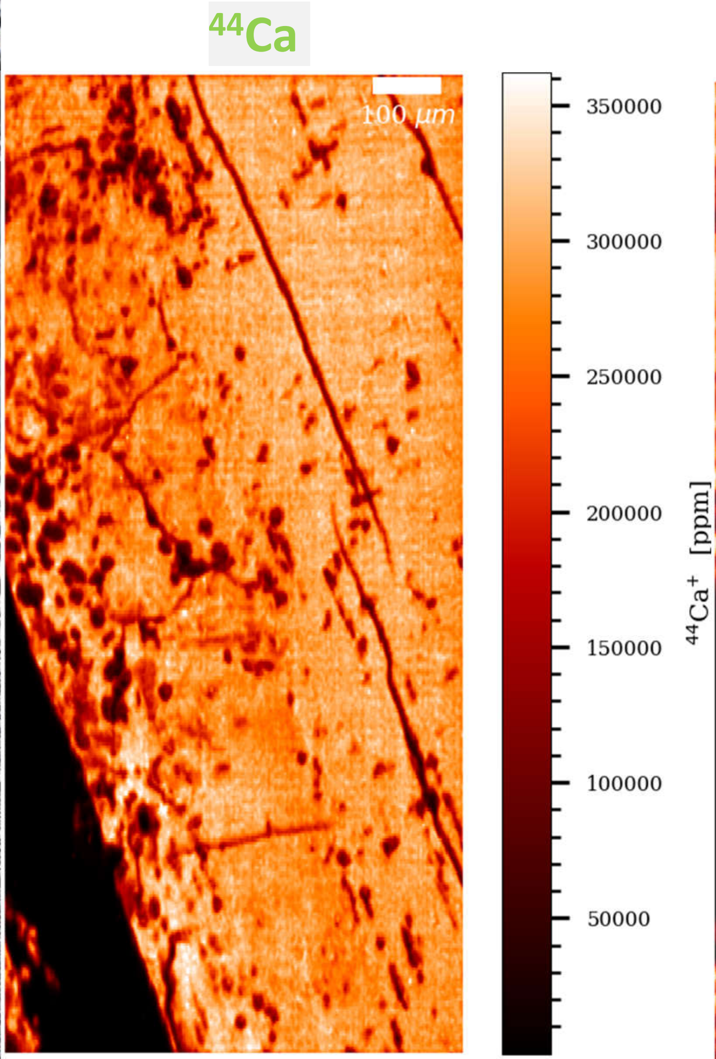

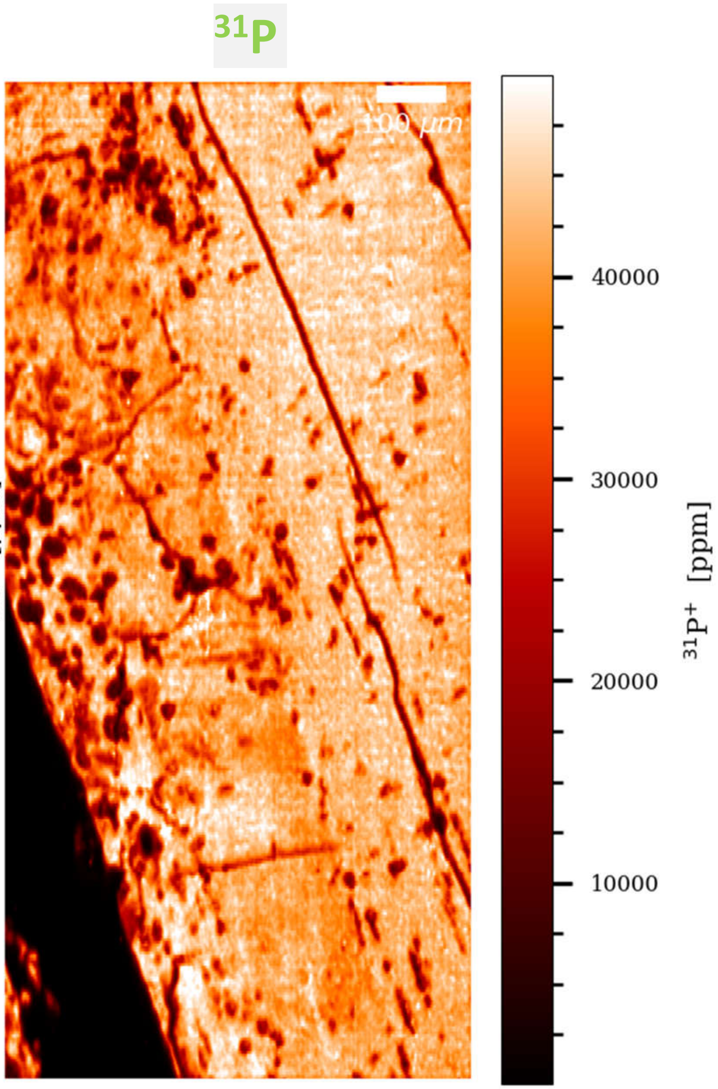

Odense 533M – Secondary Dentine

^{66}Zn

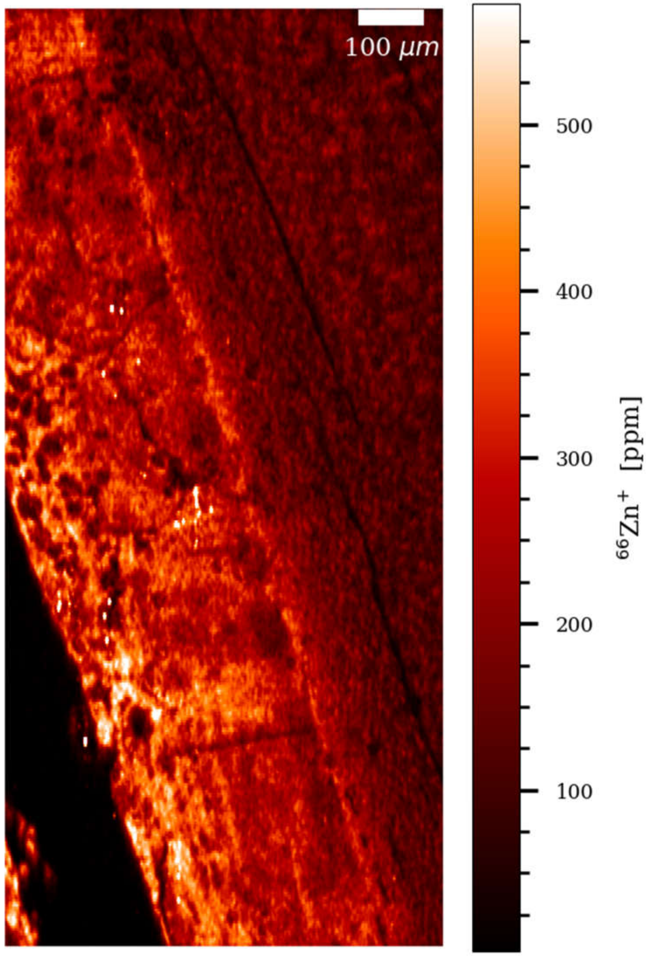

^{23}Na

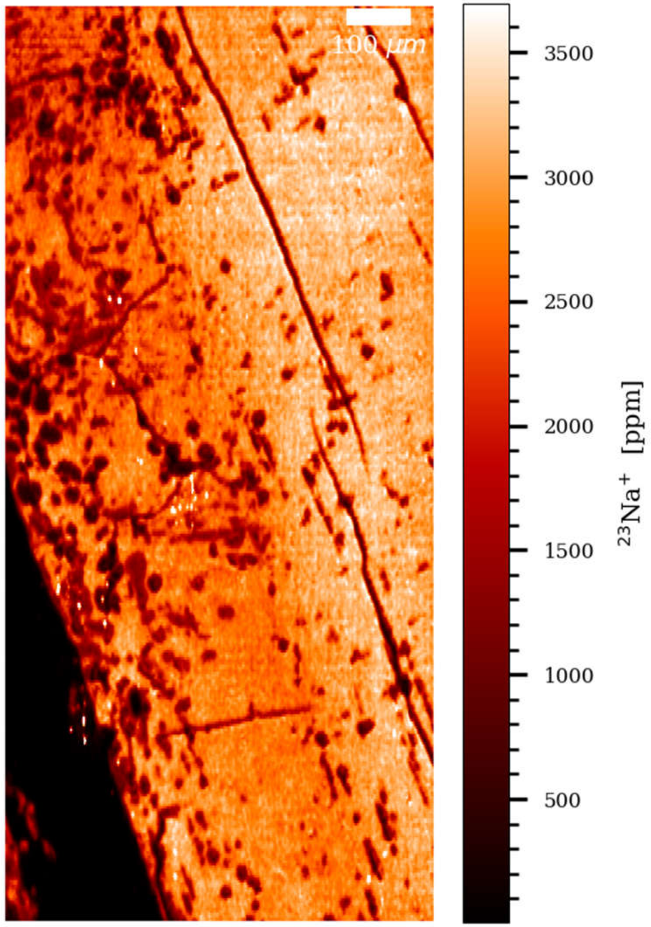

^{138}Ba

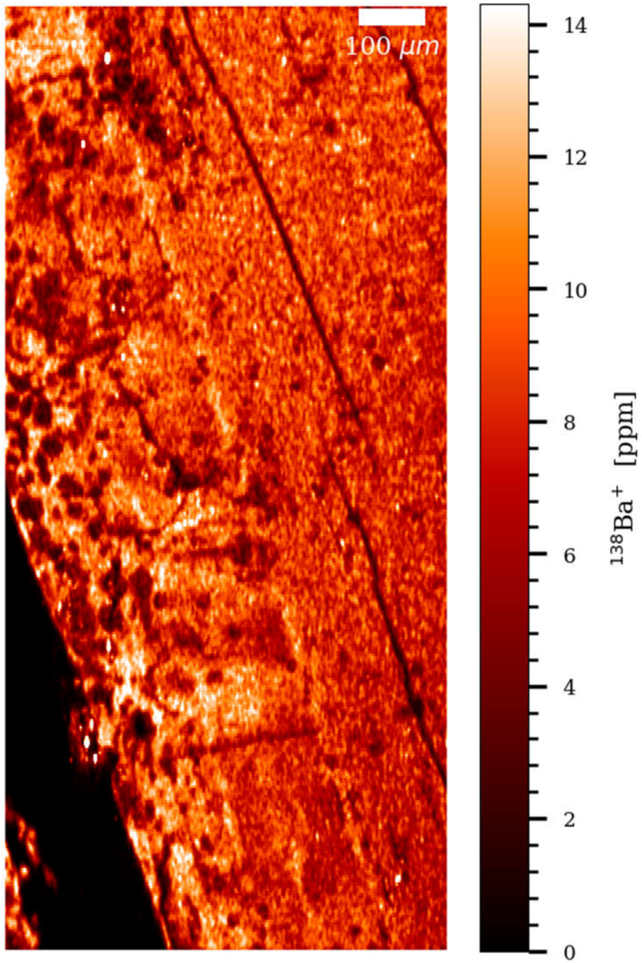

Odense 533M – Secondary Dentine

^{88}Sr

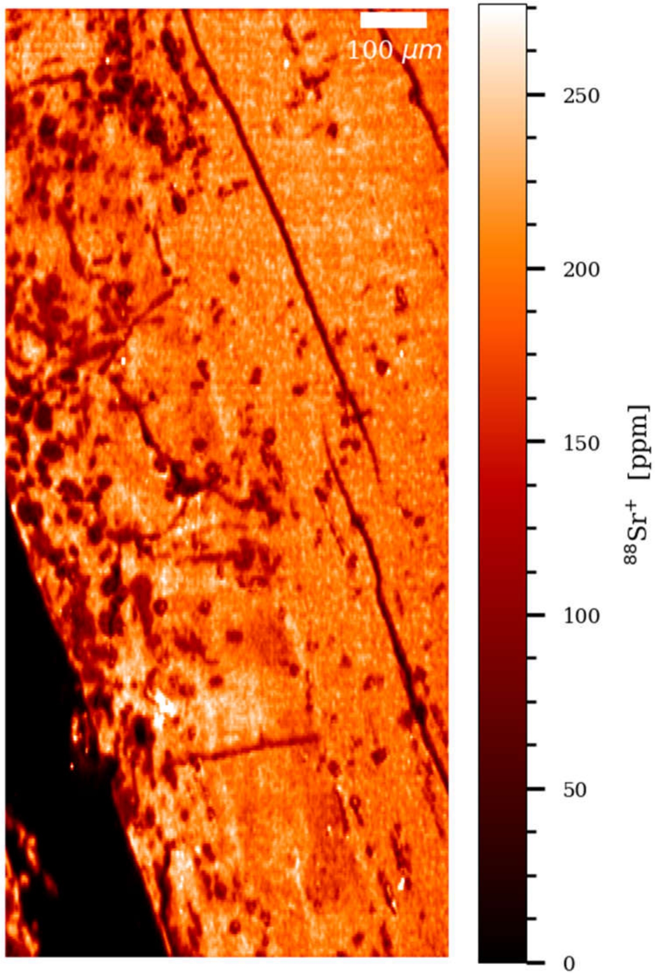

^{56}Fe

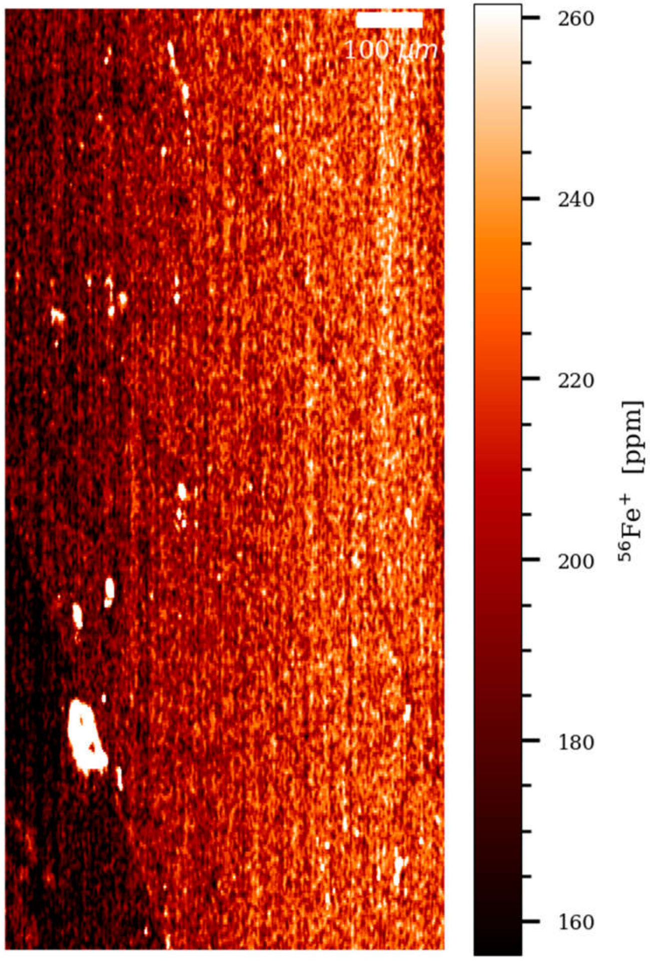

^{24}Mg

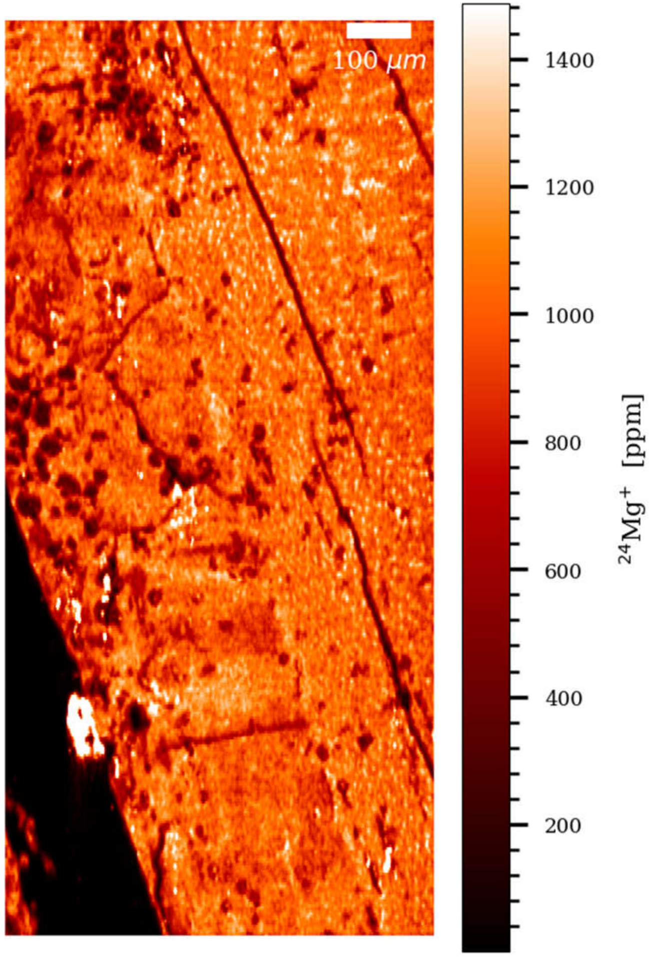

Odense 533M – Secondary Dentine

^{63}Cu

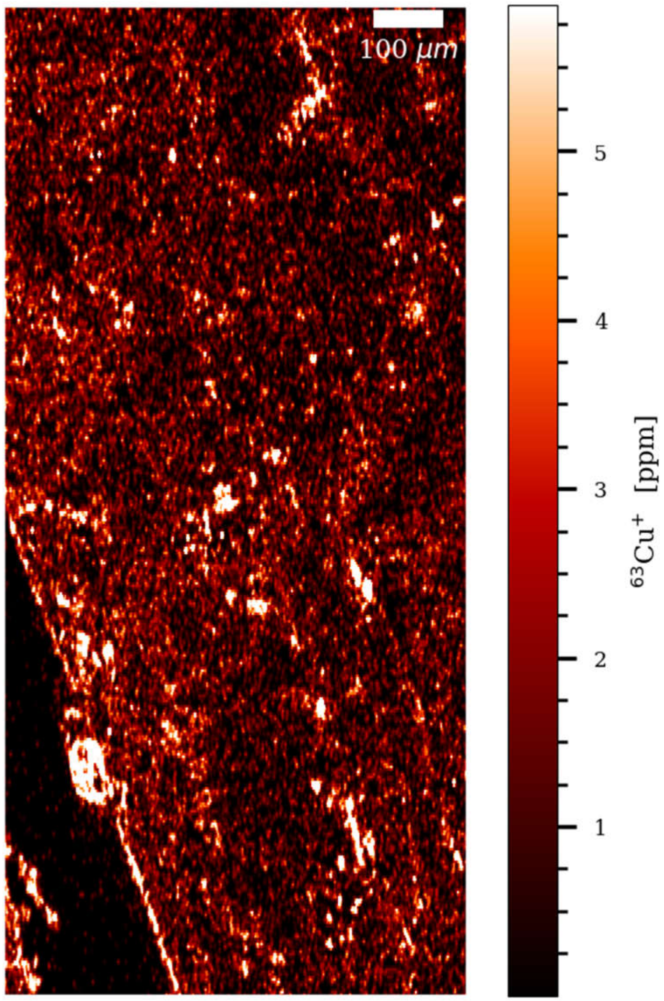

^{75}As

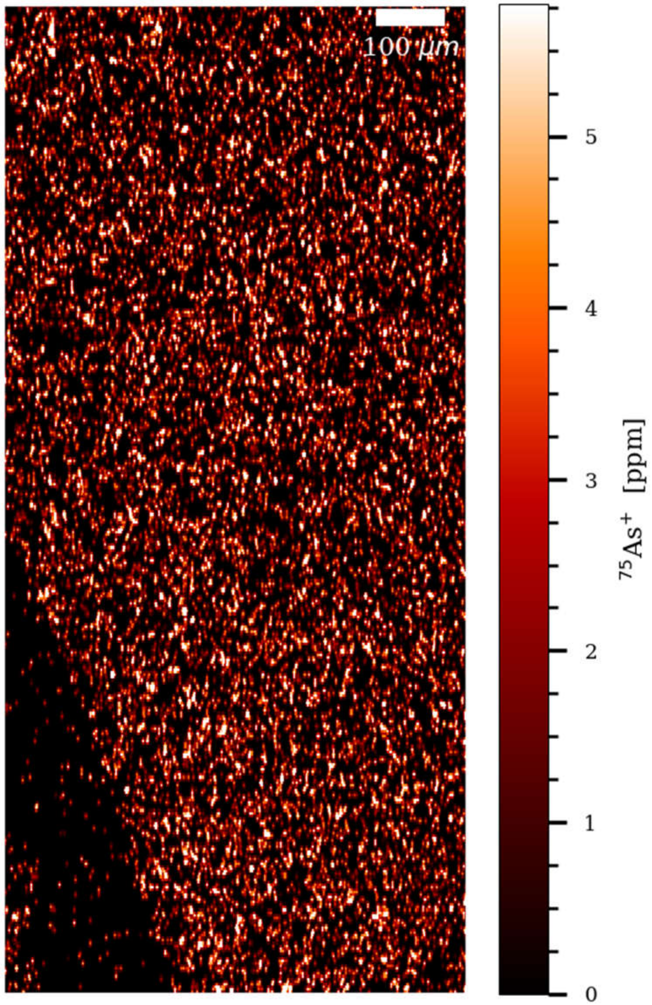

Odense 533M – Secondary Dentine

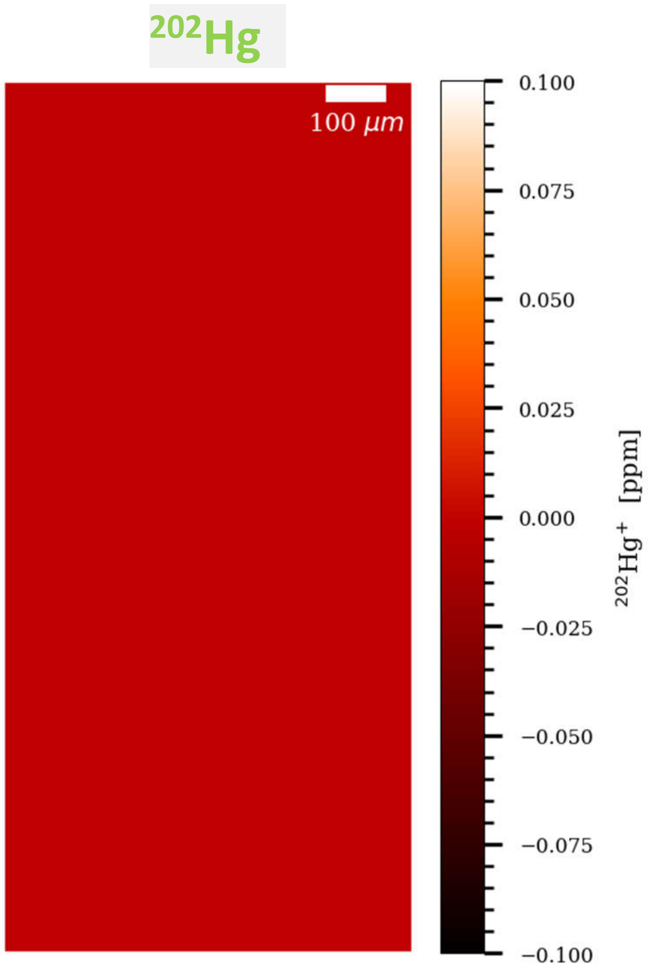

No detectable mercury

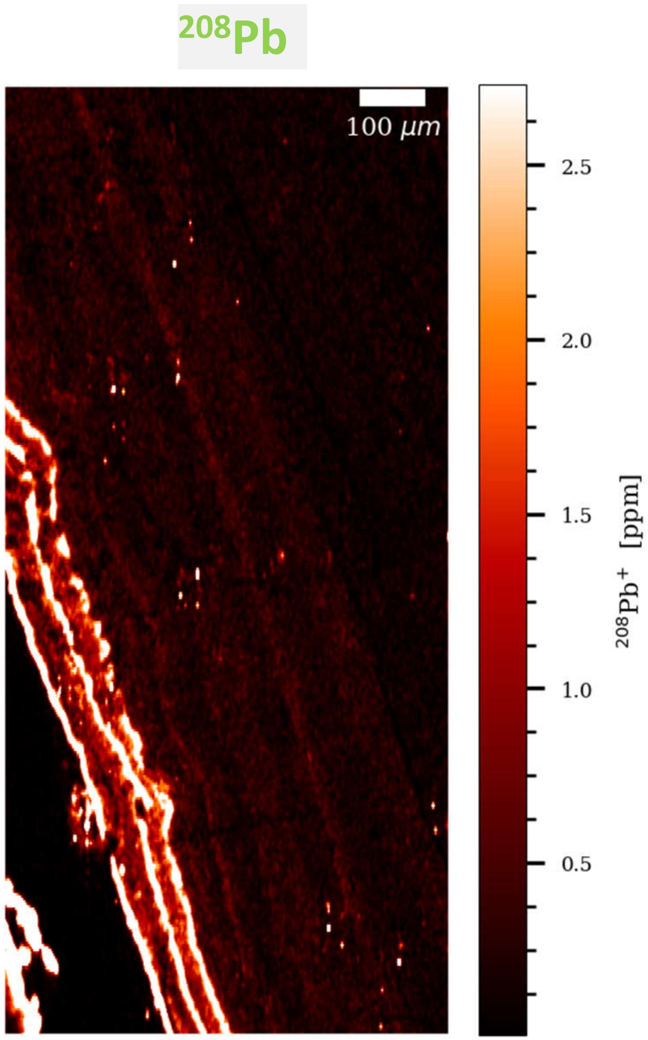

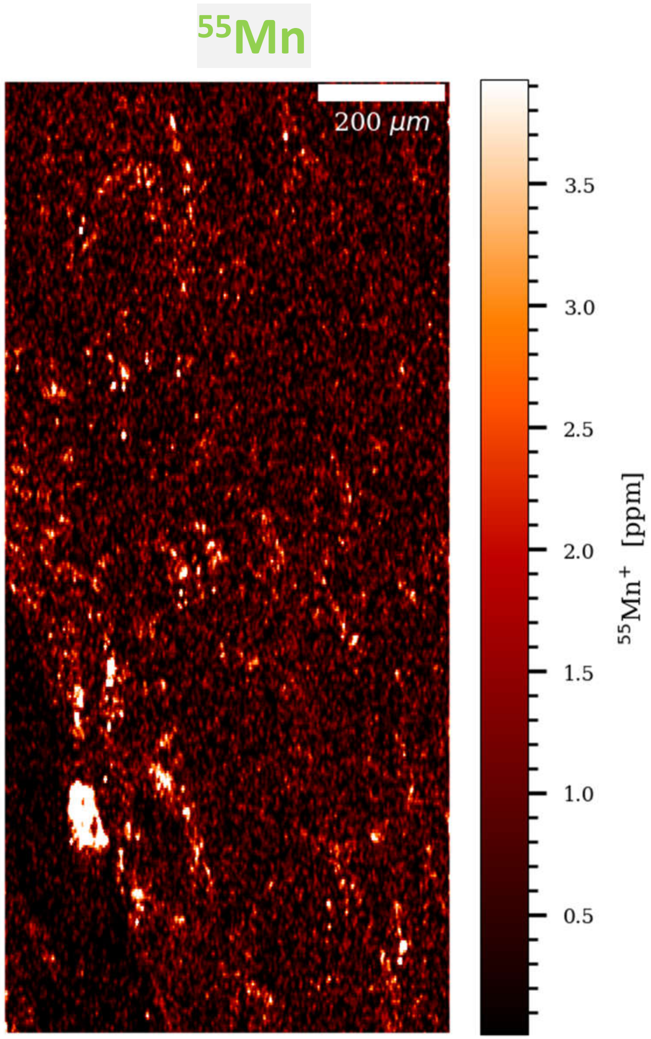

Supplement: Supplementary file 1 [file biology-12-00184-s001.zip › biology-2121048-Supplementary Material/Brozou_et_al_File_S3.pdf]
